# Atropisomeric Properties of *N*-Acyl/*N*-Sulfonyl 5*H*-Dibenzo[*b*,*d*]azepin-7(6*H*)-ones

**DOI:** 10.1021/acs.joc.1c00594

**Published:** 2021-05-17

**Authors:** Takuya Namba, Mayuno Hotta, Hidetsugu Tabata, Kosho Makino, Tetsuta Oshitari, Hideaki Natsugari, Hideyo Takahashi

**Affiliations:** † Faculty of Pharmaceutical Sciences, Tokyo University of Science, 2641Yamazaki, Noda-shi, Chiba 278-8510, Japan; ‡ Faculty of Pharma Sciences, Teikyo University, 2-11-1 Kaga, Itabashi-ku, Tokyo 173-8605, Japan; § Graduate School of Pharmaceutical Science, The University of Tokyo, 7-3-1 Hongo, Bunkyo-ku, Tokyo 113-0033, Japan

## Abstract

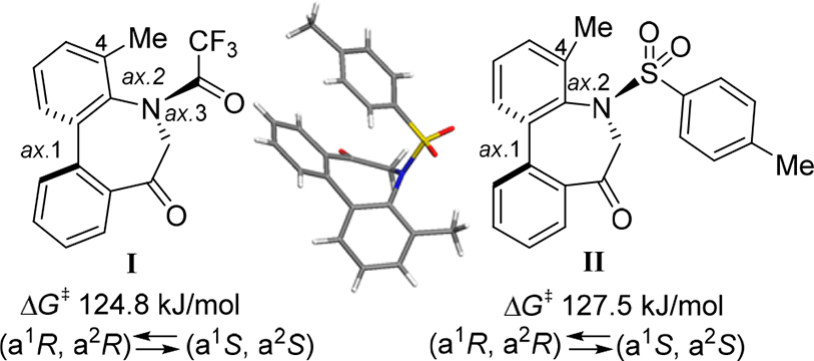

The stereochemistry of *N*-acyl/*N*-sulfonyl 5*H*-dibenzo[*b*,*d*]azepin-7(6*H*)-ones (**I**, **II**) was examined in detail by freezing the conformation with a methyl group at the C-4 of dibenzoazepine. Because the two axes (axis 1, axis 2) move together concertedly, **I** and **II** exist only as a pair of enantiomers [(a^1^*R*, a^2^*R*) and (a^1^*S*, a^2^*S*)], which was confirmed by X-ray analysis of **IIBc**. It was elucidated that the amide derivatives **I** exist in equilibrium with the *E*/*Z*-amide (100:2–100:34), which means that the exocyclic bond (axis 3) is not in concert with the endocyclic axes (axis 1, axis 2). For the preparation of 5*H*-dibenzo[*b*,*d*]azepin-7(6*H*)-one, the intramolecular Friedel–Crafts acylation of *N*-(1,1′)-biphenyl-2-yl-glycine derivatives was revisited. It was revealed that the electron-withdrawing property of the amino-protective group was a key to the success of seven-membered cyclization.

## Introduction

**Figure 1:**
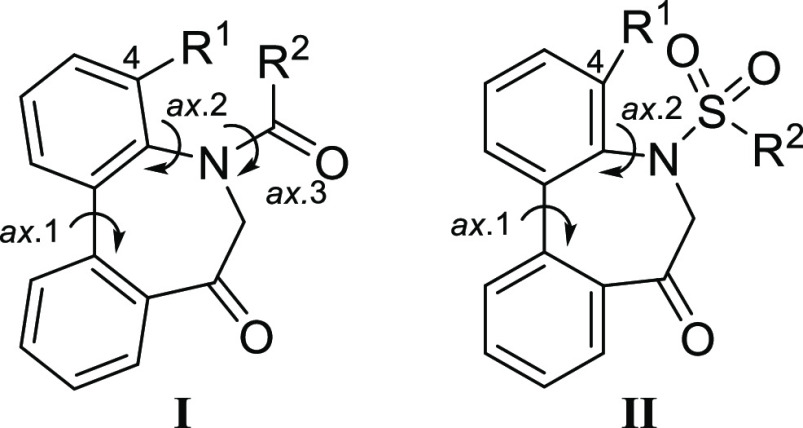
*N*-Acyl 5*H*-dibenzo[*b*,*d*]azepin-7(6*H*)-ones (**I**) and *N*-sulfonyl 5*H*-dibenzo[*b*,*d*]azepin-7(6*H*)-ones (**II**).

Recently, we have been interested in the conformational analysis of benzo-fused seven-membered-ring nitrogen heterocycles, which are found as the scaffolds of many drugs.^1^ Our continuing interest in the relationship between axial chirality and biological activity^2,3^ prompted us to examine the *N*-acyl/*N*-sulfonyl 5*H*-dibenzo[*b*,*d*]azepin-7(6*H*)-ones (**I**, **II**) (Figure 1), which were reported to have immunosuppressive effects by inhibiting the potassium channel (Kv1.3, IK-1) of T cells.^4^ The Ca^2+^-dependent potassium channel IK-1 and the voltage-gated potassium channel Kv1.3 in human T cells play a pivotal role during cell proliferation. Thus, inhibitors of these channels could be expected to be new drug candidates for treating autoimmune diseases such as rheumatoid arthritis and multiple sclerosis.^5^

The 5*H*-dibenzo[*b*,*d*]azepin-7(6*H*)-one moiety has dynamic axial chirality based on the sp^2^–sp^2^ axis arising from the biphenyl (axis 1). In addition, *N*-acylated derivatives (**I**) have another axial chirality around the Ar–NC(=O) (sp^2^–sp^2^) axis (axis 2) and *E*/*Z*-amide rotamers based on the N–C(=O) axis (axis 3). Thus, *N*-acylated derivatives (**I**) should exist in (a*S*)/(a*R*) axial isomers^6^ derived from axes 1 and 2, and *E*/*Z*-amide rotamers derived from axis 3. Similarly, the congener *N*-sulfonyl derivatives (**II**) were considered to have atropisomeric properties caused by the biphenyl (axis 1) and Ar–N(SO_2_) (axis 2).^7^ Their complex stereochemical structures are considered to constitute a key core structure of the immunosuppressive activity. Although the conformational change, i.e., ring flip, in molecules without a methyl substituent at the ortho position of the benzene ring (R^1^ = H) was anticipated to be too rapid for isolation of the stereoisomers at room temperature, molecules with 4-methyl (R^1^ = Me) were expected to freeze the conformations so that relatively stable stereoisomers could be separated. Such investigations should reveal the active structure (eutomer) exerting the inhibitory activity on the potassium channel (Kv1.3, IK-1) of T cell activity. Herein we describe a study of the conformational properties of the *N*-acyl/*N*-sulfonyl 5*H*-dibenzo[*b*,*d*]azepin-7(6*H*)-ones nucleus (**I**, **II**), and preliminary results of the blockade of the potassium channel. Through the synthesis, the intramolecular Friedel–Crafts acylation as a crucial step to provide the 5*H*-dibenzo[*b*,*d*]azepin-7(6*H*)-one nucleus was revisited. It was shown that the electron-withdrawing effect of the *N*-substituent of the amino acids affects the yield of cyclized compounds.

## Results and Discussion

### Preparation of 5*H*-Dibenzo[*b*,*d*]azepin-7(6*H*)-ones

For the preparation of 5*H*-dibenzo[*b*,*d*]azepin-7(6*H*)-one, we intended to utilize the intramolecular Friedel–Crafts acylation of *N*-(1,1′)-biphenyl-2-yl-glycine derivatives (**1**). The cyclization of the aryl amino acids appeared to be an obvious route. According to the procedure reported in a previous paper,^4^ the corresponding acid chlorides, prepared from *N*-(1,1′)-biphenyl-2-yl-glycine using thionyl chloride, were treated with anhydrous aluminum chloride. However, the reaction of *N*-(1,1′)-biphenyl-2-yl-glycine derivatives with an *N*-acetyl (**1Aa**), *N*-*p*-toluoyl (**1Ab**) provided complex mixtures (Table 1, entries 1, 2). Since further examination of the various reaction conditions was not rewarding, the pioneering work on Friedel–Crafts cyclization of aryl amino acids^8^ was reviewed. It was reported that Friedel–Crafts intramolecular acylation of aryl amino acids has little hope of succeeding because it gave a mixture of isoquinoline derivatives, oxazolonium halides, and phenanthridine derivatives as major products. Among them, Paterson and Procter reported that the *N*-*p*-tosylated aryl amino acid reacted to give the desired cyclic compound, although other *N*-acylated ones did not cyclize.^9^ In light of this, we focused on the amino-protective groups of the electron-withdrawing property and revisited the intramolecular Friedel–Crafts acylation of aryl amino acids.

**Table 1:**
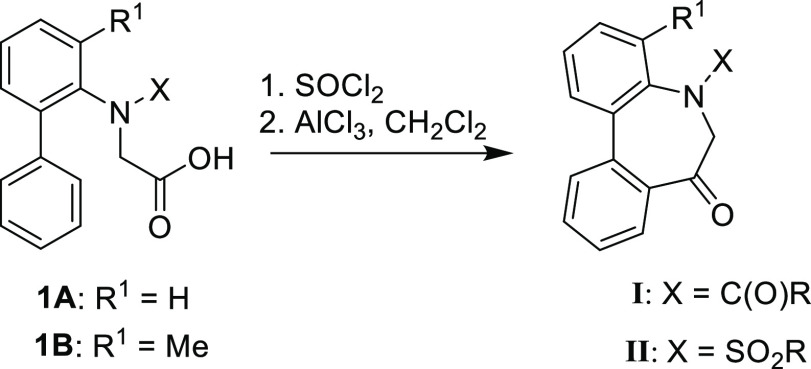
Intramolecular Friedel–Crafts Acylation of Aryl Amino Acids

Entry	Aryl amino acid^10^	R^1^	X		Yield (%)
1	**1Aa**	H	Acetyl		— (Complex mixture)
2	**1Ab**	H	*p*-Toluoyl		— (Complex mixture)
3	**1Ac**	H	*p*-Tosyl	**IIAc**	91
4	**1Ad**	H	Mesyl	**IIAd**	86
5	**1Ae**	H	*o*-Nosyl	**IIAe**	74
6	**1Af**	H	*p*-Nosyl	**IIAf**	69
7	**1Ag**	H	Trifluoroacetyl	**IAg**	97
8	**1Ah**	H	Methoxycarbonyl	**IAh**	84
9	**1Bc**	Me	*p*-Tosyl	**IIBc**	99
10	**1Bd**	Me	Mesyl	**IIBd**	72
11	**1Be**	Me	*o*-Nosyl	**IIBe**	83
12	**1Bf**	Me	*p*-Nosyl	**IIBf**	83
13	**1Bg**	Me	Trifluoroacetyl	**IBg**	99
14	**1Bh**	Me	Methoxycarbonyl	**IBh**	96

As expected, the cyclization of *N*-(1,1′)-biphenyl-2-yl-glycine derivatives with an *N*-*p*-tosyl group (**1Ac**) provided the corresponding 5*H*-dibenzo[*b*,*d*]azepin-7(6*H*)-one derivative (**IIAc**) in 91% yield (Table 1, entry 3). Similarly, *N*-mesyl (**1Ad**), *N*-*o*-nosyl (**1Ae**), and *N*-*p*-nosyl (**1Af**) were feasible for producing *N*-sulfonyl derivatives (**IIAd–f**) (Table 1, entries 4–6). Additionally, *N*-trifluoroacetyl (**1Ag**) and *N*-methoxycarbonyl (**1Ah**) also provided *N*-acyl derivatives (**IAg**, **IAh**) in good yields (Table 1, entries 7, 8). These results indicate that the electron-withdrawing property of the amino-protecting group is very important for this ring-closing reaction. Pleased with this, we further examined the cyclization of 4-methyl-substituted derivatives (**1Bc–h**). Despite the steric hindrance, 4-methyl-*N*-acyl/*N*-sulfonyl 5*H*-dibenzo[*b*,*d*]azepin-7(6*H*)-ones (**IIBc–h**) were obtained in good yields (Table 1, entries 9–14).

### Stereochemistry of *N*-Acyl-5*H*-Dibenzo[*b*,*d*]azepin-7(6*H*)-ones

**Figure 2:**
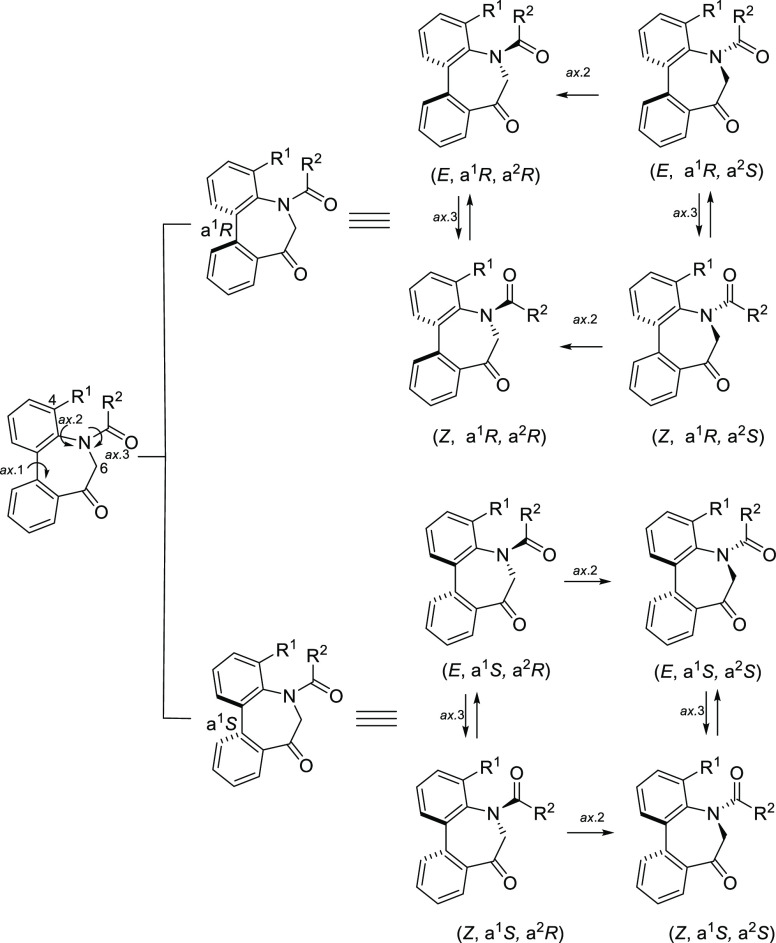
Conformational properties of *N*-acyl-5*H*-dibenzo[*b*,*d*]azepin-7(6*H*)-ones.

*N*-Acyl 5*H*-dibenzo[*b*,*d*]azepin-7(6*H*)-ones (**IA**) (R^1^ = H) and (**IB**) (R^1^ = Me) should have chirality based on the sp^2^–sp^2^ axis arising from the biphenyl (axis 1). In addition, another axial chirality arising from the sp^2^–sp^2^ axis of the benzene-amide bond (axis 2) should exist as well as *E*/*Z*-amide diastereomers around the N–C(=O) bond (axis 3). It was therefore anticipated that **IA** and **IB** exist as complicated stereoisomers. However, our preceding studies on this dibenzoazepinone nucleus revealed that axes 1 and 2 move concertedly to form the stable relative configuration.^11^ Thus, we presumed that the configuration of the enantiomers should be (a^1^*R*, a^2^*R*) and (a^1^*S*, a^2^*S*), respectively. Additionally, *E*/*Z*-amide diastereomers around the N–C(=O) bond (axis 3) were assumed to exist. The conformational properties of **IA** and **IB** are highlighted in Figure 2.

**Figure 3:**
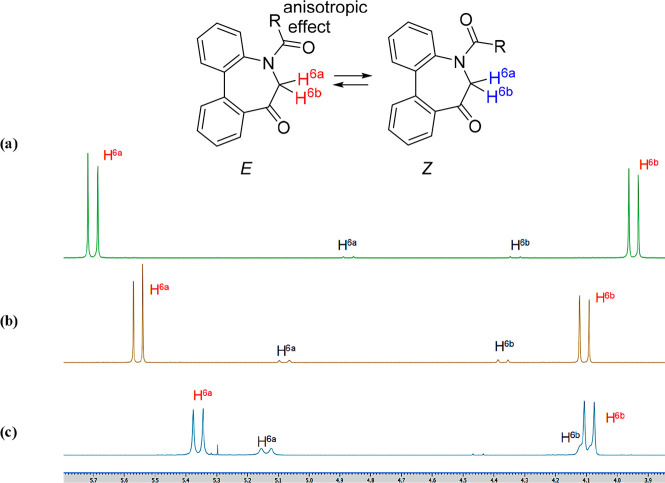
^1^H NMR spectra of **IAa** (a), **IAg** (b), and **IAh** (c).

First, the conformational properties of **IAg**-**h** (R^1^ = H) in the solution state were investigated precisely using ^1^H NMR spectroscopy (Figure 3). Compounds **IAg** and **IAh** were shown to exist as an equilibrium mixture of diastereomers in solution (CDCl_3_) at the ratios 100:7 [Figure 3b] and 100:30 [Figure 3c], respectively. In each spectrum, one of the two diastereotopic H-6 proton resonances in the major amide diastereomer is located at about 5.6 ppm (**IAg**) and 5.4 ppm (**IAh**), each 1.5 ppm, 1.3 ppm downfield from its partner, respectively. This downfield shift was also previously observed by Hassner^12a^ and Qadir et al.,^12b^ who ascribed the phenomenon to coplanarity between the exocyclic amide carbonyl bond and the equatorial proton on the adjacent carbon (C-6). Based on this anisotropic effect of the carbonyl group, we presumed that both **IAg** and **IAh** exist in the *E*-amide in preference to the *Z*-amide. It is clear that the two endocyclic axes (axes 1 and 2) move together concertedly, although the exocyclic axis (axis 3) does not move in concert with them. Viewed in this light, it was assumed that the seven-membered ring 5*H*-dibenzo[*b*,*d*]azepin-7(6*H*)-one exists only as a pair of enantiomers [(a^1^*R*, a^2^*R*) and (a^1^*S*, a^2^*S*)] without the presence of diastereomers [(a^1^*R*, a^2^*S*) and (a^1^*S*, a^2^*R*)].^11^

**Scheme 1:**
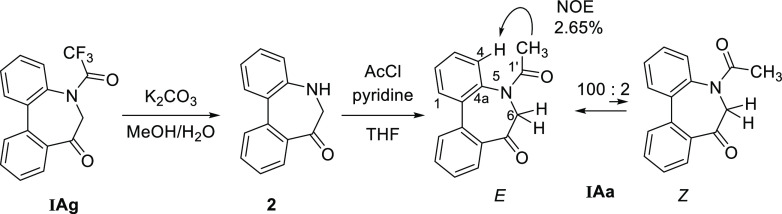
Preparation of **IAa** from **IAg** and Its *E*/*Z*-Amide Stereochemistry

In need of solid evidence for the determination of the *E*/*Z*-amide stereochemistry, investigation using NOE spectra seemed promising. However, the trifluoroacetyl group in **IAg** was not observed in ^1^H NMR, and the methoxy carbonyl group in **IAh** was inadequate because of the flexibility of the −O–Me bond. Thus, the *N*-acetylated compound **IAa** was prepared for this purpose from **IAg** through two steps (hydrolysis and acetylation) (Scheme 1).

**IAa** as well as **IAg**–**h** was shown to exist as an equilibrium mixture of *E*/*Z*-amide diastereomers in solution (CDCl_3_) at the ratio 100:2 [Figure 3a]. Additionally, one of the two diastereotopic H-6 proton resonances in the major amide diastereomer is located at about 5.7 ppm, 1.7 ppm downfield from its partner. Irradiation of the dominant CH_3_ resonance of acetyl in the major amide diastereomer led to 2.65% enhancement of the 4-H proton of benzene (Scheme 1). Therefore, the preference of the *E*-amide in **IAa** was determined. Based on this, the preference of the *E*-amide observed in **IAg** and **IAh** was confirmed. It was also revealed that **IBg**-**h** (R^1^ = Me) showing similar spectra (see Supporting Information) preferred the *E*-amide to the *Z*-amide in solution (Table 2).

**Table 2:**
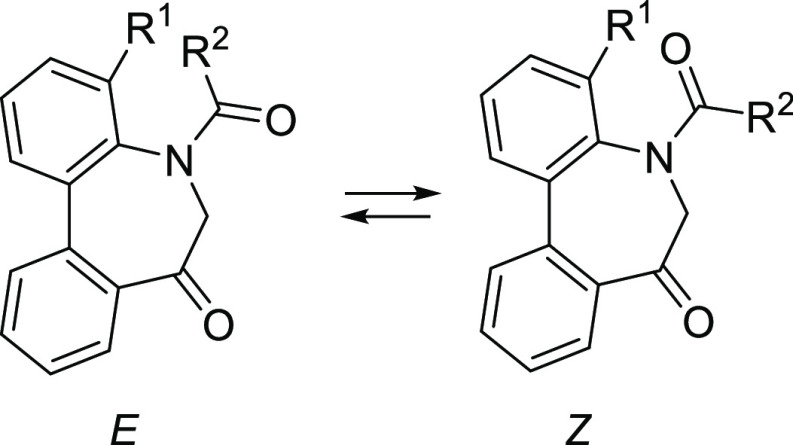
*E*/*Z* Equilibrium Ratio and Energy Differences of **IAa**, **IAg**, **IAh**, **IBg**, and **IBh** Based on ^1^H NMR and DFT Calculation^13^

				Energy difference (kJ/mol)	
	R^1^	R^2^	*E*/*Z* ratio (^1^H NMR at 296 K in CDCl_3_)	Experimental Δ*G*_Tc_	Calculated (DFT) Δ*G*_298_
**IAa**	H	Me	100:2	9.7	10.3
**IAg**	H	CF_3_	100:7	6.7	7.2
**IAh**	H	MeO	100:30	3.0	2.8
**IBg**	Me	CF_3_	100:17	4.4	3.5
**IBh**	Me	MeO	100:34	2.7	3.0

a Unfortunately, each *E*/*Z*-amide of *N*-acyl 5*H*-dibenzo[*b*,*d*]azepin-7(6*H*)-ones (**IAa**, **IAg**-**h**, **IBg**-**h**) was not separated by HPLC at rt.

**Figure 4:**
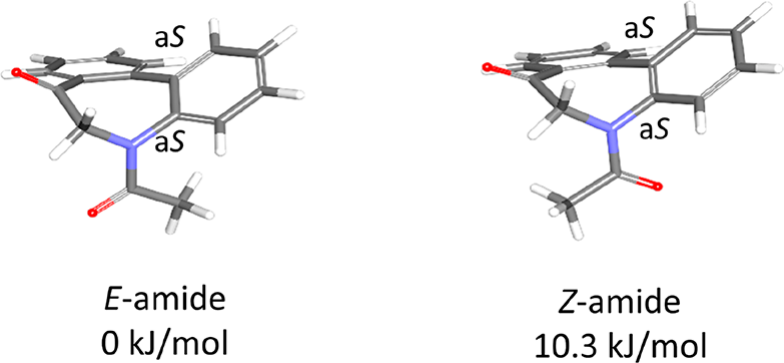
Conformers of **IAa** with the (a^1^*S*, a^2^*S*) stereochemistry and free energy difference (*ΔG*_298_) calculated by the DFT method.

Furthermore, the following computational studies were carried out to study the conformational preferences of *N*-acyl 5*H*-dibenzo[*b*,*d*]azepin-7(6*H*)-ones of **IAa**, **IAg**, **IAh**, **IBg**, and **IBh**. First, the conformational ensembles of **IAa**, **IAg**, **IAh**, **IBg**, and **IBh** were generated from 2D chemical structures as the initial structures for the density functional theory (DFT) calculations. These conformations were generated and optimized with the RDKit using the universal force field (UFF) and clustered using a tolerance of 0.2 Å root-mean-square deviation. For each conformer, the Hartree–Fock (HF) calculations were carried out to obtain optimized geometries and energies at the RHF/6-31G(d) levels. For each conformer excluding atropisomers, DFT calculations were carried out to obtain optimized geometries and energies at the RB3LYP/6-31G(d) and the RmPW1PW91/6-311+G(d,p) levels. The relative energy differences of the two conformers were estimated on the basis of geometries fully optimized with mPW1PW91/6-311+G(d,p) with energy calculations with the RmPW1PW91/6-311+G(d,p) in the SCRF/IEFPCM model in CHCl_3_.^13^ Zero-point energy (ZPE) correction was made on the basis of the frequency calculation with the RmPW1PW91/6-311+G(d,p). The results are shown in Table 2. As a representative result obtained in those computational studies, the selected conformers of the *E*/*Z*-amide of **IAa** are illustrated in Figure 4. Others are shown in the Supporting Information. It was confirmed that *N*-acyl 5*H*-dibenzo[*b*,*d*]azepin-7(6*H*)-ones exist in the stable relative configuration of a pair of enantiomers [(a^1^*R*, a^2^*R*) and (a^1^*S*, a^2^*S*)] without the presence of diastereomers [(a^1^*R*, a^2^*S*) and (a^1^*S*, a^2^*R*)]. Additionally, in each case, the *E*-amide was preferred, although the energy difference between the *E*-amide and *Z*-amide was less than 10.3 kJ/mol.

Then we investigated the physicochemical properties of (a^1^*R*, a^2^*R*)- and (a^1^*S*, a^2^*S*)-axial isomers. As mentioned above, methylene protons (H-6) in these compounds were observed as diastereotopic, meaning the presence of axial chirality. In **IAg** (R^1^ = H), however, the ring inversion via rotation around the axis was too rapid for separation of the axial isomers at rt, and thus **IAg** was not separated by chiral HPLC. On the other hand, compound **IBg** with a 4-methyl substituent (R^1^ = Me) was conformationally frozen and separable into the stable (a^1^*R*, a^2^*R*)- and (a^1^*S*, a^2^*S*)-isomers, and the separated isomers showed a high energy barrier to rotation (*ΔG*^‡^ = 124.8 kJ/mol).^14^ Next, *N*-methoxycarbonyl 5*H*-dibenzo[*b*,*d*]azepin-7(6*H*)-one (**IAh**) and its 4-methyl derivative (**IBh**) were investigated, and similar results were obtained. While each enantiomer of **IAh** without the 4-methyl substituent (R^1^ = H) was not separable at rt, **IBh** with the 4-methyl substituent (R^1^ = Me) was separable into the stable (a^1^*R*, a^2^*R*)- and (a^1^*S*, a^2^*S*)-axial isomers, and the separated isomers showed a high energy barrier to rotation (*ΔG*^‡^ = 116.0 kJ/mol). The physicochemical properties of **IBg** and **IBh** are shown in Table 3. As expected, the 4-methyl (R^1^ = Me) substituent was helpful to freeze the conformations so that the relatively stable stereoisomers could be separated.

**Table 3:**
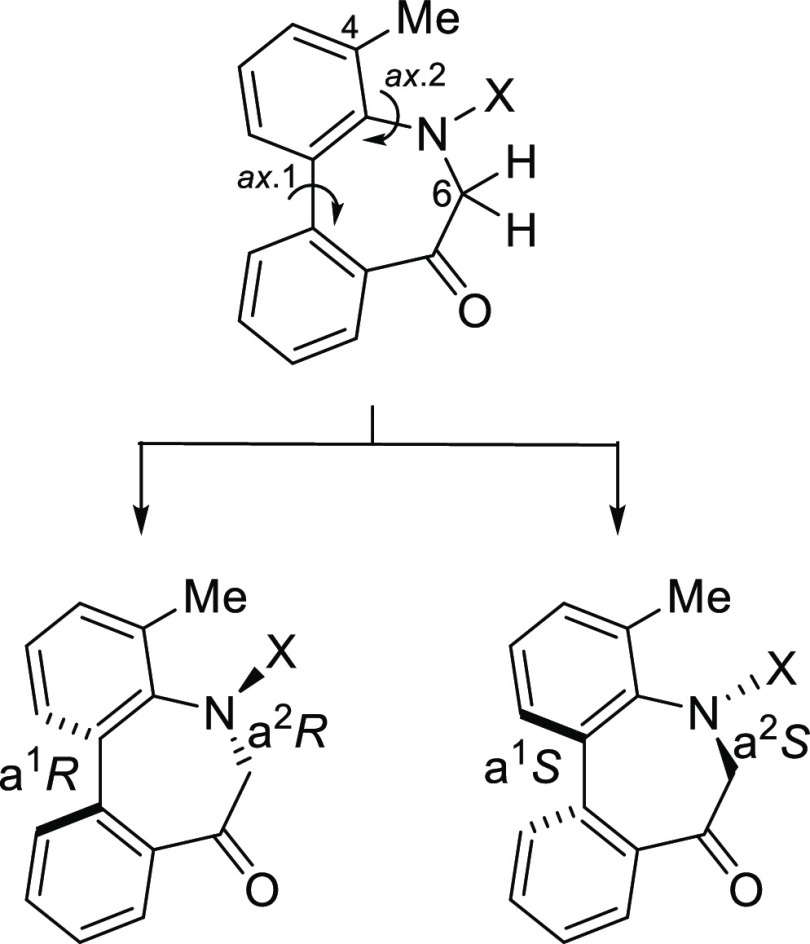
Optical Rotation of the Atropisomers [(+) and (−)] and Their Stereochemical Stability

		[α]_D_^a^		
	X	(+)	(−)	Δ*G*^‡^ (kJ/mol)^b^
**IBg**	COCF_3_	+12.2 as 99.5% ee	–12.1 as 99.9% ee	124.8
**IBh**	COOMe	+145.8 as 94.7% ee	–146.8 as 94.8% ee	116.0
**IIBc**	*p*-Tosyl	+64.9 as 96.1% ee	–65.8 as 96.8% ee	127.5
**IIBd**	Mesyl	+89.8 as 97.0% ee	–90.8 as 98.7% ee	126.3
**IIBe**	*o*-Nosyl	+61.5 as 99.8% ee	–61.1 as 99.8% ee	131.6
**IIBf**	*p*-Nosyl	+27.2 as 98.5% ee	–27.9 as 99.8% ee	131.6

a In CHCl_3_ and for each concentration (*c*), see the Experimental Section.

b Conditions required for determination of Δ*G*^‡^ in toluene at 80 °C.

### Stereochemistry of *N*-Sulfonyl-5*H*-dibenzo[*b*,*d*]azepin-7(6*H*)-ones

**Figure 5:**
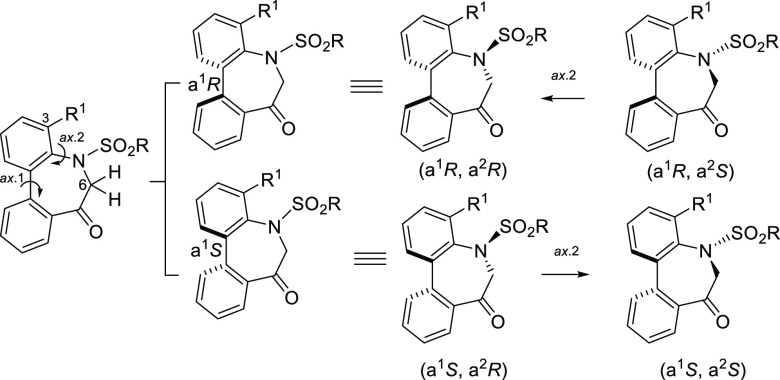
Conformational property of *N*-sulfonyl-5*H*-dibenzo[*b*,*d*]azepin-7(6*H*)-ones.

The stereochemistry of the *N*-sulfonyl derivatives (**IIA**/**B**) was investigated next, and a general picture of the conformational property is shown in Figure 5. Although the sulfonamide group is an important functional moiety observed in various biologically active compounds, its physicochemical properties are not as well understood as those of the amide group. Similar to *N*-acyl derivatives (**IA**/**B**), *N*-sulfonyl derivatives (**IIA**/**B**) should have chirality based on the sp^2^–sp^2^ axis arising from the biphenyl (axis 1). In addition, another axial chirality arising from the benzene–sulfonamide bond should exist. Our studies have recently revealed that the atropisomeric property of the sulfonamide group is caused by the Ar–N(SO_2_) axis (axis 2).^7a,15^ The planarity of the N–SO_2_ arises from both the nitrogen atom possessing an sp^2^-like nature and the double-bond character between the S–N bond. As well as *N*-acyl derivatives (**IA**/**B**), it was anticipated that axes 1 and 2 would move together concertedly to form the stable relative configuration of (a^1^R*, a^2^R*). Thus, the configuration of the enantiomers was presumed to be (a^1^*R*, a^2^*R*) and (a^1^*S*, a^2^*S*), respectively.

In order to elucidate how axes 1 and 2 move together concertedly to form the stable relative configuration, *N*-tosyl 5*H*-dibenzo[*b*,*d*]azepin-7(6*H*)-one (**IIAc**) (R^1^ = H) and its 4-methyl derivative (R^1^ = Me) (**IIBc**) were examined. In the ^1^H NMR (CDCl_3_) spectra of **IIAc** and **IIBc**, they exist as a single compound, and each methylene proton (6-H) was observed as a separated sharp peak, which indicates the presence of chirality. The atropisomers of (**IIAc**) (R^1^ = H) were inseparable by chiral HPLC because the ring inversion via rotation around the axis was too rapid for separation at rt. In contrast, those of **IIBc** (R^1^ = Me) were sufficiently stable to be separated and isolated with chiral HPLC at rt; the separated isomers showed a high energy barrier to rotation (*ΔG*^‡^ = 127.5 kJ/mol). Each isomer has opposite [α]_D_ values: that with shorter retention time in HPLC at 96.8% ee showed [α]_D_ −65.8 (*c* 0.23, CHCl_3_) and that with a longer retention time in HPLC at 96.1% ee showed [α]_D_ +64.9 (*c* 0.21, CHCl_3_), confirming that they are enantiomers. As well as *N*-tosyl 5*H*-dibenzo[*b*,*d*]azepin-7(6*H*)-one (**IIAc**) (R^1^ = H), the atropisomers of other *N*-sulfonyl derivatives (**IIAd**–**f**) (R^1^ = H) were inseparable by chiral HPLC. On the other hand, the presence of the atropisomers of the corresponding 4-methyl derivative (**IIBc**–**f**) (R^1^ = Me) was confirmed by isolating each isomer by chiral HPLC. The separated isomers showed a high energy barrier to rotation (**IIBd**: *ΔG*^‡^ = 126.3 kJ/mol, **IIBe**: *ΔG*^‡^ = 131.6 kJ/mol, **IIBf**: *ΔG*^‡^ = 131.6 kJ/mol). The physicochemical properties of **IIBc**–**f** are shown in Table 3. It is noteworthy that compounds **IIBe** and **IIBf** with the most electron-withdrawing nosyl group showed the highest energy barrier to rotation.

**Figure 6:**
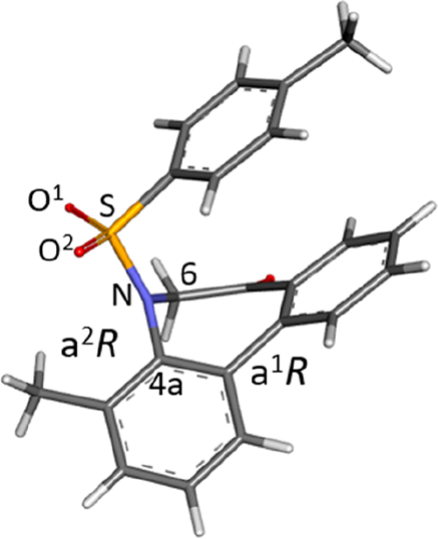
X-ray crystal structure of **IIBc**. The structure with the (a^1^*R*, a^2^*R*) stereochemistry was extracted from the CIF data of the racemates.

Fortunately, a single crystal for the X-ray crystal structure analysis of **IIBc** (racemate) was obtained, in which **IIBc** possessed the stable relative configuration of (a*R*, a*R*) and (a*S*, a*S*) as expected in a unit cell (Figure 6). It was revealed that axial chirality caused by the Ar–N(SO_2_) axis (axis 2), which showed a rather high energy barrier (*ΔG*^‡^ = 126.3–131.6 kJ/mol), moves concertedly with the axis at the biphenyl (axis 1) to form the stable relative configuration of (aR*, aR*) without the presence of diastereomers (aR*, aS*). Such a high energy barrier might be due to the planarity of the nitrogen atom.^16^ The sum of angles around the nitrogen atom in the >N–SO_2_ moiety is 359.2°, indicating the sp^2^-like nature of the nitrogen atom. In addition, the bond length between N–S (0.16 nm) suggests the double-bond character of the N–S bond. While these data imply that the > N–S moiety forms a plane, it was found that the SO_2_ moiety locates so as to interweave with the N–SO_2_ axis: dihedral angles ∠O^1^–S–N–C6 and ∠O^2^–S–N–C4a were −44.90° and +12.64°, respectively. The important point to note is that the sulfonyl (S=O) bond is not on the >N–S plane. Considering that the carbonyl (C=O) is on the amide (>N–C=O) plane and the N–C bond has a double-bond character due to the resonance, the double-bond character of the N–S bond without the planarity of sulfonamide (>N–S=O) is interesting. It was also found that the seven-membered ring exists in a boat-like form, the benzene ring of the tosyl moiety locates over the benzene ring of biphenyl (folded form), and they are nearly parallel to each other.

### Blockade of Potassium Channel Kv1.3

We next conducted a preliminary investigation of the blockade of the potassium channel. Considering the level of activity on Kv1.3 of **IIAc** (IC_50_ 5.8 μM),^4^ blocking activity on the voltage-gated potassium channel Kv1.3 with 4-aminopyridine as a positive control was tested for **IIBc** using patch-clamp technology (Table 4). **IIBc** in racemic form showed a moderate level of inhibitory activity of 63% at 10 μM when the channel was in the closed state. Hence, the separated enantiomers of (+)-**IIBc** and (−)-**IIBc** were subjected to the binding assay to examine the difference in potency between the enantiomers. Although the enantiomers and the racemate exhibited similar levels of affinity (within a 4.5-fold difference), (−)-**IIBc** showed more potent affinity than (+)-**IIBc**.

**Table 4:** Blocking Activity of **IIBc** at 10 μM (Racemate and Atropisomers)

	% Inhibition^a^
**IIBc**	63
(+)-**IIBc**	14
(−)-**IIBc**	43
4-Aminopyridine^b^	19

a % inhibition values shown are the means of duplicate measurements.

b At 100 μM.

## Conclusion

The efficient synthesis of *N*-acyl/*N*-sulfonyl 5*H*-dibenzo[*b*,*d*]azepin-7(6*H*)-ones and the physicochemical properties of *N*-acyl/*N*-sulfonyl 5*H*-dibenzo[*b*,*d*]azepin-7(6*H*)-ones were elucidated. Improvement of the Friedel–Crafts acylation of *N*-(1,1′)-biphenyl-2-yl-glycine derivatives^17^ was achieved by the introduction of the amino-protective groups with electron-withdrawing properties. ^1^H NMR revealed that *N*-acyl 5*H*-dibenzo[*b*,*d*]azepin-7(6*H*)-ones exist in *E*-amide in preference to *Z*-amide in solution, which was also supported by DFT calculations. The equilibration of the amide diastereomer means that the exocyclic bond (axis 3) is not in concert with the endocyclic axes (axis 1, axis 2). Additionally, stable atropisomers [(a^1^*R*, a^2^*R*) and (a^1^*S*, a^2^*S*)] of 4-methyl derivatives of *N*-acyl-5*H*-dibenzo[*b*,*d*]azepin-7(6*H*)-ones were isolated. Similarly, the atropisomers of 4-methyl derivatives of *N*-sulfonyl 5*H*-dibenzo[*b*,*d*]azepin-7(6*H*)-ones were isolated. The separated isomers showed a high energy barrier to rotation (*ΔG*^‡^ = 116.0–131.6 kJ/mol), and X-ray crystal structure analysis of the racemate of 4-methyl-5-tosyl-5,6-dihydro-7*H*-dibenzo[*b*,*d*]azepin-7-one showed that it possessed the stable relative configuration of (a^1^*R*, a^2^*R*) and (a^1^*S*, a^2^*S*) in a unit cell. It was revealed that axial chirality caused by the Ar–N(SO_2_) axis moves together concertedly with the axis at the biphenyl to form the stable relative configuration of (a^1^R*, a^2^R*). The preliminary results on the difference between the atropisomers of *N*-sulfonyl 5*H*-dibenzo[*b*,*d*]azepin-7(6*H*)-ones in the potency of potassium channel Kv1.3 blockade might be a clue for the design of potassium channel inhibitors. More detailed investigation through the structure–activity relationship (SAR) study of *N*-acyl/*N*-sulfonyl 5*H*-dibenzo[*b*,*d*]azepin-7(6*H*)-ones is under consideration.

## Experimental Section

### General Information

All reagents were purchased from commercial suppliers and used as received. Reaction mixtures were stirred magnetically, and the reactions were monitored by thin-layer chromatography (TLC) on precoated silica gel plates. For the reactions that require heating, an oil bath was used. Column chromatography was performed using silica gel (45–60 μm). For recrystallizaton, crude products were dissolved in AcOEt/diisopropyl ether/hexane, and the precipitated crystals were collected. Extracted solutions were dried over anhydrous Na_2_SO_4_. Solvents were evaporated under reduced pressure. NMR spectra were recorded on a spectrometer at 600 MHz for ^1^H NMR and at 150 MHz for ^13^C NMR at 296 K unless otherwise stated. Tetramethylsilane (TMS) (δ 0.00) or residual internal CHCl_3_ (δ 7.26) was used as an internal reference for the ^1^H spectroscopy measurements of samples in CDCl_3_. TMS (δ 0.00) or residual internal CHCl_3_ (δ 77.16) was used as an internal reference for the ^13^C spectroscopy measurements of samples in CDCl_3_. Coupling constants (*J*) are reported in hertz (Hz). Splitting patterns are abbreviated as follows: singlet (s); doublet (d); triplet (t); quartet (q); multiplet (m); and broad (br). The high-resolution mass spectra (HRMS) were recorded using an ESI/TOF, APCI/TOF, or EI-MS mass spectrometer. IR spectra were recorded on an FT-IR spectrometer equipped with ATR (Diamond). Melting points were recorded on a melting point apparatus and are uncorrected. The chemical structures of **S1**–**S10** were shown in the Supporting Information.

#### *N*-([1,1′-Biphenyl]-2-yl)-*N*-acetylglycine (**1Aa**)


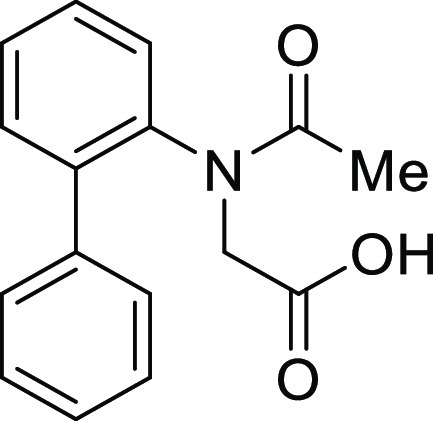
NaOH aq (930 μL, 9.30 mmol) was added to a stirred solution of methyl ester **S2a** (1.05 g, 3.70 mmol) in MeOH (7.4 mL) at rt under an argon atmosphere. After stirring for 2 h, the mixture was treated with HCl and extracted with ethyl acetate. The extract was washed with 1 M HCl aq. and brine, dried, and concentrated. The concentrate was purified by recrystallization. Colorless crystal (800.0 mg, 80%), mp 173–174 °C: ^1^H NMR (600 MHz, CDCl_3_) δ 7.55 (d, 1H, *J* = 8.4 Hz), 7.44–7.36 (m, 6H), 7.25–7.24 (m, 2H), 4.52 (d, 1H, *J* = 17.4 Hz), 3.38 (d, 1H, *J* = 17.4 Hz), 1.95 (s, 3H); ^13^C{1H} NMR (150 MHz, CDCl_3_) δ 172.8, 172.2, 140.3, 139.4, 138.4, 131.5, 129.8, 129.2, 129.0, 128.5, 128.1, 51.2, 22.2; IR (ATR) 2873, 1716, 1608 cm^–1^; HRMS (ESI-TOF) *m*/*z* calcd for C_16_H_15_NO_3_Na 292.0944 (M+Na)^+^, found 292.0951.

#### *N*-([1,1′-Biphenyl]-2-yl)-*N*-(4-methylbenzoyl)glycine (**1Ab**)


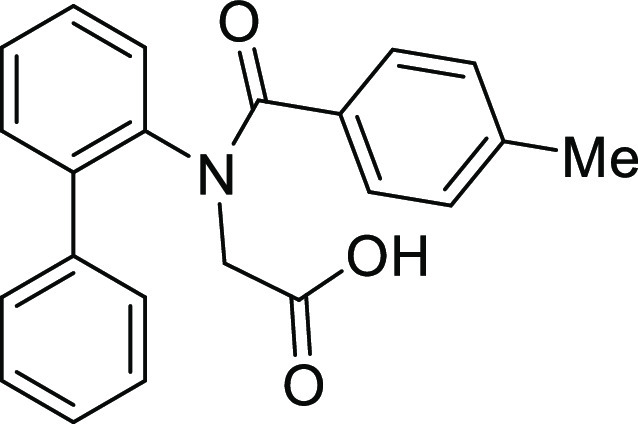
Compound **1Ab** was prepared according to a similar procedure as described for the preparation of **1Aa** from **S2a**. Colorless crystal (900.0 mg, 62%), mp 147–149 °C: ^1^H NMR (600 MHz, CDCl_3_) δ 7.37–7.34 (m, 4H), 7.31 (ddd, 2H, *J* = 7.8, 7.8, 3.0 Hz), 7.26 (ddd, 1H, *J* = 7.2, 7.2, 1.8 Hz), 7.15 (dd, 2H, *J* = 7.2, 1.2 Hz), 7.05 (d, 2H, *J* = 8.4 Hz), 6.90 (d, 2H, *J* = 8.4 Hz), 4.78 (d, 1H, *J* = 17.4 Hz), 3.76 (d, 1H, *J* = 17.4 Hz), 2.27 (s, 3H); ^13^C{1H} NMR (150 MHz, CDCl_3_) δ 173.6, 170.7, 141.2, 140.8, 138.4, 138.3, 131.4, 131.2, 129.6, 129.3, 128.9, 128.7, 128.4, 128.3, 127.9, 53.0, 21.5; IR (ATR) 2827, 1716, 1607 cm^–1^; HRMS (ESI-TOF) *m*/*z* calcd for C_22_H_20_NO_3_ 346.1438 (M+H)^+^, found 346.1439.

#### *N*-([1,1′-Biphenyl]-2-yl)-*N*-(2,2,2-trifluoroacetyl)glycine (**1Ag**)


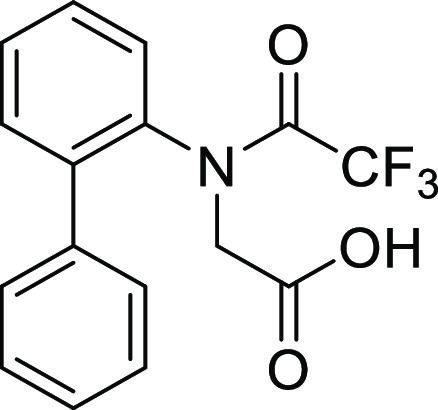
The benzyl ester of **S2g** (141.0 mg, 0.34 mmol) was dissolved in THF/MeOH (5.0 mL), and 10% palladium on activated carbon (14.1 mg, 10% w/w) was added at rt for 18 h under a hydrogen atmosphere. The mixture was filtered and washed with 1 M HCl aq. and brine. The filtrate was dried and concentrated under reduced pressure. The concentrate was purified by recrystallization to afford **1Ag** as colorless crystals (116.0 mg, 99%), mp 190–191 °C: ^1^H NMR (600 MHz, CDCl_3_) δ 7.56 (d, 1H, *J* = 7.2 Hz), 7.49 (ddd, 1H, *J* = 7.2, 7.2, 1.2 Hz), 7.45–7.38 (m, 5H), 7.27 (dd, 2H, *J* = 6.6, 1.8 Hz), 4.37 (d, 1H, *J* = 17.4 Hz), 3.42 (d, 1H, *J* = 17.4 Hz); ^13^C{1H} NMR (150 MHz, CDCl_3_) δ 171.8, 158.2 (C–F, ^2^*J*_C–F_ = 37.6 Hz), 139.3, 137.9, 136.9, 131.6, 130.0, 129.7, 129.1, 128.7, 128.6, 128,4, 116.3 (C–F, ^1^*J*_C–F_ = 289.0 Hz), 52.1; IR (ATR) 2929, 1732, 1693 cm^–1^; HRMS (ESI-TOF) *m*/*z* calcd for C_16_H_12_F_3_NO_3_Na 322.0697 (M+Na)^+^, found 322.0702.

#### *N*-([1,1′-Biphenyl]-2-yl)-*N*-(methoxycarbonyl)glycine (**1Ah**)


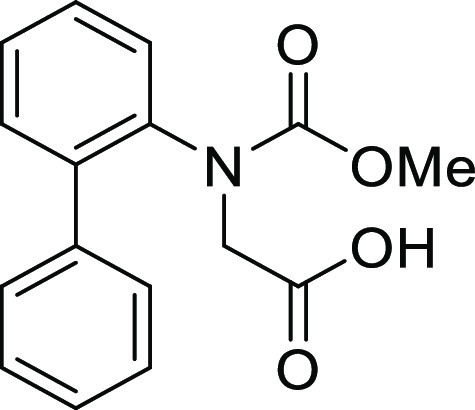
Compound **1Ah** was prepared according to a similar procedure as described for the preparation of **1Aa** from **S2a**, purified by recrystallization. Colorless crystal (331.0 mg, 70%), mp 138–140 °C: ^1^H NMR (600 MHz, CDCl_3_) δ 7.50 (dt, 1H, *J* = 6.6, 1.8 Hz), 7.42–7.33 (m, 7H), 7.28–7.26 (m, 1H), 4.34 (d, 1H, *J* = 18.0 Hz), 3.64 (s, 3H), 3.40 (d, 1H, *J* = 18.0 Hz); ^13^C{1H} NMR (150 MHz, CDCl_3_) δ 174.6, 156.8, 139.4, 139.0, 138.9, 130.9, 129.7, 128.8, 128.6, 128.5, 128.4, 127.8, 53.5, 51.6; IR (ATR) 3070, 1770, 1664 cm^–1^; HRMS (ESI-TOF) *m*/*z* calcd for C_16_H_15_NO_4_Na 308.0893 (M+Na)^+^, found 308.0908.

### General Procedure of Intramolecular Friedel–Crafts Acylation

#### 5-(2,2,2-Trifluoroacetyl)-5,6-dihydro-7*H*-dibenzo[*b*,*d*]azepin-7-one (**IAg**)


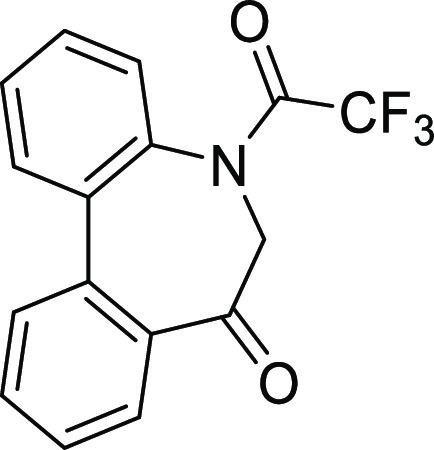
**1Ag** (1.00 g, 3.09 mmol) was dissolved in SOCl_2_ (7.00 mL, 0.5 M) at reflux for 1 h under an argon atmosphere. The mixture was concentrated under reduced pressure. The concentrate was dissolved in CH_2_Cl_2_ (20.0 mL) at −78 °C under an argon atmosphere, and aluminum chloride (1.98 g, 14.8 mmol) was added. After being stirred at rt for 1 h, the mixture was treated with 1 M HCl aq. and extracted with ethyl acetate. The extract was washed with 1 M HCl aq., 1 M NaHCO_3_ aq., and brine, dried, and concentrated. The concentrate was purified by column chromatography (silica gel, hexane/EtOAc = 4:1) to afford **IAg** as colorless crystals (914.0 mg, 97%), mp 95–96 °C: ^1^H NMR (600 MHz, CDCl_3_) *E*-isomer: δ 7.79 (dd, 1H, *J* = 8.4, 1.8 Hz), 7.68 (ddd, 1H, *J* = 7.5, 7.5, 1.2 Hz), 7.60–7.58 (m, 2H), 7.53–7.49 (m, 3H), 7.43 (d, 1H, *J* = 7.8 Hz), 5.56 (d, 1H, *J* = 18.6 Hz), 4.11 (d, 1H, *J* = 18.6 Hz); *Z*-isomer: δ 7.79 (dd, 1H, *J* = 8.4, 1.8 Hz), 7.68 (ddd, 1H, *J* = 7.5, 7.5, 1.2 Hz), 7.60–7.58 (m, 2H), 7.53–7.49 (m, 3H), 7.43 (d, 1H, *J* = 7.8 Hz), 5.07 (d, 1H, *J* = 21.0, 1.8 Hz), 4.37 (d, 1H, *J* = 21.0, 1.8 Hz); ^13^C{1H} NMR (150 MHz, CDCl_3_) *E*-isomer: δ 200.9, 156.8 (C–F, ^2^*J*_C–F_ = 37.6 Hz), 138.5, 136.7, 136.5, 135.4, 133.9, 130.9, 130,9 129.9, 129.8, 129.7, 129.3, 127.6, 116.1 (C–F, ^1^*J*_C–F_ = 289.0 Hz), 62.3; IR (ATR) 1695, 1678 cm^–1^; HRMS (ESI-TOF) *m*/*z* calcd for C_15_H_9_F_3_N 350.0646 (M+HCOO)^−^, found 350.0652.

#### Methyl 7-Oxo-6,7-dihydro-5*H*-dibenzo[*b,d*]azepine-5-carboxylate (**IAh**)


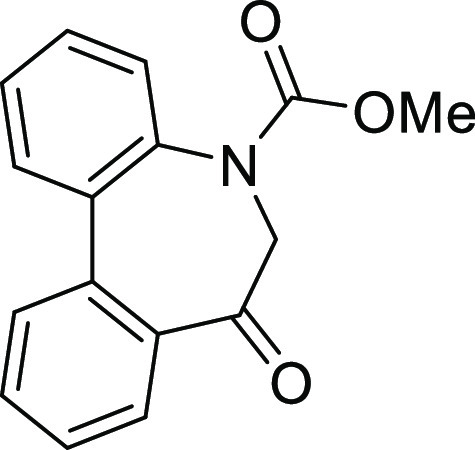
Compound **IAh** was prepared according to a similar procedure as described for the preparation of **IAg** from **1Ag**. Colorless crystal (31.5 mg, 84%), mp 130–131 °C: ^1^H NMR (600 MHz, CDCl_3_) *E*-isomer: δ 7.81 (d, 1H, *J* = 7.8 Hz), 7.65 (dd, 1H, *J* = 7.2, 7.2 Hz), 7.59–7.52 (m, 2H), 7.50–7.43 (m, 3H), 7.34 (d, 1H, *J* = 6.6 Hz), 5.36 (d, 1H, *J* = 18.6 Hz), 4.09 (d, 1H, *J* = 18.6 Hz), 3.55 (s, 3H); *Z*-isomer: δ 7.81 (d, 1H, *J* = 7.8 Hz), 7.65 (dd, 1H, *J* = 7.2, 7.2 Hz), 7.59–7.52 (m, 2H), 7.50–7.43 (m, 3H), 7.34 (d, 1H, *J* = 6.6 Hz), 5.14 (d, 1H, *J* = 19.2 Hz), 4.10 (d, 1H, *J* = 19.2 Hz), 3.67 (s, 3H); ^13^C{1H} NMR (150 MHz, CDCl_3_) *E*-isomer: δ 203.8, 155.9, 139.0, 137.7, 137.3, 136.4, 133.4, 130.7, 129.9, 129.8, 129.6, 129.2, 128.6, 128.0, 62.6, 53.5; IR (ATR) 1699, 1686 cm^–1^; HRMS (ESI-TOF) *m*/*z* calcd for C_16_H_13_NO_3_Na 297.0788 (M+Na)^+^, found 290.0792.

#### *N*-(3-Methyl-[1,1′-biphenyl]-2-yl)-*N*-(2,2,2-trifluoroacetyl)glycine (**1Bg**)


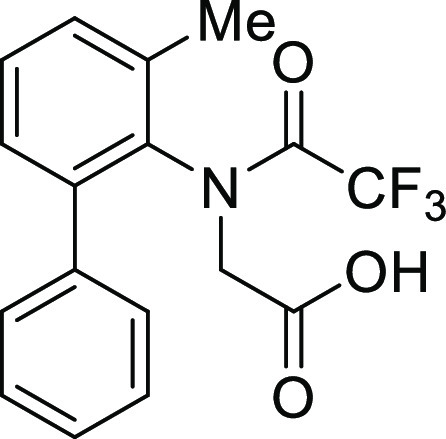
K_2_CO_3_ (265.9 mg, 1.92 mmol) was added to a stirred solution of amide **S5g** (358.0 mg, 1.28 mmol) in DMF (2.6 mL) at rt under an argon atmosphere and treated with benzyl bromoacetate (181.0 μL, 1.15 mmol) for 2 days. The reaction mixture was poured into water and extracted with ethyl acetate. The extract was washed with brine, dried, and concentrated. The concentrate was dissolved in CH_2_Cl_2_, and TFAA (537.6 μL, 3.84 mmol) was added at 0 °C under an argon atmosphere for 1 h. The mixture was treated with 1 M HCl aq. and extracted with ethyl acetate. The extract was washed with 1 M HCl aq., 1 M NaHCO_3_ aq., and brine, dried, and concentrated. The concentrate was purified by column chromatography (silica gel, hexane/ethyl acetate = 4:1) to afford benzyl ester as a crude crystal (506.0 mg, 1.50 mmol). Benzyl ester was dissolved in THF/MeOH (7.5 mL), and 10% palladium on activated carbon (50.6 mg, 10% w/w) was added at rt for 18 h under a hydrogen atmosphere. The mixture was filtered and washed with 1 M HCl aq. and brine. The filtrate was dried and concentrated under reduced pressure. The concentrate was purified by recrystallization to afford **1Bg** as colorless crystals (334.2 mg, 52%), mp 168–169 °C: ^1^H NMR (600 MHz, CDCl_3_) δ 7.42–7.38 (m, 2H), 7.38–7.34 (m, 2H), 7.30 (d, 1H, *J* = 6.0 Hz), 7.19–7.18 (m, 3H), 4.06 (d, 1H, *J* = 17.2 Hz), 3.62 (d, 1H, *J* = 17.2 Hz), 2.46 (s, 3H); ^13^C{1H} NMR (150 MHz, CDCl_3_) δ171.6, 158.5 (C–F, ^2^*J*_C–F_ = 37.6 Hz), 141.0, 138.6, 137.2, 137.0, 130.1, 129.4, 129.3, 129.2, 128.9, 128.8, 128.4, 128.2, 116.1 (C–F, ^1^*J*_C–F_ = 287.6 Hz), 53.8, 18.3; IR (ATR) 2948, 1740, 1692 cm^–1^; HRMS (ESI-TOF) *m*/*z* calcd for C_17_H_14_F_3_NO_3_Na 360.0818 (M+Na)^+^, found 360.0826.

#### *N*-(Methoxycarbonyl)-*N*-(3-methyl-[1,1′-biphenyl]-2-yl)glycine (**1Bh**)


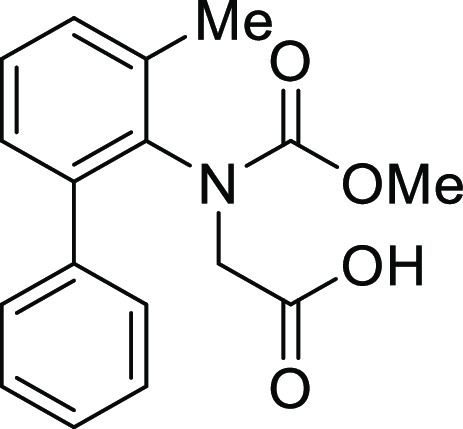
Compound **1Bh** was prepared according to a similar procedure as described for the preparation of **1Aa** from **S2a**. Colorless crystal (232.5 mg, 99%), mp 179–180 °C: ^1^H NMR (600 MHz, CDCl_3_) δ 7.42–7.39 (m, 2H), 7.35 (ddd, 1H, *J* = 7.2, 7.2, 1.2 Hz), 7.28 (dd, 1H, *J* = 7.8, 7.8 Hz), 7.26–7.23 (m, 1H), 7.22 (dd, 2H, *J* = 9.0, 1.8 Hz), 7.18 (dd, 1H, *J* = 6.6, 1.8 Hz), 3.88 (d, 1H, *J* = 17.7 Hz), 3.78 (s, 3H), 3.33 (d, 1H, *J* = 17.7 Hz), 2.40 (s, 3H); ^13^C{1H} NMR (150 MHz, CDCl_3_) δ 172.9, 157.1, 139.6, 139.3, 138.0, 137.8, 130.4, 128.8, 128.7, 128.6, 128.5, 128.3, 128.0, 127.7, 53.6, 52.1, 18.2; IR (ATR) 3061, 1755, 1670 cm^–1^; HRMS (ESI-TOF) *m*/*z* calcd for C_17_H_17_NO_4_Na 322.1050 (M+Na)^+^, found 332.1051.

#### 4-Methyl-5-(2,2,2-trifluoroacetyl)-5,6-dihydro-7*H*-dibenzo[*b,d*]azepin-7-one (**IBg**)


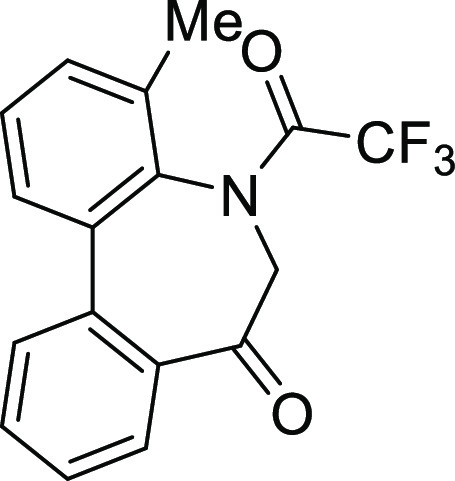
Compound **IBg** was prepared according to a similar procedure as described for the preparation of **IAg** from **1Ag**. Colorless crystal (37.4 mg, 99%), mp 84–85 °C: ^1^H NMR (600 MHz, CDCl_3_) *E*-isomer: δ 7.73 (dd, 1H, *J* = 7.8, 1.8 Hz), 7.66 (ddd, 1H, *J* = 7.6, 7.6, 1.6 Hz), 7.52–7.45 (m, 3H), 7.38 (dd, 2H, *J* = 6.4, 6.4 Hz), 5.50 (d, 1H, *J* = 18.0 Hz), 3.99 (d, 1H, *J* = 18.0 Hz), 2.37 (s, 3H); *Z*-isomer: δ 7.73 (dd, 1H, *J* = 7.8, 1.8 Hz), 7.66 (ddd, 1H, *J* = 7.6, 7.6, 1.6 Hz), 7.52–7.45 (m, 3H), 7.38 (dd, 2H, *J* = 6.4, 6.4 Hz), 5.07 (d, 1H, *J* = 19.6, 1.8 Hz), 4.26 (d, 1H, *J* = 19.6, 1.8 Hz), 2.30 (s, 3H); ^13^C{1H} NMR (150 MHz, CDCl_3_) *E*-isomer: δ 201.3, 157.4 (C–F, ^2^*J*_C–F_ = 111.2 Hz), 139.3, 136.9, 136.1, 135.4, 135.2, 133.7, 131.5, 130.6, 129.7, 129.5, 129.2, 128.5, 115.8 (C–F, ^1^*J*_C–F_ = 865.5 Hz), 61.6, 17.6; *Z*-isomer: δ 200.5, 155.6 (C–F, ^2^*J*_C–F_ = 37.6 Hz), 137.1, 137.0, 136.8, 135.1, 134.8, 134.1, 131.7, 130.1, 129.7, 129.2, 129.0, 128.9, 116.2 (C–F, ^1^*J*_C–F_ = 289.0 Hz), 61.8, 17.4; IR (ATR) 1700, 1686 cm^–1^; HRMS (ESI-TOF) *m*/*z* calcd for C_17_H_11_F_3_NO_2_ 318.0747 (M–H)^−^, found 318.0743. Separation of atropisomers. CHIRALPAK IA (1.0 cmϕ × 25 cm): eluent, 30% 2-propanol in hexane; flow rate, 0.5 mL/min; temperature, 25 °C; detection, 254 nm; former peak, retention time = 9.4 min; [α]_D_^20^ +12.2 as 99.5% ee (*c* 0.15, CHCl_3_); latter peak, retention time = 13.8 min; [α]_D_^20^ −12.1 as 99.9% ee (*c* 0.35, CHCl_3_).

#### Methyl 4-Methyl-7-oxo-6,7-dihydro-5*H*-dibenzo[*b,d*]azepine-5-carboxylate (**IBh**)


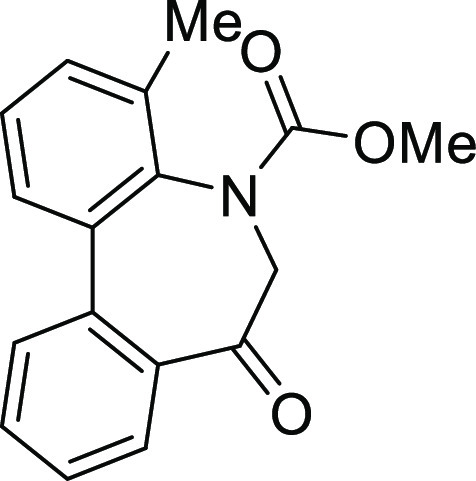
Compound **IBh** was prepared according to a similar procedure as described for the preparation of **IAg** from **1Ag**. Colorless crystal (32.3 mg, 96%), mp 116–118 °C: ^1^H NMR (600 MHz, CDCl_3_) *E*-isomer: δ 7.77 (dd, 1H, *J* = 8.4, 1.2 Hz), 7.63 (ddd, 1H, *J* = 7.8, 7.8, 1.2 Hz), 7.50–7.45 (m, 2H), 7.40–7.35 (m, 2H), 7.34–7.31 (m, 1H), 5.31 (d, 1H, *J* = 18.6 Hz), 3.98 (d, 1H, *J* = 18.6 Hz), 3.52 (s, 3H), 2.30 (s, 3H); *Z*-isomer: δ 7.77 (dd, 1H, *J* = 8.4, 1.2 Hz), 7.63 (ddd, 1H, *J* = 7.8, 7.8, 1.2 Hz), 7.50–7.45 (m, 2H), 7.39–7.35 (m, 2H), 7.34–7.30 (m, 1H), 5.08 (d, 1H, *J* = 18.6 Hz), 4.00 (d, 1H, *J* = 18.6 Hz), 3.63 (s, 3H), 2.34 (s, 3H); ^13^C{1H} NMR (150 MHz, CDCl_3_) *E*-isomer: δ 204.2, 155.8, 138.4, 137.7, 136.4, 136.0, 134.7, 133.2, 131.1, 129.7, 129.6, 129.1, 128.6, 128.5, 61.7, 53.5, 17.7; IR (ATR) 1705, 1678 cm^–1^; HRMS (ESI-TOF) *m*/*z* calcd for C_17_H_15_NO_3_SK 320.0684 (M+K)^+^, found 320.0685. Separation of atropisomers. CHIRALPAK IA (1.0 cmϕ × 25 cm): eluent, 30% 2-propanol in hexane; flow rate, 0.5 mL/min; temperature, 25 °C; detection, 254 nm; former peak, retention time = 10.3 min; [α]_D_^20^ +145.8 as 94.7% ee (*c* 0.07, CHCl_3_); latter peak, retention time = 15.0 min; [α]_D_^20^ −146.8 as 94.8% ee (*c* 0.06, CHCl_3_).

#### *N*-([1,1′-Biphenyl]-2-yl)-*N*-tosylglycine (**1Ac**)


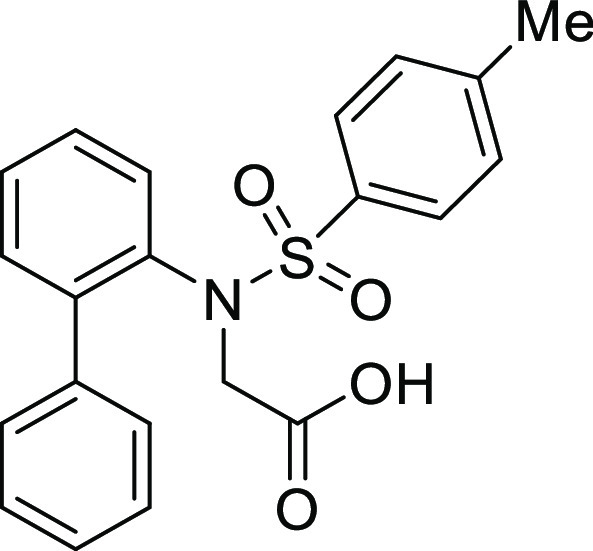
NaOH aq. (200.0 μL, 0.50 mmol) was added to a stirred solution of methyl ester **S10c** (116.0 mg, 0.29 mmol) in MeOH (4.0 mL) at rt under an argon atmosphere. After stirring for 2 h, the mixture was treated with HCl and extracted with ethyl acetate. The extract was washed with 1 M HCl aq. and brine, dried, and concentrated. The concentrate was purified by recrystallization to afford **1Ac** as colorless crystals (159.7 mg, 84%), mp 193–194 °C: ^1^H NMR (600 MHz, CDCl_3_) δ 7.62 (d, 2H, *J* = 8.4 Hz), 7.38–7.35 (m, 5H), 7.32–7.26 (m, 6H), 3.99 (br, 2H), 2.44 (s, 3H); ^13^C{1H} NMR (150 MHz, CDCl_3_) δ 173.2, 144.0, 141.4, 138.7, 137.4, 137.1, 131.8, 130.7, 129.6, 129.1, 128.9, 128.5, 128.2, 127.9, 52.0, 21.7; IR (ATR) 3200, 1743, 1327, 1140 cm^–1^; HRMS (ESI-TOF) *m*/*z* calcd for C_21_H_19_NO_4_SNa 404.0927 (M+Na)^+^, found 404.0936.

#### *N*-([1,1′-Biphenyl]-2-yl)-*N*-(methylsulfonyl)glycine (**1Ad**)


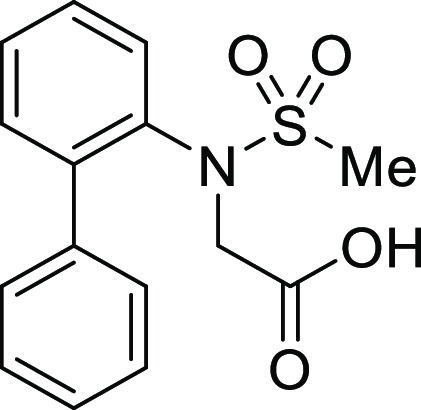
Compound **1Ad** was prepared according to a similar procedure as described for the preparation of **1Ac** from **S10c**. Colorless crystal (186.0 mg, 70%), mp 100–101 °C: ^1^H NMR (600 MHz, CDCl_3_) δ 7.63–7.62 (m, 1H), 7.51 (dd, 2H, *J* = 7.2, 1.8 Hz), 7.46–7.39 (m, 6H), 4.01 (br, 2H), 3.15 (s, 3H); ^13^C{1H} NMR (150 MHz, CDCl_3_) δ 174.1, 141.6, 138.6, 137.4, 132.1, 129.9, 129.3, 129.2, 128.7, 128.1, 52.1, 42.6; IR (ATR) 3365, 1706, 1323, 1142 cm^–1^; HRMS (ESI-TOF) *m*/*z* calcd for C_15_H_15_NO_4_SNa 328.0614 (M+Na)^+^, found 328.0622.

#### *N*-([1,1′-Biphenyl]-2-yl)-*N*-(2-nitrobenzenesulfonyl)glycine (**1Ae**)


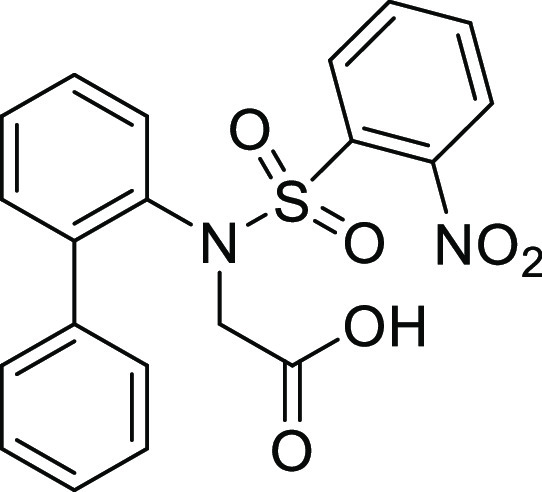
Compound **1Ae** was prepared according to a similar procedure as described for the preparation of **1Ac** from **S10c**. Colorless crystal (163.0 mg, 66%), mp 120–121 °C: ^1^H NMR (600 MHz, CDCl_3_) δ 8.27 (dd, 1H, *J* = 8.4, 8.4 Hz), 7.87 (d, 1H, *J* = 7.8 Hz), 7.76–7.73 (m, 1H), 7.69 (ddd, 1H, *J* = 7.2, 7.2, 1.8 Hz), 7.64 (dd, 1H, *J* = 7.2, 7.2 Hz), 7.56 (d, 1H, *J* = 7.8 Hz), 7.43 (dd, 1H, *J* = 8.4, 8.4 Hz), 7.39 (ddd, 1H, *J* = 8.4, 8.4, 1.8 Hz), 7.31–7.27 (m, 3H), 7.14–7.15 (m, 2H), 3.60 (br, 2H); ^13^C{1H} NMR (150 MHz, CDCl_3_) δ 172.6, 148.0, 141.9, 138.3, 136.8, 135.2, 134.1, 133.8, 131.9, 131.8, 131.7, 129.5, 128.8, 128.6, 128.4, 127.8, 126.1, 125.4, 124.6, 53.0; IR (ATR) 3276, 1758, 1539, 1349, 1334, 1121 cm^–1^; HRMS (ESI-TOF) *m*/*z* calcd for C_20_H_16_N_2_O_6_SNa 435.0621 (M+Na)^+^, found 435.0625.

#### *N*-([1,1′-Biphenyl]-2-yl)-*N*-(4-nitrobenzenesulfonyl)glycine (**1Af**)


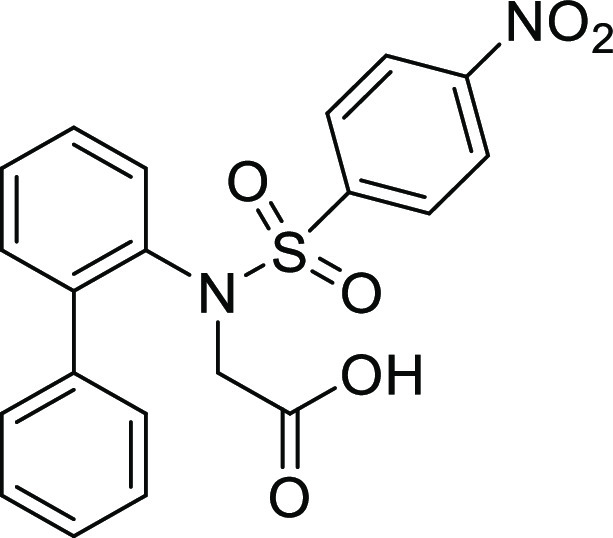
Compound **1Af** was prepared according to a similar procedure as described for the preparation of **1Ac** from **S10c**. Colorless crystal (249.6 mg, 85%), mp 179–181 °C: ^1^H NMR (600 MHz, CDCl_3_) δ 8.25 (ddd, 2H, *J* = 8.4, 2.4, 2.4 Hz), 7.87 (dd, 2H, *J* = 8.4, 2.4 Hz), 7.44 (ddd, 1H, *J* = 6.6, 6.6, 2.4 Hz), 7.40–7.32 (m, 6H), 7.31–7.29 (m, 2H), 4.42 (br, 2H); ^13^C{1H} NMR (150 MHz, CDCl_3_) δ 173.0, 150.2, 146.1, 141.7, 138.3, 136.4, 132.2, 130.6, 129.7, 129.4, 128.9, 128.7, 128.6, 128.1, 124.1, 52.7; IR (ATR) 3318, 1774, 1528, 1338, 1307, 1138 cm^–1^; HRMS (ESI-TOF) *m*/*z* calcd for C_20_H_16_N_2_O_6_SNa 435.0621 (M+Na)^+^, found 435.0626.

#### 5-Tosyl-5,6-dihydro-7*H*-dibenzo[*b*,*d*]azepin-7-one (**IIAc**)


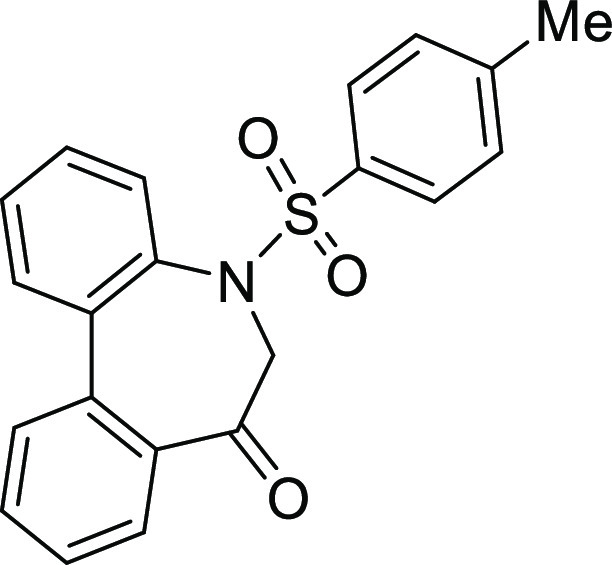
Compound **IIAc** was prepared according to a similar procedure as described for the preparation of **IAg** from **1Ag**. Colorless crystal (33.0 mg, 91%), mp 124–125 °C: ^1^H NMR (600 MHz, CDCl_3_) δ 7.64–7.61 (m, 1H), 7.52 (dd, 1H, *J* = 8.4, 1.6 Hz), 7.49–7.45 (m, 2H), 7.42–7.39 (m, 2H), 7.27 (ddd, 1H, *J* = 7.8, 7.8, 1.2 Hz), 7.17 (d, 2H, *J* = 7.8 Hz), 7.04 (d, 1H, *J* = 6.6 Hz), 6.84 (d, 2H, *J* = 7.8 Hz), 5.27 (d, 1H, *J* = 19.2 Hz), 4.38 (d, 1H, *J* = 19.2 Hz), 2.26 (s, 3H); ^13^C{1H} NMR (150 MHz, CDCl_3_) δ 203.0, 143.1, 139.2, 137.5, 137.1, 137.0, 136.2, 133.0, 130.9, 130.8, 130.2, 130.0, 129.9, 129.5, 129.4, 127.9, 126.8, 63.7, 21.5; IR (ATR) 1675, 1335, 1155 cm^–1^; HRMS (ESI-TOF) *m*/*z* calcd for C_21_H_17_NO_3_SNa 386.0821 (M+Na)^+^, found 386.0822.

#### 5-(Methylsulfonyl)-5,6-dihydro-7*H*-dibenzo[*b,d*]azepin-7-one (**IIAd**)


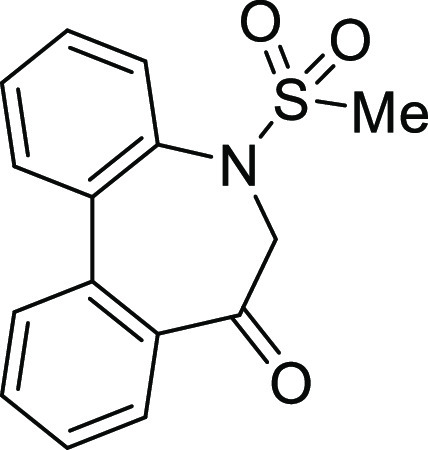
Compound **IIAd** was prepared according to a similar procedure as described for the preparation of **IAg** from **1Ag**. Colorless crystal (32.0 mg, 86%), mp 170–171 °C: ^1^H NMR (600 MHz, CDCl_3_) δ 7.83 (d, 1H, *J* = 7.2 Hz), 7.71 (ddd, 1H, *J* = 7.6, 7.6, 1.2 Hz), 7.61–7.57 (m, 2H), 7.56–7.53 (m, 3H), 7.51–7.48 (m, 1H), 5.19 (d, 1H, *J* = 19.8 Hz), 4.36 (d, 1H, *J* = 19.8 Hz), 2.41 (s, 3H); ^13^C{1H} NMR (150 MHz, CDCl_3_) δ 203.3, 139.1, 137.6, 136.8, 136.5, 133.9, 130.9, 130.6, 130.4, 130.3, 130.2, 129.8, 129.0, 64.0, 40.5; IR (ATR) 1686, 1337, 1153 cm^–1^; HRMS (ESI-TOF) *m*/*z* calcd for C_15_H_13_NO_3_SK 326.0248 (M+K)^+^, found 326.0252.

#### 5-[(2-Nitrophenyl)sulfonyl]-5,6-dihydro-7*H*-dibenzo[*b,d*]vazepin-7-one (**IIAe**)


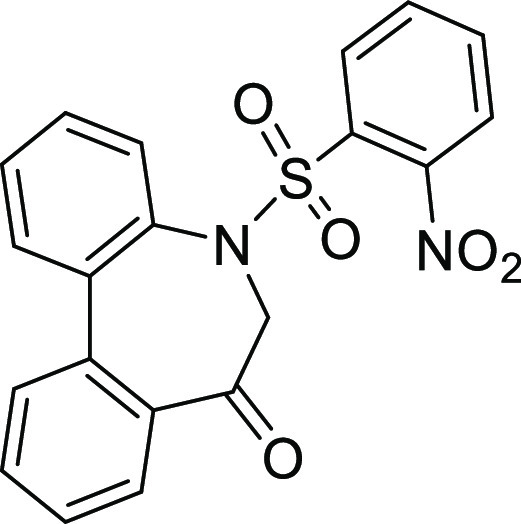
Compound **IIAe** was prepared according to a similar procedure as described for the preparation of **IAg** from **1Ag**. Colorless crystal (28.6 mg, 74%), mp 194–195 °C: ^1^H NMR (600 MHz, CDCl_3_) δ 7.62–7.58 (m, 2H), 7.54 (ddd, 1H, *J* = 7.8, 7.8, 1.8 Hz), 7.50 (ddd, 1H, *J* = 7.6, 7.6, 1.2 Hz), 7.44–7.39 (m, 3H), 7.32 (dd, 1H, *J* = 7.8, 7.8 Hz), 7.28–7.25 (m, 2H), 7.18 (dd, 1H, *J* = 7.2, 7.2 Hz), 7.01 (d, 1H, *J* = 7.2 Hz), 5.43 (d, 1H, *J* = 19.6 Hz), 4.46 (d, 1H, *J* = 19.6 Hz); ^13^C{1H} NMR (150 MHz, CDCl_3_) δ 202.6, 146.9, 139.5, 137.0, 136.5, 135.9, 133.5, 133.0, 132.9, 131.6, 131.2, 131.0, 130.8, 130.7, 130.1, 128.9, 128.3, 128.2, 124.1, 64.7; IR (ATR) 1688, 1541, 1362, 1297, 1166 cm^–1^; HRMS (ESI-TOF) *m*/*z* calcd for C_20_H_14_N_2_O_5_SNa 417.0516 (M+Na)^+^, found 417.0522.

#### 5-[(4-Nitrophenyl)sulfonyl]-5,6-dihydro-7*H*-dibenzo[*b,d*]azepin-7-one (**IIAf**)


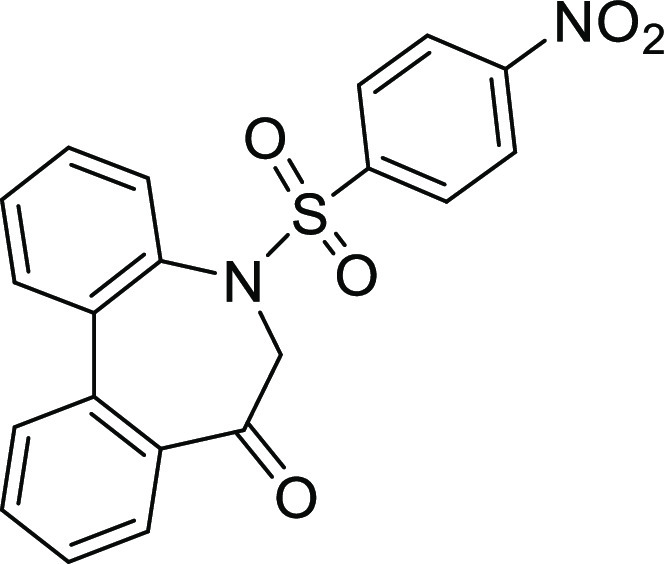
Compound **IIAf** was prepared according to a similar procedure as described for the preparation of **IAg** from **1Ag**. Colorless crystal (386.4 mg, 69%), mp 168–169 °C: ^1^H NMR (600 MHz, CDCl_3_) δ 7.84 (ddd, 2H, *J* = 9.0, 2.4, 2.4 Hz), 7.71–7.69 (m, 1H), 7.55–7.51 (m, 3H), 7.43–7.40 (m, 3H), 7.34 (ddd, 1H, *J* = 7.2, 7.2, 1.8 Hz), 7.27 (ddd, 1H, *J* = 7.2, 7.2, 1.8 Hz), 6.96 (d, 1H, *J* = 7.2 Hz), 5.33 (d, 1H, *J* = 19.2 Hz), 4.45 (d, 1H, *J* = 19.2 Hz); ^13^C{1H} NMR (150 MHz, CDCl_3_) δ 201.5, 149.8, 145.2, 138.8, 137.1, 136.0, 135.9, 133.4, 131.1, 131.0, 130.7, 130.4, 130.2, 129.6, 128.5, 127.9, 123.8, 64.0; IR (ATR) 1686, 1523, 1357, 1348, 1171 cm^–1^; HRMS (APCI-TOF) *m*/*z* calcd for C_22_H_20_NO_4_S 394.0551 (M–H)^−^, found 393.0552.

#### *N*-(3-Methyl-[1,1′-biphenyl]-2-yl)-*N*-tosylglycine (**1Bc**)


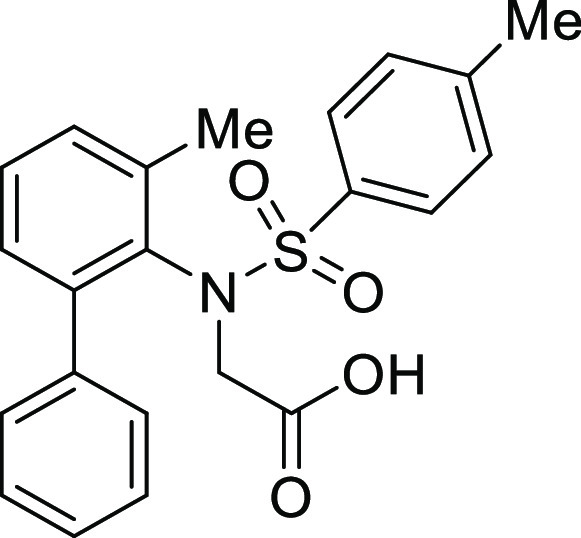
Compound **1Bc** was prepared according to a similar procedure as described for the preparation of **1Ac** from **S10c**. Colorless crystal (195.0 mg, 93%), mp 208–210 °C: ^1^H NMR (600 MHz, CDCl_3_) δ 7.56 (d, 2H, *J* = 8.4 Hz), 7.33–7.30 (m, 1H), 7.29–7.26 (m, 3H), 7.25–7.22 (m, 5H), 7.09 (dd, 1H, *J* = 7.8, 2.4 Hz), 4.11 (d, 1H, *J* = 17.6 Hz), 3.97 (d, 1H, *J* = 17.6 Hz), 2.44 (s, 3H), 2.23 (s, 3H); ^13^C{1H} NMR (150 MHz, CDCl_3_) δ 171.1, 144.2, 143.4, 139.7, 139.5, 137.0, 136.5, 131.3, 130.0, 129.7, 129.6, 128.6, 128.3, 128.0, 127.6, 53.4, 21.7, 20.0; IR (ATR) 3201, 1730, 1337, 1151 cm^–1^; HRMS (ESI-TOF) *m*/*z* calcd for C_22_H_21_NO_4_SNa 418.1084 (M+Na)^+^, found 418.1086.

#### *N*-(3-Methyl-[1,1′-biphenyl]-2-yl)-*N*-(methylsulfonyl)glycine (**1Bd**)


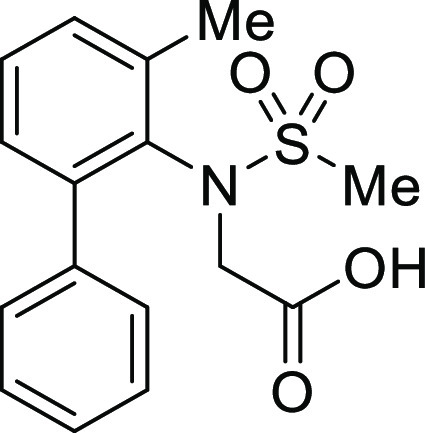
Compound **1Bd** was prepared according to a similar procedure as described for the preparation of **1Ac** from **S10c**. Colorless crystal (192.9 mg, 86%), mp 162–165 °C: ^1^H NMR (600 MHz, CDCl_3_) δ 7.41–7.36 (m, 5H), 7.31–7.29 (m, 2H), 7.17–7.14 (m, 1H), 4.42 (d, 1H, *J* = 18.0 Hz), 4.03 (d, 1H, *J* = 18.0 Hz), 2.75 (s, 3H), 2.54 (s, 3H); ^13^C{1H} NMR (150 MHz, CDCl_3_) δ 173.5, 142.7, 139.9, 139.3, 138.4, 131.4, 129.8, 129.7, 128.7, 128.2, 127.9, 53.3, 42.2, 19.9; IR (ATR) 3020, 1758, 1327, 1138 cm^–1^; HRMS (ESI-TOF) *m*/*z* calcd for C_16_H_17_NO_4_SNa 342.0771 (M+Na)^+^, found 342.0774.

#### *N*-(3-Methyl-[1,1′-biphenyl]-2-yl)-*N*-([2-nitrophenyl]sulfonyl)glycine (**1Be**)


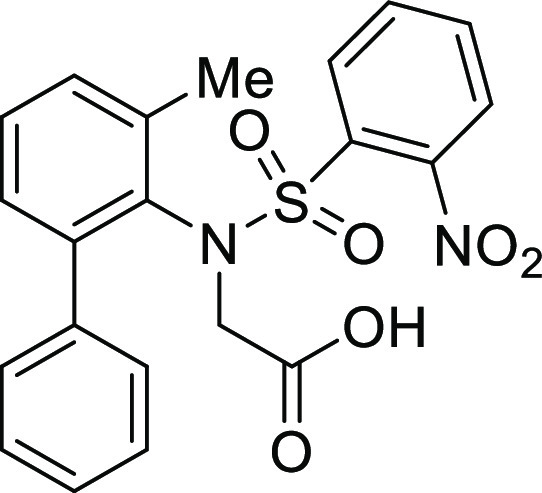
Compound **1Be** was prepared according to a similar procedure as described for the preparation of **1Ac** from **S10c**. Colorless crystal (150.5 mg, 68%), mp 199–201 °C: ^1^H NMR (600 MHz, CDCl_3_) δ 7.70 (d, 1H, *J* = 7.8 Hz), 7.66 (dd, 1H, *J* = 7.8, 7.8 Hz), 7.55–7.52 (m, 2H), 7.32–7.28 (m, 3H), 7.23 (dd, 2H, *J* = 7.8, 7.8 Hz), 7.16 (d, 2H, *J* = 7.8 Hz), 7.06 (d, 1H, *J* = 7.2 Hz), 4.52 (d, 1H, *J* = 18.0 Hz), 4.16 (d, 1H, *J* = 18.0 Hz), 2.41 (s, 3H); ^13^C{1H} NMR (150 MHz, CDCl_3_) δ 171.1, 148.5, 143.5, 140.1, 139.6, 136.4, 133.9, 133.7, 132.0, 131.7, 131.6, 130.3, 129.5, 129.0, 128.1, 127.7, 124.3, 54.2, 20.3; IR (ATR) 3028, 1708, 1543, 1366, 1364, 1166 cm^–1^; HRMS (ESI-TOF) *m*/*z* calcd for C_21_H_18_N_2_O_6_SNa 449.0778 (M+Na)^+^, found 449.0781.

#### *N*-(3-Methyl-[1,1′-biphenyl]-2-yl)-*N*-([4-nitrophenyl]sulfonyl)glycine (**1Bf**)


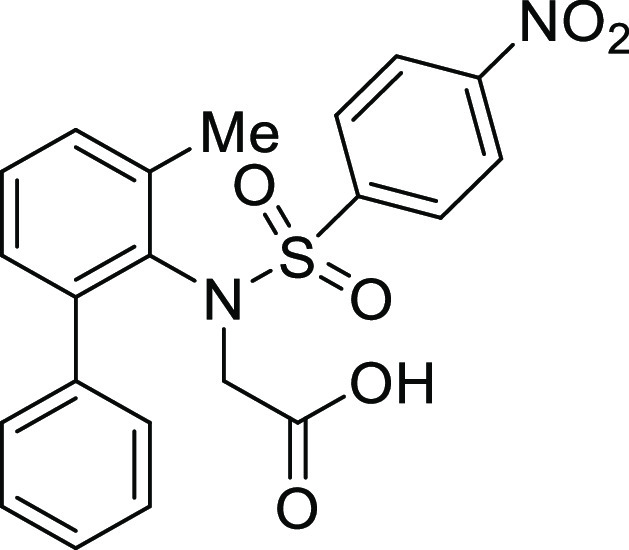
Compound **1Bf** was prepared according to a similar procedure as described for the preparation of **1Ac** from **S10c**. Colorless crystal (235.4 mg, 77%), mp 185–187 °C: ^1^H NMR (600 MHz, CDCl_3_) δ 8.14 (ddd, 2H, *J* = 9.0, 2.4, 2.4 Hz), 7.78 (ddd, 2H, *J* = 9.0, 2.4, 2.4 Hz), 7.33–7.28 (m, 3H), 7.22 (dd, 2H, *J* = 7.8, 7.8 Hz), 7.19 (dd, 2H, *J* = 7.8, 1.2 Hz), 7.09 (dd, 1H, *J* = 6.6, 1.8 Hz), 4.29 (d, 2H, *J* = 1.8 Hz), 2.37 (s, 3H); ^13^C{1H} NMR (150 MHz, CDCl_3_) δ 171.7, 150.1, 145.6, 143.1, 139.8, 139.4, 136.7, 131.6, 130.3, 129.5, 129.2, 128.0, 127.7, 123.8, 53.9, 20.1; IR (ATR) 3318, 1775, 1528, 1339, 1307, 1138 cm^–1^; HRMS (ESI-TOF) *m*/*z* calcd for C_21_H_17_N_2_O_6_S 425.0813 (M–H)^−^, found 425.0812.

#### 4-Methyl-5-tosyl-5,6-dihydro-7*H*-dibenzo[*b,d*]azepin-7-one (**IIBc**)


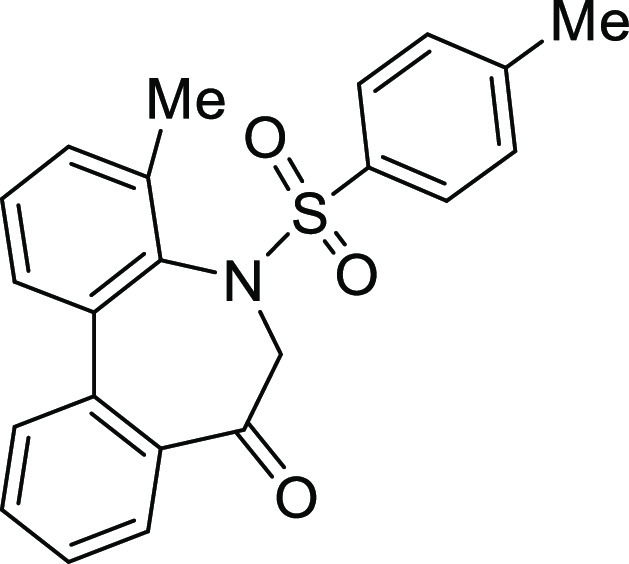
Compound **IIBc** was prepared according to a similar procedure as described for the preparation of **IAg** from **1Ag**. Colorless crystal (25.3 mg, 99%), mp 159–160 °C: ^1^H NMR (600 MHz, CDCl_3_) δ 7.42–7.36 (m, 3H), 7.27 (d, 1H, *J* = 1.8 Hz), 7.23–7.16 (m, 5H), 6.83 (d, 2H, *J* = 7.8 Hz), 5.12 (d, 1H, *J* = 19.6 Hz), 4.31 (d, 1H, *J* = 19.6 Hz), 2.57 (s, 3H), 2.28 (s, 3H); ^13^C{1H} NMR (150 MHz, CDCl_3_) δ 203.3, 142.9, 140.1, 140.0, 138.3, 137.5, 136.4, 136.2, 133.0, 131.9, 130.0, 129.8, 129.5, 129.3, 129.0, 127.8, 126.9, 63.0, 21.5, 19.3; IR (ATR) 1682, 1344, 1161 cm^–1^; HRMS (ESI-TOF) *m*/*z* calcd for C_22_H_19_NO_3_SNa 400.0978 (M+Na)^+^, found 400.0980. Separation of atropisomers. CHIRALPAK IB (1.0 cmϕ × 25 cm): eluent, 30% 2-propanol in hexane; flow rate, 0.3 mL/min; temperature, 25 °C; detection, 254 nm; former peak, retention time = 24.1 min; [α]_D_^20^ −65.8 as 96.8% ee (*c* 0.23, CHCl_3_); latter peak, retention time = 28.5 min; [α]_D_^20^ +64.9 as 96.1% ee (*c* 0.21, CHCl_3_).

#### 4-Methyl-5-(methylsulfonyl)-5,6-dihydro-7*H*-dibenzo[*b*,*d*]azepin-7-one (**IIBd**)


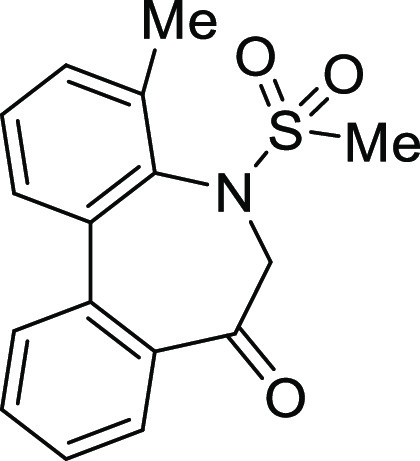
Compound **IIBd** was prepared according to a similar procedure as described for the preparation of **IAg** from **1Ag**. Colorless crystal (28.0 mg, 72%), mp 177–178 °C: ^1^H NMR (600 MHz, CDCl_3_) δ 7.76 (dd, 1H, *J* = 8.4, 1.6 Hz), 7.70 (ddd, 1H, *J* = 7.8, 7.8, 1.2 Hz), 7.59 (dd, 1H, *J* = 7.8, 1.2 Hz), 7.52 (dd, 1H, *J* = 7.6, 7.6 Hz), 7.42 (dd, 1H, *J* = 7.8, 7.8 Hz), 7.39–7.35 (m, 2H), 5.08 (d, 1H, *J* = 20.0 Hz), 4.32 (d, 1H, *J* = 20.0 Hz), 2.52 (s, 3H), 2.28 (s, 3H); ^13^C{1H} NMR (150 MHz, CDCl_3_) δ 203.6, 139.9, 139.8, 138.4, 136.9, 135.6, 133.9, 132.2, 130.2, 130.1, 129.5, 129.0, 128.8, 63.6, 40.5, 19.1; IR (ATR) 1683, 1337, 1151 cm^–1^; HRMS (ESI-TOF) *m*/*z* calcd for C_16_H_15_NO_3_SNa 324.0665 (M+Na)^+^, found 324.0665. Separation of atropisomers. CHIRALPAK IA (1.0 cmϕ × 25 cm): eluent, 30% 2-propanol in hexane; flow rate, 0.5 mL/min; temperature, 25 °C; detection, 254 nm; former peak, retention time = 30.0 min; [α]_D_^20^ +89.8 as 97.0% ee (*c* 0.23, CHCl_3_),; latter peak, retention time = 34.9 min; [α]_D_^20^ −90.8 as 98.7% ee (*c* 0.27, CHCl_3_).

#### 4-Methyl-5-([2-nitrophenyl]sulfonyl)-5,6-dihydro-7*H*-dibenzo[*b*,*d*]azepin-7-one (**IIBe**)


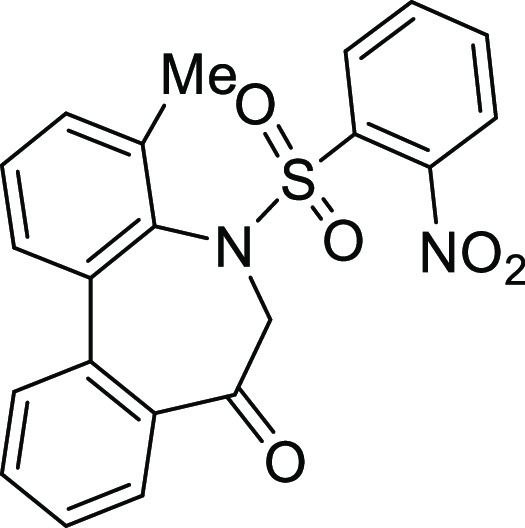
Compound **IIBe** was prepared according to a similar procedure as described for the preparation of **IAg** from **1Ag**. Colorless crystal (61.0 mg, 83%), mp 185–187 °C: ^1^H NMR (600 MHz, CDCl_3_) δ 7.49 (dd, 1H, *J* = 7.2, 1.2 Hz), 7.45–7.39 (m, 4H), 7.32 (ddd, 1H, *J* = 7.8, 7.8, 1.2 Hz), 7.27 (dd, 1H, *J* = 8.4, 1.8 Hz), 7.24–7.21 (m, 2H), 7.12–7.09 (m, 2H), 5.40 (d, 1H, *J* = 19.8 Hz), 4.36 (d, 1H, *J* = 19.8 Hz), 2.51 (s, 3H); ^13^C{1H} NMR (150 MHz, CDCl_3_) δ 202.8, 147.0, 140.2, 139.9, 137.3, 137.1, 135.0, 133.6, 133.4, 132.8, 132.2, 131.7, 131.2, 130.4, 129.7, 129.2, 128.9, 128.1, 124.1, 64.1, 19.0; IR (ATR) 1688, 1533, 1356, 1356, 1166 cm^–1^; HRMS (ESI-TOF) *m*/*z* calcd for C_21_H_17_N_2_O_5_S 409.0853 (M+H)^+^, found 409.0856. Separation of atropisomers. CHIRALPAK IA (1.0 cmϕ × 25 cm): eluent, 30% 2-propanol in hexane; flow rate, 0.5 mL/min; temperature, 25 °C; detection, 254 nm; former peak, retention time = 19.1 min; [α]_D_^20^ +61.5 as 99.8% ee (*c* 0.55, CHCl_3_); latter peak, retention time = 26.2 min; [α]_D_^20^ −61.1 as 99.8% ee (*c* 0.90, CHCl_3_).

#### 4-Methyl-5-([4-nitrophenyl]sulfonyl)-5,6-dihydro-7*H*-dibenzo[*b*,*d*]azepin-7-one (**IIBf**)


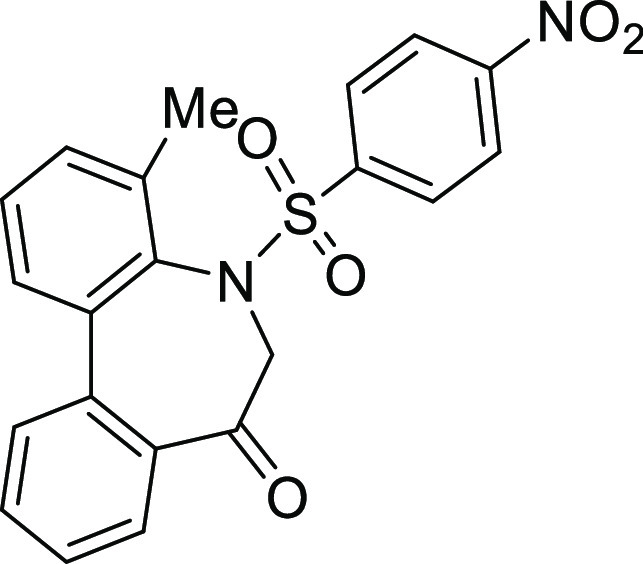
Compound **IIBf** was prepared according to a similar procedure as described for the preparation of **IAg** from **1Ag**. Colorless crystal (77.2 mg, 83%), mp 184–186 °C: ^1^H NMR (600 MHz, CDCl_3_) δ 7.85 (ddd, 2H, *J* = 8.4, 2.4, 2.4 Hz), 7.47 (ddd, 2H, *J* = 9.0, 2.4, 2.4 Hz), 7.42 (d, 2H, *J* = 4.8 Hz), 7.36 (ddd, 1H, *J* = 7.6, 7.6, 1.2 Hz), 7.31 (dd, 1H, *J* = 7.8, 1.2 Hz), 7.22 (dd, 1H, *J* = 4.6, 4.6 Hz), 7.17 (ddd, 1H, *J* = 7.6, 7.6, 1.2 Hz), 7.11 (dd, 1H, *J* = 7.8, 1.2 Hz), 5.19 (d, 1H, *J* = 19.8 Hz), 4.38 (d, 1H, *J* = 19.8 Hz), 2.61 (s, 3H); ^13^C{1H} NMR (150 MHz, CDCl_3_) δ 201.8, 149.7, 145.6, 140.1. 139.5, 138.0, 136.1, 135.2, 133.4, 132.4, 130.4, 130.1, 129.4, 129.1, 128.4, 128.1, 124.0, 63.3, 19.2; IR (ATR) 1683, 1523, 1351, 1351, 1166 cm^–1^; HRMS (ESI-TOF) *m*/*z* calcd for C_21_H_17_N_2_O_5_S 409.0853 (M+H)^+^, found 409.0853. Separation of atropisomers. CHIRALPAK IA (1.0 cmϕ × 25 cm): eluent, 30% 2-propanol in hexane; flow rate, 0.5 mL/min; temperature, 25 °C; detection, 254 nm; former peak, retention time = 22.5 min; [α]_D_^20^ +27.2 as 98.5% ee (*c* 0.34, CHCl_3_); latter peak, retention time = 30.1 min; [α]_D_ −27.9 as 99.8% ee (*c* 0.07, CHCl_3_).

#### 5,6-Dihydro-7*H*-dibenzo[*b*,*d*]azepin-7-one (**2**)


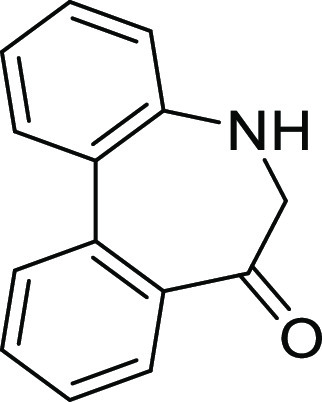
K_2_CO_3_ (45.2 mg, 0.32 mmol) was added to a stirred solution of **IAg** (50.0 mg, 0.16 mmol) in MeOH/H_2_O = 5:1 (1.6 mL) at reflux under an argon atmosphere. After being stirred at reflux for 30 min, the mixture was treated with 1 M NaHCO_3_ aq. and brine, dried, and concentrated. The concentrate was purified by column chromatography (silica gel, hexane/ethyl acetate = 4:1) to afford **2** as yellow oil (33.0 mg, 99%): ^1^H NMR (600 MHz, CDCl_3_) δ 7.94 (dd, 1H, *J* = 7.8, 1.2 Hz), 7.63 (ddd, 1H, *J* = 7.8, 7.8, 1.2 Hz), 7.59 (dd, 1H, *J* = 7.2, 1.2 Hz), 7.50 (dd, 1H, *J* = 7.2, 1.8 Hz), 7.44 (ddd, 1H, *J* = 7.2, 7.2, 1.2 Hz), 7.31 (ddd, 1H, *J* = 7.2, 7.2, 1.2 Hz), 7.22 (ddd, 1H, *J* = 7.2, 7.2, 1.2 Hz), 7.02 (dd, 1H, *J* = 7.8, 1.2 Hz), 4.16 (s, 2H); ^13^C{1H} NMR (150 MHz, CDCl_3_) δ 204.3, 147.5, 138.8, 136.9, 133.0, 132.6, 130.8, 130.5, 129.6, 129.5, 127.7, 124.6, 121.1, 63.8; IR (ATR) 3335, 1665 cm^–1^; HRMS (ESI-TOF) *m*/*z*: calcd for C_14_H_12_NO 210.0913 (M+H)^+^, found 210.0914.

#### 5-Acetyl-5,6-dihydro-7*H*-dibenzo[*b*,*d*]azepin-7-one (**IAa**)


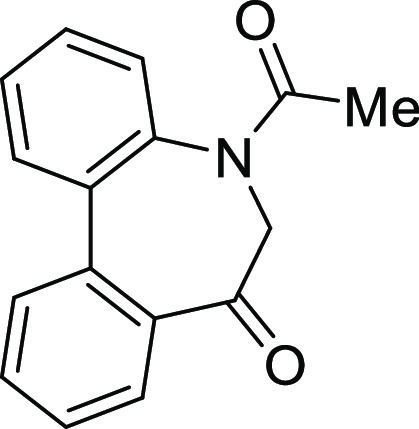
Acetyl chloride (30.7 μL, 0.43 mmol) and pyridine (47.7 μL, 0.58 mmol) were added to a stirred solution of **2** (60.5 mg, 0.29 mmol) in THF (3 mL) at 0 °C under an argon atmosphere. The mixture was stirred at rt for 1.5 h, poured into aqueous HCl, and extracted with ethyl acetate. The organic phase was washed with 1 M HCl aq., 1 M NaHCO_3_ aq., and brine, then dried, and concentrated. The residue was purified by column chromatography (silica gel, hexane/EtOAc = 1:2) to afford **IAa** as colorless crystals (24.0 mg, 33%), mp 126–127 °C: ^1^H NMR (600 MHz, CDCl_3_); *E*-isomer: δ 7.77 (d, 1H, *J* = 6.6 Hz), 7.65 (ddd, 1H, *J* = 7.2, 7.2, 1.2 Hz), 7.60 (dd, 1H, *J* = 7.2, 1.2 Hz), 7.53 (ddd, 1H, *J* = 8.4, 8.4, 1.8 Hz), 7.50–7.47 (m, 3H), 7.36 (dd, 1H, *J* = 7.8, 1.8 Hz), 5.71 (d, 1H, *J* = 18.6 Hz), 3.95 (d, 1H, *J* = 18.6 Hz), 1.79 (s, 3H); *Z*-isomer: δ 7.77 (d, 1H, *J* = 6.6 Hz), 7.65 (ddd, 1H, *J* = 7.2, 7.2, 1.2 Hz), 7.60 (dd, 1H, *J* = 7.2, 1.2 Hz), 7.53 (ddd, 1H, *J* = 8.4, 8.4, 1.8 Hz), 7.50–7.47 (m, 3H), 7.36 (dd, 1H, *J* = 7.8, 1.8 Hz), 4.88 (d, 1H, *J* = 19.6 Hz), 4.34 (d, 1H, *J* = 19.6 Hz), 1.79 (s, 3H); ^13^C{1H} NMR (150 MHz, CDCl_3_) δ 203.5, 170.2, 140.1, 138.2, 136.5, 136.4, 133.2, 130.8, 129.9, 129.8, 129.7, 129.2, 128.9, 127.6, 60.7, 21.9; IR (ATR) 1667, 1596 cm^–1^; HRMS (ESI-TOF) *m*/*z* calcd for C_16_H_14_NO_2_ 252.1019 (M+H)^+^, found 252.1020.

### Preparation of Starting Materials (**1Aa**–**h**, **1Bc**–**h**)

*N*-Acyl-/sulfonyl-(1,1′)-biphenyl-2-yl-glycines **1Aa**–**j**, **1Be**–**j** were prepared in accordance with the method reported in a previous paper.^4^ The characterization of the intermediates **S1a**, **S1b**, **S1g**, **S1h**, **S2a**, **S2b**, **S2g**, **S2h**, **S3**, **S4**, **S5g**, **S5h**, **S6h**, **S7c**, **S7d**, **S7e**, **S7f**, **S8c**, **S8d**, **S8e**, **S8f**, **S9c**, **S9d**, **S9e**, **S9f**, **S10c**, **S10d**, **S10e**, and **S10f** are described in the Experimental Section. The reaction schemes are described in the Supporting Information.

#### *N*-([1,1′-Biphenyl]-2-yl)acetamide (**S1a**)


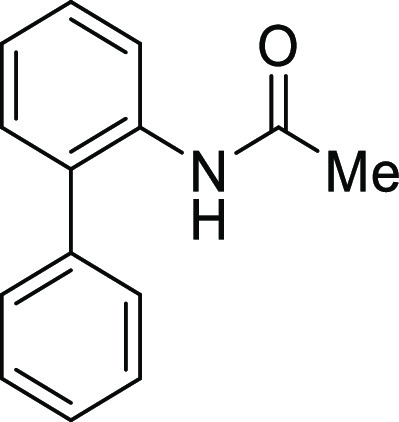
Acetyl chloride (2.33 mL, 33 mmol) and pyridine (3.67 mL, 45 mmol) were added to a stirred solution of 2-aminobiphenyl (5.02 g, 30 mmol) in THF (30 mL) at 0 °C under an argon atmosphere. After stirring at rt for 30 min, the mixture was treated with 1 M HCl aq. and extracted with ethyl acetate. The extract was washed with 1 M HCl aq., 1 M NaHCO_3_ aq., and brine, dried, and concentrated. The concentrate was purified by column chromatography (silica gel, hexane/ethyl acetate = 1:1) to afford **S1a** as colorless crystals (5.47 g, 87%), mp 108–110 °C: ^1^H NMR (600 MHz, CDCl_3_) δ 8.26 (d, 1H, *J* = 8.4 Hz), 7.49 (dd, 1H, *J* = 7.6, 7.6 Hz), 7.42 (dd, 1H, *J* = 7.2, 7.2 Hz), 7.38–7.36 (m, 3H), 7.24 (d, 1H, *J* = 7.8 Hz), 7.18 (dd, 1H *J* = 7.6, 7.6 Hz), 7.13 (br, 1H), 2.02 (s, 3H); ^13^C{1H} NMR (150 MHz, CDCl_3_) δ 168.4, 138.3, 134.8, 132.3, 130.2, 129.4, 129.2, 128.6, 128.1, 124.5, 121.8, 24.7; IR (ATR) 3287, 1659 cm^–1^; HRMS (ESI-TOF) *m*/*z* calcd for C_14_H_14_NO 212.1070 (M+H)^+^, found 212.1074.

#### *N*-([1,1′-Biphenyl]-2-yl)-4-methylbenzamide (**S1b**)


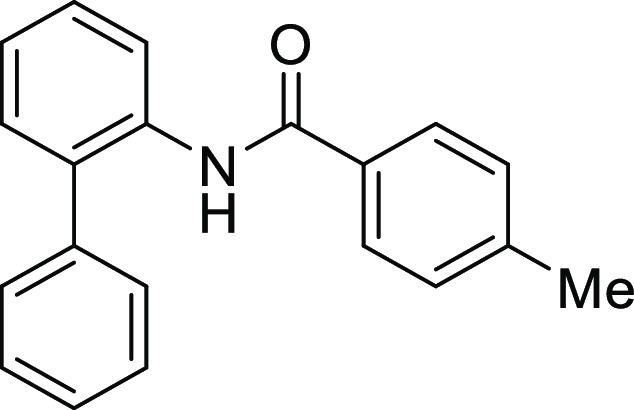
Compound **S1b** was prepared according to a similar procedure as described for the preparation of **S1a** from 2-aminobiphenyl. Colorless crystal (6.81 g, 96%), mp 95–96 °C: ^1^H NMR (600 MHz, CDCl_3_) δ 8.55 (d, 1H, *J* = 8.4 Hz), 7.97 (br, 1H), 7.52–7.48 (m, 4H), 7.45–7.42 (m, 4H), 7.30 (dd, 1H, *J* = 7.8, 1.2 Hz), 7.21 (ddd, 1H, *J* = 7.2, 7.2, 1.2 Hz), 7.19 (d, 2H, *J* = 7.8 Hz), 2.37 (s, 3H); ^13^C{1H} NMR (150 MHz, CDCl_3_) δ 165.1, 142.4, 138.3, 135.2, 132.3, 132.1, 130.1, 129.5, 129.4, 128.8, 128.3, 127.0, 124.3, 121.2, 21.6; IR (ATR) 3311, 1709 cm^–1^; HRMS (ESI-TOF) *m*/*z* calcd for C_20_H_18_NO 288.1383 (M+H)^+^, found 288.1391.

#### *N*-([1,1′-Biphenyl]-2-yl)-2,2,2-trifluoroacetamide (**S1g**)


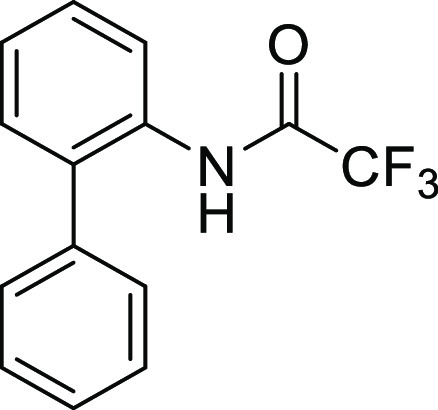
TFAA (168.0 μL, 1.20 mmol) was added to a solution of 2-aminobiphenyl (169.0 mg, 1.00 mmol) in CH_2_Cl_2_ (5.00 mL) at 0 °C. After stirring at rt for 20 min, the mixture was treated with 1 M HCl aq. and extracted with ethyl acetate. The extract was washed with 1 M HCl aq., 1 M NaHCO_3_ aq., and brine, dried, and concentrated. The concentrate was purified by recrystallization to afford **S1g** as colorless crystals (263.0 mg, 99%), mp 88–89 °C: ^1^H NMR (600 MHz, CDCl_3_) δ 8.29 (d, 1H, *J* = 8.4 Hz), 7.99 (br, 1H), 7.53–7.51 (m, 2H), 7.47–7.43 (m, 2H), 7.37–7.29 (m, 4H); ^13^C{1H} NMR (150 MHz, CDCl_3_) δ 154.7 (C–F, ^2^*J*_C–F_ = 37.6 Hz), 136.9, 133.3, 132.3, 130.5, 129.6, 129.2, 128.9, 128.8, 126.4, 121.5, 115.8 (C–F, ^1^*J*_C–F_ = 289.0 Hz); IR (ATR) 3244, 1709 cm^–1^; HRMS (ESI-TOF) *m*/*z* calcd for C_14_H_9_F_3_NO 264.0642 (M–H)^−^, found 264.0639.

#### Methyl [1,1′-Biphenyl]-2-ylcarbamate (**S1h**)


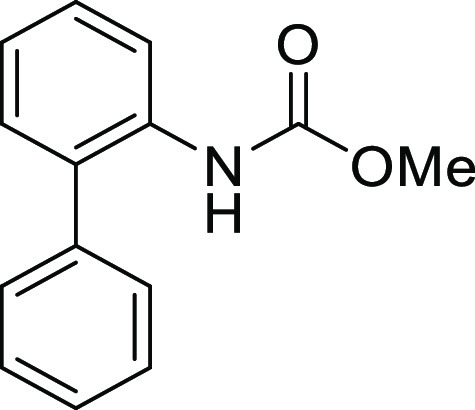
Compound **S1h** was prepared according to a similar procedure as described for the preparation of **S1a** from 2-aminobiphenyl. Colorless crystal (421.3 mg, 93%), mp 56–57 °C: ^1^H NMR (600 MHz, CDCl_3_) δ 8.14 (br, 1H), 7.48 (dd, 2H, *J* = 7.8, 7.8 Hz), 7.42–7.34 (m, 4H), 7.22 (dd, 1H, *J* = 7.2, 1.2 Hz), 7.13 (ddd, 1H, *J* = 7.4, 7.4, 1.2 Hz), 3.71 (s, 3H), 6.66 (br, 1H); ^13^C{1H} NMR (150 MHz, CDCl_3_) δ 154.1, 138.2, 134.9, 131.5, 130.3, 129.4, 129.3, 128.6, 128.1, 123.5, 119.7, 52.4; IR (ATR) 3412, 1729 cm^–1^; HRMS (ESI-TOF) *m*/*z* calcd for C_14_H_14_NO_2_ 228.1019 (M+H)^+^, found 228.1010.

#### Methyl *N*-([1,1′-Biphenyl]-2-yl)-*N*-acetylglycinate (**S2a**)


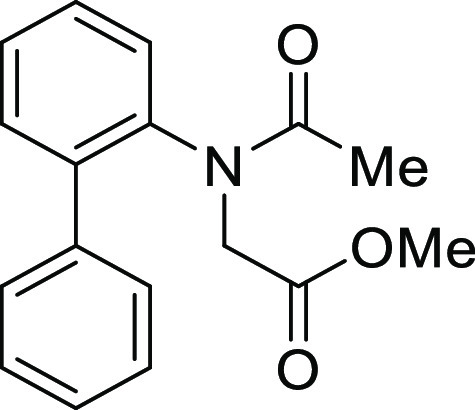
Sodium hydride (60% oil) (1.5 mg, 37.5 mmol) was added to a stirred solution of **S1a** (5.30 g, 25.0 mmol) in DMF (50.0 mL) at 0 °C under an argon atmosphere. After stirring at rt for 20 min, the mixture was cooled to 0 °C and treated with methyl bromoacetate (3.46 mL, 37.5 mmol). After being stirred at rt for 1 h, the mixture was treated with 1 M HCl aq. and extracted with ethyl acetate. The extract was washed with 1 M HCl aq., 1 M NaHCO_3_ aq., and brine, dried, and concentrated. The concentrate was purified by column chromatography (silica gel, hexane/EtOAc = 1:2) to afford **S2a** as colorless crystals (7.76 g, 99%), mp 112–114 °C: ^1^H NMR (600 MHz, CDCl_3_) δ 7.59 (dd, 1H, *J* = 7.8, 1.8 Hz), 7.44–7.39 (m, 5H), 7.38–7.35 (m, 1H), 7.27–7.25 (m, 2H), 4.54 (d, 1H, *J* = 17.4 Hz), 3.68 (s, 3H), 3.30 (d, 1H, *J* = 17.4 Hz), 1.96 (s, 3H); ^13^C{1H} NMR (150 MHz, CDCl_3_) δ 171.3, 169.7, 140.5, 139.5, 138.5, 131.4, 130.1, 129.0, 128.9, 128.6, 128.0, 52.2, 50.7, 22.3; IR (ATR) 1749, 1658 cm^–1^; HRMS (ESI-TOF) *m*/*z* calcd for C_17_H_17_NO_3_Na 306.1101 (M+Na)^+^, found 306.1107.

#### Methyl *N*-([1,1′-Biphenyl]-2-yl)-*N*-(4-methylbenzoyl)glycinate (**S2b**)


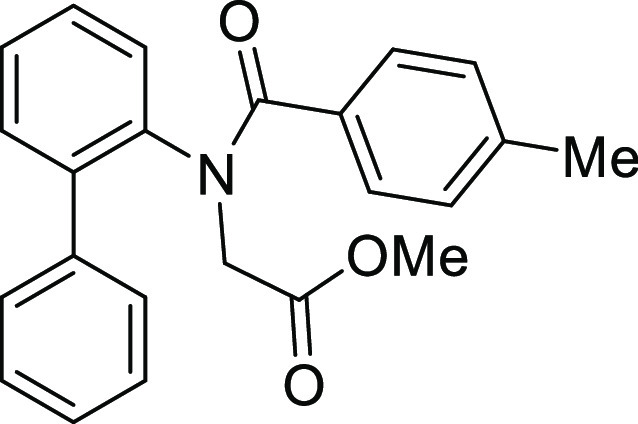
Compound **S2b** was prepared according to a similar procedure as described for the preparation of **S2a** from **S1a**. Colorless crystal (3.03 g, 82%), mp 99–100 °C: ^1^H NMR (600 MHz, CDCl_3_) δ 7.39–7.34 (m, 4H), 7.31 (d, 2H, *J* = 4.2 Hz), 7.25–7.23 (m, 1H), 7.21 (dd, 2H, *J* = 7.8, 1.2 Hz), 7.09 (d, 2H, *J* = 8.4 Hz), 6.91 (d, 2H, *J* = 7.8 Hz), 4.78 (d, 1H, *J* = 17.4 Hz), 3.74 (s, 3H), 3.65 (d, 1H, *J* = 17.4 Hz), 2.27 (s, 3H); ^13^C{1H} NMR (150 MHz, CDCl_3_) δ 170.2, 169.9, 141.5, 140.5, 138.5, 138.1, 131.6, 131.3, 129.9, 129.2, 128.9, 128.7, 128.5, 128.3, 128.1, 127.8, 52.5, 52.3, 21.5; IR (ATR) 1604, 1607 cm^–1^; HRMS (ESI-TOF) *m*/*z* calcd for C_23_H_22_NO_3_ 360.1594 (M+H)^+^, found 360.1594.

#### Benzyl *N*-([1,1′-Biphenyl]-2-yl)-*N*-(2,2,2-trifluoroacetyl)glycinate (**S2g**)


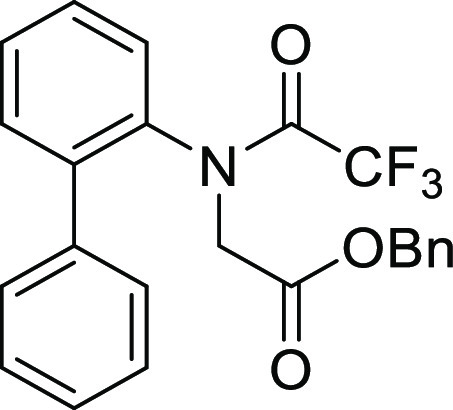
K_2_CO_3_ (124.4 mg, 0.90 mmol) was added to a stirred solution of **S1g** (159.0 mg, 0.60 mmol) in DMF (1.0 mL) at rt under an argon atmosphere and treated with benzyl bromoacetate (75.0 μL, 0.8 mmol) for 2 days. The reaction mixture was poured into water and extracted with ethyl acetate. The extract was washed with brine, dried, and concentrated. The concentrate was dissolved in CH_2_Cl_2_, and TFAA (252.0 μL, 1.80 mmol) was added at 0 °C under an argon atmosphere for 1 h. The mixture was treated with 1 M HCl aq. and extracted with ethyl acetate. The extract was washed with 1 M HCl aq., 1 M NaHCO_3_ aq., and brine, dried, and concentrated. The concentrate was purified by column chromatography (silica gel, hexane/ethyl acetate = 4:1) to afford **S2g** as colorless crystals (149.0 mg, 82%), mp 82–83 °C: ^1^H NMR (600 MHz, CDCl_3_) δ 7.54 (d, 1H, *J* = 8.4 Hz), 7.48 (ddd, 1H, *J* = 7.6, 7.6, 1.2 Hz), 7.43–7.33 (m, 8H), 7.29–7.27 (m, 4H), 5.14 (d, 1H, *J* = 12.4 Hz), 5.09 (d, 1H, *J* = 12.4 Hz), 4.41 (d, 1H, *J* = 17.1 Hz), 3.41 (d, 1H, *J* = 17.1 Hz); ^13^C{1H} NMR (150 MHz, CDCl_3_) δ 167.2, 158.1 (C–F, ^2^*J*_C–F_ = 36.1 Hz), 139.3, 137.9, 137.1, 135.0, 131.5, 129.9 (C–F, ^5^*J*_C–F_ = 5.8 Hz), 129.1, 128.9, 128.8, 128.7, 128.6, 128.5, 128.4, 128.3, 117.3 (C–F, ^1^*J*_C–F_ = 576.5 Hz), 67.6, 52.5; IR (ATR) 1754, 1698 cm^–1^; HRMS (ESI-TOF) *m*/*z* calcd for C_23_H_18_F_3_NO_3_Na 436.1131 (M+Na)^+^, found 436.1133.

#### Methyl *N*-([1,1′-Biphenyl]-2-yl)-*N*-(methoxycarbonyl)glycinate (**S2h**)


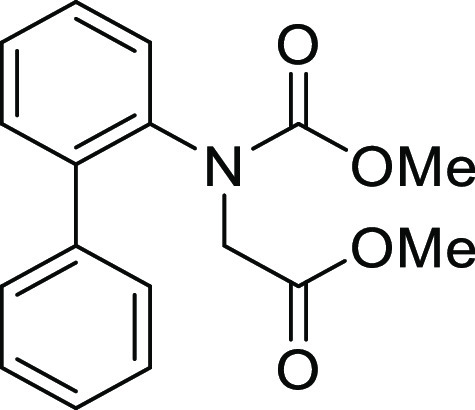
Compound **S2h** was prepared according to a similar procedure described for the preparation of **S2a** from **S1a**. Colorless crystal (492.0 mg, 89%), mp 65–66 °C: ^1^H NMR (600 MHz, CDCl_3_) δ 7.55–7.53 (m, 1H), 7.42–7.40 (m, 2H), 7.39–7.34 (m, 4H), 7.28 (dd, 2H, *J* = 7.2, 1.2 Hz), 4.33 (d, 1H, *J* = 18.0 Hz), 3.69 (s, 3H), 3.65 (s, 3H), 3.35 (d, 1H, *J* = 18.0 Hz); ^13^C{1H} NMR (150 MHz, CDCl_3_) δ 170.1, 156.7, 139.4, 139.2, 139.0, 130.8, 130.4, 129.9, 128.8, 128.7, 128.5, 128.4, 128.2, 127.7, 53.4, 52.2, 51.6; IR (ATR) 1754, 1698 cm^–1^; HRMS (ESI-TOF) *m*/*z* calcd for C_17_H_17_NO_4_Na 322.1050 (M+Na)^+^, found 322.1055.

#### 3-Methyl-2-nitro-(1,1′)-biphenyl (**S3**)


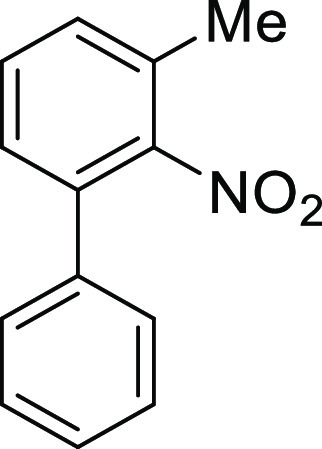
K_2_CO_3_ (1279.8 mg, 9.26 mmol) and Pd(PPh_3_)_4_ (250 mg) were added to a stirred solution of 3-bromo-2-nitrotoluene (1.00 g, 4.63 mmol) and phenylboronic acid (839.9 mg, 6.94 mmol) in DMF/H_2_O = 5:1 (46.0 mL) at reflux under an argon atmosphere. After being stirred at reflux for 16 h, the mixture was treated with 1 M HCl aq. and extracted with ethyl acetate. The extract was washed with 1 M HCl aq. and brine, dried, and concentrated. The concentrate was purified by column chromatography (silica gel, hexane/CH_2_Cl_2_ = 2:1) to afford **S3** as colorless crystals (990.2 mg, 99%), mp 74–75 °C: ^1^H NMR (600 MHz, CDCl_3_) δ 7.44–7.39 (m, 4H), 7.36–7.35 (m, 2H), 7.30 (d, 1H, *J* = 7.2 Hz), 7.26 (d, 1H, *J* = 8.4 Hz), 2.39 (s, 3H); ^13^C{1H} NMR (150 MHz, CDCl_3_) δ 151.0, 136.9, 134.5, 130.4, 130.1, 129.8, 128.9, 128.8, 128.6, 128.2, 17.6; IR (ATR) 1522, 1370 cm^–1^; HRMS (EI-MS) *m*/*z* calcd for C_13_H_11_NO_2_ 213.0790 (M^+^), found 213.0791.

#### 3-Methyl-2-amino-(1,1′)biphenyl (**S4**)


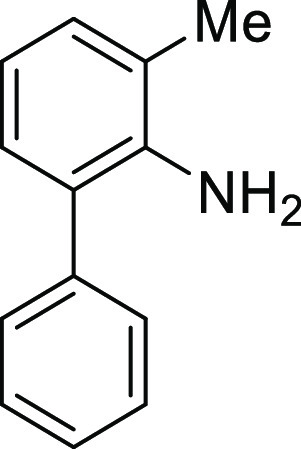
**S3** (935.5 mg, 4.39 mmol) was dissolved in THF/MeOH = 1:1 (10.0 mL), and 10% palladium on activated carbon (93.5 mg, 10% w/w) and hydrogen were added and stirred at rt for 19 h. The mixture was filtered, and the filtrate was washed with water and brine, dried, and concentrated under reduced pressure. The concentrate was purified by recrystallization to provide **S4** (800.3 mg, 99%), mp 64–65 °C: ^1^H NMR (600 MHz, CDCl_3_) δ 7.46–7.43 (m, 4H), 7.36–7.34 (m, 1H), 7.07 (d, 1H, *J* = 6.6 Hz), 7.01 (dd, 1H, *J* = 7.8, 1.2 Hz), 6.77 (dd, 1H, *J* = 7.8, 7.8 Hz), 3.74 (br, 2H), 2.23 (s, 3H); ^13^C{1H} NMR (150 MHz, CDCl_3_) δ 141.7, 140.0, 129.8, 129.4, 129.0, 128.4, 127.7, 127.3, 122.6, 118.3, 18.1; IR (ATR) 3376, 3457 cm^–1^; HRMS (ESI-TOF) *m*/*z* calcd for C_13_H_14_N 184.1121 (M+H)^+^, found 184.1128.

#### 2,2,2-Trifluoro-*N*-(3-methyl-[1,1′-biphenyl]-2-yl)acetamide (**S5g**)


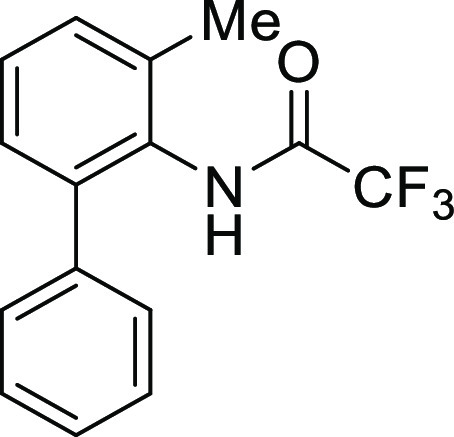
Compound **S5g** was prepared according to a similar procedure as described for the preparation of **S1g** from 2-aminobiphenyl. Colorless crystal (285.0 mg, 99%), mp 152–153 °C: ^1^H NMR (600 MHz, CDCl_3_) δ 7.42–7.35 (m, 3H), 7.33 (d, 1H, *J* = 4.2 Hz), 7.29 (d, 1H, *J* = 7.2 Hz), 7.26–7.24 (m, 2H), 7.22 (dd, 1H, *J* = 7.2, 1.2 Hz), 2.29 (s, 3H); ^13^C{1H} NMR (150 MHz, CDCl_3_) δ 156.0 (C–F, ^2^*J*_C–F_ = 37.6 Hz), 139.9, 138.5, 136.4, 130.5, 129.6, 128.8, 128.7, 128.4, 128.1, 116.0 (C–F, ^1^*J*_C–F_ = 289.0 Hz), 18.4; IR (ATR) 3253, 1680 cm^–1^; HRMS (ESI-TOF) *m*/*z* calcd for C_15_H_11_F_3_NO 278.0798 (M–H)^−^, found 278.0795.

#### Methyl (3-Methyl-[1,1′-biphenyl]-2-yl)carbamate (**S5h**)


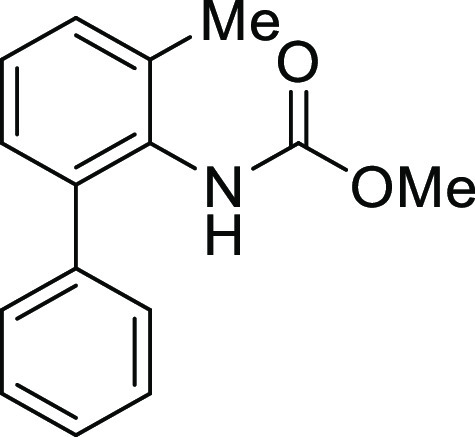
Compound **S5h** was prepared according to a similar procedure as described for the preparation of **S1a**. Colorless crystal (195.0 mg, 82%), mp 124–125 °C: ^1^H NMR (600 MHz, CDCl_3_) δ 7.43–7.40 (m, 2H), 7.36–7.33 (m, 2H), 7.32 (dd, 2H, *J* = 8.4, 1.2 Hz), 7.25–7.24 (m, 1H), 7.18–7.17 (m, 1H), 5.95 (br, 1H), 3.67 (s, 3H), 2.35 (s, 3H); ^13^C{1H} NMR (150 MHz, CDCl_3_) δ 139.8, 139.7, 136.8, 136.7, 132.5, 130.3, 129.0, 128.5, 128.2, 127.5, 127.3, 52.7, 18.6; IR (ATR) 3228, 1706 cm^–1^; HRMS (ESI-TOF) *m*/*z* calcd for C_15_H_16_NO_2_ 242.1176 (M+H)^+^, found 242.1176.

#### Methyl *N*-(Methoxycarbonyl)-*N*-(3-methyl-[1,1′-biphenyl]-2-yl)glycinate (**S6h**)


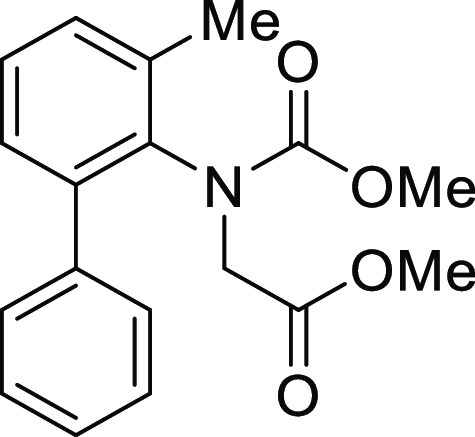
Compound **S6h** was prepared according to a similar procedure as described for the preparation of **S2a**. Colorless crystal (246.0 mg, 99%), mp 160–162 °C: ^1^H NMR (600 MHz, CDCl_3_) δ 7.42–7.39 (m, 2H), 7.37–7.34 (m, 1H), 7.31–7.25 (m, 2H), 7.24–7.23 (m, 2H), 7.18 (dd, 1H, *J* = 6.0, 2.4 Hz), 3.91 (d, 1H, *J* = 17.1 Hz), 3.79 (s, 3H), 3.62 (s, 3H), 3.24 (d, 1H, *J* = 17.1 Hz), 2.49 (s, 3H); ^13^C{1H} NMR (150 MHz, CDCl_3_) δ 169.7, 156.8, 139.7, 139.6, 138.4, 138.3, 130.5, 128.9, 128.5, 128.4, 128.0, 127.7, 53.6, 52.1, 52.0, 18.3; IR (ATR) 1755, 1705 cm^–1^; HRMS (ESI-TOF) *m*/*z* calcd for C_18_H_19_NO_4_Na 336.1206 (M+Na)^+^, found 336.1209.

#### *N*-([1,1′-Biphenyl]-2-yl)-4-methylbenzenesulfonamide (**S7c**)


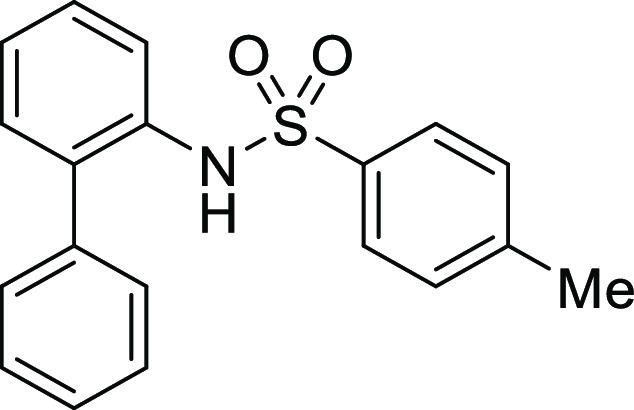
*p*-Tosyl choloride (270.0 mg, 1.41 mmol) was added to a solution of 2-aminobiphenyl (200.0 mg, 1.18 mmol) in pyridine (10.0 mL) at 0 °C. After stirring at rt for 23 h, the mixture was treated with 1 M HCl aq. and extracted with ethyl acetate. The extract was washed with 1 M HCl aq., 1 M NaHCO_3_ aq., and brine, dried, and concentrated. The concentrate was purified by column chromatography (silica gel, hexane/EtOAc = 4:1) to afford **S7c** as colorless crystals (243.1 mg, 64%), mp 92–93 °C: ^1^H NMR (600 MHz, CDCl_3_) δ 7.71(d, 1H, *J* = 8.4 Hz), 7.47 (d, 2H, *J* = 8.4 Hz), 7.38–7.31 (m, 4H), 7.19 (d, 2H, *J* = 8.4 Hz), 7.14 (ddd, 1H, *J* = 7.6, 7.6, 1.2 Hz), 7.10 (dd, 1H, *J* = 7.2, 1.8 Hz), 6.86 (dd, 2H, *J* = 7.2, 1.8 Hz), 6.58 (br, 1H), 2.40 (s, 3H); ^13^C{1H} NMR (150 MHz, CDCl_3_) δ 144.0, 137.4, 136.3, 134.0, 133.9, 130.4, 129.7, 129.2, 129.0, 128.8, 128.2, 127.3, 125.0, 121.5, 21.7; IR (ATR) 3245, 1328, 1158 cm^–1^; HRMS (ESI-TOF) *m*/*z* calcd for C_19_H_17_NO_2_SNa 346.0872 (M+Na)^+^, found 346.0872.

#### *N*-([1,1′-Biphenyl]-2-yl)methanesulfonamide (**S7d**)


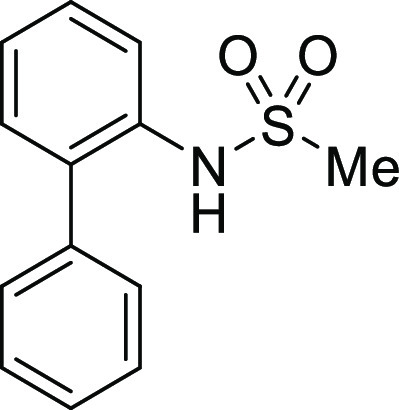
Compound **S7d** was prepared according to a similar procedure as described for the preparation of **S7c** from 2-aminobiphenyl. Colorless crystal (225.0 mg, 91%), mp 62–63 °C: ^1^H NMR (600 MHz, CDCl_3_) δ 7.67 (d, 1H, *J* = 7.8 Hz), 7.50 (dd, 2H, *J* = 7.2, 7.2 Hz), 7.44 (ddd, 1H, *J* = 7.8, 7.8, 1.8 Hz), 7.39 (ddd, 1H, *J* = 8.4, 8.4, 1.8 Hz), 7.33–7.32 (m, 2H), 7.28 (dd, 1H, *J* = 7.2, 1.8 Hz), 7.23 (ddd, 1H, *J* = 7.8, 7.8, 1.8 Hz), 6.49 (br, 1H), 2.87 (s, 3H); ^13^C{1H} NMR (150 MHz, CDCl_3_) δ 137.5, 134.1, 133.4, 130.9, 129.6, 129.1, 128.6, 125.0, 120.1, 39.8; IR (ATR) 3252, 1317, 1144 cm^–1^; HRMS (ESI-TOF) *m*/*z* calcd for C_13_H_12_NO_2_S 246.0594 (M–H)^−^, found 246.0600.

#### *N*-([1,1′-Biphenyl]-2-yl)-2-nitrobenzenesulfonamide (**S7e**)


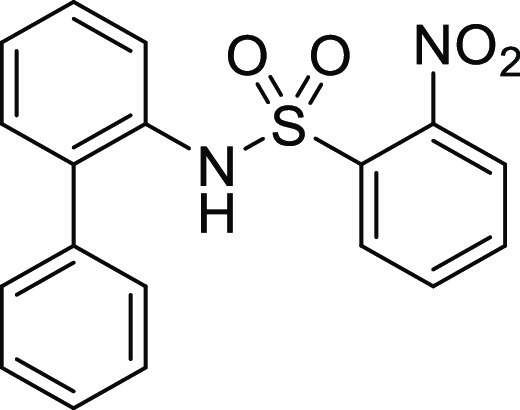
Compound **S7e** was prepared according to a similar procedure as described for the preparation of **S7c** from 2-aminobiphenyl. Colorless crystal (284.5 mg, 80%), mp 101–102 °C: ^1^H NMR (600 MHz, CDCl_3_) δ 7.75 (dd, 1H, *J* = 7.2, 1.2 Hz), 7.71 (d, 2H, *J* = 8.4 Hz), 7.67 (ddd, 1H, *J* = 7.8, 7.8, 1.2 Hz), 7.65 (br, 1H), 7.59 (ddd, 1H, *J* = 7.8, 7.8, 1.8 Hz), 7.40 (ddd, 1H, *J* = 7.8, 7.8, 1.2 Hz), 7.28–7.27 (m, 1H), 7.25–7.21 (m, 3H), 7.13 (dd, 1H, *J* = 7.8, 1.8 Hz), 6.86 (dd, 2H, *J* = 8.4, 1.8 Hz); ^13^C{1H} NMR (150 MHz, CDCl_3_) δ 147.6, 137.5, 136.4, 133.6, 133.4, 133.1, 132.7, 131.0, 130.4, 129.0, 128.9, 128.7, 128.1, 126.4, 125.8, 125.3; IR (ATR) 3326, 1533, 1391, 1359, 1176 cm^–1^; HRMS (ESI-TOF) *m*/*z* calcd for C_18_H_14_N_2_O_4_SNa 377.0566 (M+Na)^+^, found 377.0567.

#### *N*-([1,1′-Biphenyl]-2-yl)-4-nitrobenzenesulfonamide (**S7f**)


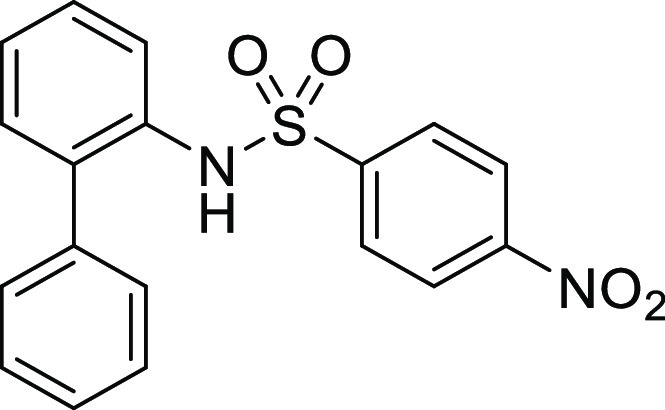
Compound **S7f** was prepared according to a similar procedure as described for the preparation of **S7c** from 2-aminobiphenyl. Colorless crystal (329.6 mg, 93%), mp 143–144 °C: ^1^H NMR (600 MHz, CDCl_3_) δ 8.17 (dt, 2H, *J* = 8.4, 1.8, Hz), 7.71 (dd, 1H, *J* = 7.8, 1.2 Hz), 7.64 (dt, 2H, *J* = 9.0, 1.8, Hz), 7.40–7.37 (m, 2H), 7.33 (dd, 2H, *J* = 7.2, 7.2 Hz), 7.24 (ddd, 1H, *J* = 7.8, 7.8, 1.2 Hz), 7,14 (dd, 1H, *J* = 7.8, 1.8 Hz), 6.82 (dd, 2H, *J* = 7.2, 1.8 Hz), 6.80 (br, 1H); ^13^C{1H} NMR (150 MHz, CDCl_3_) δ 150.3, 144.7, 137.1, 135.1, 132.5, 130.6, 129.4, 129.1, 128.7, 128.5, 126.4, 124.2, 123.1; IR (ATR) 3267, 1523, 1400, 1347, 1165 cm^–1^; HRMS (ESI-TOF) *m*/*z* calcd for C_18_H_13_N_2_O_4_S 353.0602 (M–H)^−^, found 353.0600.

#### Methyl *N*-([1,1′-Biphenyl]-2-yl)-*N*-tosylglycinate (**S8c**)


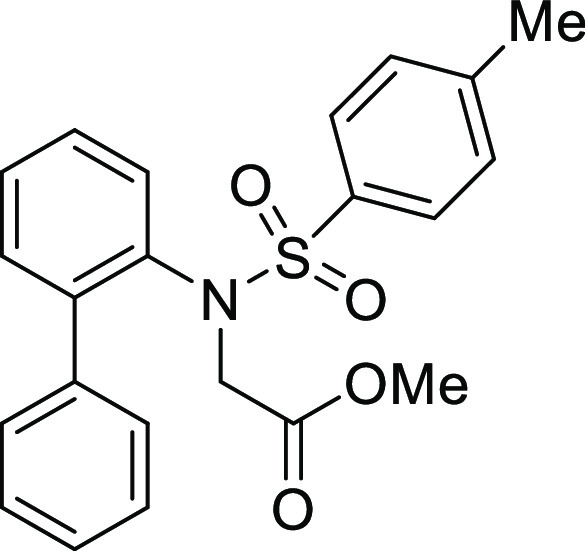
Sodium hydride (60% oil) (80.0 mg, 2.01 mmol) was added to a stirred solution of **S7c** (218.0 mg, 0.67 mmol) in DMF (5.0 mL) at 0 °C under an argon atmosphere. The mixture was stirred at rt for 20 min, then cooled to 0 °C and treated with methyl bromoacetate (93.0 μL, 1.00 mmol). After stirring at rt for 1 h, the mixture was treated with 1 M HCl aq. and extracted with ethyl acetate. The extract was washed with 1 M HCl aq., 1 M NaHCO_3_ aq., and brine, dried, and concentrated. The concentrate was purified by column chromatography (silica gel, hexane/EtOAc = 4:1) to afford **S8c** as colorless crystal (212.0 mg, 80%), mp 103–104 °C: ^1^H NMR (600 MHz, CDCl_3_) δ 7.66 (ddd, 2H, *J* = 8.4, 2.4, 1.8 Hz), 7.39–7.35 (m, 7H), 7.32 (dd, 1H, *J* = 7.8, 1.8 Hz), 7.29 (dd, 2H, *J* = 8.4, 1.8 Hz), 7.27–7.26 (m, 1H), 3.98 (br, 2H), 3.52 (s, 3H), 2.46 (s, 3H); ^13^C{1H} NMR (150 MHz, CDCl_3_) δ 169.5, 143.8, 141.4, 138.8, 138.0, 137.3, 131.8, 130.6, 129.5, 129.2, 128.8, 128.5, 128.2, 128.1, 127.8, 52.1, 52.1, 21.8; IR (ATR) 1740, 1332, 1155 cm^–1^; HRMS (ESI-TOF) *m*/*z* calcd for C_22_H_21_NO_4_SNa 418.1084 (M+Na)^+^, found 418.1098.

#### Methyl *N*-([1,1′-Biphenyl]-2-yl)-*N*-(methylsulfonyl)glycinate (**S8d**)


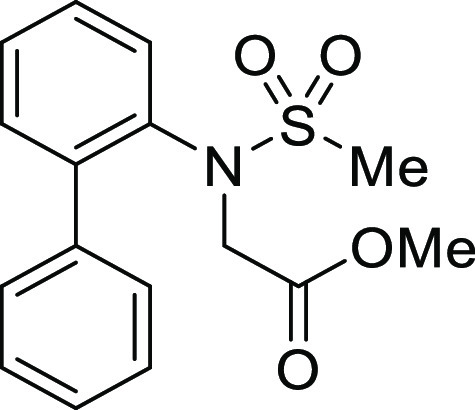
Compound **S8d** was prepared according to a similar procedure as described for the preparation of **S8c** from **S7c**. Colorless crystal (280.0 mg, 99%), mp 110–111 °C: ^1^H NMR (600 MHz, CDCl_3_) δ 7.66 (dd, 1H, *J* = 7.8, 1.2 Hz), 7.53 (d, 2H, *J* = 6.6 Hz), 7.45 (dd, 2H, *J* = 7.2, 7.2 Hz), 7.44–7.38 (m, 4H), 3.96 (br, 2H), 3.66 (s, 3H), 3.23 (s, 3H); ^13^C{1H} NMR (150 MHz, CDCl_3_) δ 170.4, 141.7, 138.7, 137.6, 132.0, 129.8, 129.2, 129.1, 128.6, 128.0, 52.4, 52.3, 42.7; IR (ATR) 1753, 1330, 1548 cm^–1^; HRMS (ESI-TOF) *m*/*z* calcd for C_16_H_17_NO_4_SNa 342.0771 (M+Na)^+^, found 342.0776.

#### Methyl *N*-([1,1′-Biphenyl]-2-yl)-*N*-([2-nitrophenyl]sulfonyl)glycinate (**S8e**)


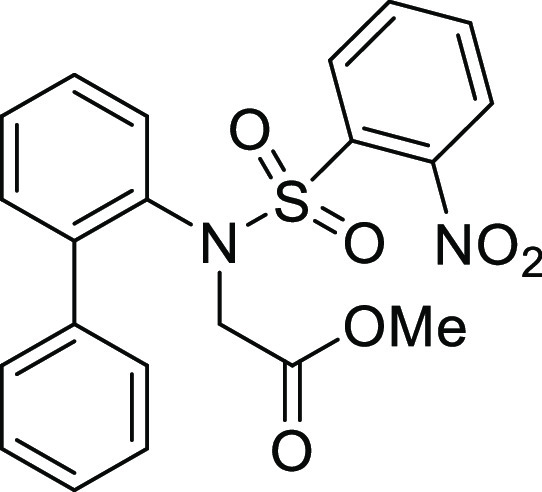
Compound **S8e** was prepared according to a similar procedure as described for the preparation of **S8c** from **S7c**. Colorless crystal (303.0 mg, 96%), mp 122–123 °C: ^1^H NMR (600 MHz, CDCl_3_) δ 7.90 (dd, 1H, *J* = 7.8, 1.8 Hz), 7.78 (dd, 1H, *J* = 7.8, 1.2 Hz), 7.69 (ddd, 1H, *J* = 7.2, 7.2, 1.2 Hz), 7.65 (ddd, 1H, *J* = 7.8, 7.8, 1.2 Hz), 7.56 (dd, 1H, *J* = 7.8, 1.2 Hz), 7.43 (ddd, 1H, *J* = 7.2, 7.2, 1.8 Hz), 7.39 (ddd, 1H, *J* = 7.2, 7.2, 1.8 Hz), 7.31–7.27 (m, 4H), 7.18–7.17 (m, 2H), 4.20 (br, 2H), 3,63 (s, 3H); ^13^C{1H} NMR (150 MHz, CDCl_3_) δ 169.7, 148.1, 141.9, 138.4, 136.9, 134.3, 133.7, 131.9, 131.9, 131.8, 131.7, 129.4, 128.8, 128.5, 128.4, 127.7, 124.5, 53.3, 52.3; IR (ATR) 1755, 1547, 1373, 1361, 1170 cm^–1^; HRMS (ESI-TOF) *m*/*z* calcd for C_21_H_18_N_2_O_6_SNa 499.0778 (M+Na)^+^, found 499.0785.

#### Methyl *N*-([1,1′-Biphenyl]-2-yl)-*N*-([4-nitrophenyl]sulfonyl)glycinate (**S8f**)


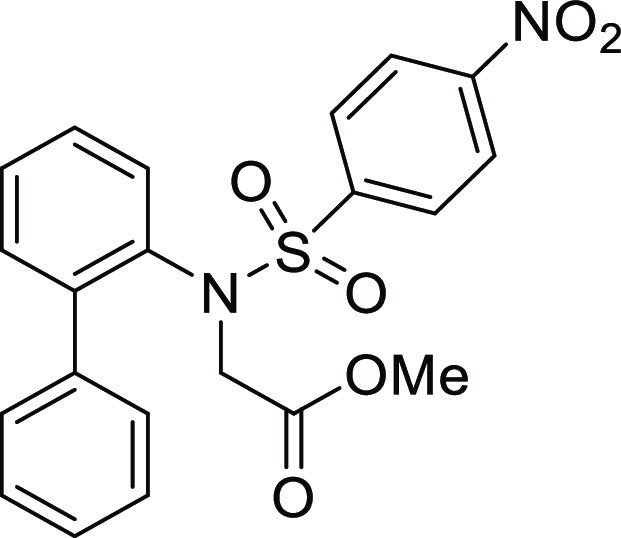
Compound **S8f** was prepared according to a similar procedure as described for the preparation of **S8c** from **S7c**. Colorless crystal (334.0 mg, 91%), mp 149–150 °C: ^1^H NMR (600 MHz, CDCl_3_) δ 8.30 (dt, 2H, *J* = 9.0, 2.4 Hz), 7.92 (dt, 2H, *J* = 9.0, 1.8 Hz), 7.44–7.39 (m, 6H), 7.36 (d, 1H, *J* = 7.2 Hz), 7.30 (dd, 2H, *J* = 4.8, 1.8 Hz), 4.13 (br, 2H), 3.59 (s, 3H); ^13^C{1H} NMR (150 MHz, CDCl_3_) δ 169.3, 150.2, 146.4, 141.8, 138.4, 136.6, 132.2, 130.4, 129.6, 129.0, 128.6, 128.5, 128.0, 124.0, 52.9, 52.4; IR (ATR) 1760, 1525, 1341, 1313, 1163 cm^–1^; HRMS (ESI-TOF) *m*/*z* calcd for C_21_H_19_N_2_O_6_S 427.0958 (M+H)^+^, found 427.0973.

#### 4-Methyl-*N*-(3-methyl-[1,1′-biphenyl]-2-yl)benzenesulfonamide (**S9c**)


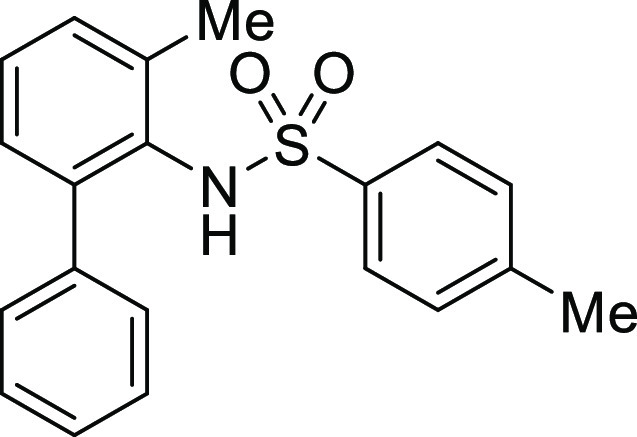
Compound **S9c** was prepared according to a similar procedure as described for the preparation of **S7c** from 2-aminobiphenyl. Colorless crystal (303.9 mg, 90%), mp 114–116 °C: ^1^H NMR (600 MHz, CDCl_3_) δ 7.29 (d, 1H, *J* = 7.8 Hz), 7.22–7.18 (m, 2H), 7.14 (dd, 2H, *J* = 7.2, 7.2 Hz), 7.09 (d, 2H, *J* = 7.8 Hz), 6.97 (d, 3H, *J* = 7.8 Hz), 6.78 (dd, 2H, *J* = 7.2, 1.2 Hz), 6.52 (br, 1H), 2.56 (s, 3H), 2.38 (s, 3H); ^13^C{1H} NMR (150 MHz, CDCl_3_) δ 143.0, 140.6, 139.1, 139.0, 136.7, 131.2, 131.1, 129.5, 128.5, 128.4, 127.6, 127.0, 21.6, 20.1; IR (ATR) 3294, 1331, 1162 cm^–1^; HRMS (ESI-TOF) *m*/*z* calcd for C_20_H_19_NO_2_SNa 360.1029 (M+Na)^+^, found 360.1029.

#### *N*-(3-Methyl-[1,1′-biphenyl]-2-yl)methanesulfonamide (**S9d**)


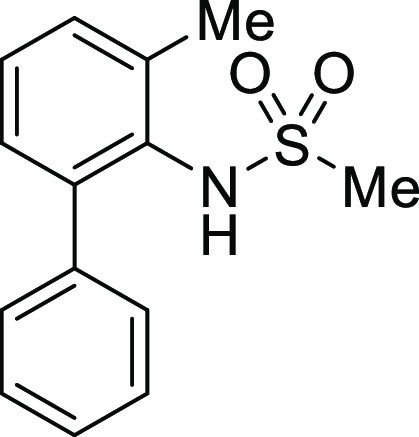
Compound **S9d** was prepared according to a similar procedure as described for the preparation of **S7c** from 2-aminobiphenyl. Colorless crystal (216.5 mg, 83%), mp 95–96 °C: ^1^H NMR (600 MHz, CDCl_3_) δ 7.47–7.44 (m, 2H), 7.41–7.36 (m, 3H), 7.32–7.29 (m, 2H), 7.22–7.19 (m, 1H), 6.05 (br, 1H), 2.53 (s, 3H), 2.21 (s, 3H); ^13^C{1H} NMR (150 MHz, CDCl_3_) δ 141.0, 139.9, 139.0, 131.7, 130.9, 129.7, 128.8, 128.7, 128.3, 127.8, 41.2, 19.8; IR (ATR) 3244, 1314, 1145 cm^–1^; HRMS (ESI-TOF) *m*/*z* calcd for C_14_H_15_NO_2_SNa 284.0716 (M+Na)^+^, found 284.0719.

#### *N*-(3-Methyl-[1,1′-biphenyl]-2-yl)-2-nitrobenzenesulfonamide (**S9e**)


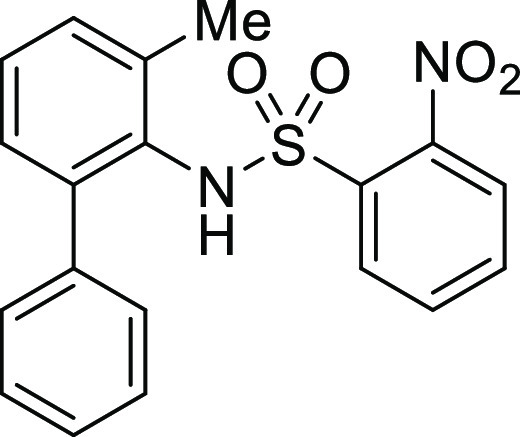
Compound **S9e** was prepared according to a similar procedure as described for the preparation of **S7c** from 2-aminobiphenyl. Colorless crystal (206.6 mg, 47%), mp 135–137 °C: ^1^H NMR (600 MHz, CDCl_3_) δ 7.66 (dd, 1H, *J* = 8.4, 1.2 Hz), 7.59–7.54 (m, 3H), 7.51 (ddd, 1H, *J* = 7.8, 7.8, 1.2 Hz), 7.34 (d, 1H, 7.2 Hz), 7.29–7.26 (m, 1H), 7.03–6.97 (m, 3H), 6.91 (dd, 2H, 7.8, 2.4 Hz), 2.61 (s, 3H); ^13^C{1H} NMR (150 MHz, CDCl_3_) δ 146.7, 140.9, 140.0, 138.9, 135.2, 133.1, 132.9, 131.5, 130.9, 130.6, 128.8, 128.5, 128.3, 128.2, 127.1, 126.1, 20.0; IR (ATR) 3386, 1532, 1385, 1328, 1162 cm^–1^; HRMS (ESI-TOF) *m*/*z* calcd for C_29_H_16_N_2_O_4_SNa 391.0723 (M+Na)^+^, found 391.0723.

#### *N*-(3-Methyl-[1,1′-biphenyl]-2-yl)-4-nitrobenzenesulfonamide (**S9f**)


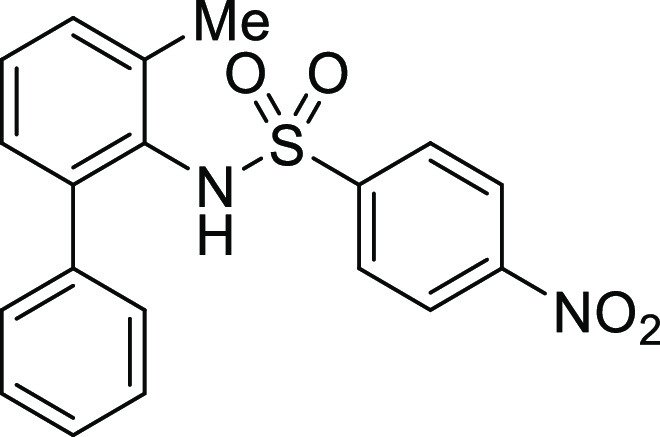
Compound **S9f** was prepared according to a similar procedure as described for the preparation of **S7c** from 2-aminobiphenyl. Colorless crystal (315.7 mg, 86%), mp 148–151 °C: ^1^H NMR (600 MHz, CDCl_3_) δ 7.95 (dd, 2H, *J* = 8.4, 1.2 Hz), 7.38 (dd, 2H, *J* = 8.4, 1.8 Hz), 7.34 (d, 1H, *J* = 8.4 Hz), 7.28 (dd, 1H, *J* = 8.4, 8.4 Hz), 7.18 (dd, 1H, *J* = 7.8, 7.8 Hz), 7.09 (dd, 2H, *J* = 7.2, 7.2 Hz), 7.00 (d, 1H, *J* = 7.8 Hz), 6.80 (d, 2H, *J* = 8.4 Hz), 6.67 (br, 1H), 2.62 (s, 3H); ^13^C{1H} NMR (150 MHz, CDCl_3_) δ 149.8, 143.3, 140.7, 139.8, 138.9, 131.3, 130.0, 128.9, 128.7, 128.6, 128.5, 128.1, 127.3, 124.0, 20.1; IR (ATR) 3259, 1525, 1351, 1310, 1154 cm^–1^; HRMS (ESI-TOF) *m*/*z* calcd for C_19_H_15_N_2_O_4_S 367.0758 (M–H)^−^, found 367.0750.

#### Methyl *N*-(3-Methyl-[1,1′-biphenyl]-2-yl)-*N*-tosylglycinate (**S10c**)


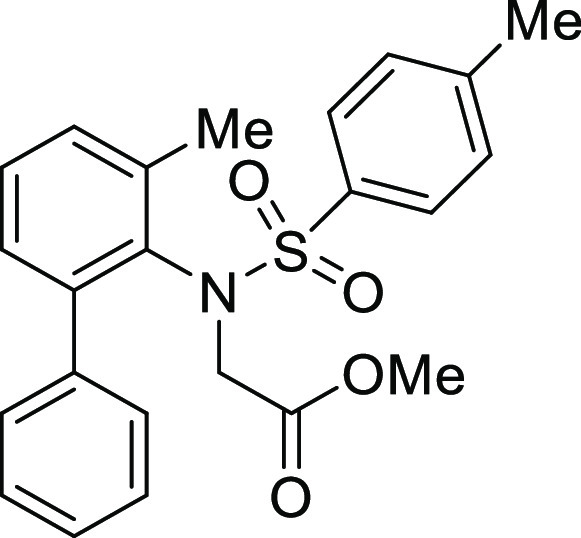
Compound **S10c** was prepared according to a similar procedure as described for the preparation of **S8c** from **S7c**. Colorless crystal (234.1 mg, 66%), mp 118–120 °C: ^1^H NMR (600 MHz, CDCl_3_) δ 7.57 (d, 2H, *J* = 8.4 Hz), 7.33–7.30 (m, 1H), 7.28–7.25 (m, 6H), 7.21 (d, 2H, *J* = 8.2 Hz), 7.08–7.06 (m, 1H), 4.17 (d, 1H, *J* = 17.2 Hz), 3.81 (d, 1H, *J* = 17.2 Hz), 3.54 (s, 3H), 2.43 (s, 3H), 2.37 (s, 3H); ^13^C{1H} NMR (150 MHz, CDCl_3_) δ 169.2, 143.6, 142.9, 140.2, 139.9, 137.5, 137.4, 131.2, 129.9, 129.6, 129.4, 128.3, 128.2, 127.9, 127.5, 53.2, 52.1, 21.7, 20.3; IR (ATR) 1766, 1335, 1158 cm^–1^; HRMS (ESI-TOF) *m*/*z* calcd for C_23_H_24_NO_4_S 410.1421 (M+H)^+^, found 410.1424.

#### Methyl *N*-(3-Methyl-[1,1′-biphenyl]-2-yl)-*N*-(methylsulfonyl)glycinate (**S10d**)


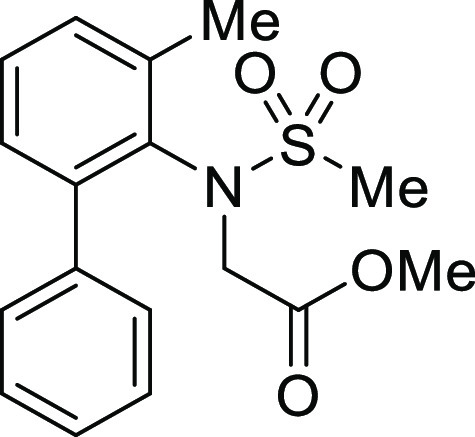
Compound **S10d** was prepared according to a similar procedure as described for the preparation of **S8c** from **S7c**. Colorless crystal (243.4 mg, 91%), mp 108–109 °C: ^1^H NMR (600 MHz, CDCl_3_) δ 7.44–7.35 (m, 5H), 7.30–7.27 (m, 2H), 7.15–7.13 (m, 1H), 4.31 (d, 1H, *J* = 18.0 Hz), 3.97 (d, 1H, *J* = 18.0 Hz), 3.68 (s, 3H), 2.80 (s, 3H), 2.55 (s, 3H); ^13^C{1H} NMR (150 MHz, CDCl_3_) δ 169.6, 142.7, 140.0, 139.2, 138.6, 131.3, 129.8, 129.6, 128.5, 128.2, 127.8, 53.5, 52.3, 42.4, 19.9; IR (ATR) 1756, 1327, 1139 cm^–1^; HRMS (ESI-TOF) *m*/*z* calcd for C_17_H_19_NO_4_SNa 356.0927 (M+Na)^+^, found 356.0927.

#### Methyl *N*-(3-Methyl-[1,1′-biphenyl]-2-yl)-*N*-([2-nitrophenyl]sulfonyl)glycinate (**S10e**)


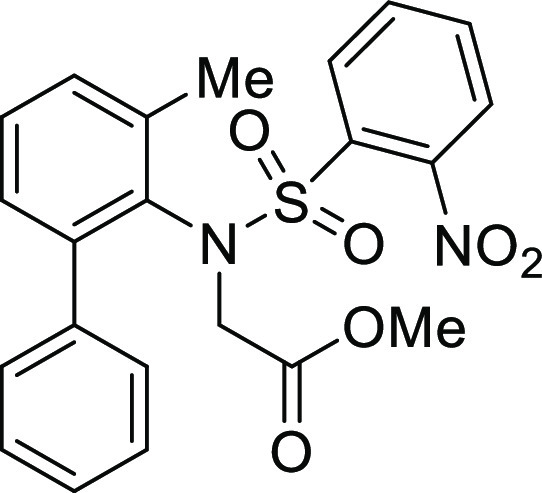
Compound **S10e** was prepared according to a similar procedure as described for the preparation of **S8c** from **S7c**. Colorless crystal (332.0 mg, 84%), mp 138–139 °C: ^1^H NMR (600 MHz, CDCl_3_) δ 7.73 (dd, 1H, *J* = 9.0, 1.8 Hz), 7.65 (ddd, 1H, *J* = 7.6, 7.6, 1.2 Hz), 7.54–7.51 (m, 2H), 7.34–7.27 (m, 3H), 7.23 (dd, 2H, *J* = 7.6, 7.6 Hz), 7.17 (d, 2H, *J* = 6.6 Hz), 7.07–7.04 (m, 1H), 4.48 (d, 1H, *J* = 18.0 Hz), 4.08 (d, 1H, *J* = 18.0 Hz), 3.64 (s, 3H), 2.45 (s, 3H); ^13^C{1H} NMR (150 MHz, CDCl_3_) δ 169.1, 148.5, 143.4, 140.2, 139.7, 136.5, 134.0, 133.7, 132.0, 131.6, 130.2, 129.5, 128.8, 128.0, 127.6, 124.2, 54.4, 52.3, 20.3; IR (ATR) 1771, 1548, 1374, 1346, 1199 cm^–1^; HRMS (ESI-TOF) *m*/*z* calcd for C_22_H_20_N_2_O_6_SNa 463.0934 (M+Na)^+^, found 463.0935.

#### Methyl *N*-(3-Methyl-[1,1′-biphenyl]-2-yl)-*N*-([4-nitrophenyl)sulfonyl)glycinate (**S10f**)


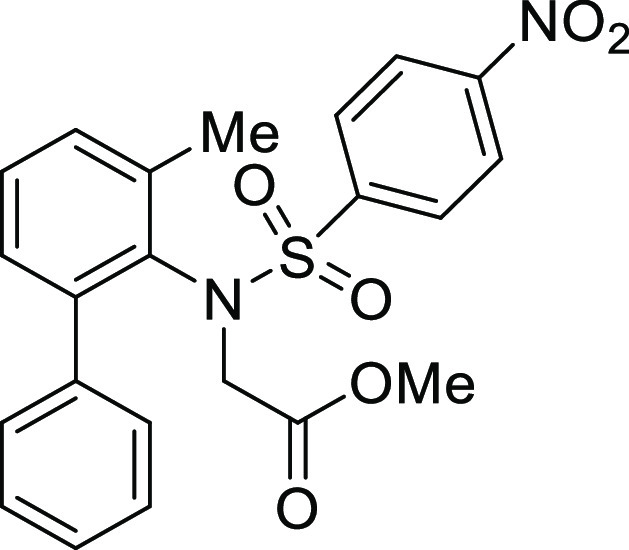
Compound **S10f** was prepared according to a similar procedure as described for the preparation of **S8c** from **S7c**. Colorless crystal (324.4 mg, 87%), mp 153–156 °C: ^1^H NMR (600 MHz, CDCl_3_) δ 8.16 (dd, 2H, *J* = 8.4, 2.4 Hz), 7.80 (dd, 2H, *J* = 9.0, 1.8 Hz), 7.32–7.27 (m, 3H), 7.24 (dd, 2H, *J* = 7.2, 7.2 Hz), 7.19 (dd, 2H, *J* = 6.6, 1.8 Hz), 7.09 (dd, 1H, *J* = 7.2, 1.8 Hz), 4.27 (d, 1H, *J* = 17.4 Hz), 4.22 (d, 1H, *J* = 17.4 Hz), 3.65 (s, 3H), 2.36 (s, 3H); ^13^C{1H} NMR (150 MHz, CDCl_3_) δ 168.8, 150.0, 145.9, 143.1, 139.8, 139.5, 136.9, 131.5, 130.2, 129.5, 129.0, 128.0, 127.6, 123.7, 54.3, 52.4, 20.0; IR (ATR) 1759, 1524, 1349, 1314, 1158 cm^–1^; HRMS (ESI-TOF) *m*/*z* calcd for C_22_H_20_N_2_O_6_SNa 463.0934 (M+Na)^+^, found 463.0934.

### Measurement of the Blocking Activity on the Voltage-Gated Potassium Channel Kv1.3

The assays were performed under the conditions described below. The parameters measured the maximum outward current evoked on stepping to 0 mV from the holding potential. The peak current amplitude was calculated before and after compound addition, and the amount of block was assessed by dividing the test compound current amplitude by the control current amplitude. Test compounds are the mean hKv1.3 current amplitude collected in the presence of test compound at each concentration, and the control is the mean hKv1.3 current amplitude collected for the last 15 s of the control. All data were filtered for seal quality, seal drop, and current amplitude.

## Supporting Information Available

Reaction schemes to prepare **1A**–**h** and **1Bc**–**h**; ^1^H NMR spectra of **IBg** and **IBh**; Chiral HPLC charts of **IBg**, **IBh**, **IIBc**, **IIBd**, **IIBe**, **IIBf**; Stereochemical stability of the enantiomers of **IBg**, **IBh**, **IIBc**, **IIBd**, **IIBe**, **IIBf**; NOE spectrum of **IAa**; ORTEP drawing of **IIBc**; DFT calculation study; ^1^H-, ^13^C-, and 2D-NMR (COSY, HMBC for **IAa**) spectra (PDF)

## Accession Codes

CCDC 2057747 contains the supplementary crystallographic data for this paper. These data can be obtained free of charge via www.ccdc.cam.ac.uk/data_request/cif, or by emailing data_request@ccdc.cam.ac.uk, or by contacting The Cambridge Crystallographic Data Centre, 12 Union Road, Cambridge CB2 1EZ, UK; fax: +44 1223 336033.
