# Supplementary material for: Atropisomeric Properties of N-Acyl/N-Sulfonyl 5H-Dibenzo[b,d]azepin-7(6H)-ones
Source: J Org Chem. 2021 May 17;86(11):7563–78. doi: 10.1021/acs.joc.1c00594 (PMC8279494; doi:10.1021/acs.joc.1c00594)

Supporting Information

Atropisomeric Properties of *N*-acyl/*N*-sulfonyl 5*H*-dibenzo[*b*,*d*]azepin-7(6*H*)-ones

Takuya Namba,[1] Mayuno Hotta,[1] Hidetsugu Tabata,[2] Kosho Makino,[1] Tetsuta Oshitari,[2] Hideaki Natsugari,[3] and Hideyo Takahashi*[1]

[1]Faculty of Pharmaceutical Sciences, Tokyo University of Science, 2641Yamazaki, Noda-shi, Chiba 278-8510, Japan

[2]Faculty of Pharma Sciences, Teikyo University, 2-11-1 Kaga, Itabashi-ku, Tokyo 173-8605, Japan

[3]Graduate School of Pharmaceutical Science, The University of Tokyo, 7-3-1 Hongo, Bunkyo-ku, Tokyo 113-0033, Japan

hide-tak@rs.tus.ac.jp

Contents

1. **Reaction schemes to prepare 1A~h and 1Bc~h……………………………………….S2**
2. **^{1}H NMR spectra of IBg and IBh………………………………………………………..S4**
3. **Chiral HPLC charts of IBg, IBh, IIBc, IIBd, IIBe, IIBf…………………………….S5**
4. **Stereochemical stability of the enantiomers of IBg, IBh, IIBc, IIBd, IIBe, IIBf…...S8**
5. **NOE spectrum of IAa……………………………………………………………….S14**
6. **ORTEP drawing of IIBc……………………………………………………………....S15**
7. **DFT calculation study……………………………………………………………….S16**
8. **^{1}H-, ^{13}C-, and 2D-NMR Spectra ………………….………….……………………S42**

1. Reaction schemes to prepare 1A~h and 1Bc~h

Scheme S1. Preparation of **1Aa**, **1Ab**, **1Ag**, **1Ah**

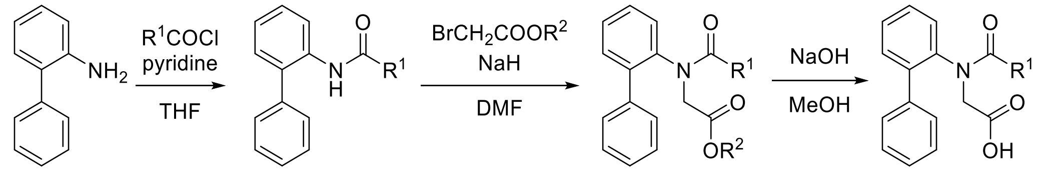

2-aminobiphenyl **S1** **S2** **1A**

S1a: R^1 = Me
S1b: R^1 = *p*-$CH_3C_6H_4$
S1h: R^1 = OMe

S2a: R^1 = Me, R^2 = Me
S2b: R^1 = *p*-$CH_3C_6H_4$, R^2 = Me
S2h: R^1 = OMe, R^2 = Me

1Aa: R^1 = Me
1Ab: R^1 = *p*-$CH_3C_6H_4$
1Ah: R^1 = OMe

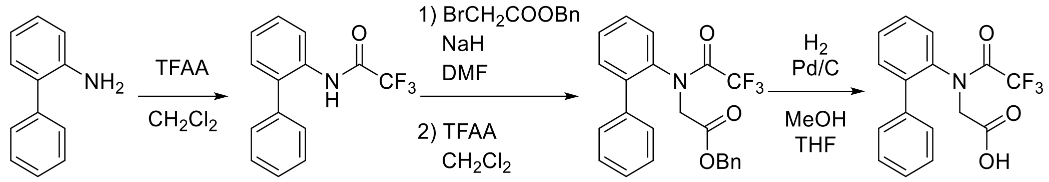

2-aminobiphenyl **S1g** **S2g** **1Ag**

Scheme S2. Preparation of **1Bg**, **1Bh**

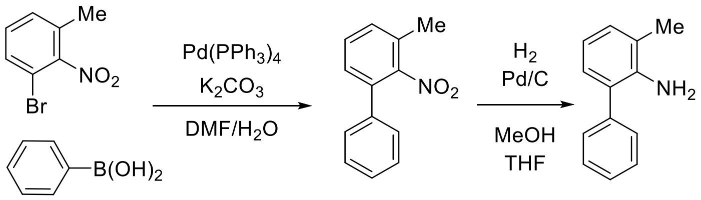

S3 **S4**

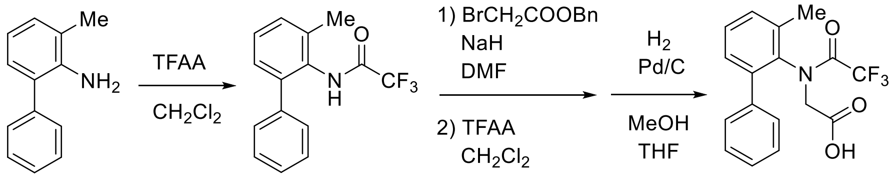

S4 **S5g** **1Bg**

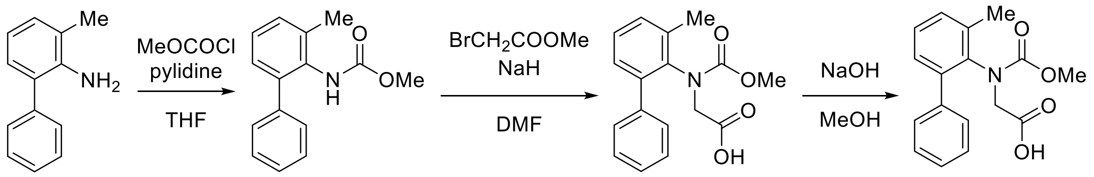

S4 **S5h** **S6h** **1Bh**

Scheme S3. Preparation of **1Ae**, **1Ad**, **1Ae**, **1Af**

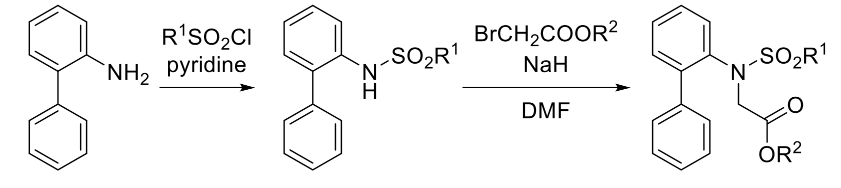

2-aminobiphenyl | **S7** | **S8**

S7c: R^1 = *p*-$CH_3C_6H_4$
S7d: R^1 = Me
S7e: R^1 = *o*-$NO_2C_6H_4$
S7f: R^1 = *p*-$NO_2C_6H_4$

S8c: R^1 = *p*-$CH_3C_6H_4$, R^2 = Me
S8d: R^1 =Me, R^2 = Me
S8e: R^1 = *o*-$NO_2C_6H_4$, R^2 = Me
S8f: R^1 = *p*-$NO_2C_6H_4$, R^2 = Me

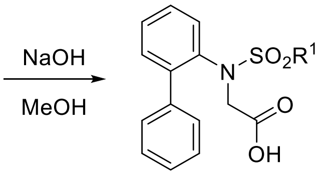

1A

1Ac: R^1 = *p*-$CH_3C_6H_4$
1Ad: R^1 = Me
1Ae: R^1 = *o*-$NO_2C_6H_4$
1Af: R^1 = *p*-$NO_2C_6H_4$

Scheme S4. Preparation of **1Bc**, **1Bd**, **1Be**, **1Bf**

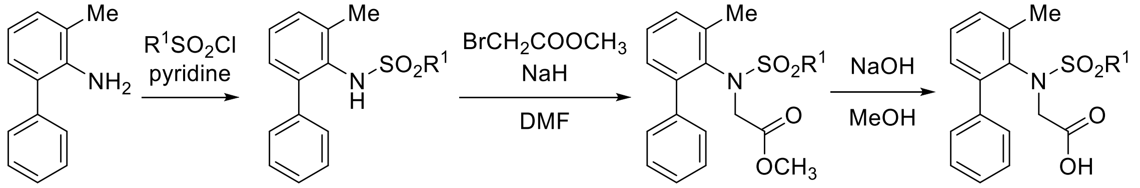

S4 | **S9** | **S10** | **1B**

c: R^1 = *p*-$CH_3C_6H_4$
d: R^1 = Me
e: R^1 = *o*-$NO_2C_6H_4$
f: R^1 = *p*-$NO_2C_6H_4$

2. ^{1}H NMR spectra of IBg (a) and IBh (b)

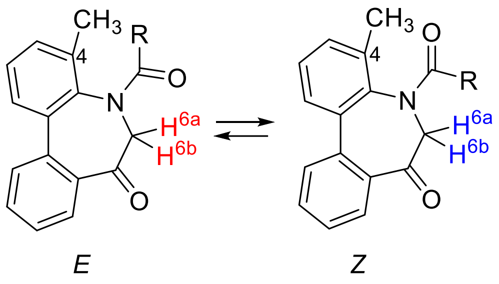

IBg: R = CF_3
IBh: R = OMe

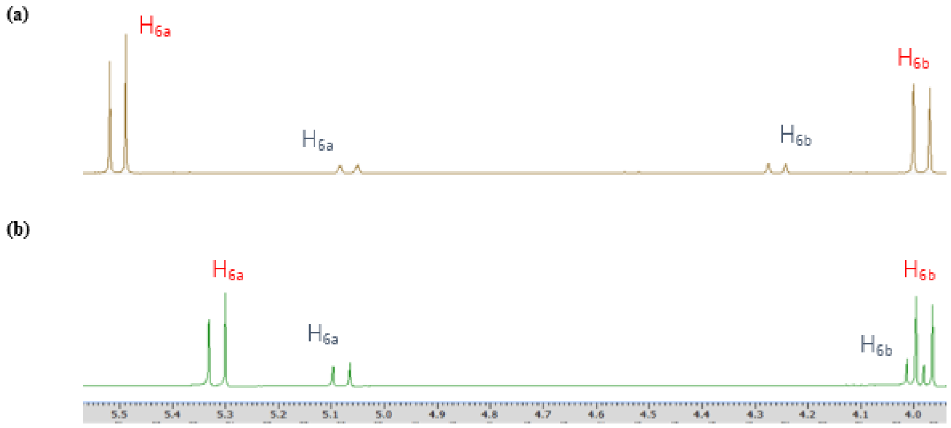

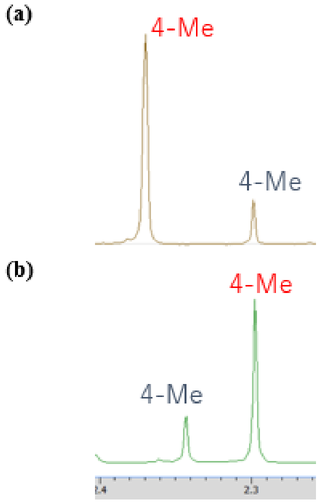

3. Chiral HPLC charts of IBg, IBh, IIBc, IIBd, IIBe, IIBf

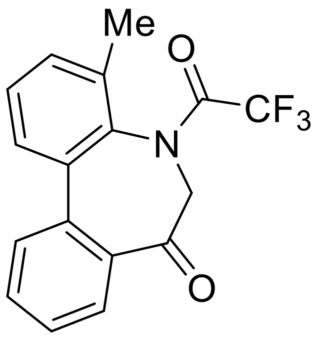

IBg

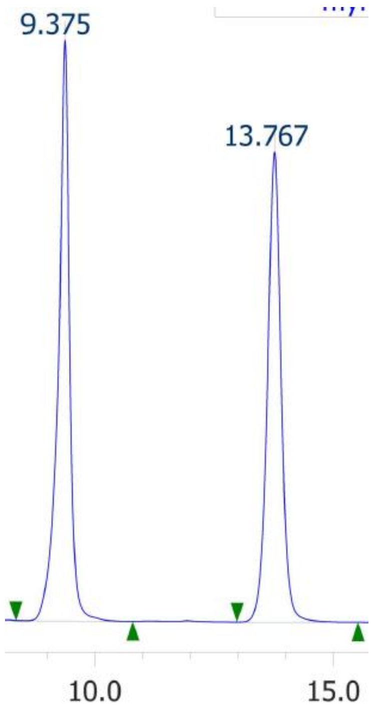

HPLC conditions

Column : CHIRALPAK IA

Eluent : 30%IPA/Hex

Flow rate : 0.5 mL/min

Temp. : 25 ° C

Rt. : 9.4 min, 13.8 min

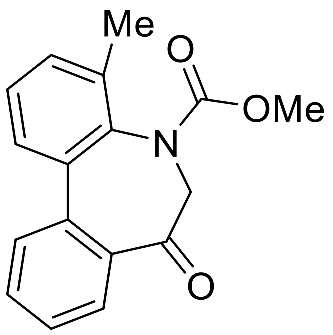

IBh

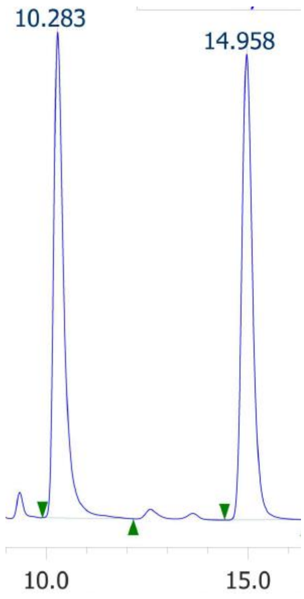

HPLC conditions

Column : CHIRALPAK IA

Eluent : 30%IPA/Hex

Flow rate : 0.5 mL/min

Temp. : 25 ° C

Rt. : 10.3 min, 15.0 min

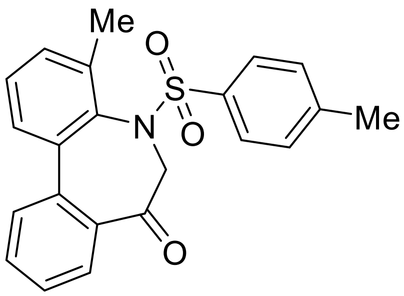

IIBc

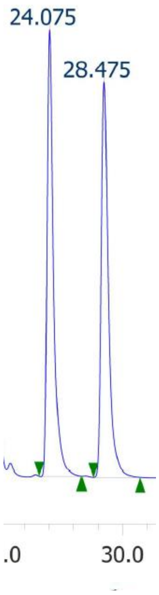

HPLC conditions

Column : CHIRALPAK IB

Eluent : 30%IPA/Hex

Flow rate : 0.3 mL/min

Temp. : 25 ° C

Rt. : 24.1 min, 28.5 min

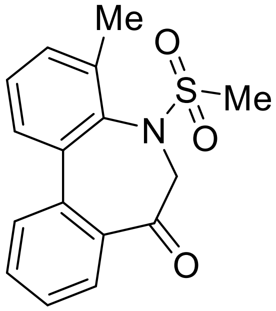

IIBd

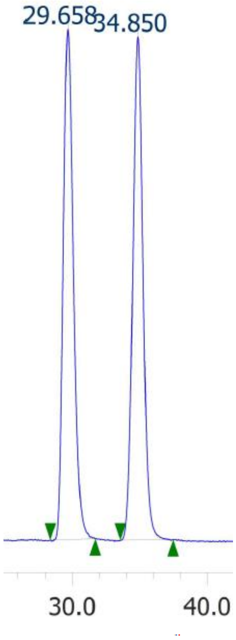

HPLC conditions

Column : CHIRALPAK IA

Eluent : 30%IPA/Hex

Flow rate : 0.5 mL/min

Temp. : 25 ° C

Rt. : 30.0 min, 34.9 min

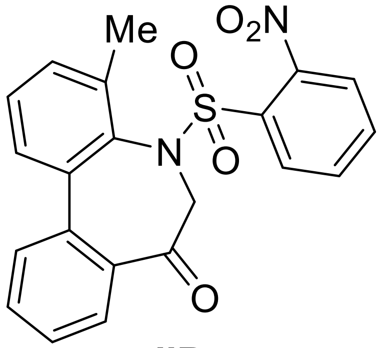

IIBe

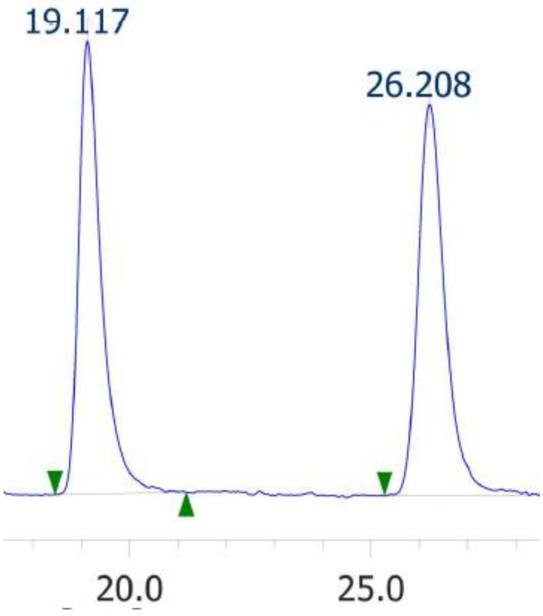

HPLC conditions
Column : CHIRALPAK IA
Eluent : 30%IPA/Hex
Flow rate : 0.5 mL/min
Temp. : 25 ° C
Rt. : 19.1 min, 26.2 min

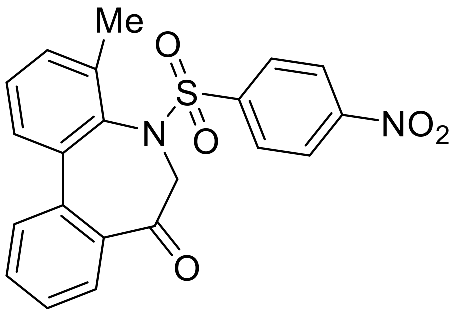

IIBf

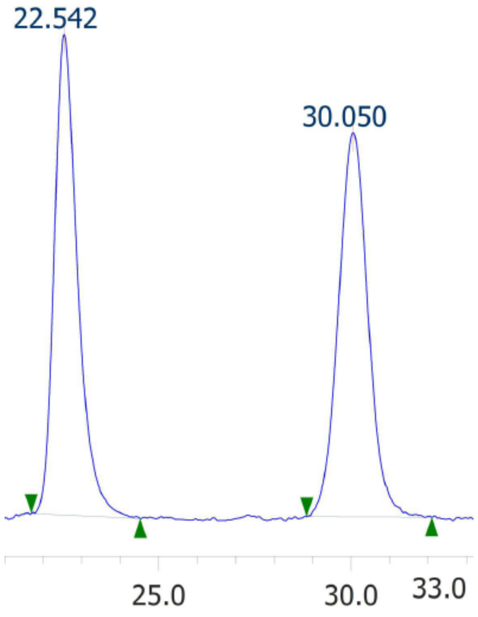

HPLC conditions
Column : CHIRALPAK IA
Eluent : 30%IPA/Hex
Flow rate : 0.5 mL/min
Temp. : 25 ° C
Rt. : 22.5 min, 30.1 min

4. Stereochemical stability of the enantiomers of IBg, IBh, IIBc, IIBd, IIBe, IIBf

The conversion profile of the enantiomer of IBg:

In toluene at 80 ℃ (0 to 26 hours) analyzed with HPLC using a chiral column (CHIRALPAK IA).

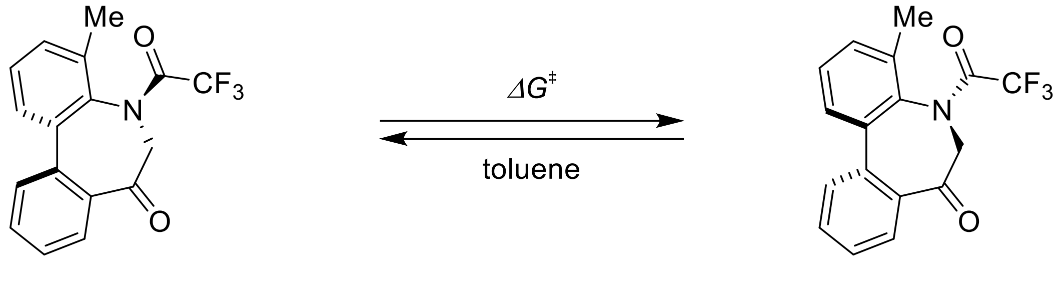

(a*R*, a*R*)-**IBg** (a*S*, a*S*)-**IBg**

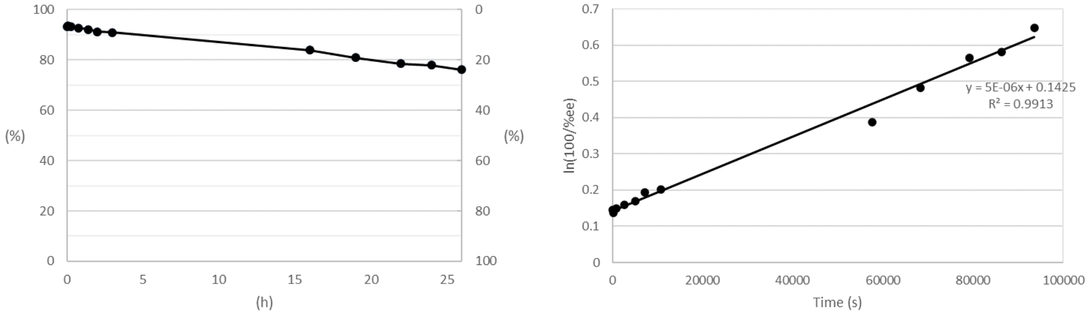

Figure S1. Conversion profile of the enantiomer of **IIBg**

T = 353

k = 1/2 slope = $2.5x10^{-6}$

K = kh/*k*T = $3.398x10^{-19}$

$\Delta G^{\ddagger}$ = -RTlnK = 124.8 kJ/mol

The conversion profile of the enantiomer of IBh:

In toluene at 80 ℃ (0 to 6 hours) analyzed with HPLC using a chiral column (CHIRALPAK IA).

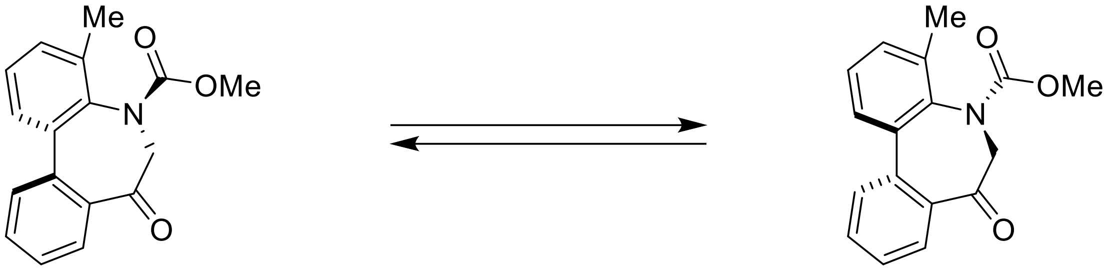

(a*R*, a*R*)-**IBh** (a*S*, a*S*)-**IBh**

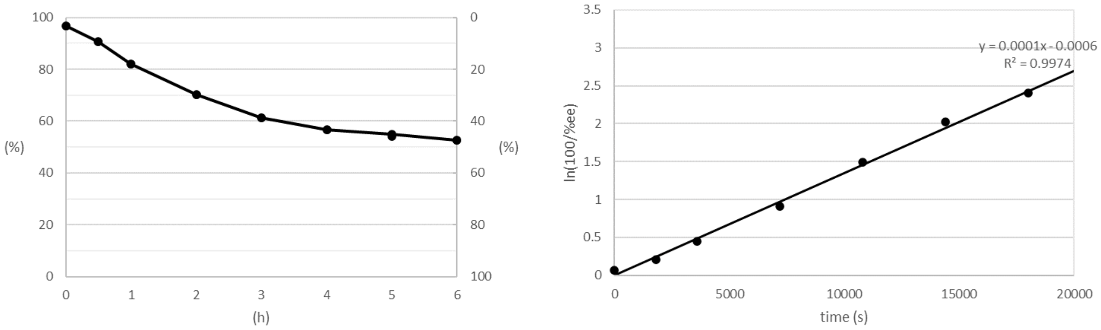

Figure S2. Conversion profile of the enantiomer of **IIBh**

T = 353

k = 1/2 slope = 5.0x10^{-5}

K = kh/*k*T = 6.796x10^{-18}

$\Delta G^{\ddagger}$ = -RTlnK = 116.0 kJ/mol

Conversion profile of the enantiomer of IIBc:

In toluene at 80 ℃ (0 to 54 hours) analyzed with HPLC using a chiral column (CHIRALPAK IB).

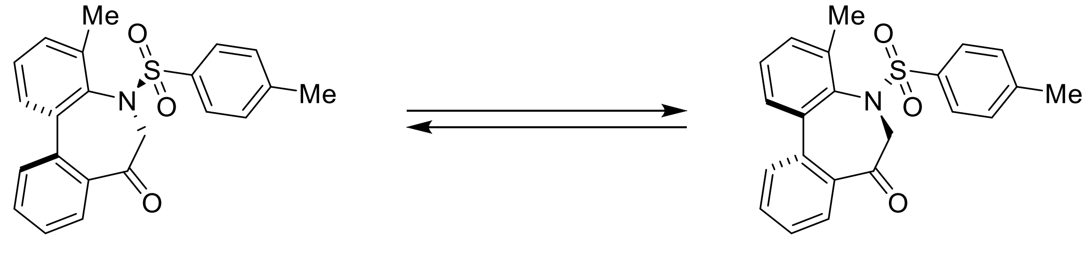

(a*R*, a*R*)-**IIBc** (a*S*, a*S*)-**IIBc**

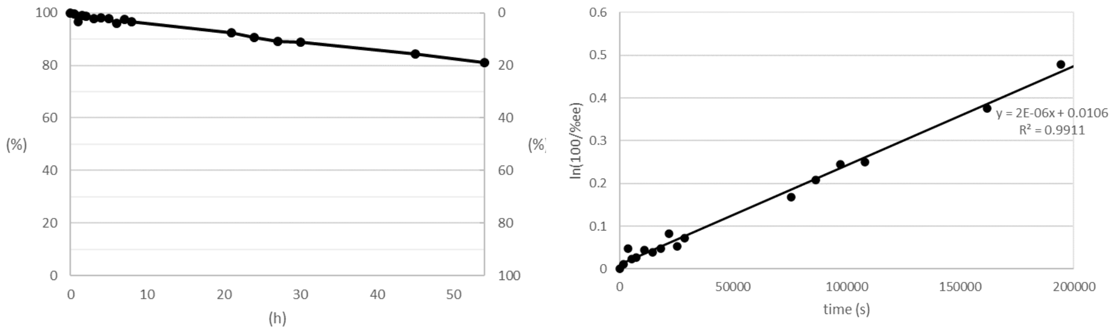

Figure S3. Conversion profile of the enantiomer of **IIBc**

T = 353

k = 1/2 slope = 1.0×10^{-6}

K = kh/*k*T = 1.359×10^{-19}

$\Delta G^{\ddagger}$ = -RTlnK = 127.5 kJ/mol

Conversion profile of the enantiomer of IIBd:

In toluene at 80 ℃ (0 to 51 hours) analyzed with HPLC using a chiral column (CHIRALPAK IA).

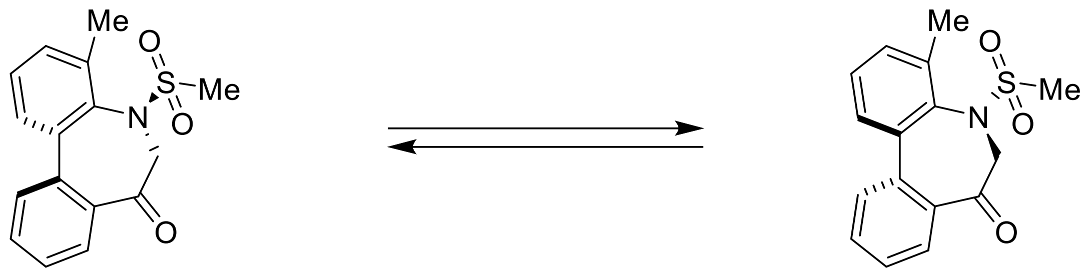

(a*R*, a*R*)-**IIBd** (a*S*, a*S*)-**IIBd**

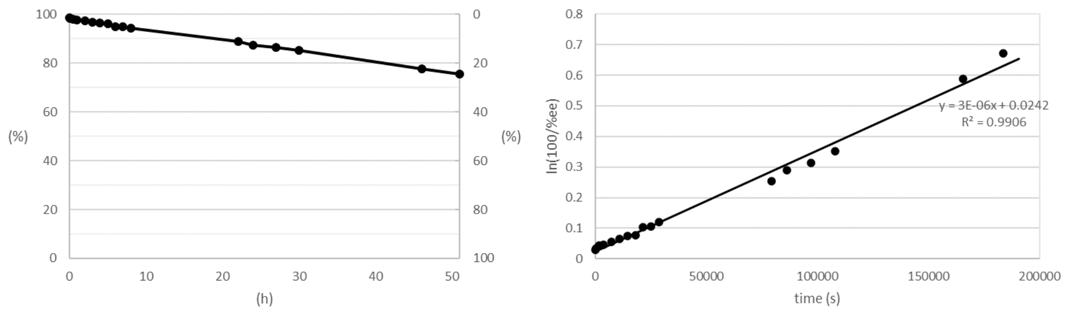

Figure S4. Conversion profile of the enantiomer of **IIBd**

T = 353

k = 1/2 slope = $1.5\text{x}10^{-6}$

K = kh/*k*T = $2.039\text{x}10^{-19}$

$\Delta G^{\ddagger}$ = -RTlnK = 126.3 kJ/mol

Conversion profile of the enantiomer of IIBe:

In toluene at 80 ℃ (0 to 54 hours) analyzed with HPLC using a chiral column (CHIRALPAK IA).

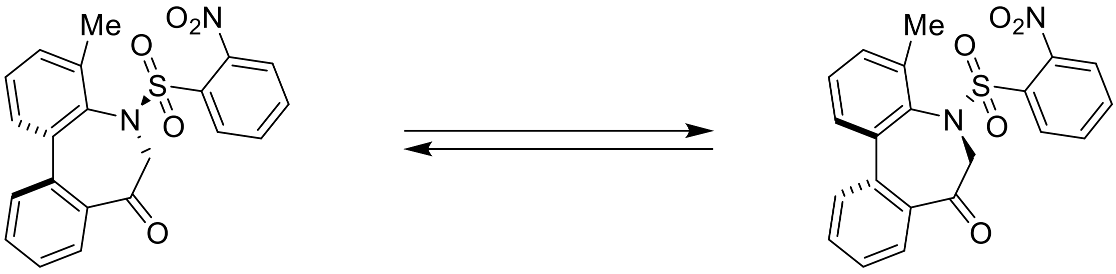

(a*R*, a*R*)-**IIBe** (a*S*, a*S*)-**IIBe**

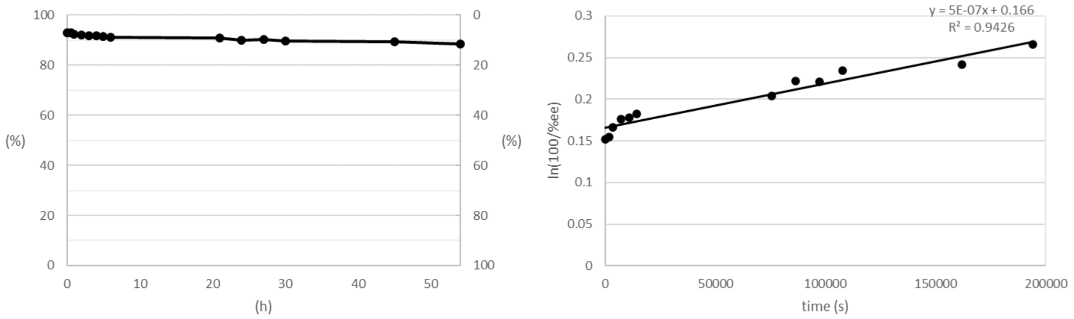

Figure S5. Conversion profile of the enantiomer of **IIBe**

T = 353

k = 1/2 slope = $2.5x10^{-7}$

K = kh/*k*T = $3.398x10^{-20}$

$\Delta G^{\ddagger}$ = -RTlnK = 131.6 kJ/mol

Conversion profile of the enantiomer of IIBf:

In toluene at 80 ℃ (0 to 51 hours) analyzed with HPLC using a chiral column (CHIRALPAK IA).

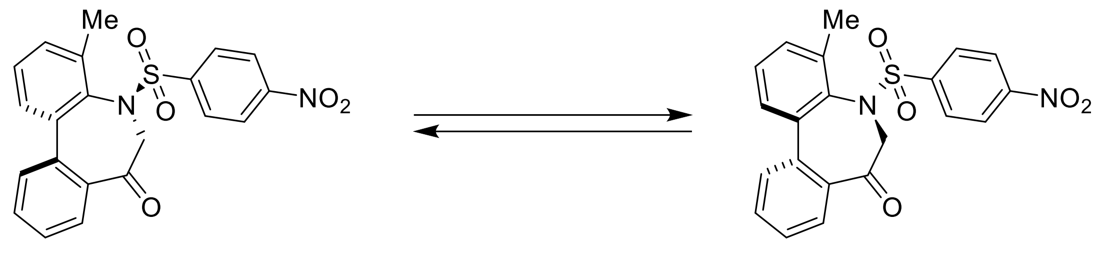

(a*R*, a*R*)-**IIBf** (a*S*, a*S*)-**IIBf**

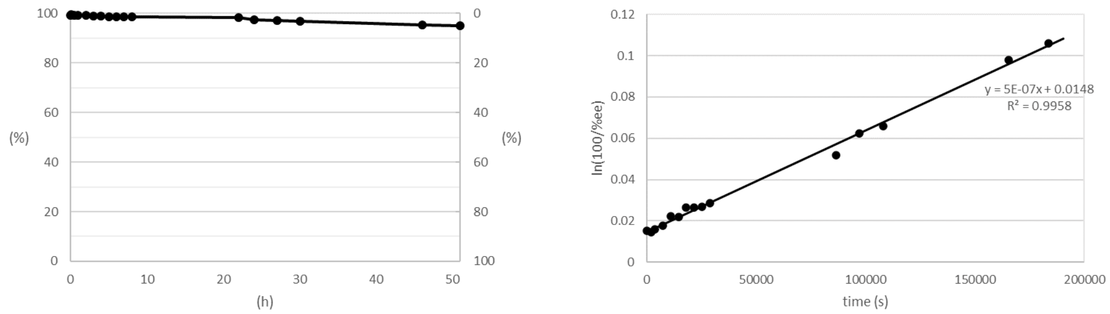

Figure S6. Conversion profile of the enantiomer of **IIBf**

T = 353

k = 1/2 slope = 2.5×10^{-7}

K = kh/*k*T = 3.398×10^{-20}

$\Delta G^{\ddagger}$ = -RTlnK = 131.6 kJ/mol

5. NOE spectrum of IA

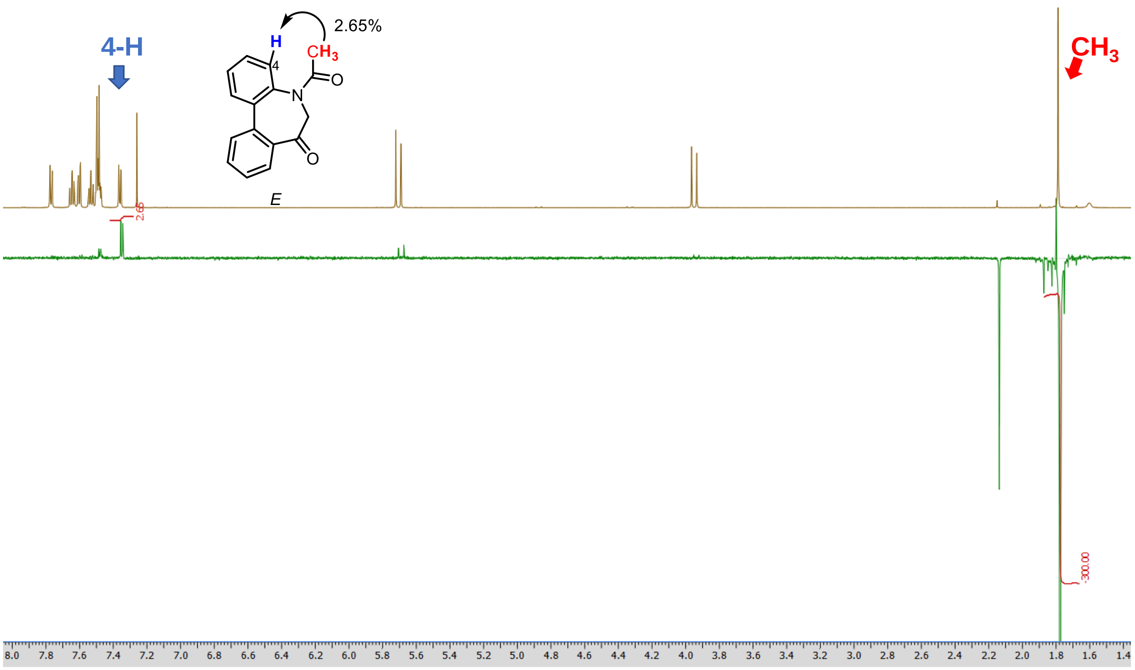

NOE spectrum (600 MHz, $CDCl_3$) of **IAa**

6. **ORTEP drawings of IIBc**

All measurements were made on a Rigaku Raxis Rapid imaging plate area detector with graphite monochromated Cu-Kα radiation. The data were collected at a temperature of –100 °C. The structure was solved by direct method SIR97 and expanded using Fourier techniques. The non-hydrogen atoms were refined anisotropically. All calculations were performed using the Crystal Structure (Crystal Structure 4.2.2) crystallographic software package except for refinement, which was performed using SHELXL97. Typical crystal data and ORTEP diagrams are as follows.

Crystal of **IIBc** was obtained by dissolving the compounds in dichloromethane/hexane and allowing the solvent to slowly evaporate at room temperature.

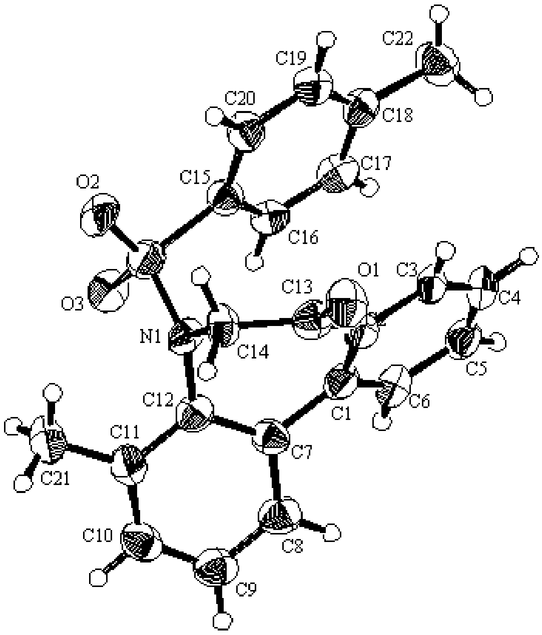

Ellipsoid contour probability level = 50 %

Crystal data of **IIBc** (CCDC 2057747)

$C_{22}H_{19}NO_3S$: mp 48–51 °C, Mr = 377.46, CuKα (λ = 1.54187 Å), orthorhombic, Pbca, colorless prism 0.200 × 0.200 × 0.040 mm, crystal dimensions a = 11.2275(2) Å, b = 17.5275(3) Å, c = 18.5389(3) Å, α = 90°, β = 90°, γ = 90°, T = 173 K, Z = 8, V = 3648.24(12) Å^3, D_{calc} = 1.374 g/cm^3, μ CuKα = 17.647 cm^{-1}, F_{000} = 1584.00, GOF = 1.547, R_{int} = 0.0847, R_1 = 0.0815, wR_2 = 0.1885.

7. DFT calculation study

Computational Studies

DFT calculations were performed with Gaussian 09 (**note 1**).

Note 1: Full reference

Gaussian 09, Revision D.01, M. J. Frisch, G. W. Trucks, H. B. Schlegel, G. E. Scuseria, M. A. Robb, J. R. Cheeseman, G. Scalmani, V. Barone, G. A. Petersson, H. Nakatsuji, X. Li, M. Caricato, A. Marenich, J. Bloino, B. G. Janesko, R. Gomperts, B. Mennucci, H. P. Hratchian, J. V. Ortiz, A. F. Izmaylov, J. L. Sonnenberg, D. Williams-Young, F. Ding, F. Lipparini, F. Egidi, J. Goings, B. Peng, A. Petrone, T. Henderson, D. Ranasinghe, V. G. Zakrzewski, J. Gao, N. Rega, G. Zheng, W. Liang, M. Hada, M. Ehara, K. Toyota, R. Fukuda, J. Hasegawa, M. Ishida, T. Nakajima, Y. Honda, O. Kitao, H. Nakai, T. Vreven, K. Throssell, J. A. Montgomery, Jr., J. E. Peralta, F. Ogliaro, M. Bearpark, J. J. Heyd, E. Brothers, K. N. Kudin, V. N. Staroverov, T. Keith, R. Kobayashi, J. Normand, K. Raghavachari, A. Rendell, J. C. Burant, S. S. Iyengar, J. Tomasi, M. Cossi, J. M. Millam, M. Klene, C. Adamo, R. Cammi, J. W. Ochterski, R. L. Martin, K. Morokuma, O. Farkas, J. B. Foresman, and D. J. Fox, Gaussian, Inc., Wallingford CT, 2016.

Energy differences between the conformers determined by DFT calculation.

Conformer generations were performed for **IAa, IAg, IAh, IBg, IBh**. Tables S1–S5 show the energy differences of the conformers in comparison with the lowest-energy conformer determined by HF/DFT calculation,s1 i.e., E values (SCF energy) determined using the RHF/6-31G(d) and the RB3LYP/6-31G(d) levels, and E+G values (Sum of electronic and thermal Free Energies) determined using the RmPW1PW91/6-311+G(d,p) level. Since the a*R*,a*R* and a*S*,a*S* conformers are energetically equivalent, they are not described specifically. The right column of the tables shows the geometry type of the conformers determined using the RmPW1PW91/6-311+G(d,p) level, and the following abbreviations are used: *E* (*E*-amide) and *Z* (*Z*-amide). Optimized geometries were verified by frequency calculations at the RmPW1PW91/6-311+G(d,p) level as minima (zero imaginary frequencies). *E*/*Z* ratios were calculated by Sum of electronic and thermal Free Energies.

Table S1 Compound **IAa**

Conformer number	*E* (hartree) HF/ 6-31(d)	B3LYP/ 6-31(d)	*E*+*G* (hartree) mPW1Pw91/6-311+G(d,p)	Geometry type of conformers
1	-817.6684827	-822.7491063	-822.560026	*E*
2	-817.6635846	-822.7450493	-822.556118	*Z*

Table S2 Compound **IAg**

Conformer number	*E* (hartree) HF/ 6-31(d)	B3LYP/ 6-31(d)	*E*+*G* (hartree) mPW1Pw91/ 6-311+G(d,p)	Geometry type of conformers
1	-1114.232699	-1120.452759	-1120.319884	*E*
2	-1114.232699	-1120.452759		*E*
3	-1114.232699	-1120.452759		*E*
4	-1114.232699	-1120.452759		*E*
5	-1114.232699	-1120.452759		*E*
6	-1114.232699	-1120.452759		*E*
7	-1114.235114	-1120.455057	-1120.32263	*Z*
8	-1114.235114	-1120.455057		*Z*
9	-1114.235114	-1120.455057		*Z*
10	-1114.235114	-1120.455057		*Z*
11	-1114.235114	-1120.455057		*Z*
12	-1114.235114	-1120.455057		*Z*
13	-1114.235114	-1120.455057		*Z*
14	-1114.235114	-1120.455057		*Z*
15	-1114.235114	-1120.455057		*Z*

Table S3 Compound **IAh**

Conformer number	*E* (hartree) HF/ 6-31(d)	B3LYP/ 6-31(d)	*E*+*G* (hartree) mPW1Pw91/ 6-311+G(d,p)	Geometry type of conformers
1	-892.5372787	-897.9730511	-897.783329	*E*
2	-892.5355535	-897.9714905	-897.782256	*Z*

Table S4 Compound **IBg**

Conformer number	*E* (hartree) HF/ 6-31(d)	B3LYP/ 6-31(d)	*E*+*G* (hartree) mPW1Pw91/ 6-311+G(d,p)	Geometry type of conformers
1	-1153.270788	-1159.772326	-1159.614235	*E*
2	-1153.270788	-1159.772326		*E*
3	-1153.270788	-1159.772326		*E*
4	-1153.270788	-1159.772326		*E*
5	-1153.270788	-1159.772326		*E*
6	-1153.270788	-1159.772326		*E*
7	-1153.27015	-1159.771544	-1159.612913	*Z*
8	-1153.27015	-1159.771544		*Z*
9	-1153.27015	-1159.771544		*Z*
10	-1153.27015	-1159.771544		*Z*
11	-1153.27015	-1159.771544		*Z*
12	-1153.27015	-1159.771544		*Z*

Table S5 Compound **IBh**

Conformer number	*E* (hartree) HF/6-31(d)	B3LYP/6-31(d)	*E*+*G* (hartree) mPW1Pw91/6-311+G(d,p)	Geometry type of conformers
1	-931.5744039	-937.2912451	-937.076261	*E*
2	-931.5731189	-937.2902687	-937.075124	*Z*

Atomic coordinates for the lowest-energy conformers by DFT calculation.

IAa

E-amide

Zero-point correction=	0.254151 (Hartree/Particle)
Thermal correction to Energy=	0.269297
Thermal correction to Enthalpy=	0.270242
Thermal correction to Gibbs Free Energy=	0.211373
Sum of electronic and zero-point Energies=	-822.517249
Sum of electronic and thermal Energies=	-822.502102
Sum of electronic and thermal Enthalpies=	-822.501158
Sum of electronic and thermal Free Energies=	-822.560026

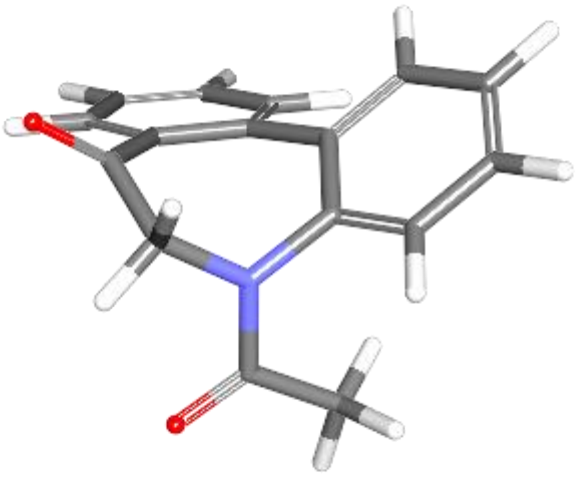

Center Number	Atomic Number	Atomic Type	Coordinates (Angstroms) X	Y	Z
1	6	0	2.367913	-2.672414	-0.610135
2	6	0	1.019570	-2.445432	-0.374272
3	6	0	0.528865	-1.151341	-0.171092
4	6	0	1.438089	-0.087467	-0.228860
5	6	0	2.787865	-0.312309	-0.474895
6	6	0	3.258350	-1.605656	-0.654780
7	7	0	0.945903	1.239447	-0.089472
8	6	0	0.185477	1.742516	-1.208897
9	6	0	-1.174844	1.097765	-1.406891
10	8	0	-1.863107	1.470267	-2.333557
11	6	0	-1.683671	0.091991	-0.428249
12	6	0	-0.902010	-0.931974	0.143211
13	6	0	-1.529146	-1.826769	1.017029
14	6	0	-2.876086	-1.716256	1.328069
15	6	0	-3.643410	-0.711573	0.750211
16	6	0	-3.046795	0.173970	-0.131488
17	6	0	1.099080	2.008385	1.036838
18	8	0	0.655646	3.145398	1.092405
19	6	0	1.843238	1.386343	2.188727
20	1	0	2.721987	-3.683958	-0.768547
21	1	0	0.330270	-3.281135	-0.359853
22	1	0	3.461466	0.534797	-0.527528
23	1	0	4.310898	-1.777357	-0.844217
24	1	0	0.752112	1.640662	-2.138583
25	1	0	0.005048	2.806397	-1.041100
26	1	0	-0.940611	-2.613380	1.473595
27	1	0	-3.324818	-2.415221	2.023887
28	1	0	-4.696764	-0.619470	0.984862
29	1	0	-3.628335	0.956123	-0.603490
30	1	0	1.571547	0.341895	2.341993
31	1	0	2.919301	1.426981	2.005222
32	1	0	1.623021	1.964252	3.083270

Z-amide

Zero-point correction=	0.254302 (Hartree/Particle)
Thermal correction to Energy=	0.269382
Thermal correction to Enthalpy=	0.270326
Thermal correction to Gibbs Free Energy=	0.211612
Sum of electronic and zero-point Energies=	-822.513428
Sum of electronic and thermal Energies=	-822.498348
Sum of electronic and thermal Enthalpies=	-822.497404
Sum of electronic and thermal Free Energies=	-822.556118

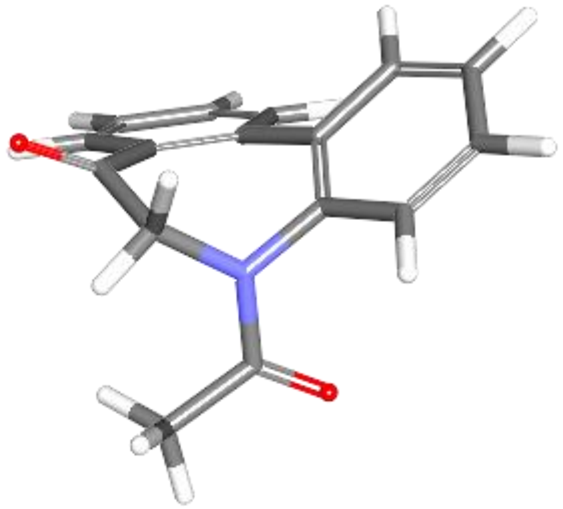

Center Number	Atomic Number	Atomic Type	Coordinates (Angstroms) X	Y	Z
1	6	0	-2.280622	-2.848334	0.583602
2	6	0	-0.940250	-2.548417	0.394560
3	6	0	-0.514882	-1.230046	0.189983
4	6	0	-1.482751	-0.220794	0.213251
5	6	0	-2.827755	-0.518417	0.391710
6	6	0	-3.231588	-1.833474	0.569151
7	7	0	-1.057290	1.134635	0.116492
8	6	0	-0.327455	1.611667	1.268497
9	6	0	1.119032	1.150104	1.367733
10	8	0	1.831648	1.678889	2.195997
11	6	0	1.652759	0.124227	0.428894
12	6	0	0.909164	-0.947621	-0.109793
13	6	0	1.574461	-1.837786	-0.961507
14	6	0	2.915443	-1.685446	-1.277315
15	6	0	3.644805	-0.635572	-0.730857
16	6	0	3.012201	0.250887	0.122045
17	6	0	-1.248227	1.839500	-1.045483
18	8	0	-1.825237	1.340609	-1.997521
19	6	0	-0.722217	3.252497	-1.086135
20	1	0	-2.583105	-3.876049	0.745111
21	1	0	-0.205802	-3.344508	0.415815
22	1	0	-3.548401	0.289929	0.391508
23	1	0	-4.280873	-2.063377	0.708871
24	1	0	-0.313553	2.700203	1.298891
25	1	0	-0.839901	1.286121	2.179270
26	1	0	1.016328	-2.654534	-1.402097
27	1	0	3.388462	-2.385774	-1.955515
28	1	0	4.693400	-0.508671	-0.970338
29	1	0	3.560029	1.071650	0.567965
30	1	0	0.350492	3.291426	-0.887223
31	1	0	-1.225482	3.884957	-0.351066
32	1	0	-0.913274	3.649621	-2.079766

IAg

E-amide

Zero-point correction= 0.231032 (Hartree/Particle)

Thermal correction to Energy= 0.248124

Thermal correction to Enthalpy= 0.249069

Thermal correction to Gibbs Free Energy= 0.184853

Sum of electronic and zero-point Energies= -1120.276451

Sum of electronic and thermal Energies= -1120.259359

Sum of electronic and thermal Enthalpies= -1120.258415

Sum of electronic and thermal Free Energies= -1120.322630

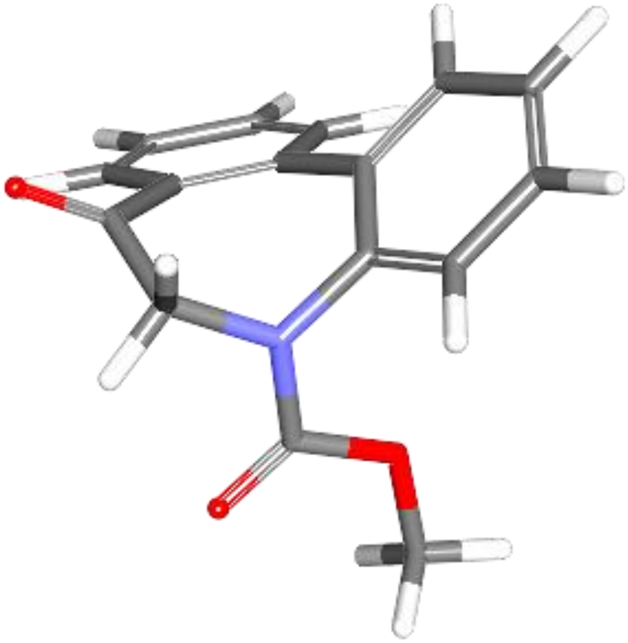

Center Number	Atomic Number	Atomic Type	Coordinates (Angstroms) X	Y	Z
1	6	0	1.285904	3.448397	0.413790
2	6	0	0.111091	2.791138	0.079573
3	6	0	0.027035	1.394949	0.121778
4	6	0	1.158898	0.682571	0.532504
5	6	0	2.337153	1.337823	0.868266
6	6	0	2.406957	2.721825	0.800644
7	7	0	1.062301	-0.730750	0.666579
8	6	0	0.258685	-1.211456	1.763426
9	6	0	-1.238768	-1.003609	1.609809
10	8	0	-1.971299	-1.414468	2.484992
11	6	0	-1.792047	-0.383804	0.370186
12	6	0	-1.211051	0.705398	-0.311097
13	6	0	-1.871163	1.197627	-1.442479
14	6	0	-3.055579	0.637535	-1.896522
15	6	0	-3.628954	-0.427871	-1.211993
16	6	0	-3.001879	-0.920277	-0.080287
17	6	0	1.547833	-1.617923	-0.251653
18	8	0	1.429124	-2.822736	-0.168550
19	8	0	2.176420	-0.990856	-1.252967
20	6	0	2.724520	-1.841898	-2.261755
21	1	0	1.324212	4.530542	0.377963
22	1	0	-0.761357	3.365789	-0.207423
23	1	0	3.192856	0.751638	1.180394
24	1	0	3.326713	3.230980	1.061783
25	1	0	0.575097	-0.740378	2.698053
26	1	0	0.416134	-2.286523	1.864521
27	1	0	-1.431762	2.025059	-1.986195
28	1	0	-3.528539	1.032903	-2.787675
29	1	0	-4.555192	-0.870383	-1.557571
30	1	0	-3.436391	-1.741445	0.476156
31	1	0	3.188997	-1.173524	-2.981991
32	1	0	1.937967	-2.425215	-2.739757
33	1	0	3.468383	-2.514180	-1.834889

Z-amide

Zero-point correction= 0.231299 (Hartree/Particle)

Thermal correction to Energy= 0.248326

Thermal correction to Enthalpy= 0.249270

Thermal correction to Gibbs Free Energy= 0.185338

Sum of electronic and zero-point Energies= -1120.273923

Sum of electronic and thermal Energies= -1120.256897

Sum of electronic and thermal Enthalpies= -1120.255952

Sum of electronic and thermal Free Energies= -1120.319884

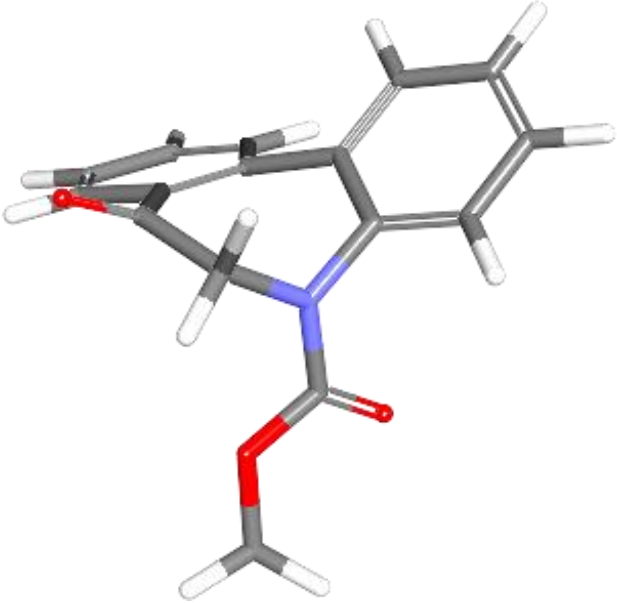

Center Number	Atomic Number	Atomic Type	Coordinates (Angstroms) X	Y	Z
1	6	0	0.103613	-3.842538	0.293362
2	6	0	0.970324	-2.777747	0.098063
3	6	0	0.506227	-1.457024	0.098960
4	6	0	-0.855830	-1.244656	0.336174
5	6	0	-1.728766	-2.308683	0.523306
6	6	0	-1.253226	-3.611601	0.492871
7	7	0	-1.324058	0.093244	0.451092
8	6	0	-0.875416	0.809784	1.623450
9	6	0	0.568159	1.282627	1.585791
10	8	0	0.953066	2.020955	2.468769
11	6	0	1.463916	0.896809	0.457530
12	6	0	1.430783	-0.341979	-0.217147
13	6	0	2.364444	-0.553271	-1.239011
14	6	0	3.294583	0.412028	-1.591446
15	6	0	3.330556	1.624349	-0.912081
16	6	0	2.425515	1.851480	0.109543
17	6	0	-2.010870	0.664354	-0.581881
18	8	0	-2.331459	0.084531	-1.596426
19	8	0	-2.295000	1.951665	-0.330314
20	6	0	-3.030512	2.628913	-1.351689
21	1	0	0.489146	-4.855040	0.292009
22	1	0	2.026613	-2.968173	-0.048719
23	1	0	-2.778211	-2.102359	0.693857
24	1	0	-1.935028	-4.440509	0.638960
25	1	0	-0.994357	0.169486	2.502562
26	1	0	-1.494701	1.691162	1.781132
27	1	0	2.341259	-1.490037	-1.781858
28	1	0	3.988211	0.217255	-2.400825
29	1	0	4.054572	2.384611	-1.178420
30	1	0	2.440031	2.783658	0.660371
31	1	0	-3.999939	2.155237	-1.504278
32	1	0	-3.159969	3.645094	-0.988544
33	1	0	-2.473666	2.629306	-2.288163

IAh

E-amide

Zero-point correction= 0.259500 (Hartree/Particle)

Thermal correction to Energy= 0.275732

Thermal correction to Enthalpy= 0.276676

Thermal correction to Gibbs Free Energy= 0.214780

Sum of electronic and zero-point Energies= -897.738609

Sum of electronic and thermal Energies= -897.722377

Sum of electronic and thermal Enthalpies= -897.721432

Sum of electronic and thermal Free Energies= -897.783329

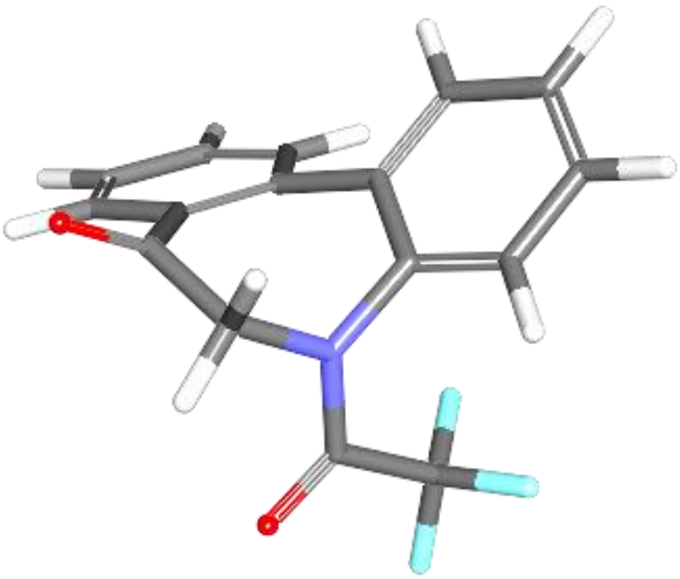

Center Number	Atomic Number	Atomic Type	Coordinates (Angstroms) X	Y	Z
1	6	0	0.810798	3.584251	0.332171
2	6	0	-0.296135	2.844053	-0.059130
3	6	0	-0.328896	1.455731	0.097635
4	6	0	0.789570	0.840006	0.670279
5	6	0	1.893992	1.575617	1.078371
6	6	0	1.910976	2.952096	0.898022
7	7	0	0.728054	-0.566105	0.921130
8	6	0	-0.150947	-0.947808	2.010068
9	6	0	-1.636323	-0.832622	1.701366
10	8	0	-2.421918	-1.195096	2.549740
11	6	0	-2.099815	-0.363087	0.364627
12	6	0	-1.495686	0.674557	-0.374018
13	6	0	-2.067039	1.025856	-1.601331
14	6	0	-3.189141	0.376119	-2.092909
15	6	0	-3.788056	-0.636594	-1.352607
16	6	0	-3.248837	-0.989066	-0.127362
17	6	0	1.331466	-1.544945	0.205981
18	8	0	1.237544	-2.729455	0.441595
19	6	0	2.219122	-1.103861	-0.991425
20	9	0	3.345152	-0.511164	-0.566792
21	9	0	1.591725	-0.255471	-1.814018
22	9	0	2.568329	-2.168938	-1.700644
23	1	0	0.808575	4.659802	0.203177
24	1	0	-1.157056	3.348587	-0.480458
25	1	0	2.732896	1.065739	1.534539
26	1	0	2.773742	3.526595	1.211510
27	1	0	0.068852	-0.337019	2.888627
28	1	0	0.039840	-1.990497	2.266319
29	1	0	-1.607766	1.812417	-2.187508
30	1	0	-3.593912	0.661112	-3.056681
31	1	0	-4.666455	-1.146972	-1.727873
32	1	0	-3.705282	-1.766818	0.472071

Z-amide

Zero-point correction= 0.259605 (Hartree/Particle)

Thermal correction to Energy= 0.275851

Thermal correction to Enthalpy= 0.276795

Thermal correction to Gibbs Free Energy= 0.214511

Sum of electronic and zero-point Energies= -897.737162

Sum of electronic and thermal Energies= -897.720916

Sum of electronic and thermal Enthalpies= -897.719971

Sum of electronic and thermal Free Energies= -897.782256

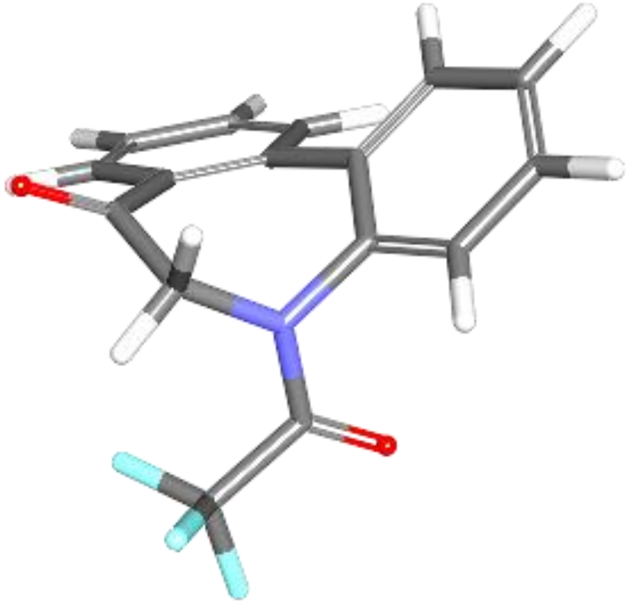

Center Number	Atomic Number	Atomic Type	Coordinates (Angstroms) X	Y	Z
1	6	0	2.748998	-3.056915	0.225847
2	6	0	2.809149	-1.681384	0.060158
3	6	0	1.647436	-0.900800	0.063254
4	6	0	0.430438	-1.556538	0.263768
5	6	0	0.359683	-2.933174	0.423436
6	6	0	1.522475	-3.689213	0.397648
7	7	0	-0.757959	-0.766789	0.361750
8	6	0	-0.845526	0.019625	1.579389
9	6	0	0.042087	1.257042	1.615135
10	8	0	-0.103306	2.025482	2.540961
11	6	0	1.003720	1.536738	0.513441
12	6	0	1.723794	0.555527	-0.199615
13	6	0	2.608016	0.987496	-1.194258
14	6	0	2.780131	2.332508	-1.482858
15	6	0	2.079747	3.294564	-0.764254
16	6	0	1.207858	2.891436	0.231535
17	6	0	-1.603594	-0.739672	-0.695756
18	8	0	-1.452688	-1.364974	-1.720140
19	6	0	-2.864732	0.156254	-0.558744
20	9	0	-2.541684	1.435125	-0.307641
21	9	0	-3.567242	0.127776	-1.678681
22	9	0	-3.661404	-0.265528	0.437551
23	1	0	3.663919	-3.636905	0.223937
24	1	0	3.770681	-1.197847	-0.062078
25	1	0	-0.606630	-3.400689	0.566540
26	1	0	1.471504	-4.763833	0.521555
27	1	0	-0.564773	-0.619330	2.420673
28	1	0	-1.863457	0.349983	1.773507
29	1	0	3.155130	0.247635	-1.765271
30	1	0	3.460236	2.627568	-2.272990
31	1	0	2.209827	4.347604	-0.981098
32	1	0	0.656630	3.622028	0.810137

IBg

E-amide

Zero-point correction= 0.258752 (Hartree/Particle)

Thermal correction to Energy= 0.277547

Thermal correction to Enthalpy= 0.278491

Thermal correction to Gibbs Free Energy= 0.210633

Sum of electronic and zero-point Energies= -1159.566116

Sum of electronic and thermal Energies= -1159.547322

Sum of electronic and thermal Enthalpies= -1159.546377

Sum of electronic and thermal Free Energies= -1159.614235

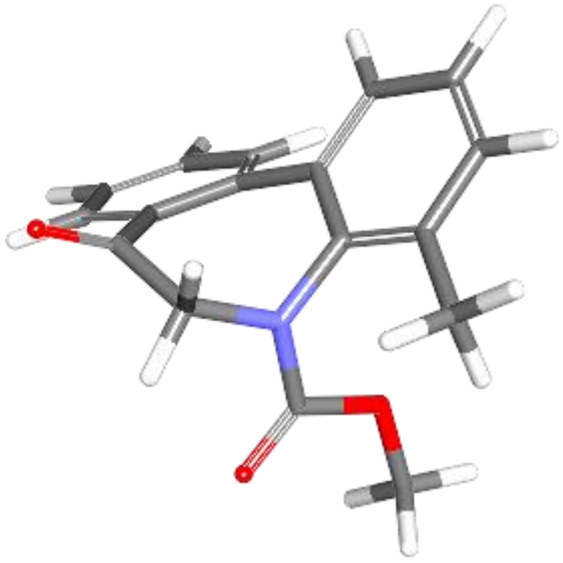

Center Number	Atomic Number	Atomic Type	Coordinates (Angstroms) X	Y	Z
1	6	0	1.297085	3.301299	-0.703457
2	6	0	0.065110	2.679930	-0.848890
3	6	0	-0.150551	1.410036	-0.313709
4	6	0	0.909186	0.783131	0.355437
5	6	0	2.144501	1.407453	0.549756
6	6	0	3.256094	0.769223	1.329991
7	6	0	2.319784	2.674889	-0.007884
8	7	0	0.637530	-0.487060	0.959744
9	6	0	-0.143230	-0.411390	2.179705
10	6	0	-1.612259	-0.075319	1.958933
11	8	0	-2.337892	-0.021909	2.927595
12	6	0	-2.145333	0.091490	0.575870
13	6	0	-1.468968	0.751041	-0.471072
14	6	0	-2.111873	0.849717	-1.708812
15	6	0	-3.372758	0.311500	-1.917350
16	6	0	-4.039597	-0.324993	-0.876874
17	6	0	-3.428803	-0.419345	0.361930
18	6	0	0.830737	-1.706483	0.402545
19	8	0	0.501987	-2.755187	0.912154
20	6	0	1.515975	-1.751675	-0.993211
21	9	0	1.652127	-3.013545	-1.379160
22	9	0	2.735512	-1.197992	-0.982688
23	9	0	0.786964	-1.117660	-1.920244
24	1	0	1.452133	4.291082	-1.116050
25	1	0	-0.743712	3.190501	-1.356654
26	1	0	3.942911	0.236532	0.668333
27	1	0	2.885267	0.050888	2.061175
28	1	0	3.831228	1.531389	1.856985
29	1	0	3.269817	3.179931	0.125224
30	1	0	0.289420	0.339253	2.844336
31	1	0	-0.109999	-1.375942	2.687494
32	1	0	-1.600767	1.342696	-2.526713
33	1	0	-3.833317	0.388761	-2.895052

34	1	0	-5.026151	-0.744564	-1.030653
35	1	0	-3.934182	-0.901101	1.189669

Z-amide

Zero-point correction= 0.258922 (Hartree/Particle)

Thermal correction to Energy= 0.277640

Thermal correction to Enthalpy= 0.278584

Thermal correction to Gibbs Free Energy= 0.211341

Sum of electronic and zero-point Energies= -1159.565333

Sum of electronic and thermal Energies= -1159.546615

Sum of electronic and thermal Enthalpies= -1159.545671

Sum of electronic and thermal Free Energies= -1159.612913

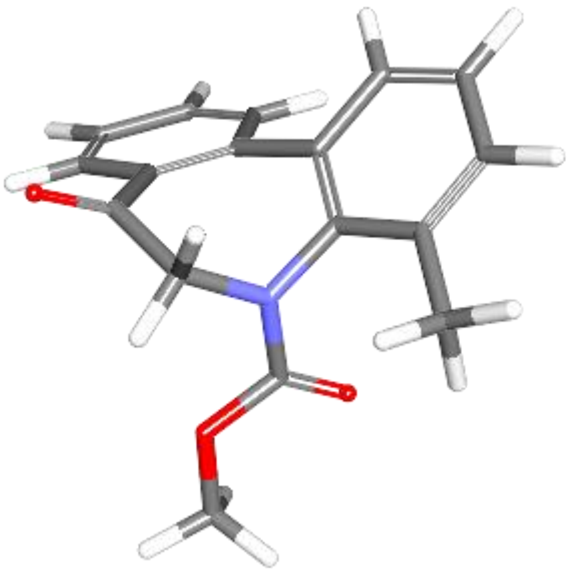

Center Number	Atomic Number	Atomic Type	Coordinates (Angstroms) X	Y	Z
1	6	0	-1.572482	3.702901	0.052616
2	6	0	-2.121273	2.435198	-0.055434
3	6	0	-1.303041	1.302550	-0.008569
4	6	0	0.070207	1.493083	0.166267
5	6	0	0.647833	2.760720	0.270754
6	6	0	2.127842	2.931913	0.438600
7	6	0	-0.201619	3.863298	0.206477
8	7	0	0.888154	0.324774	0.299690
9	6	0	0.716913	-0.374722	1.560530
10	6	0	-0.557736	-1.201780	1.668650
11	8	0	-0.686215	-1.914526	2.640383
12	6	0	-1.567742	-1.178379	0.575928
13	6	0	-1.891514	-0.045117	-0.199094
14	6	0	-2.877403	-0.187283	-1.181891
15	6	0	-3.521595	-1.395488	-1.399900
16	6	0	-3.209400	-2.502951	-0.619615
17	6	0	-2.245388	-2.383735	0.365541
18	6	0	1.612511	-0.080405	-0.769181
19	8	0	1.666081	0.502059	-1.828703
20	6	0	2.448252	-1.377937	-0.599734
21	9	0	3.058060	-1.678550	-1.734004
22	9	0	1.680239	-2.423981	-0.255527
23	9	0	3.389759	-1.234682	0.348313
24	1	0	-2.217877	4.572617	0.022057
25	1	0	-3.192250	2.315590	-0.162635
26	1	0	2.512776	2.329786	1.264874
27	1	0	2.372743	3.975239	0.635655
28	1	0	2.658863	2.623738	-0.465480
29	1	0	0.220459	4.858387	0.288534
30	1	0	1.551068	-1.040841	1.771100
31	1	0	0.704950	0.366011	2.364288
32	1	0	-3.126622	0.666654	-1.799490
33	1	0	-4.266216	-1.470945	-2.183406

34	1	0	-3.709711	-3.449790	-0.781032
35	1	0	-1.988857	-3.229733	0.990817

IBh

E-amide

Zero-point correction=	0.287065 (Hartree/Particle)
Thermal correction to Energy=	0.305020
Thermal correction to Enthalpy=	0.305964
Thermal correction to Gibbs Free Energy=	0.240651
Sum of electronic and zero-point Energies=	-937.029846
Sum of electronic and thermal Energies=	-937.011892
Sum of electronic and thermal Enthalpies=	-937.010948
Sum of electronic and thermal Free Energies=	-937.076261

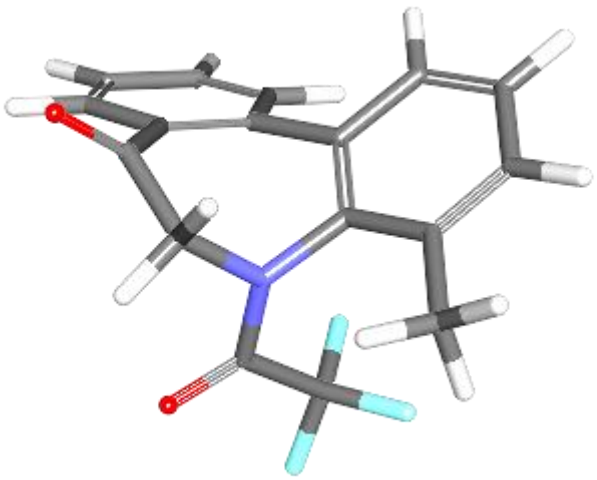

Center Number	Atomic Number	Atomic Type	Coordinates (Angstroms) X	Y	Z
1	6	0	1.522640	3.263940	0.028220
2	6	0	0.250428	2.749091	-0.164407
3	6	0	0.013651	1.378236	-0.027814
4	6	0	1.086098	0.549389	0.319563
5	6	0	2.376178	1.051266	0.519589
6	6	0	3.512730	0.142844	0.881840
7	6	0	2.575482	2.421258	0.360803
8	7	0	0.815409	-0.832016	0.538263
9	6	0	0.087168	-1.154587	1.740166

10	6	0	-1.376804	-0.746878	1.735422
11	8	0	-2.052086	-1.000940	2.710496
12	6	0	-1.978010	-0.129861	0.517440
13	6	0	-1.337186	0.828007	-0.294708
14	6	0	-2.044659	1.334343	-1.390738
15	6	0	-3.332422	0.912538	-1.685281
16	6	0	-3.962820	-0.020942	-0.870564
17	6	0	-3.287686	-0.523951	0.228376
18	6	0	1.060859	-1.811318	-0.380712
19	8	0	0.789423	-2.983875	-0.223641
20	8	0	1.648345	-1.315287	-1.476886
21	6	0	1.953405	-2.270255	-2.495331
22	1	0	1.693550	4.329255	-0.072213
23	1	0	-0.571320	3.411997	-0.406280
24	1	0	3.806086	-0.472290	0.027058
25	1	0	4.382313	0.720003	1.196250
26	1	0	3.239864	-0.538679	1.690146
27	1	0	3.567511	2.831141	0.514032
28	1	0	0.567157	-0.695819	2.609035
29	1	0	0.109492	-2.236558	1.881537
30	1	0	-1.563027	2.061528	-2.033008
31	1	0	-3.842225	1.313215	-2.553489
32	1	0	-4.969830	-0.353624	-1.090592
33	1	0	-3.762534	-1.243467	0.883852
34	1	0	1.044151	-2.752885	-2.852755
35	1	0	2.643120	-3.025781	-2.119724
36	1	0	2.417348	-1.700288	-3.296034

Z-amide

Zero-point correction=	0.287199 (Hartree/Particle)
Thermal correction to Energy=	0.305132
Thermal correction to Enthalpy=	0.306077
Thermal correction to Gibbs Free Energy=	0.240656
Sum of electronic and zero-point Energies=	-937.028580
Sum of electronic and thermal Energies=	-937.010647
Sum of electronic and thermal Enthalpies=	-937.009703
Sum of electronic and thermal Free Energies=	-937.075124

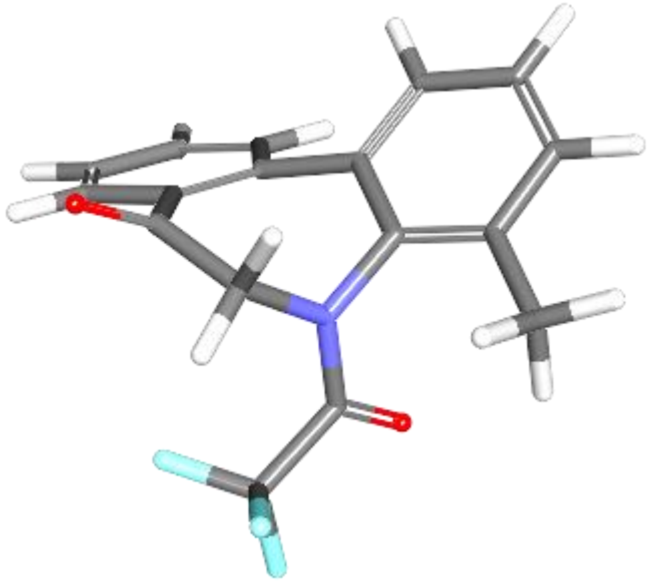

Center Number	Atomic Number	Atomic Type	Coordinates (Angstroms) X	Y	Z
1	6	0	1.660280	-3.359701	0.057819
2	6	0	0.364079	-2.881912	-0.048333
3	6	0	0.109211	-1.507770	0.003754
4	6	0	1.190628	-0.639953	0.189425
5	6	0	2.506194	-1.102521	0.289560
6	6	0	3.649034	-0.148330	0.465660
7	6	0	2.721379	-2.476951	0.213508
8	7	0	0.908471	0.746246	0.351495
9	6	0	0.254651	1.104456	1.589791
10	6	0	-1.225772	0.769719	1.659665
11	8	0	-1.863989	1.173651	2.609639
12	6	0	-1.880546	0.021038	0.549331
13	6	0	-1.270196	-1.003202	-0.205333
14	6	0	-2.036384	-1.623777	-1.199532
15	6	0	-3.348931	-1.254613	-1.449490
16	6	0	-3.947492	-0.254760	-0.691430
17	6	0	-3.214405	0.364790	0.305180
18	6	0	1.084261	1.604048	-0.695204
19	8	0	1.565585	1.292566	-1.763311
20	8	0	0.667975	2.843600	-0.391663
21	6	0	0.833371	3.816827	-1.425165
22	1	0	1.845223	-4.426848	0.023259
23	1	0	-0.460849	-3.574753	-0.160214
24	1	0	3.793479	0.450550	-0.436997
25	1	0	3.466868	0.545827	1.289248
26	1	0	4.573855	-0.688272	0.668984
27	1	0	3.733497	-2.857689	0.292391
28	1	0	0.348127	2.174669	1.767669
29	1	0	0.753020	0.593170	2.418772
30	1	0	-1.579936	-2.399026	-1.802461
31	1	0	-3.902100	-1.746652	-2.240792
32	1	0	-4.973144	0.039409	-0.877580
33	1	0	-3.660627	1.139454	0.916375

34	1	0	0.271746	3.530283	-2.313690
35	1	0	0.442989	4.744163	-1.014185
36	1	0	1.886688	3.929813	-1.680494

8. ^{1}H-, ^{13}C-, and 2D-NMR Spectra

^{1}H-NMR (600 MHz, $CDCl_3$) of **S1a**

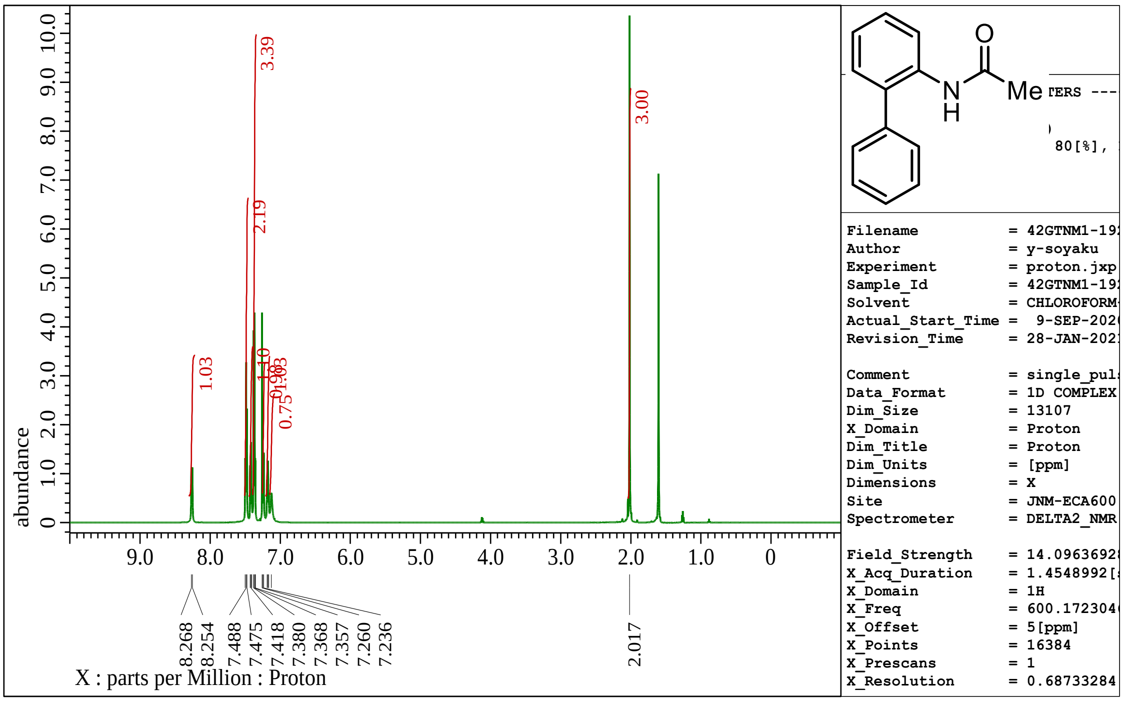

^{13}C{^{1}H }-NMR (150 MHz, $CDCl_3$) of **S1a**

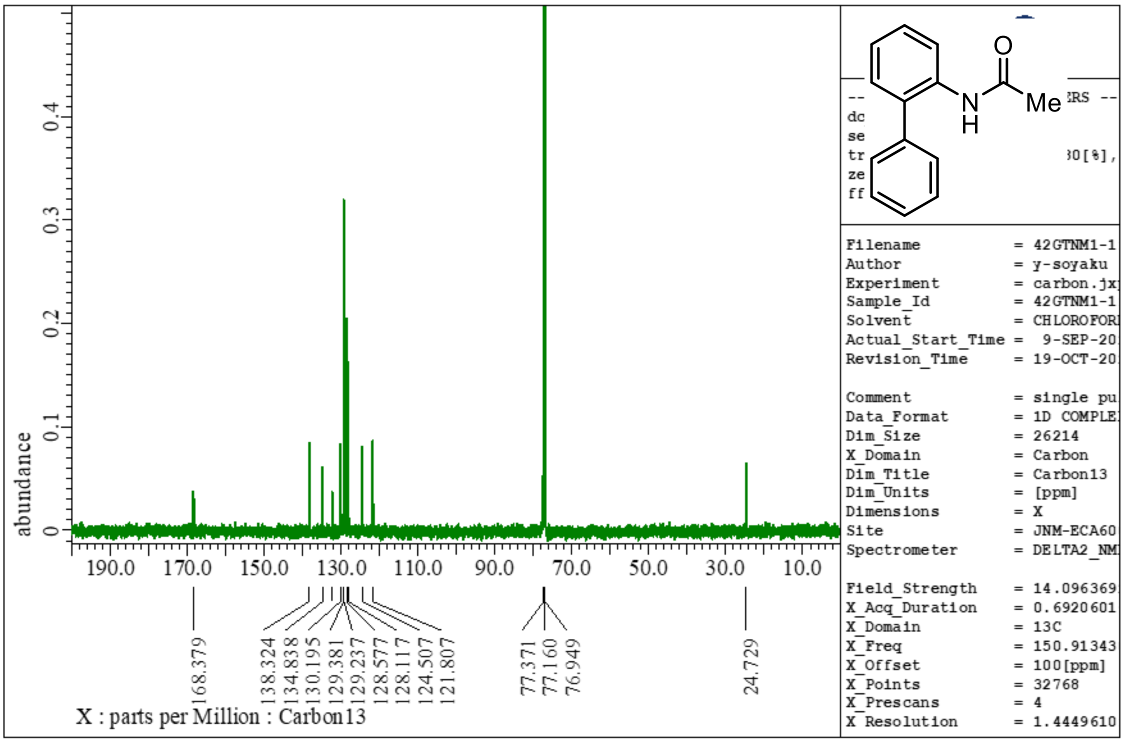

H-H COSY-NMR (600 MHz, $CDCl_3$) of **S1a**

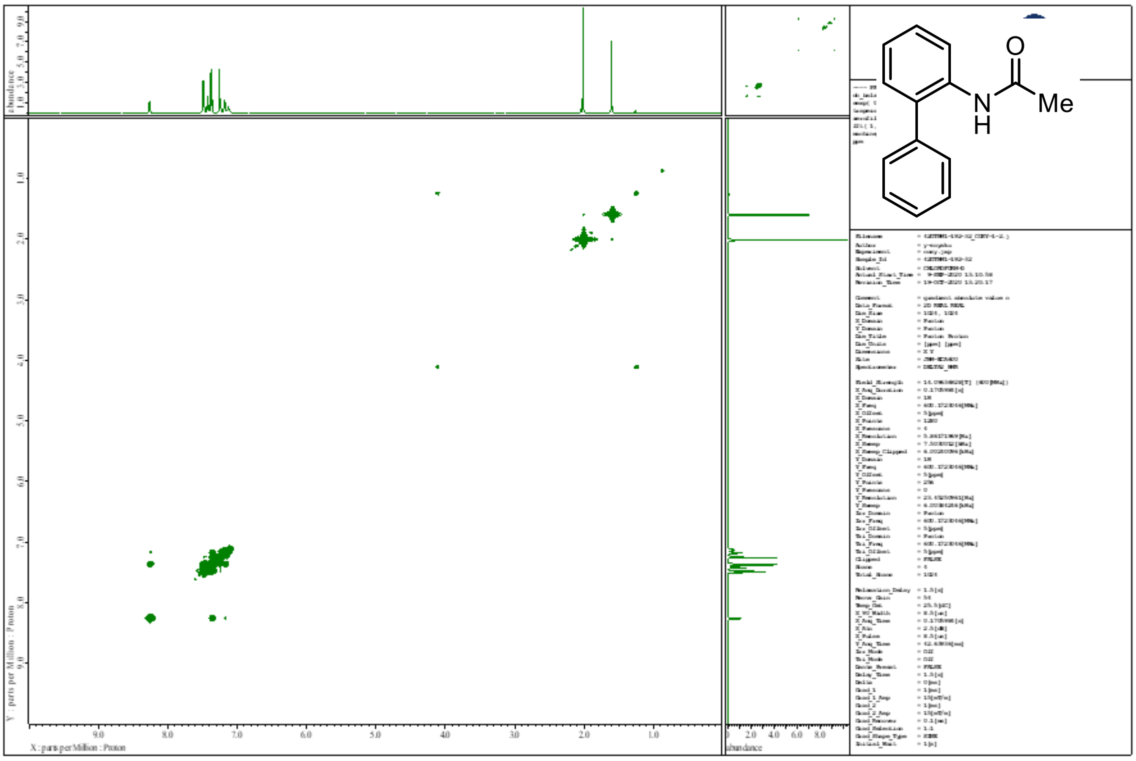

HMQC-NMR ($CDCl_3$) of **S1a**

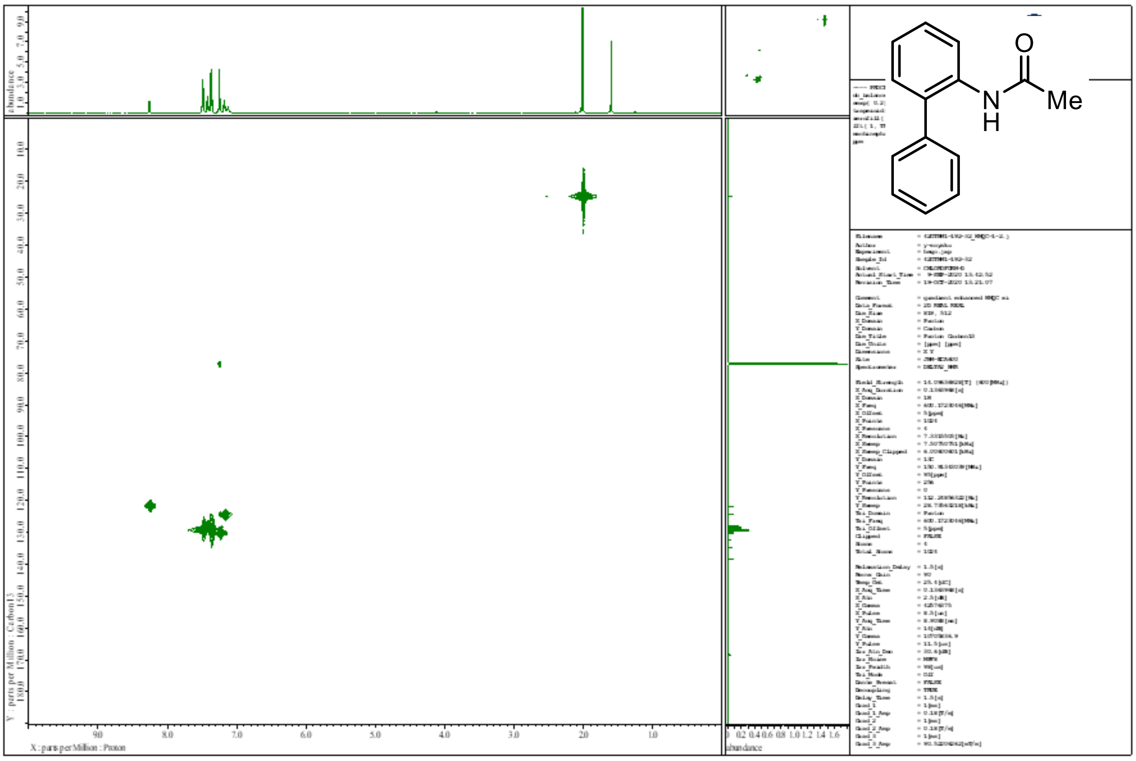

^{1}H-NMR (600 MHz, $CDCl_3$) of **S1b**

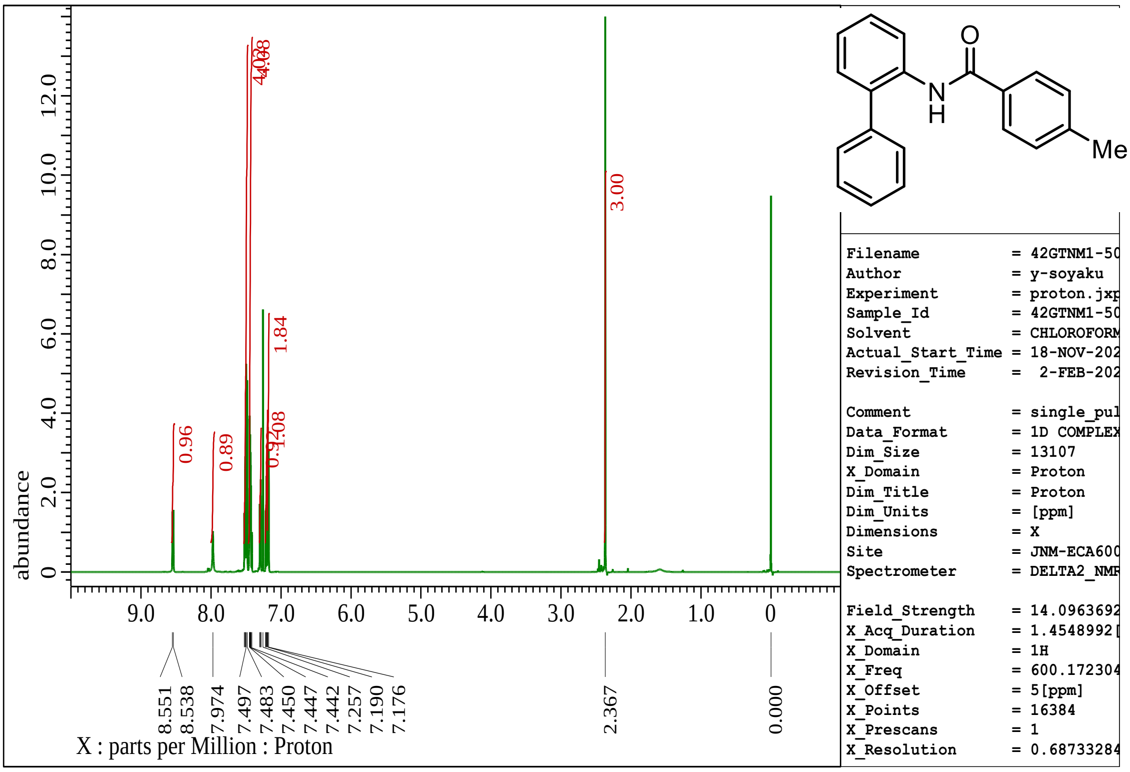

^{13}C{^{1}H }-NMR (150 MHz, $CDCl_3$) of **S1b**

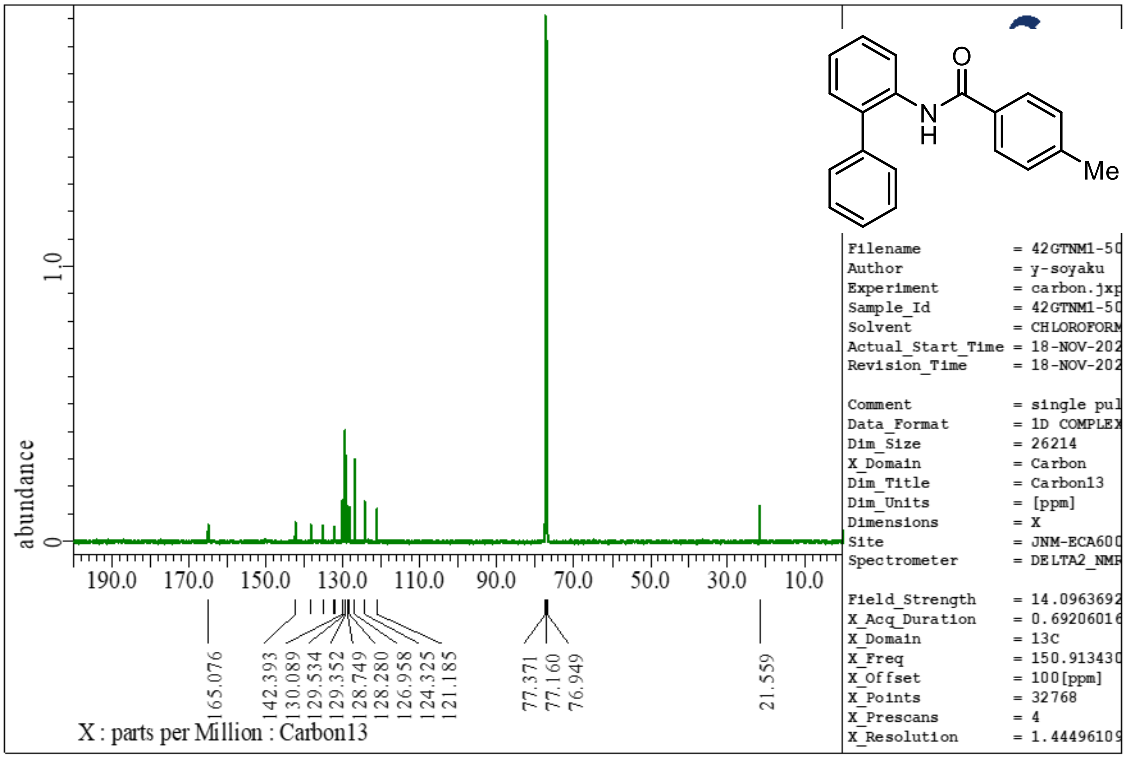

H-H COSY-NMR (600 MHz, $CDCl_3$) of **S1b**

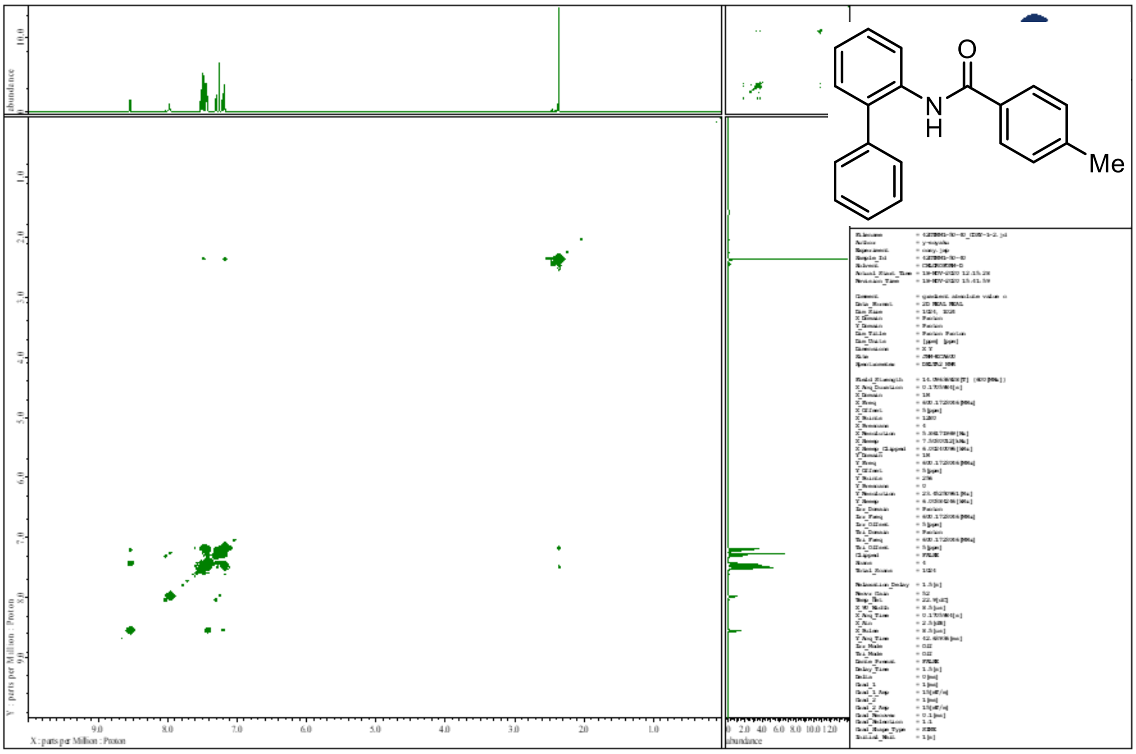

HMQC-NMR ($CDCl_3$) of **S1b**

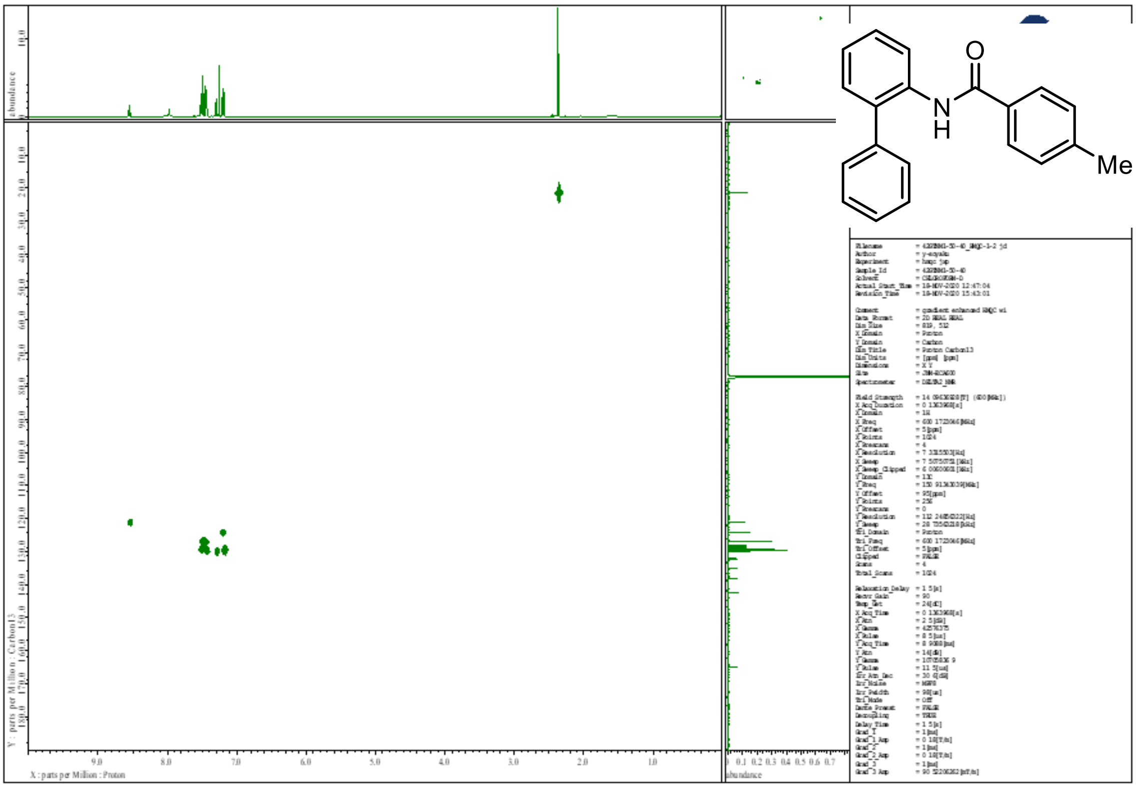

^{1}H-NMR (600 MHz, $CDCl_3$) of **S1g**

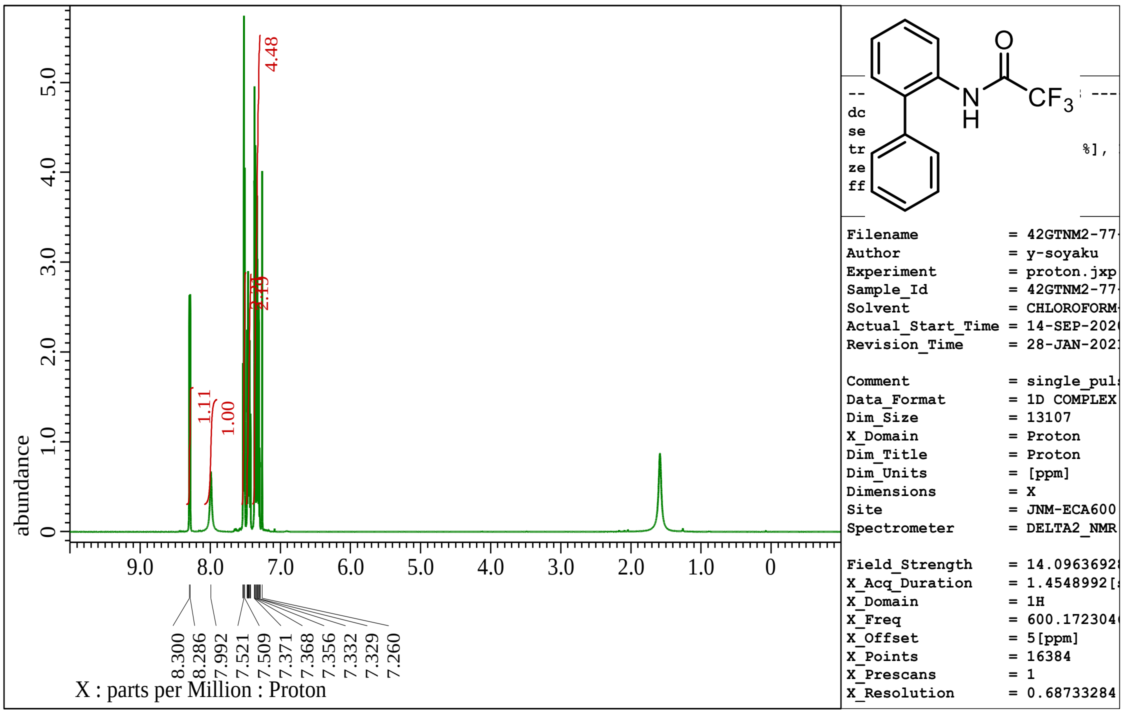

^{13}C{^{1}H }-NMR (150 MHz, $CDCl_3$) of **S1g**

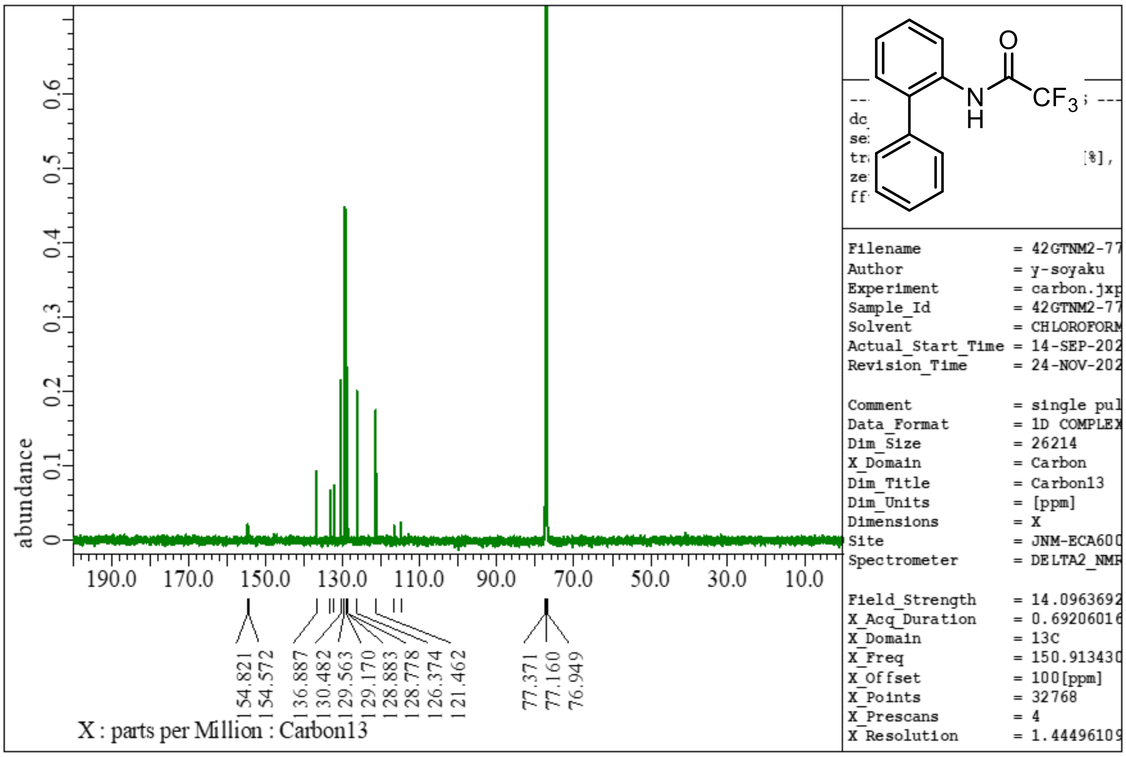

H-H COSY-NMR (600 MHz, $CDCl_3$) of **S1g**

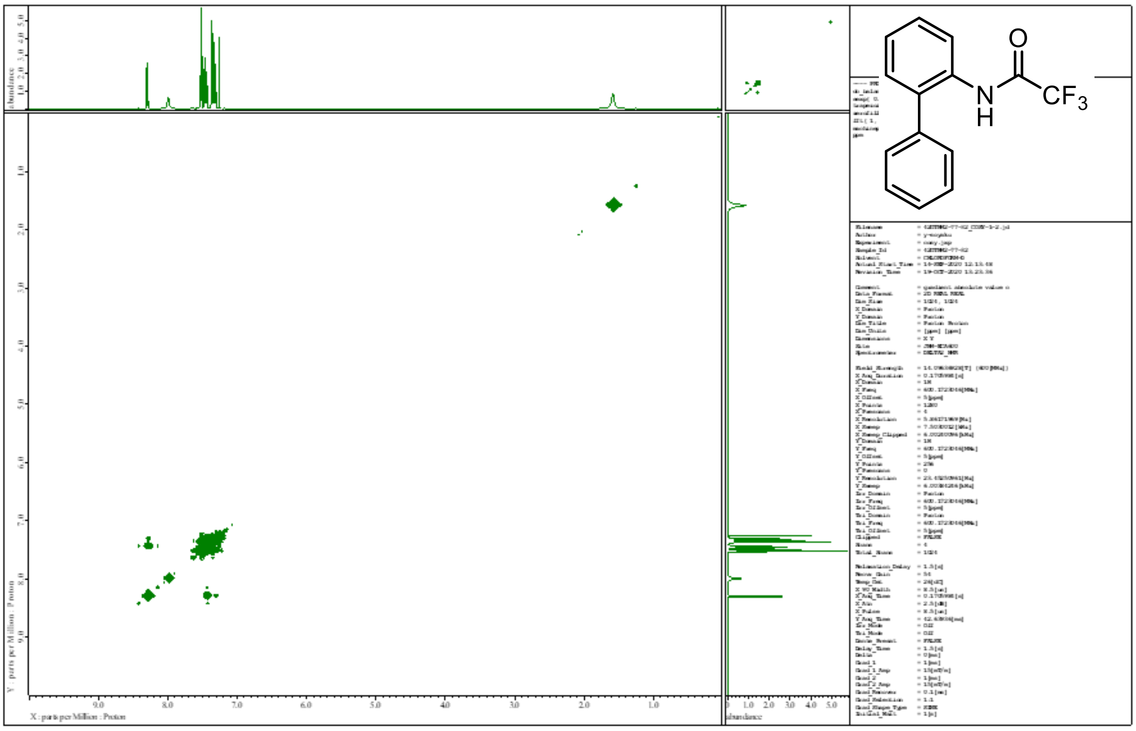

HMQC-NMR ($CDCl_3$) of **S1g**

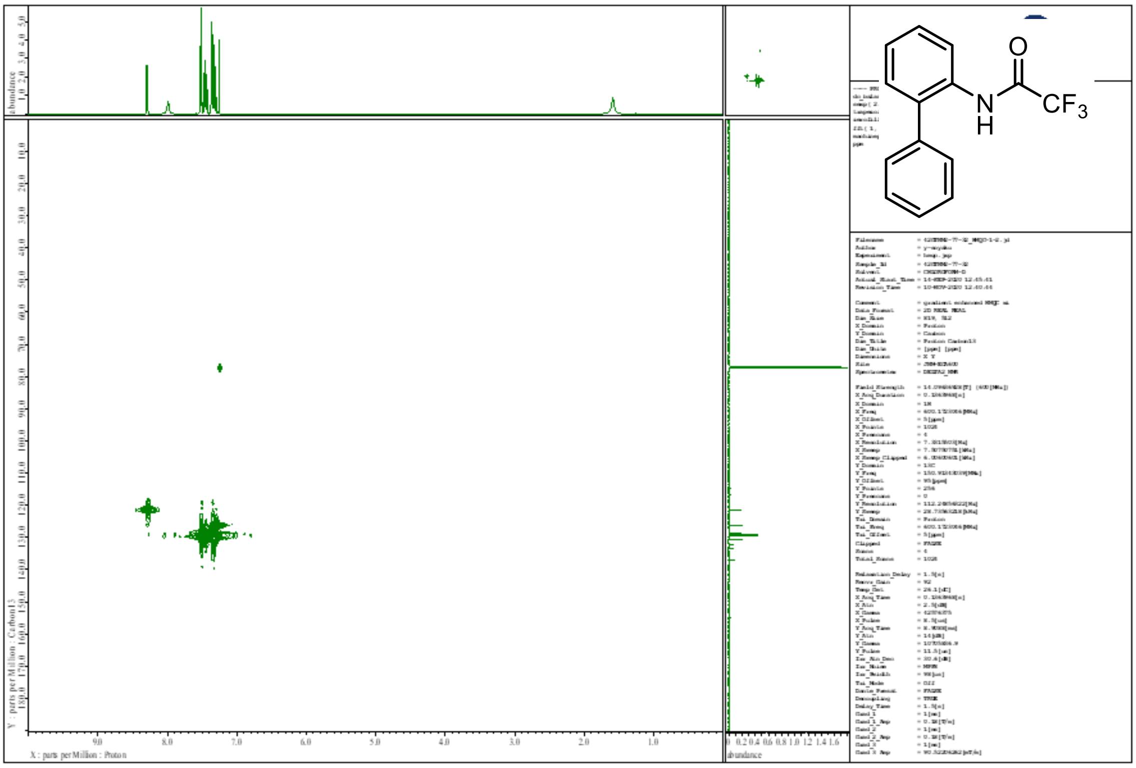

^{1}H-NMR (600 MHz, $CDCl_3$) of **S1h**

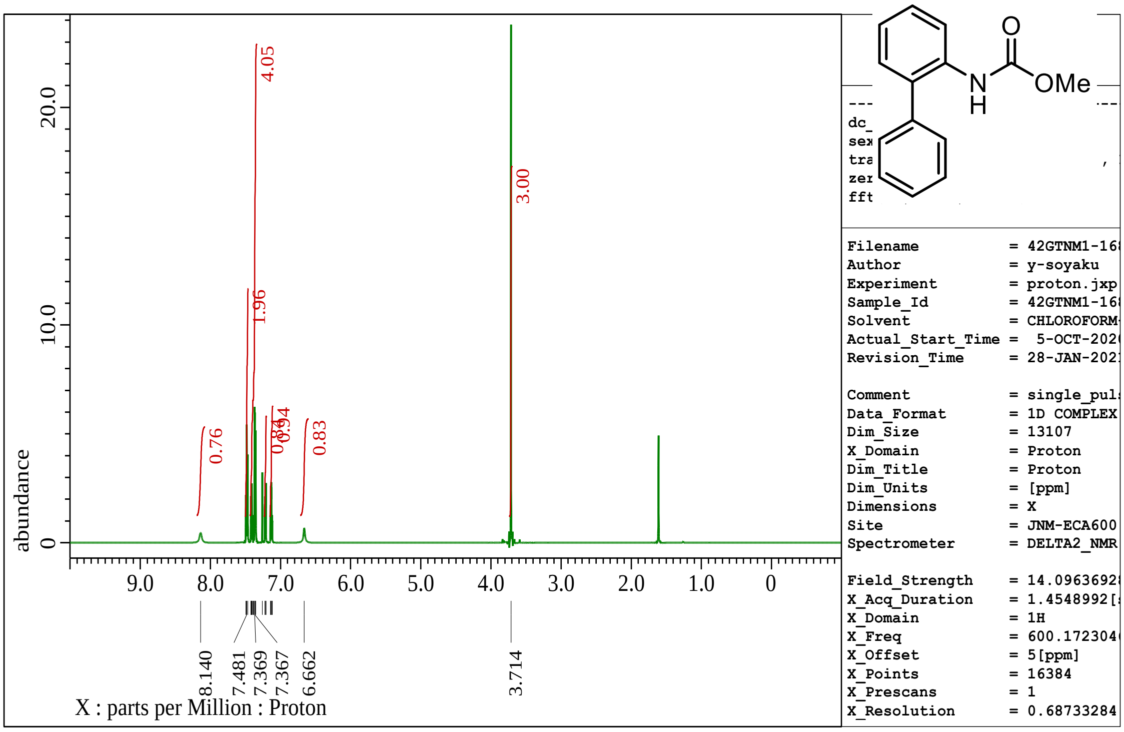

^{13}C{^{1}H }-NMR (150 MHz, $CDCl_3$) of **S1h**

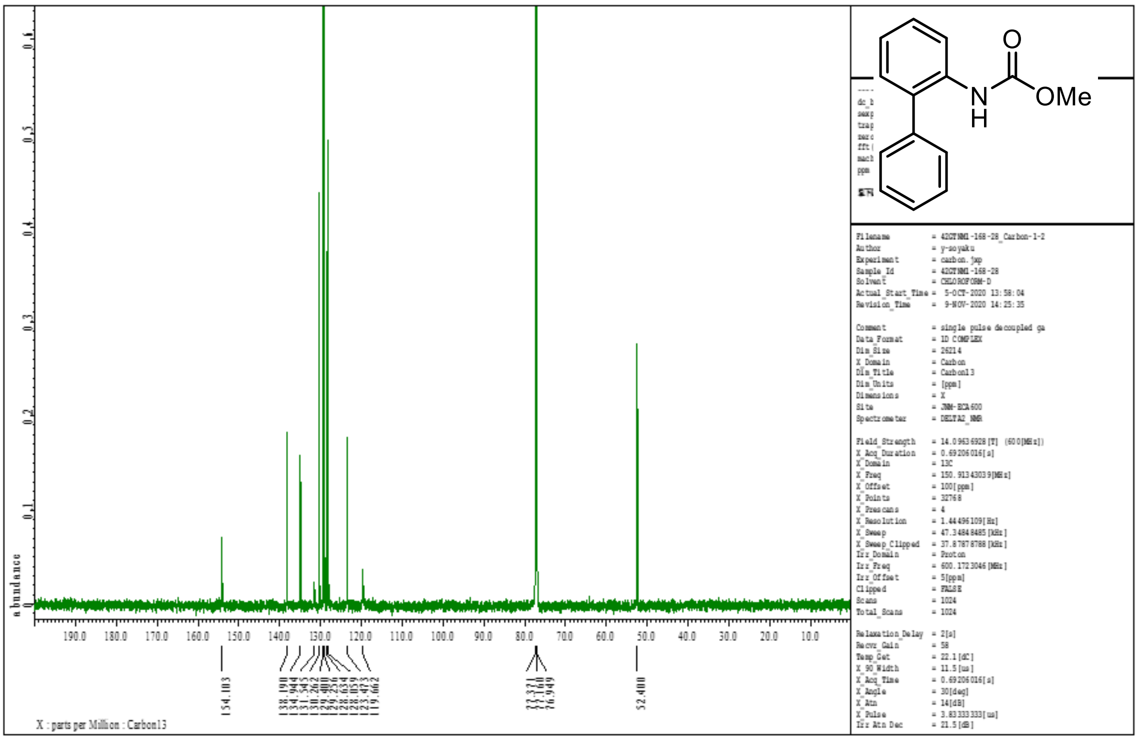

H-H COSY-NMR (600 MHz, $CDCl_3$) of **S1h**

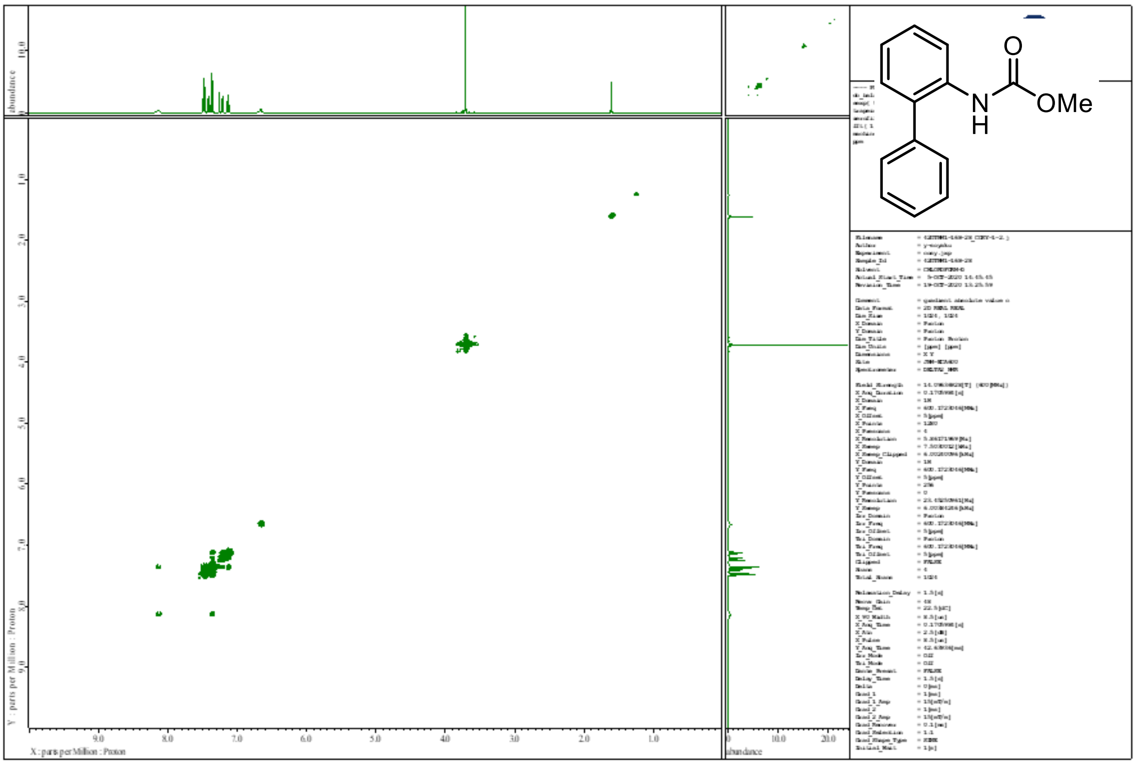

HMQC-NMR ($CDCl_3$) of **S1h**

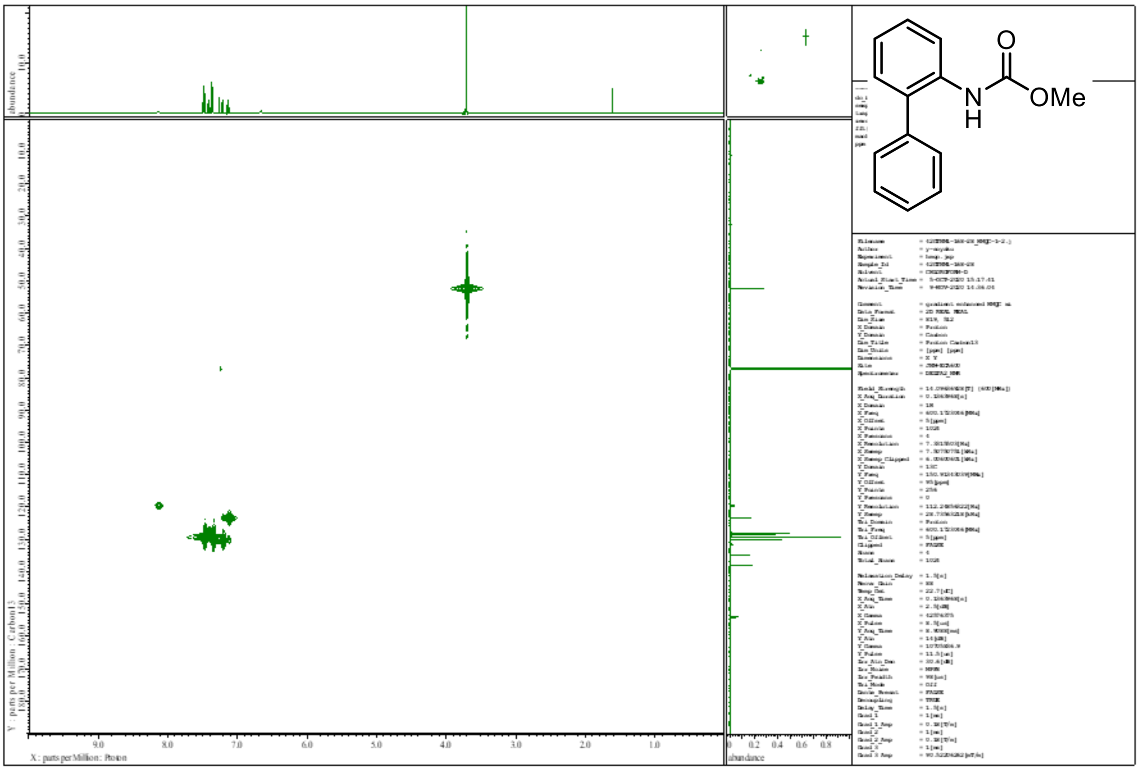

^{1}H-NMR (600 MHz, $CDCl_3$) of **S2a**

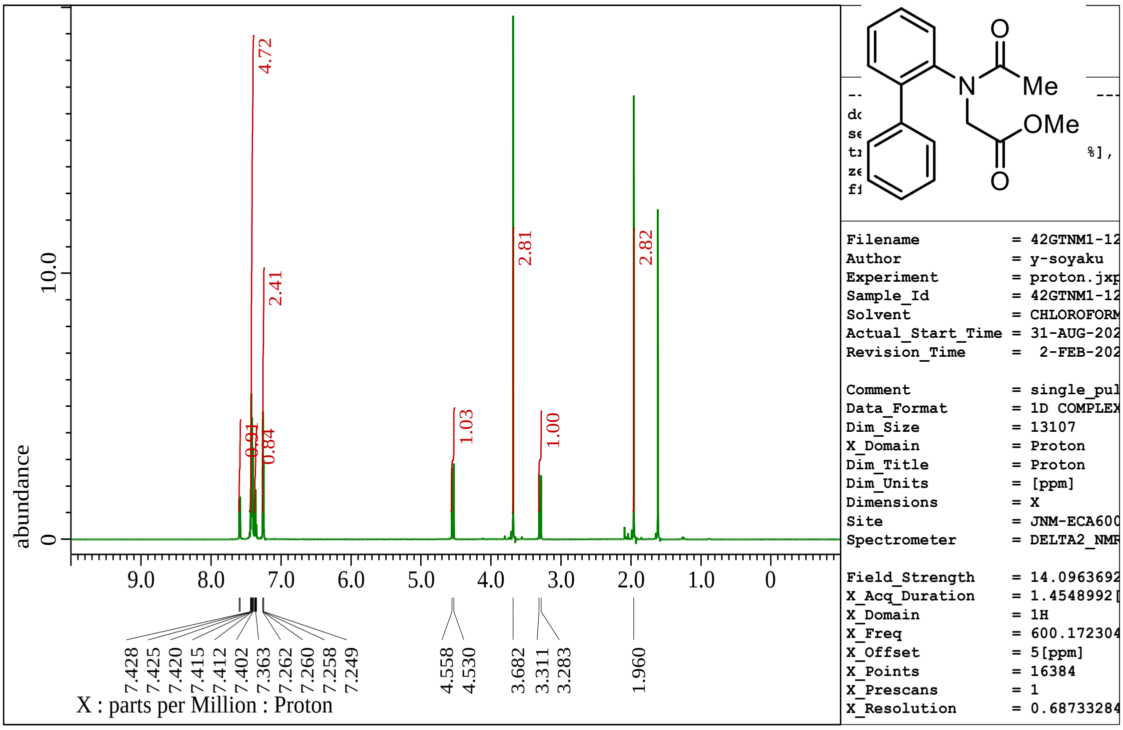

^{13}C{^{1}H }-NMR (150 MHz, $CDCl_3$) of **S2a**

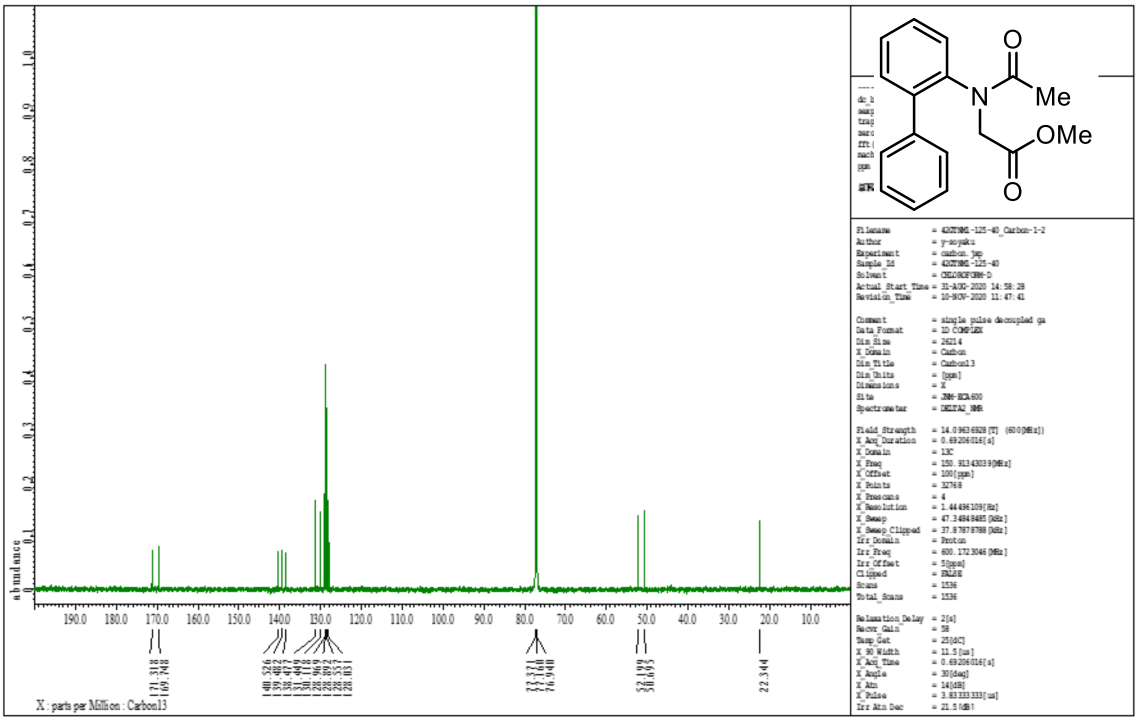

H-H COSY-NMR (600 MHz, $CDCl_3$) of **S2a**

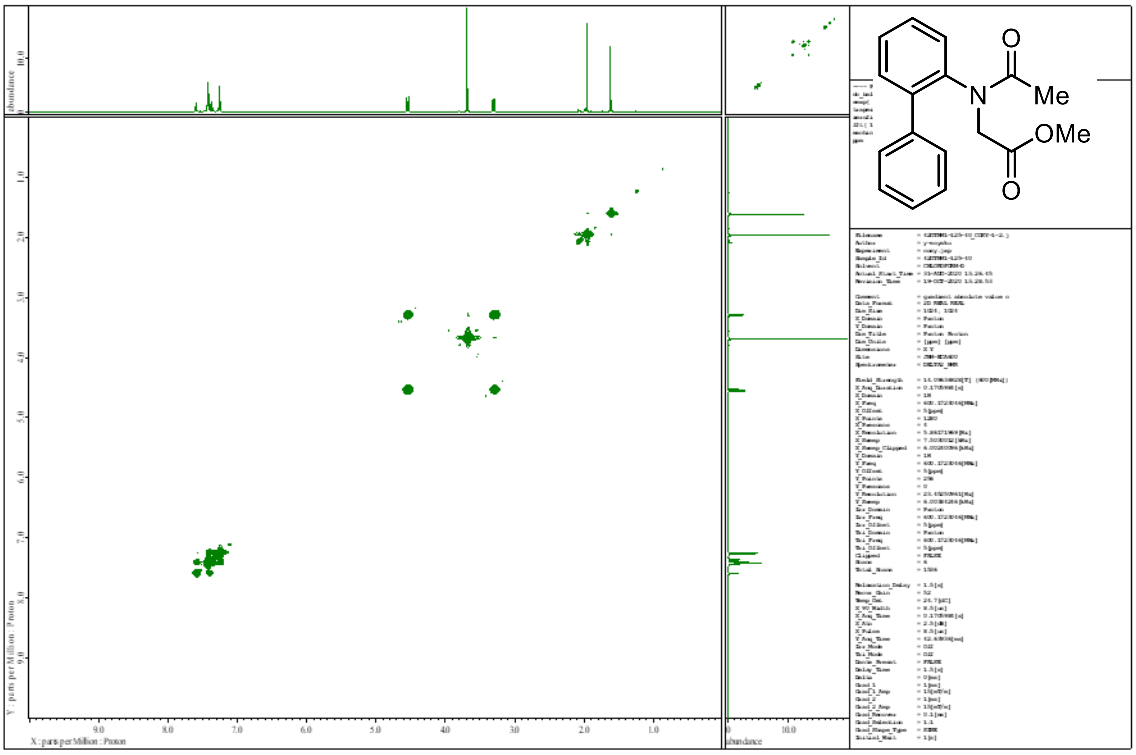

HMQC-NMR ($CDCl_3$) of **S2a**

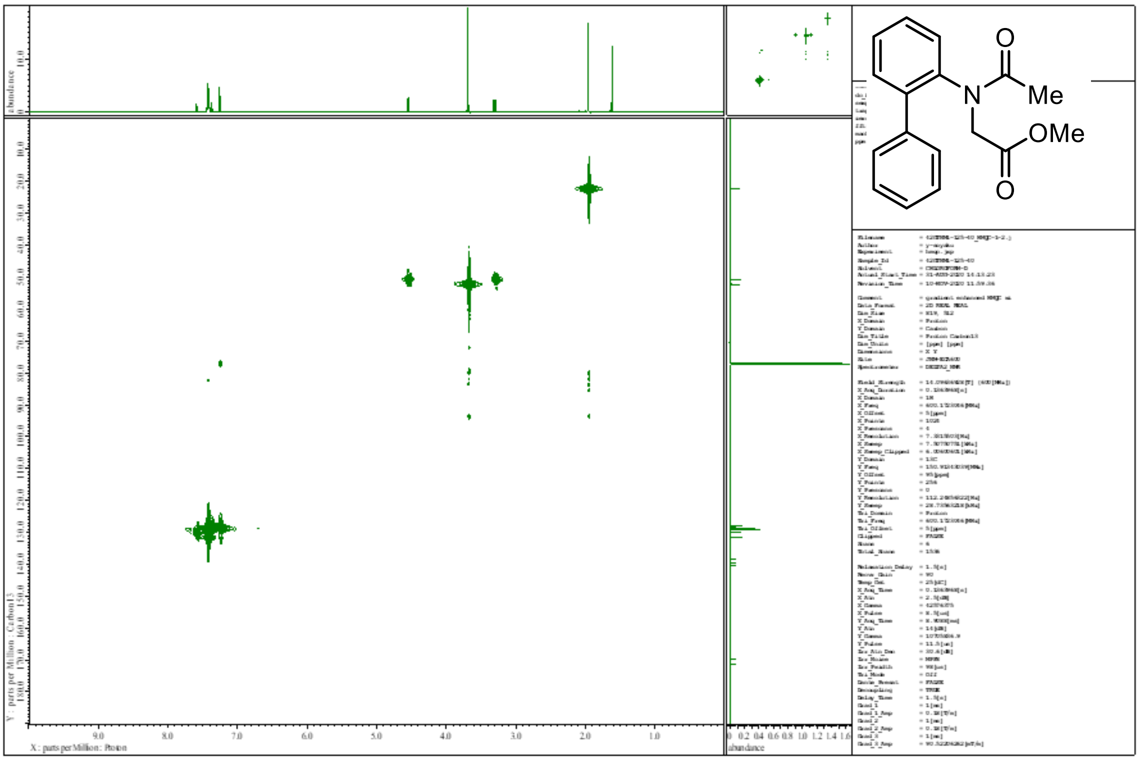

^{1}H-NMR (600 MHz, $CDCl_3$) of **S2b**

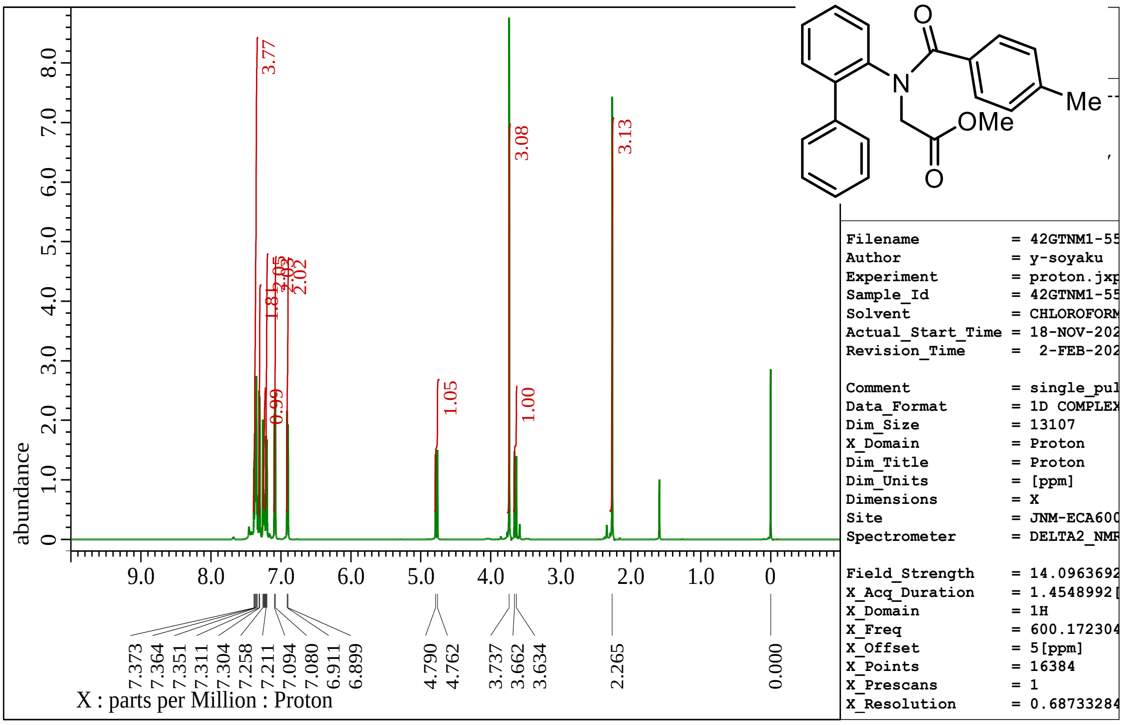

^{13}C{^{1}H }-NMR (150 MHz, $CDCl_3$) of **S2b**

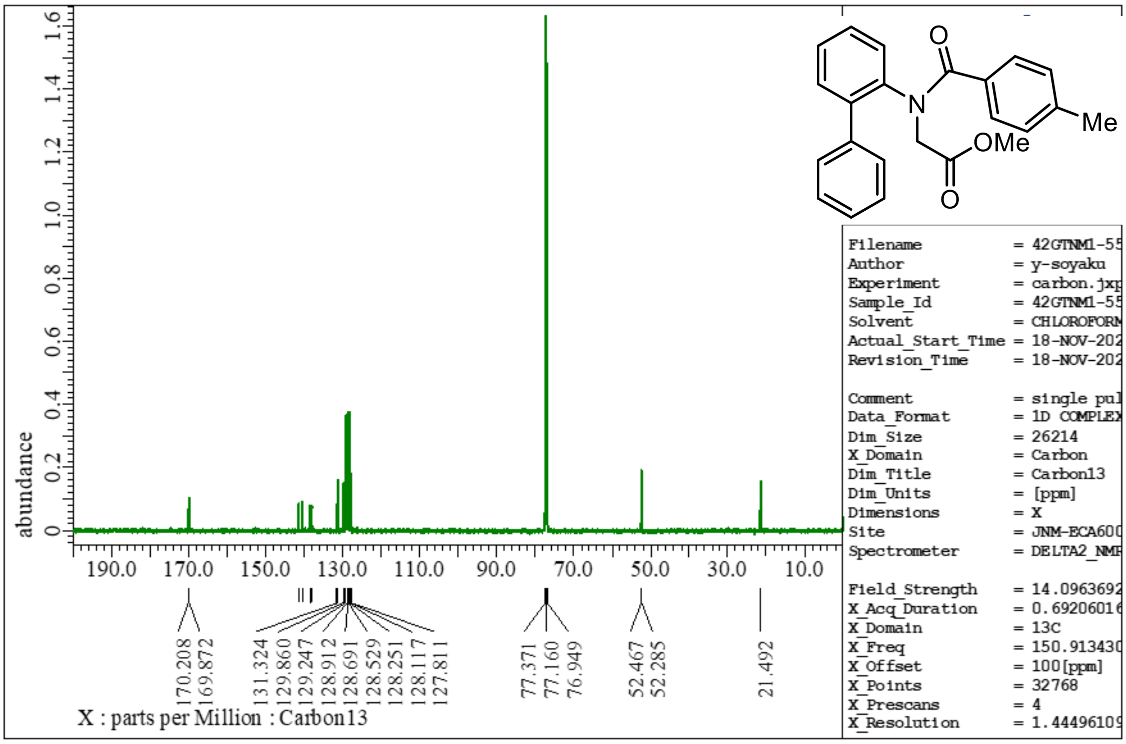

H-H COSY-NMR (600 MHz, $CDCl_3$) of **S2b**

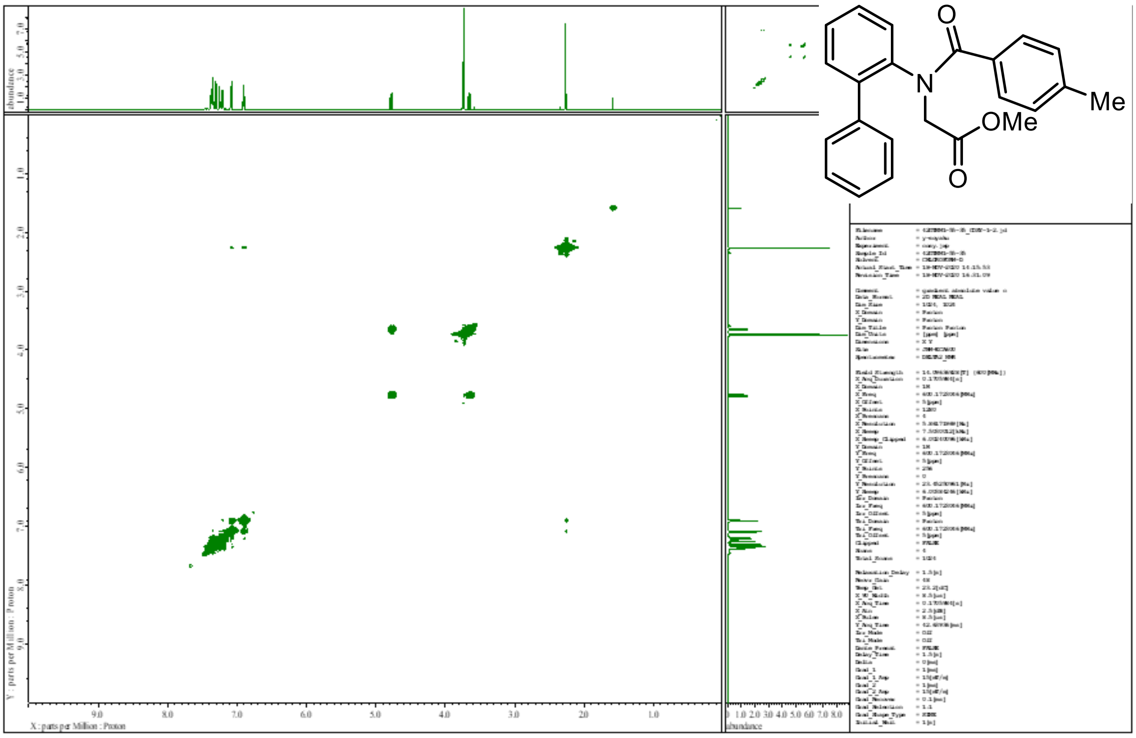

HMQC-NMR ($CDCl_3$) of **S2b**

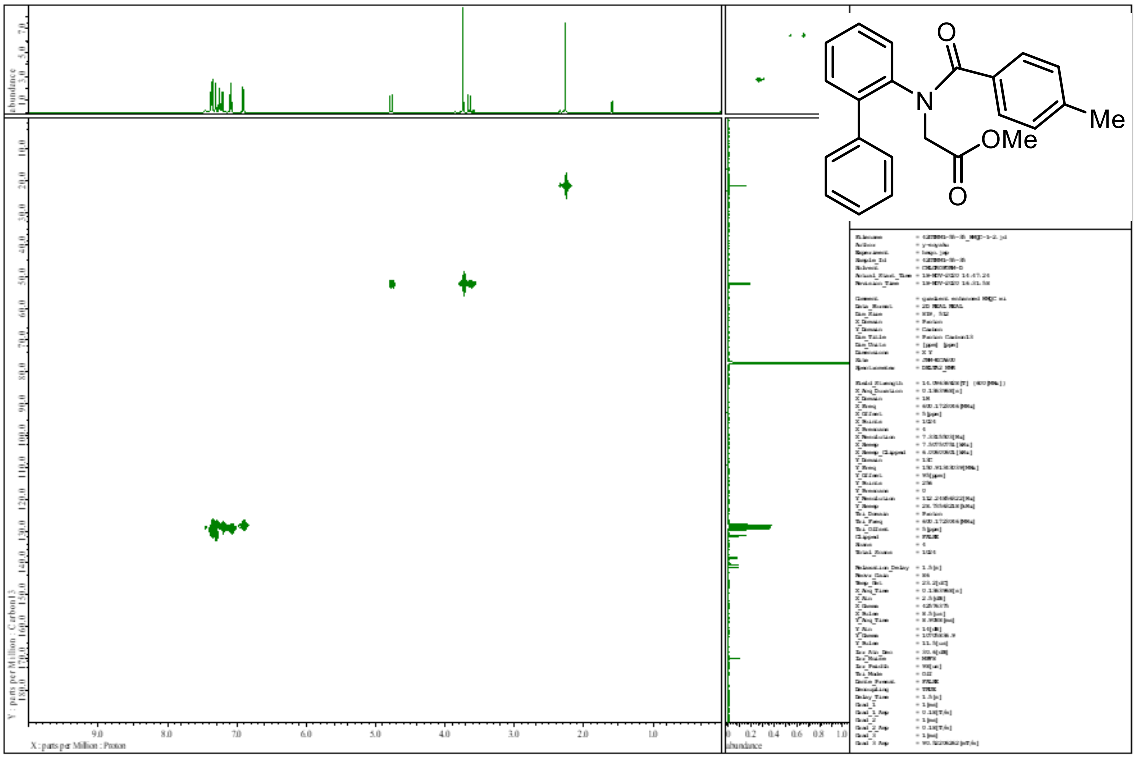

^{1}H-NMR (600 MHz, $CDCl_3$) of **S2g**

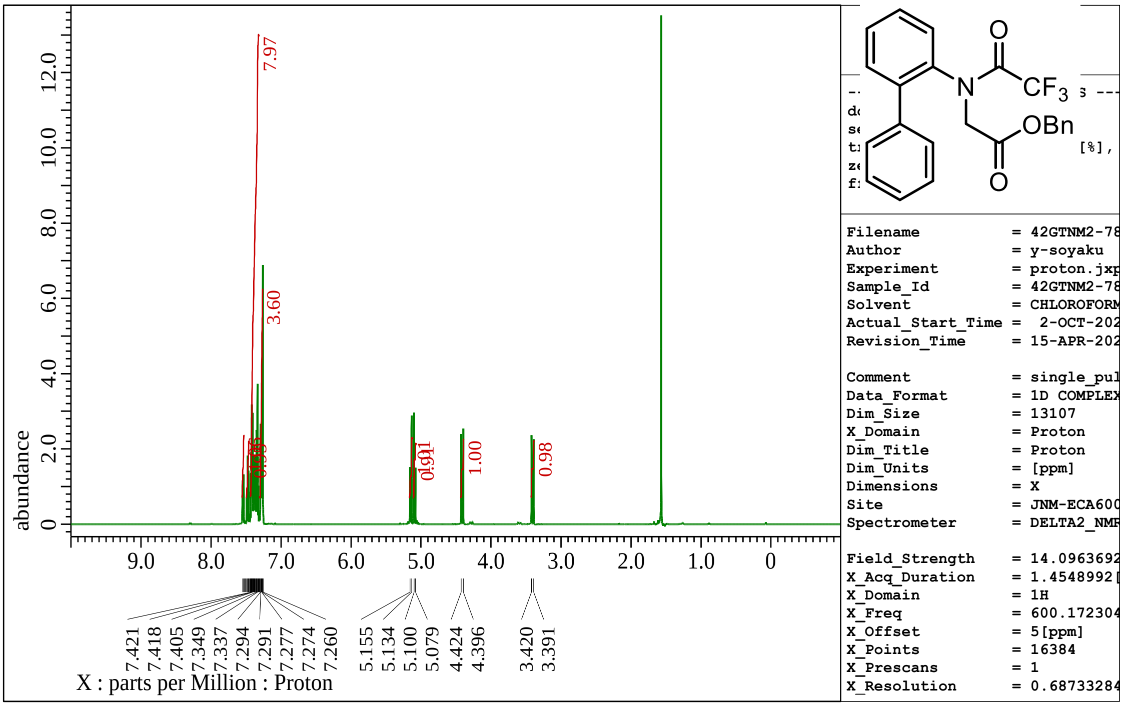

$^{13}C\{^1H\}$-NMR (150 MHz, $CDCl_3$) of **S2g**

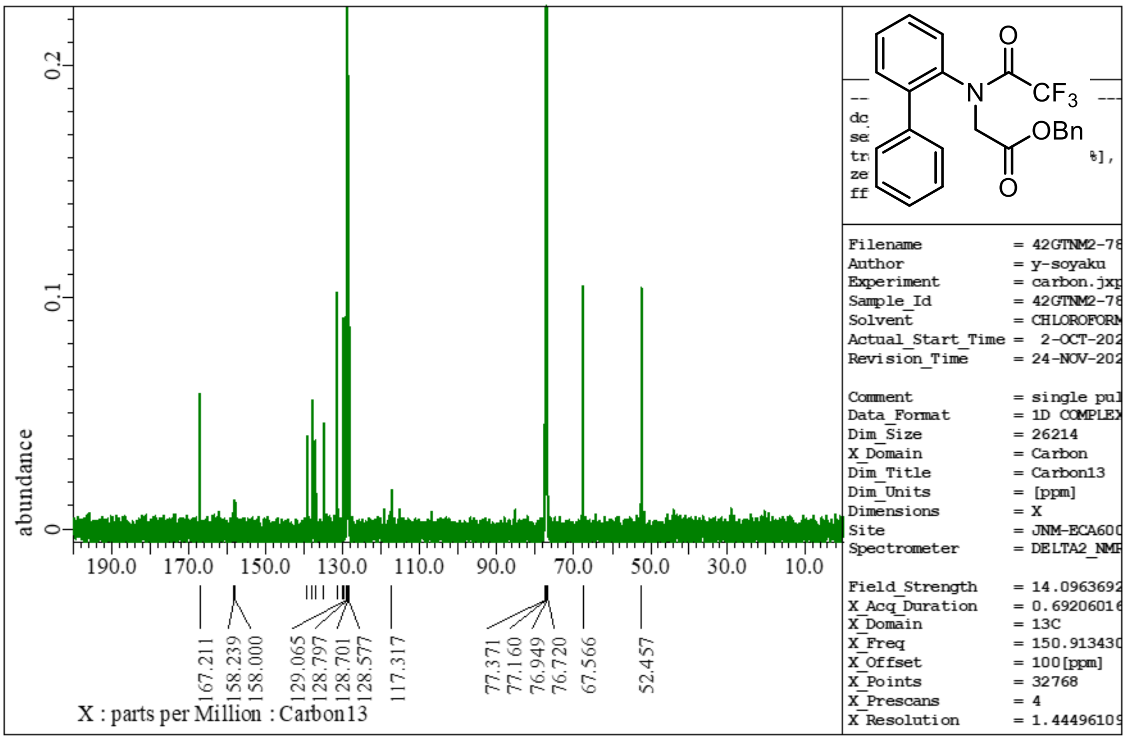

H-H COSY-NMR (600 MHz, $CDCl_3$) of **S2g**

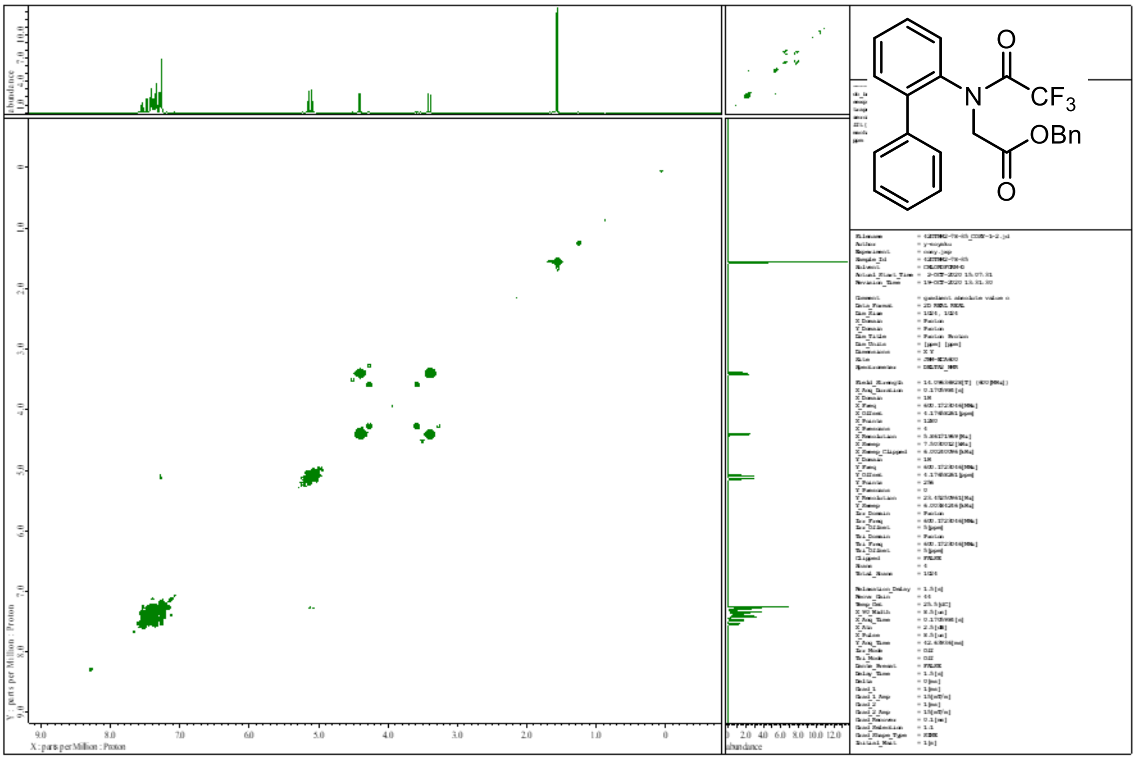

HMQC-NMR ($CDCl_3$) of **S2g**

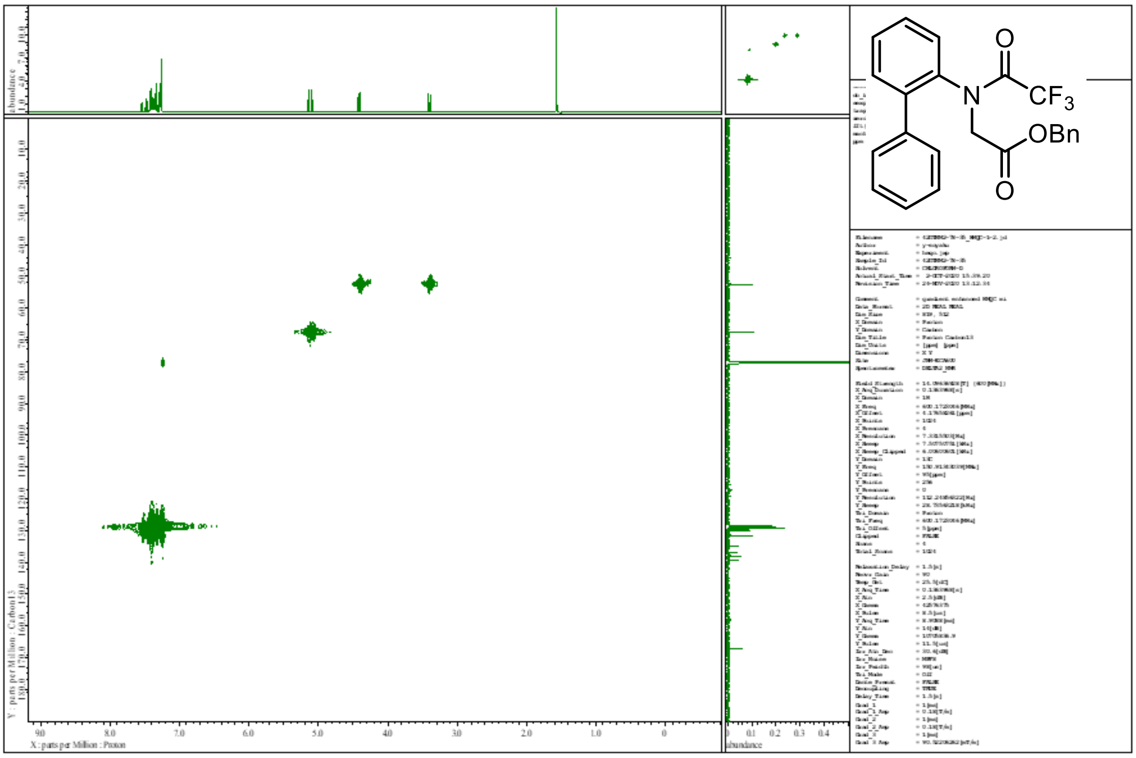

^{1}H-NMR (600 MHz, $CDCl_3$) of **S2h**

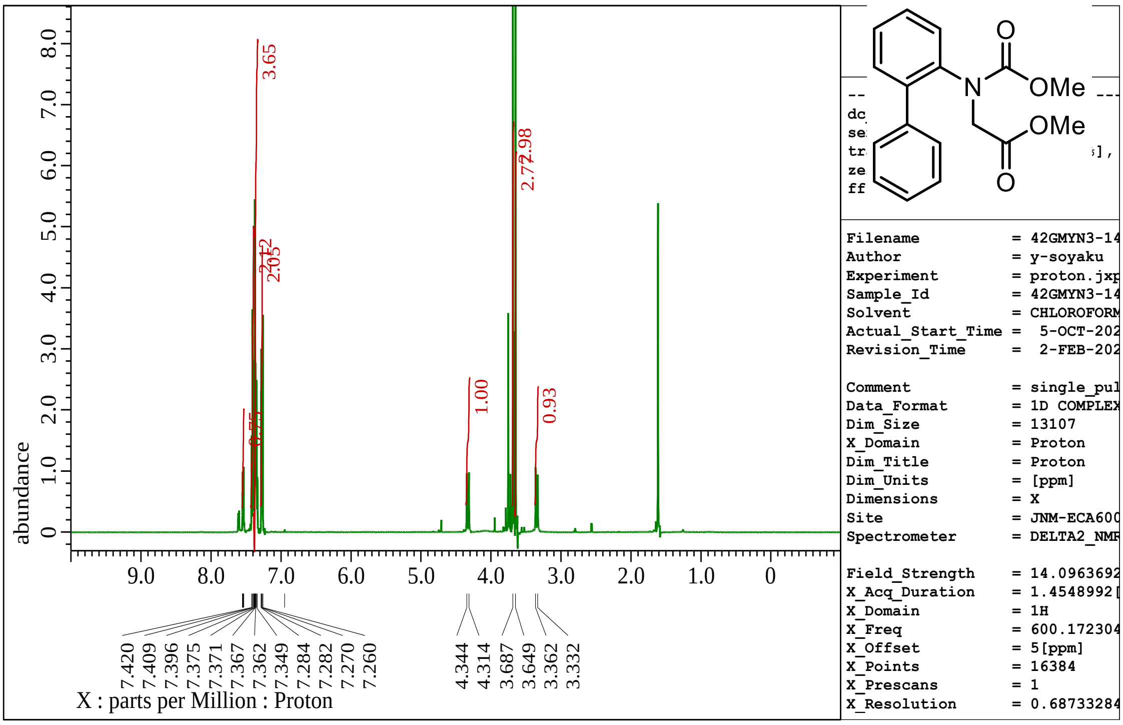

^{13}C{^{1}H }-NMR (150 MHz, $CDCl_3$) of **S2h**

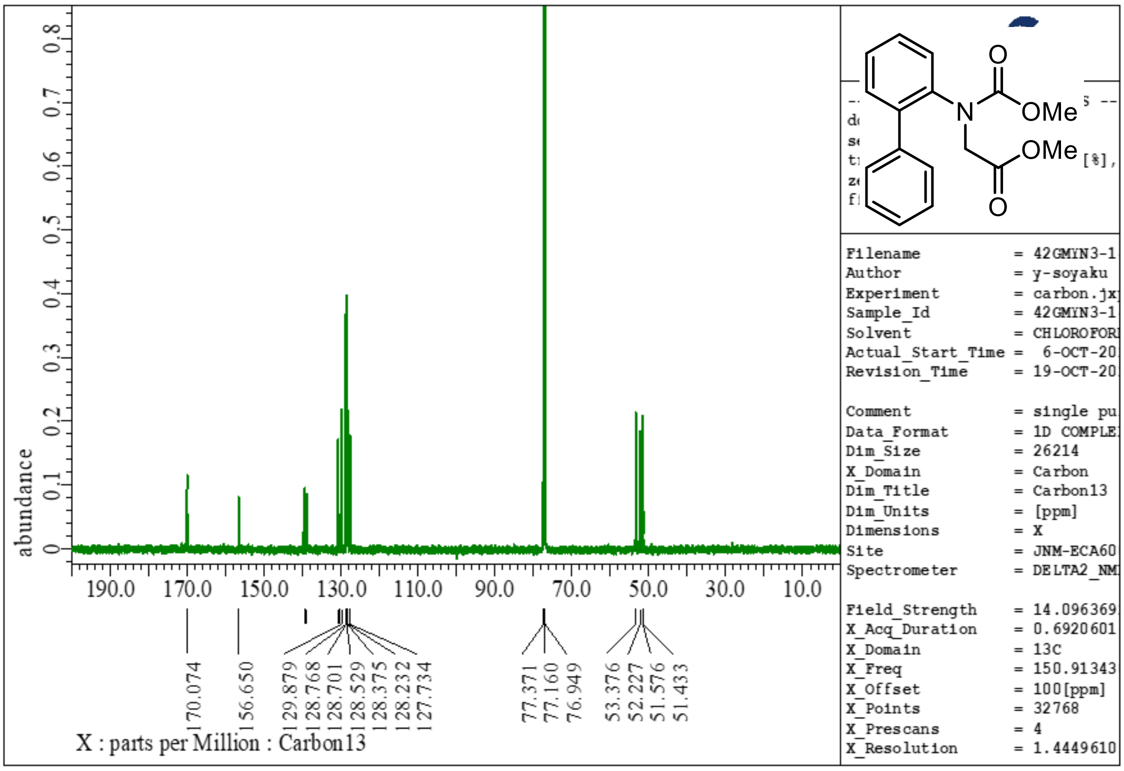

H-H COSY-NMR (600 MHz, $CDCl_3$) of **S2h**

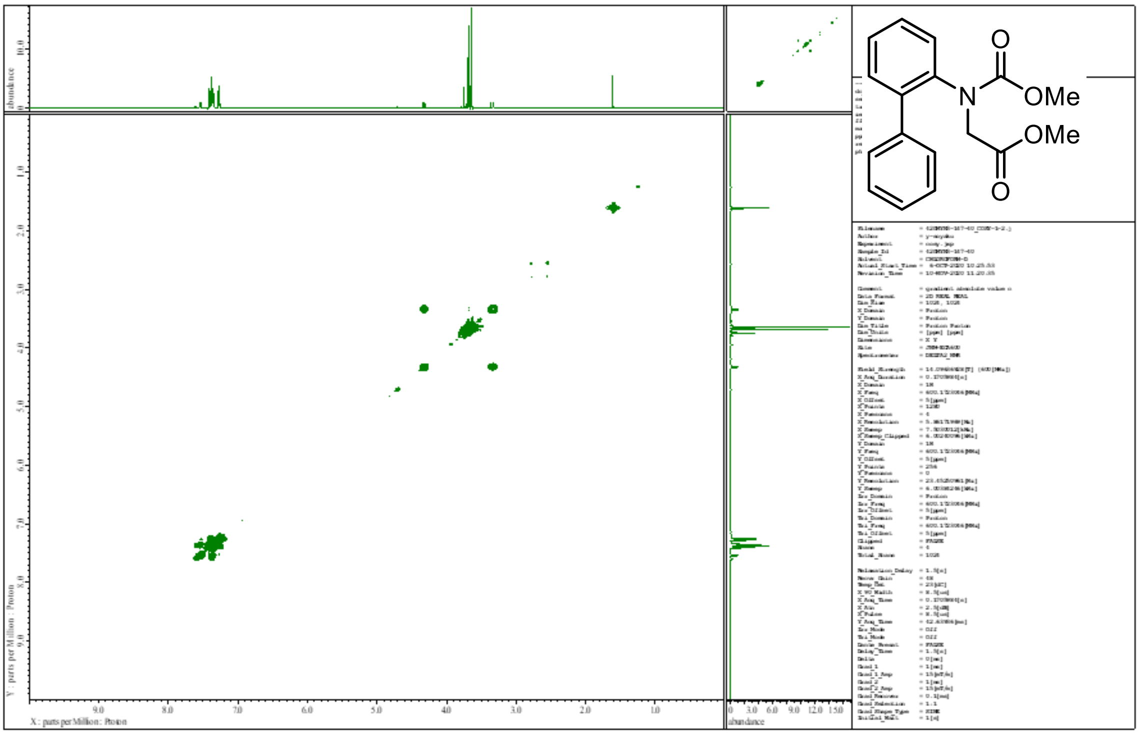

HMQC-NMR ($CDCl_3$) of **S2h**

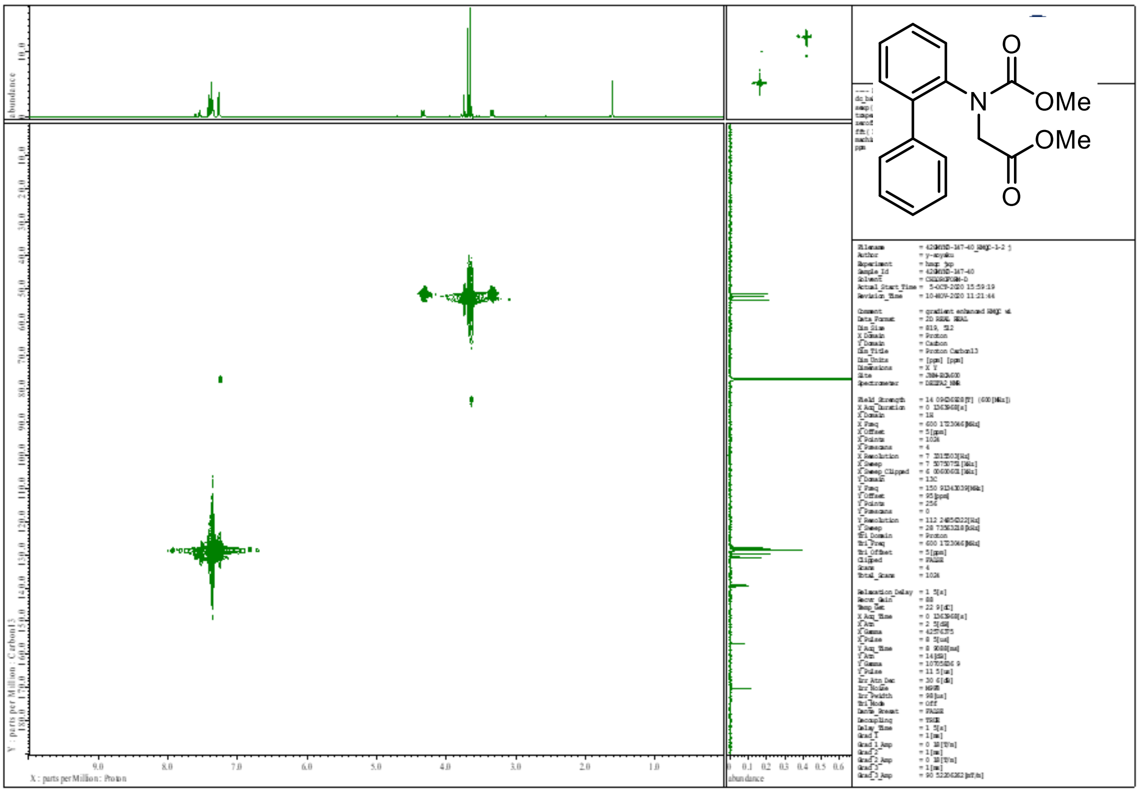

^{1}H-NMR (600 MHz, $CDCl_3$) of **S3**

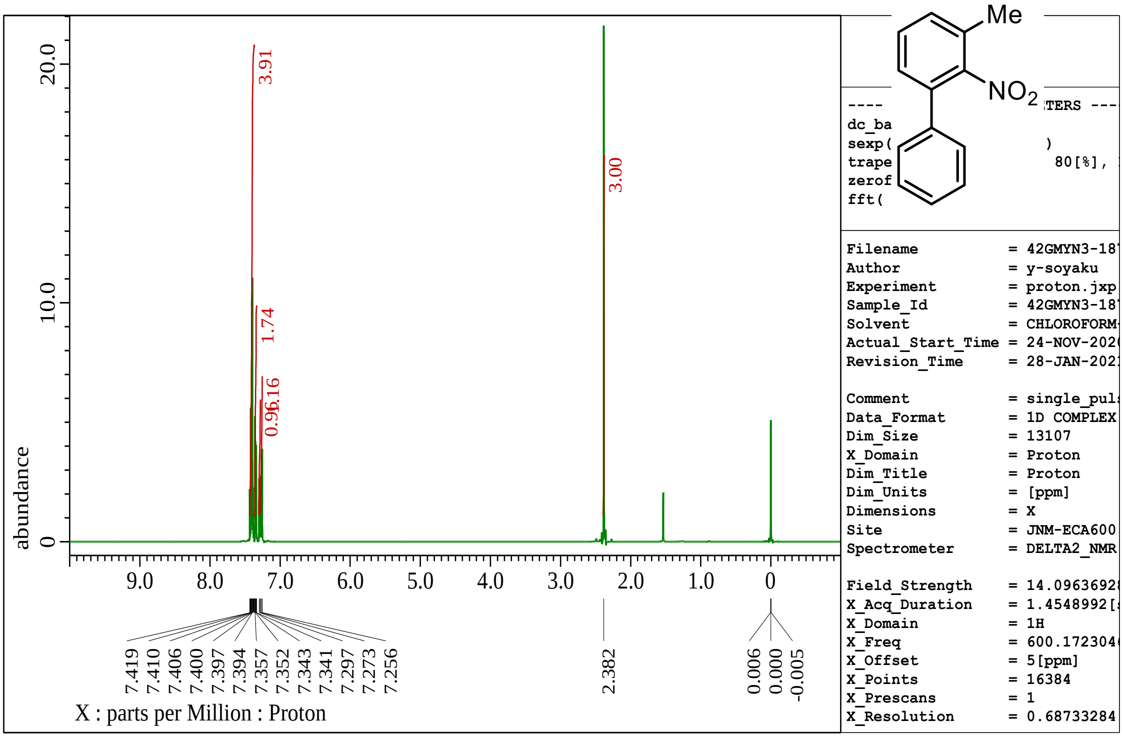

^{13}C{^{1}H }-NMR (150 MHz, $CDCl_3$) of **S3**

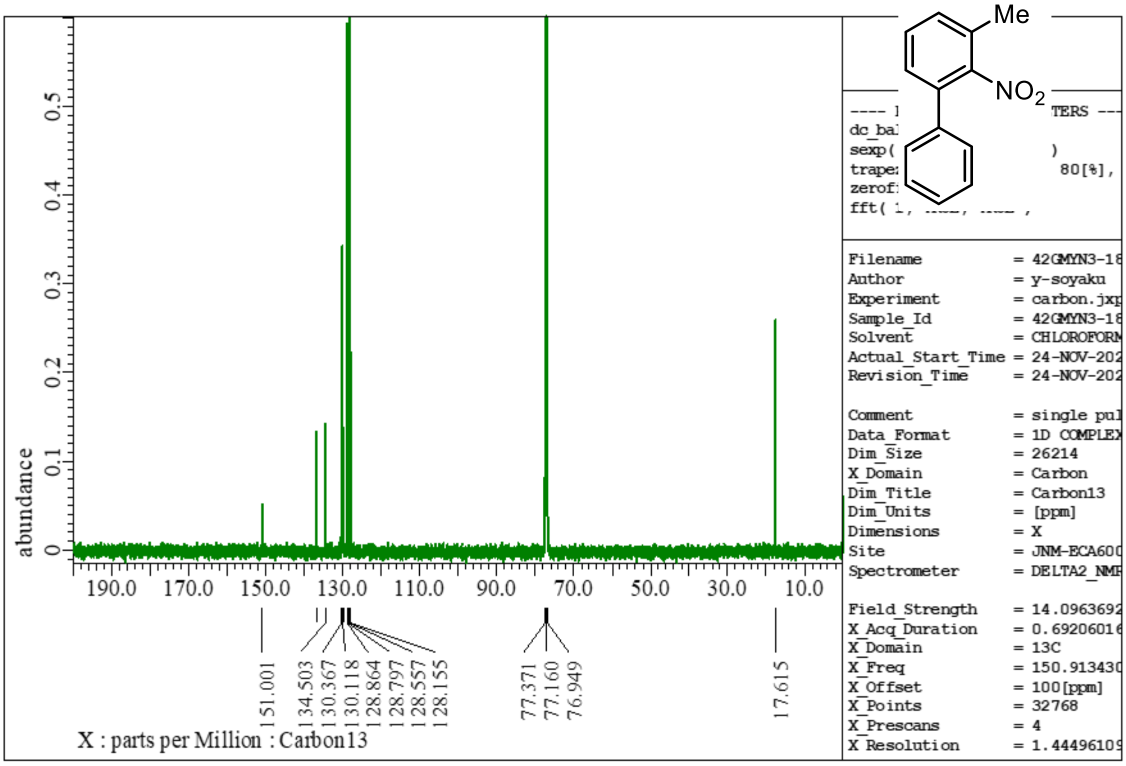

H-H COSY-NMR (600 MHz, $CDCl_3$) of **S3**

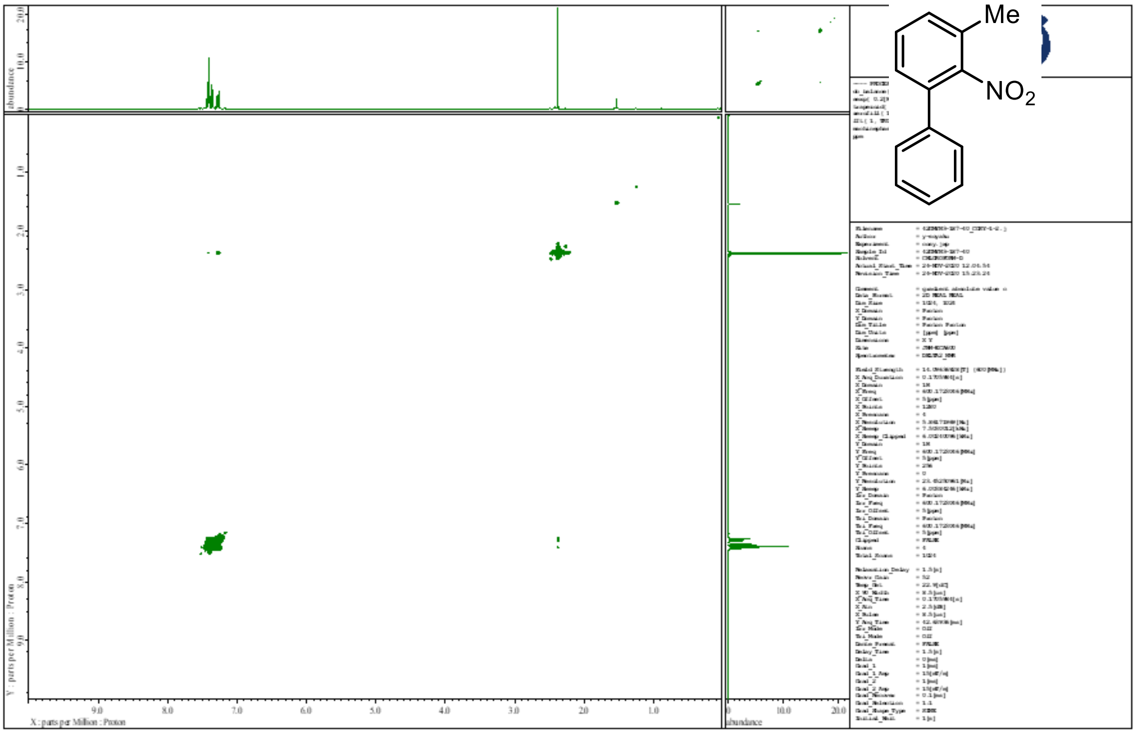

HMQC-NMR ($CDCl_3$) of **S3**

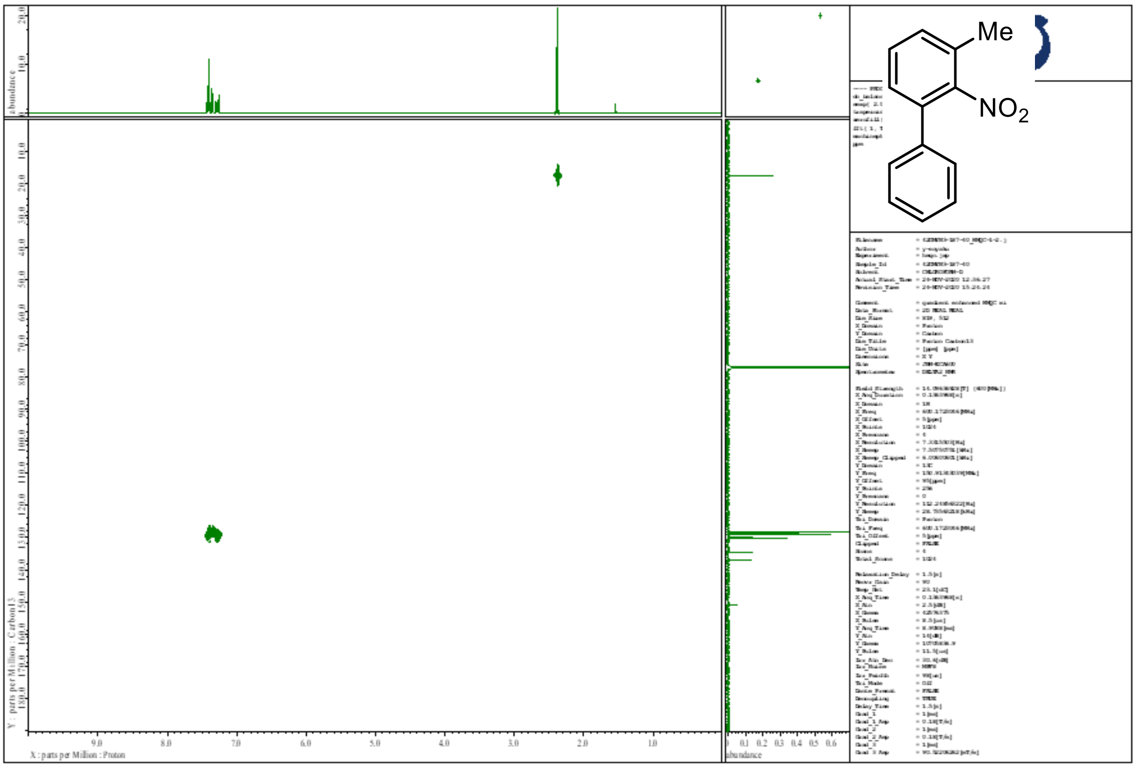

^{1}H-NMR (600 MHz, $CDCl_3$) of **S4**

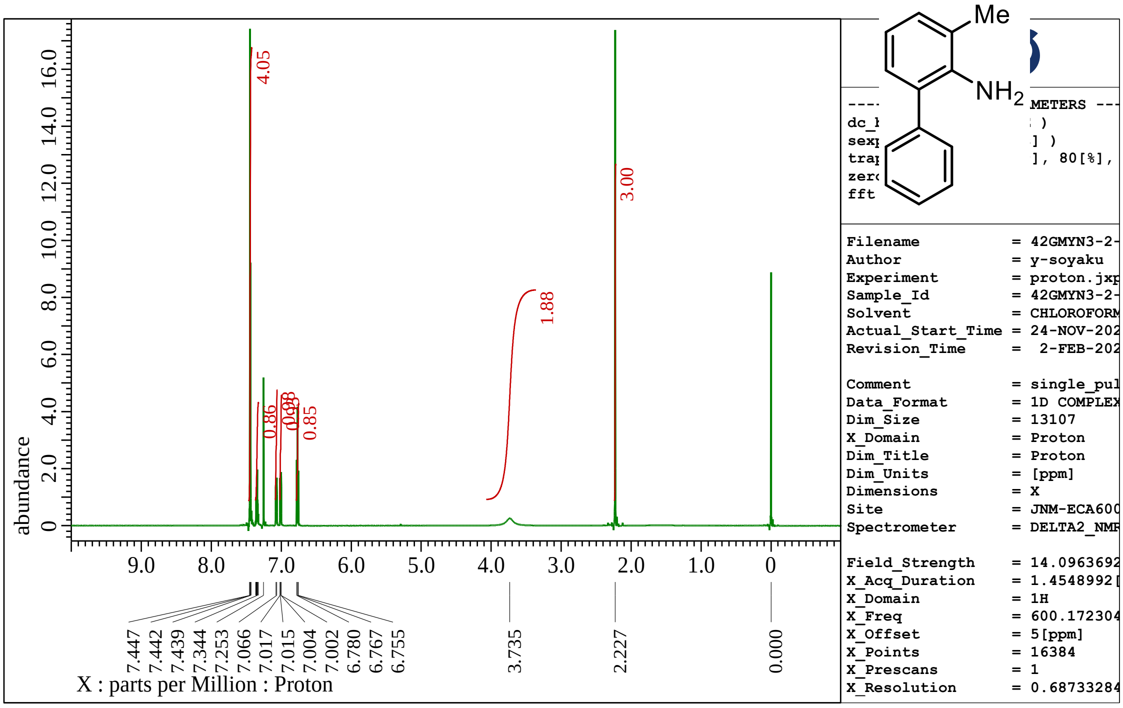

^{13}C{^{1}H }-NMR (150 MHz, $CDCl_3$) of **S4**

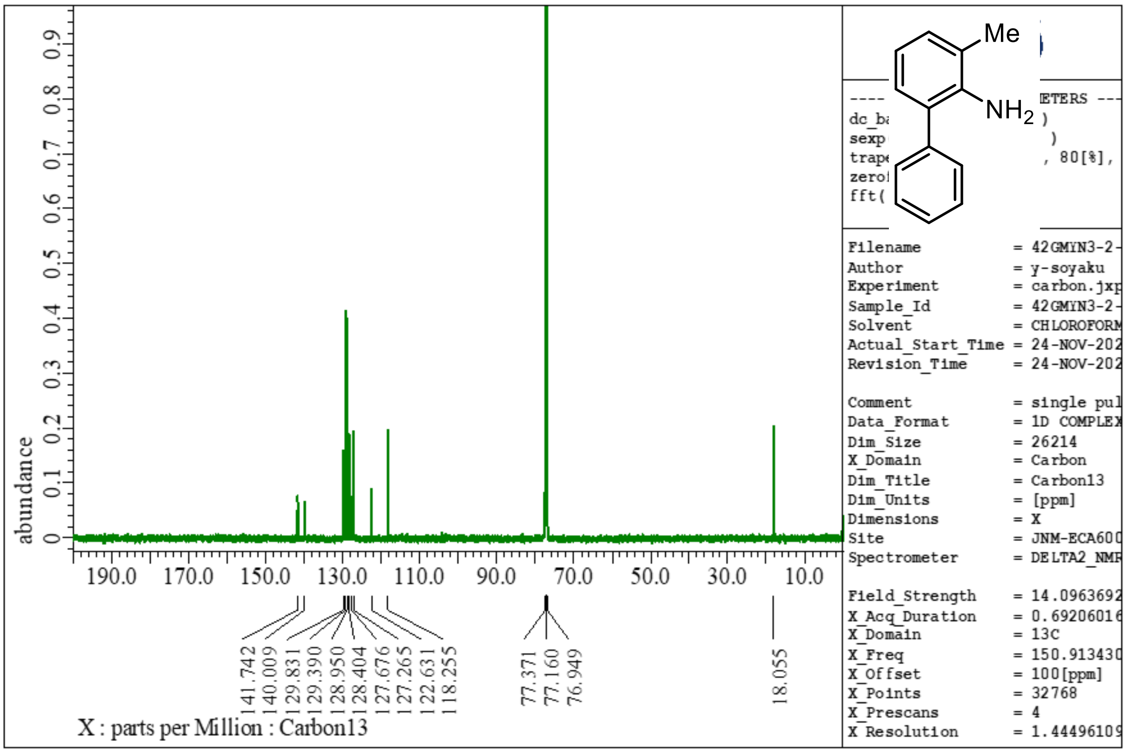

H-H COSY-NMR (600 MHz, $CDCl_3$) of **S4**

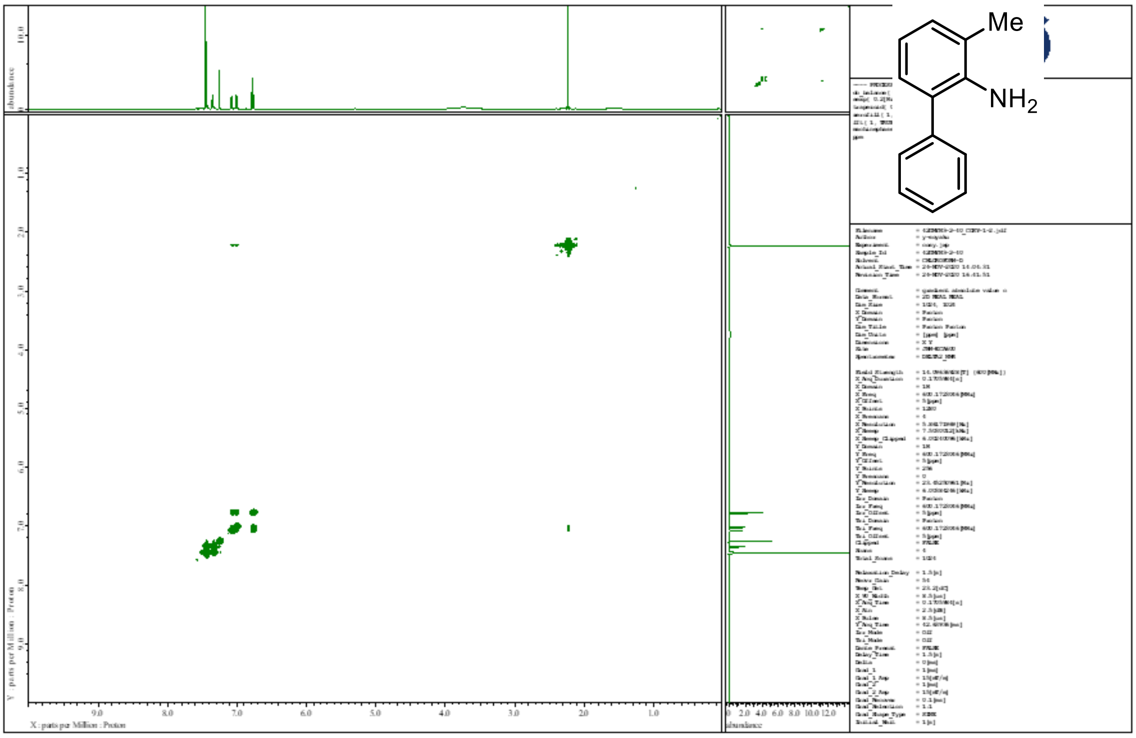

HMQC-NMR ($CDCl_3$) of **S4**

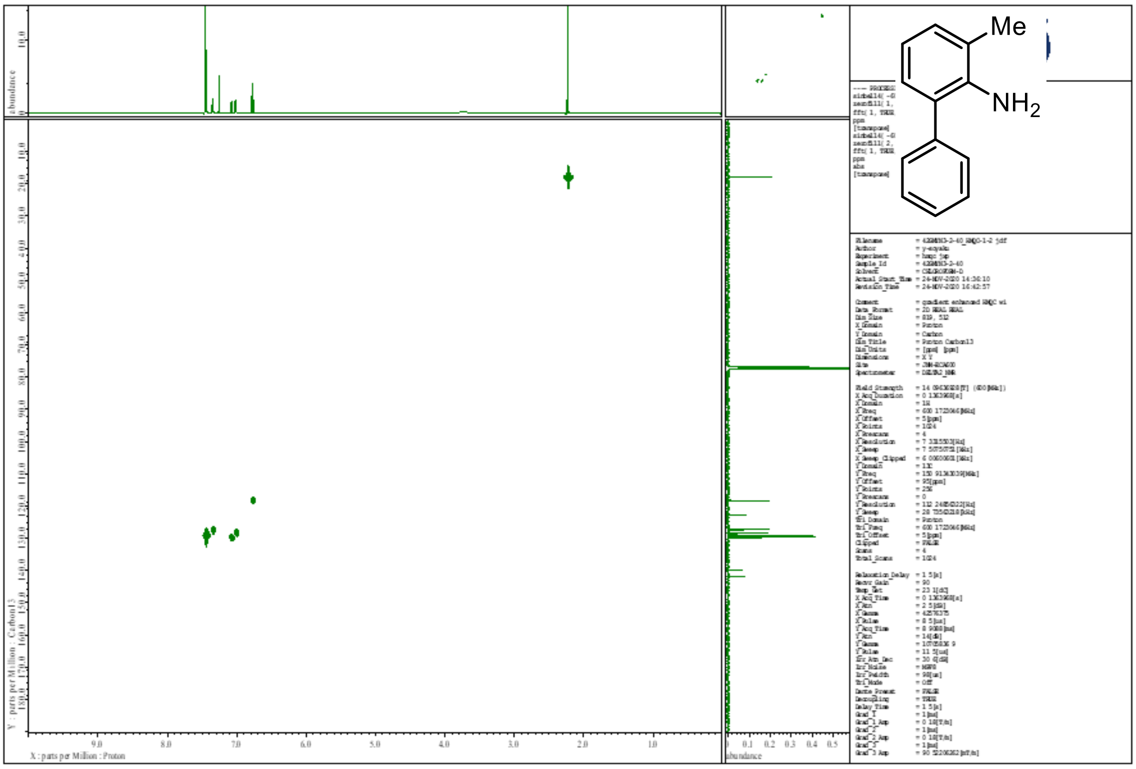

^{1}H-NMR (600 MHz, $CDCl_3$) of **S5g**

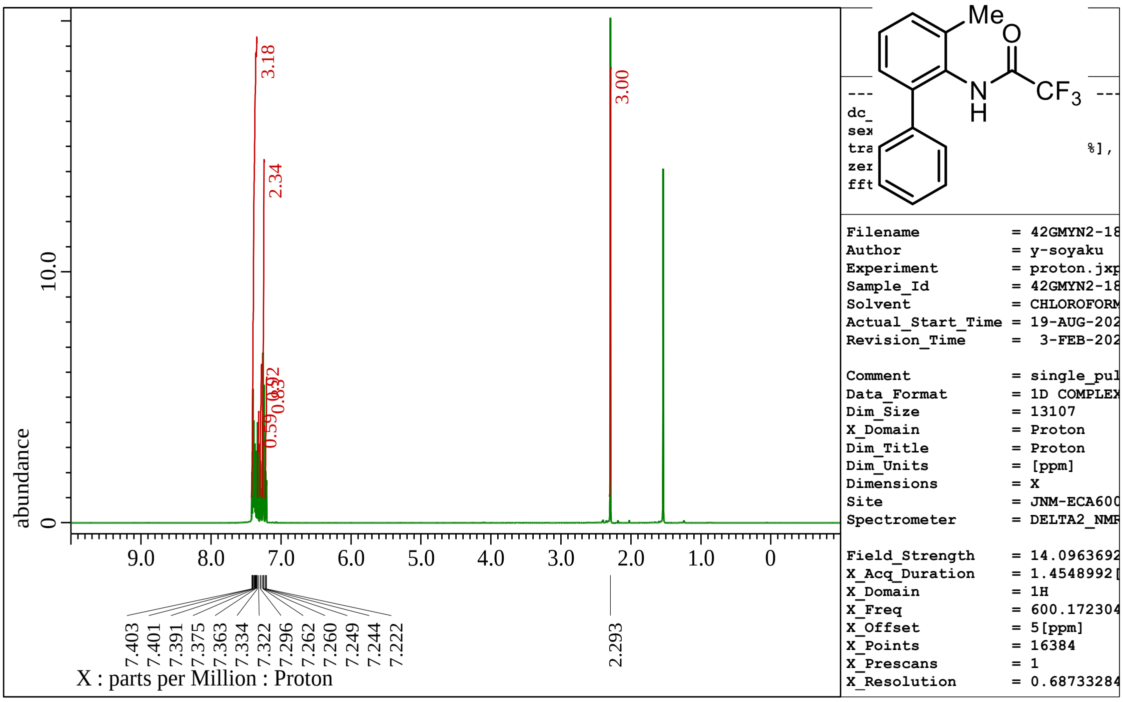

^{13}C{^{1}H}-NMR (150 MHz, $CDCl_3$) of **S5g**

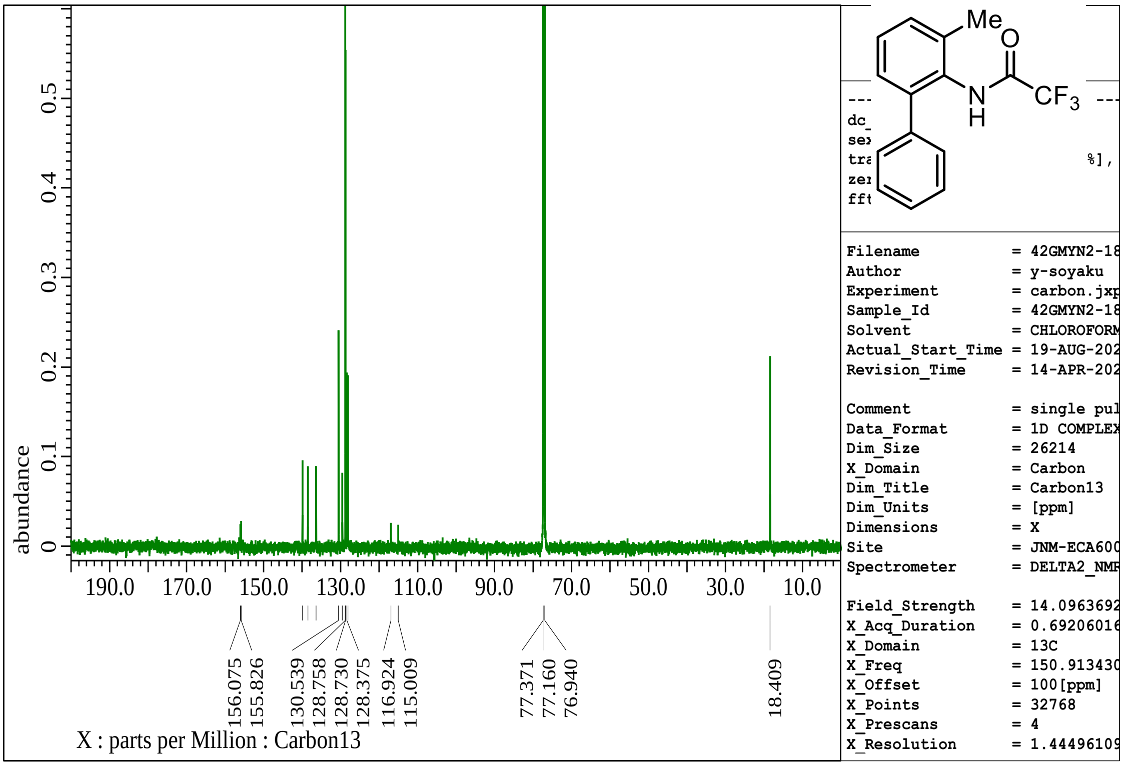

H-H COSY-NMR (600 MHz, $CDCl_3$) of **S5g**

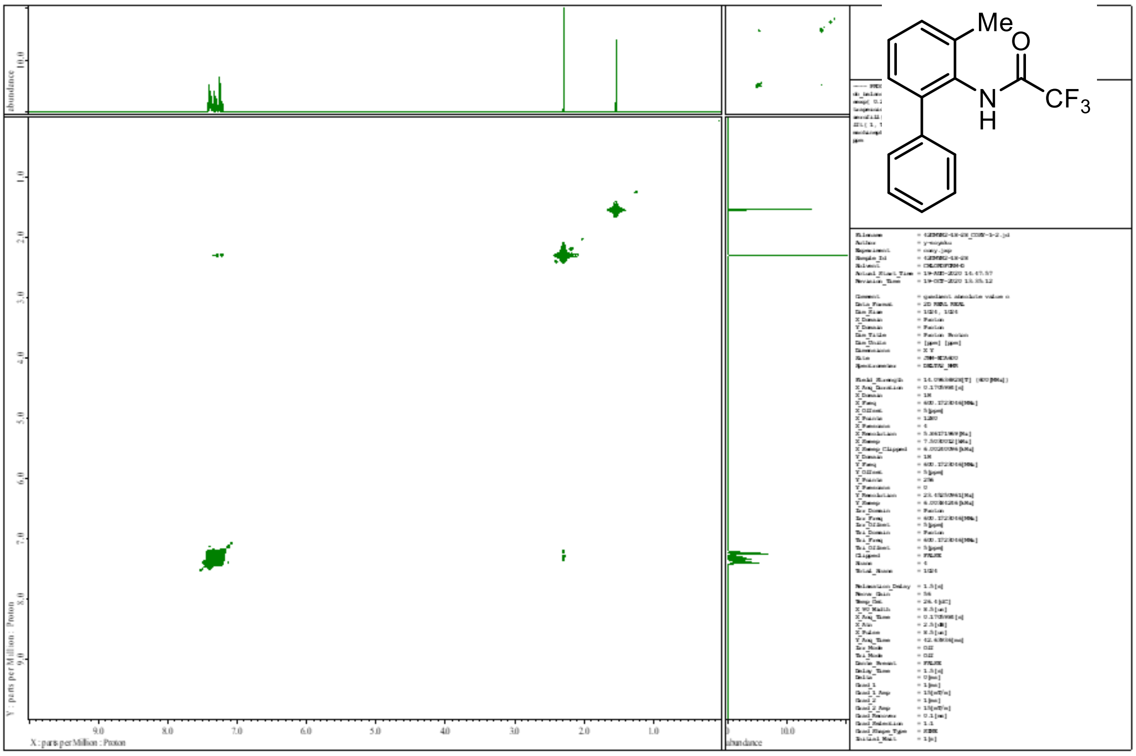

HMQC-NMR ($CDCl_3$) of **S5g**

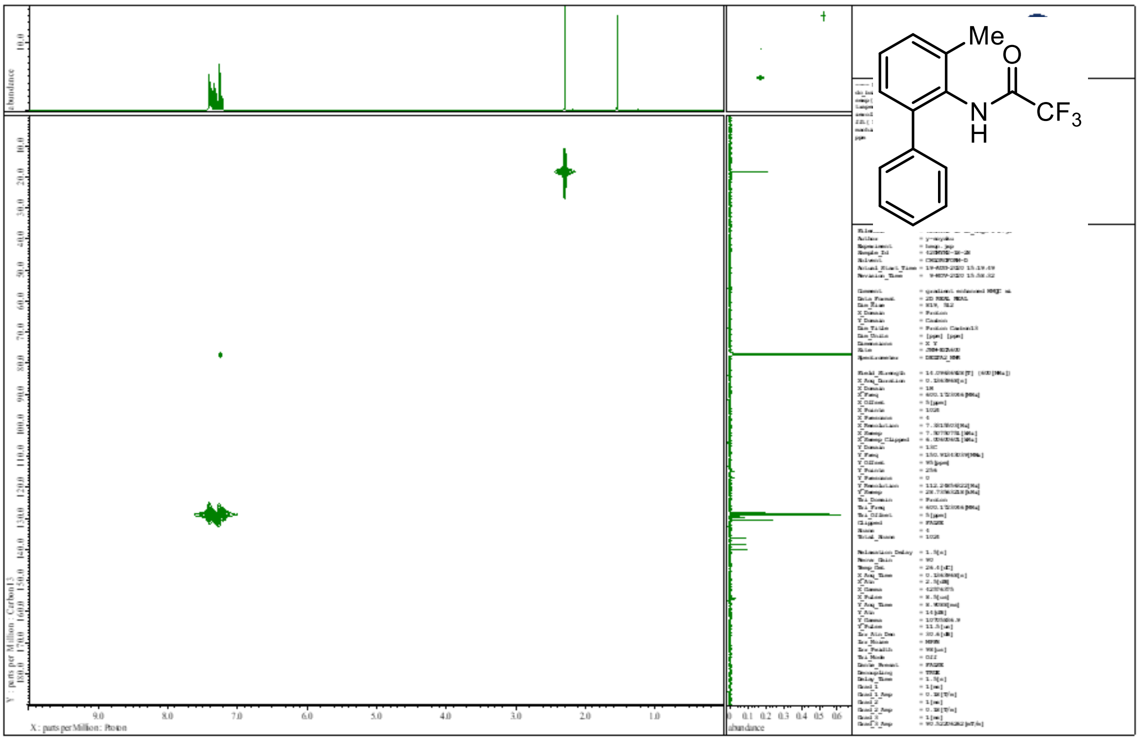

^{1}H-NMR (600 MHz, $CDCl_3$) of **S5h**

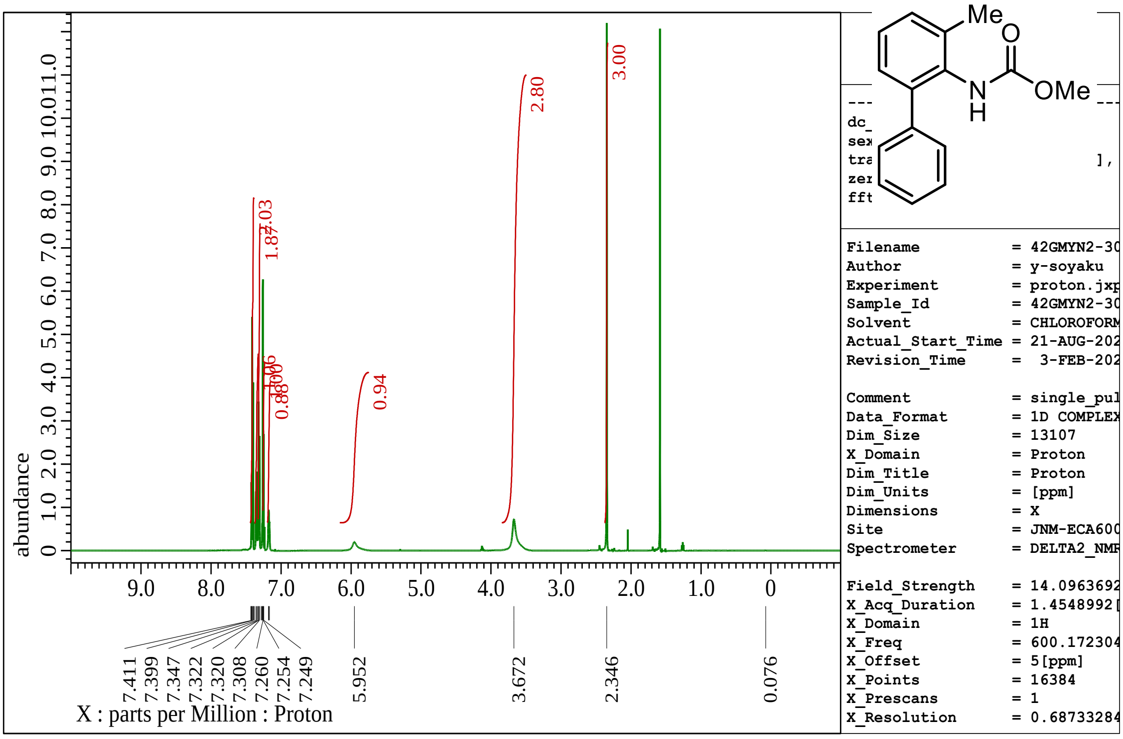

^{13}C{^{1}H }-NMR (150 MHz, $CDCl_3$) of **S5h**

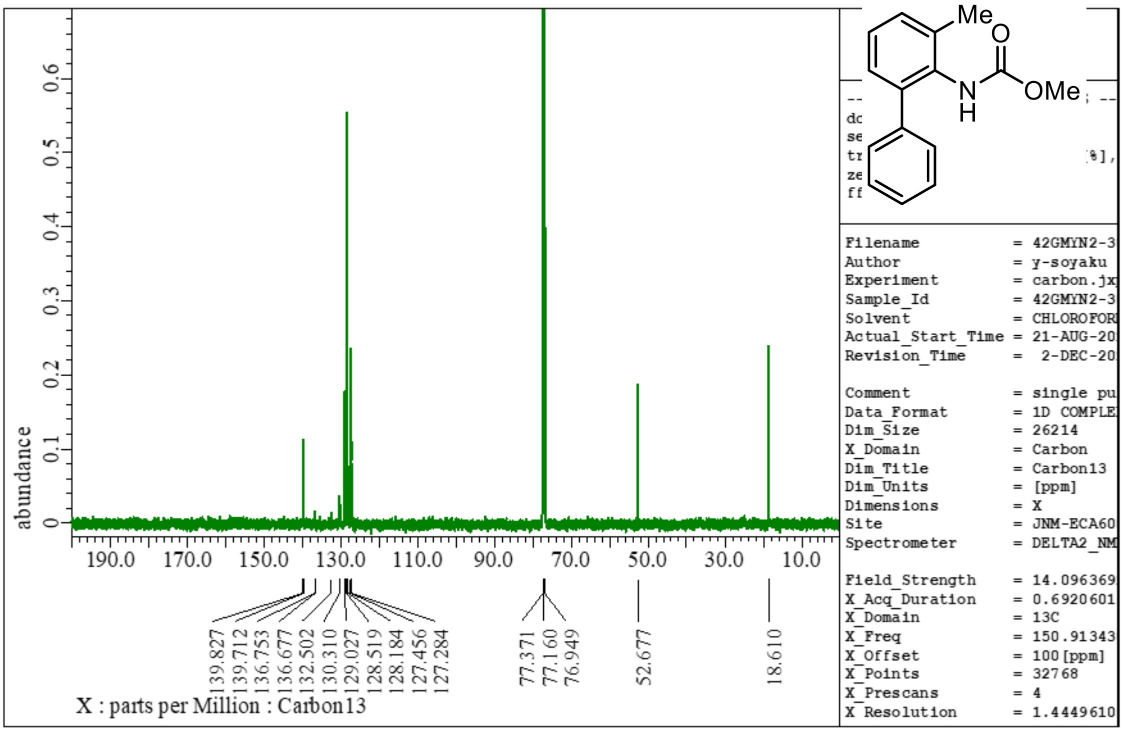

H-H COSY-NMR (600 MHz, $CDCl_3$) of **S5h**

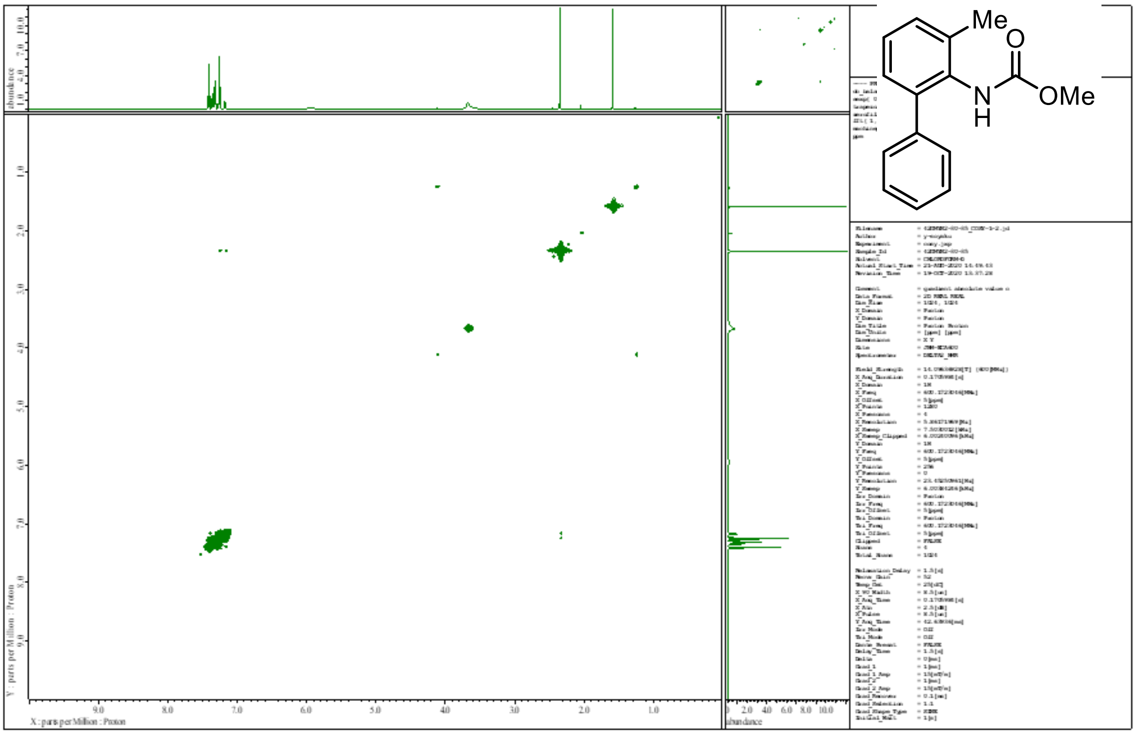

HMQC-NMR ($CDCl_3$) of **S5h**

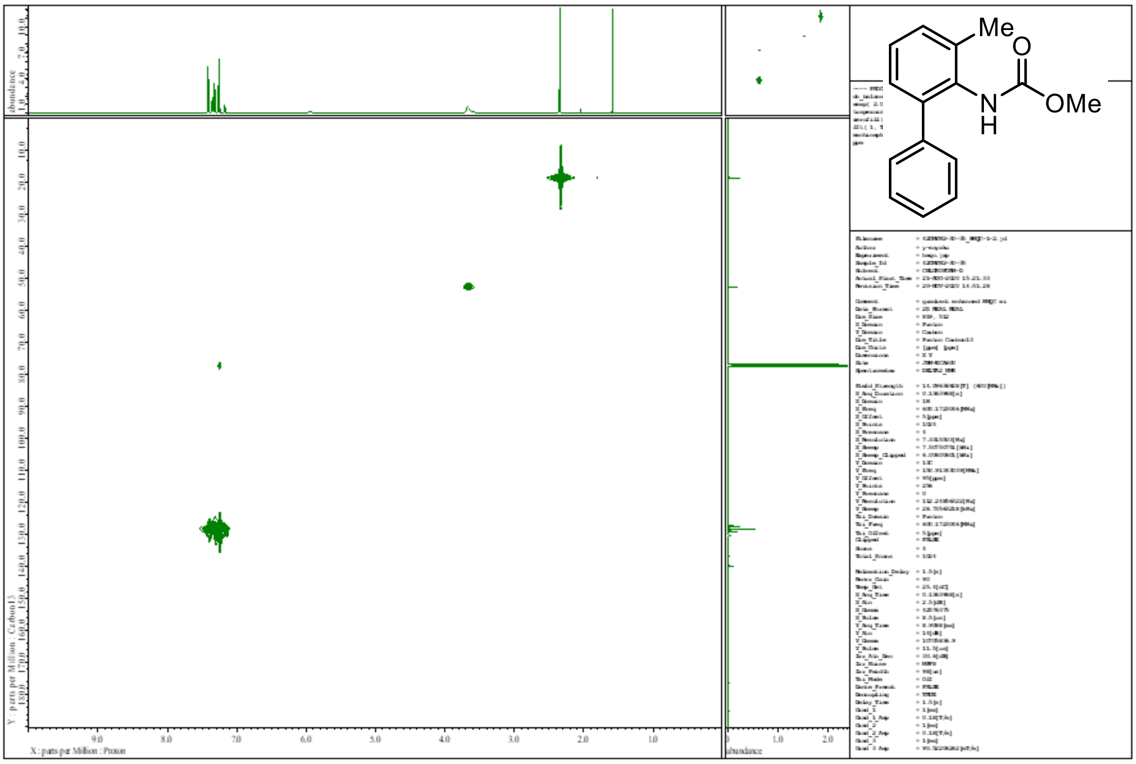

^{1}H-NMR (600 MHz, $CDCl_3$) of **S6h**

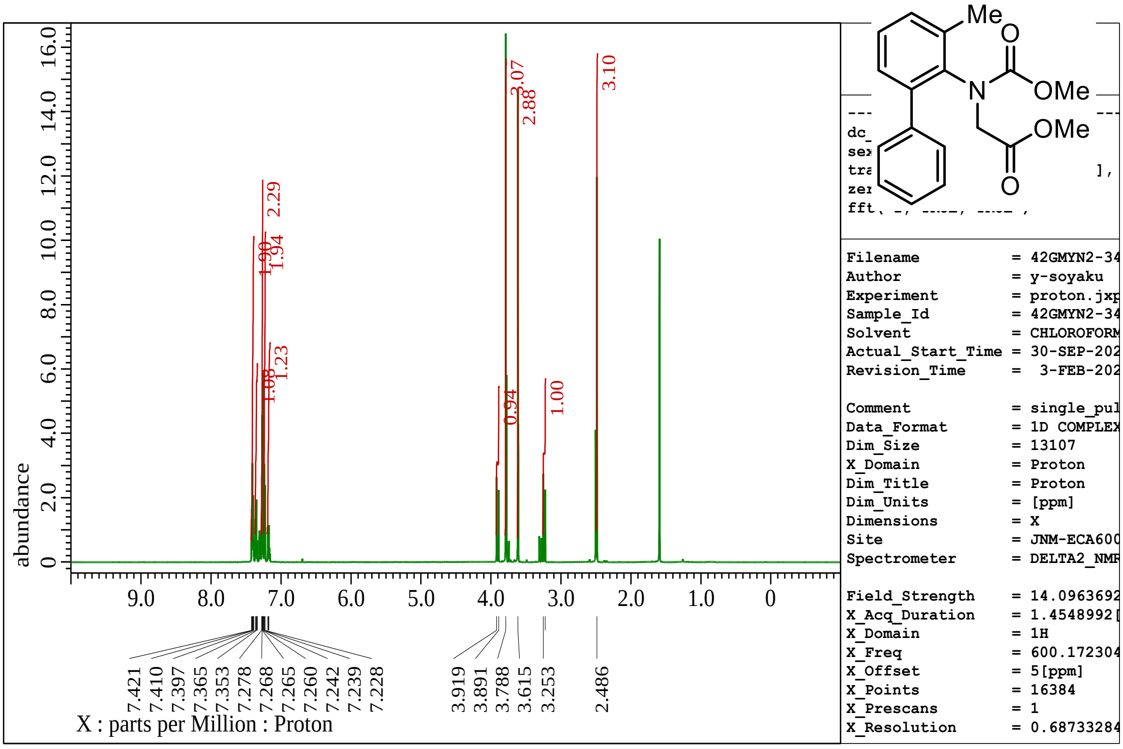

^{13}C{^{1}H }-NMR (150 MHz, $CDCl_3$) of **S6h**

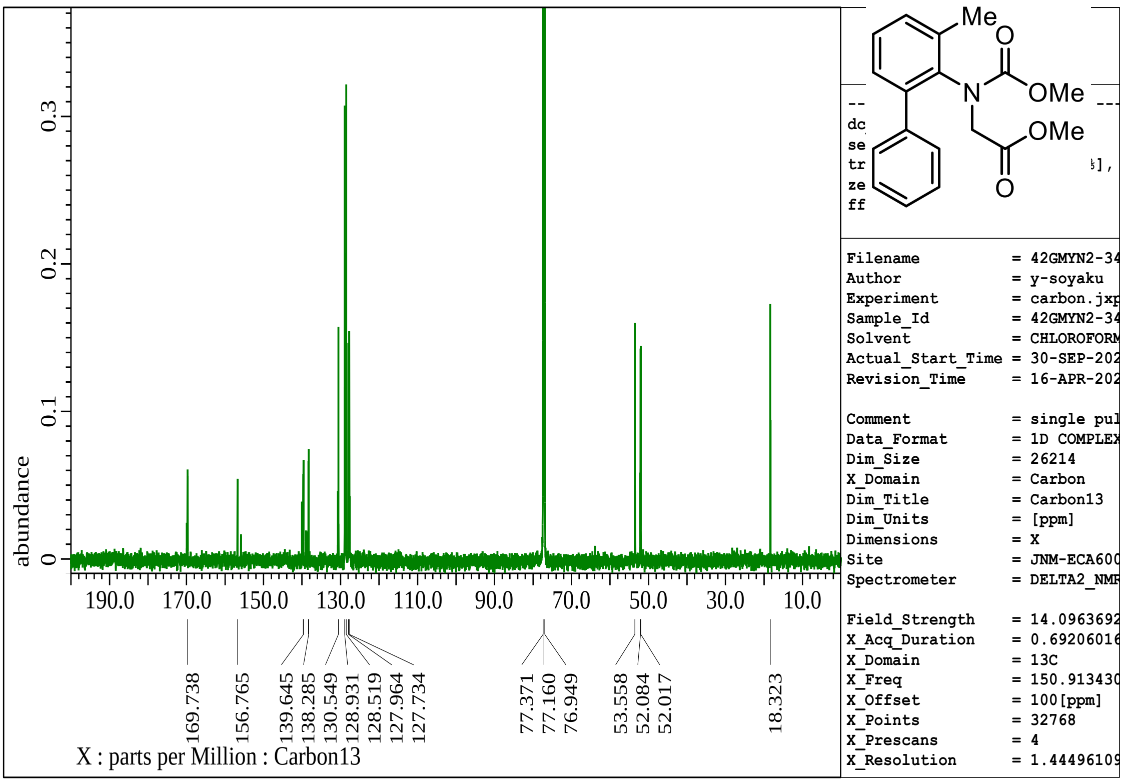

H-H COSY-NMR (600 MHz, $CDCl_3$) of **S6h**

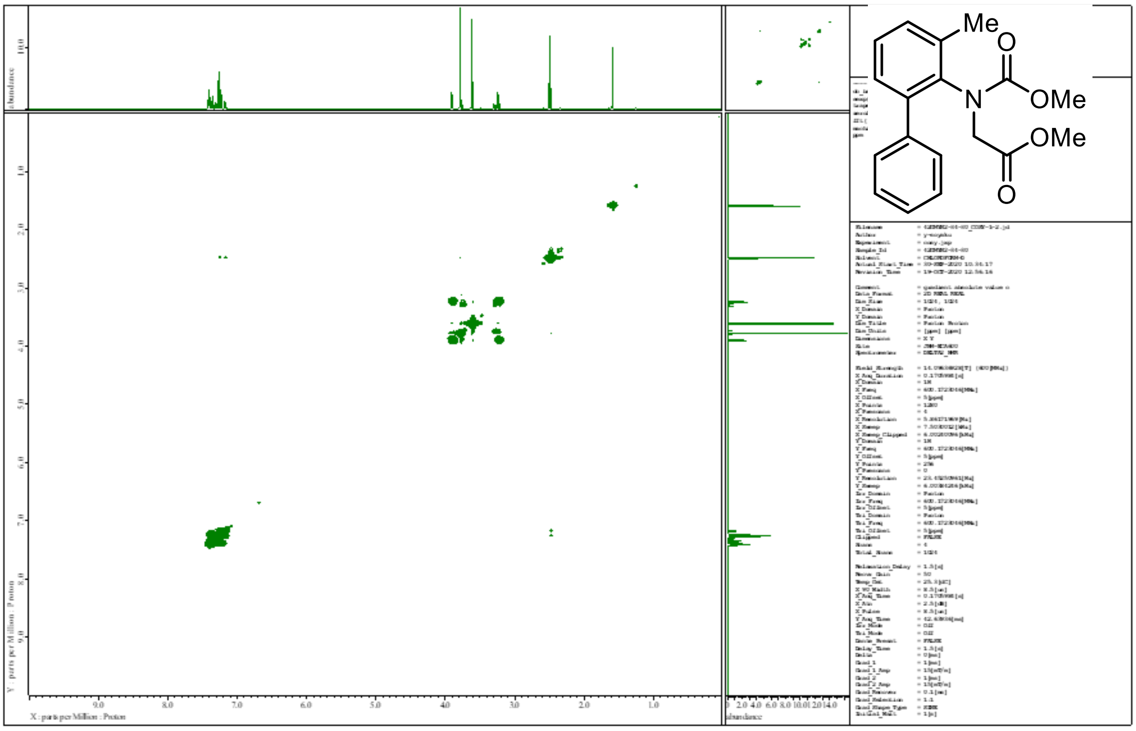

HMQC-NMR ($CDCl_3$) of **S6h**

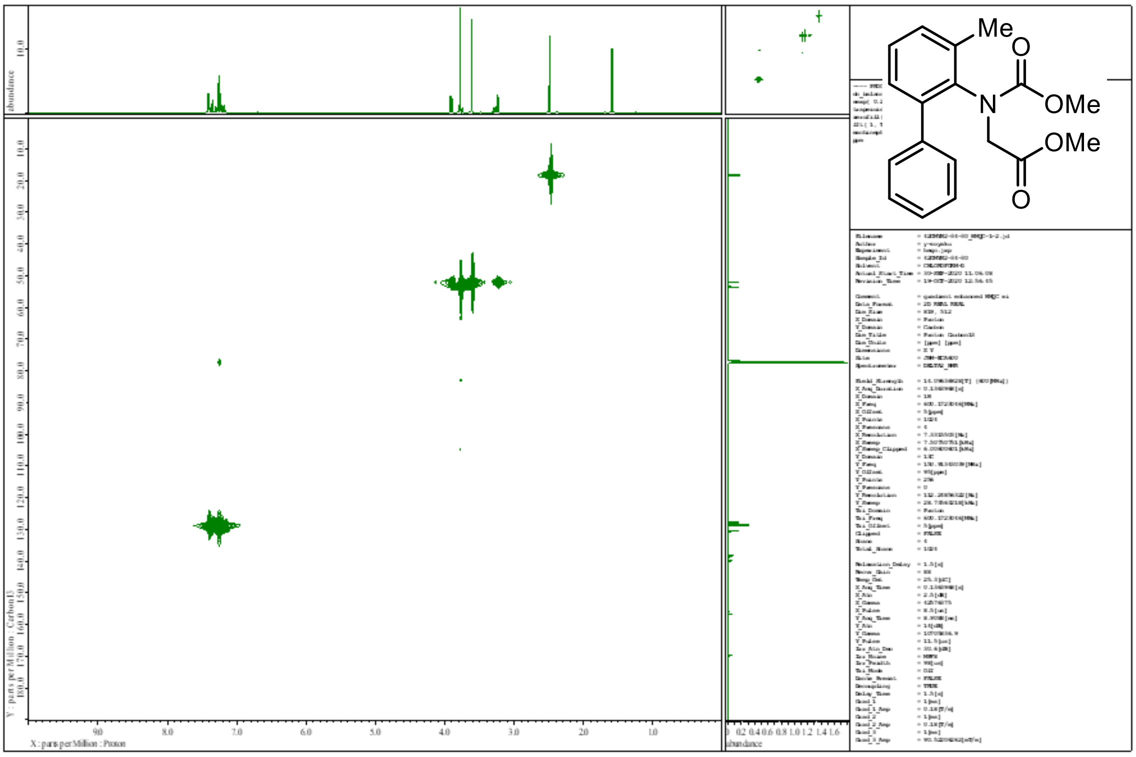

^{1}H-NMR (600 MHz, $CDCl_3$) of **S7c**

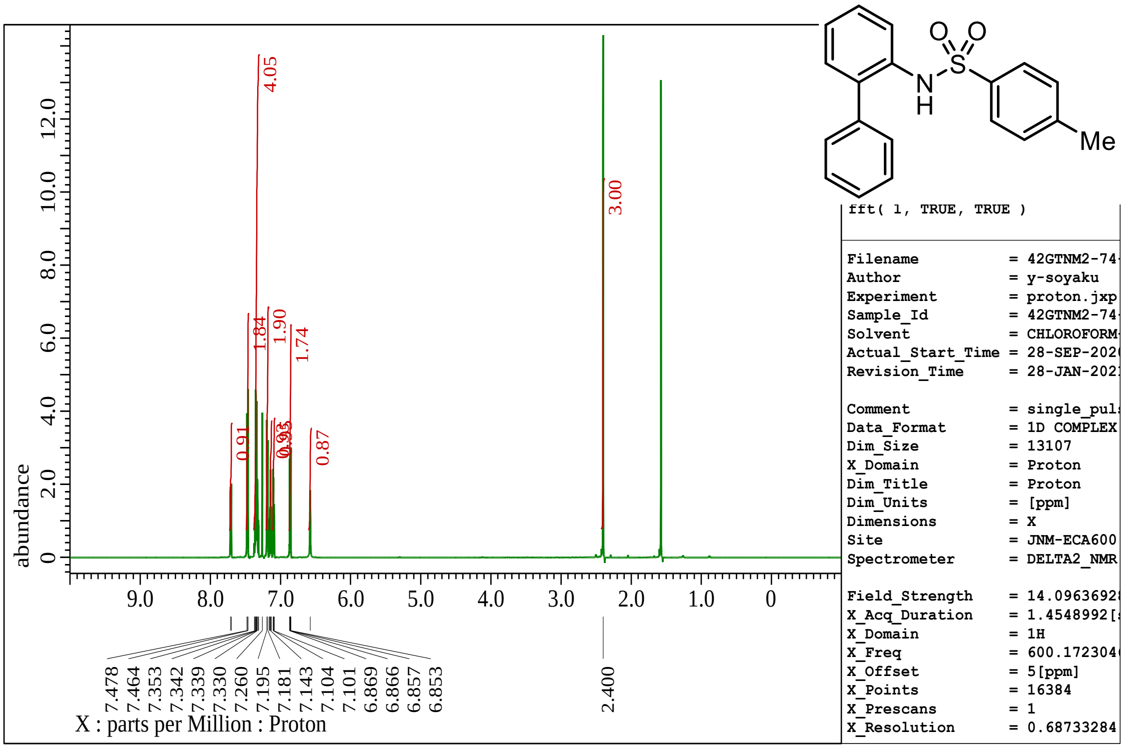

^{13}C{^{1}H }-NMR (150 MHz, $CDCl_3$) of **S7c**

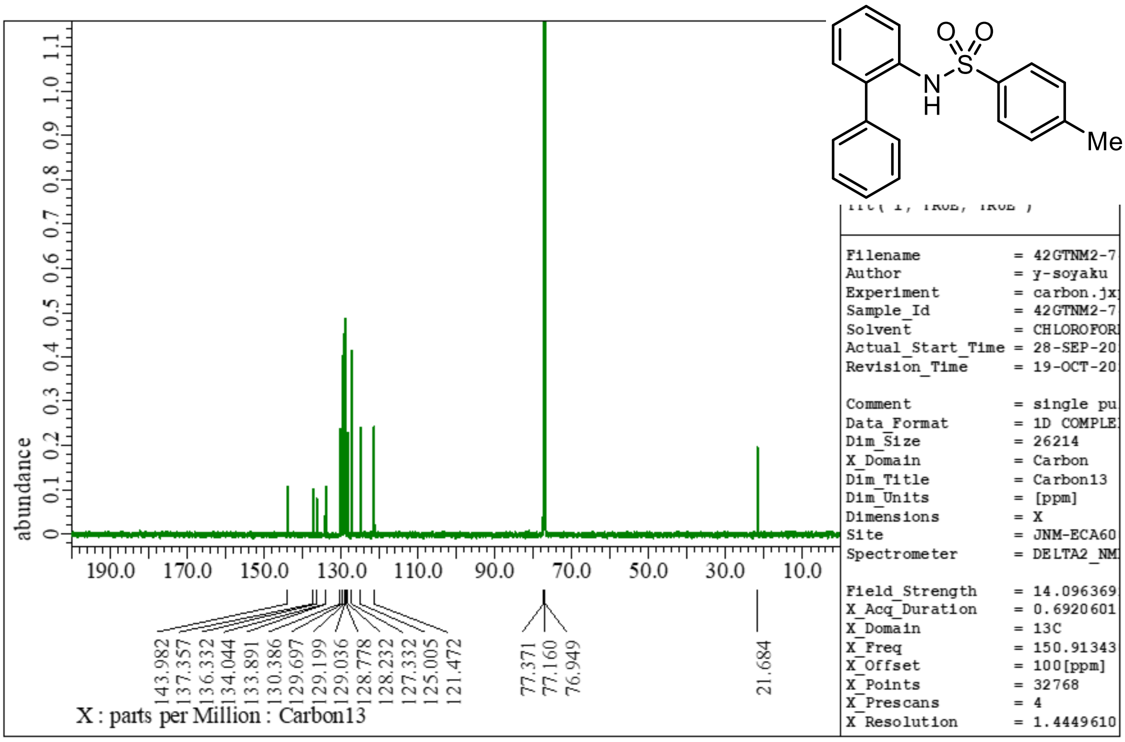

H-H COSY-NMR (600 MHz, $CDCl_3$) of **S7c**

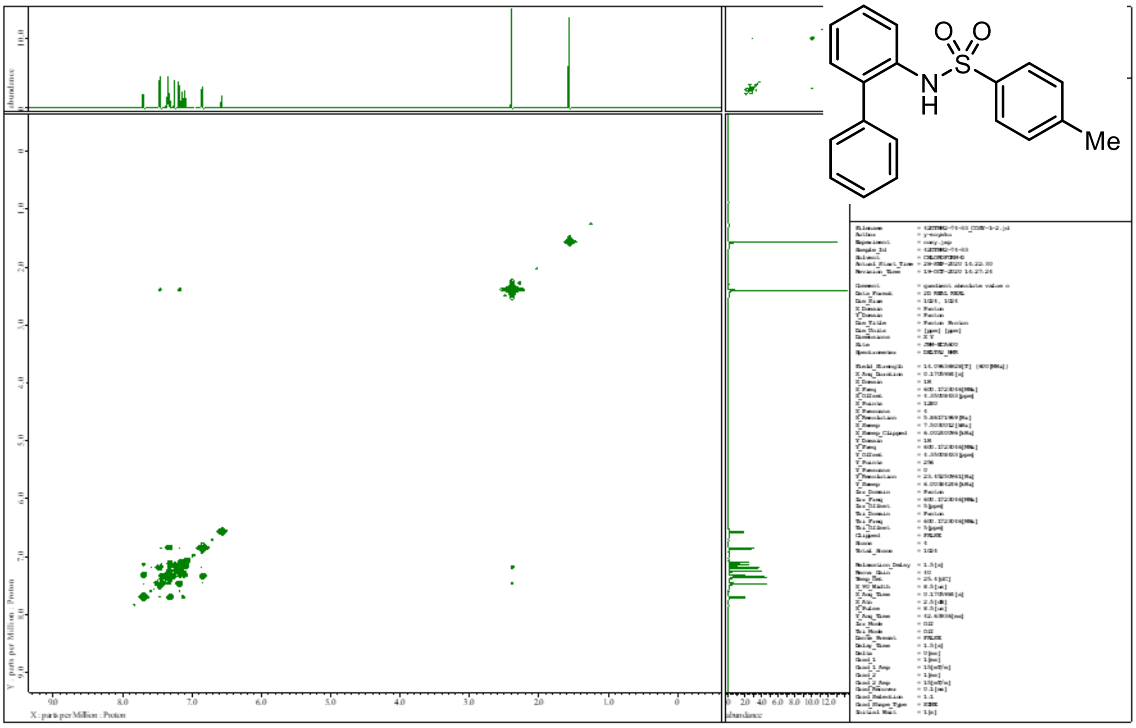

HMQC-NMR ($CDCl_3$) of **S7c**

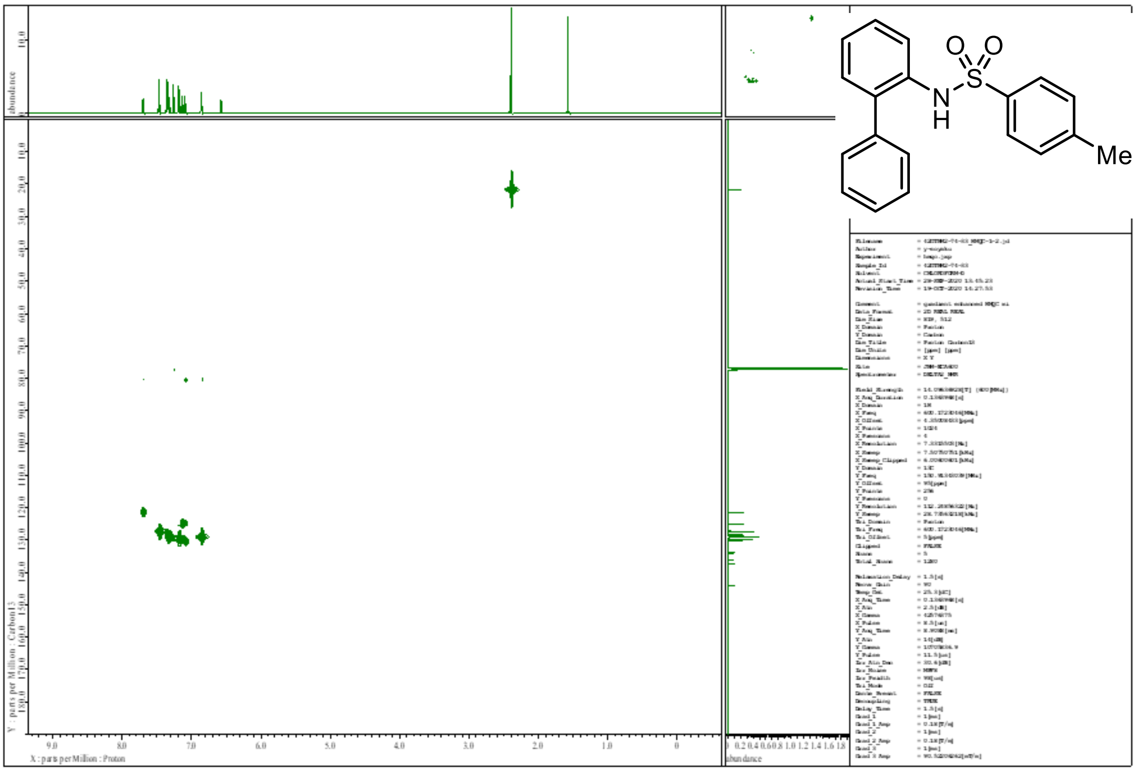

^{1}H-NMR (600 MHz, $CDCl_3$) of **S7d**

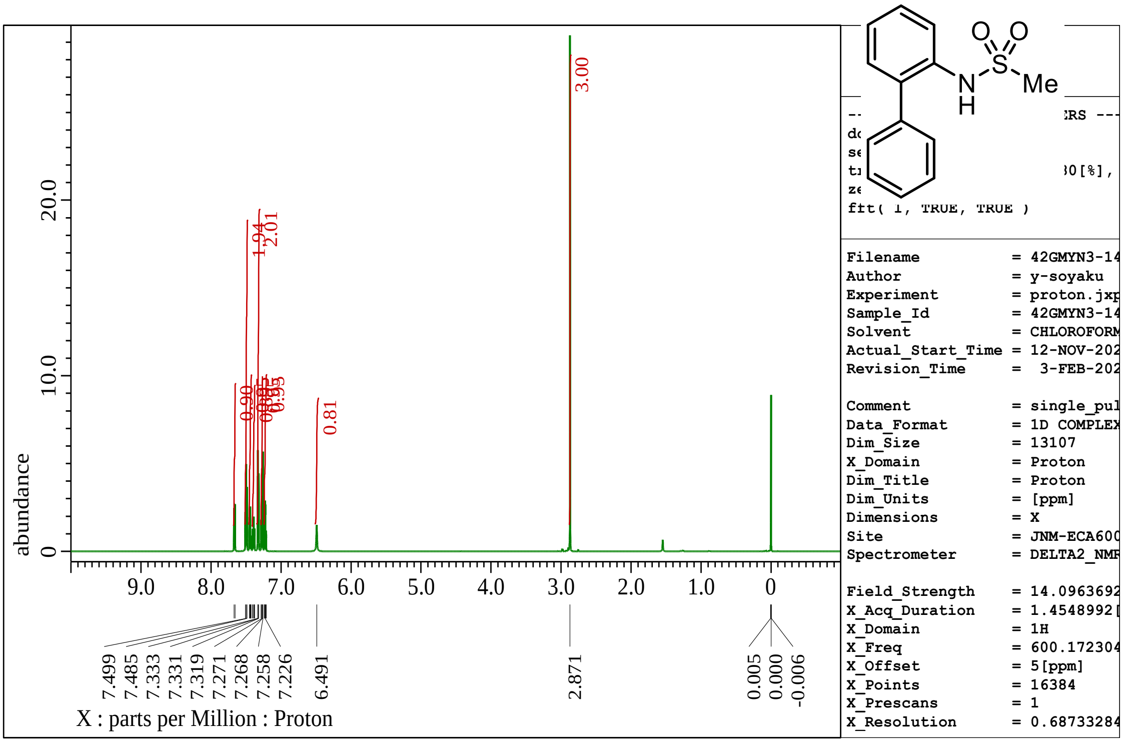

^{13}C{^{1}H }-NMR (150 MHz, $CDCl_3$) of **S7d**

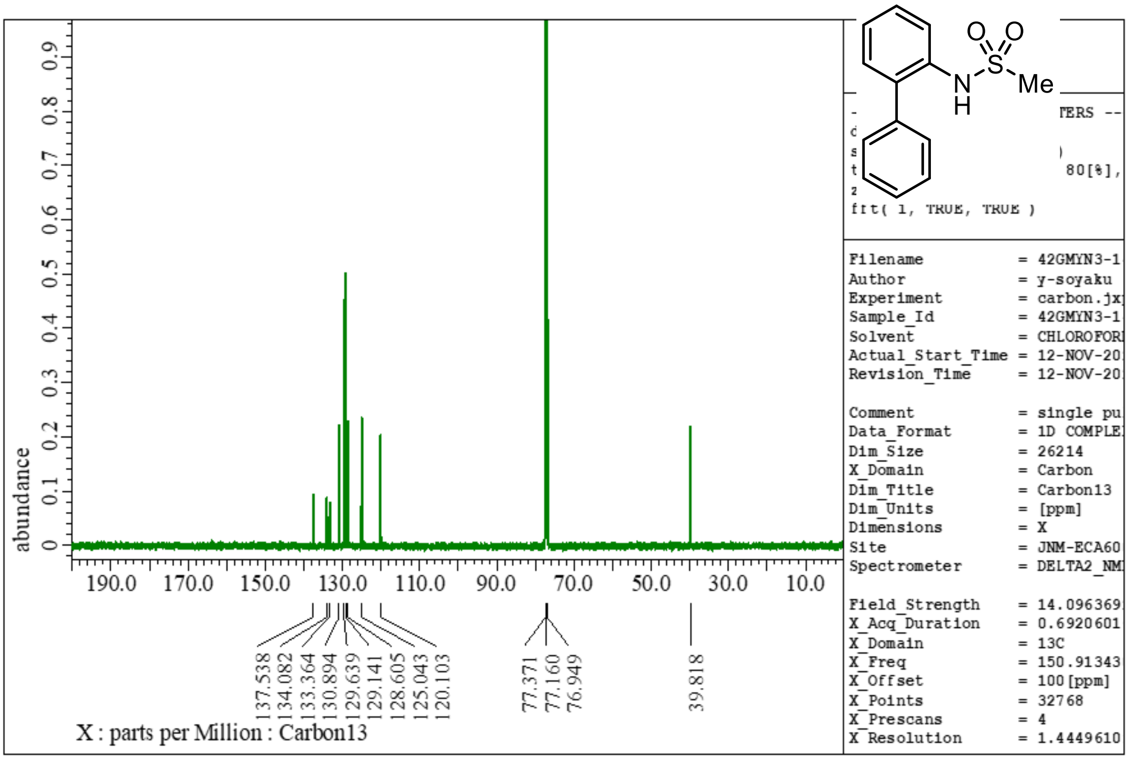

H-H COSY-NMR (600 MHz, $CDCl_3$) of **S7d**

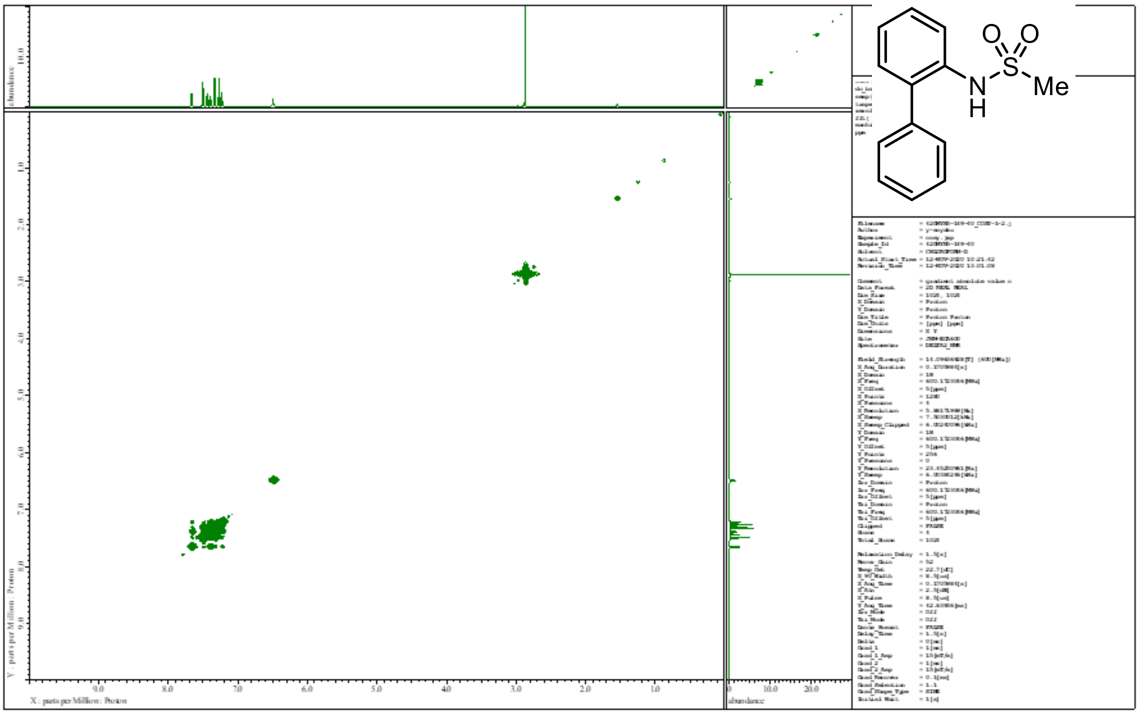

HMQC-NMR ($CDCl_3$) of **S7d**

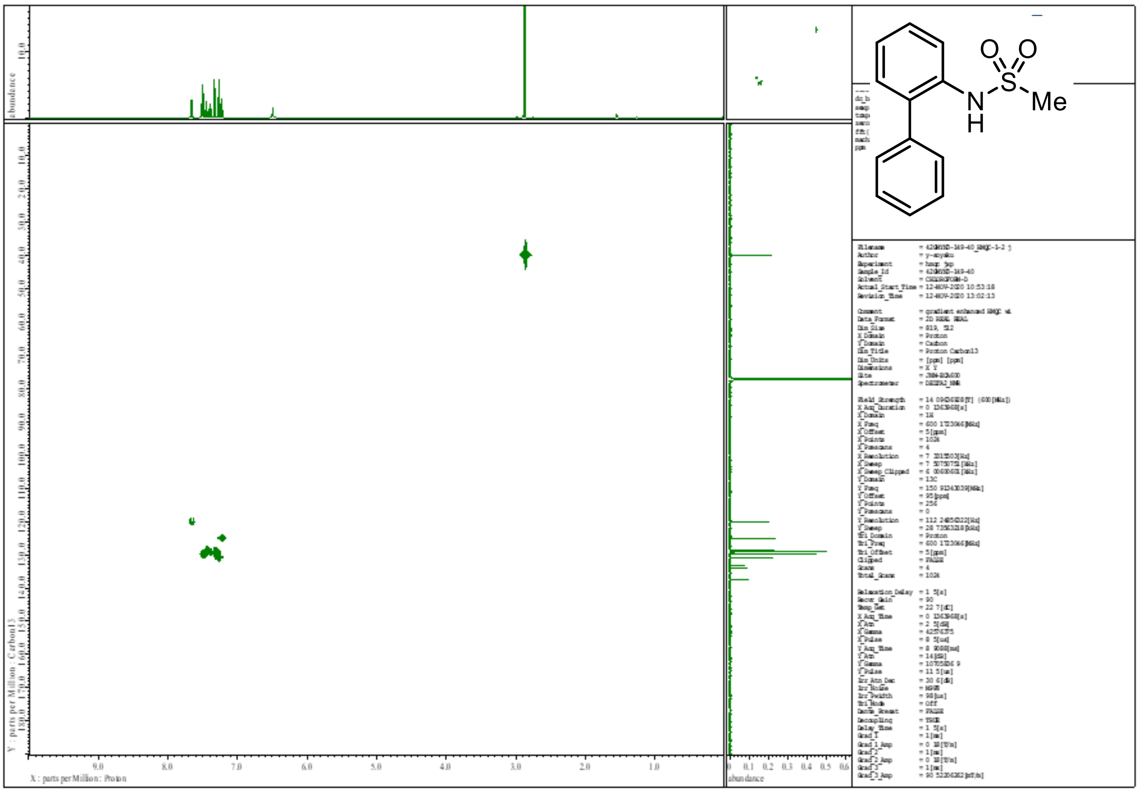

^{1}H-NMR (600 MHz, $CDCl_3$) of **S7e**

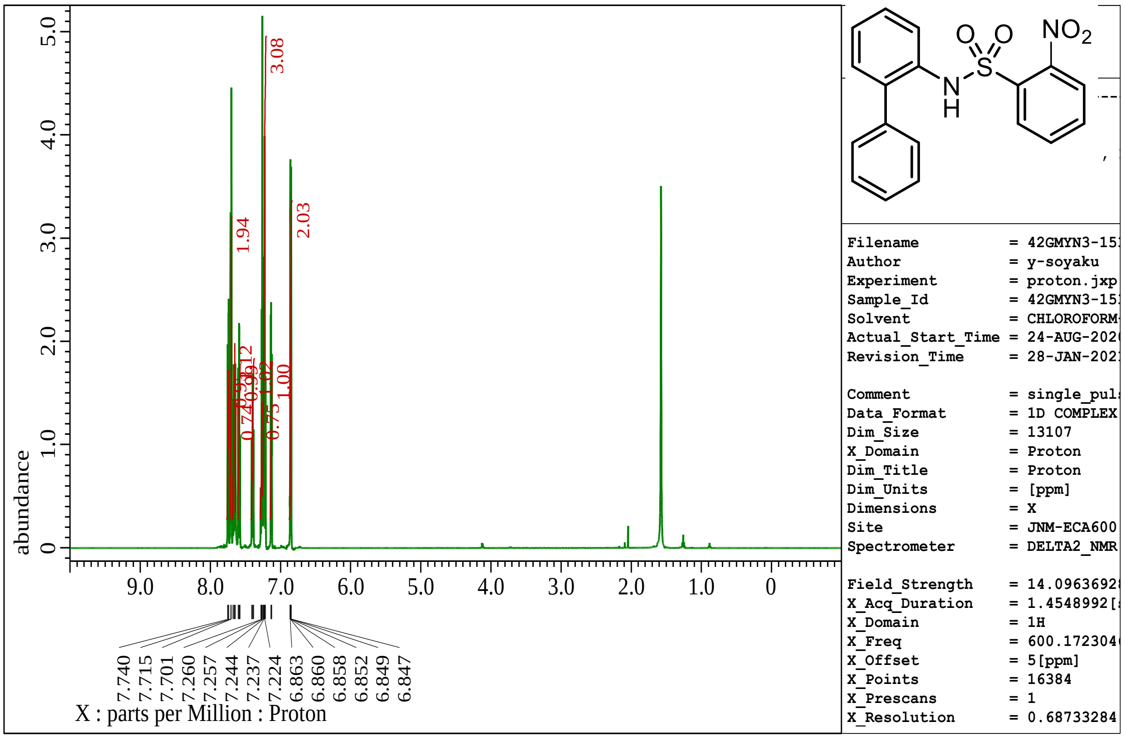

^{13}C{^{1}H }-NMR (150 MHz, $CDCl_3$) of **S7e**

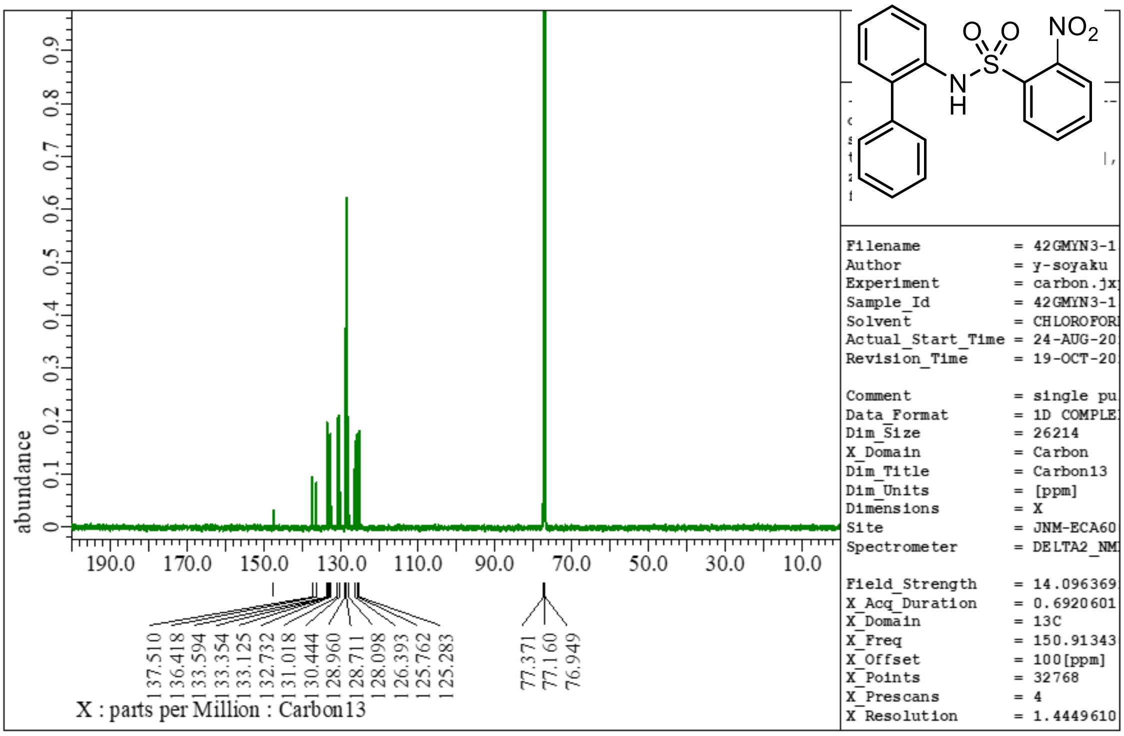

H-H COSY-NMR (600 MHz, $CDCl_3$) of **S7e**

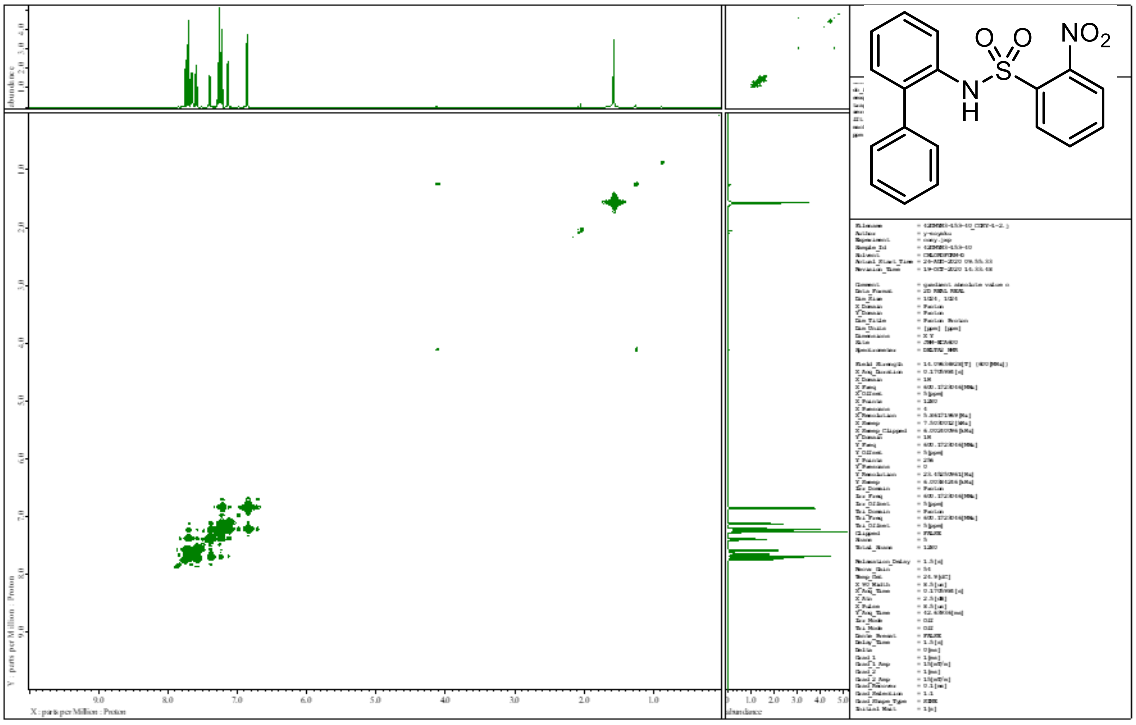

HMQC-NMR ($CDCl_3$) of **S7e**

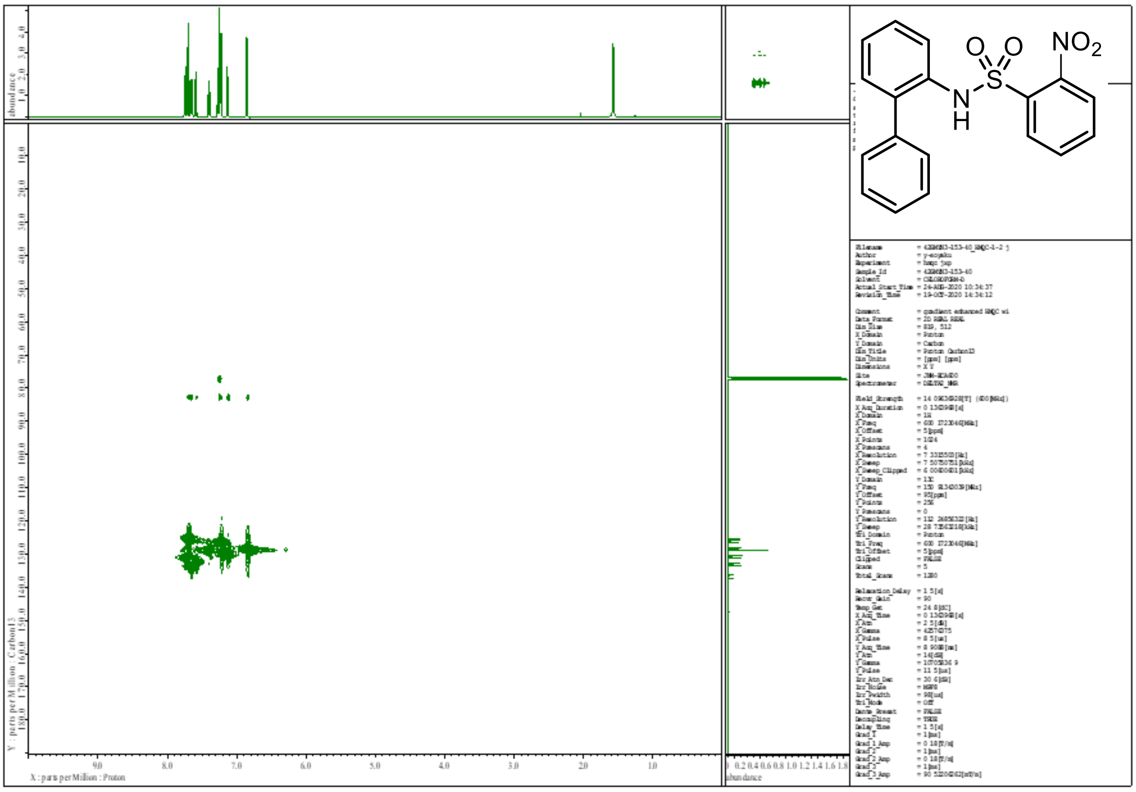

^{1}H-NMR (600 MHz, $CDCl_3$) of **S7f**

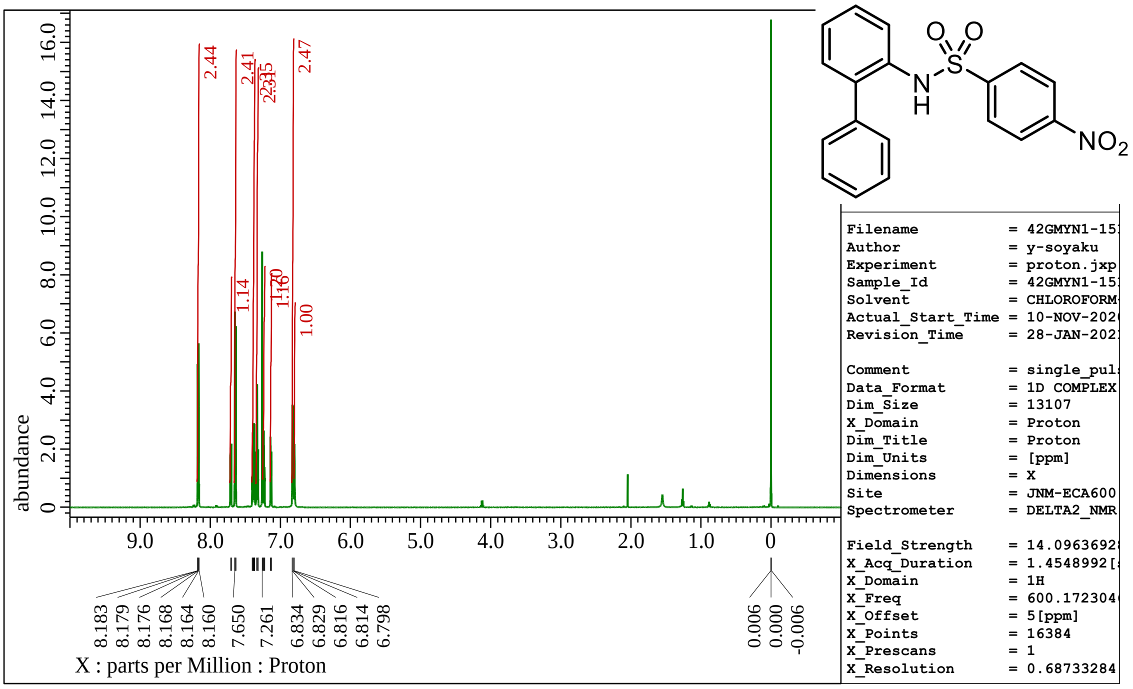

$^{13}C\{^{1}H\}$-NMR (150 MHz, $CDCl_3$) of **S7f**

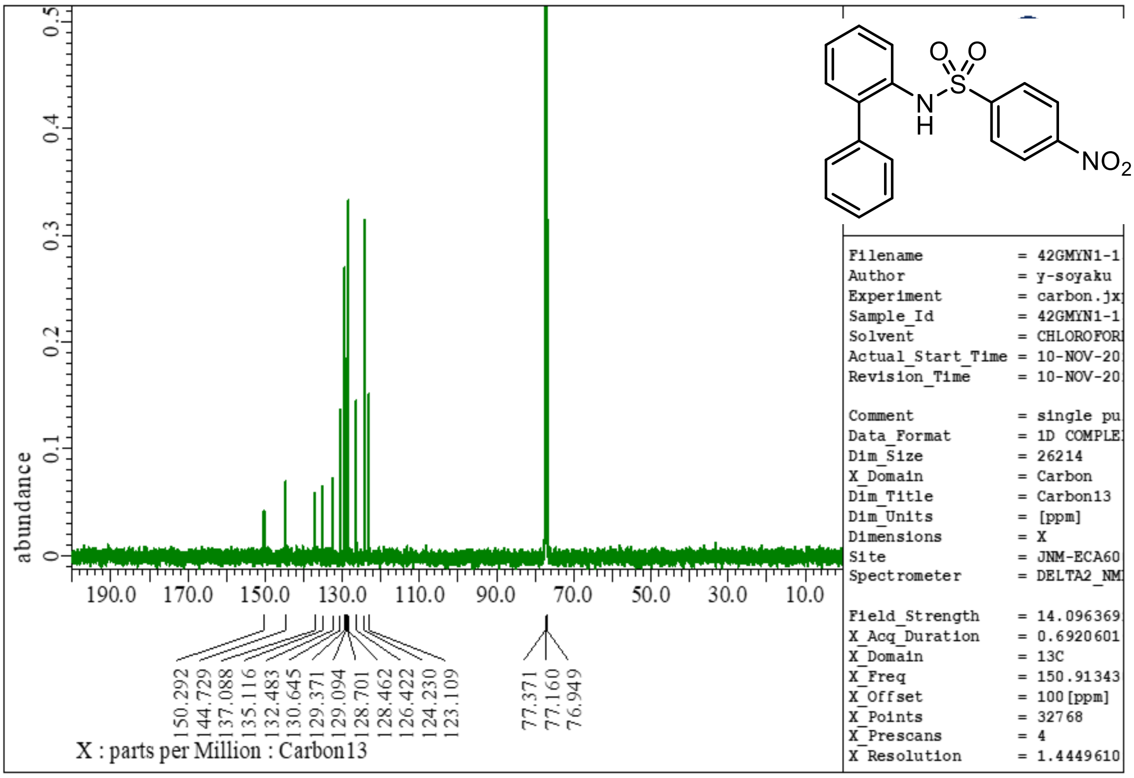

H-H COSY-NMR (600 MHz, $CDCl_3$) of **S7f**

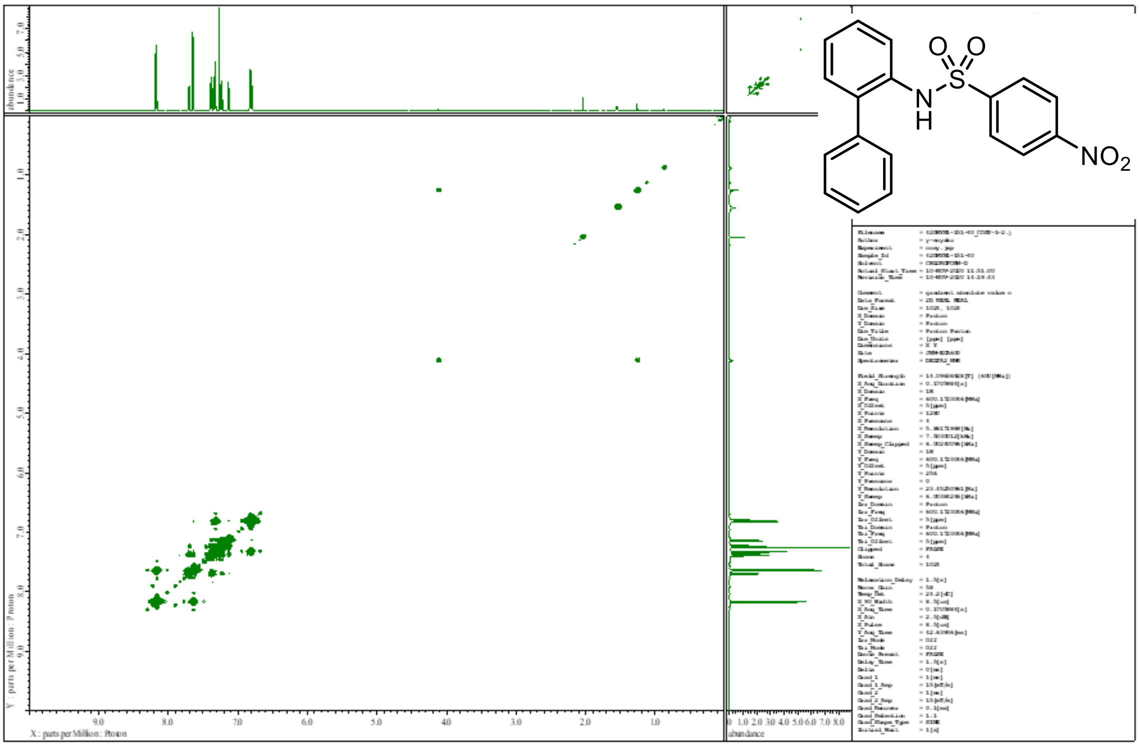

HMQC-NMR ($CDCl_3$) of **S7f**

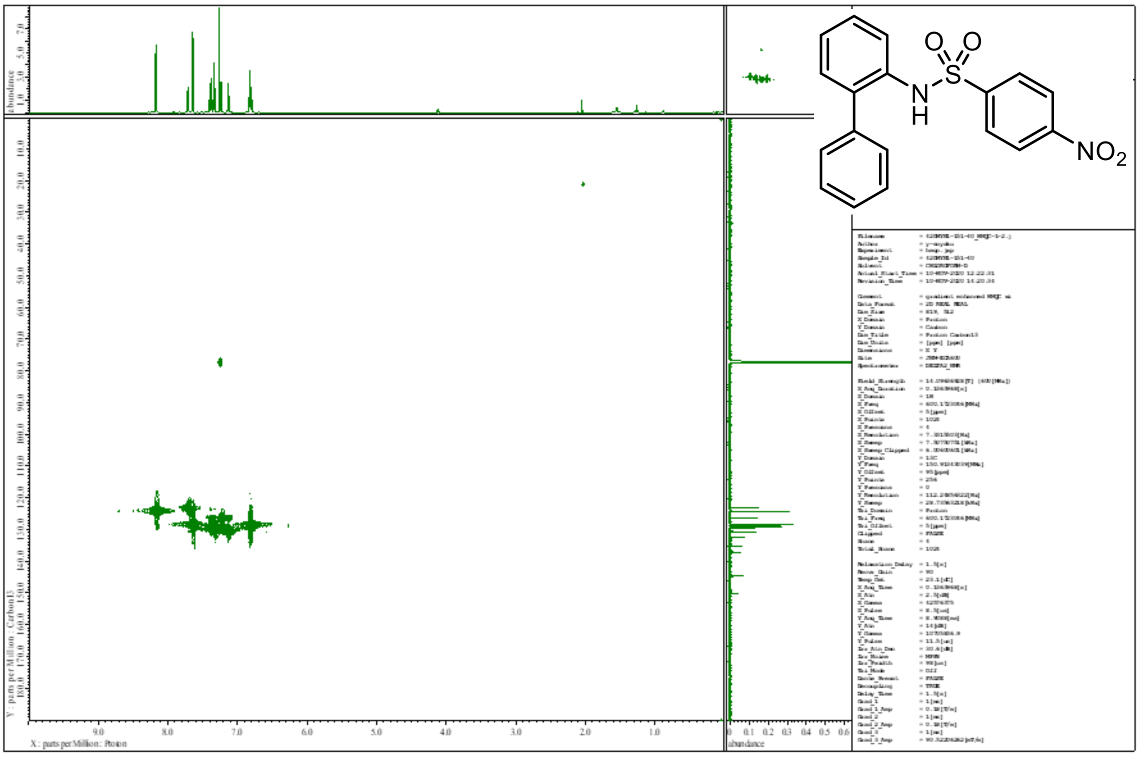

^{1}H-NMR (600 MHz, $CDCl_3$) of **S8c**

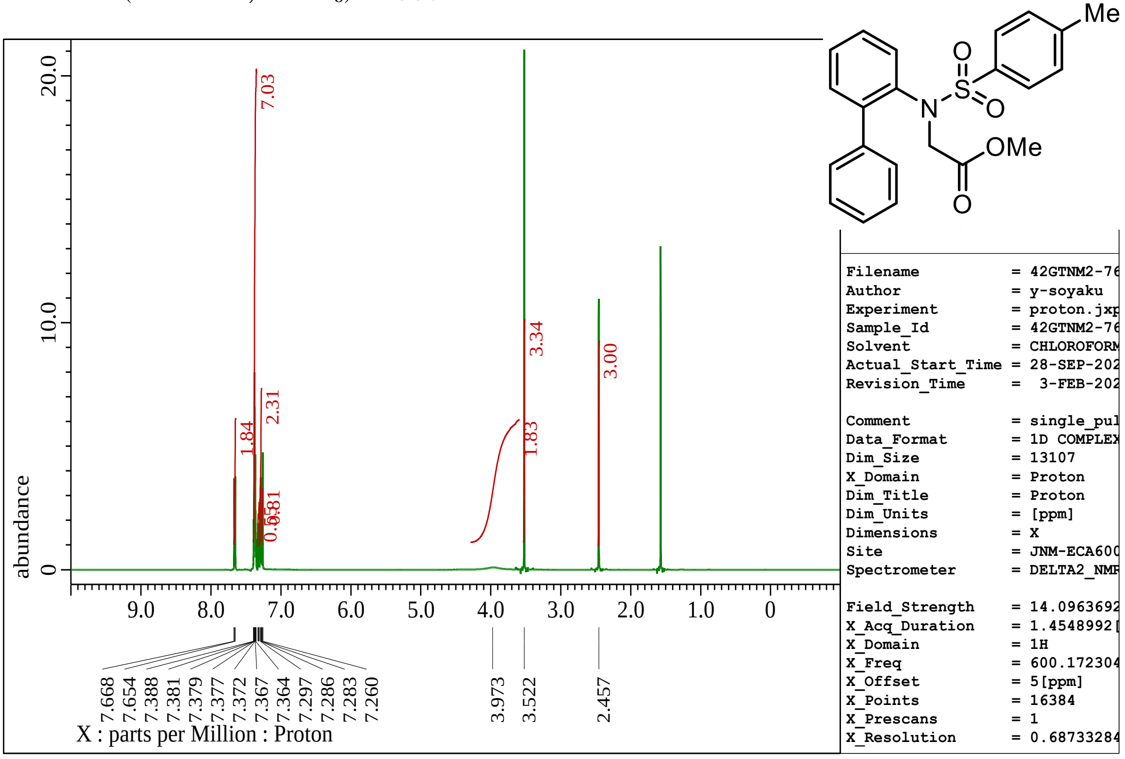

$^{13}C\{^{1}H\}$-NMR (150 MHz, $CDCl_3$) of **S8c**

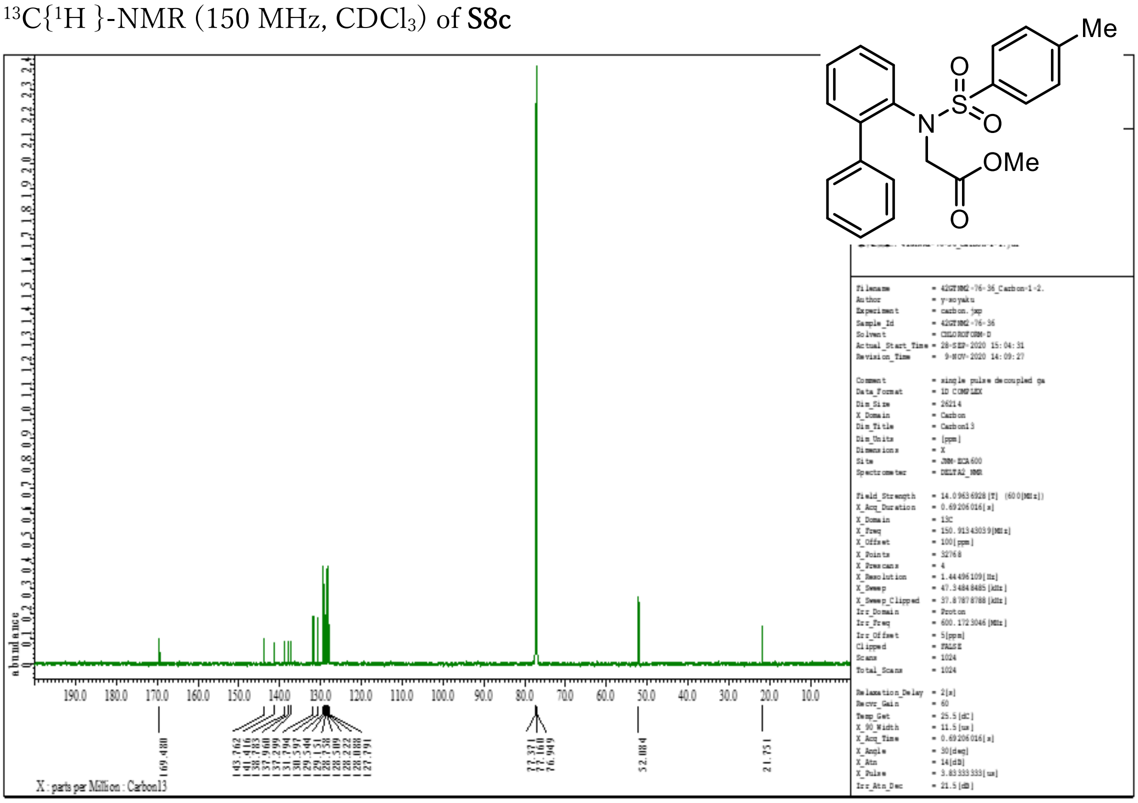

H-H COSY-NMR (600 MHz, $CDCl_3$) of **S8c**

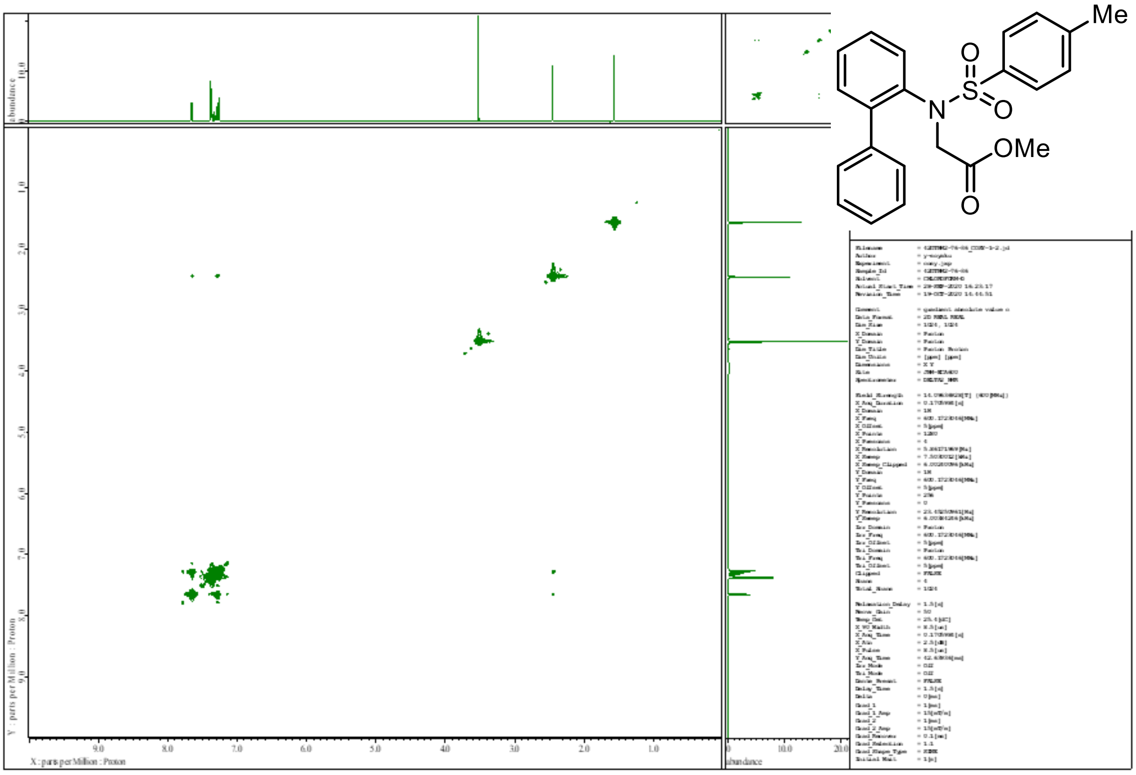

HMQC-NMR ($CDCl_3$) of **S8c**

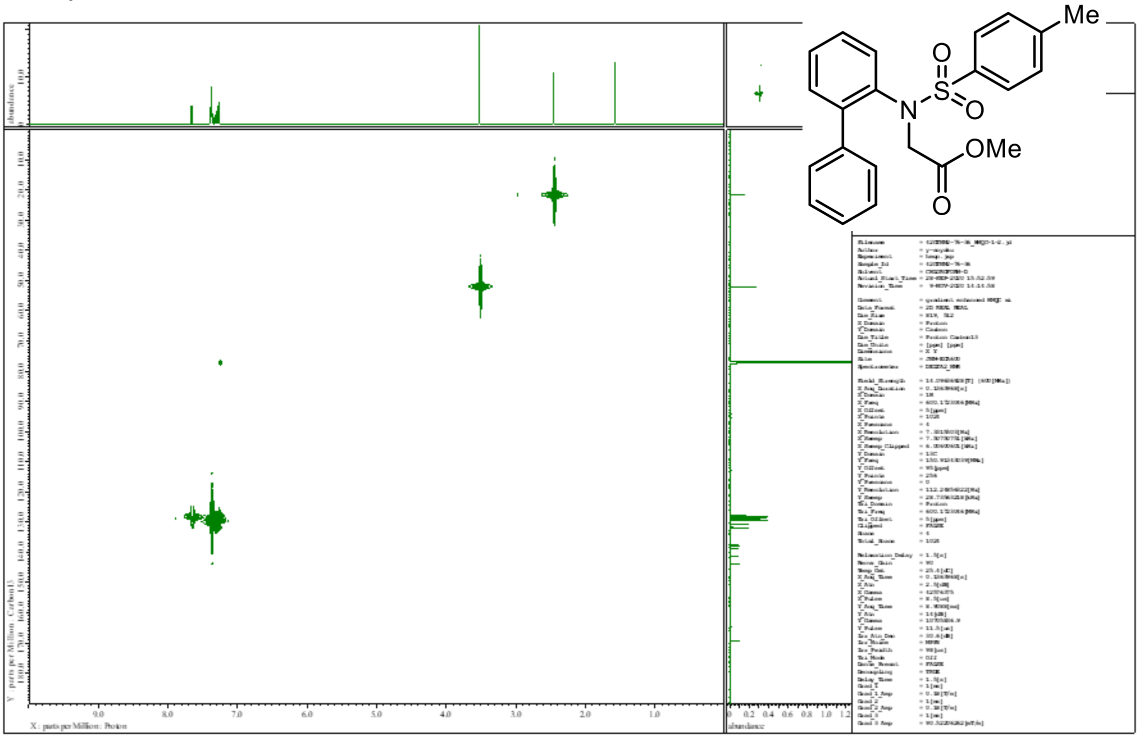

^{1}H-NMR (600 MHz, $CDCl_3$) of **S8d**

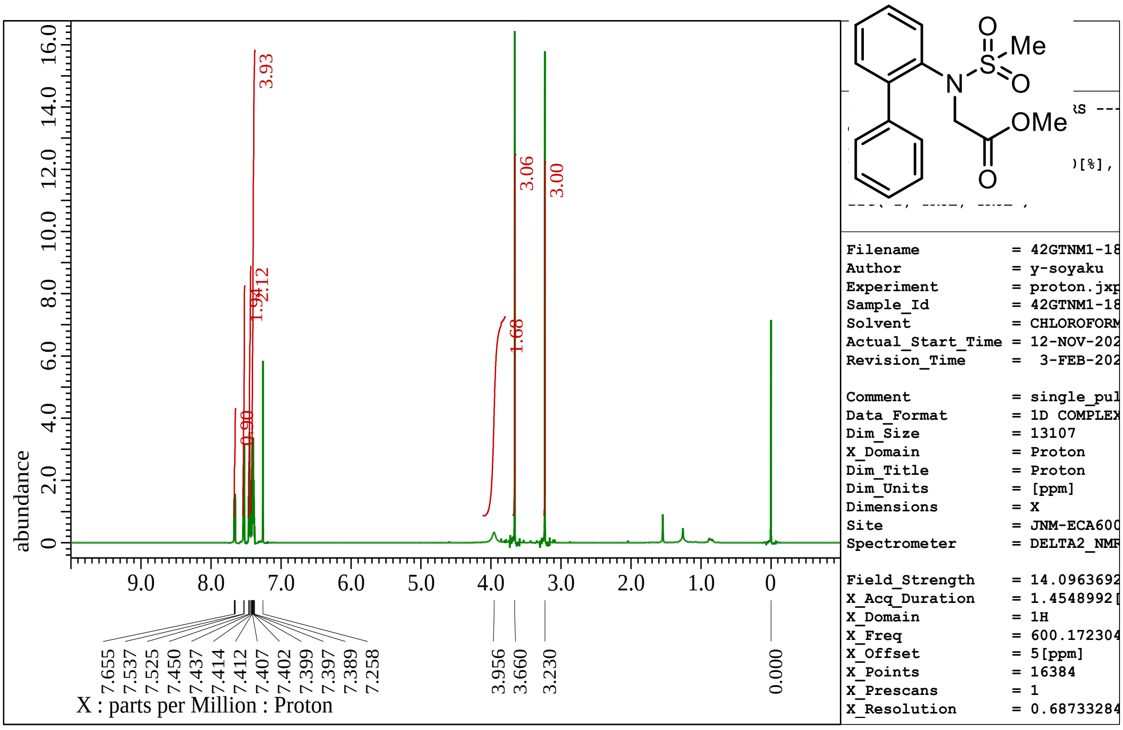

^{13}C{^{1}H }-NMR (150 MHz, $CDCl_3$) of **S8d**

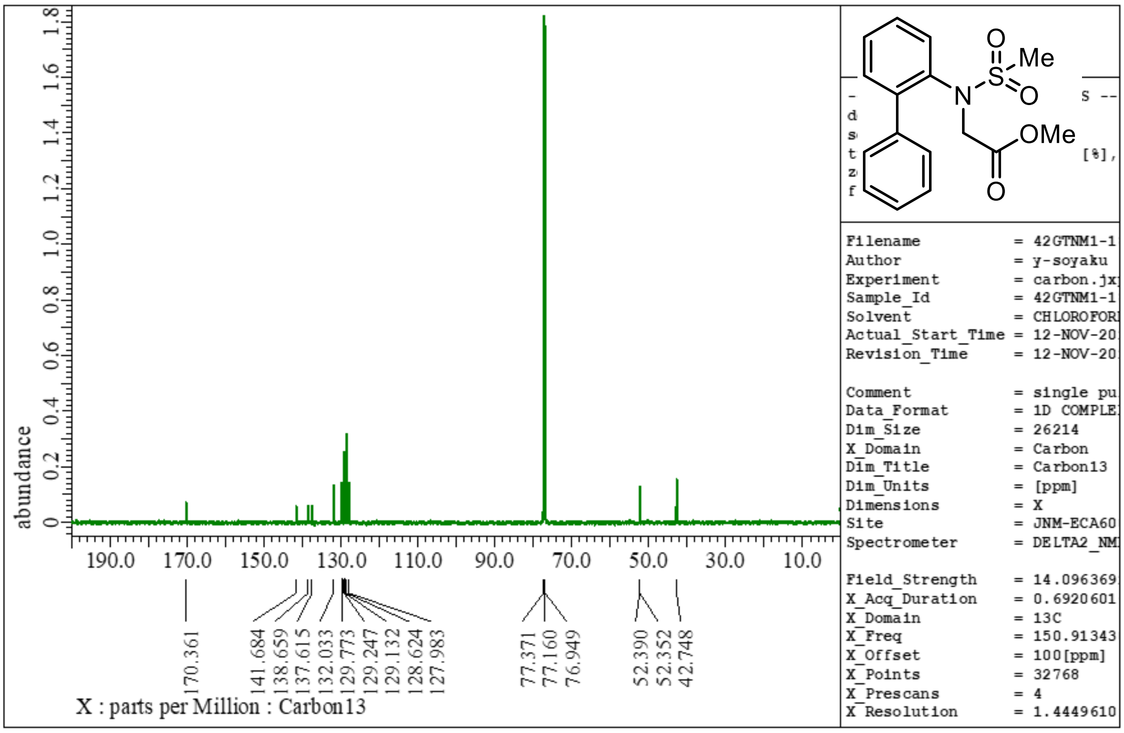

H-H COSY-NMR (600 MHz, $CDCl_3$) of **S8d**

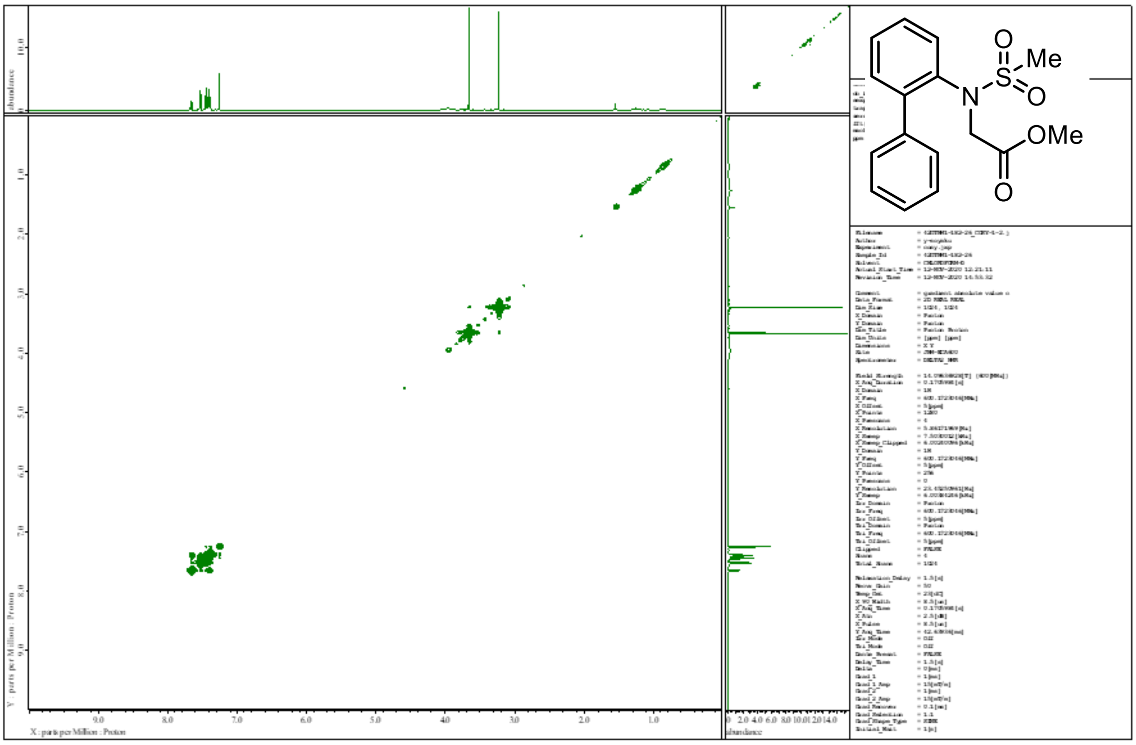

HMQC-NMR ($CDCl_3$) of **S8d**

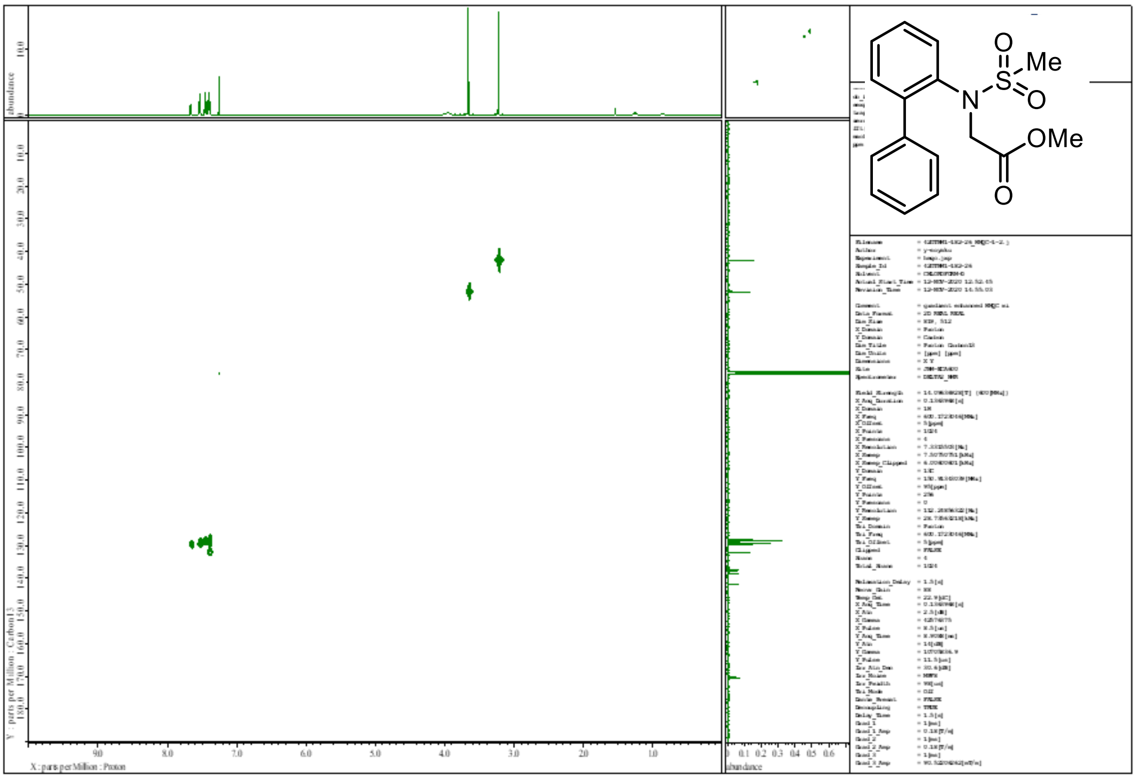

^{1}H-NMR (600 MHz, CDCl$_3$) of **S8e**

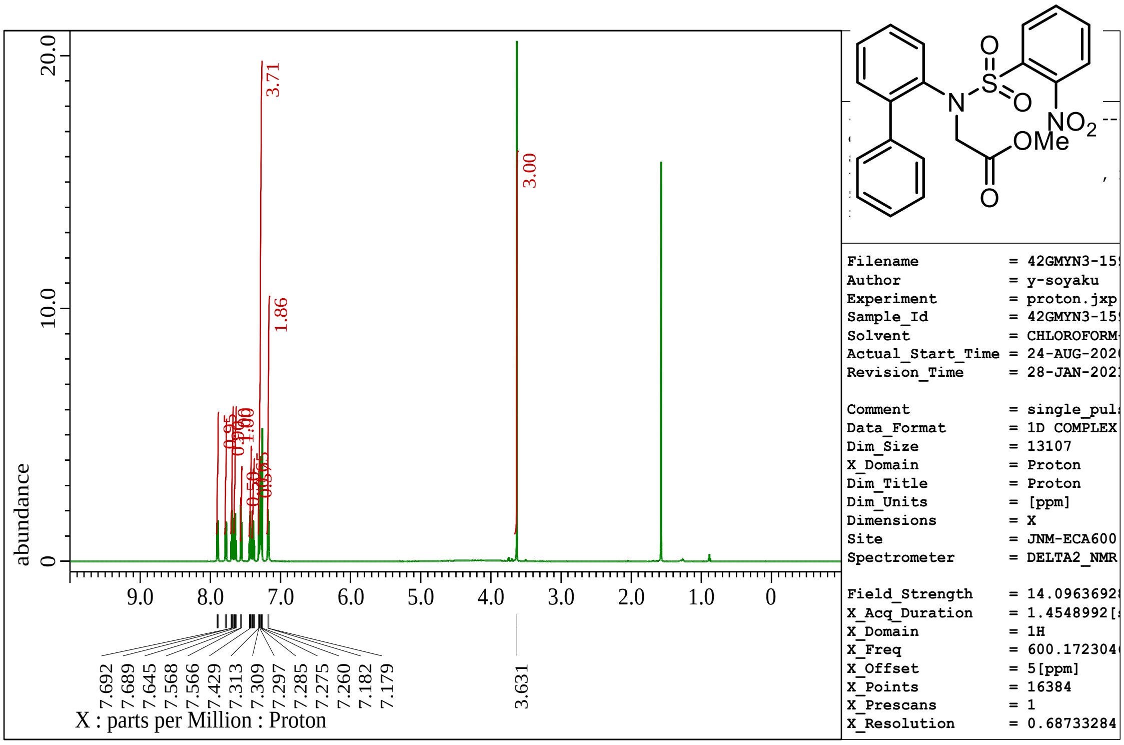

^{13}C{^{1}H }-NMR (150 MHz, CDCl$_3$) of **S8e**

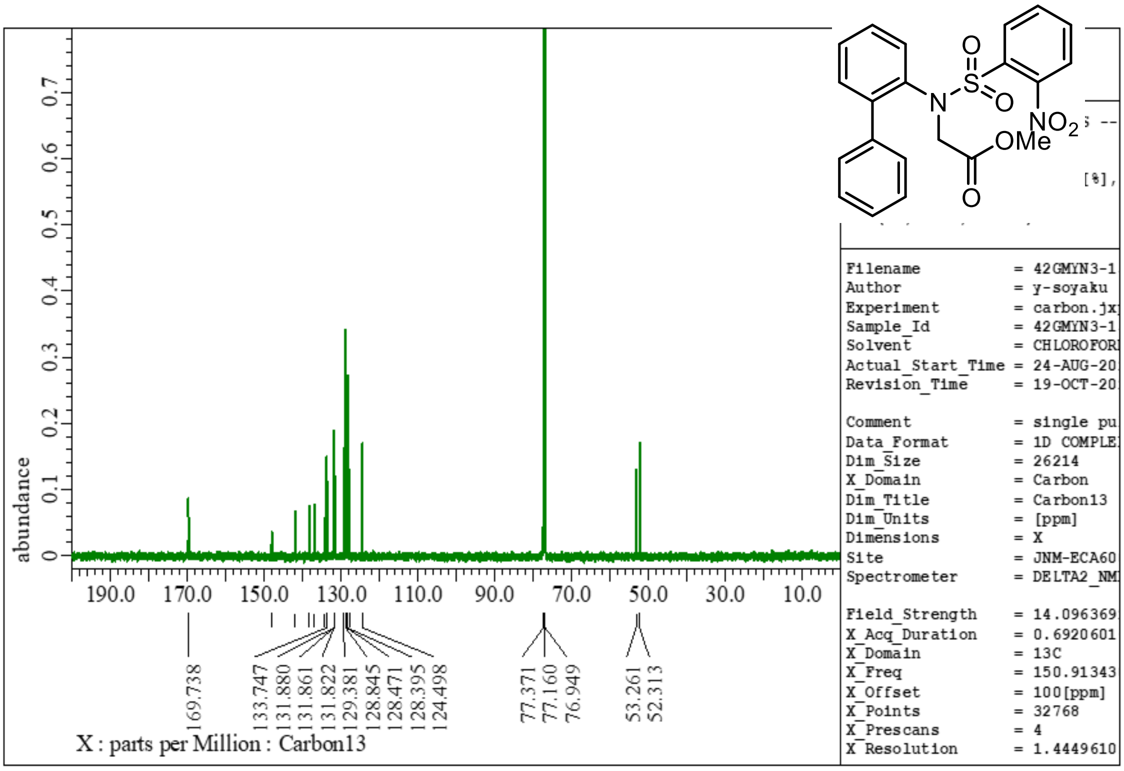

H-H COSY-NMR (600 MHz, $CDCl_3$) of **S8e**

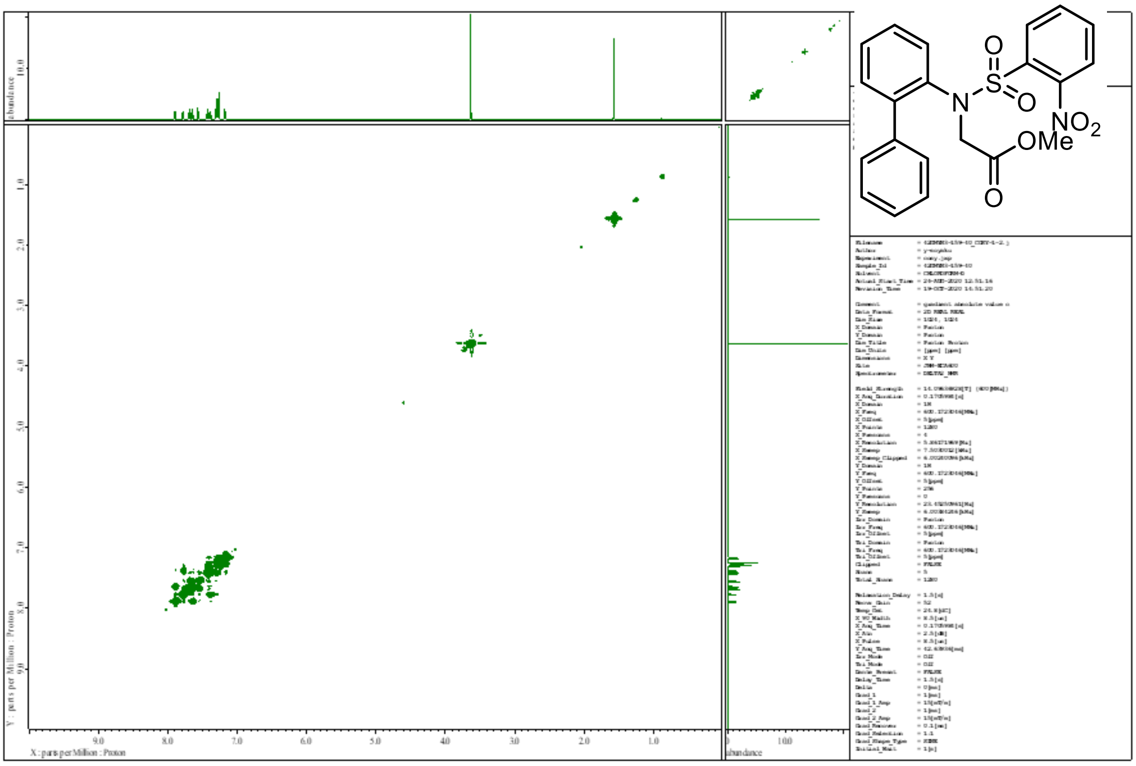

HMQC-NMR ($CDCl_3$) of **S8e**

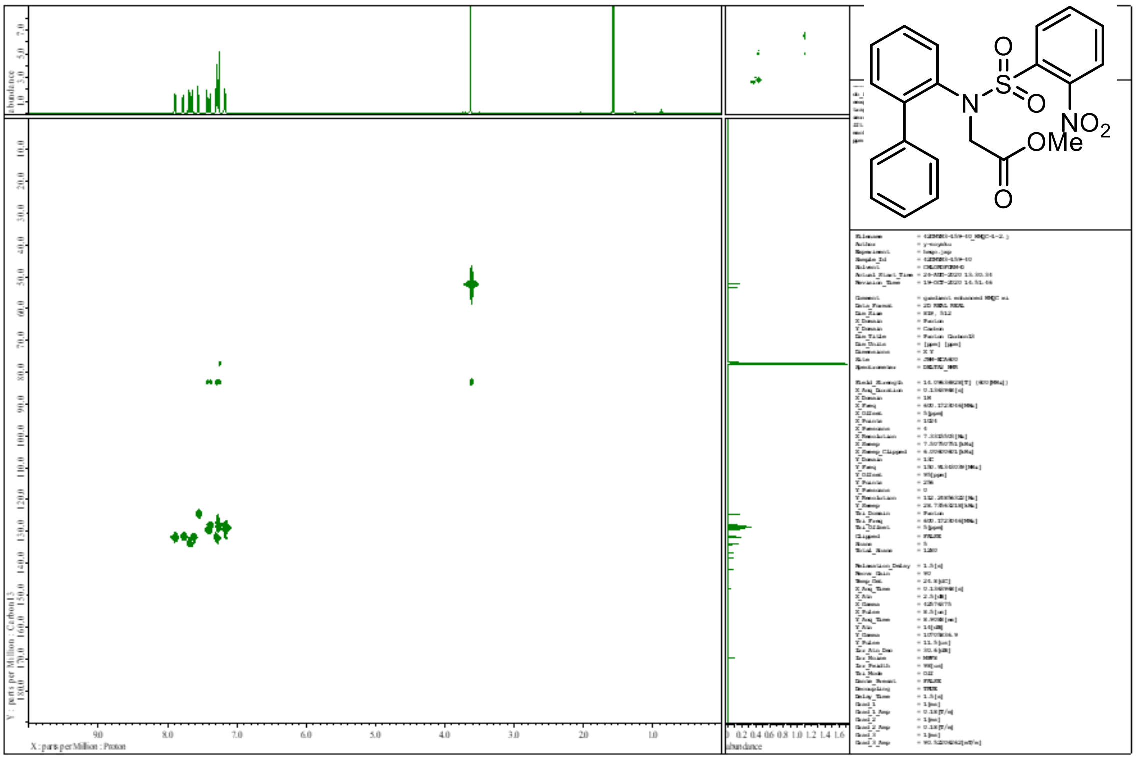

^{1}H-NMR (600 MHz, $CDCl_3$) of **S8f**

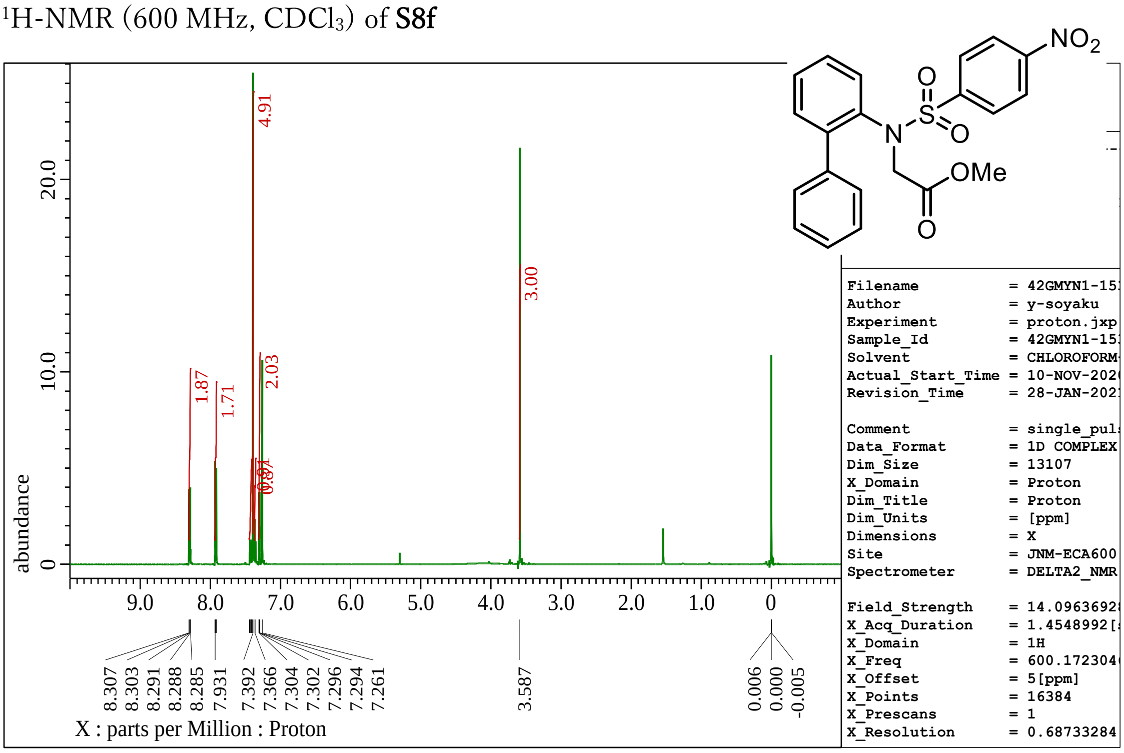

$^{13}C\{^{1}H\}$-NMR (150 MHz, $CDCl_3$) of **S8f**

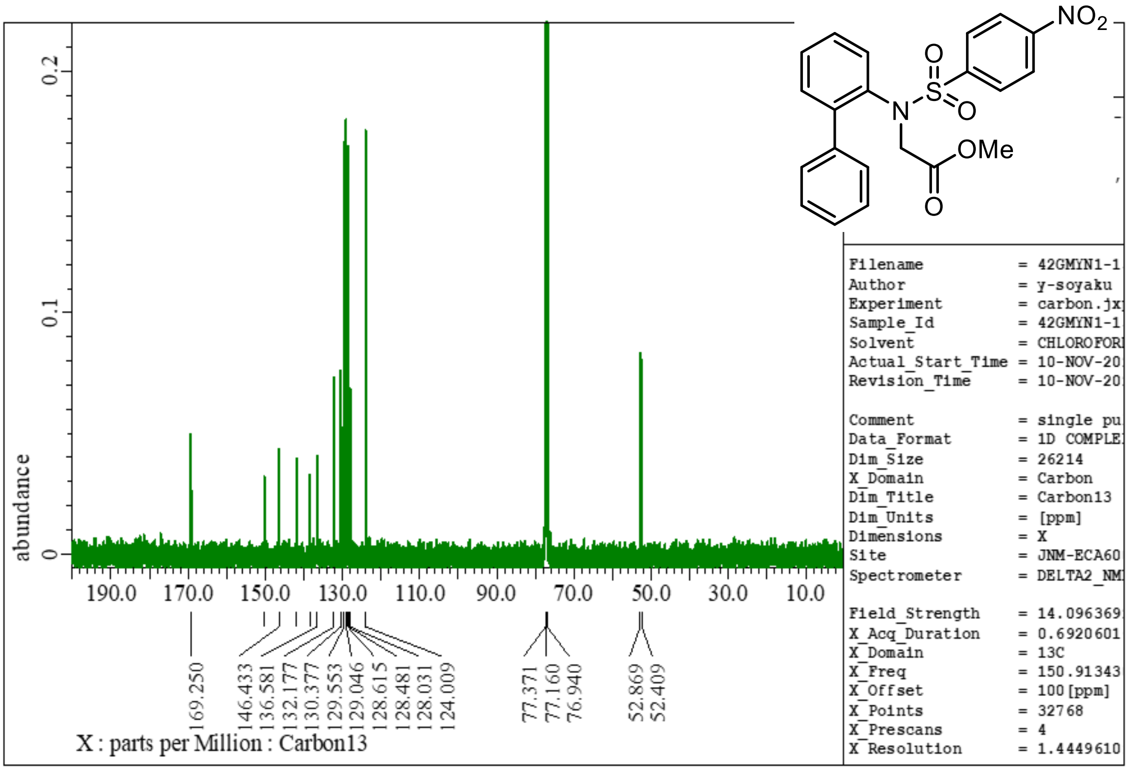

H-H COSY-NMR (600 MHz, $CDCl_3$) of **S8f**

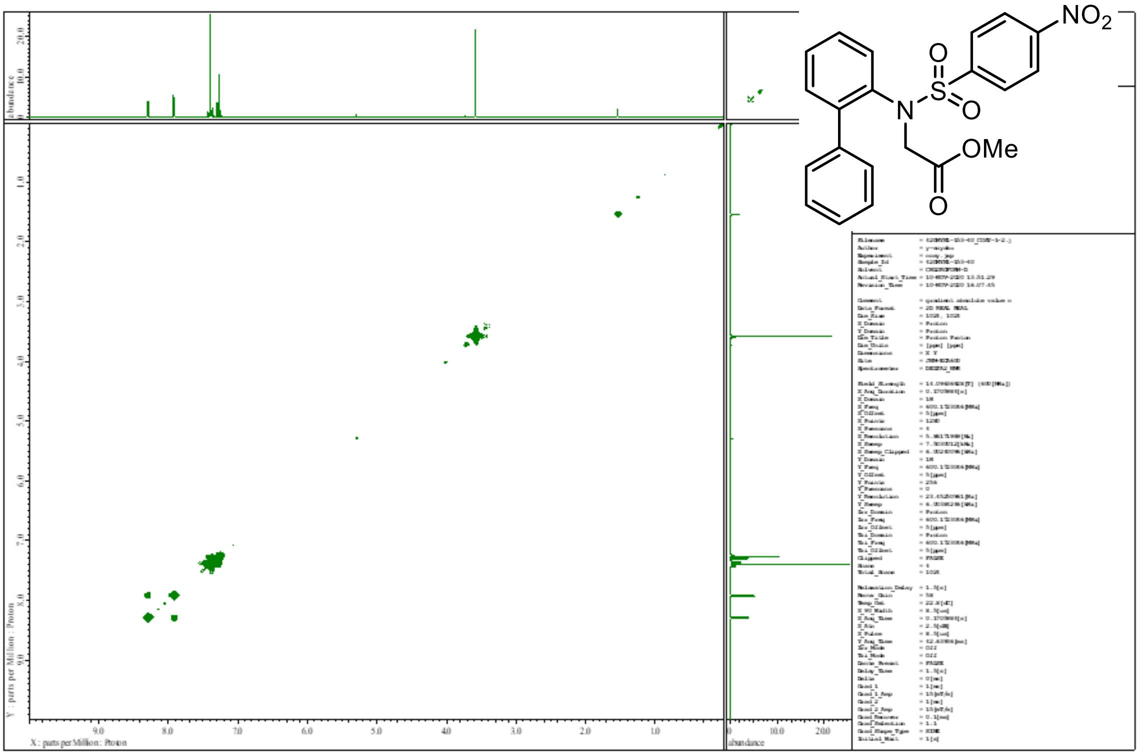

HMQC-NMR ($CDCl_3$) of **S8f**

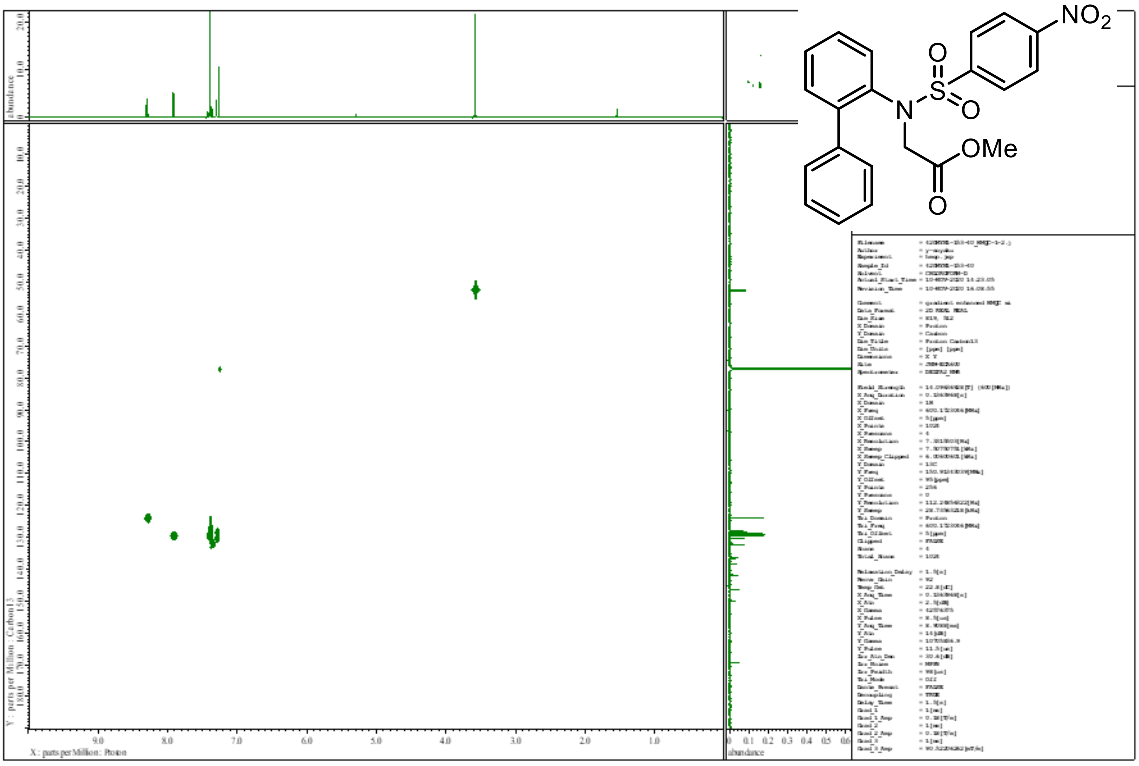

^{1}H-NMR (600 MHz, $CDCl_3$) of **S9c**

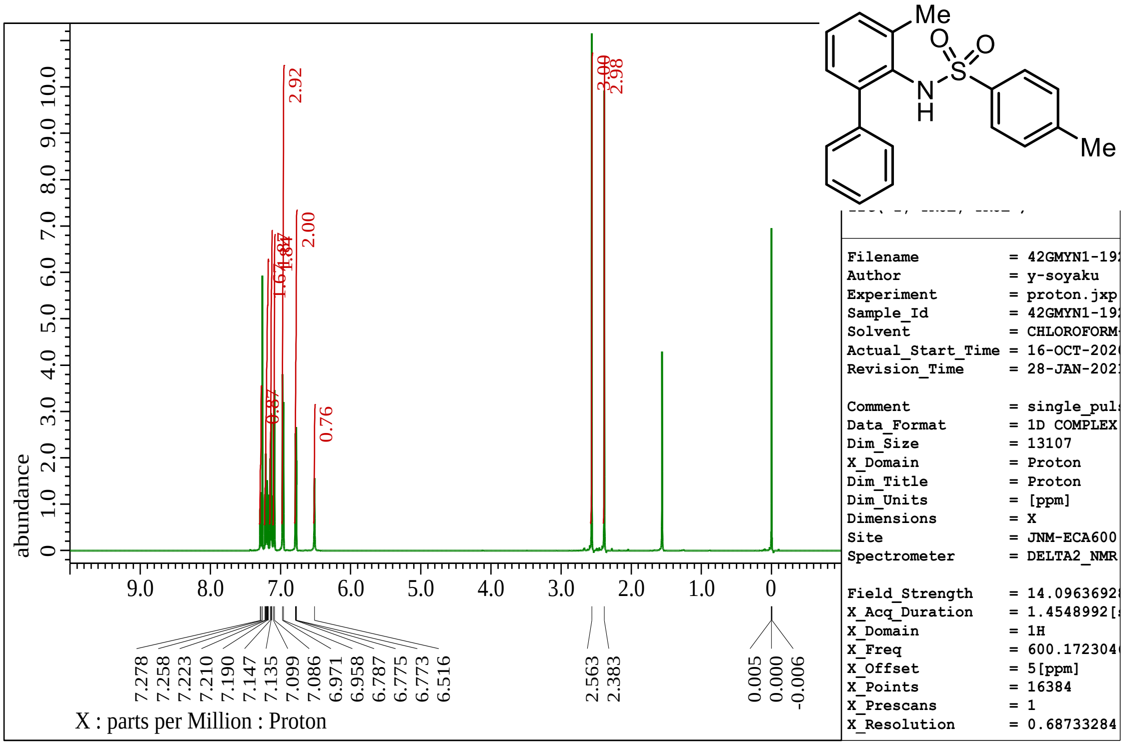

^{13}C{^{1}H }-NMR (150 MHz, $CDCl_3$) of **S9c**

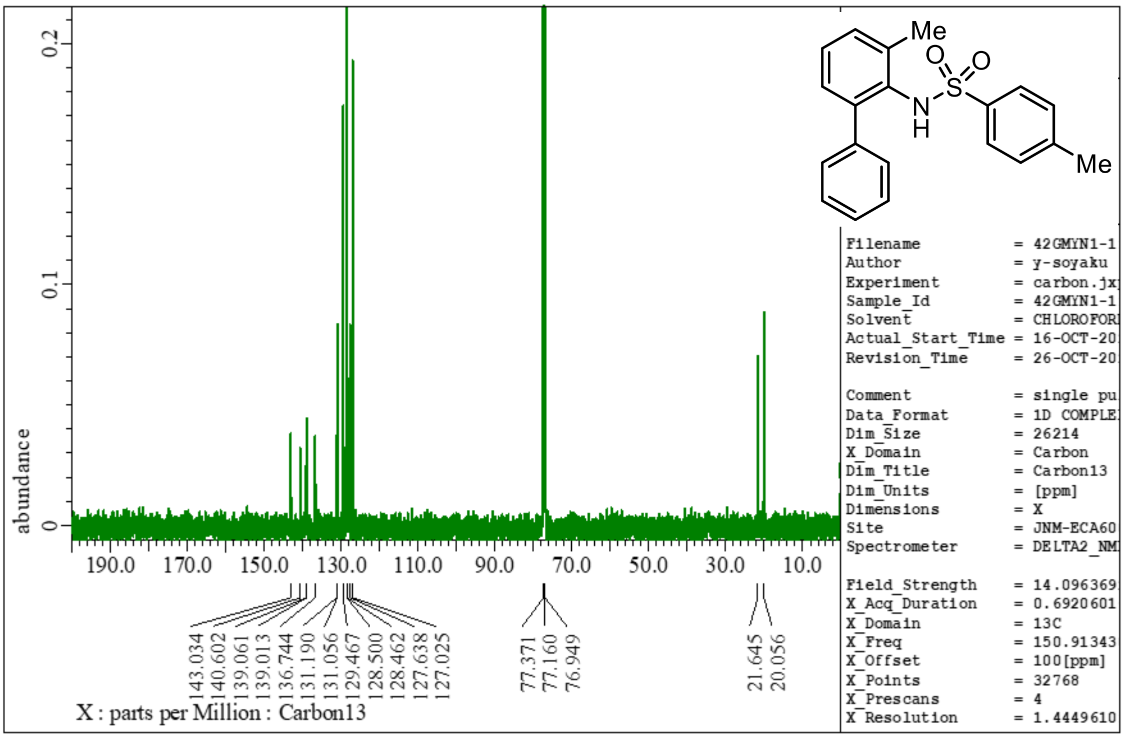

H-H COSY-NMR (600 MHz, $CDCl_3$) of **S9c**

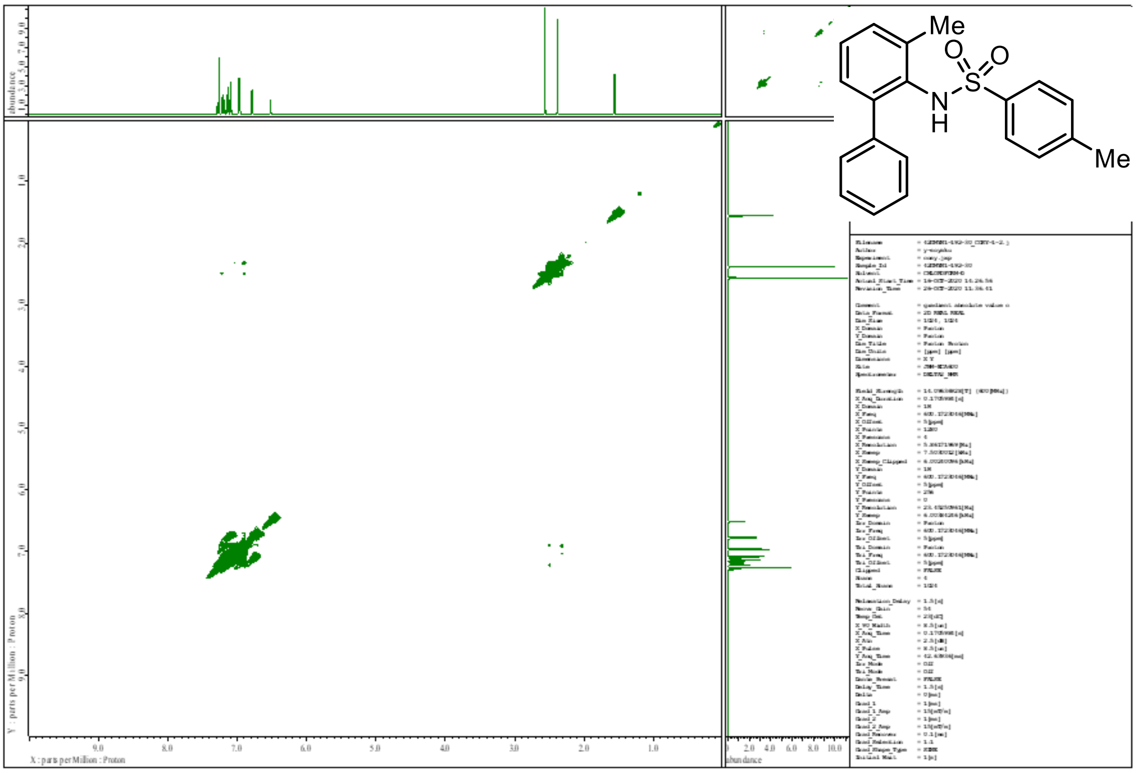

HMQC-NMR ($CDCl_3$) of **S9c**

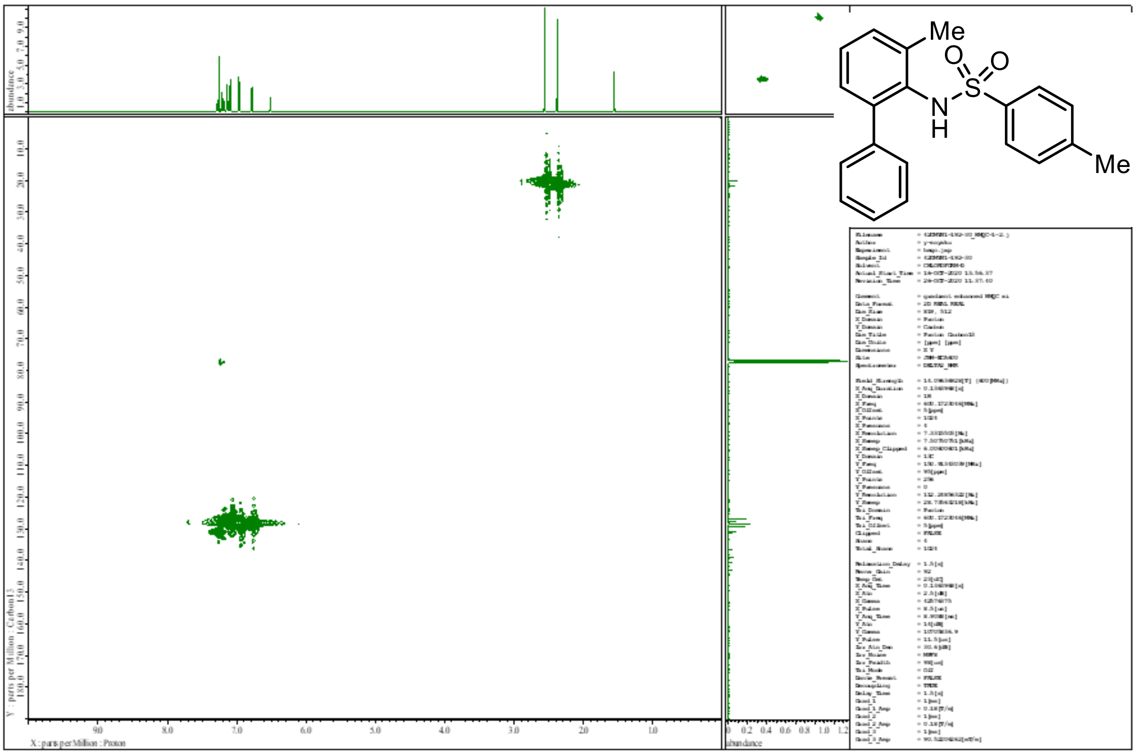

^{1}H-NMR (600 MHz, $CDCl_3$) of **S9d**

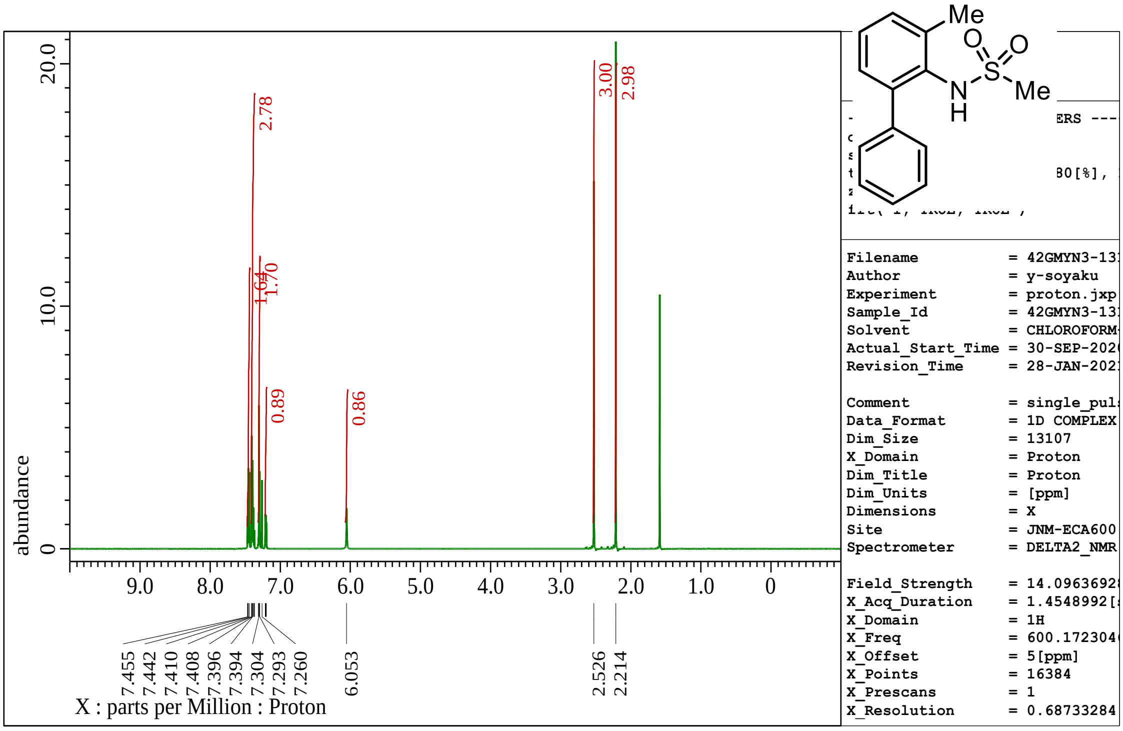

$^{13}C\{^{1}H\}$-NMR (150 MHz, $CDCl_3$) of **S9d**

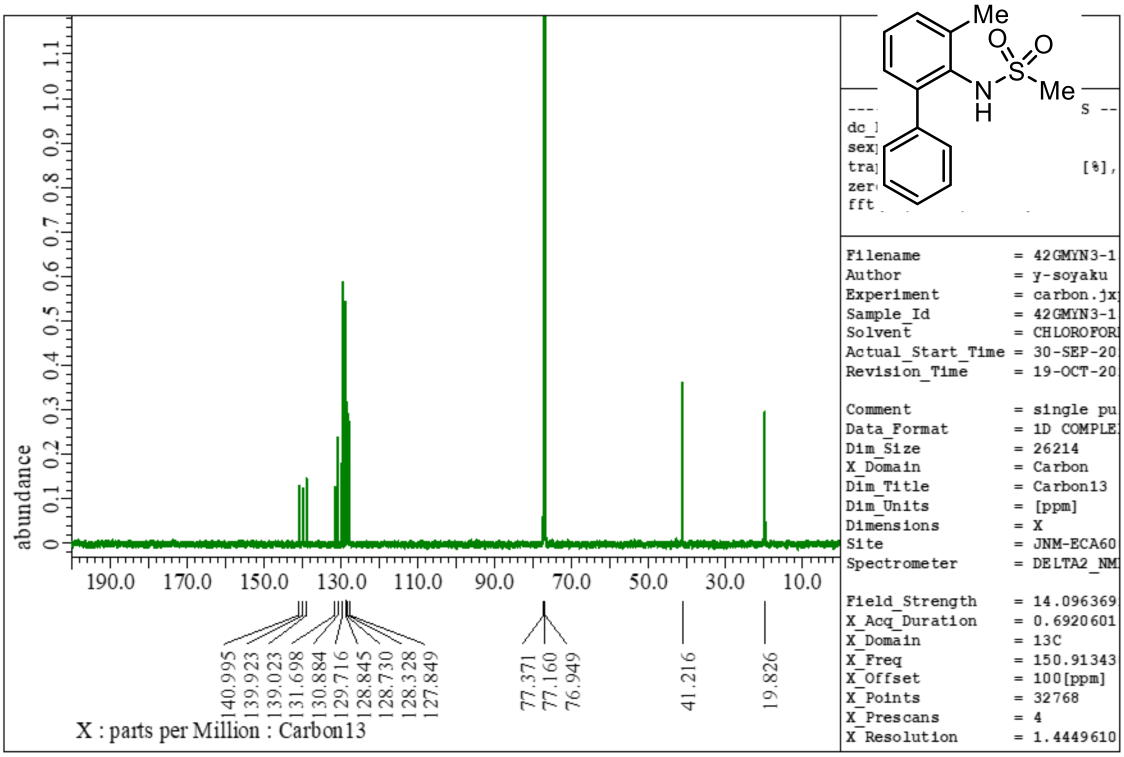

H-H COSY-NMR (600 MHz, $CDCl_3$) of **S9d**

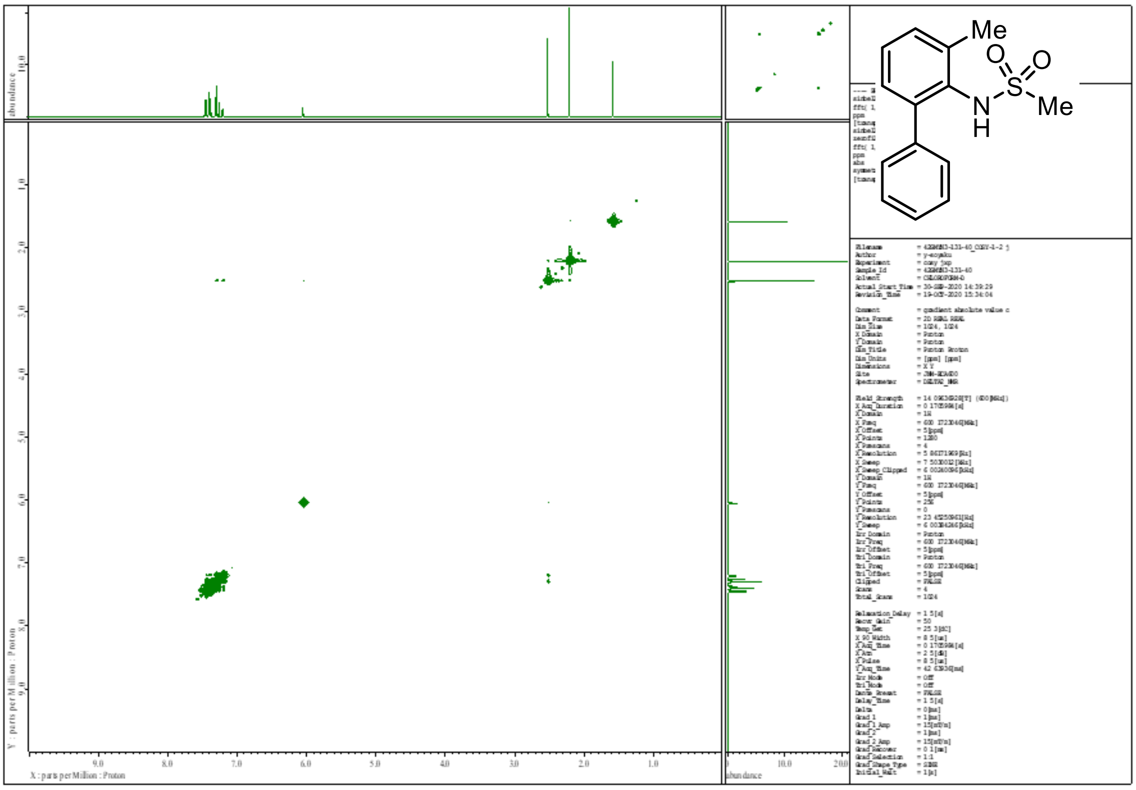

HMQC-NMR ($CDCl_3$) of **S9d**

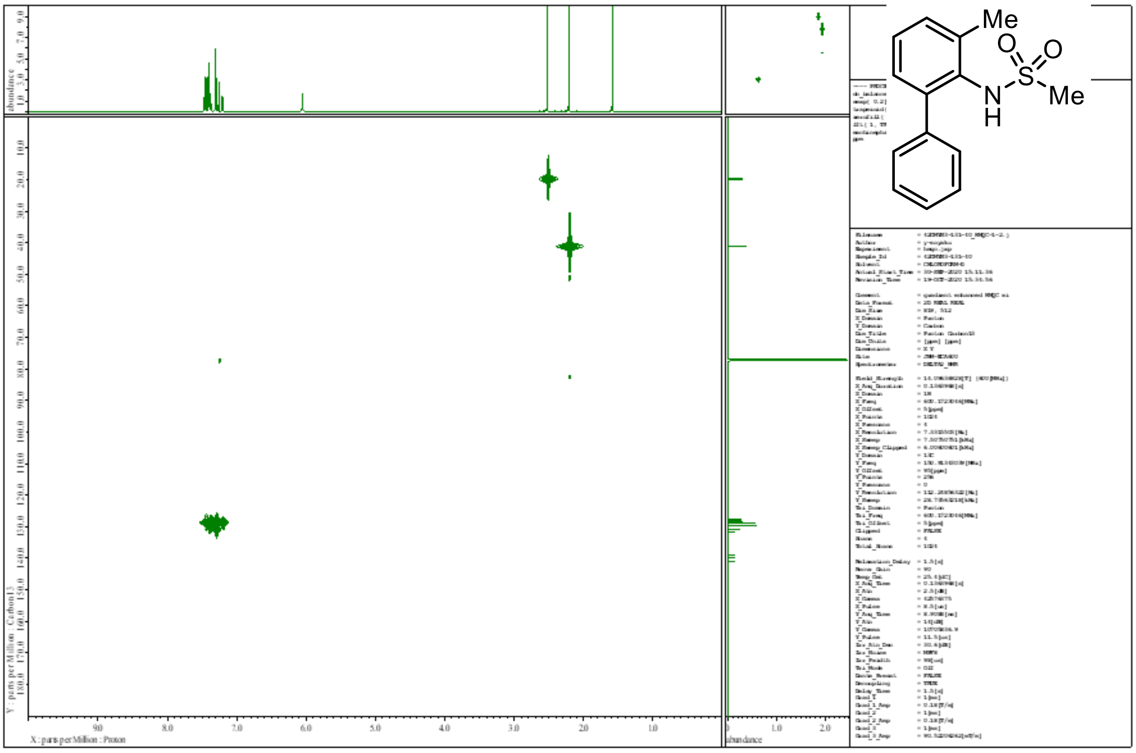

^{1}H-NMR (600 MHz, $CDCl_3$) of **S9e**

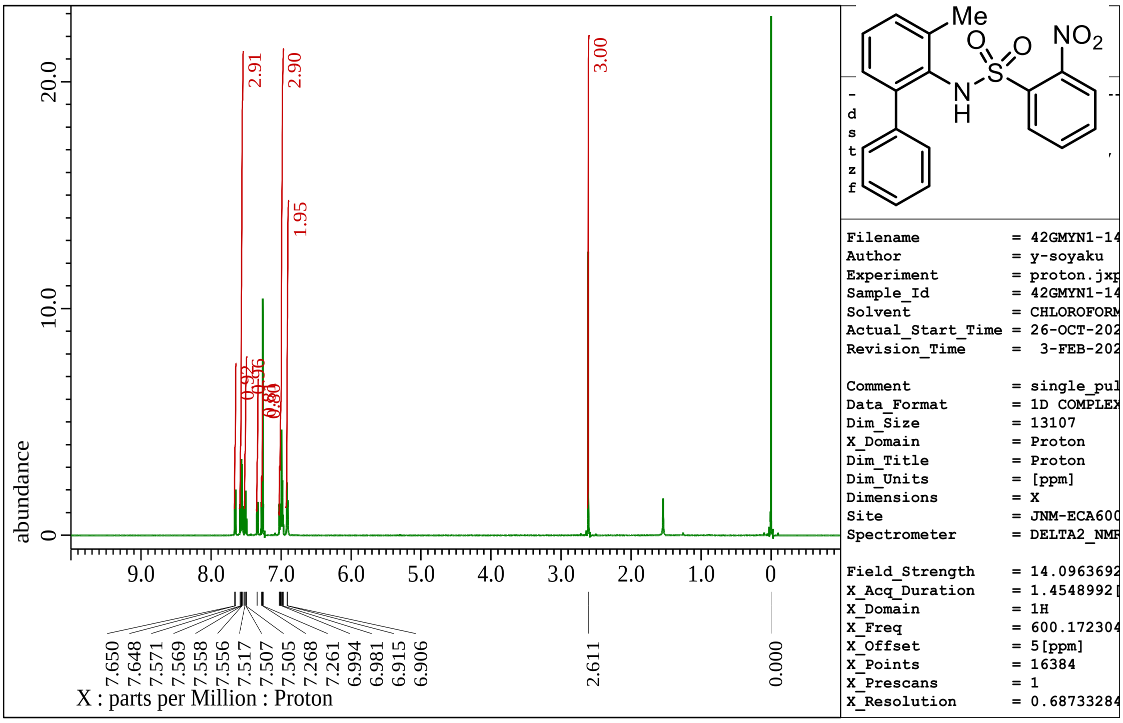

$^{13}C\{^{1}H\}$-NMR (150 MHz, $CDCl_3$) of **S9e**

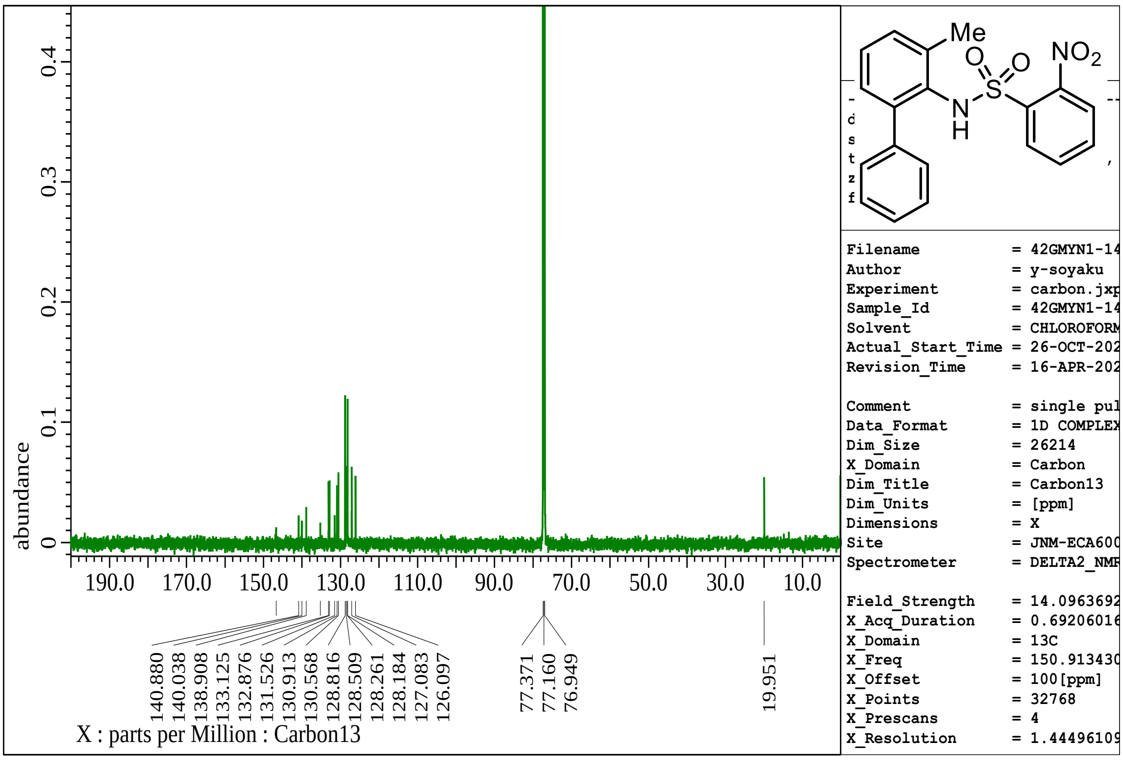

H-H COSY-NMR (600 MHz, $CDCl_3$) of **S9e**

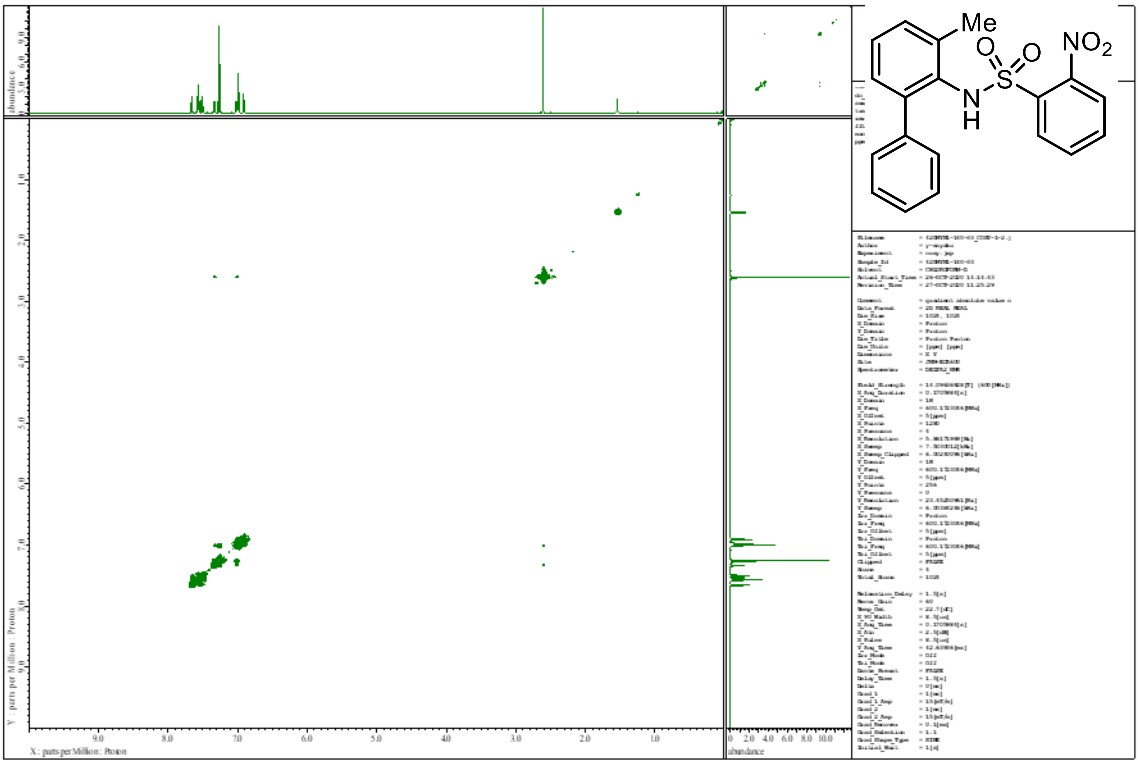

HMQC-NMR ($CDCl_3$) of **S9e**

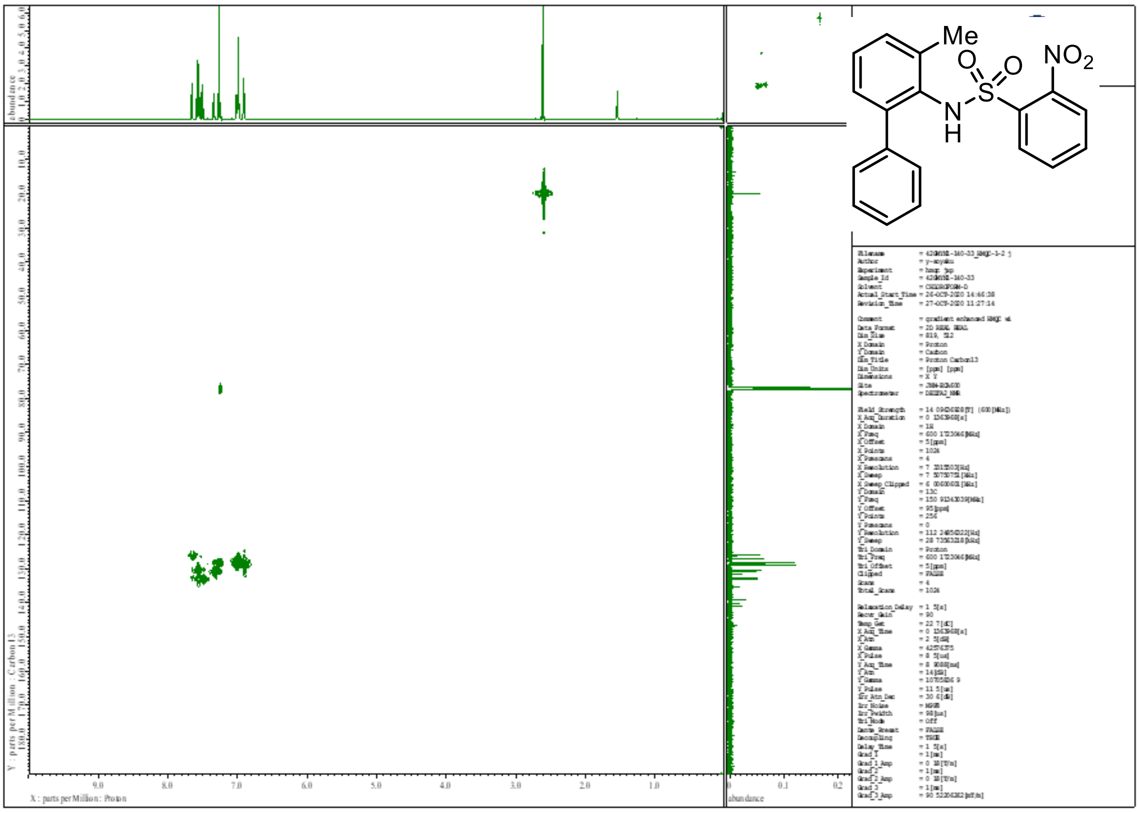

^{1}H-NMR (600 MHz, $CDCl_3$) of **S9f**

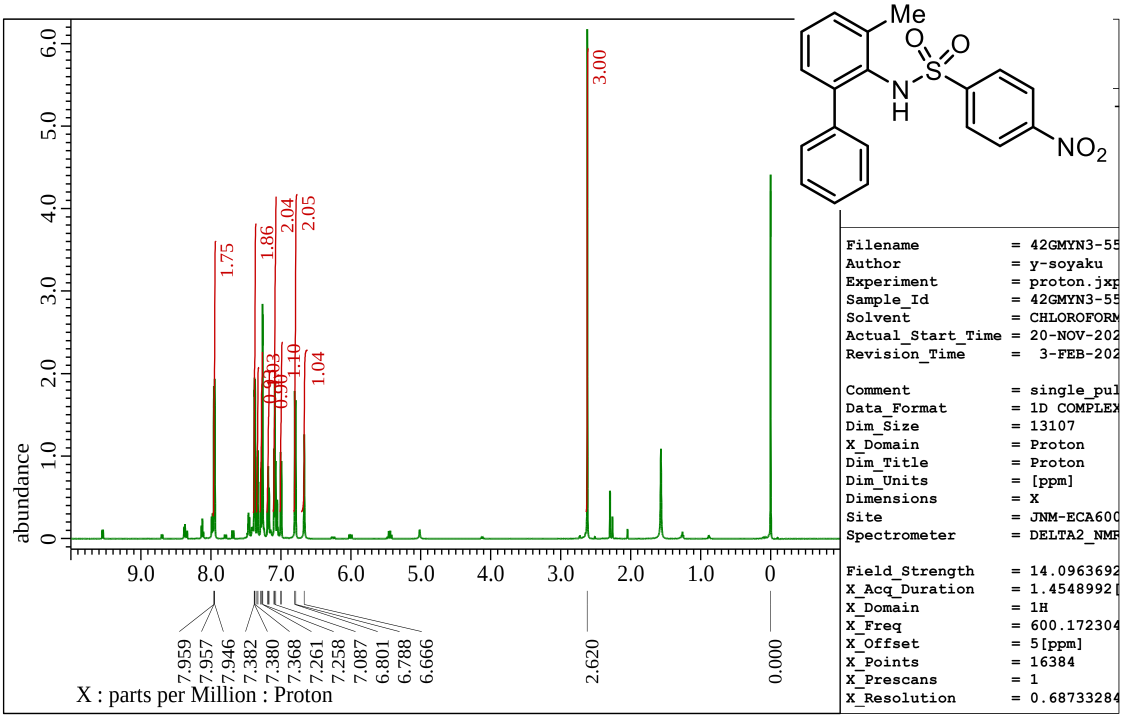

^{13}C{^{1}H }-NMR (150 MHz, $CDCl_3$) of **S9f**

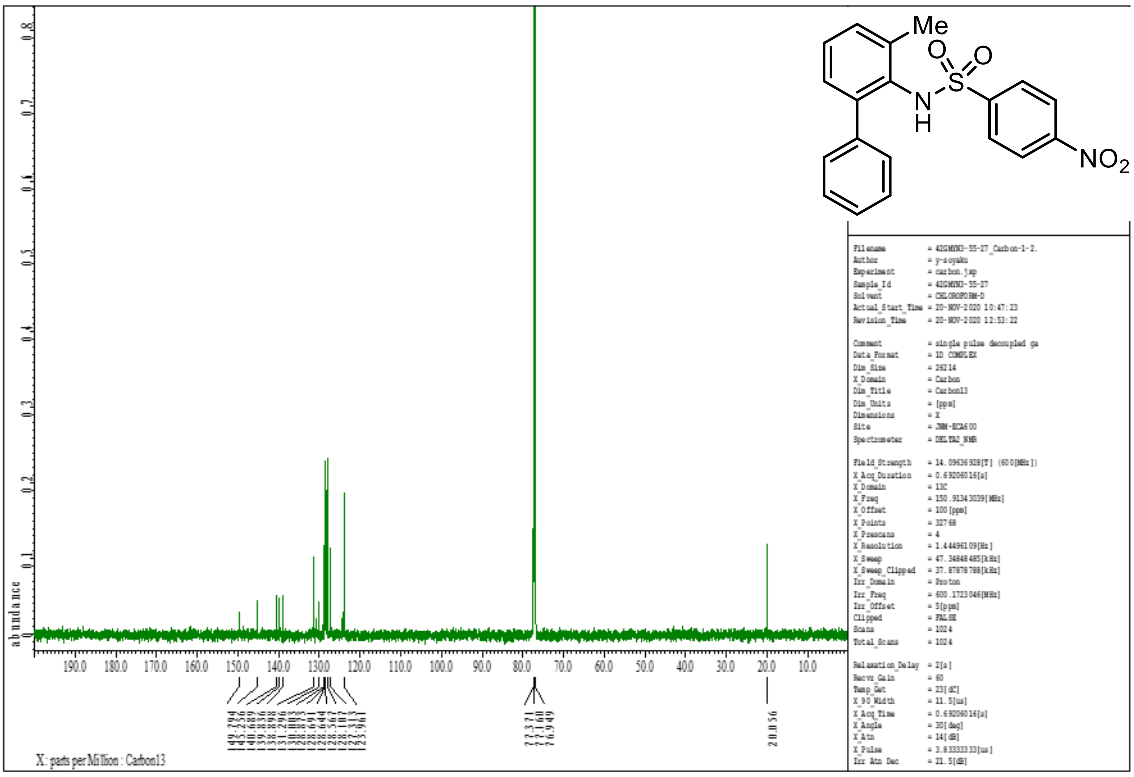

H-H COSY-NMR (600 MHz, $CDCl_3$) of **S9f**

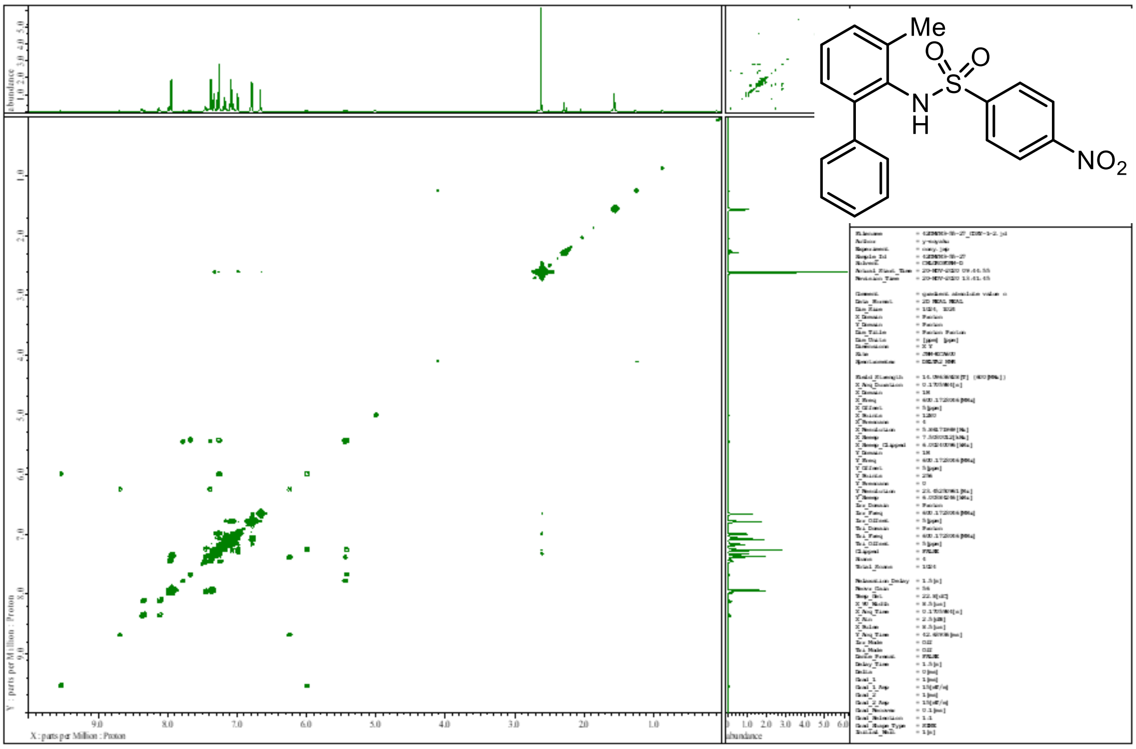

HMQC-NMR ($CDCl_3$) of **S9f**

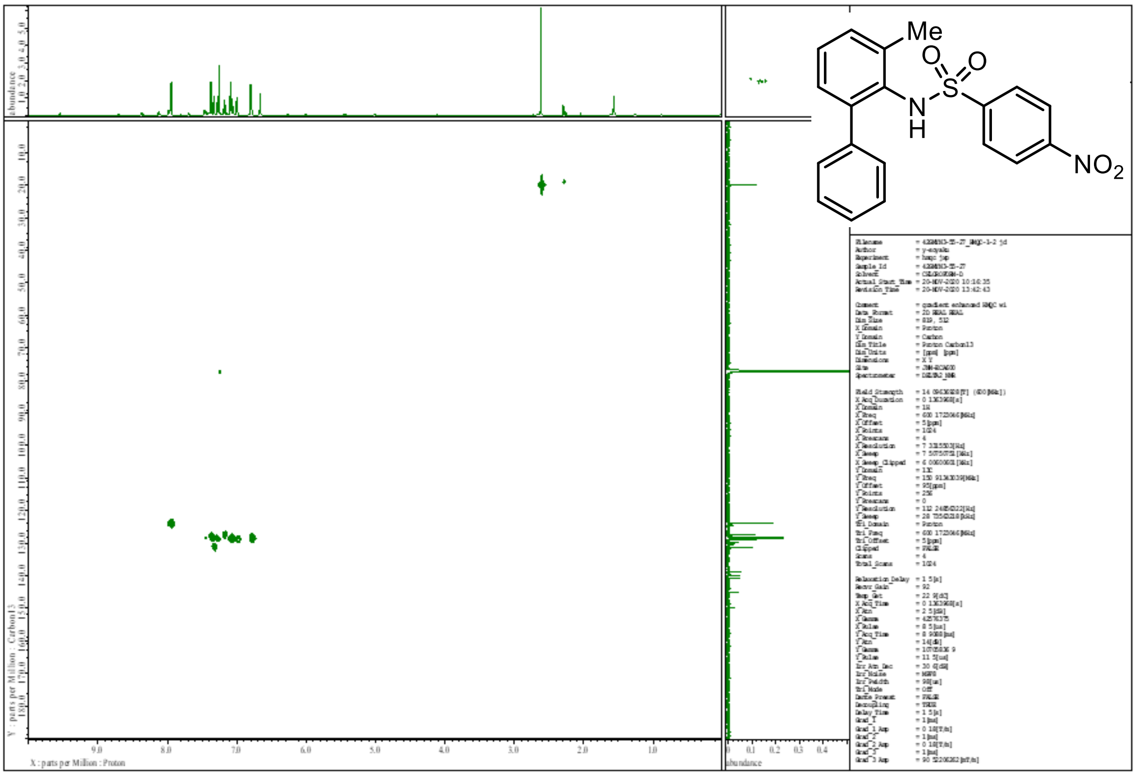

^{1}H-NMR (600 MHz, $CDCl_3$) of **S10c**

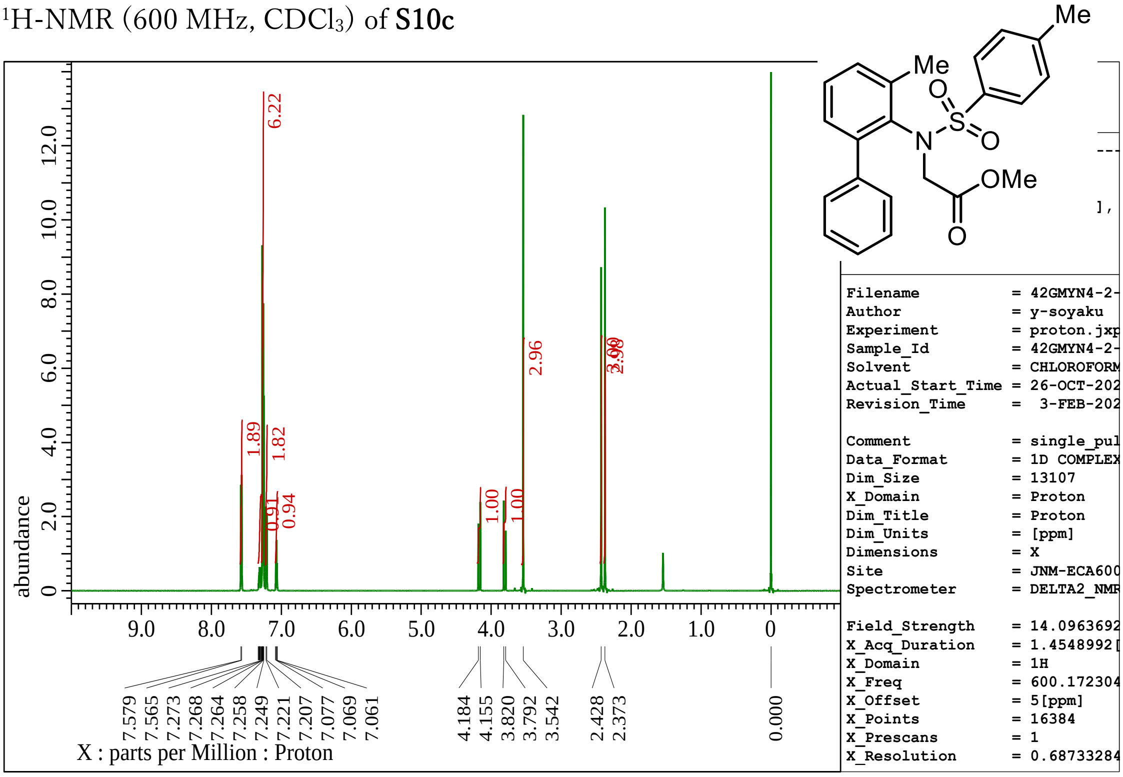

^{13}C{^{1}H }-NMR (150 MHz, $CDCl_3$) of **S10c**

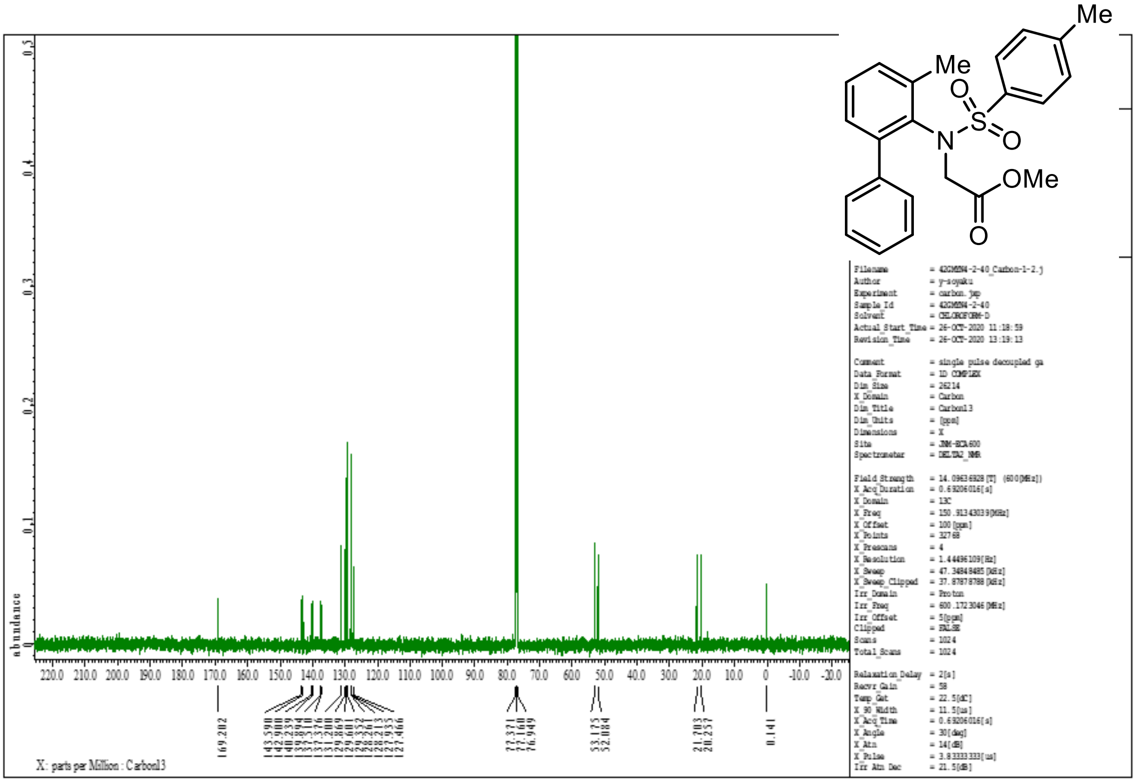

H-H COSY-NMR (600 MHz, $CDCl_3$) of **S10c**

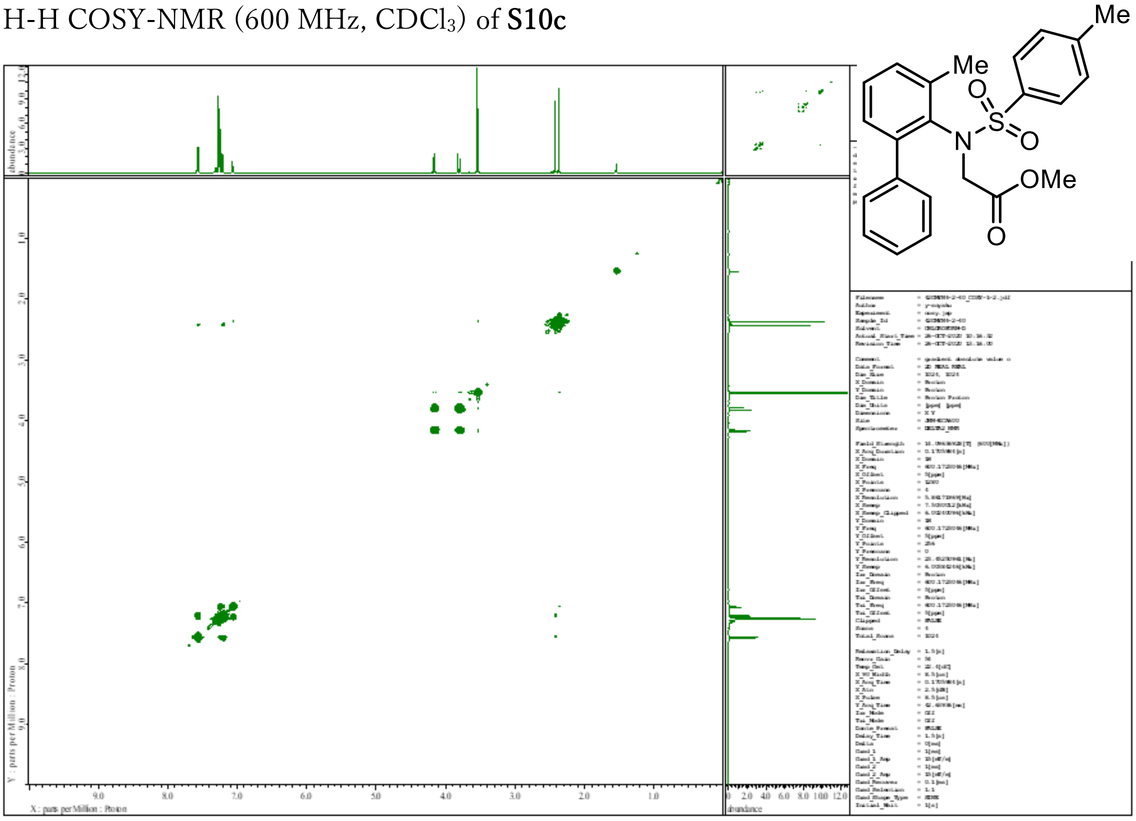

HMQC-NMR ($CDCl_3$) of **S10c**

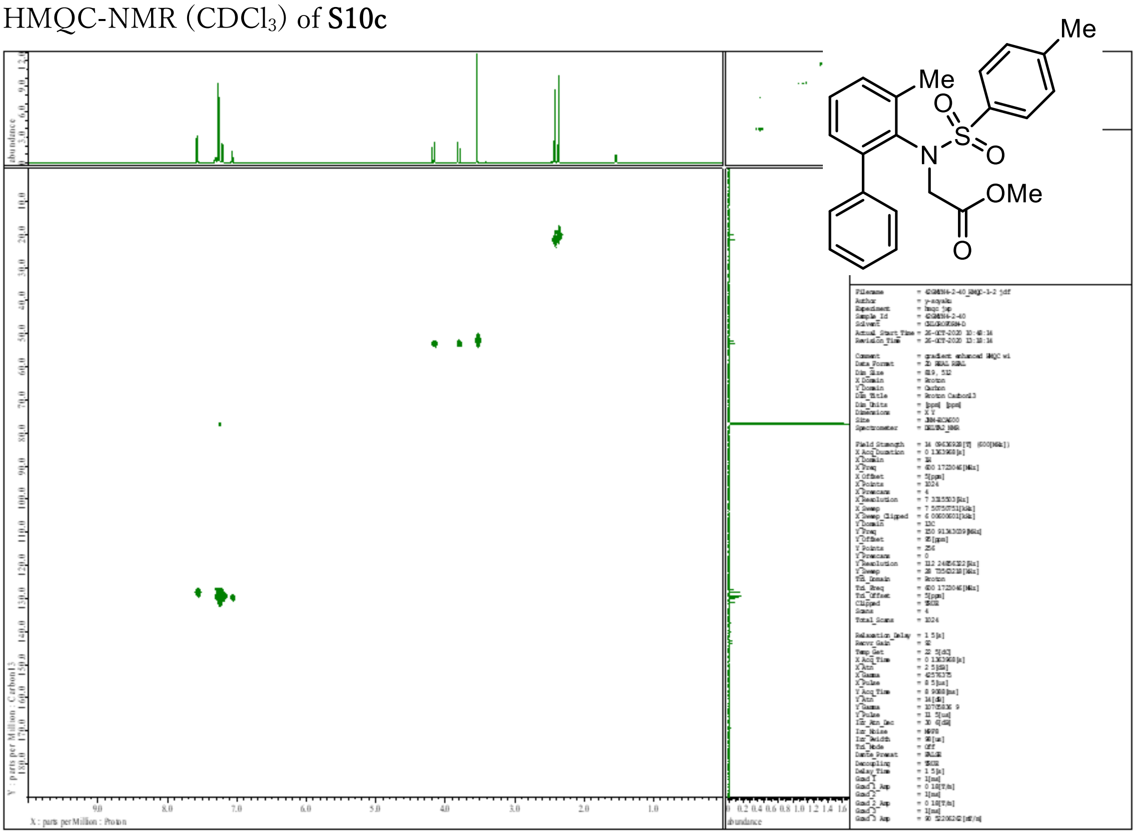

^{1}H-NMR (600 MHz, $CDCl_3$) of **S10d**

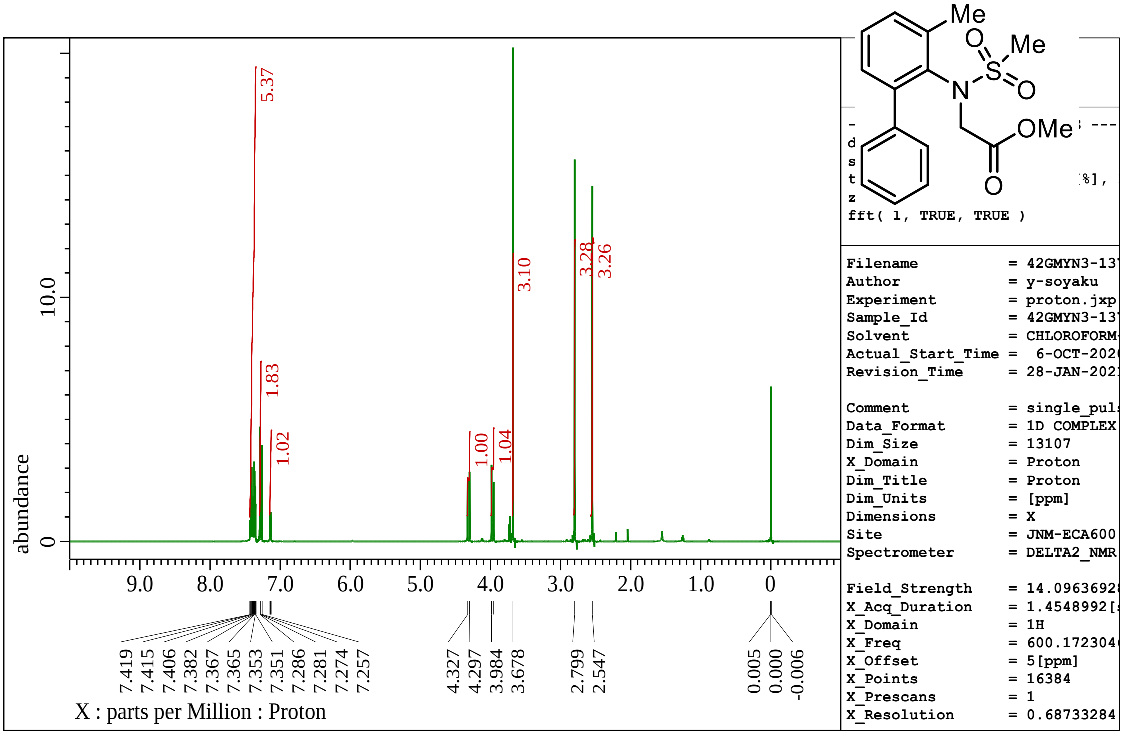

^{13}C{^{1}H }-NMR (150 MHz, $CDCl_3$) of **S10d**

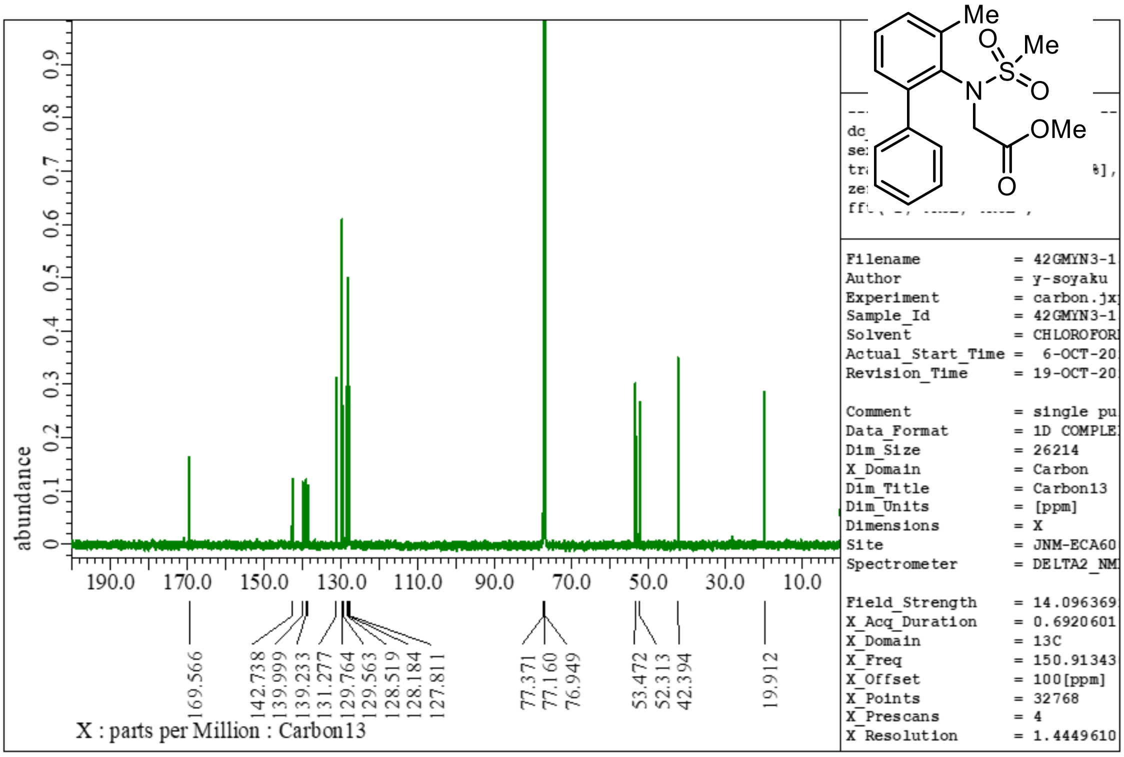

H-H COSY-NMR (600 MHz, $CDCl_3$) of **S10d**

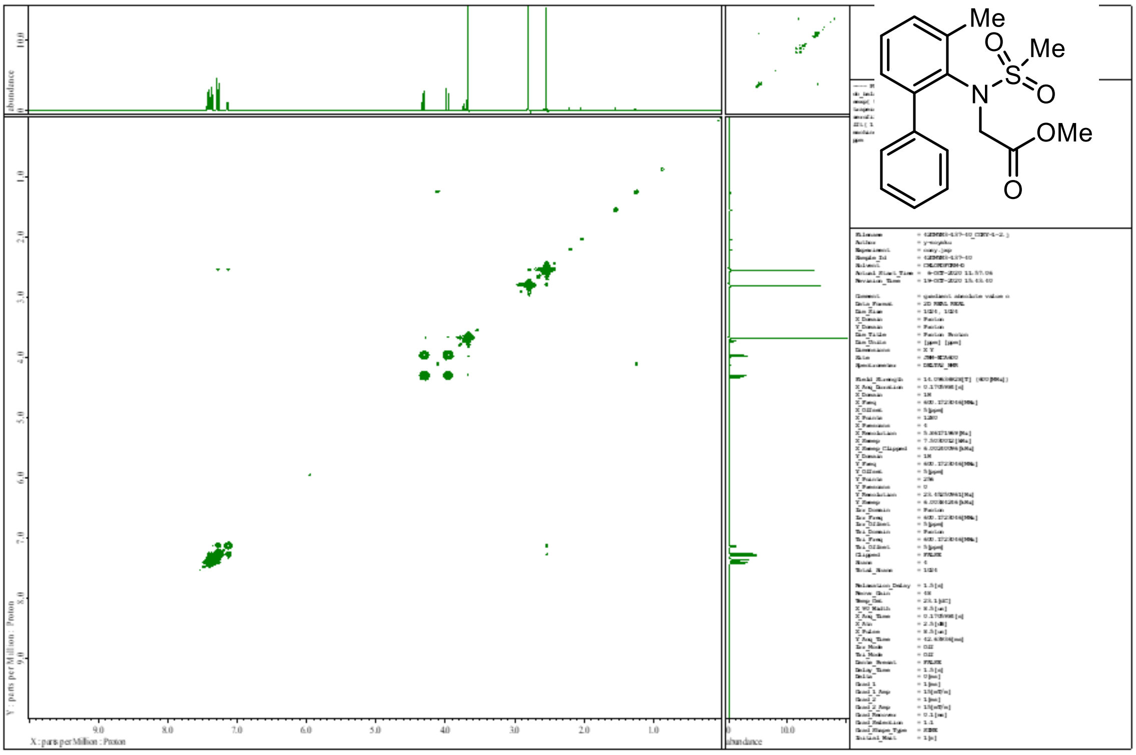

HMQC-NMR ($CDCl_3$) of **S10d**

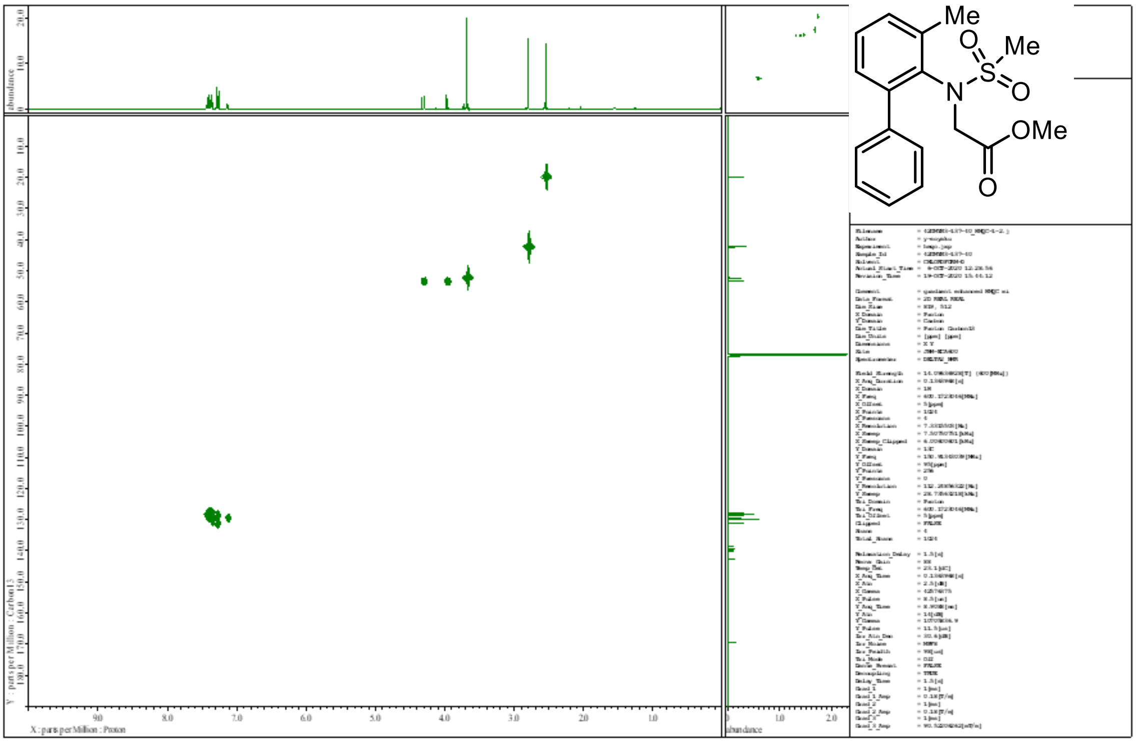

^{1}H-NMR (600 MHz, $CDCl_3$) of **S10e**

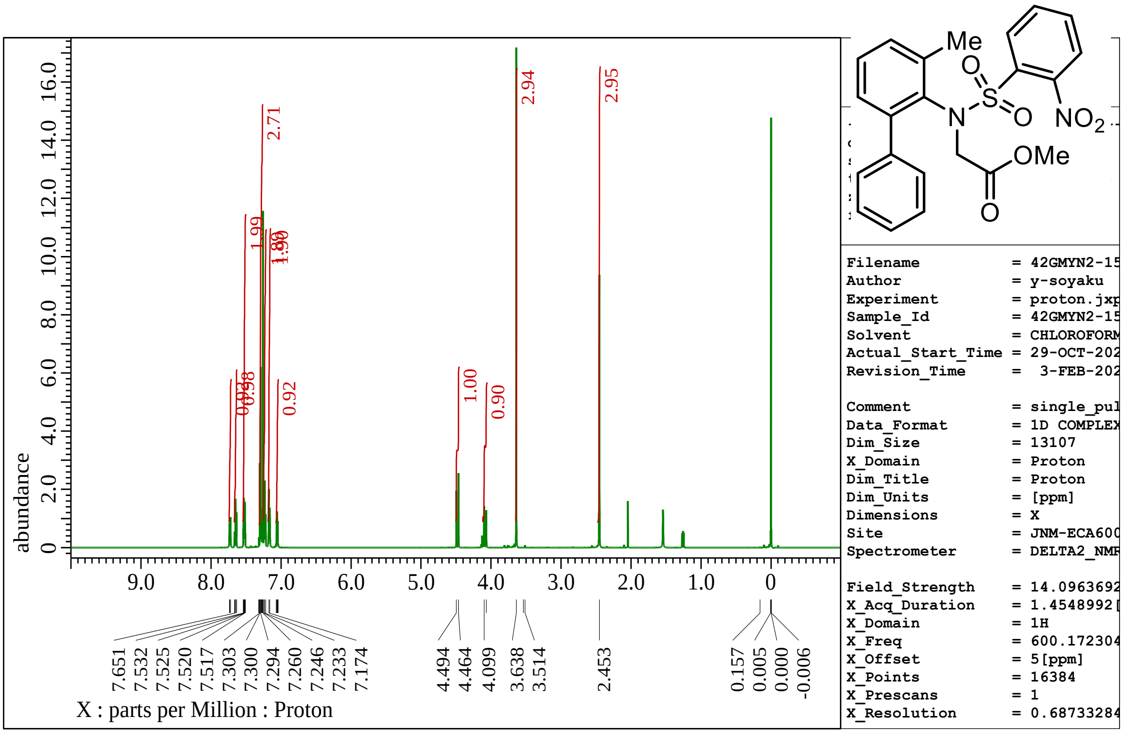

$^{13}C\{^{1}H\}$-NMR (150 MHz, $CDCl_3$) of **S10e**

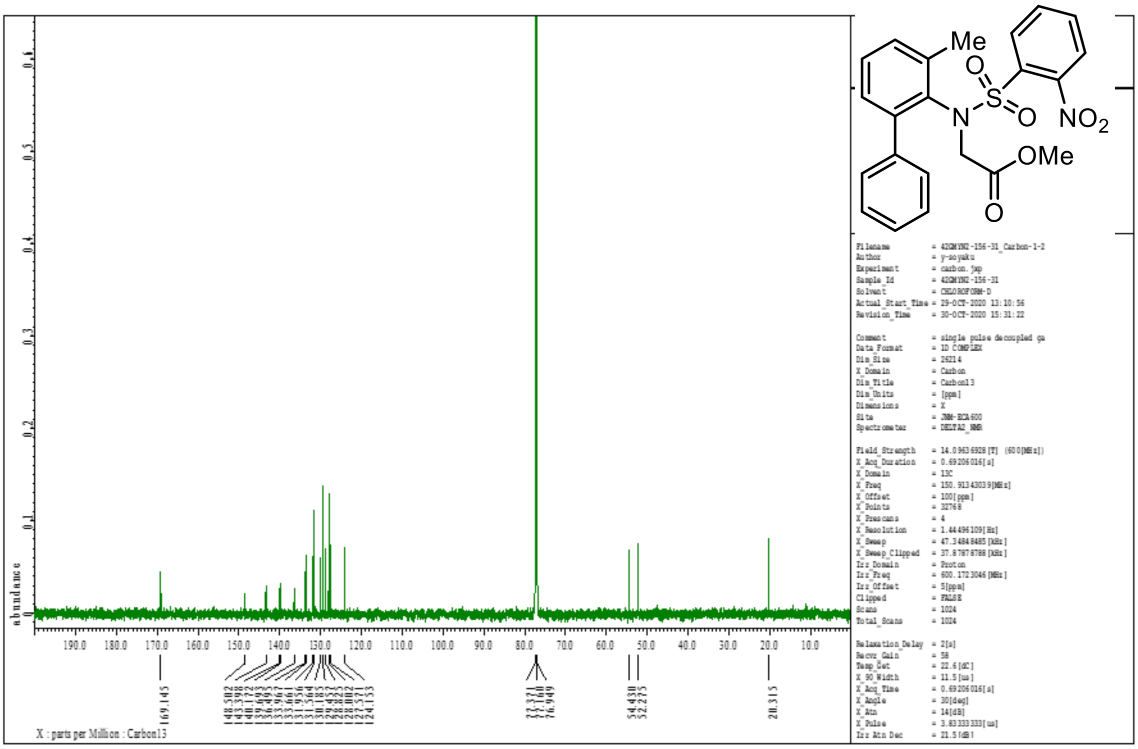

H-H COSY-NMR (600 MHz, $CDCl_3$) of **S10e**

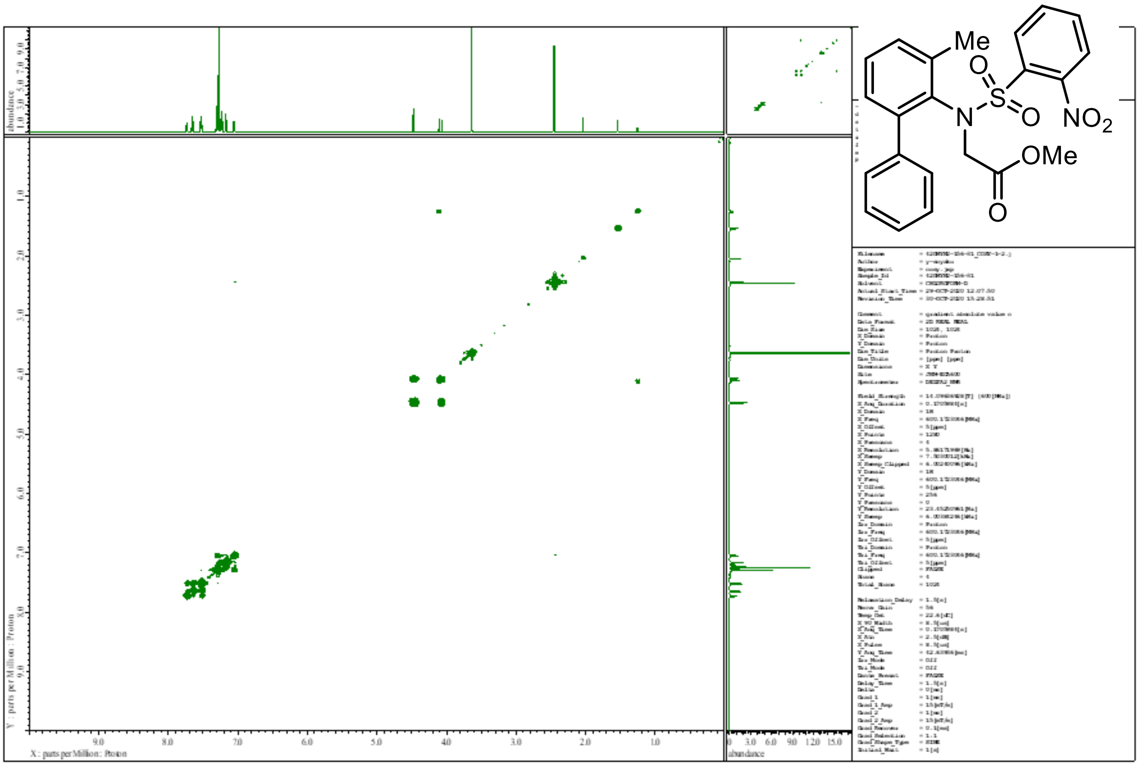

HMQC-NMR ($CDCl_3$) of **S10e**

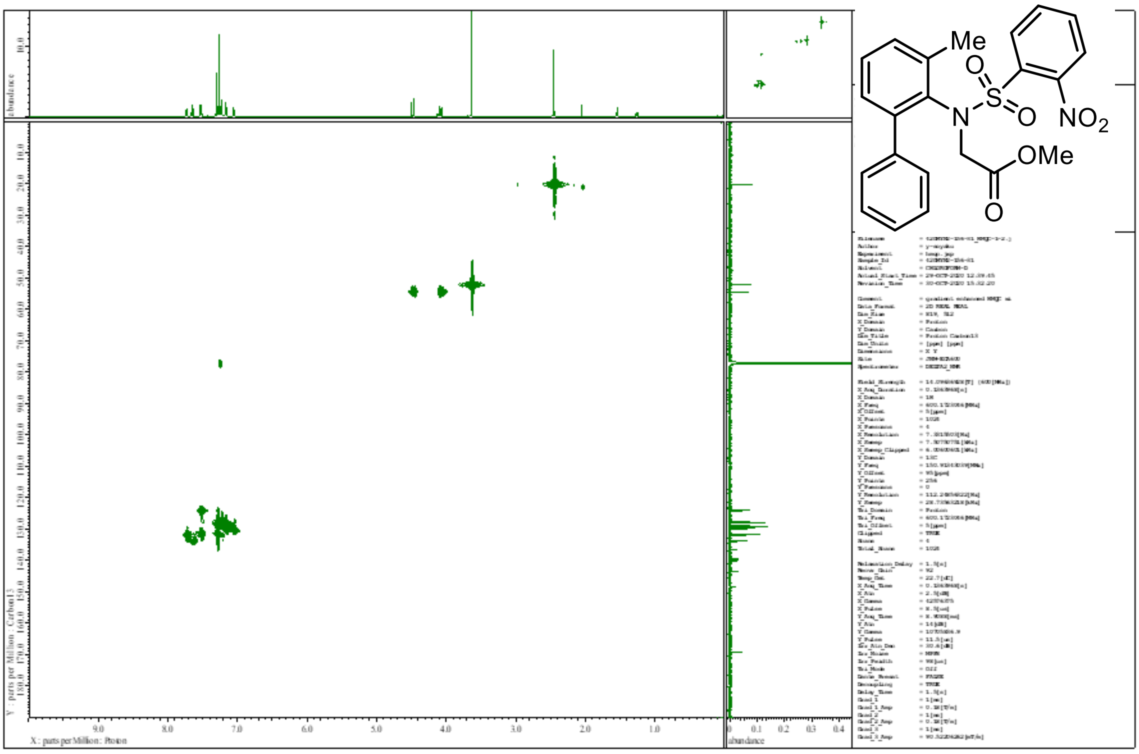

^{1}H-NMR (600 MHz, $CDCl_3$) of **S10f**

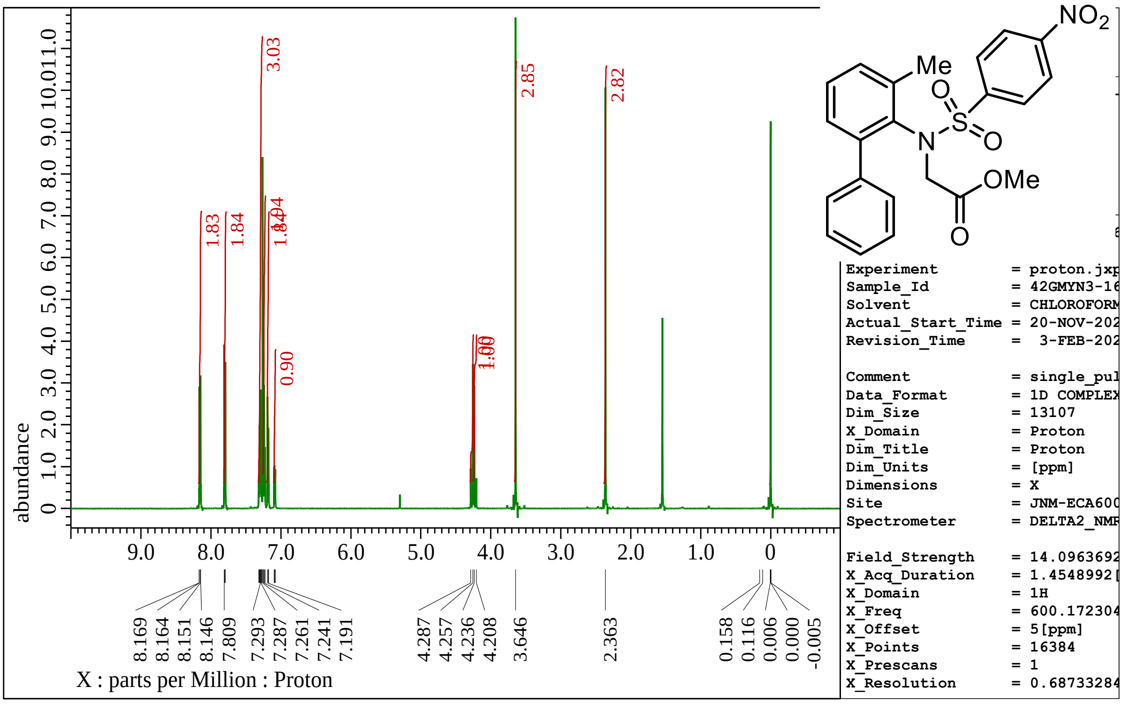

^{13}C{^{1}H}-NMR (150 MHz, $CDCl_3$) of **S10f**

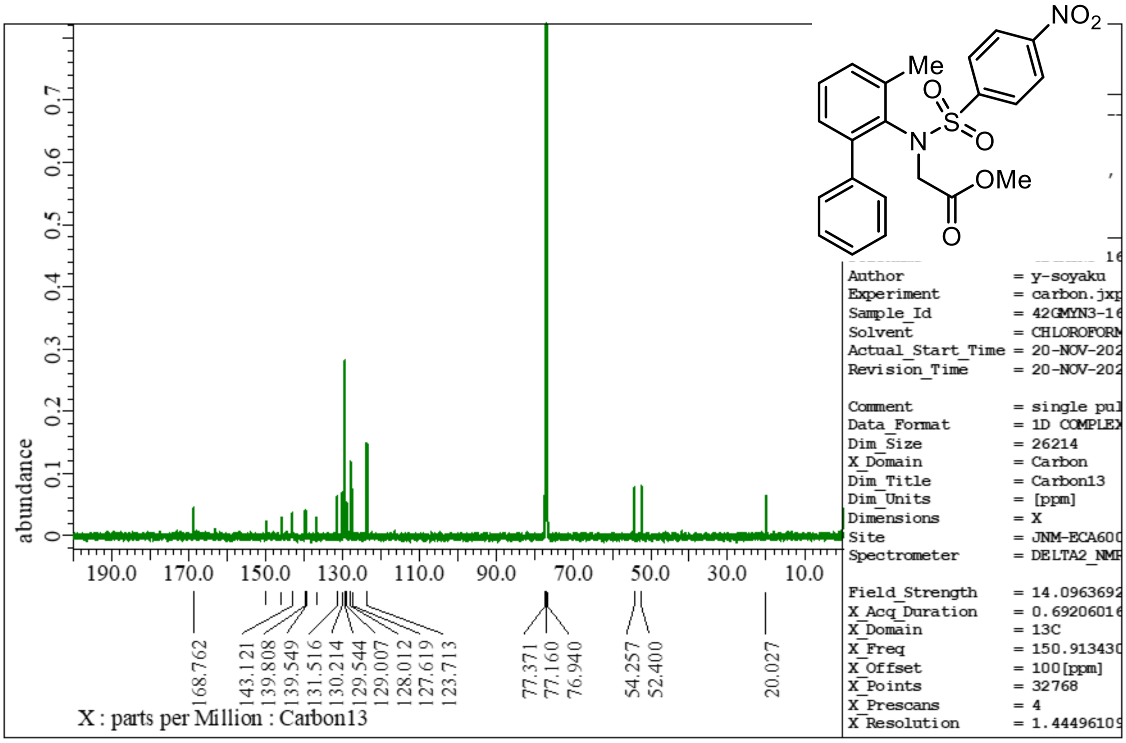

H-H COSY-NMR (600 MHz, $CDCl_3$) of **S10f**

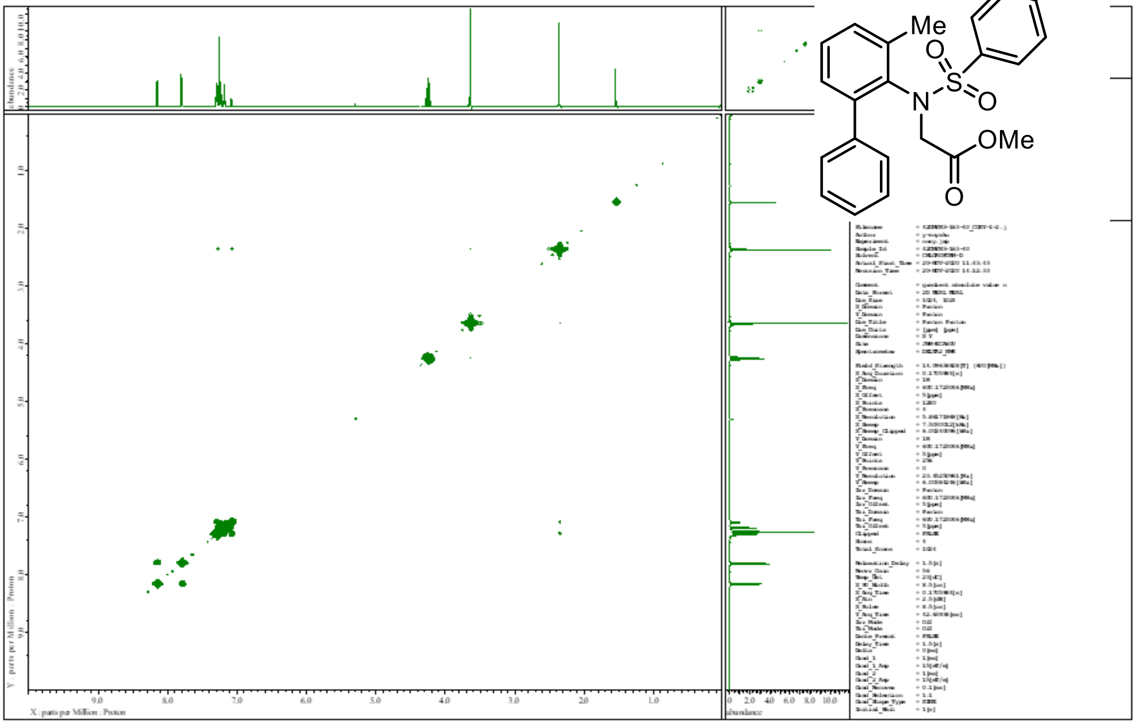

HMQC-NMR ($CDCl_3$) of **S10f**

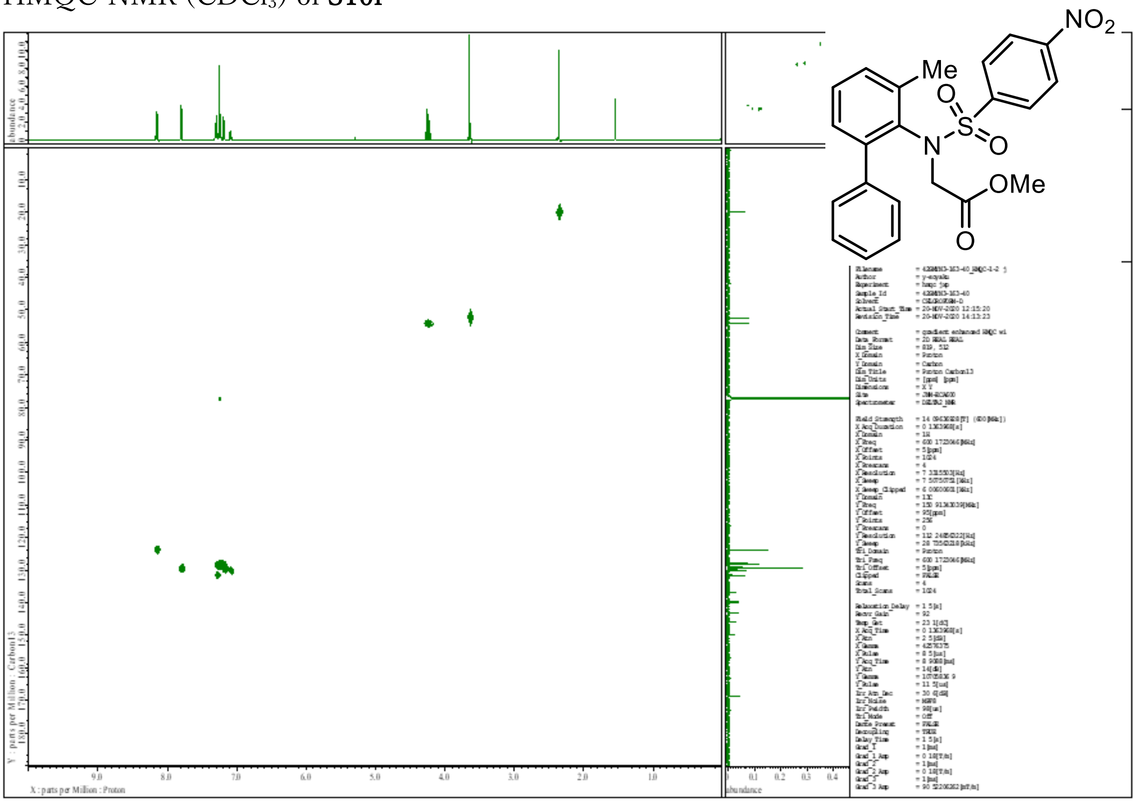

^{1}H-NMR (600 MHz, $CDCl_3$) of **1Aa**

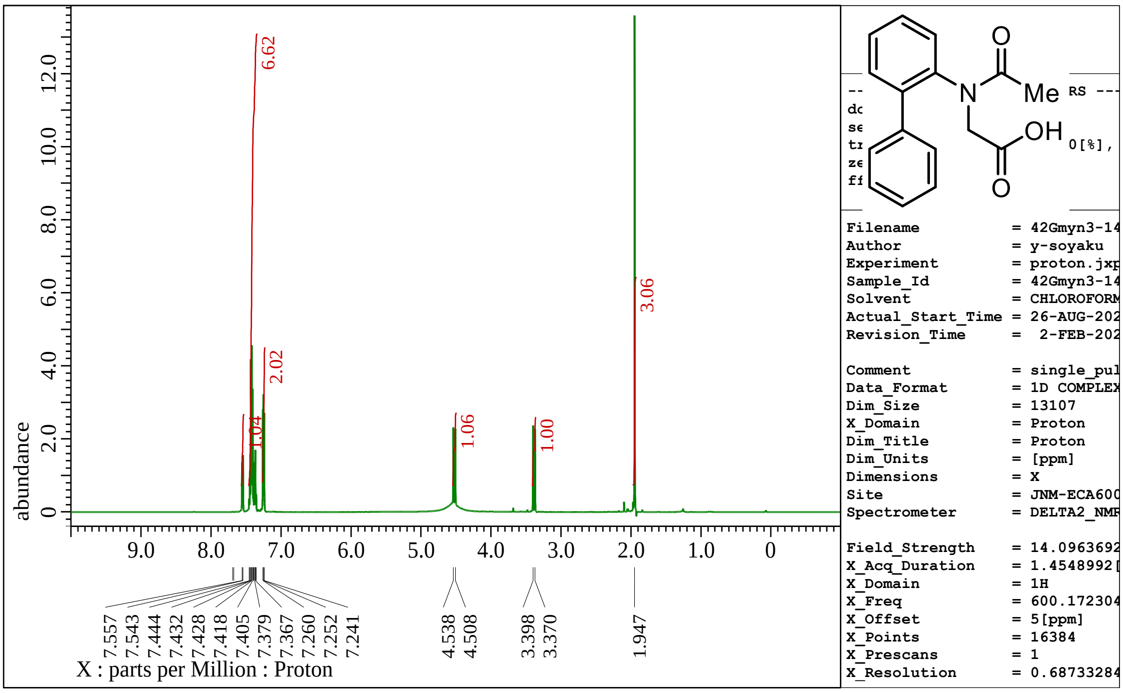

^{13}C{^{1}H }-NMR (150 MHz, $CDCl_3$) of **1Aa**

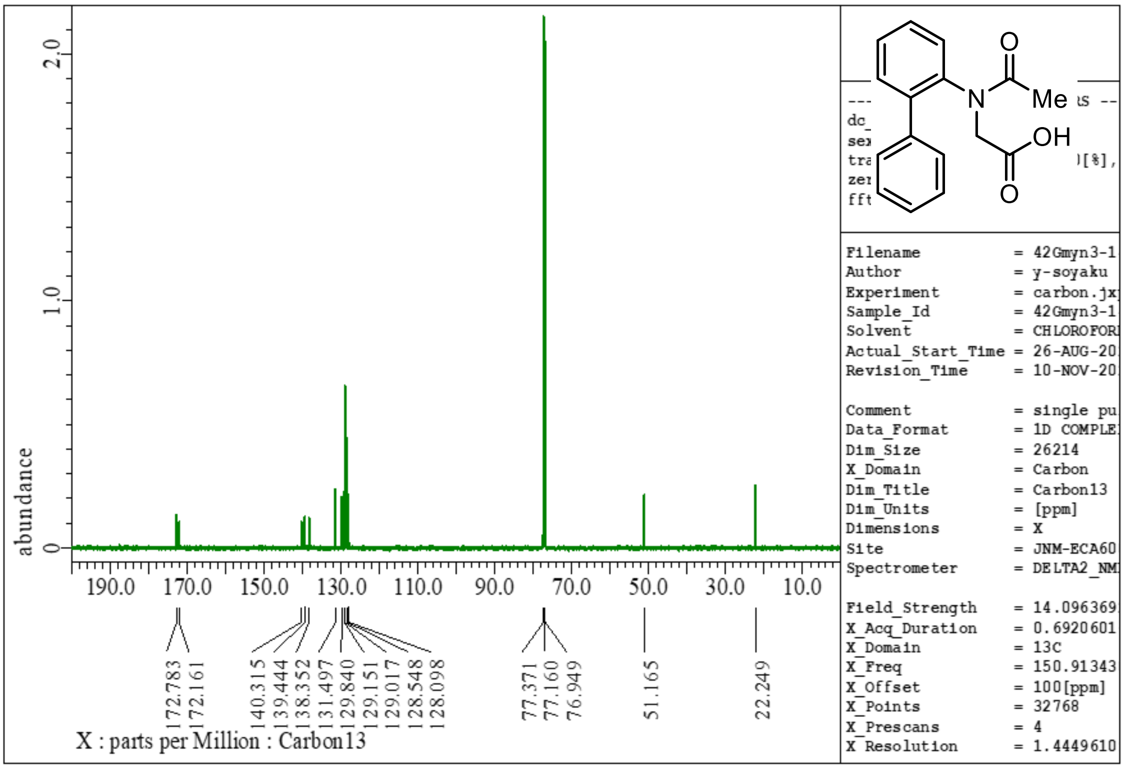

H-H COSY-NMR (600 MHz, $CDCl_3$) of **1Aa**

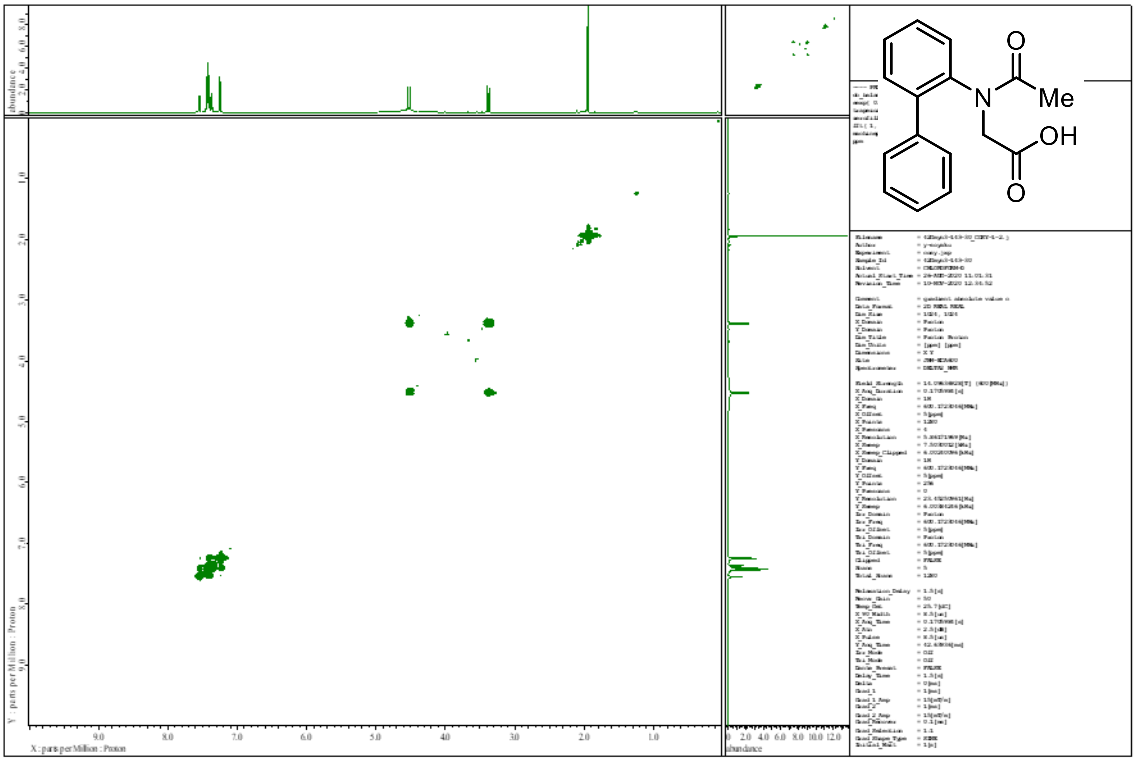

HMQC-NMR ($CDCl_3$) of **1Aa**

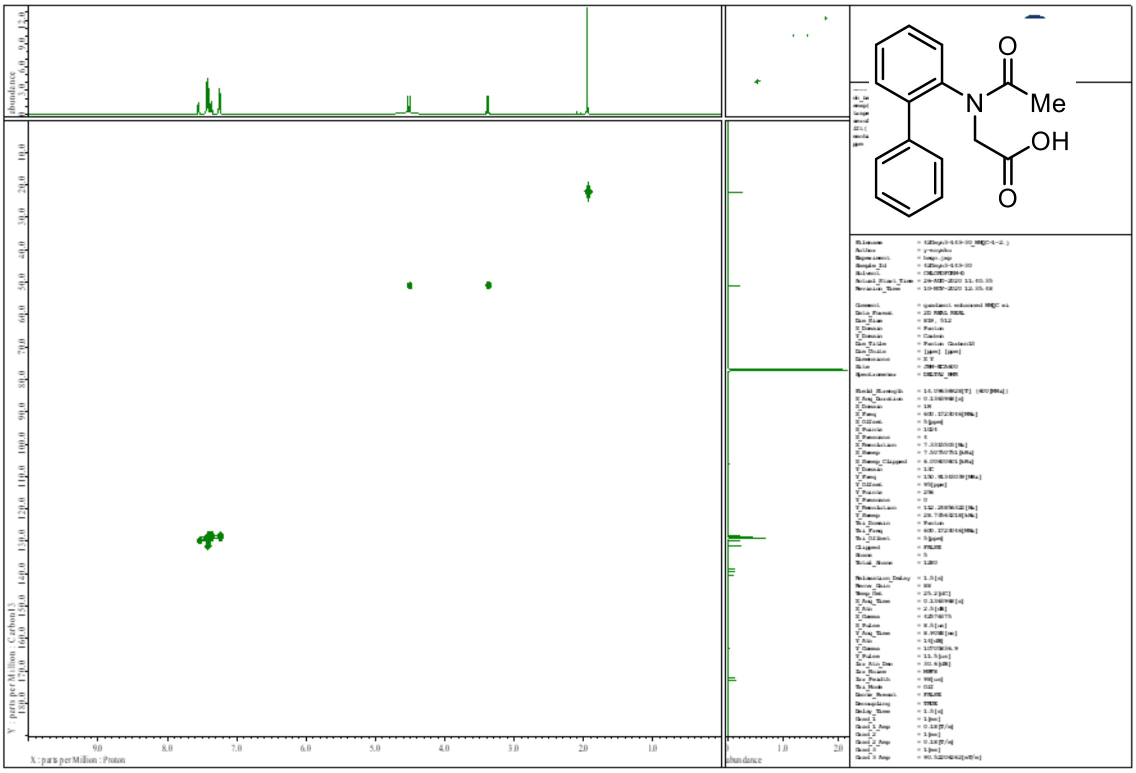

^{1}H-NMR (600 MHz, $CDCl_3$) of **1Ab**

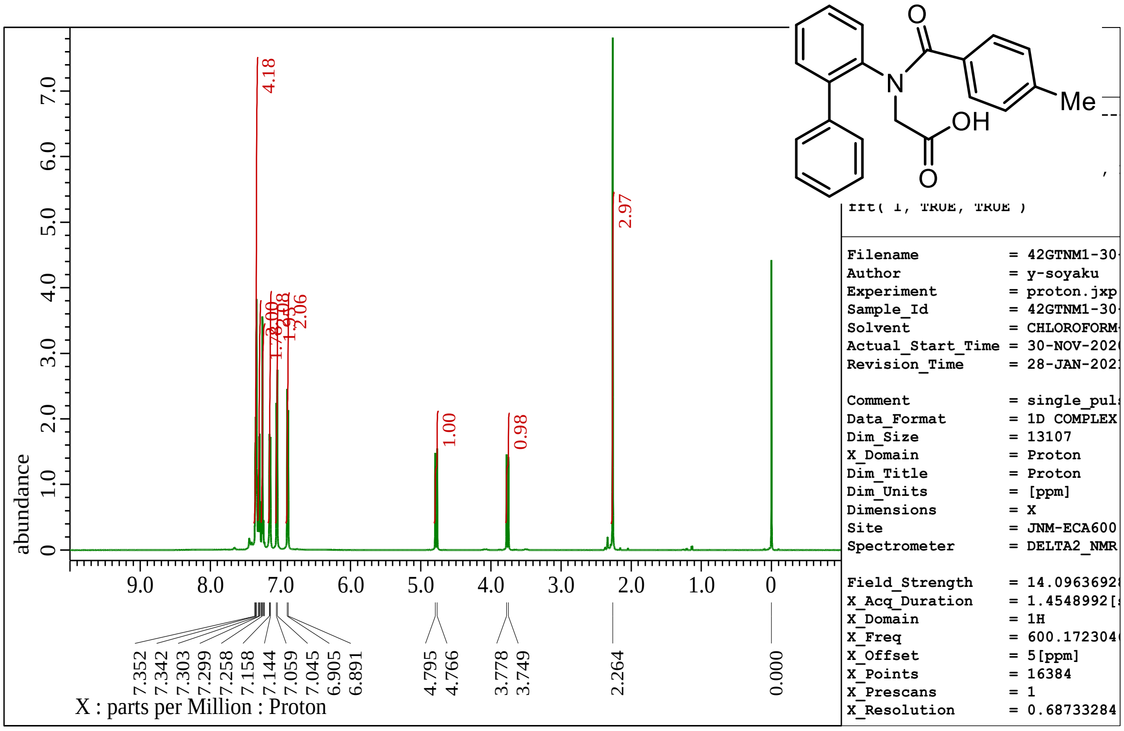

$^{13}C\{^{1}H\}$-NMR (150 MHz, $CDCl_3$) of **1Ab**

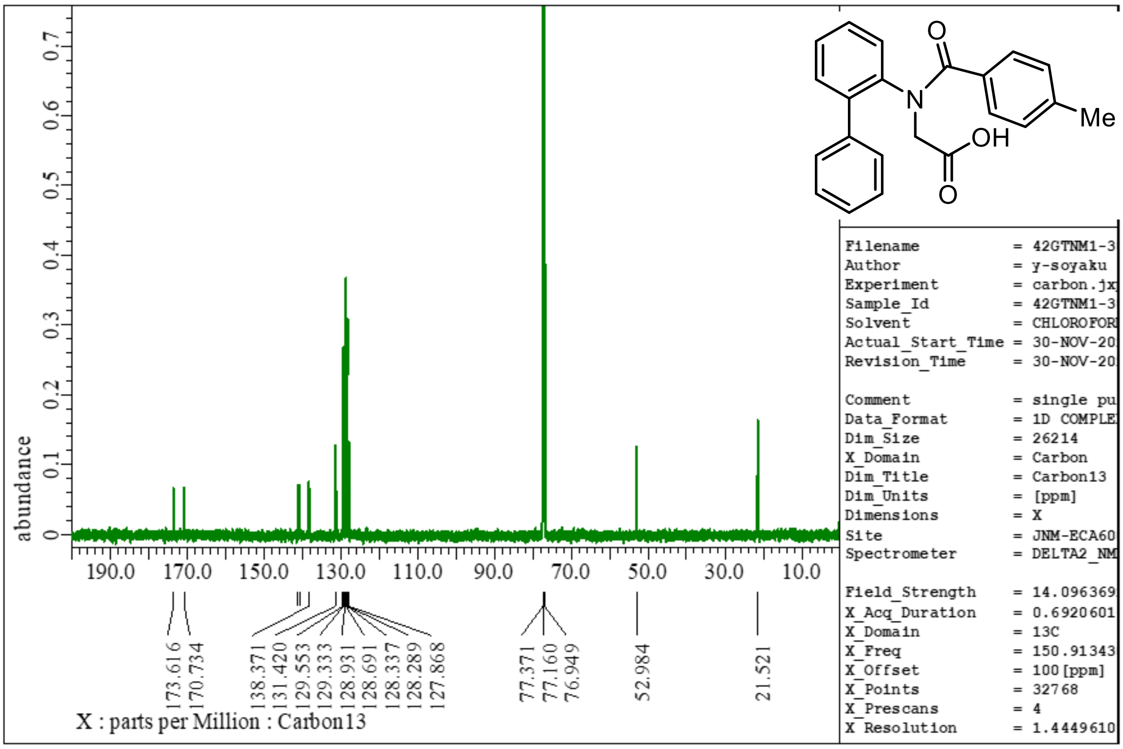

H-H COSY-NMR (600 MHz, $CDCl_3$) of **1Ab**

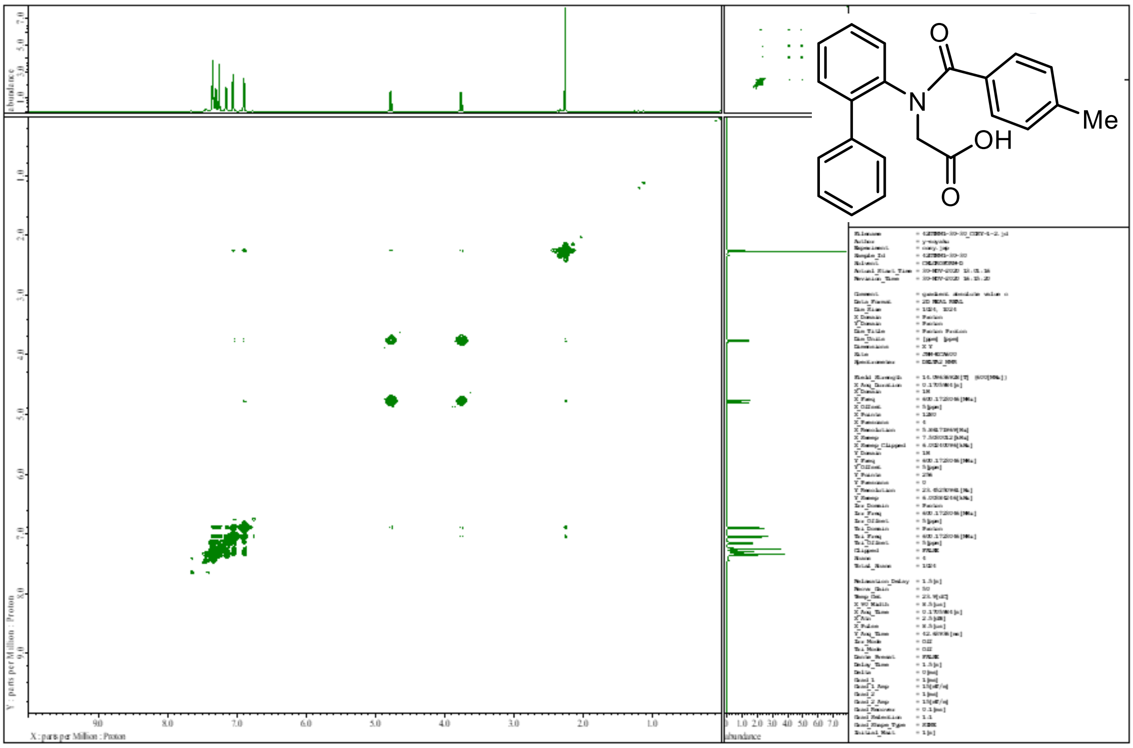

HMQC-NMR ($CDCl_3$) of **1Ab**

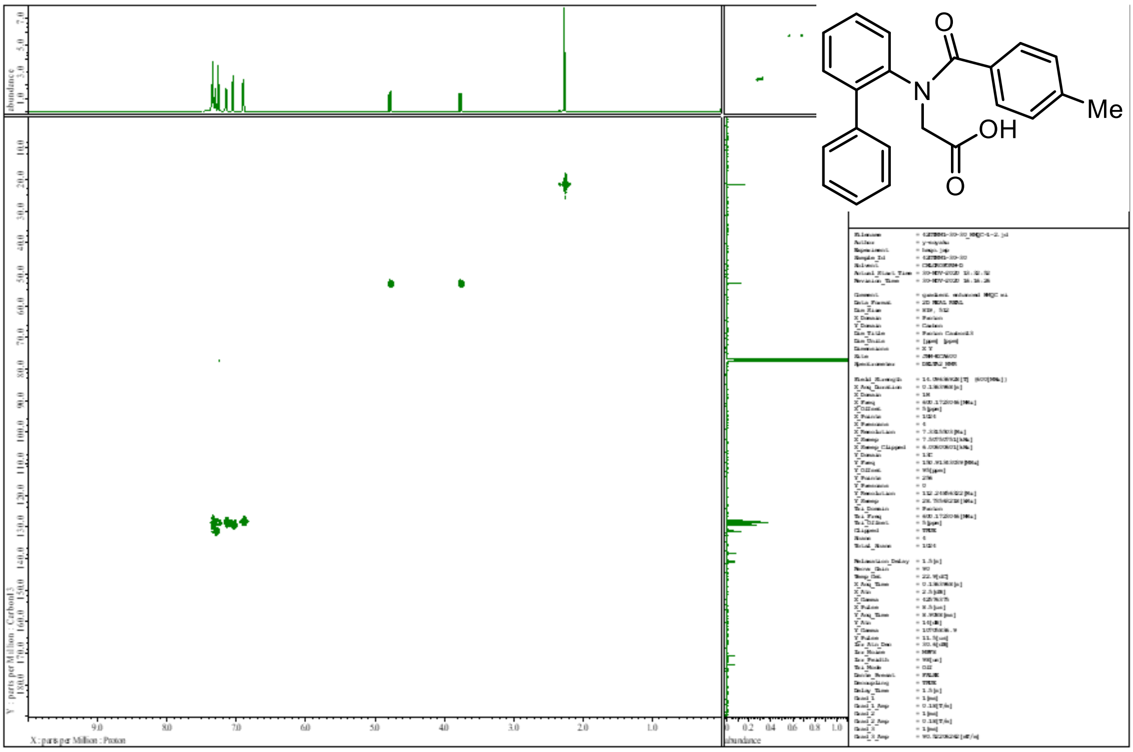

^{1}H-NMR (600 MHz, $CDCl_3$) of **1Ac**

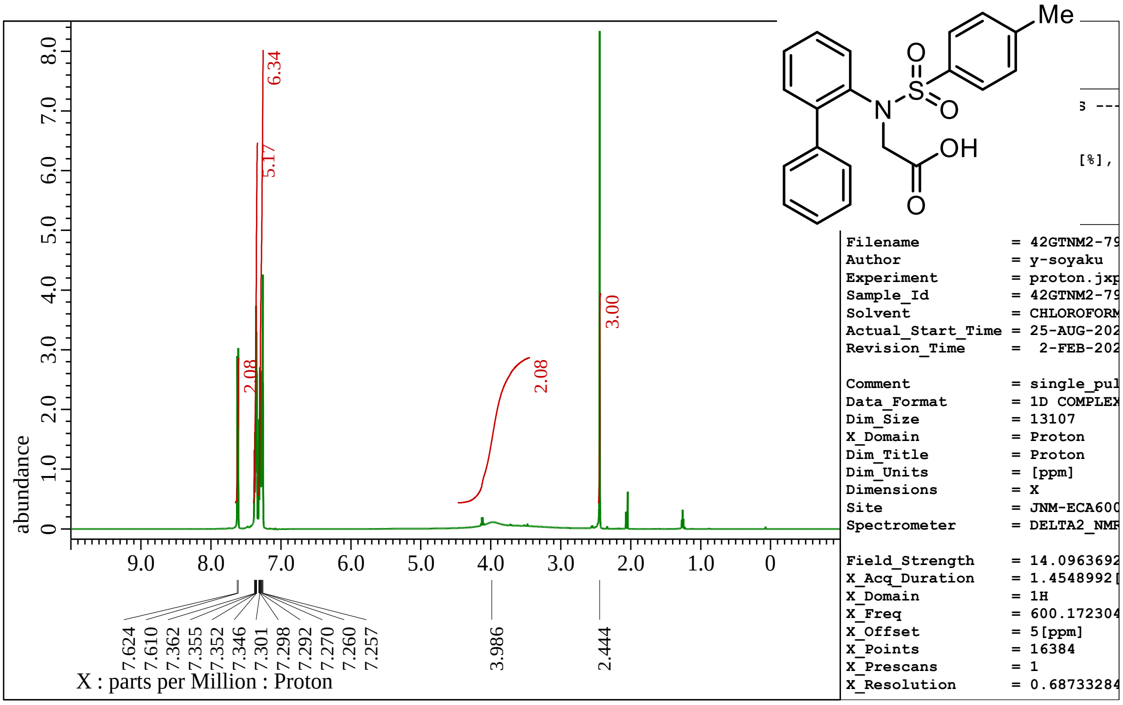

^{13}C{^{1}H }-NMR (150 MHz, $CDCl_3$) of **1Ac**

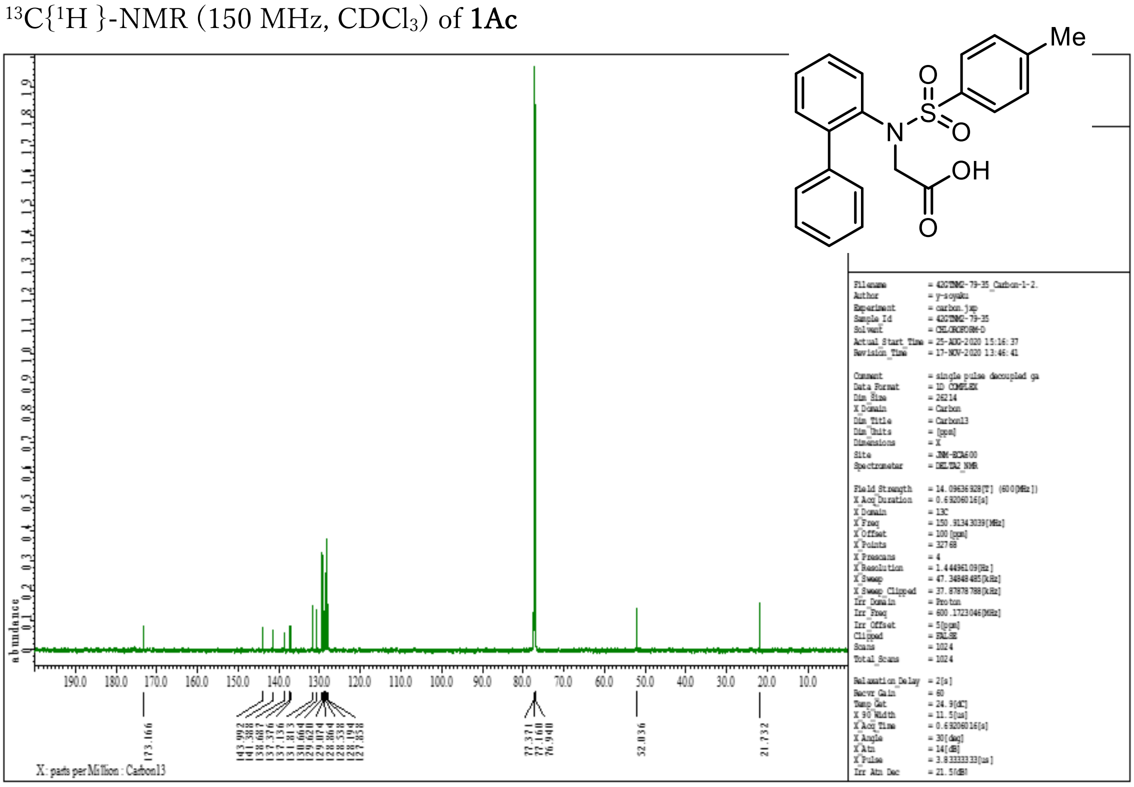

H-H COSY-NMR (600 MHz, $CDCl_3$) of **1Ac**

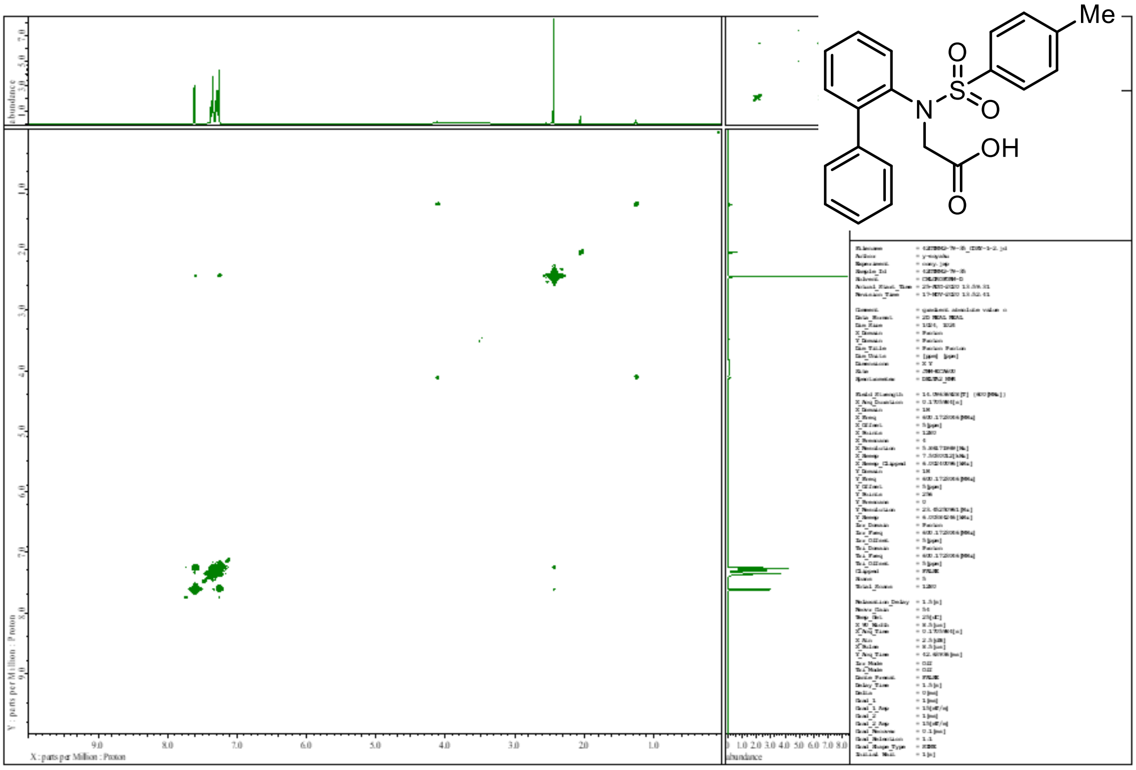

HMQC-NMR ($CDCl_3$) of **1Ac**

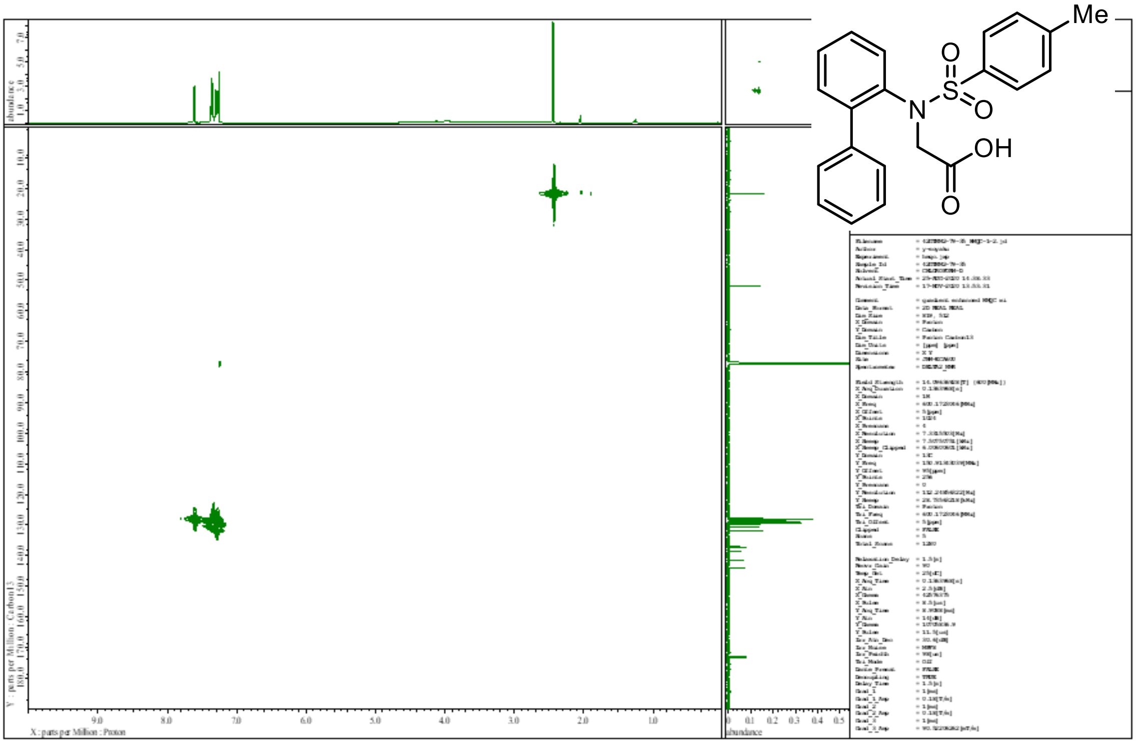

^{1}H-NMR (600 MHz, $CDCl_3$) of **1Ad**

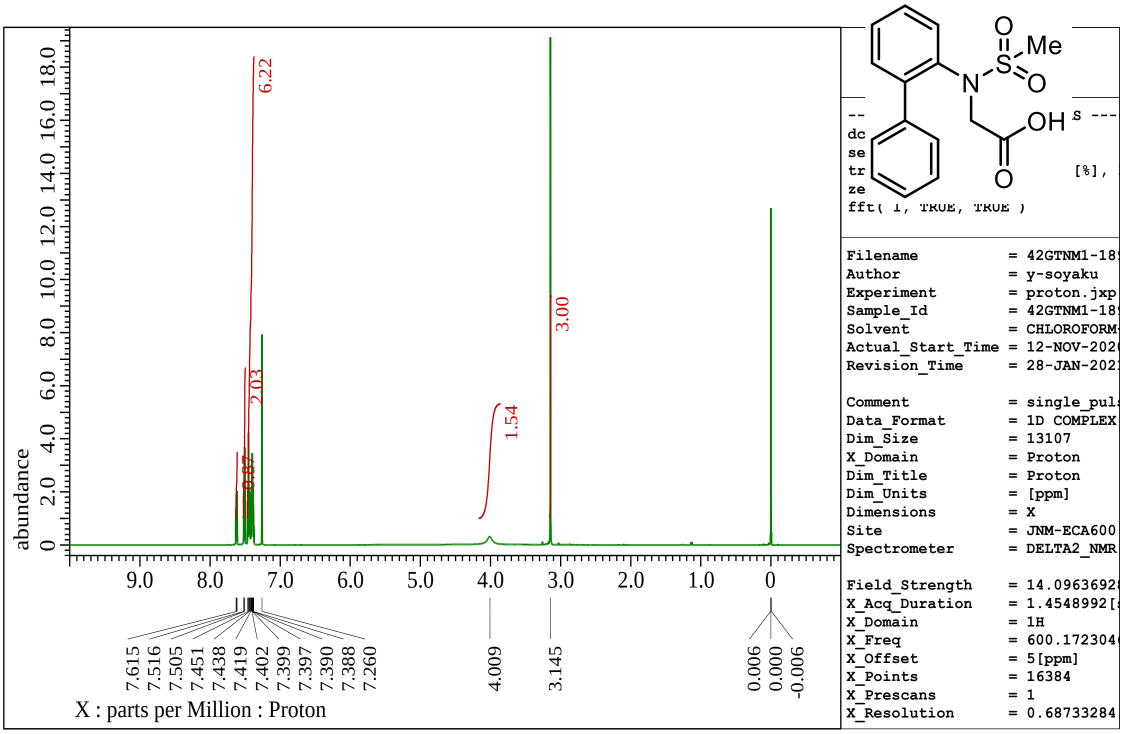

^{13}C{^{1}H }-NMR (150 MHz, $CDCl_3$) of **1Ad**

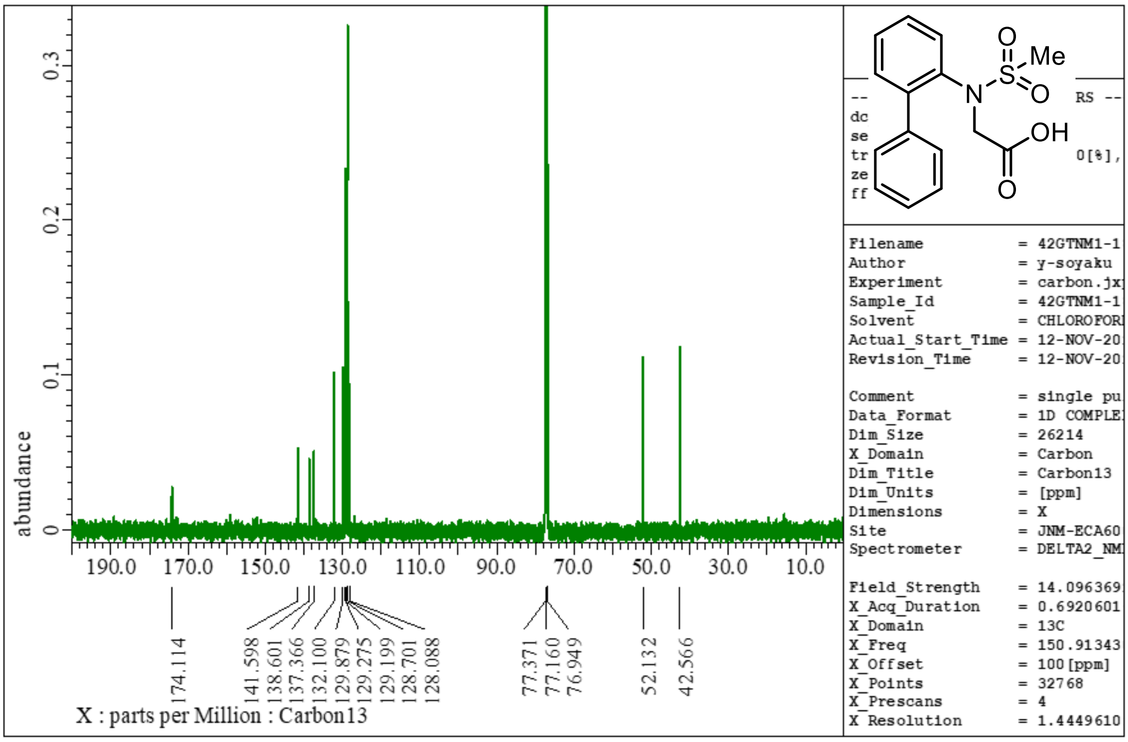

H-H COSY-NMR (600 MHz, $CDCl_3$) of **1Ad**

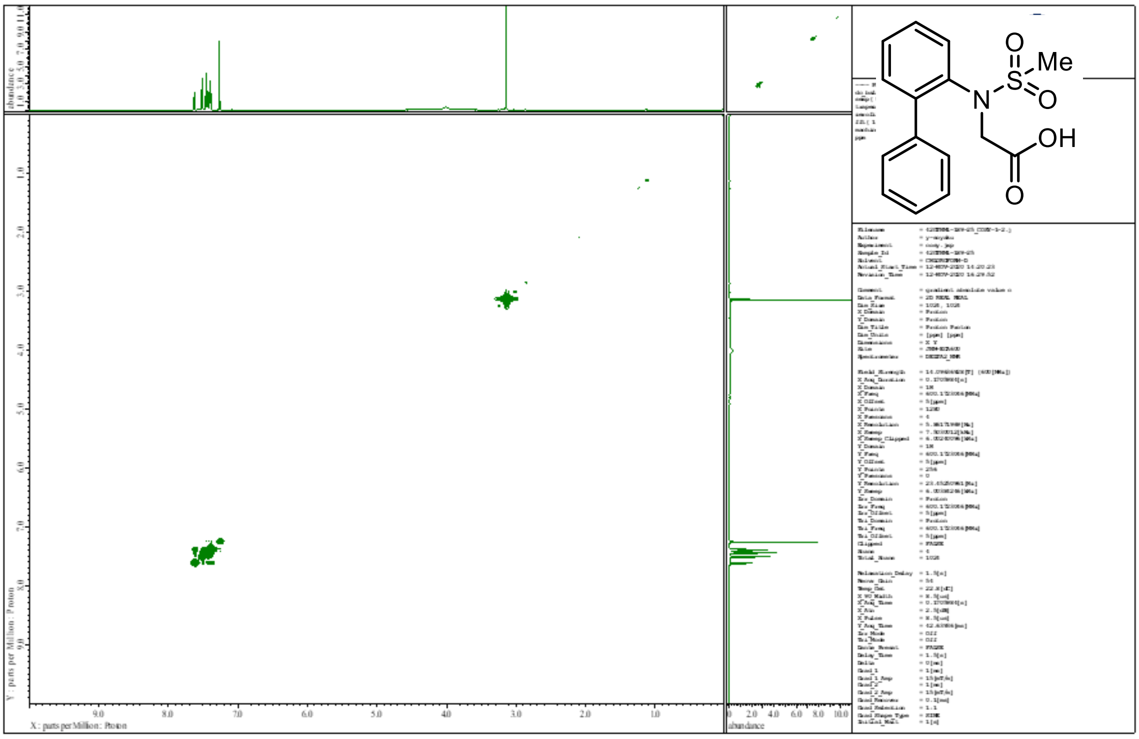

HMQC-NMR ($CDCl_3$) of **1Ad**

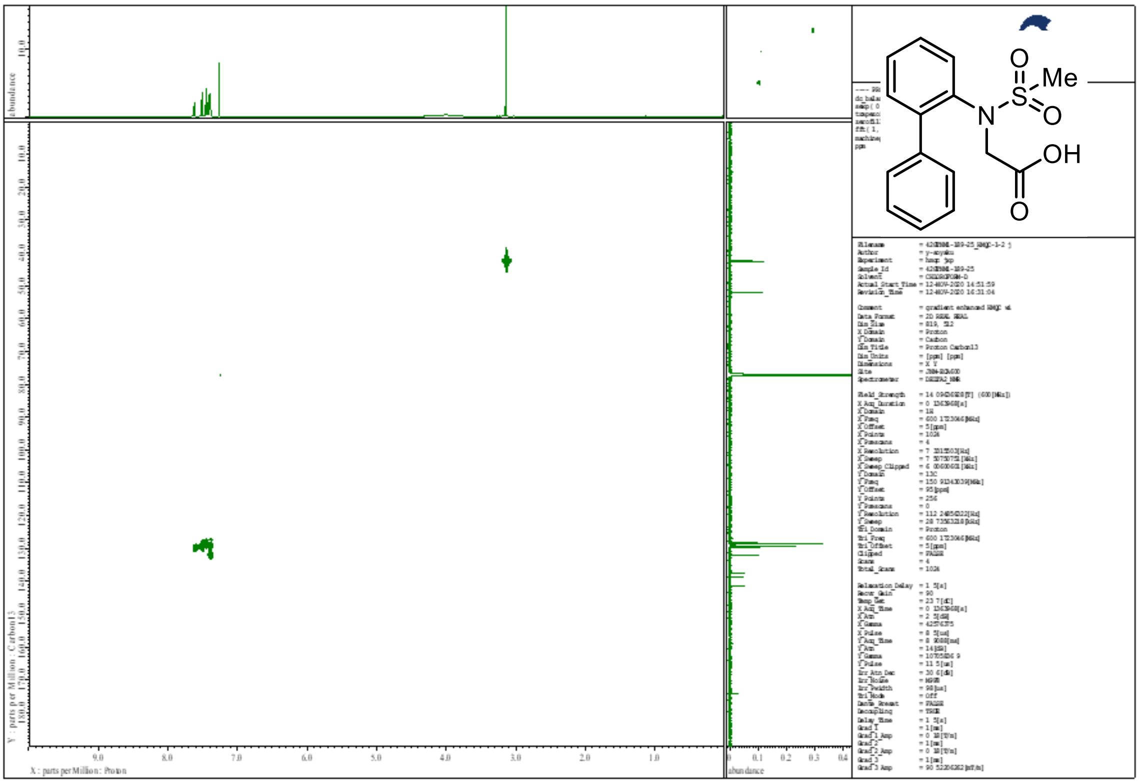

^{1}H-NMR (600 MHz, $CDCl_3$) of **1Ae**

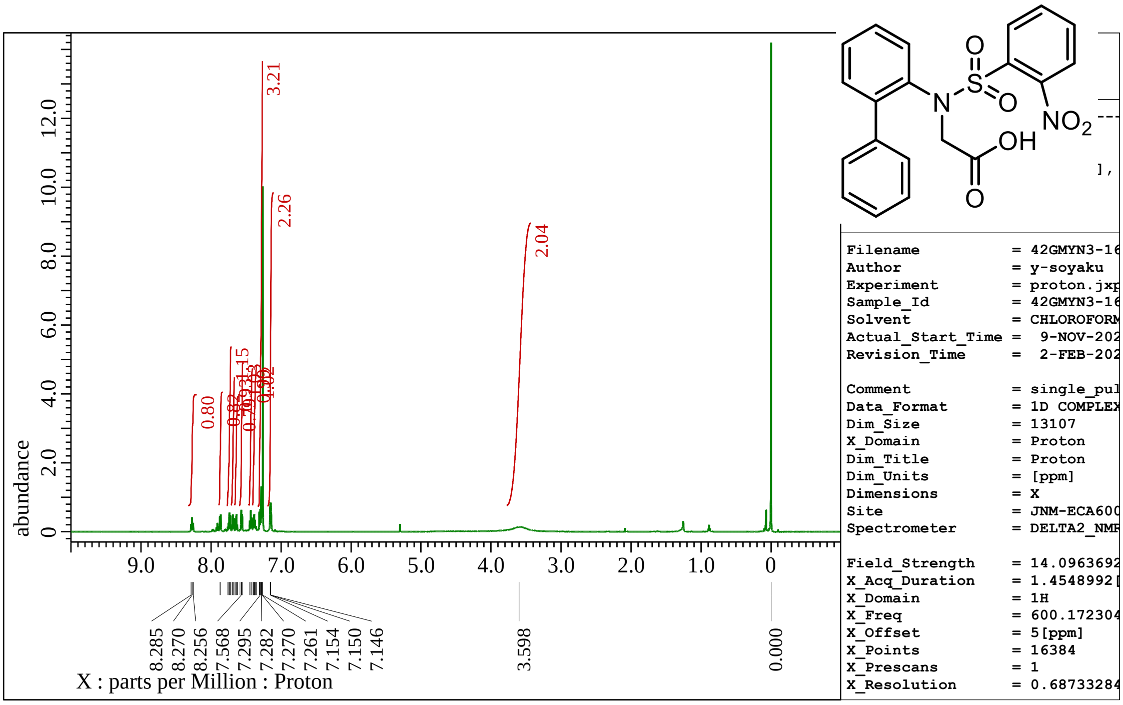

^{13}C{^{1}H }-NMR (150 MHz, $CDCl_3$) of **1Ae**

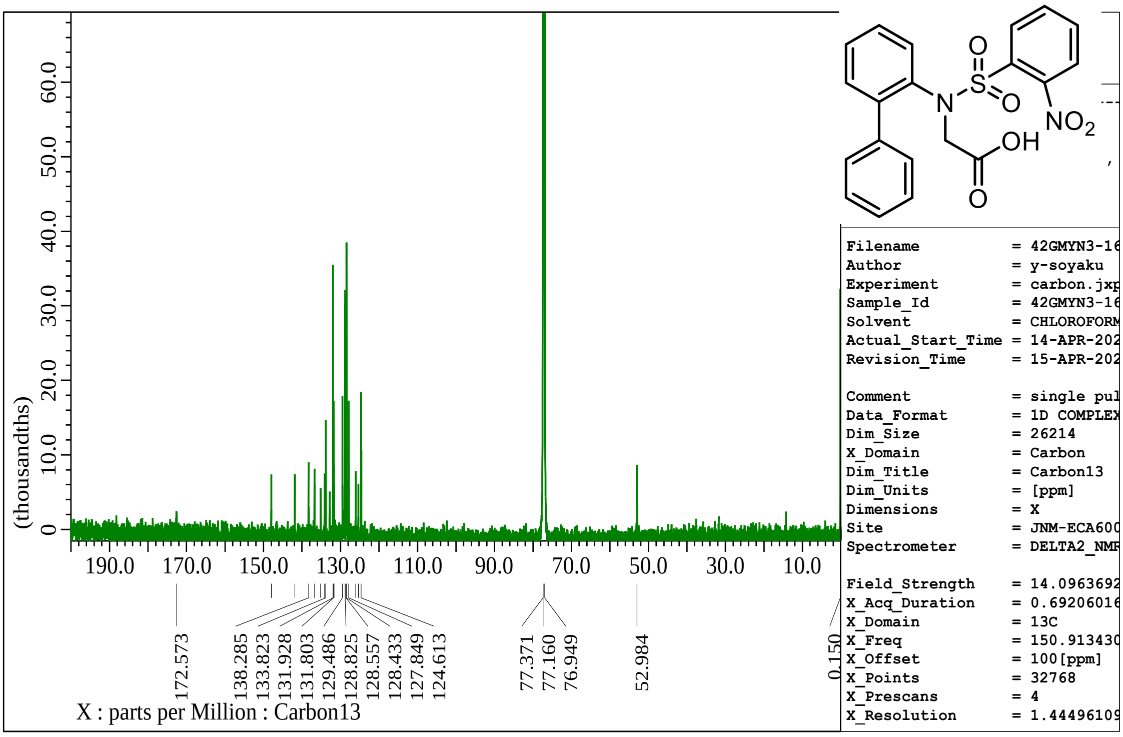

H-H COSY-NMR (600 MHz, $CDCl_3$) of **1Ae**

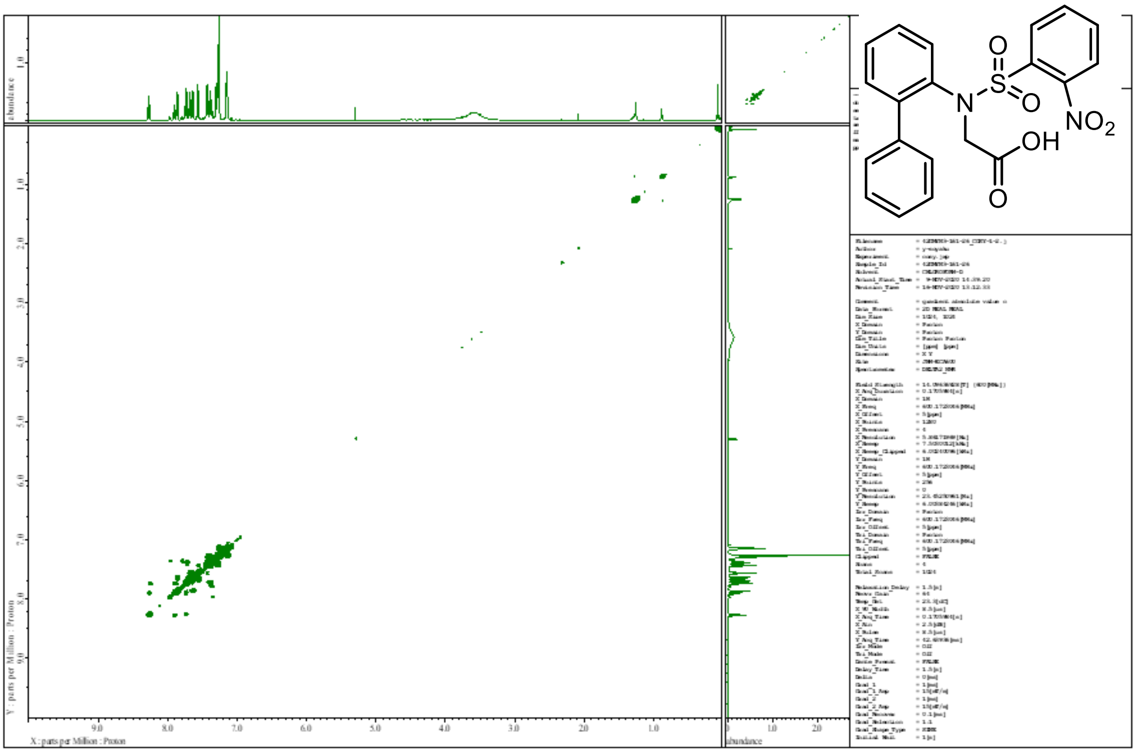

HMQC-NMR ($CDCl_3$) of **1Ae**

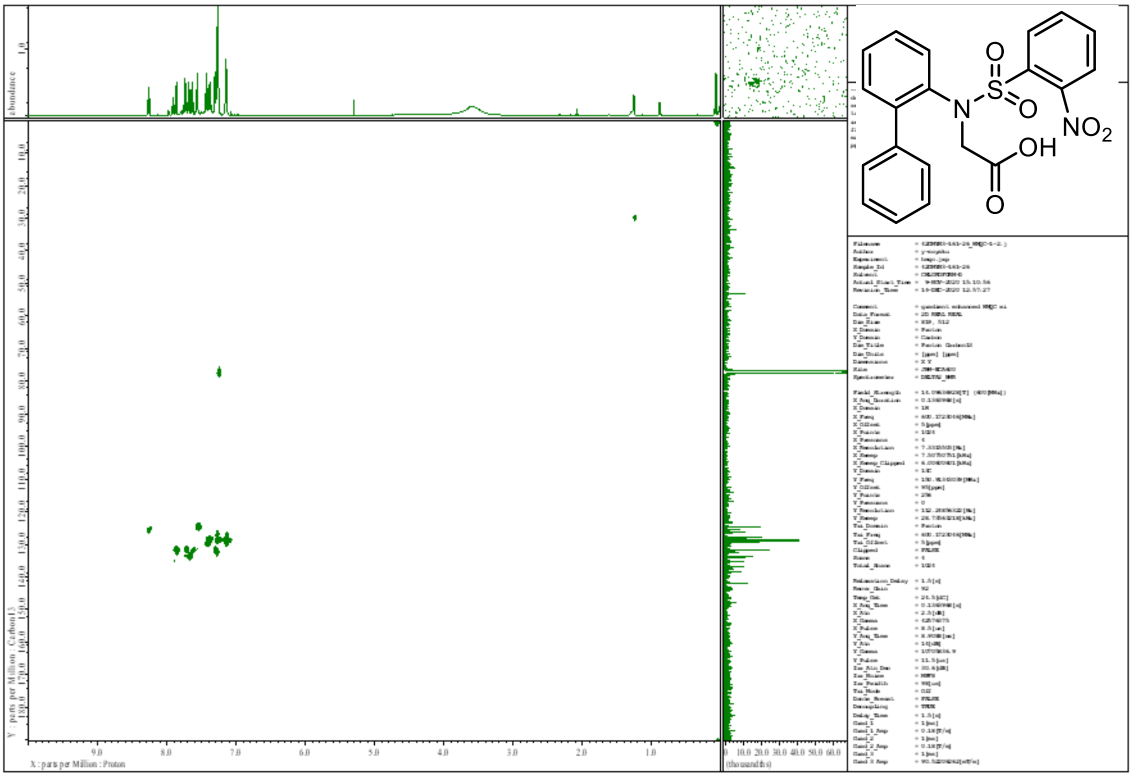

^{1}H-NMR (600 MHz, $CDCl_3$) of **1Af**

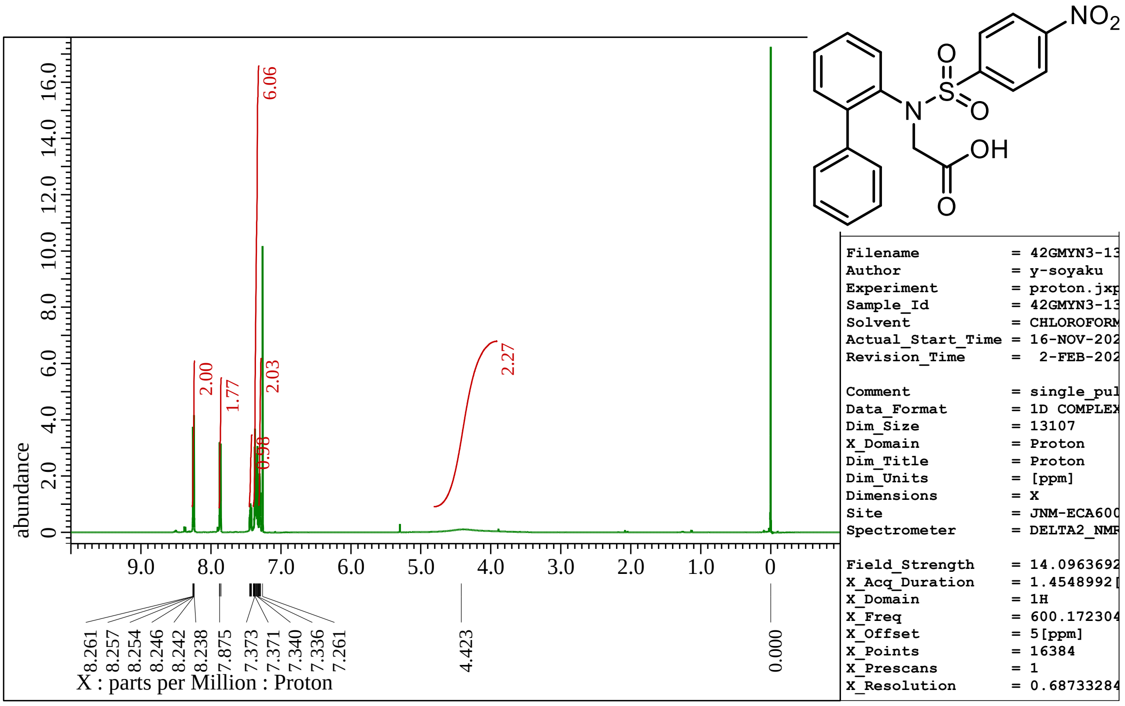

^{13}C{^{1}H }-NMR (150 MHz, $CDCl_3$) of **1Af**

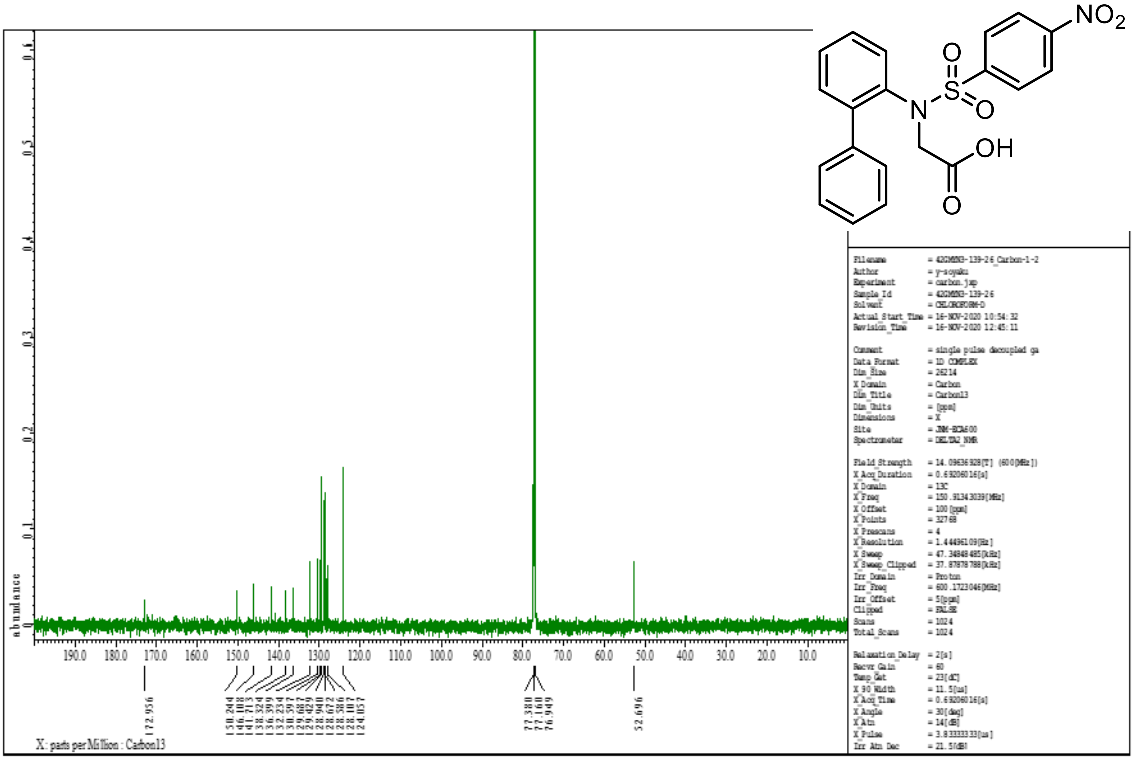

H-H COSY-NMR (600 MHz, $CDCl_3$) of **1Af**

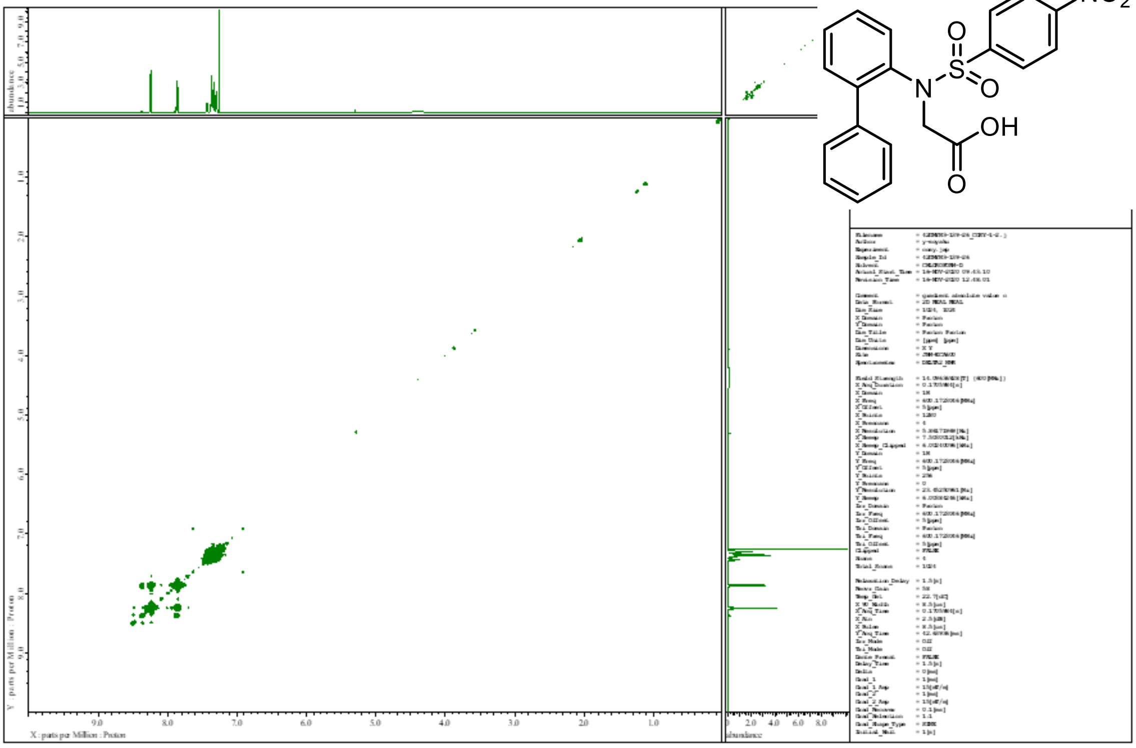

HMQC-NMR ($CDCl_3$) of **1Af**

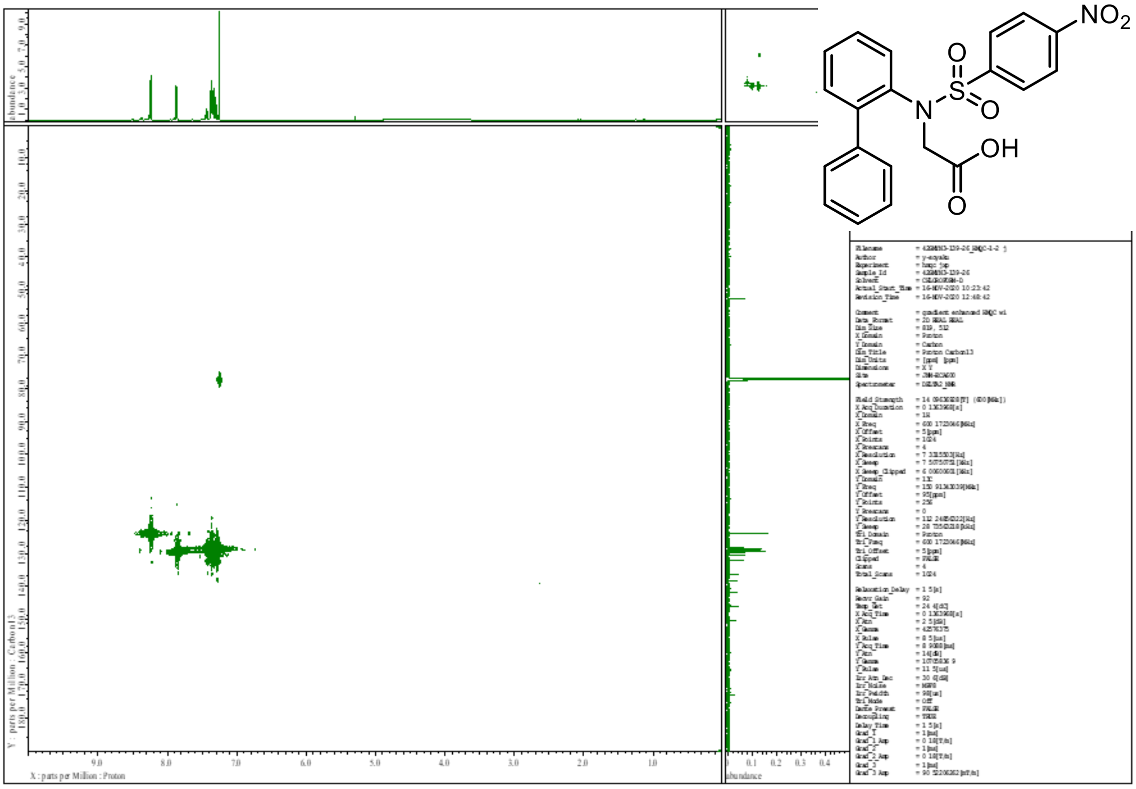

^{1}H-NMR (600 MHz, $CDCl_3$) of **1Ag**

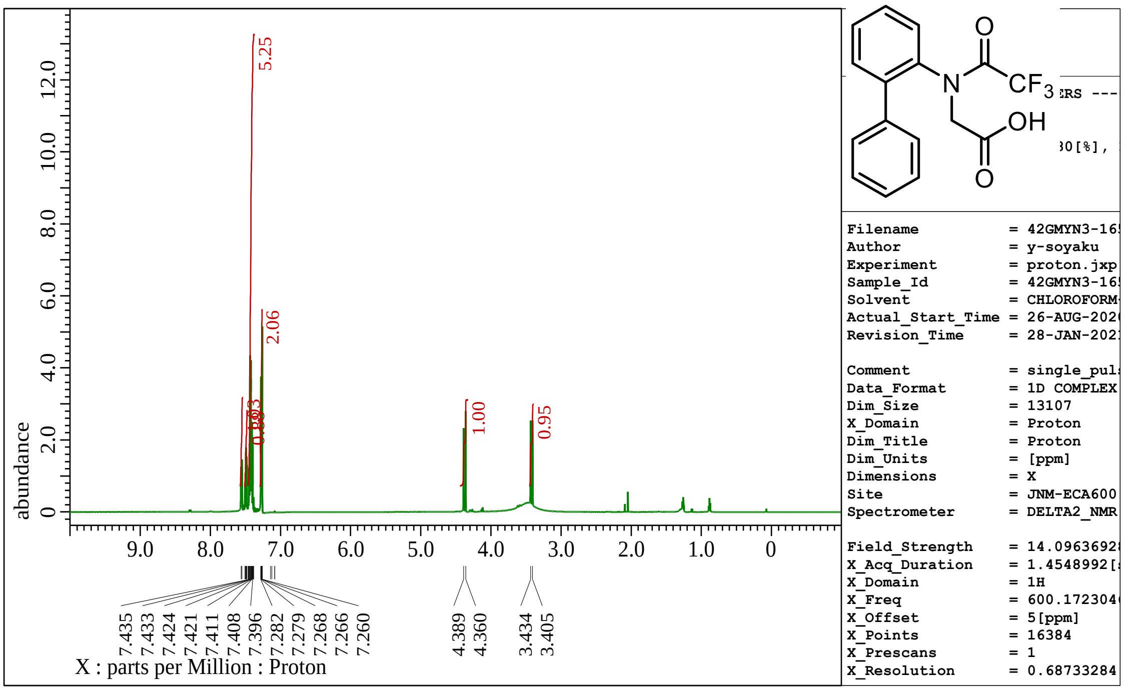

$^{13}C\{^{1}H\}$-NMR (150 MHz, $CDCl_3$) of **1Ag**

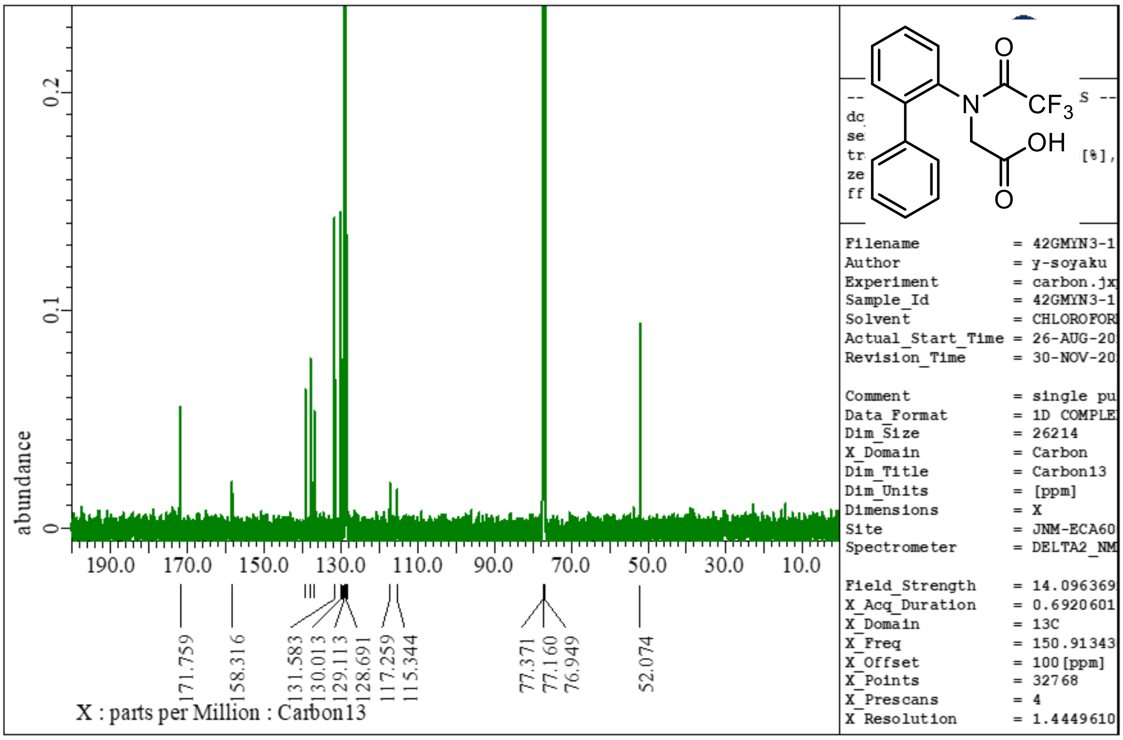

H-H COSY-NMR (600 MHz, $CDCl_3$) of **1Ag**

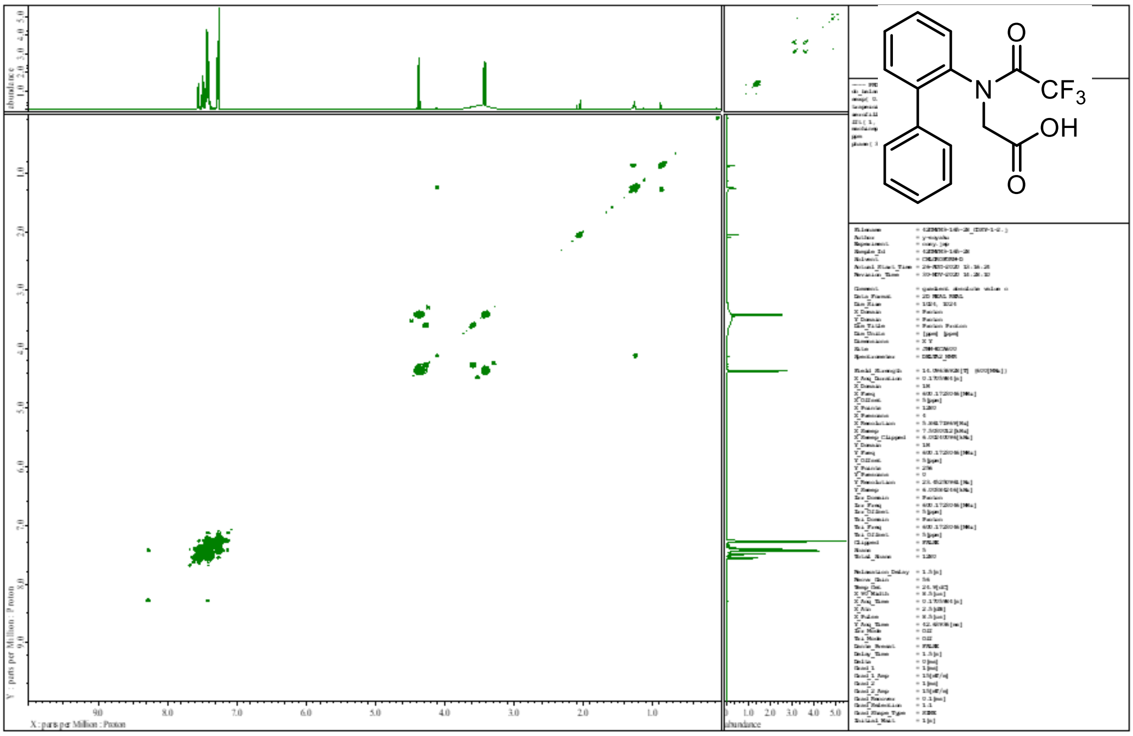

HMQC-NMR ($CDCl_3$) of **1Ag**

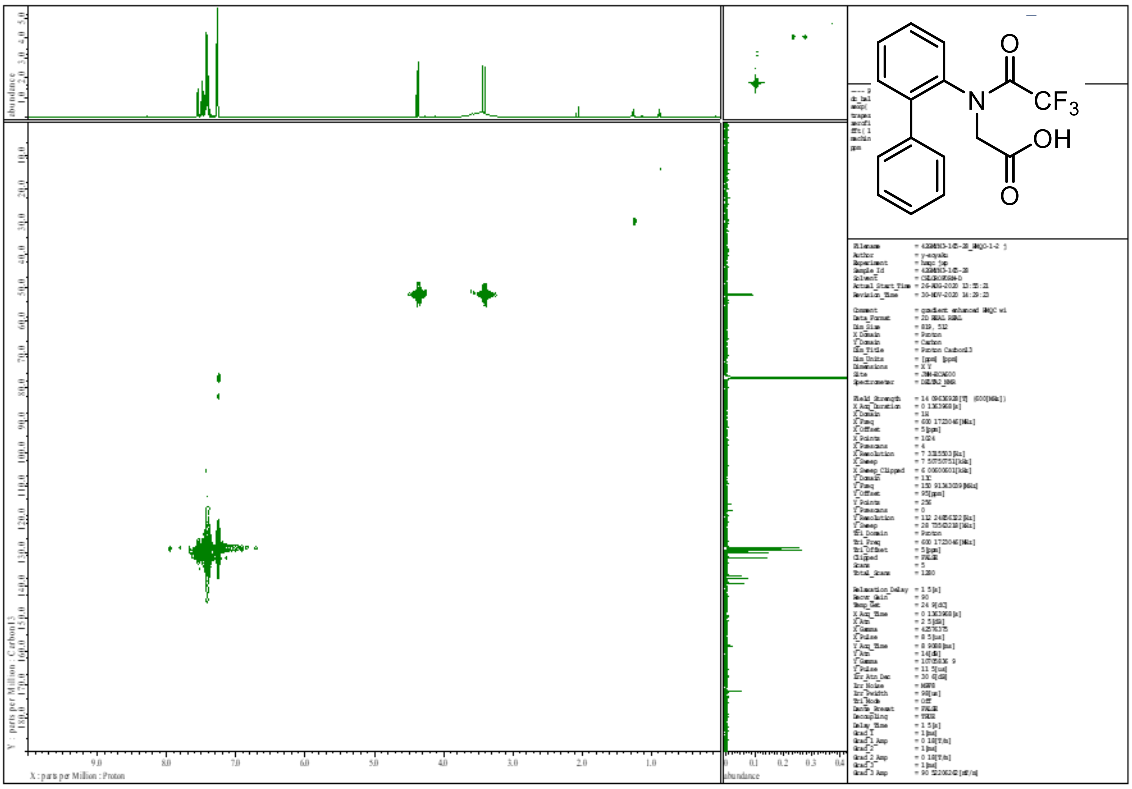

^{1}H-NMR (600 MHz, $CDCl_3$) of **1Ah**

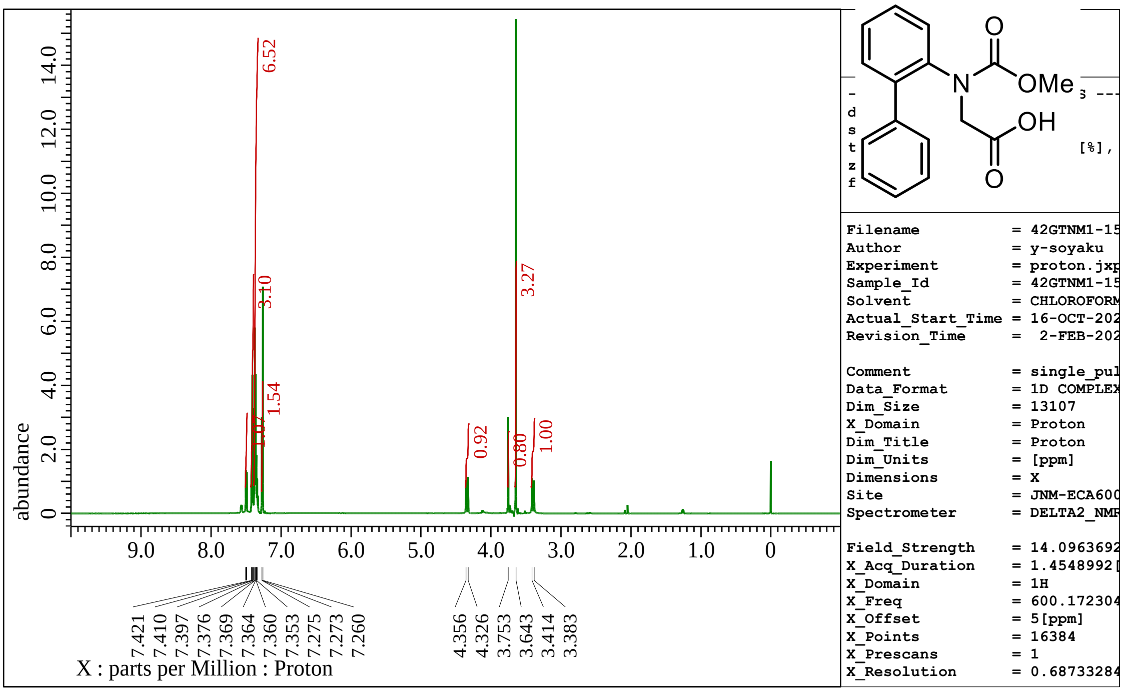

^{13}C{^{1}H}-NMR (150 MHz, $CDCl_3$) of **1Ah**

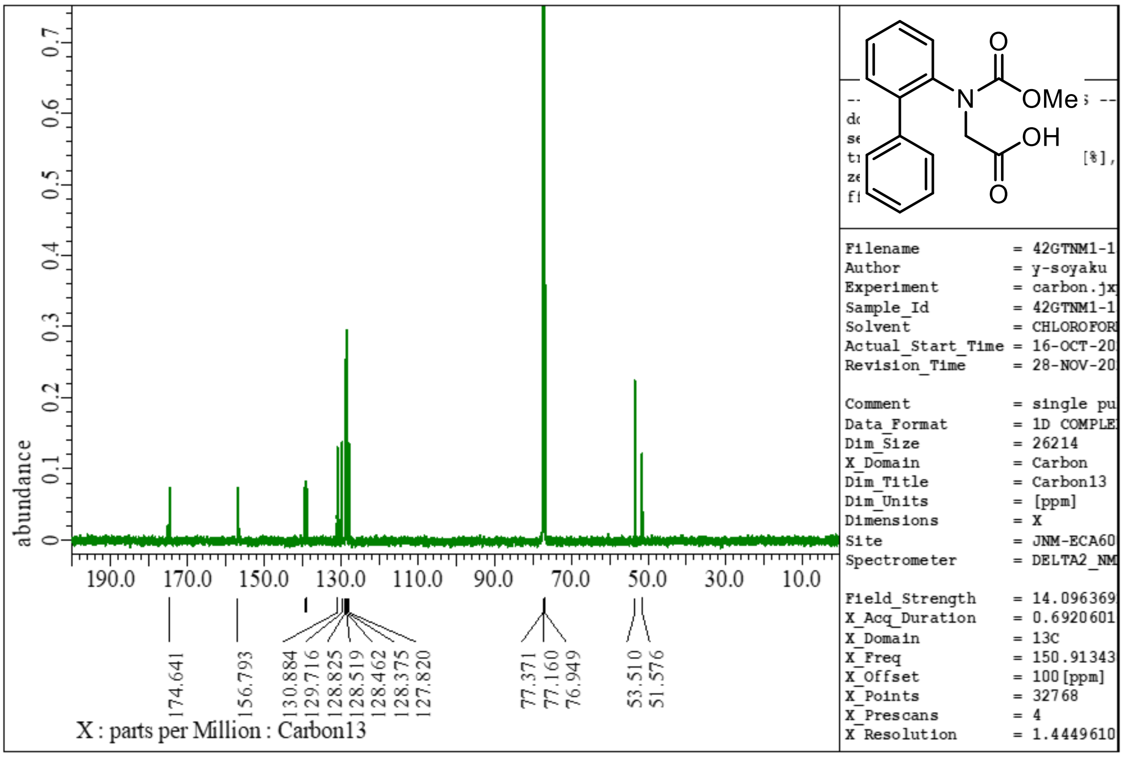

H-H COSY-NMR (600 MHz, $CDCl_3$) of **1Ah**

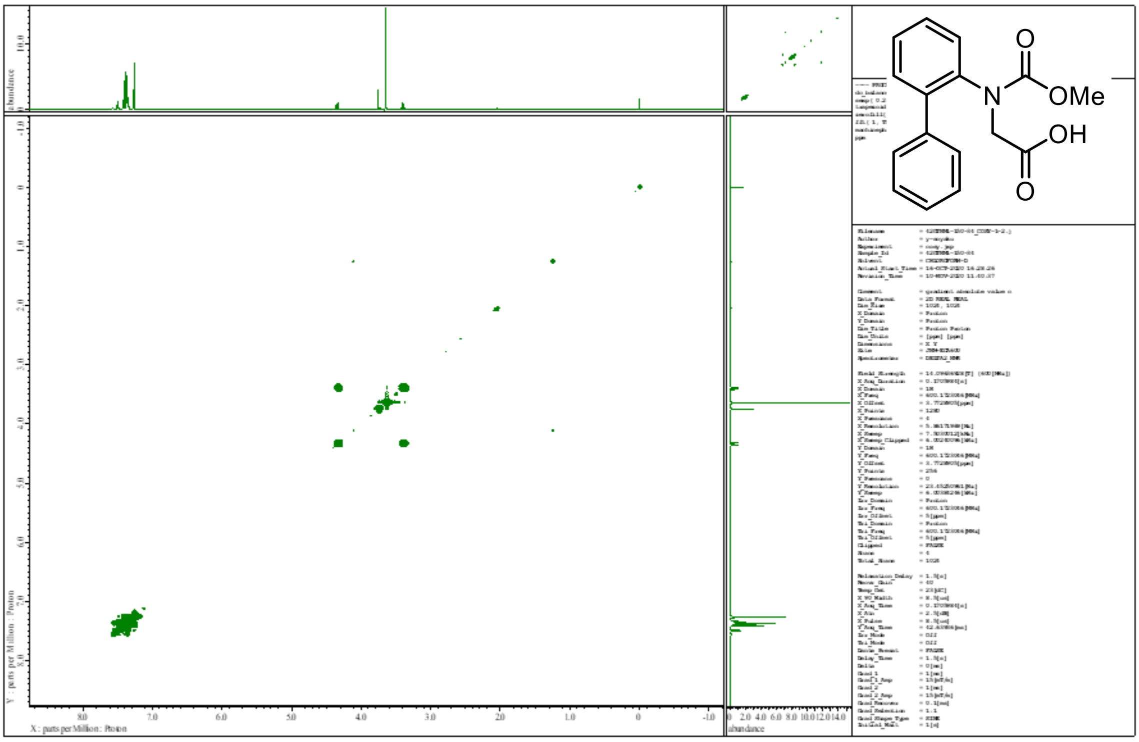

HMQC-NMR ($CDCl_3$) of **1Ah**

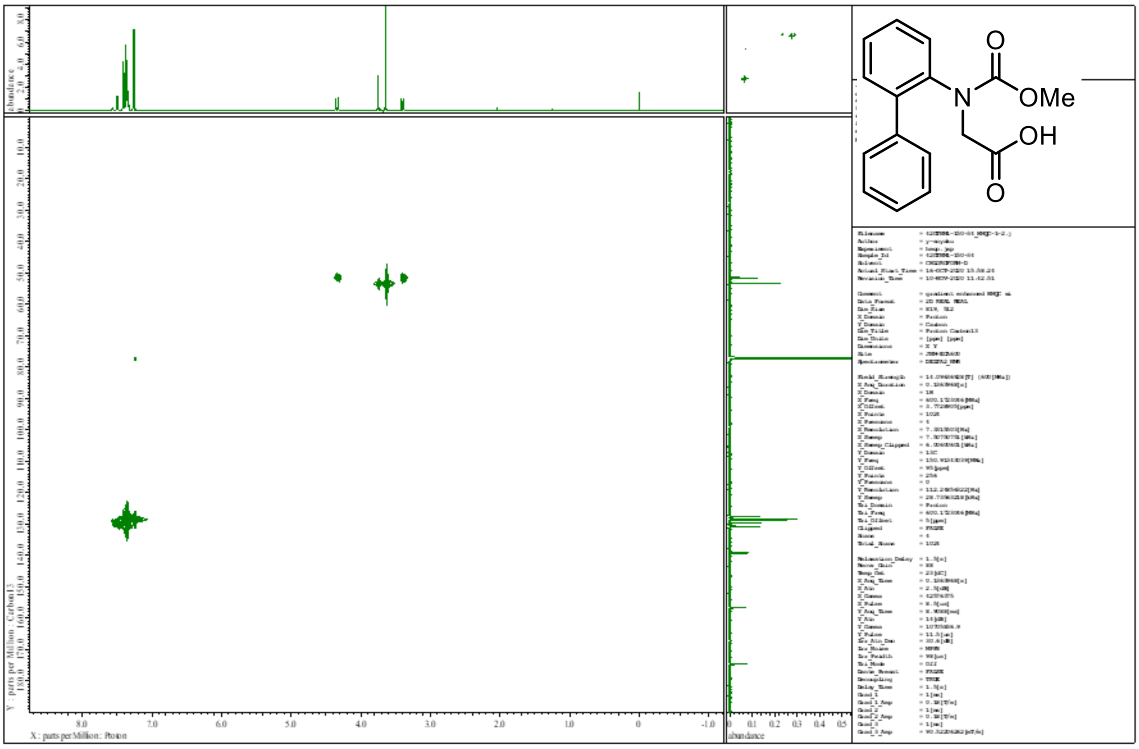

^{1}H-NMR (600 MHz, $CDCl_3$) of **1Bc**

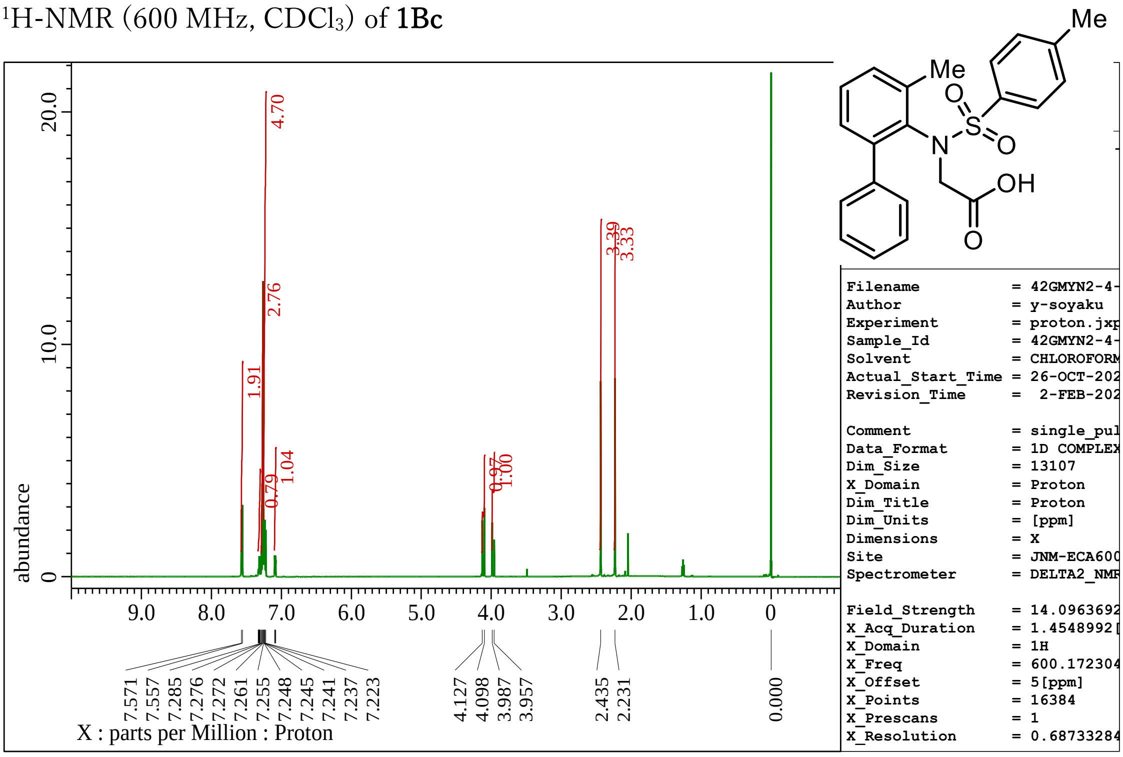

^{13}C{^{1}H }-NMR (150 MHz, $CDCl_3$) of **1Bc**

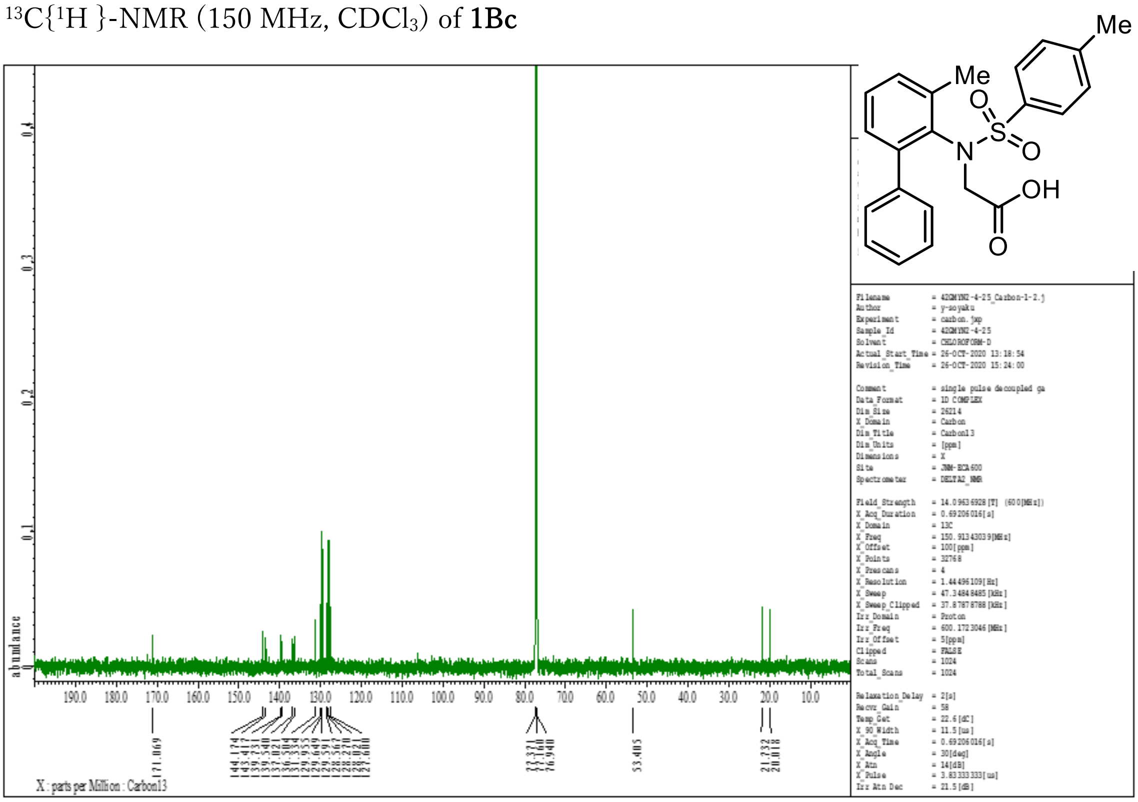

H-H COSY-NMR (600 MHz, $CDCl_3$) of **1Bc**

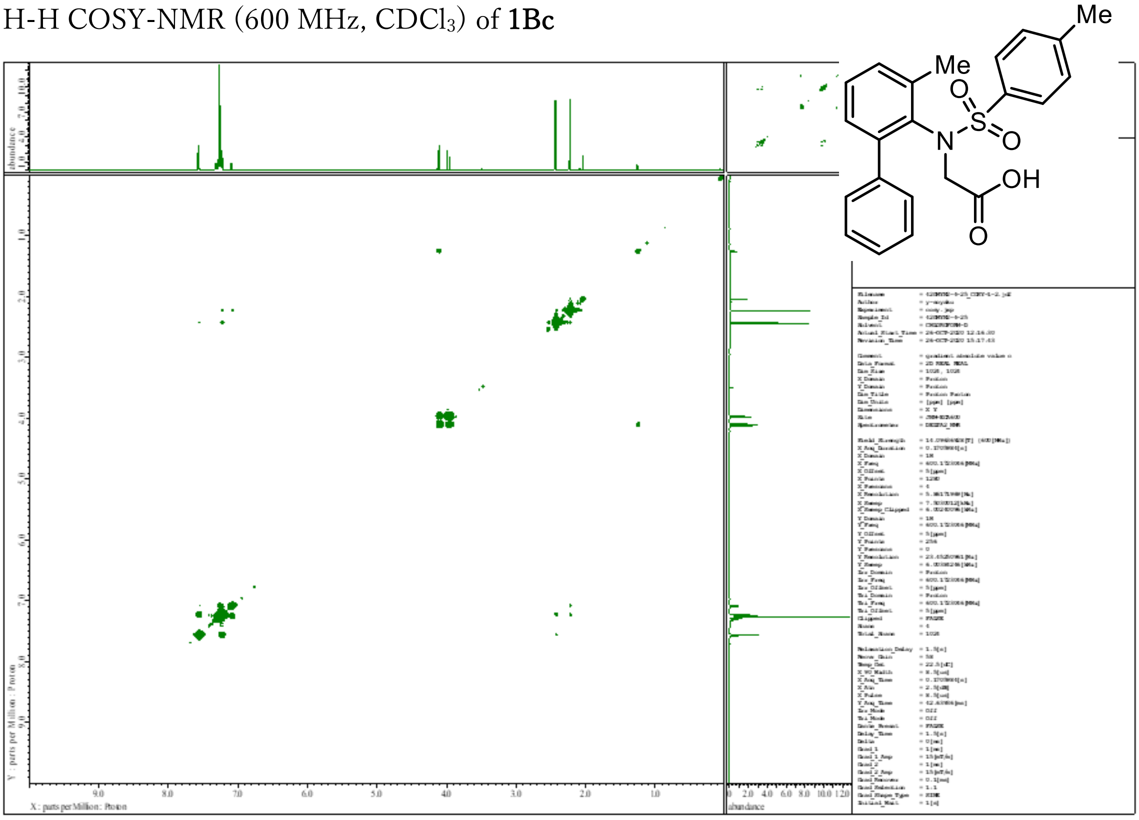

HMQC-NMR ($CDCl_3$) of **1Bc**

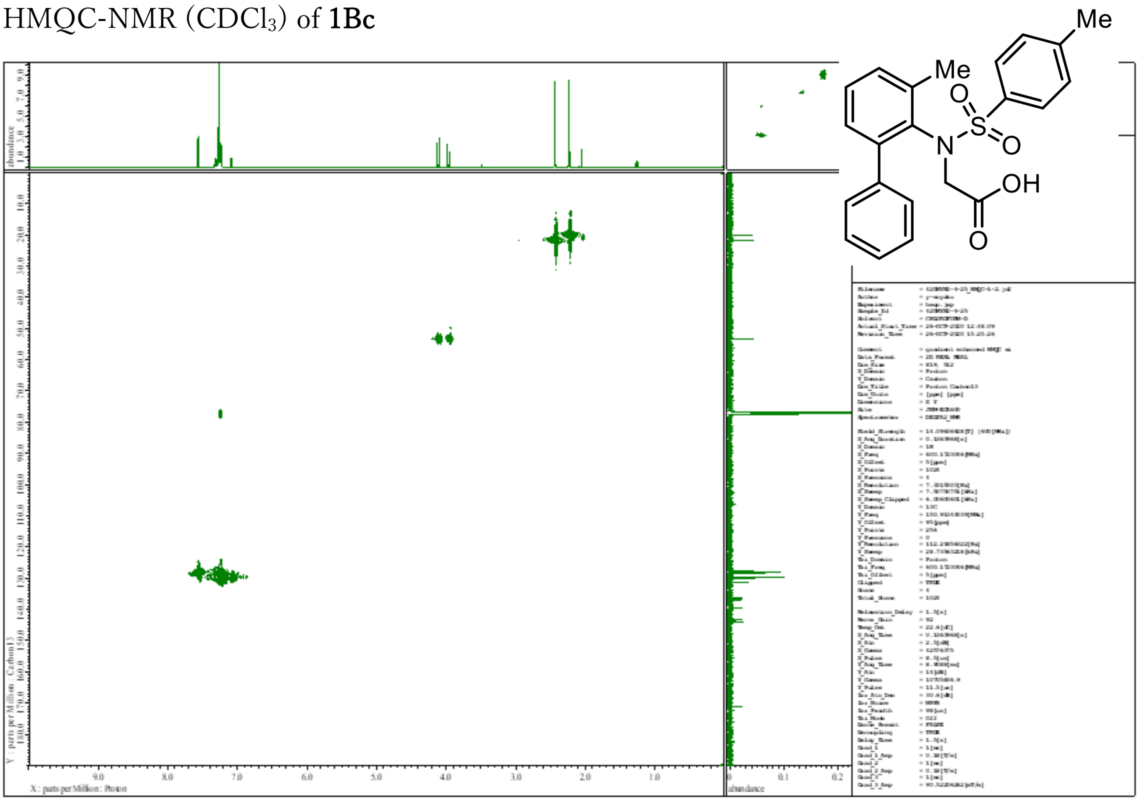

^{1}H-NMR (600 MHz, $CDCl_3$) of **1Bd**

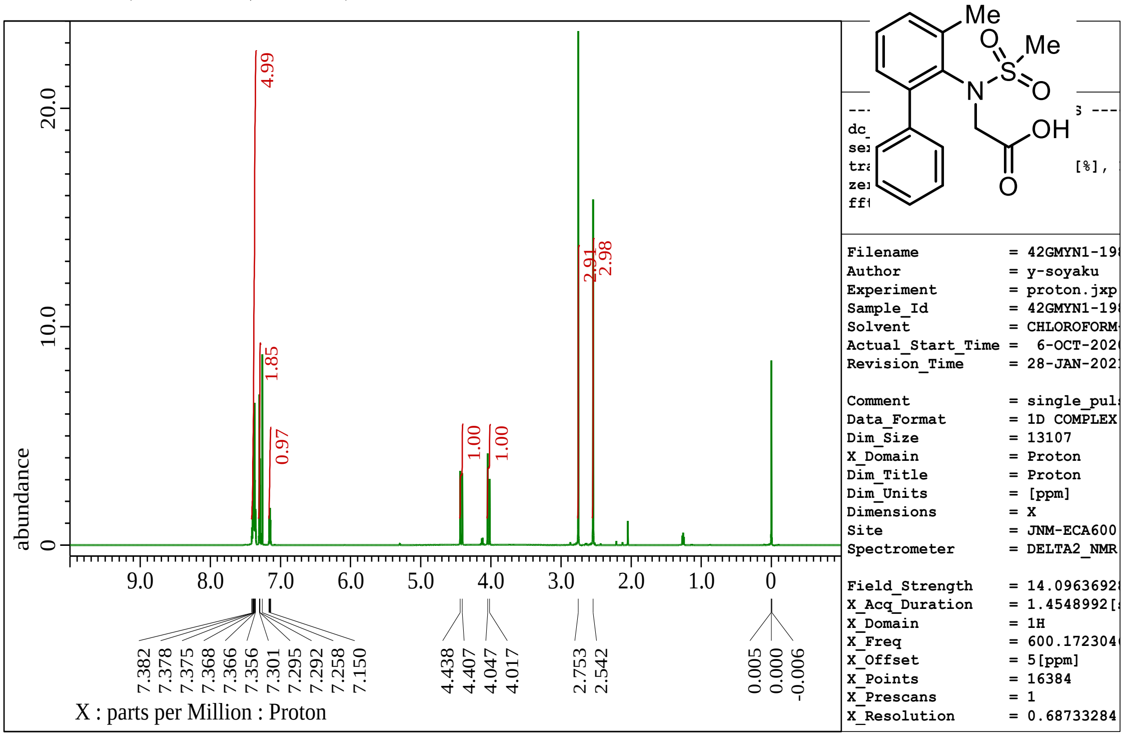

^{13}C{^{1}H}-NMR (150 MHz, $CDCl_3$) of **1Bd**

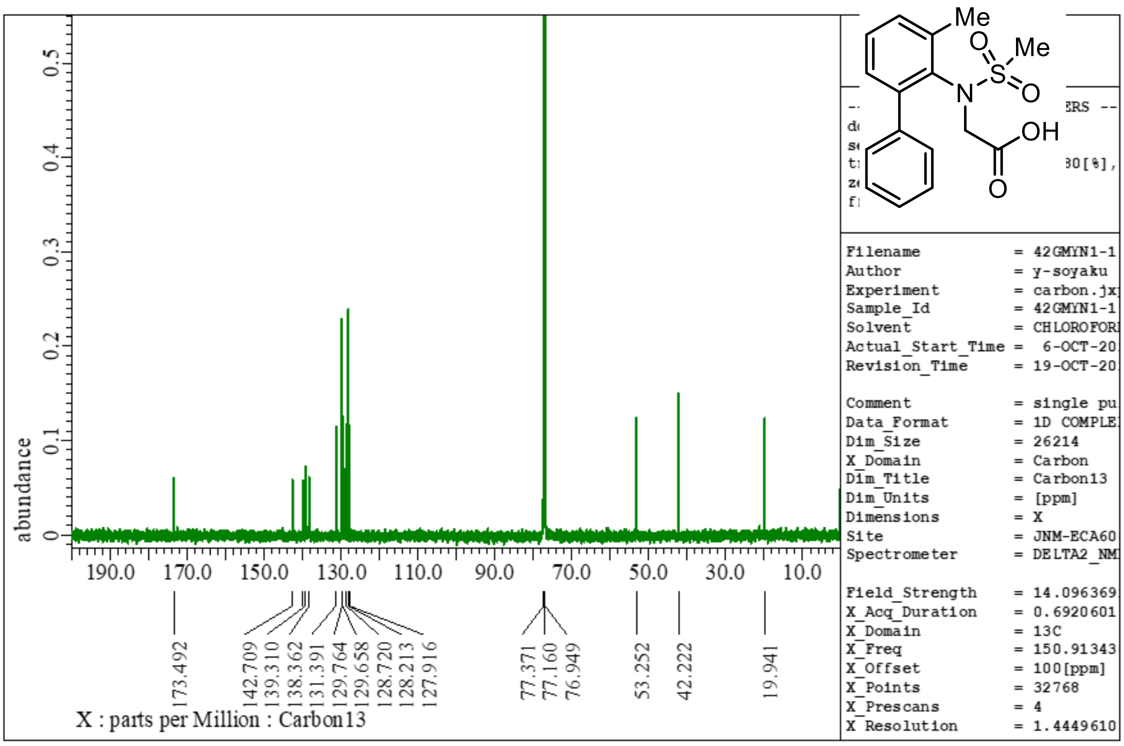

H-H COSY-NMR (600 MHz, $CDCl_3$) of **1Bd**

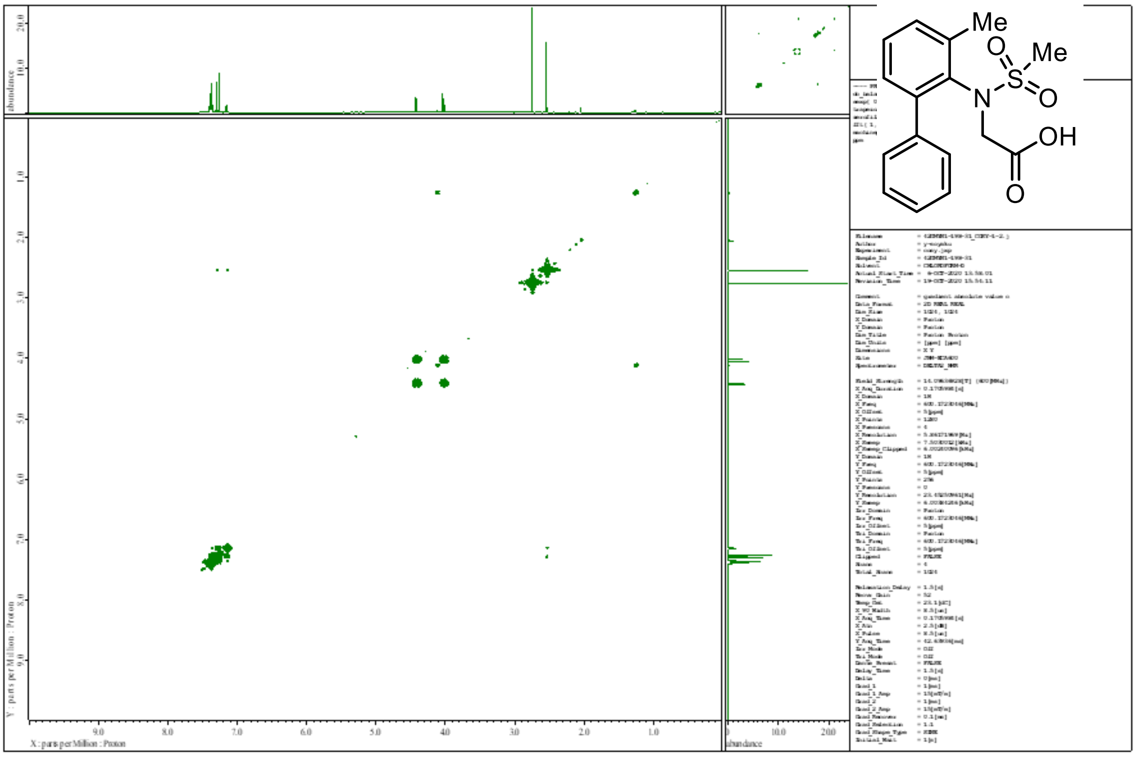

HMQC-NMR ($CDCl_3$) of **1Bd**

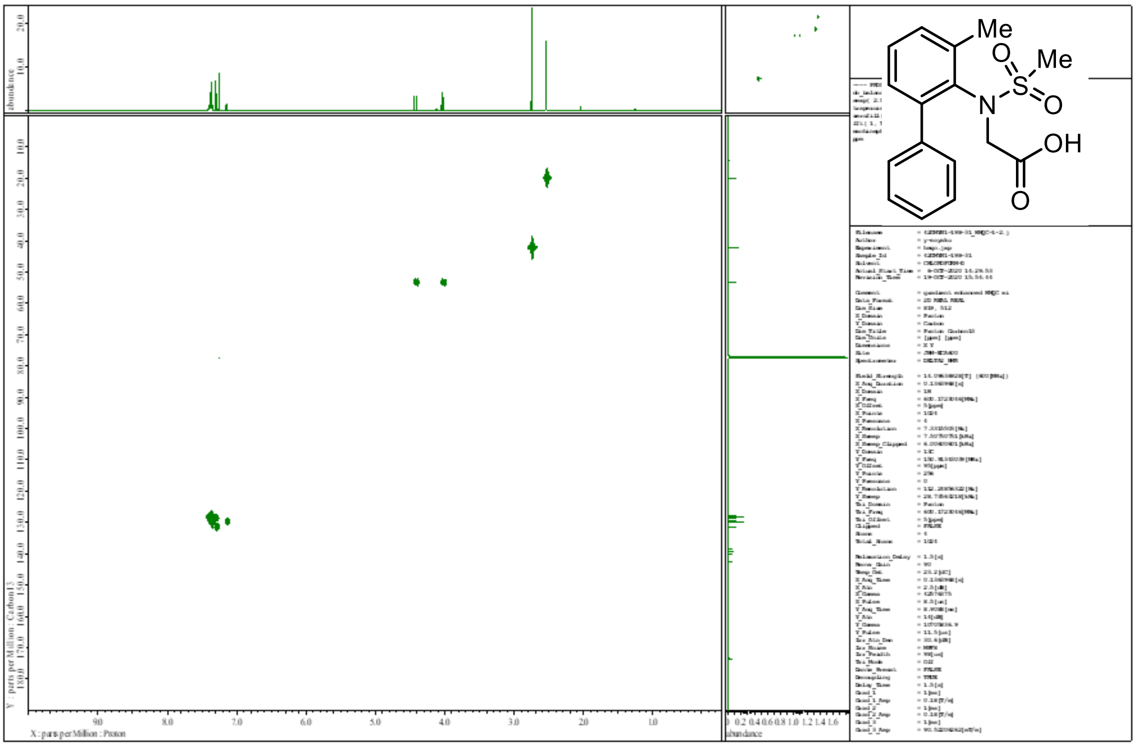

^{1}H-NMR (600 MHz, $CDCl_3$) of **1Be**

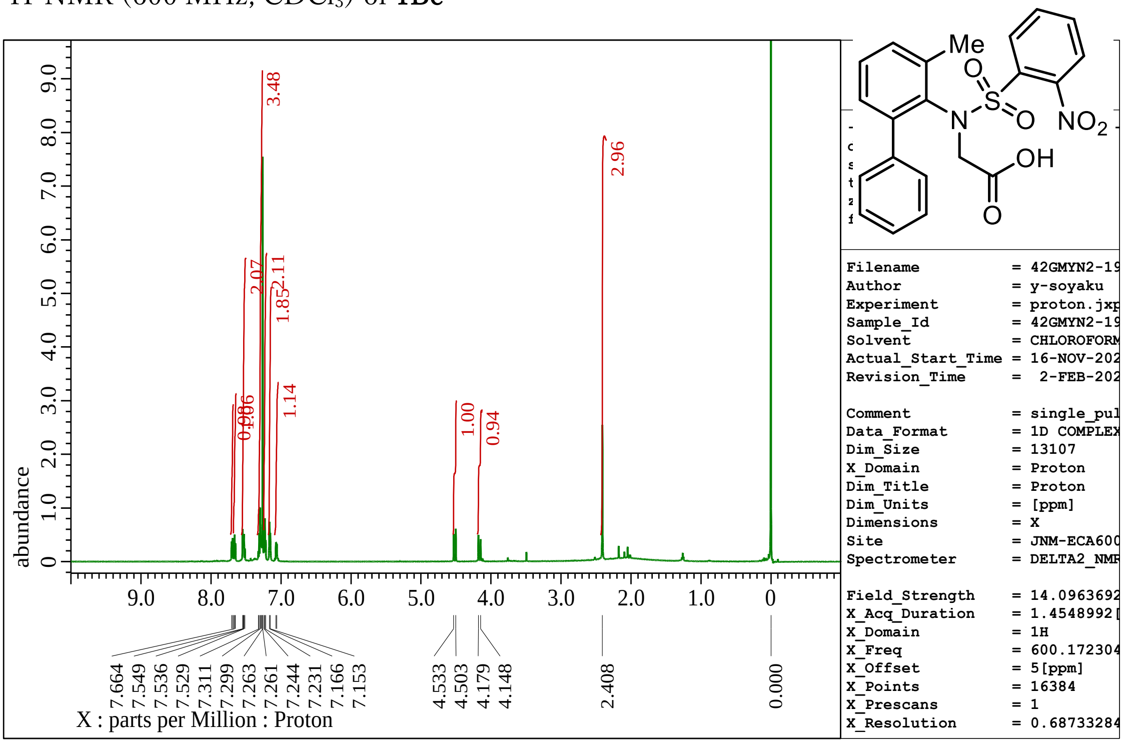

$^{13}C\{^{1}H\}$-NMR (150 MHz, $CDCl_3$) of **1Be**

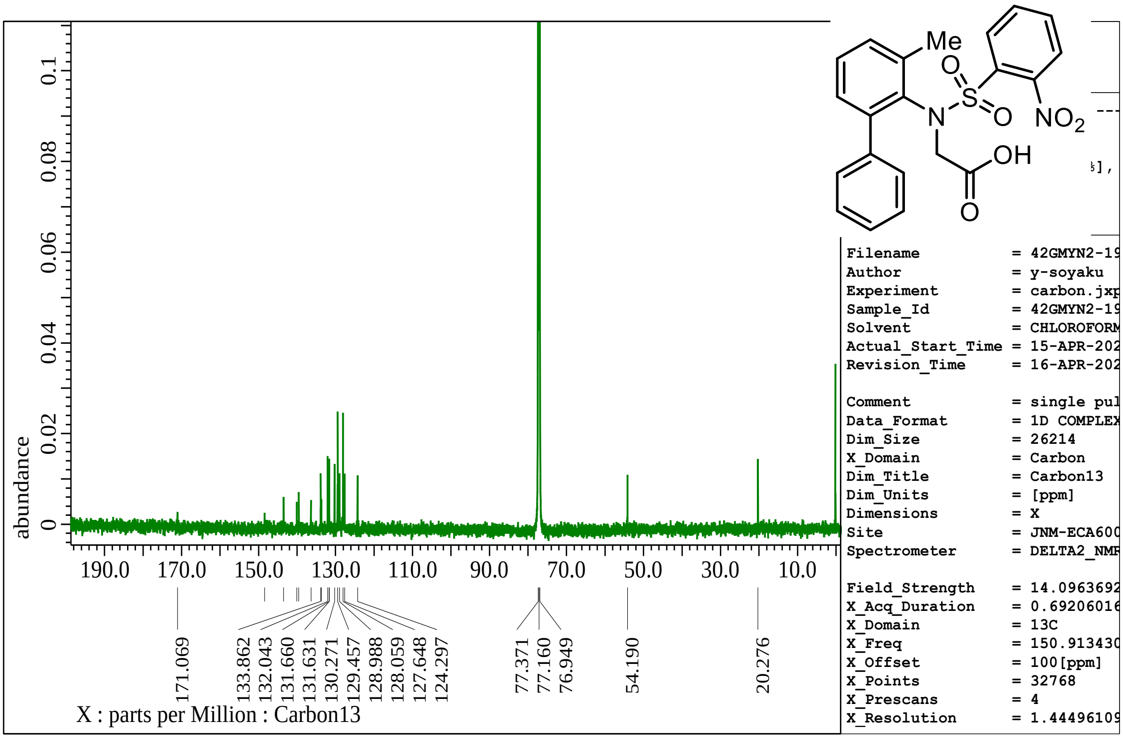

H-H COSY-NMR (600 MHz, $CDCl_3$) of **1Be**

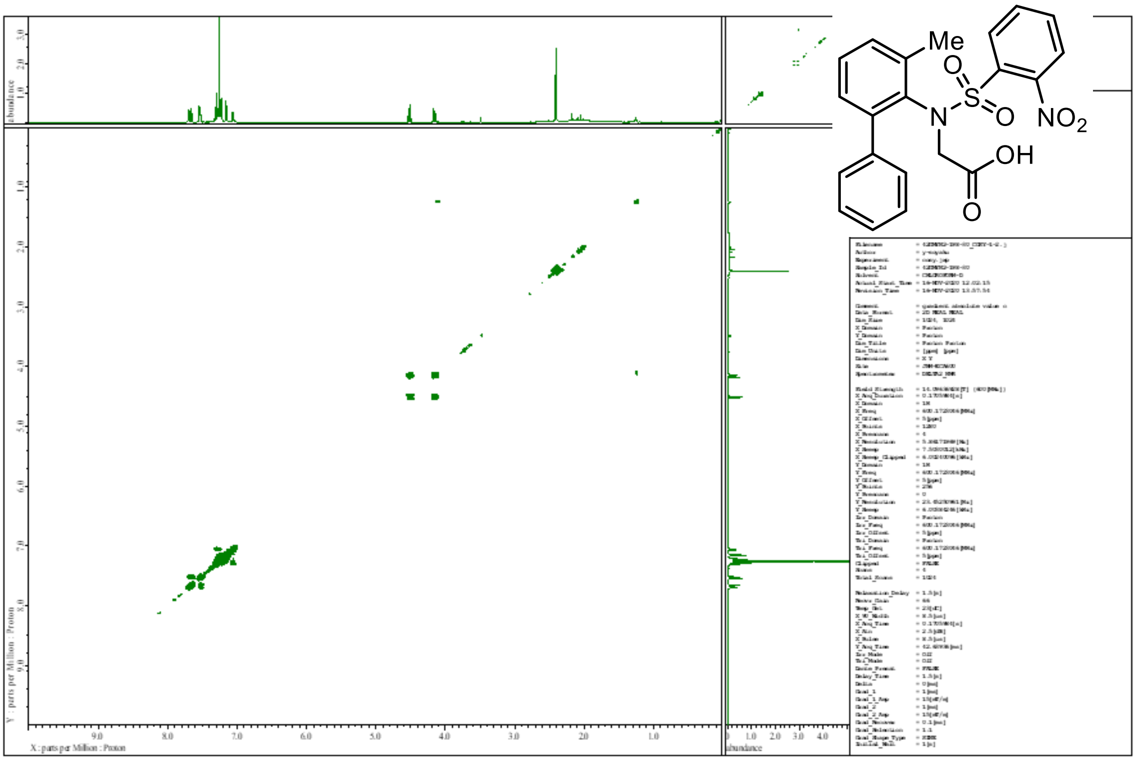

HMQC-NMR ($CDCl_3$) of **1Be**

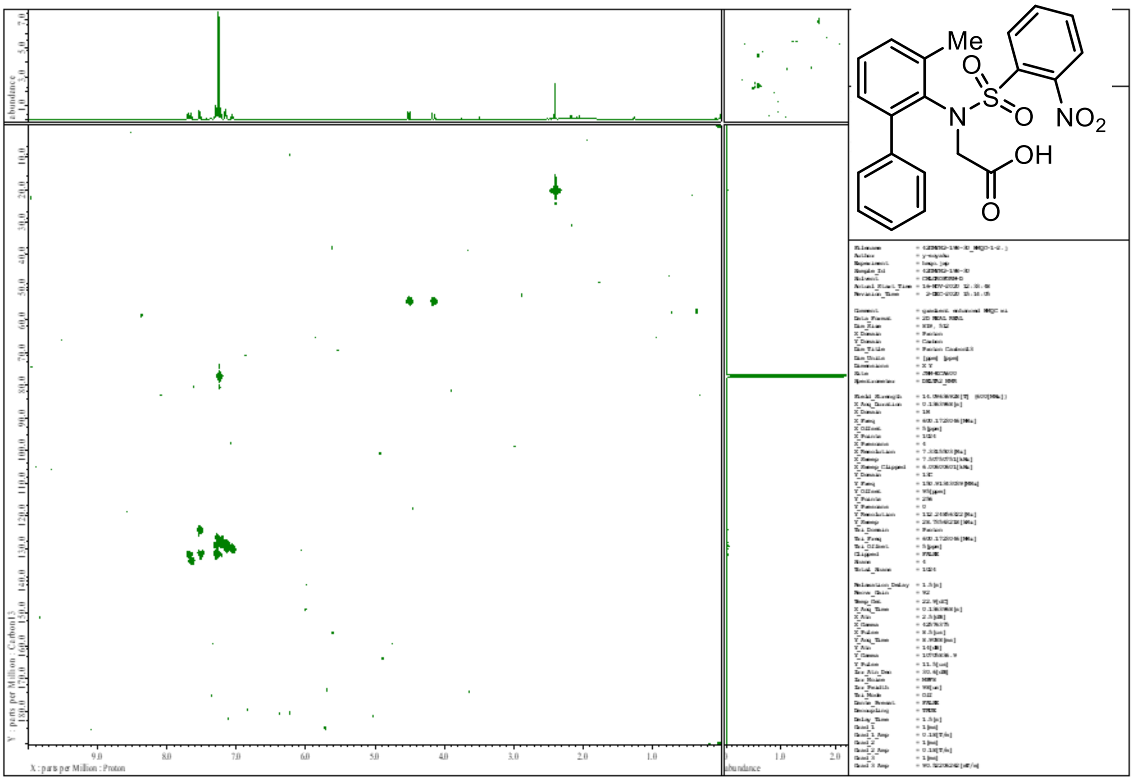

^{1}H-NMR (600 MHz, $CDCl_3$) of **1Bf**

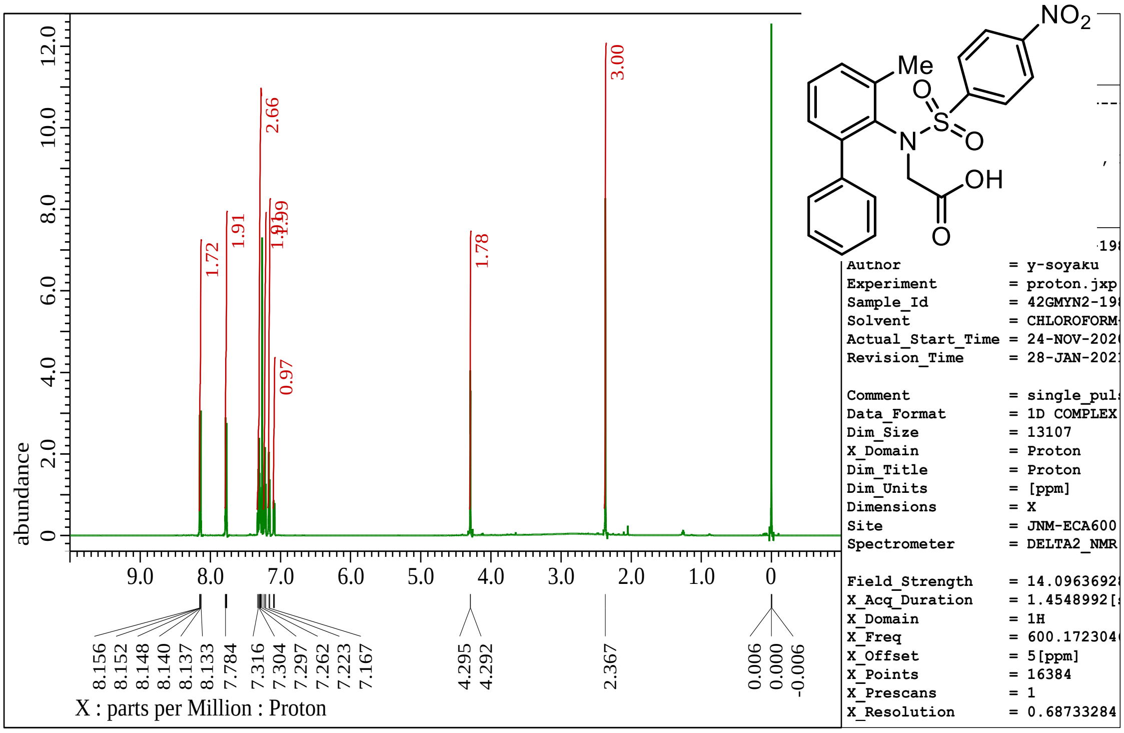

^{13}C{^{1}H }-NMR (150 MHz, $CDCl_3$) of **1Bf**

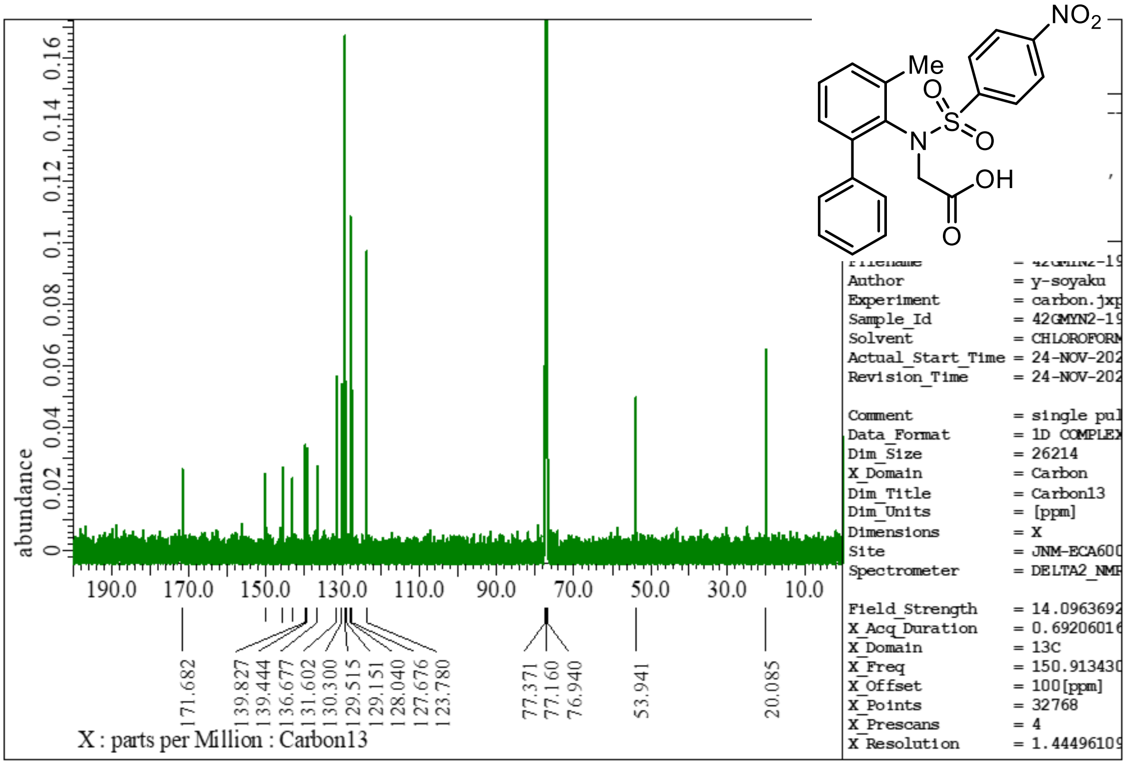

H-H COSY-NMR (600 MHz, $CDCl_3$) of **1Bf**

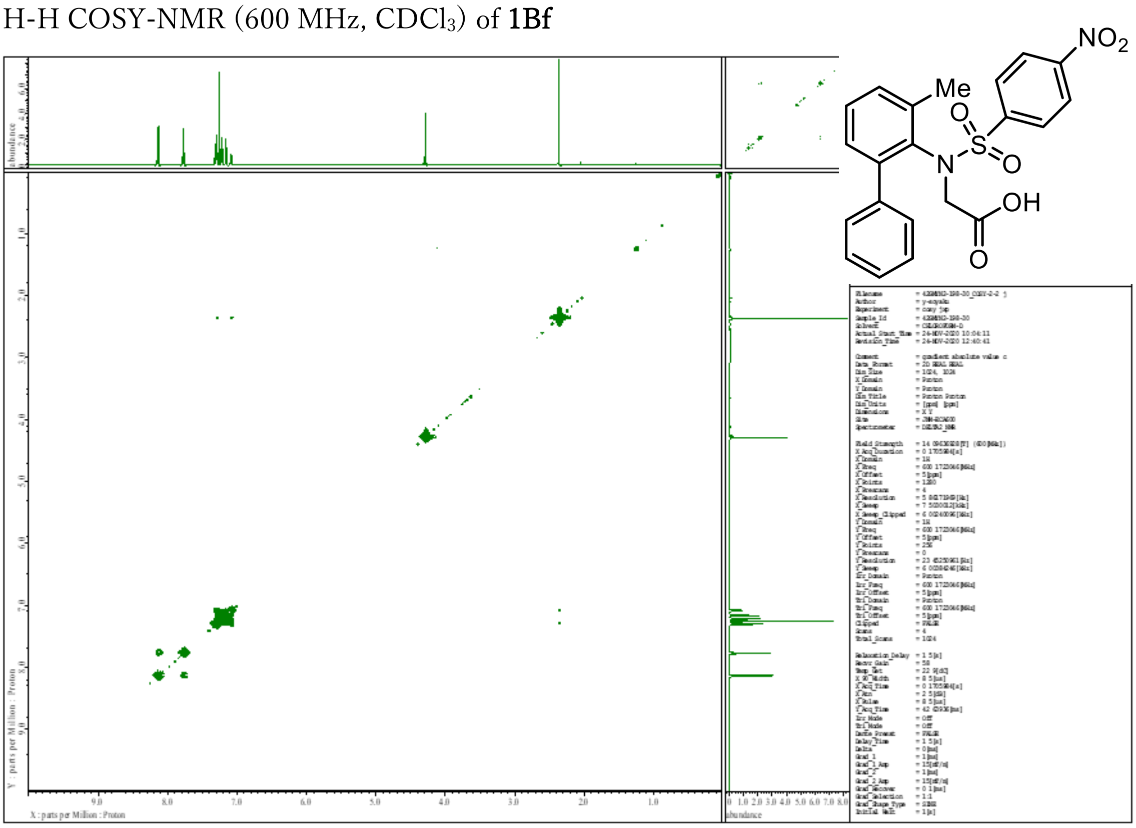

HMQC-NMR ($CDCl_3$) of **1Bf**

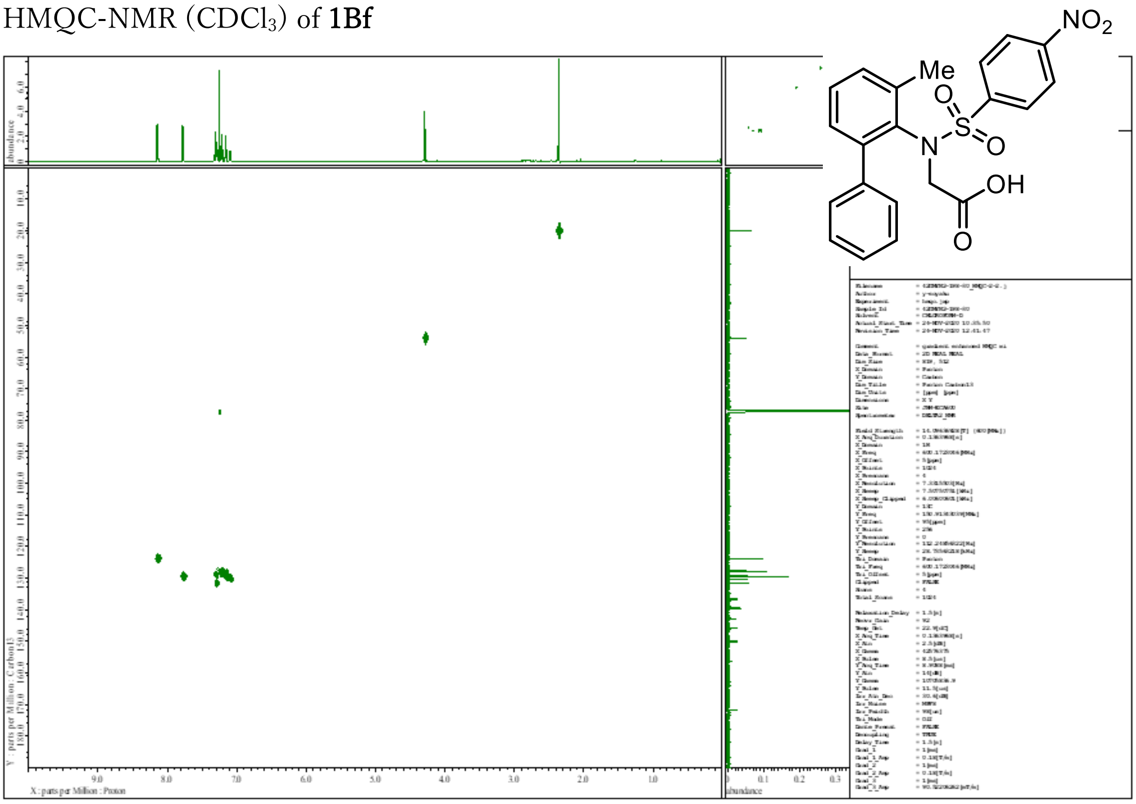

^{1}H-NMR (600 MHz, $CDCl_3$) of **1Bg**

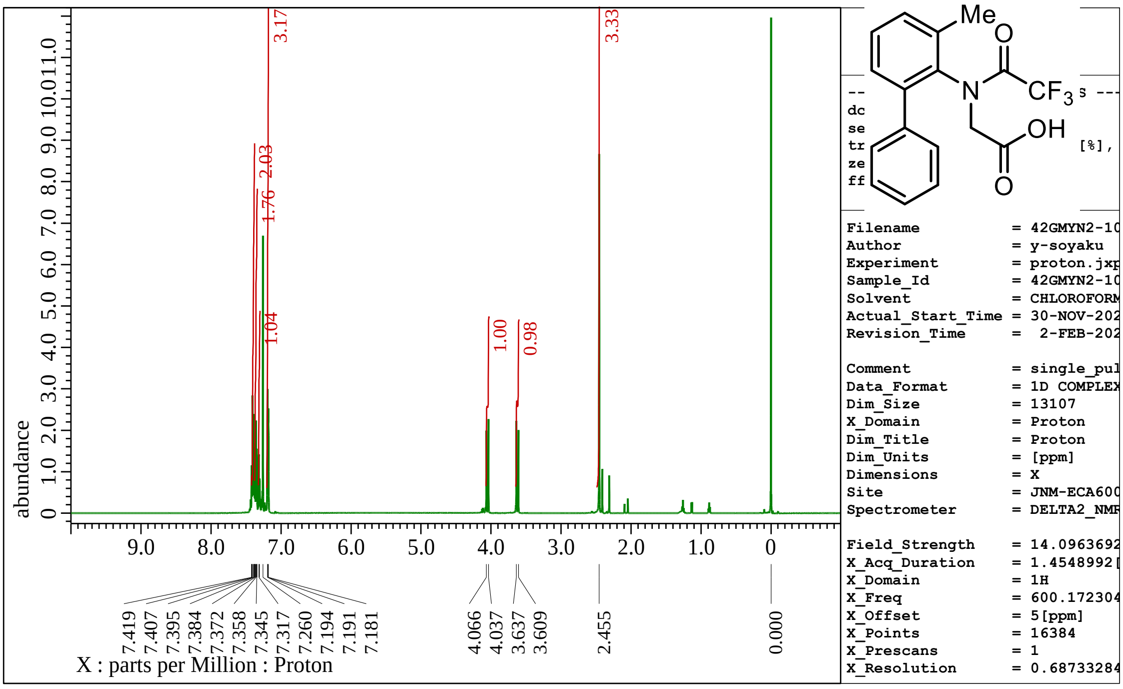

^{13}C{^{1}H }-NMR (150 MHz, $CDCl_3$) of **1Bg**

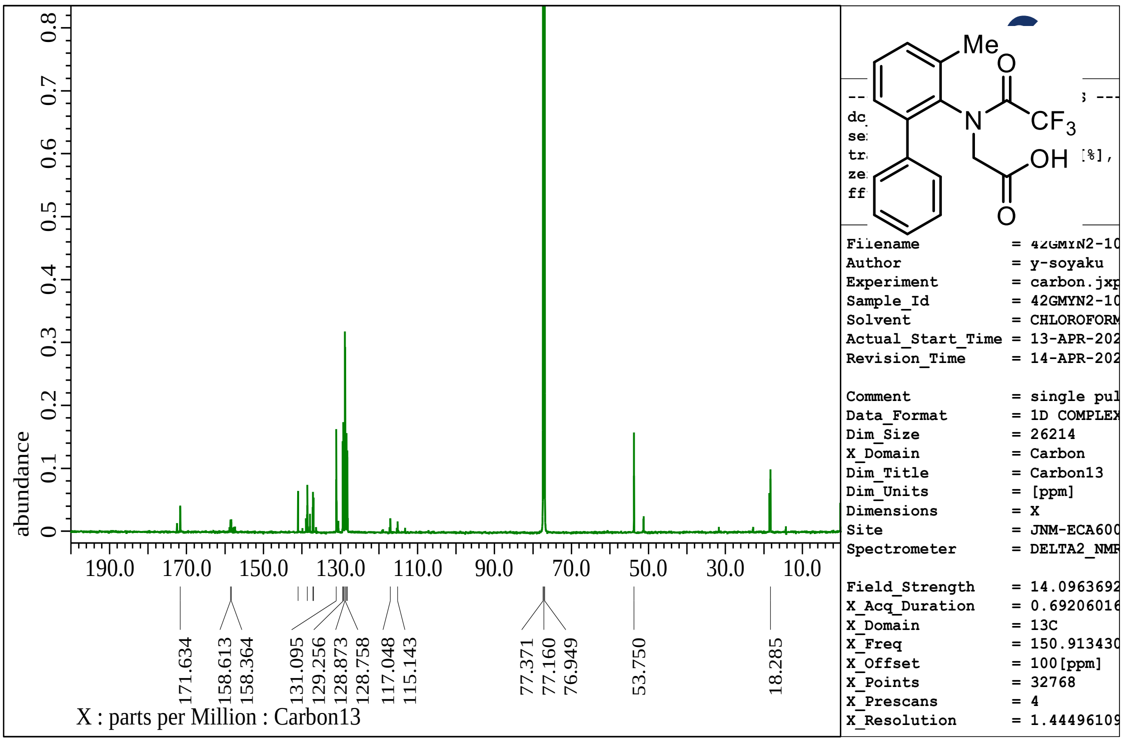

H-H COSY-NMR (600 MHz, $CDCl_3$) of **1Bg**

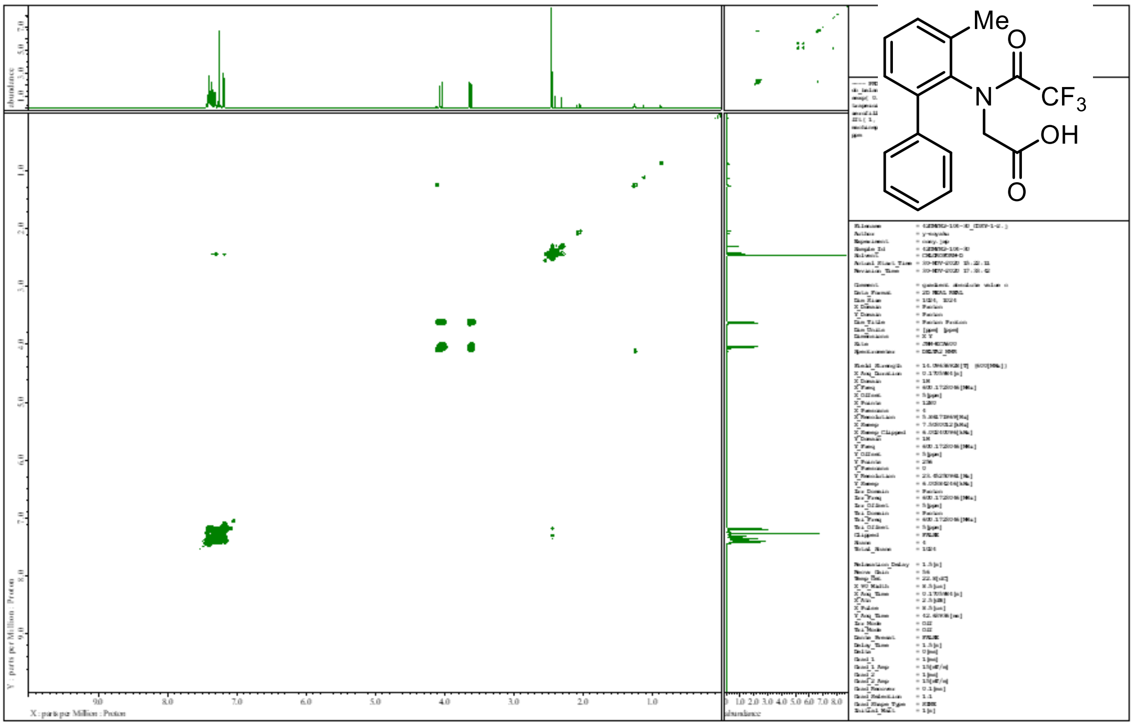

HMQC-NMR ($CDCl_3$) of **1Bg**

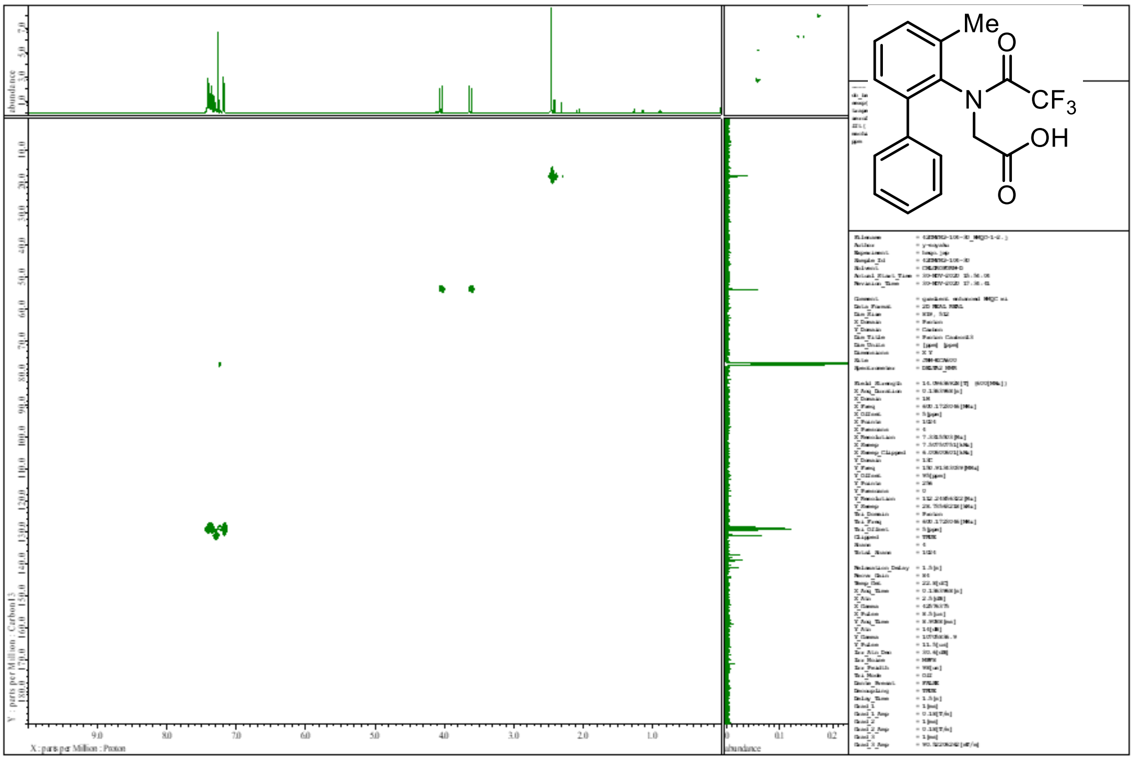

^{1}H-NMR (600 MHz, $CDCl_3$) of **1Bh**

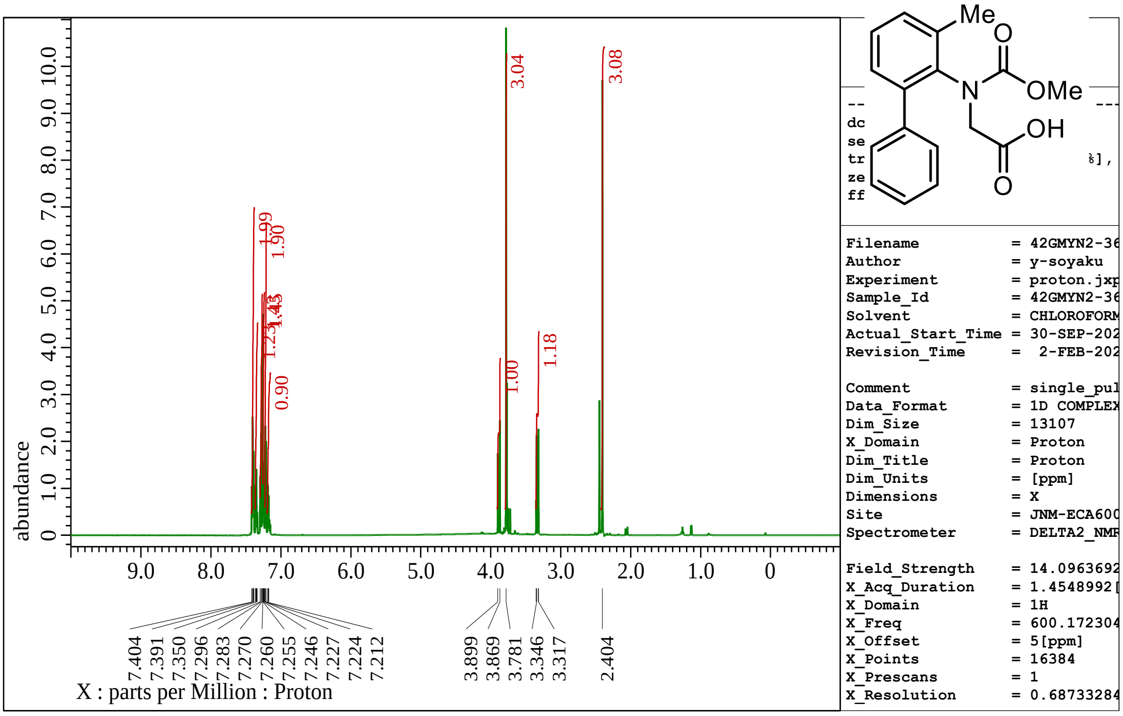

^{13}C{^{1}H }-NMR (150 MHz, $CDCl_3$) of **1Bh**

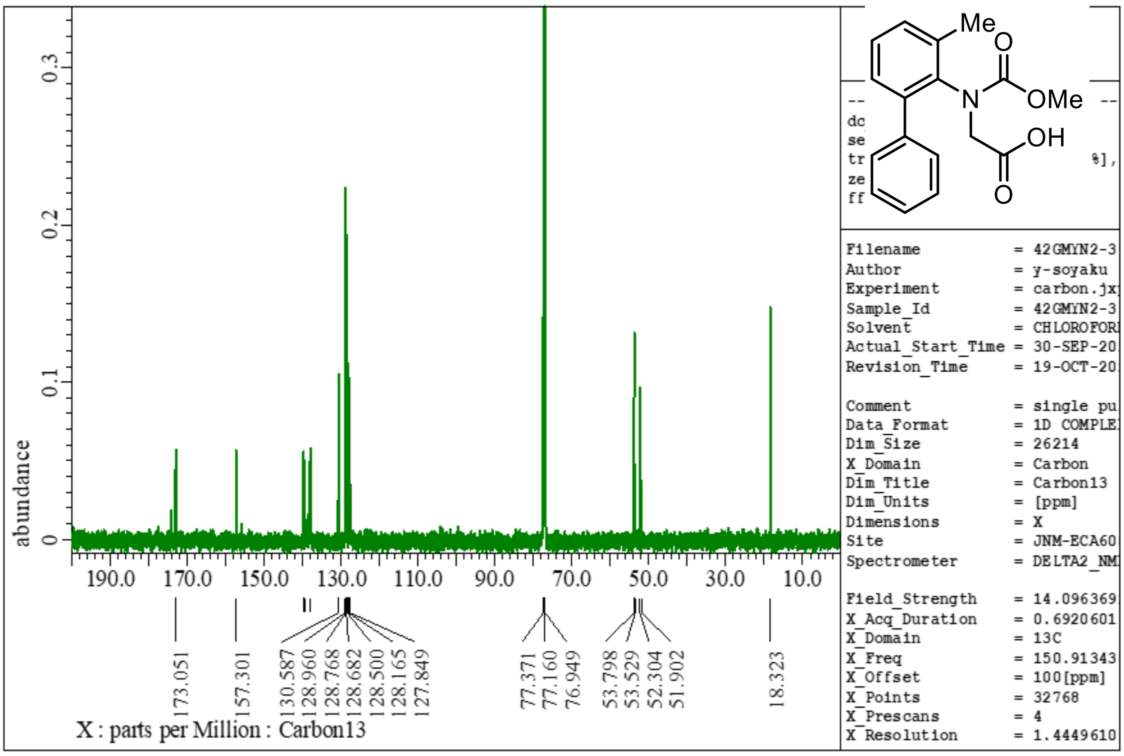

H-H COSY-NMR (600 MHz, $CDCl_3$) of **1Bh**

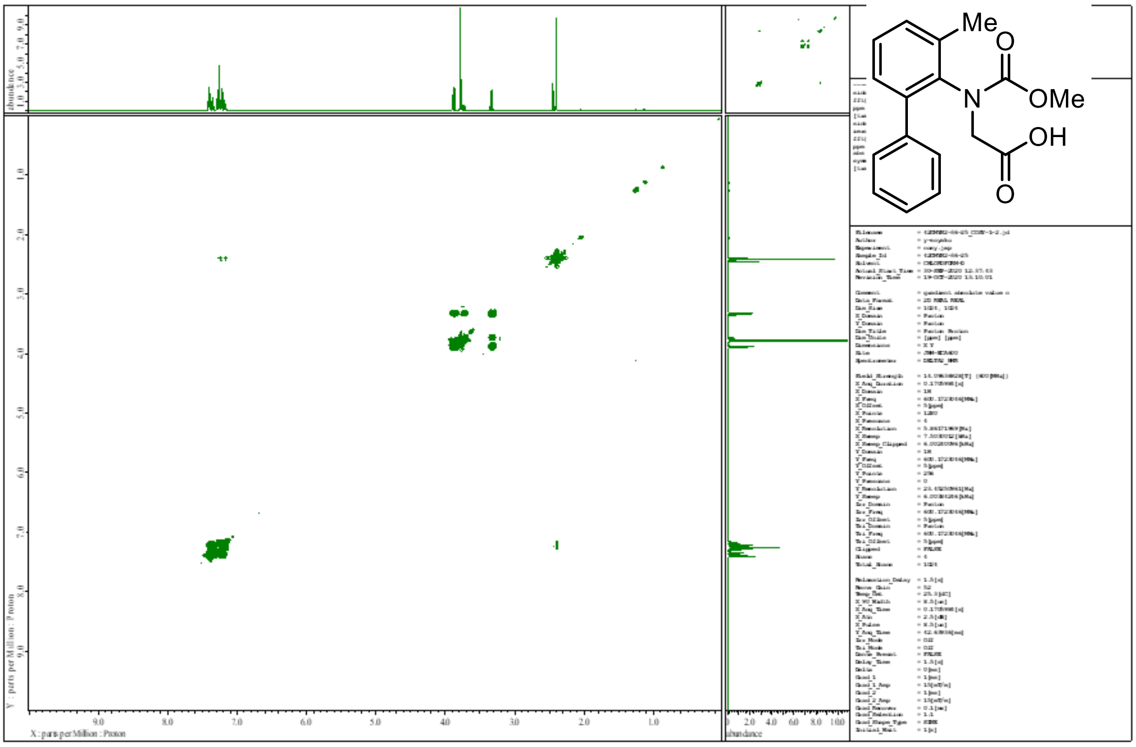

HMQC-NMR ($CDCl_3$) of **1Bh**

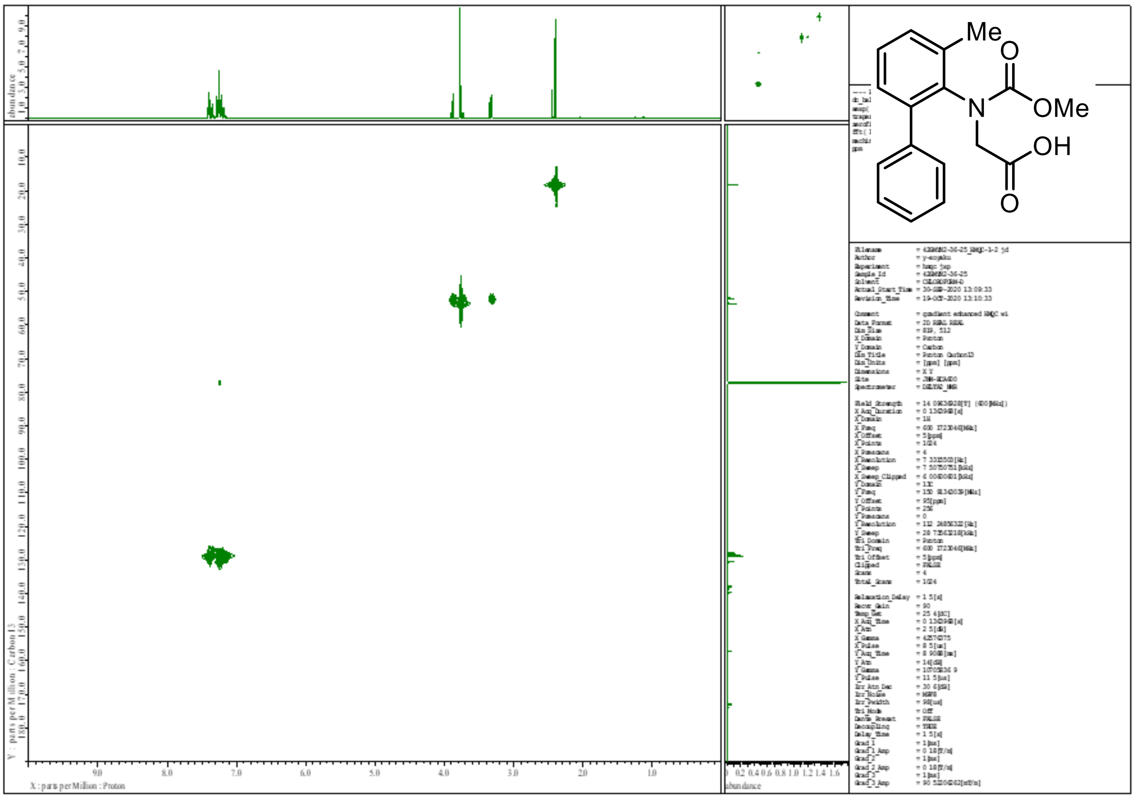

^{1}H-NMR (600 MHz, $CDCl_3$) of **2**

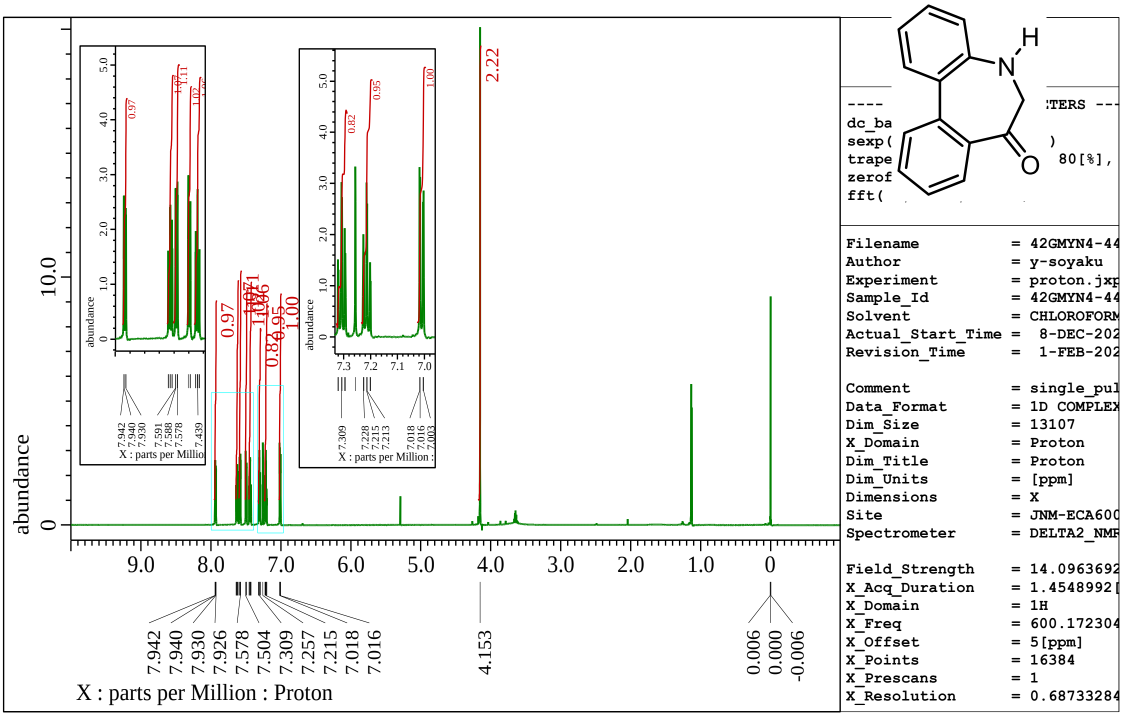

^{13}C{^{1}H }-NMR (150 MHz, $CDCl_3$) of **2**

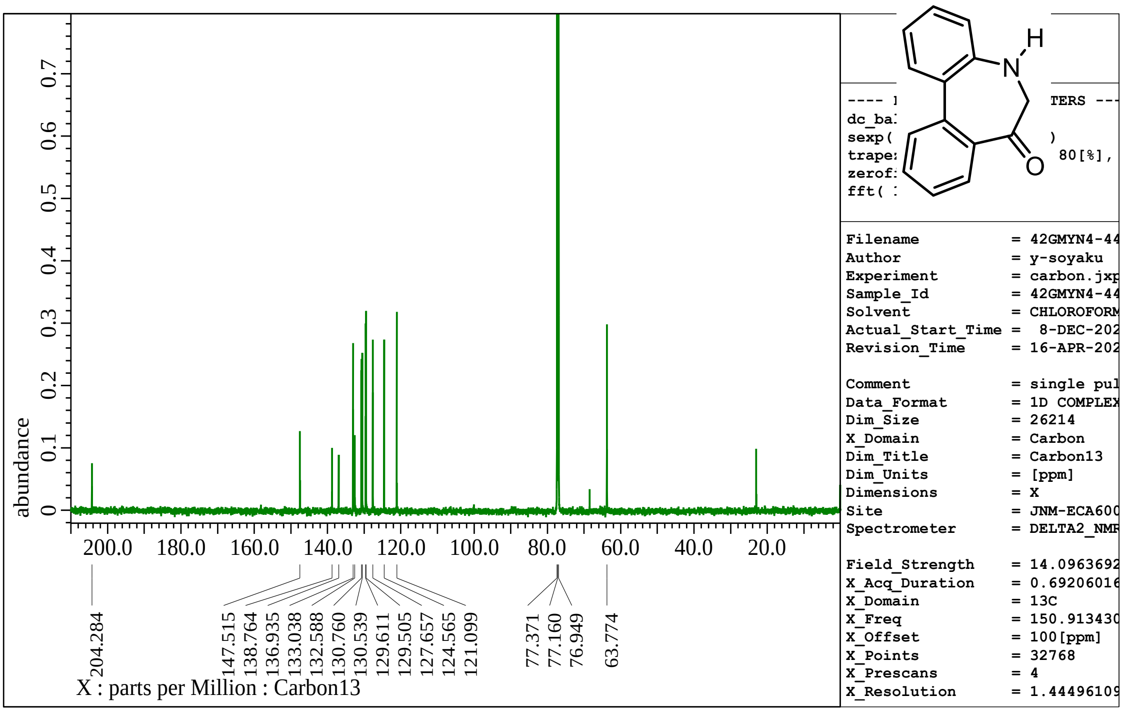

H-H COSY-NMR (600 MHz, $CDCl_3$) of **2**

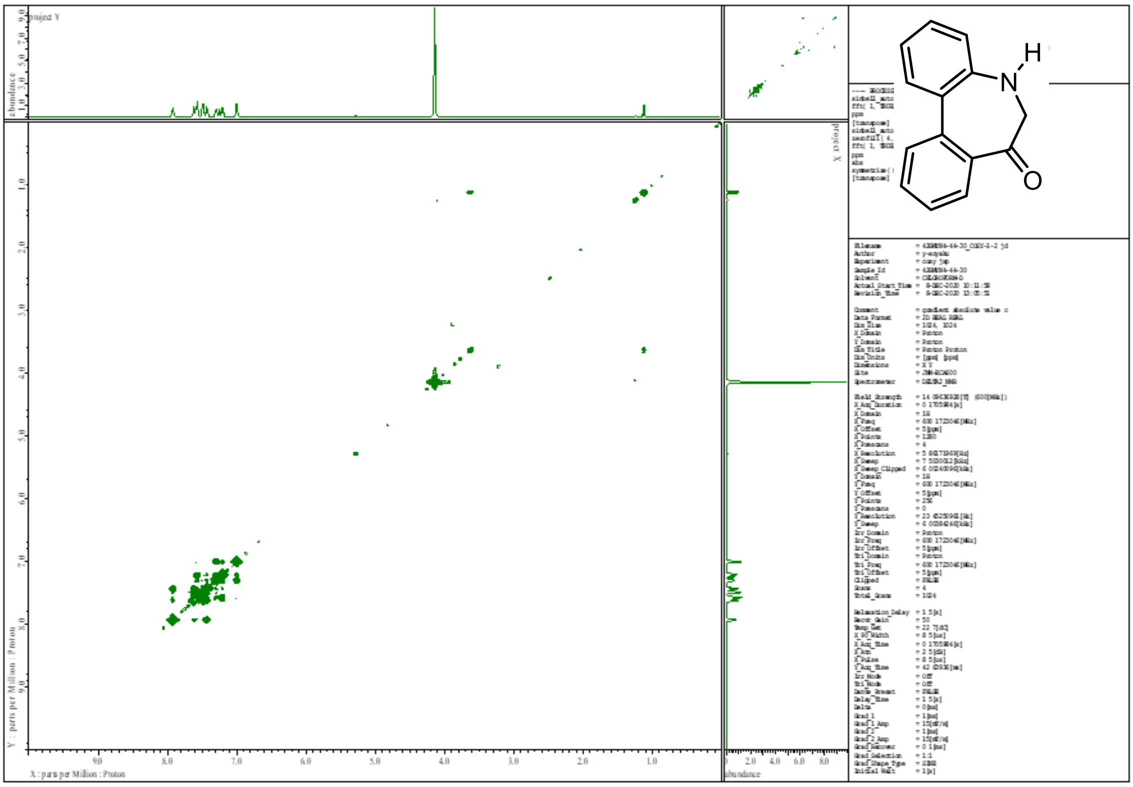

HMQC-NMR ($CDCl_3$) of **2**

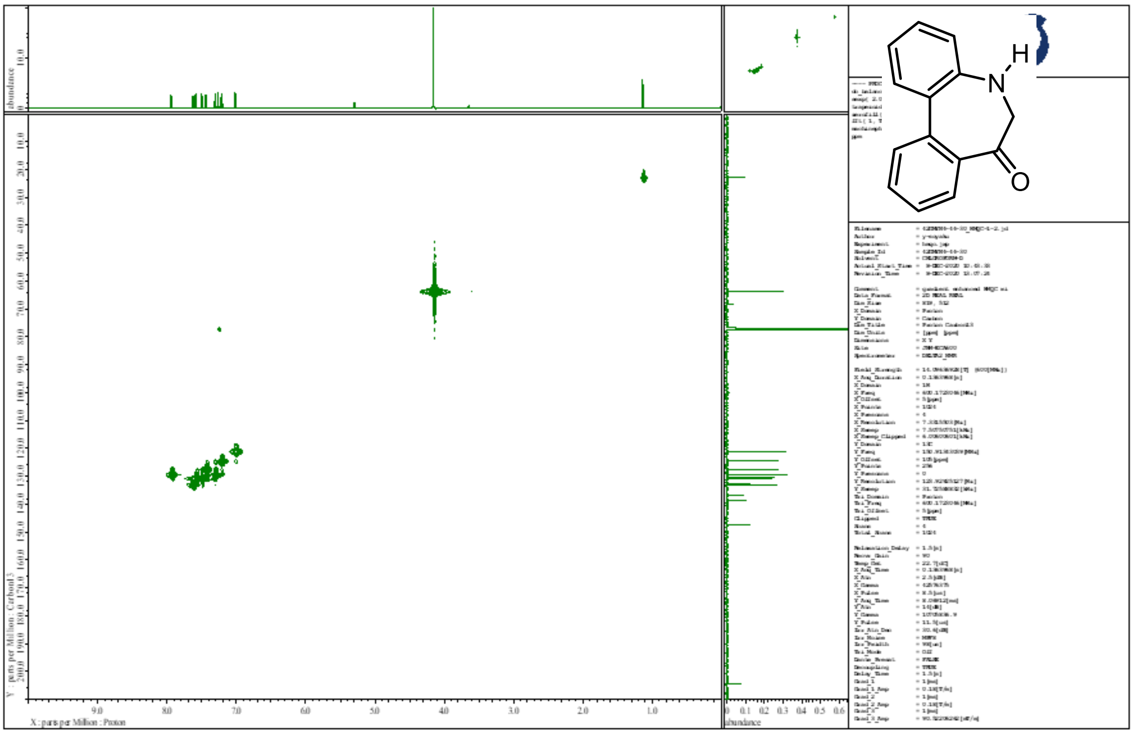

^{1}H-NMR (600 MHz, $CDCl_3$) of **IAa**

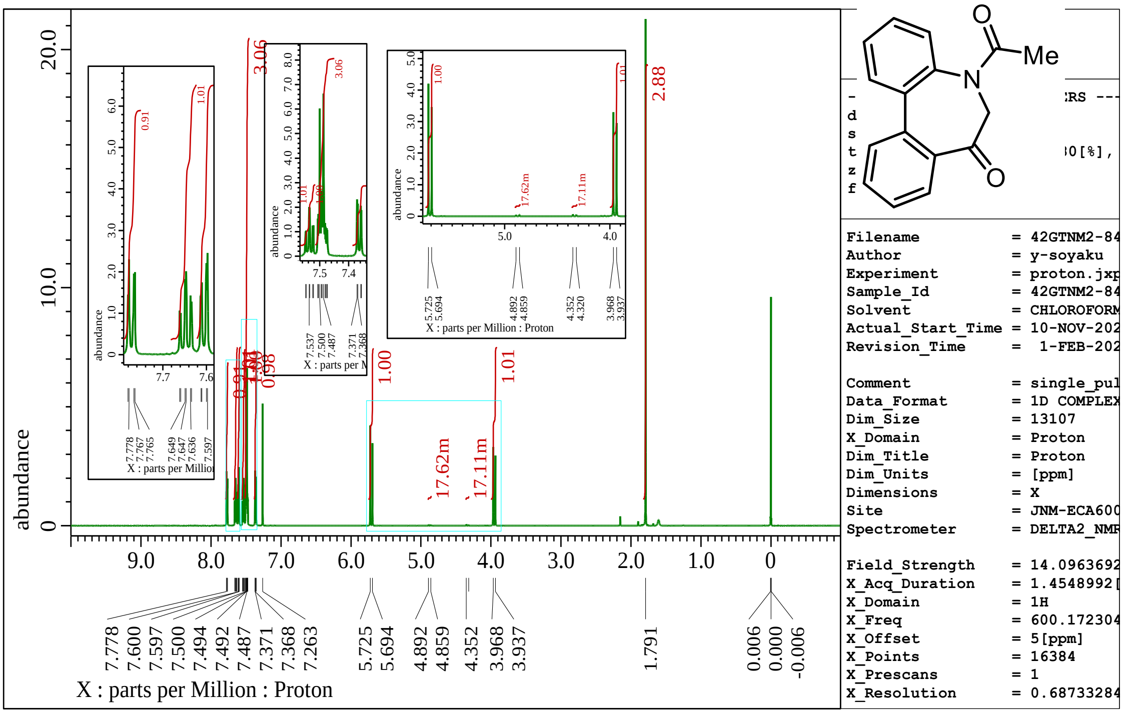

$^{13}C\{^{1}H\}$-NMR (150 MHz, $CDCl_3$) of **IAa**

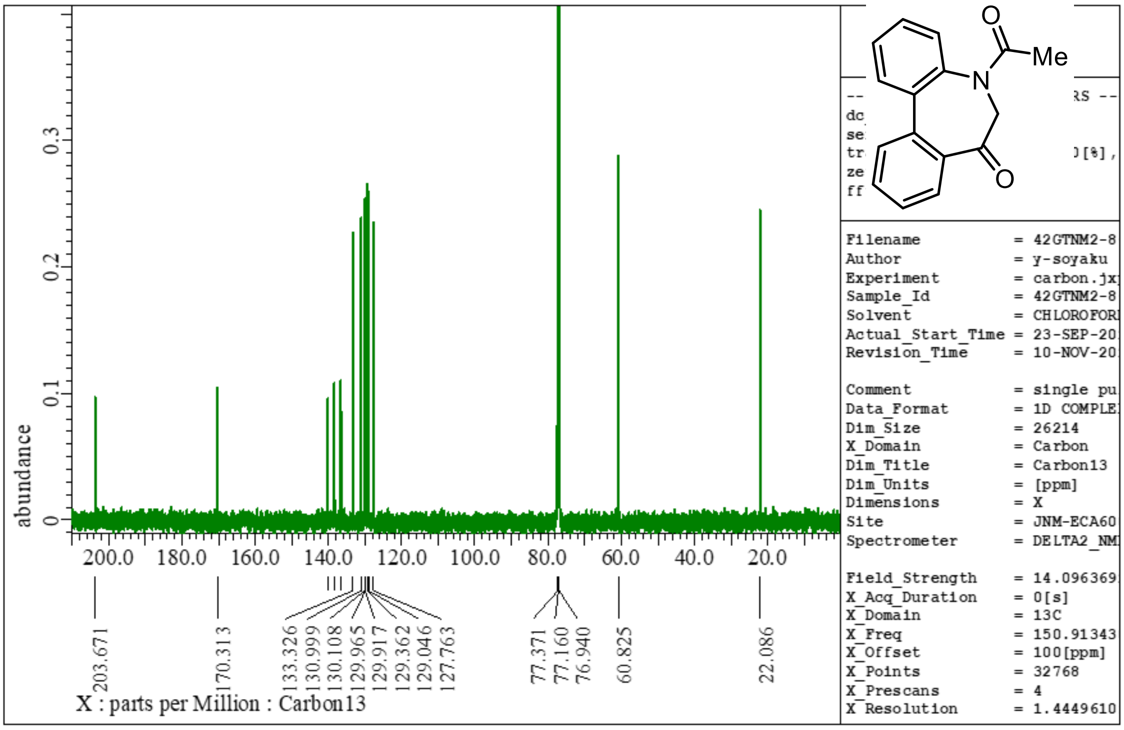

H-H COSY-NMR (600 MHz, $CDCl_3$) of **IAa**

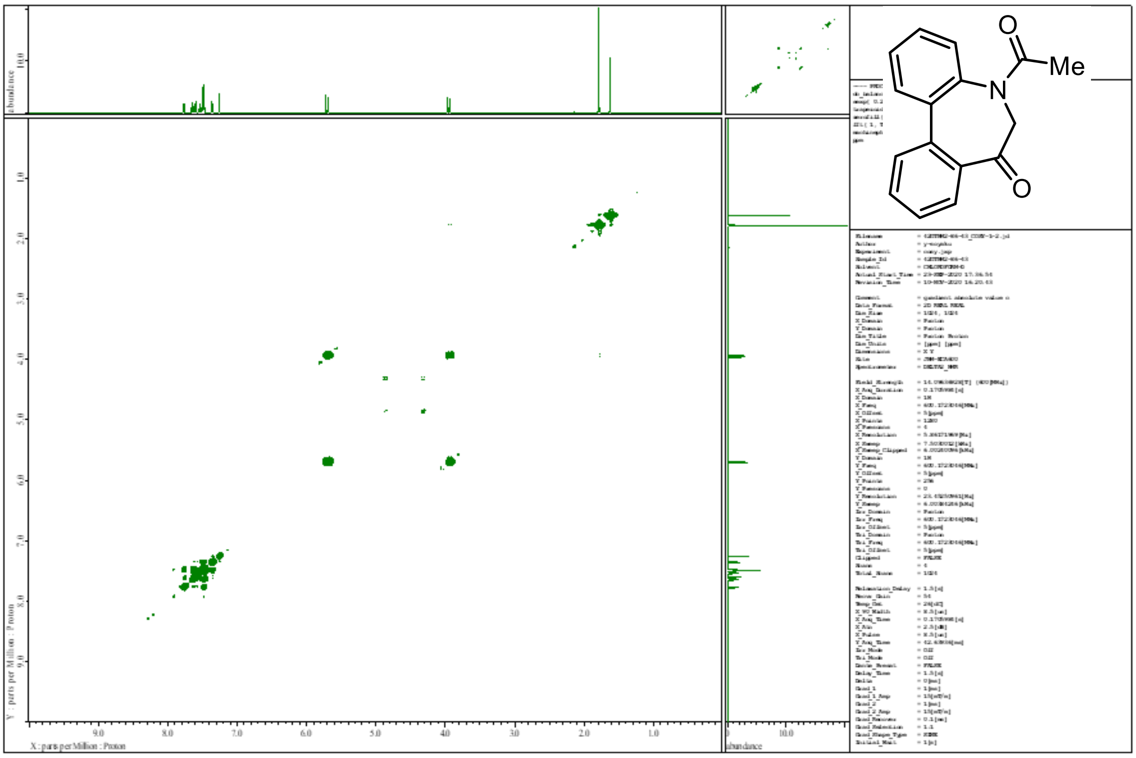

HMQC-NMR ($CDCl_3$) of **IAa**

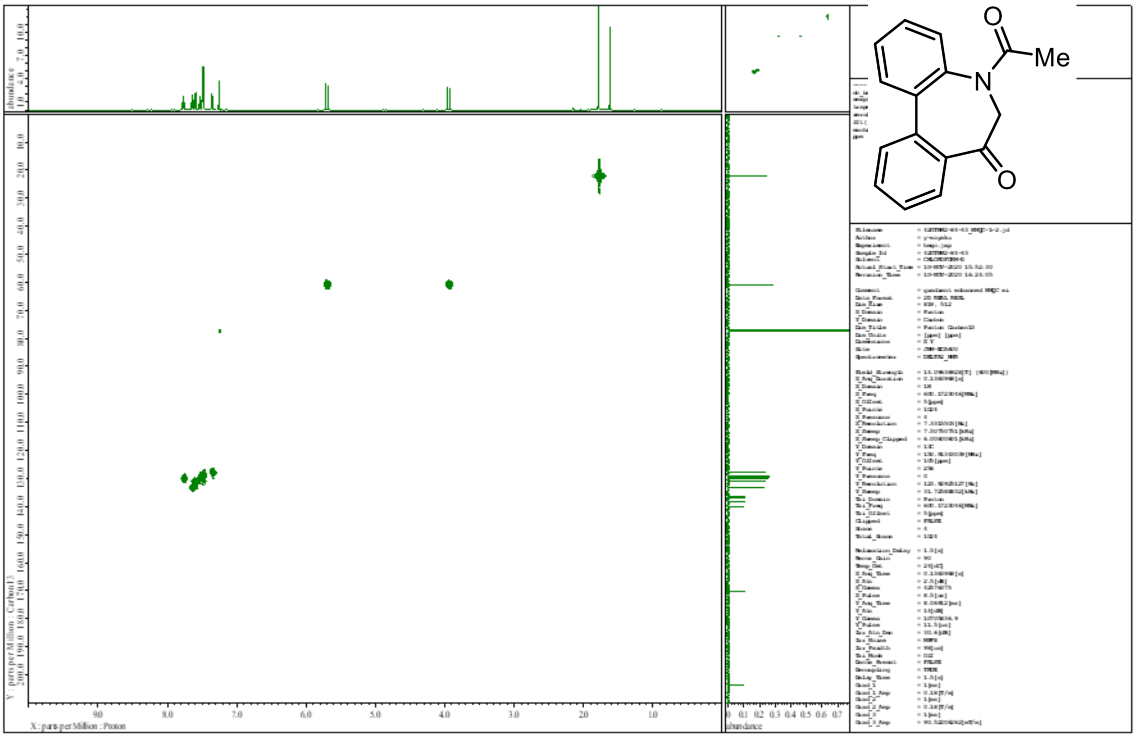

HMBC-NMR ($CDCl_3$) of **IAa**

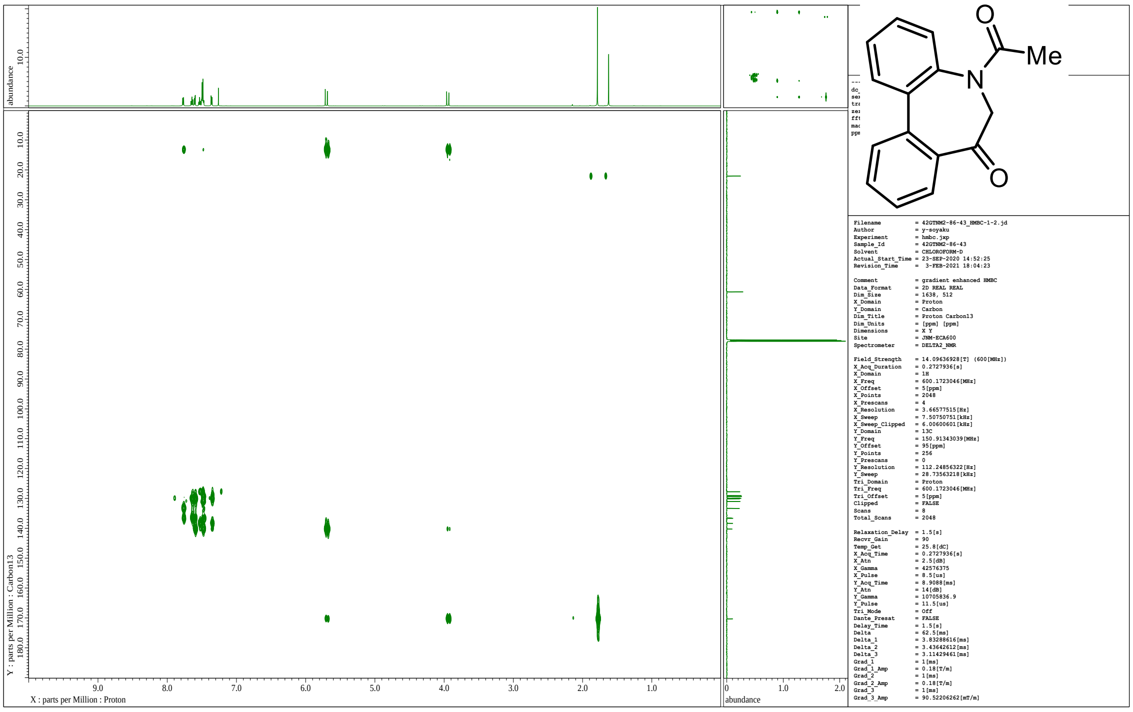

^{1}H-NMR (600 MHz, $CDCl_3$) of **IAg**

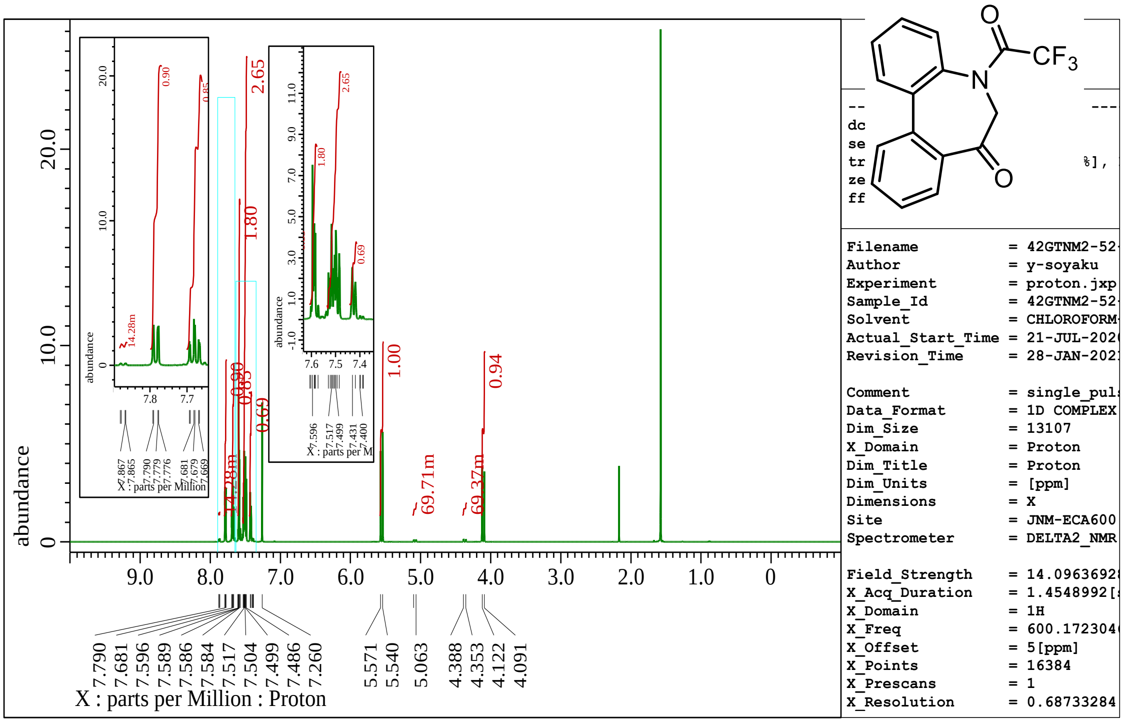

^{13}C{^{1}H }-NMR (150 MHz, $CDCl_3$) of **IAg**

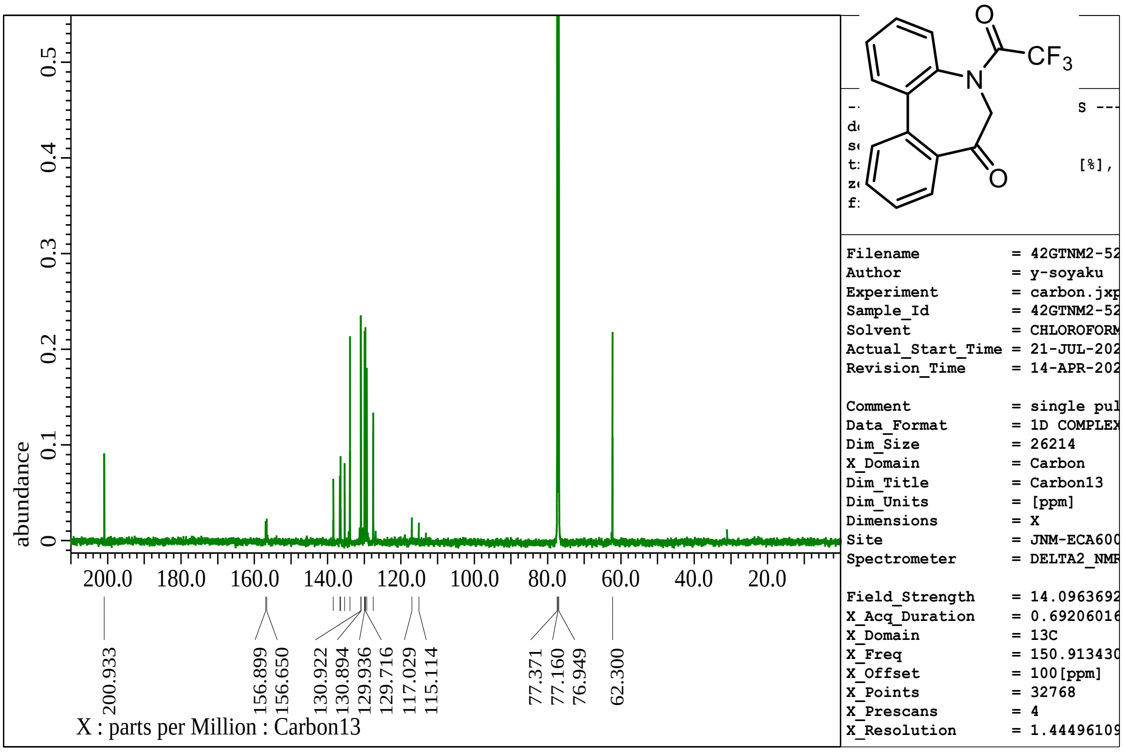

H-H COSY-NMR (600 MHz, $CDCl_3$) of **IAg**

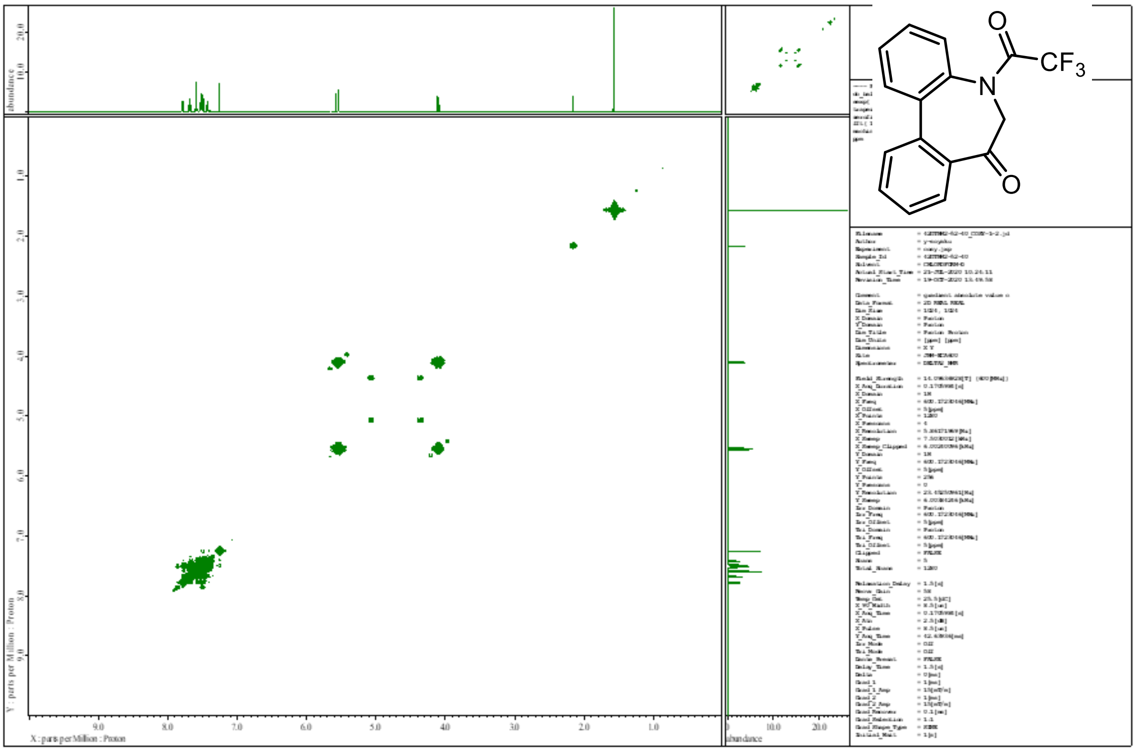

HMQC-NMR ($CDCl_3$) of **IAg**

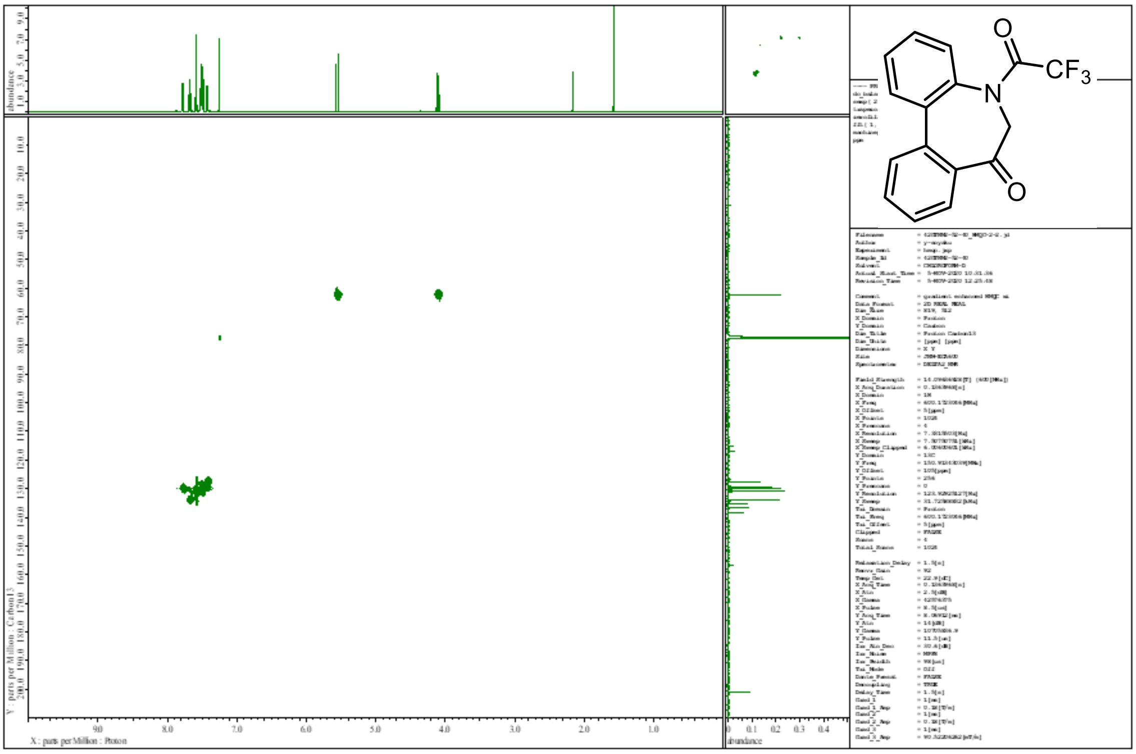

^{1}H-NMR (600 MHz, $CDCl_3$) of **IAh**

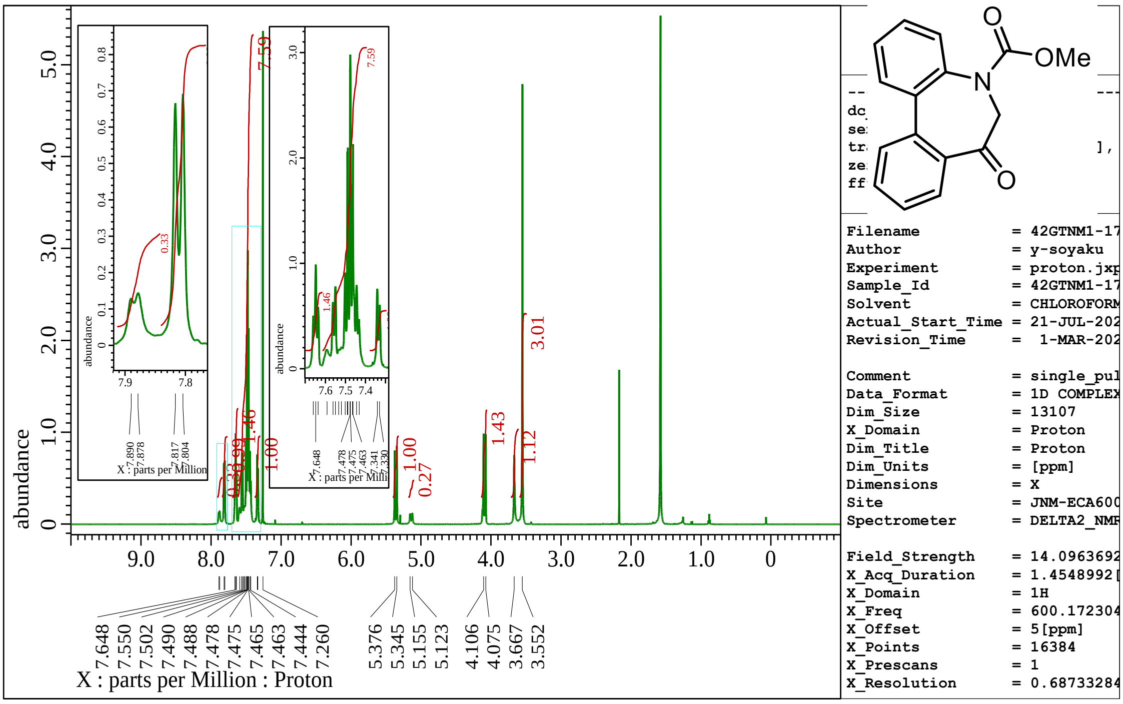

^{13}C{^{1}H }-NMR (150 MHz, $CDCl_3$) of **IAh**

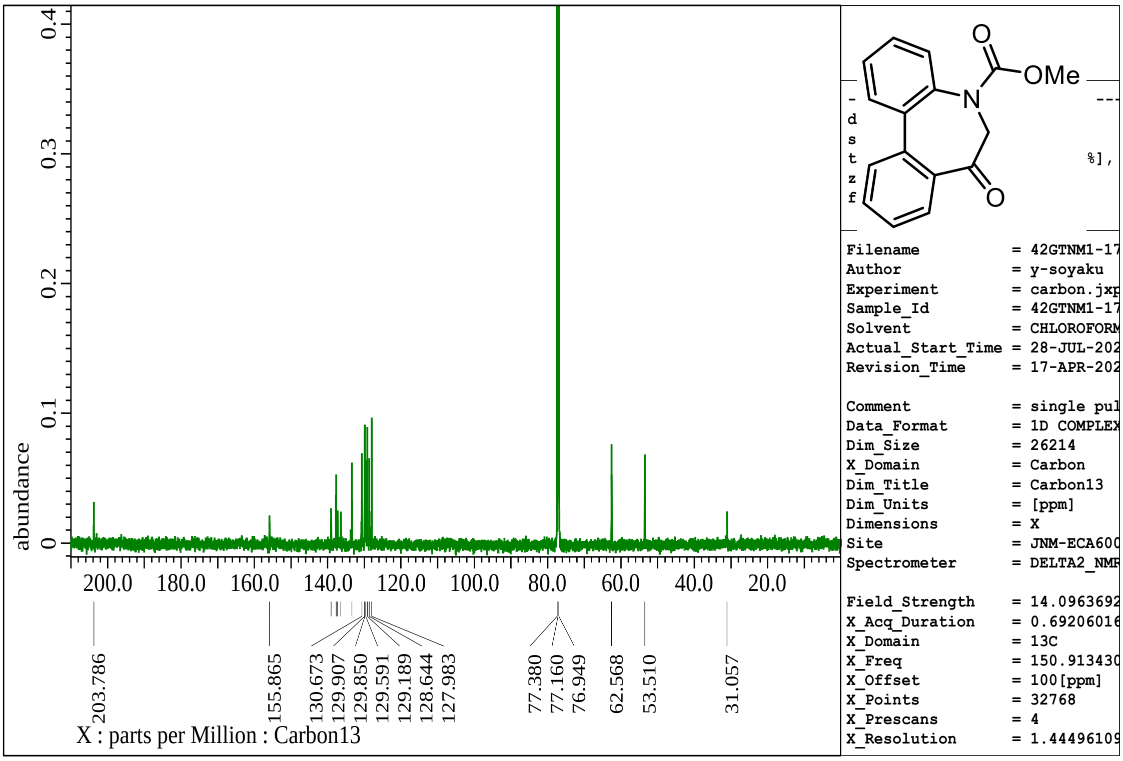

H-H COSY-NMR (600 MHz, $CDCl_3$) of **IAh**

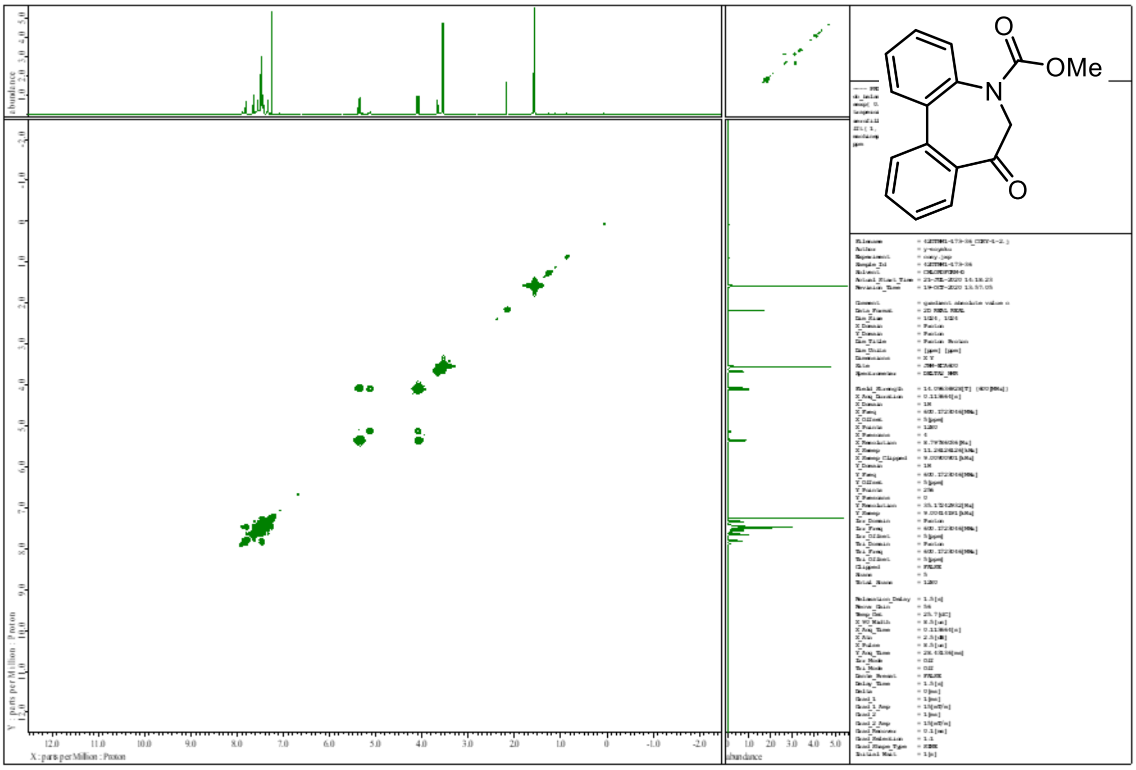

HMQC-NMR ($CDCl_3$) of **IAh**

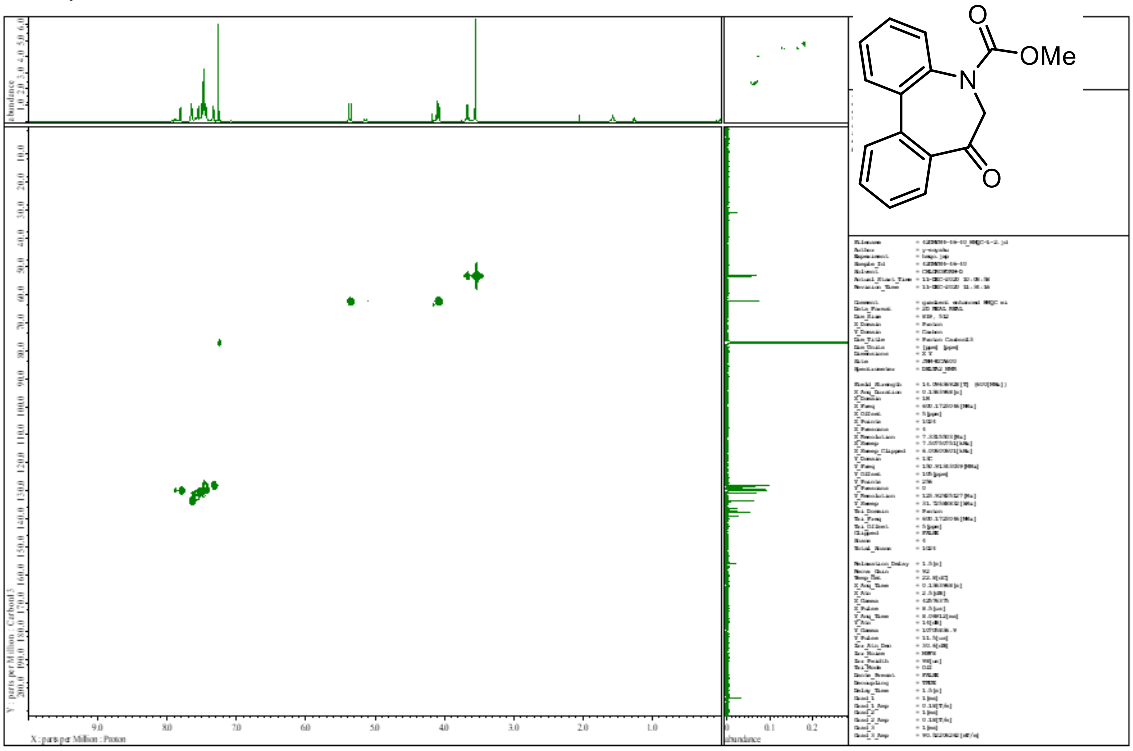

^{1}H-NMR (600 MHz, $CDCl_3$) of **IBg**

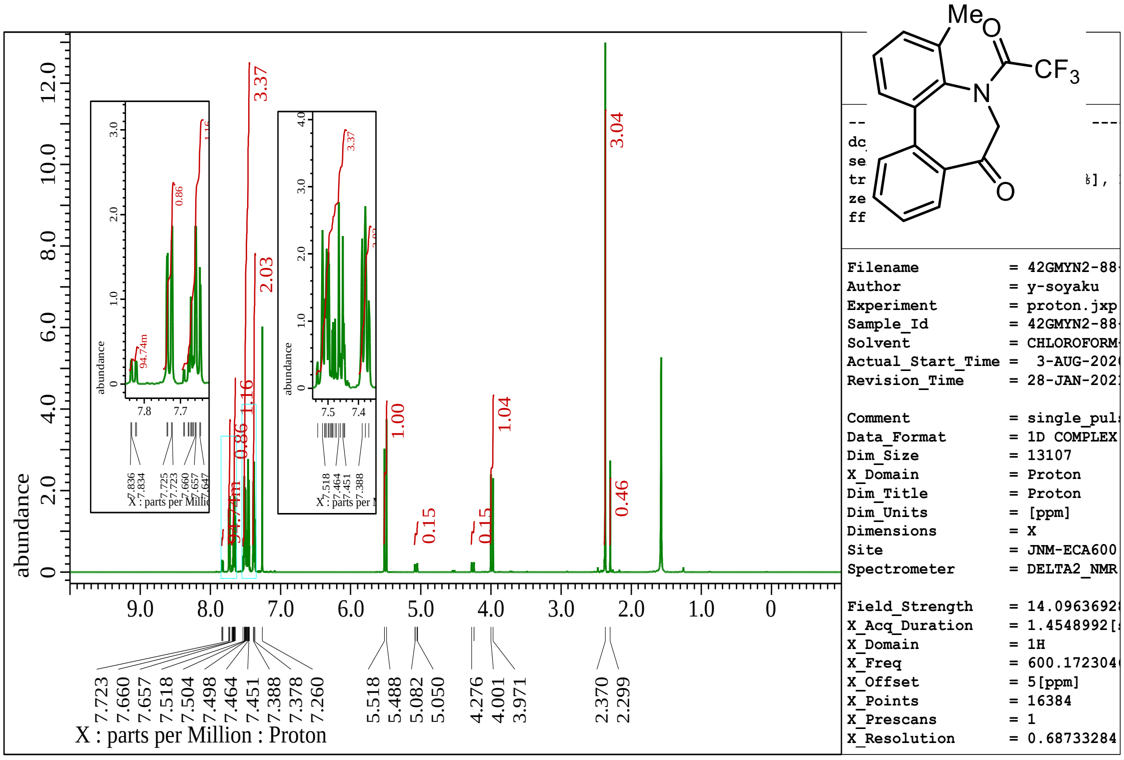

^{13}C{^{1}H }-NMR (150 MHz, $CDCl_3$) of **IBg**

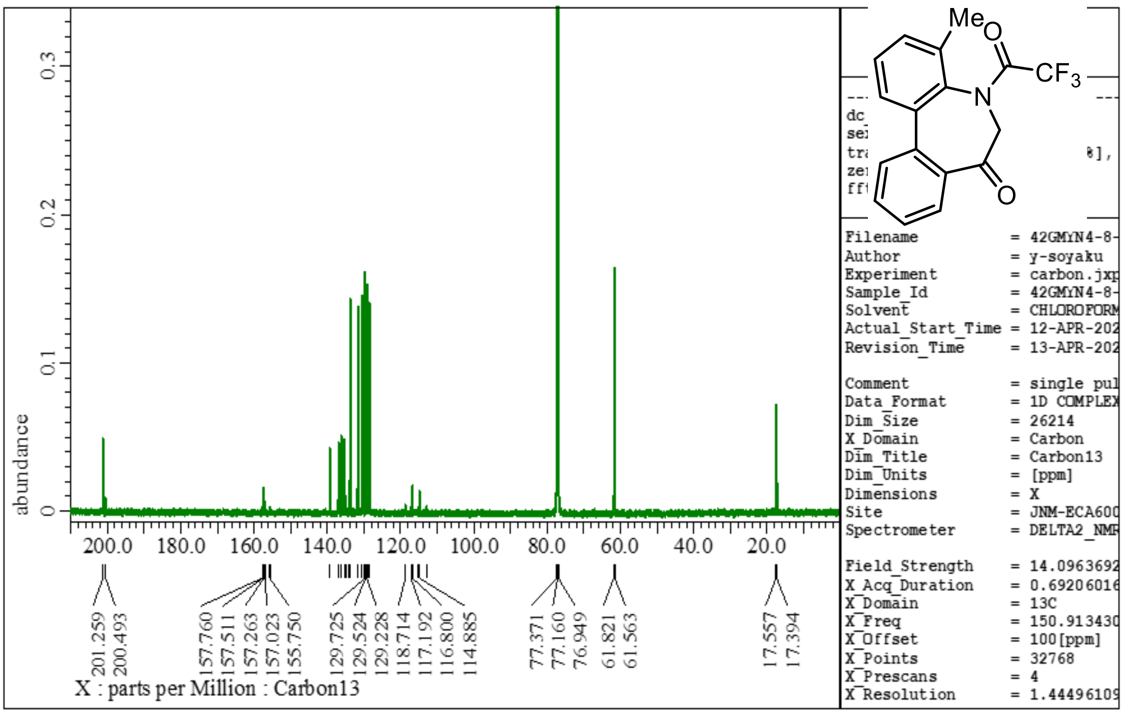

H-H COSY-NMR (600 MHz, $CDCl_3$) of **IBg**

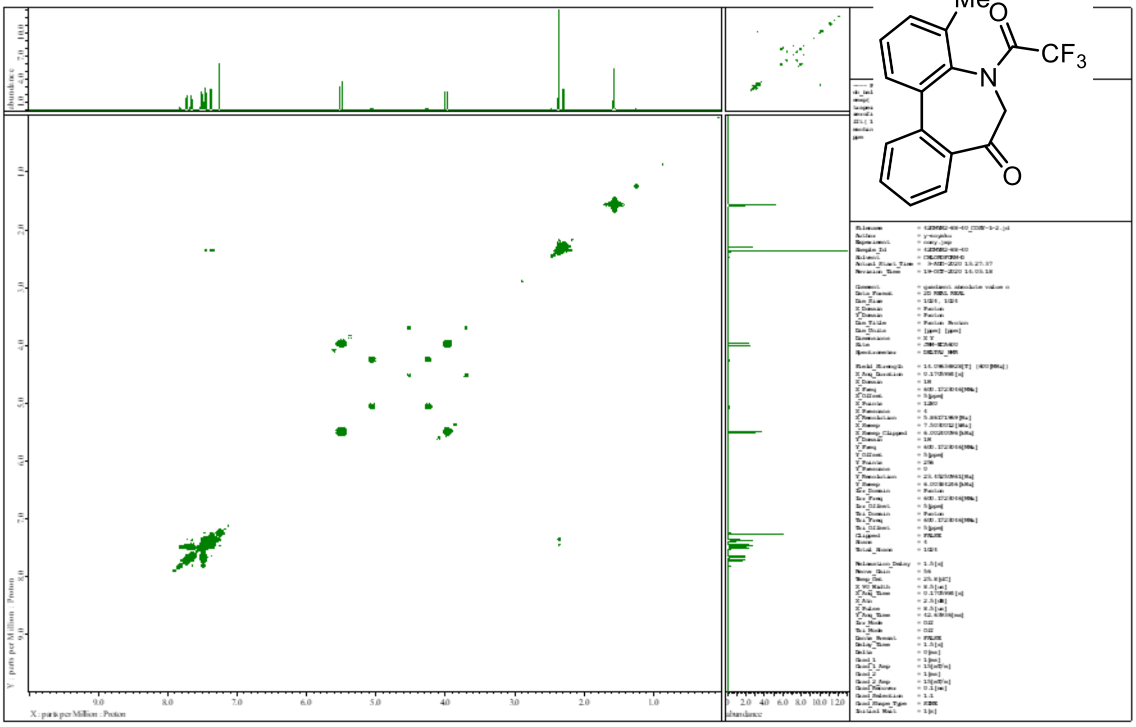

HMQC-NMR ($CDCl_3$) of **IBg**

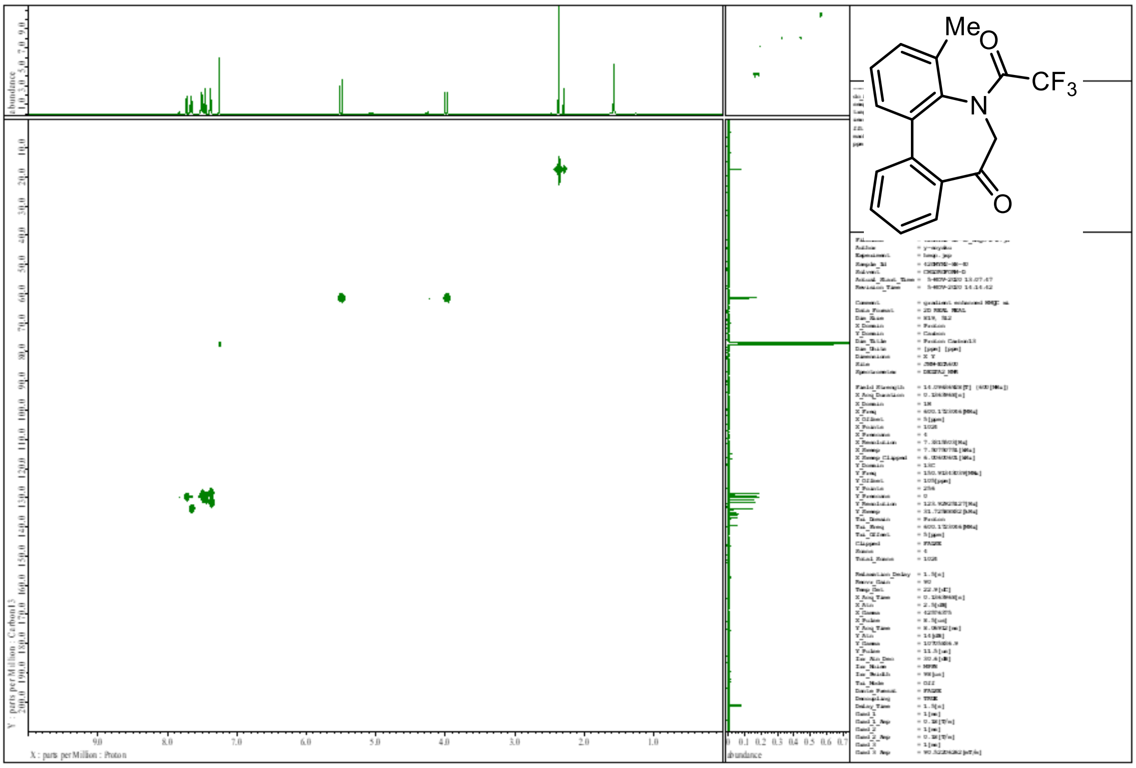

^{1}H-NMR (600 MHz, $CDCl_3$) of **IBh**

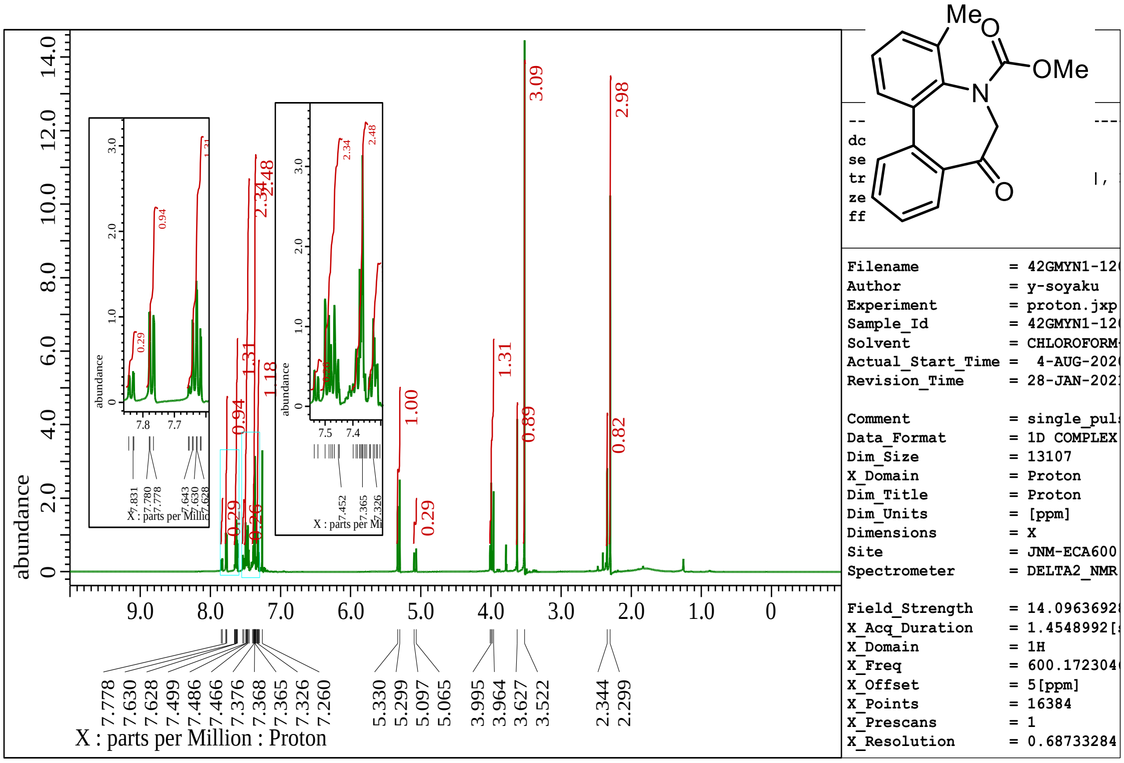

^{13}C{^{1}H }-NMR (150 MHz, $CDCl_3$) of **IBh**

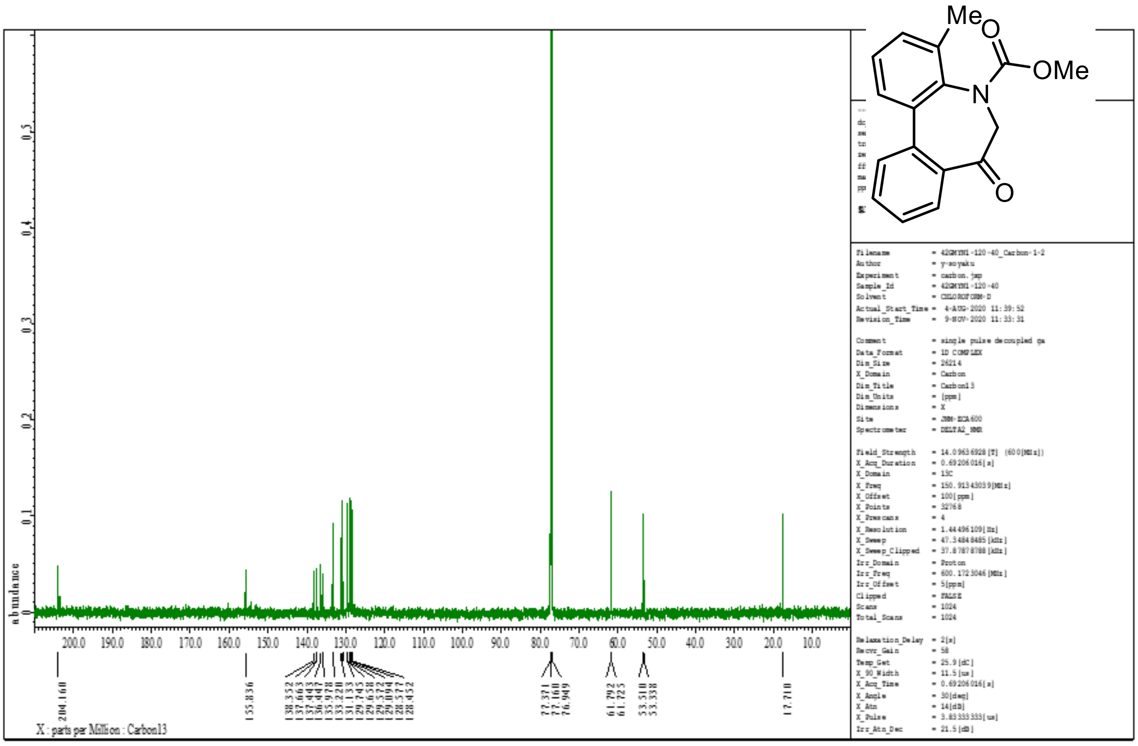

H-H COSY-NMR (600 MHz, $CDCl_3$) of **IBh**

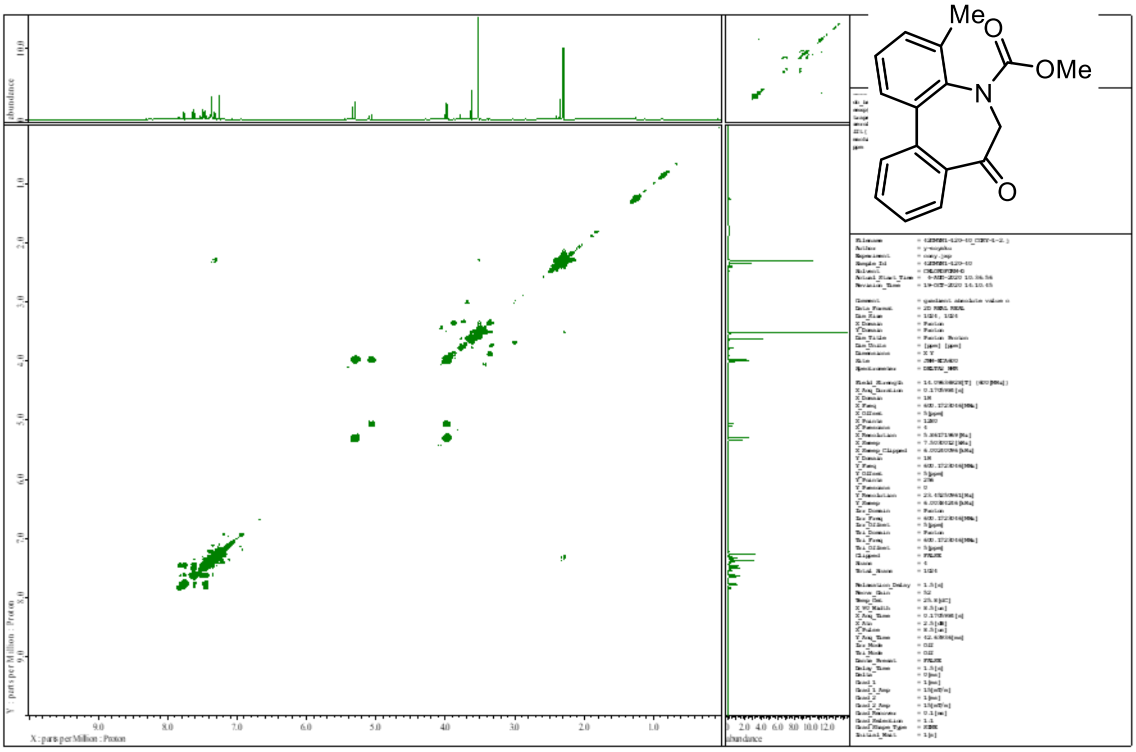

HMQC-NMR ($CDCl_3$) of **IBh**

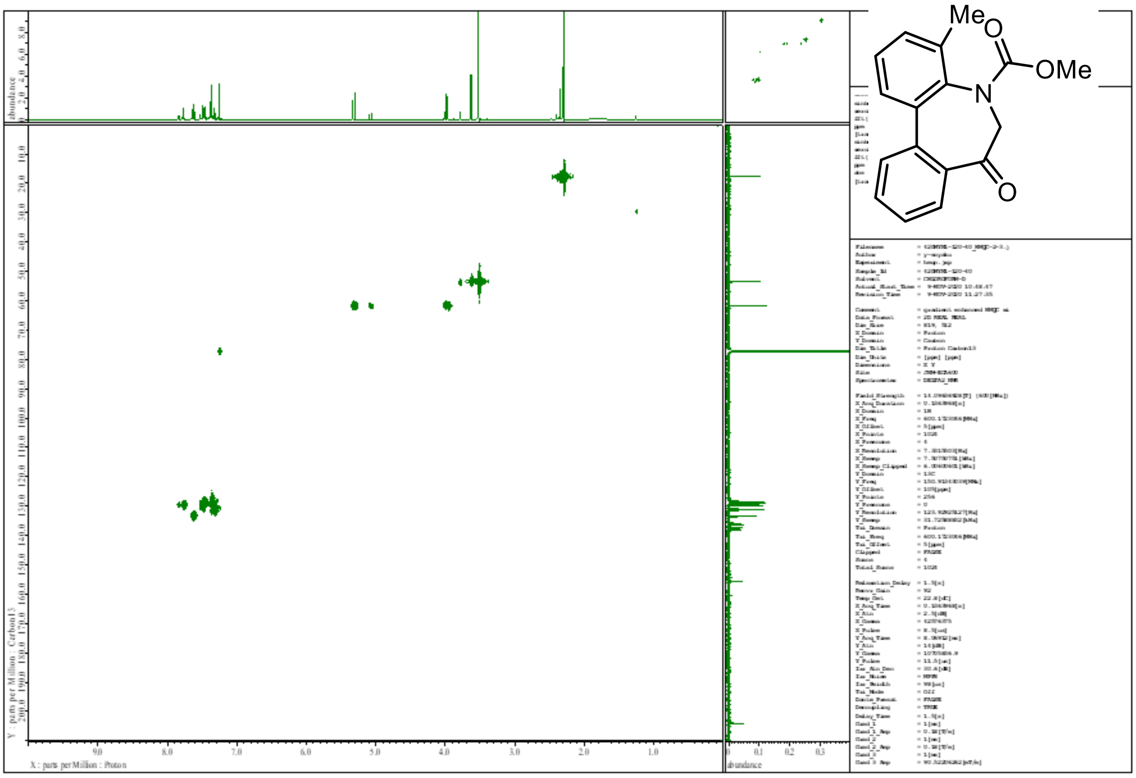

^{1}H-NMR (600 MHz, $CDCl_3$) of **IIAc**

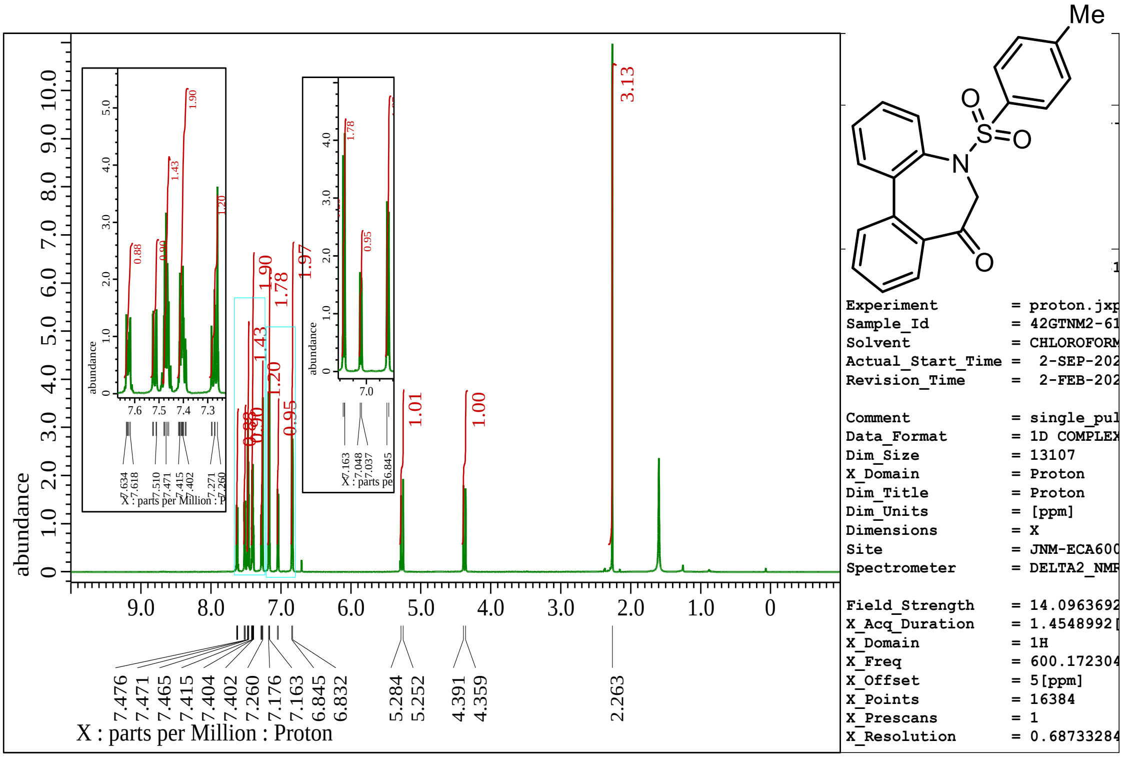

$^{13}C\{^{1}H\}$-NMR (150 MHz, $CDCl_3$) of **IIAc**

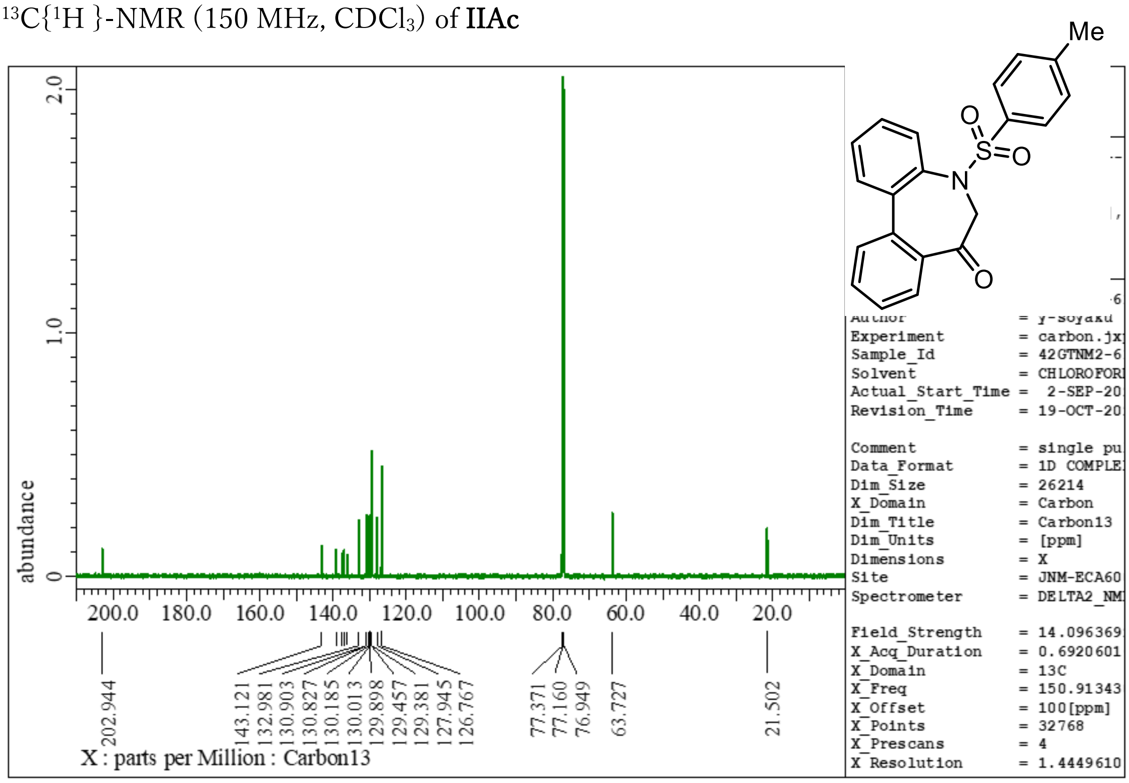

H-H COSY-NMR (600 MHz, $CDCl_3$) of **IIAc**

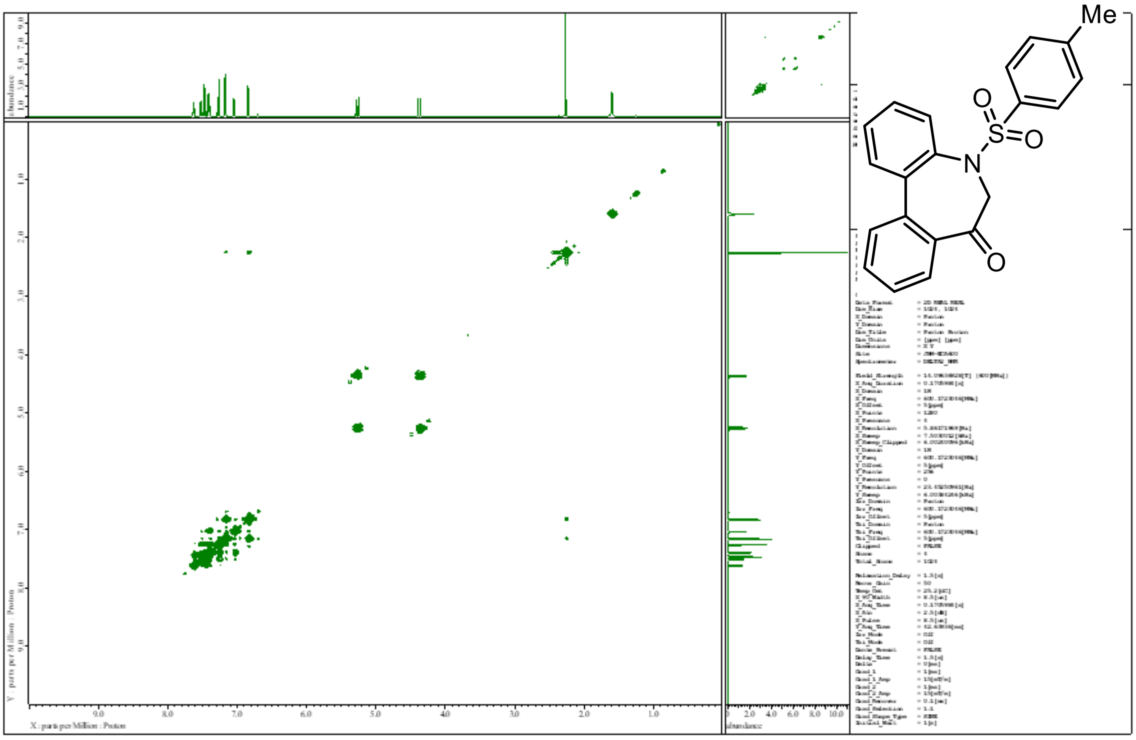

HMQC-NMR ($CDCl_3$) of **IIAc**

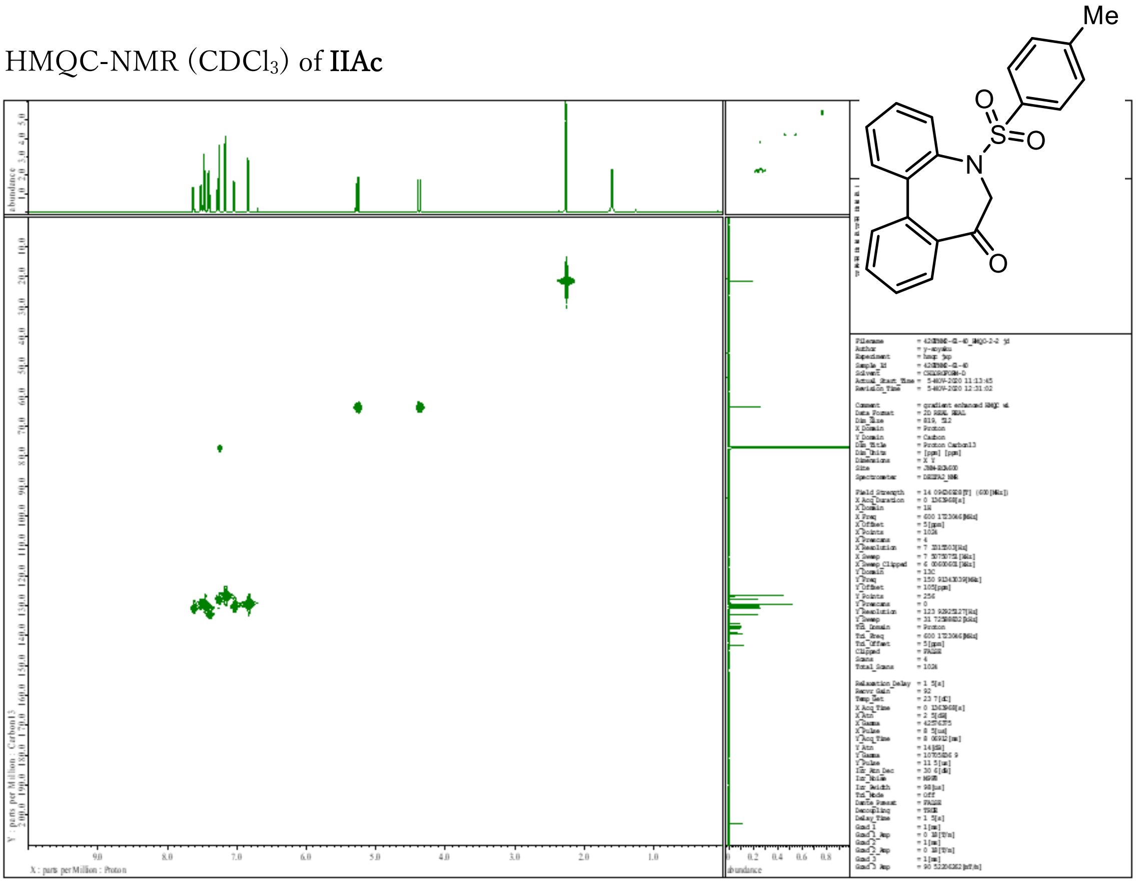

^{1}H-NMR (600 MHz, $CDCl_3$) of **IIAd**

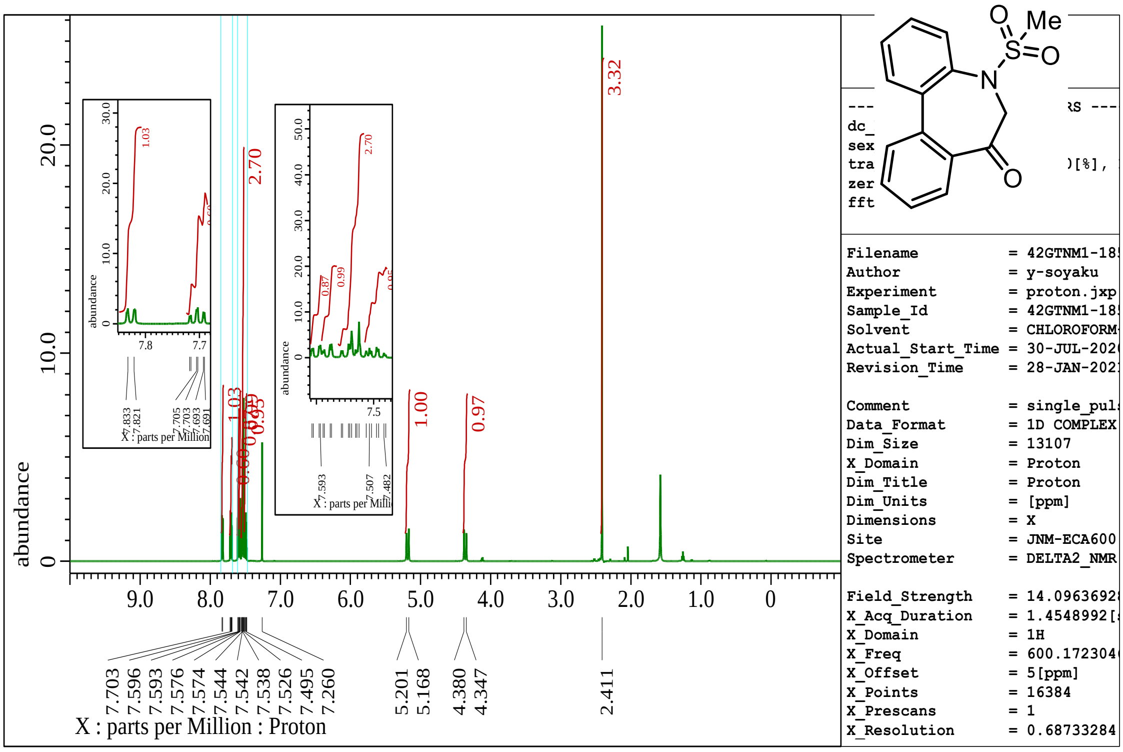

^{13}C{^{1}H }-NMR (150 MHz, $CDCl_3$) of **IIAd**

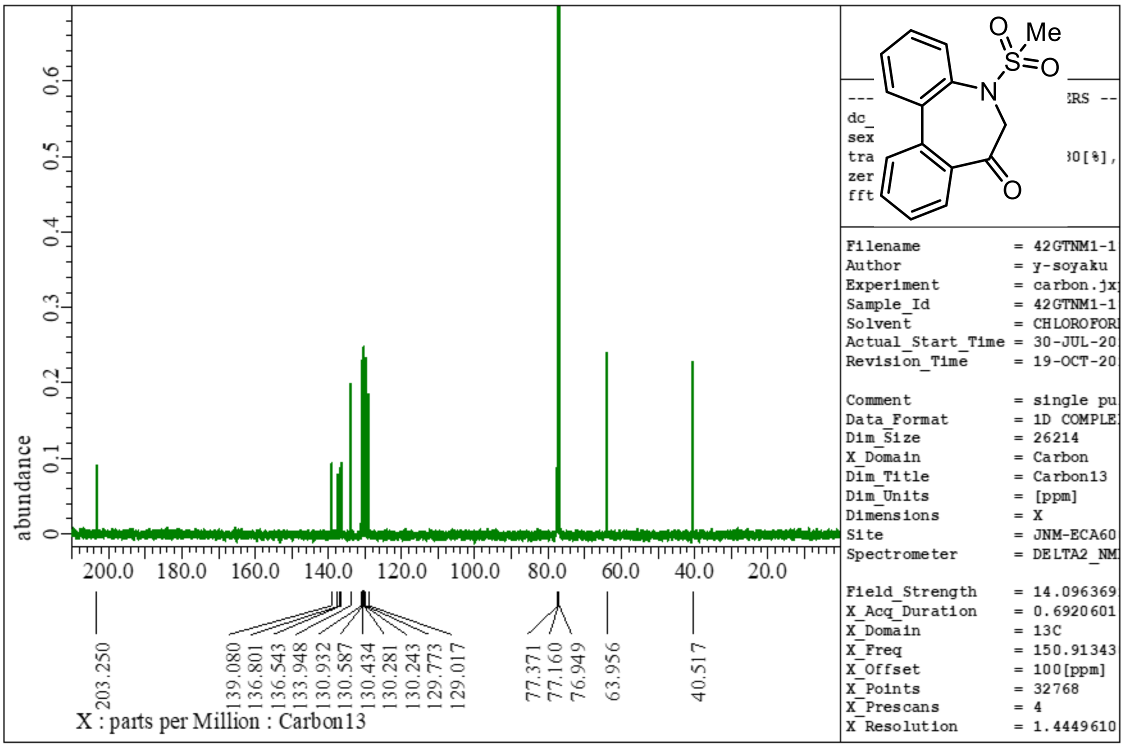

H-H COSY-NMR (600 MHz, $CDCl_3$) of **IIAd**

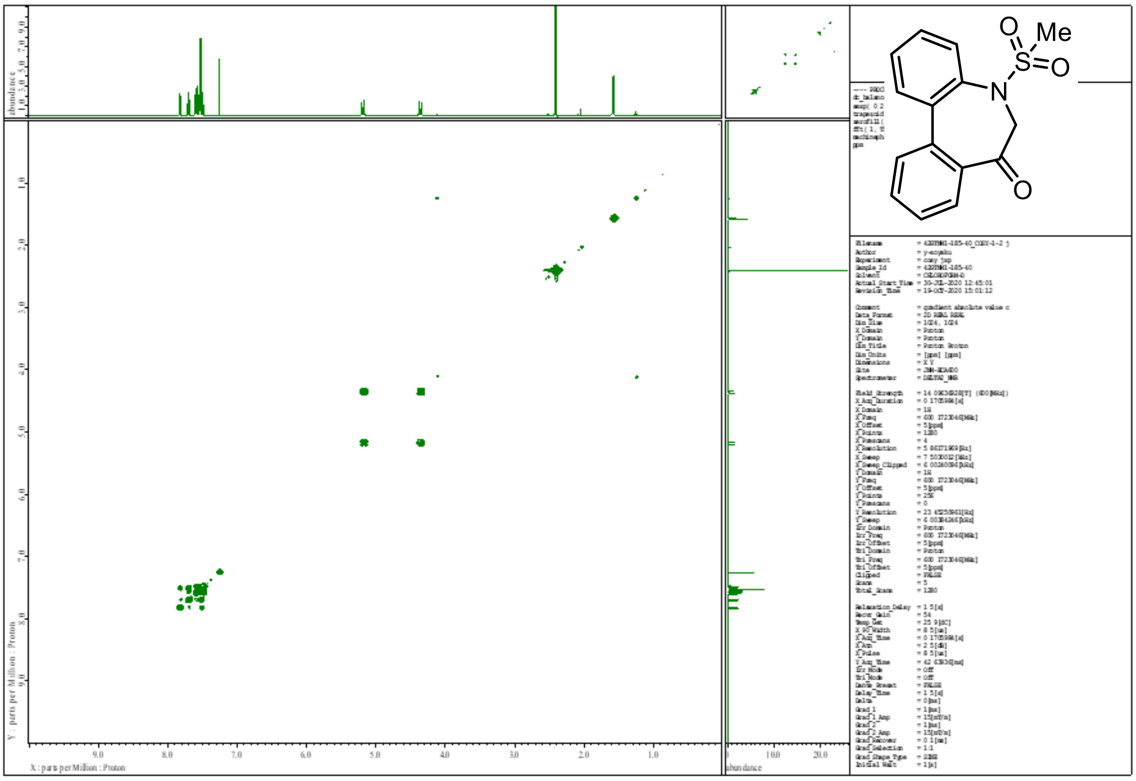

HMQC-NMR ($CDCl_3$) of **IIAd**

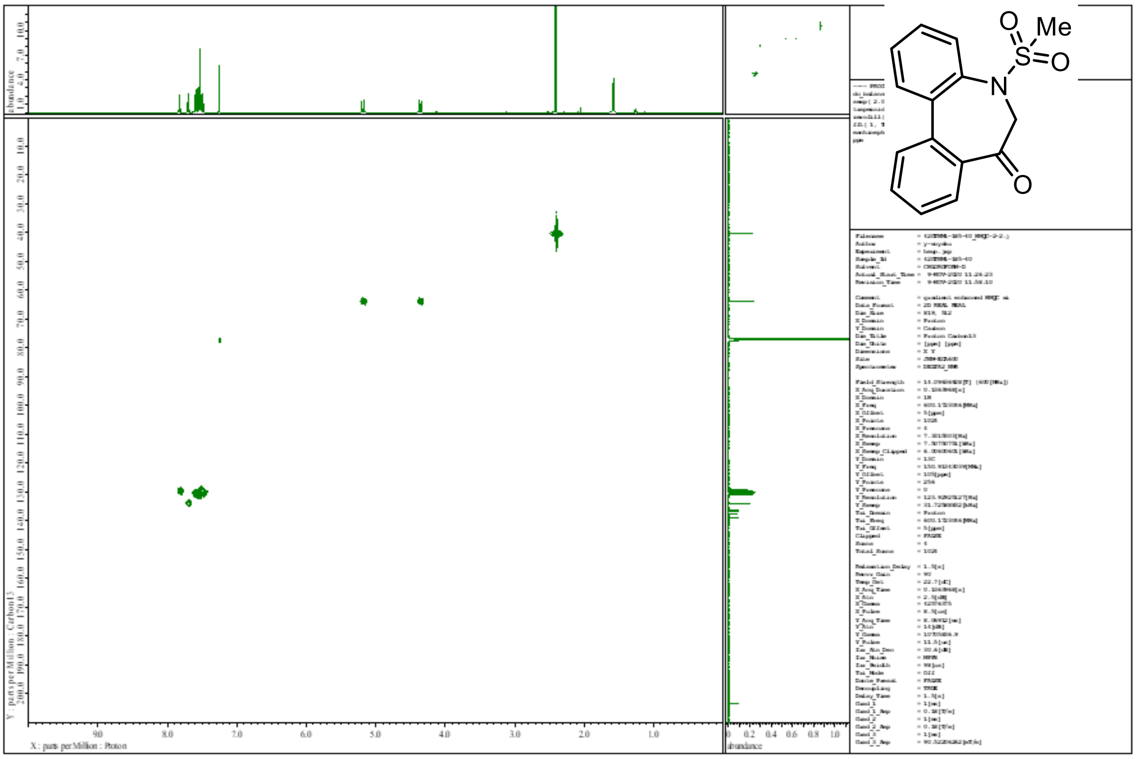

^{1}H-NMR (600 MHz, $CDCl_3$) of **IIAe**

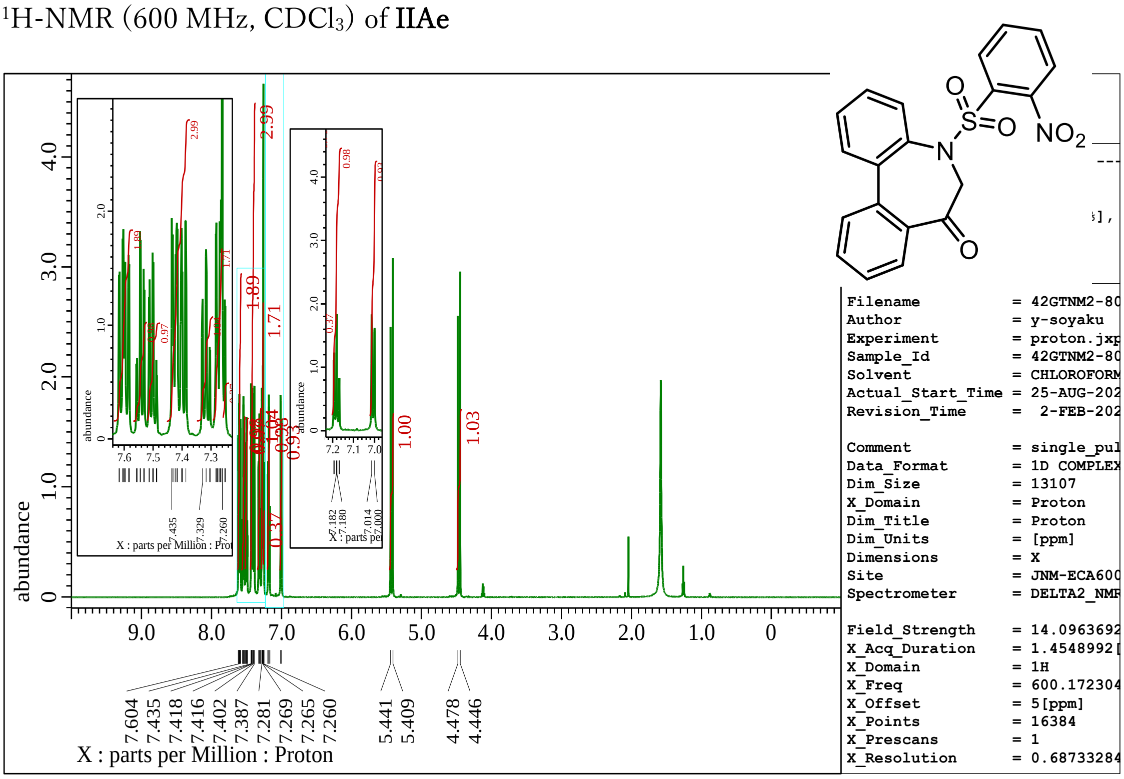

^{13}C{^{1}H }-NMR (150 MHz, $CDCl_3$) of **IIAe**

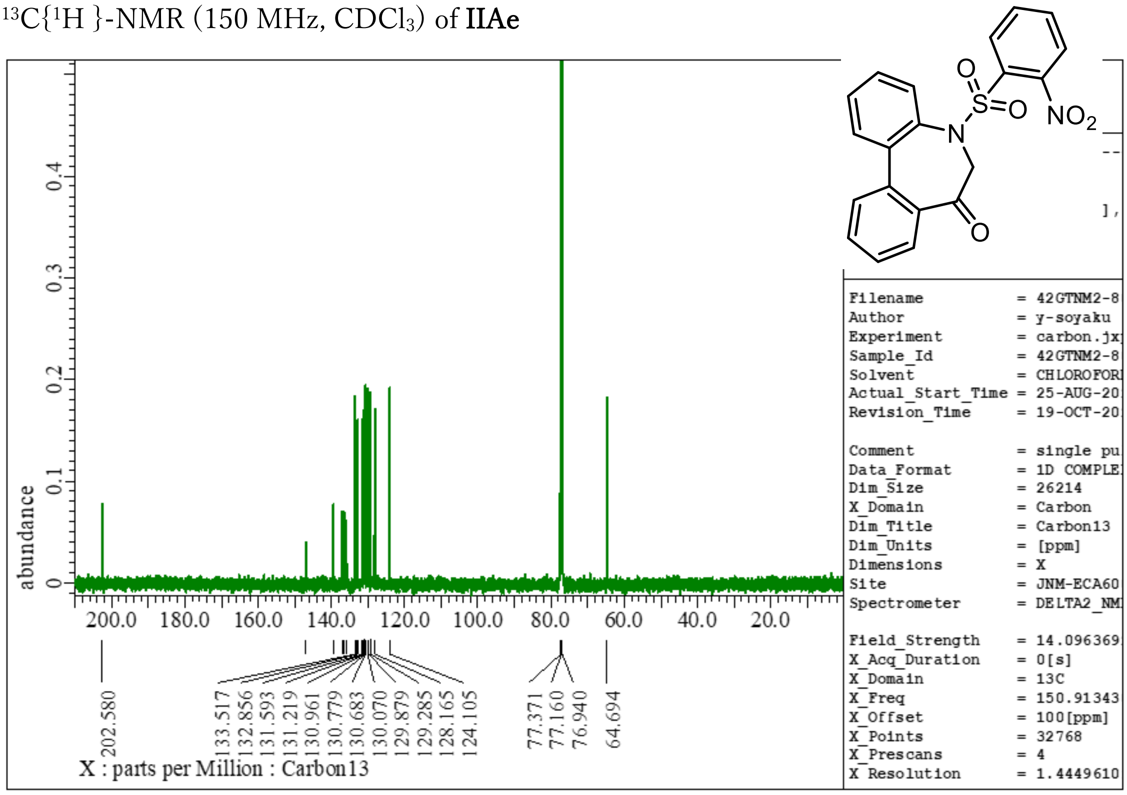

H-H COSY-NMR (600 MHz, $CDCl_3$) of **IIAe**

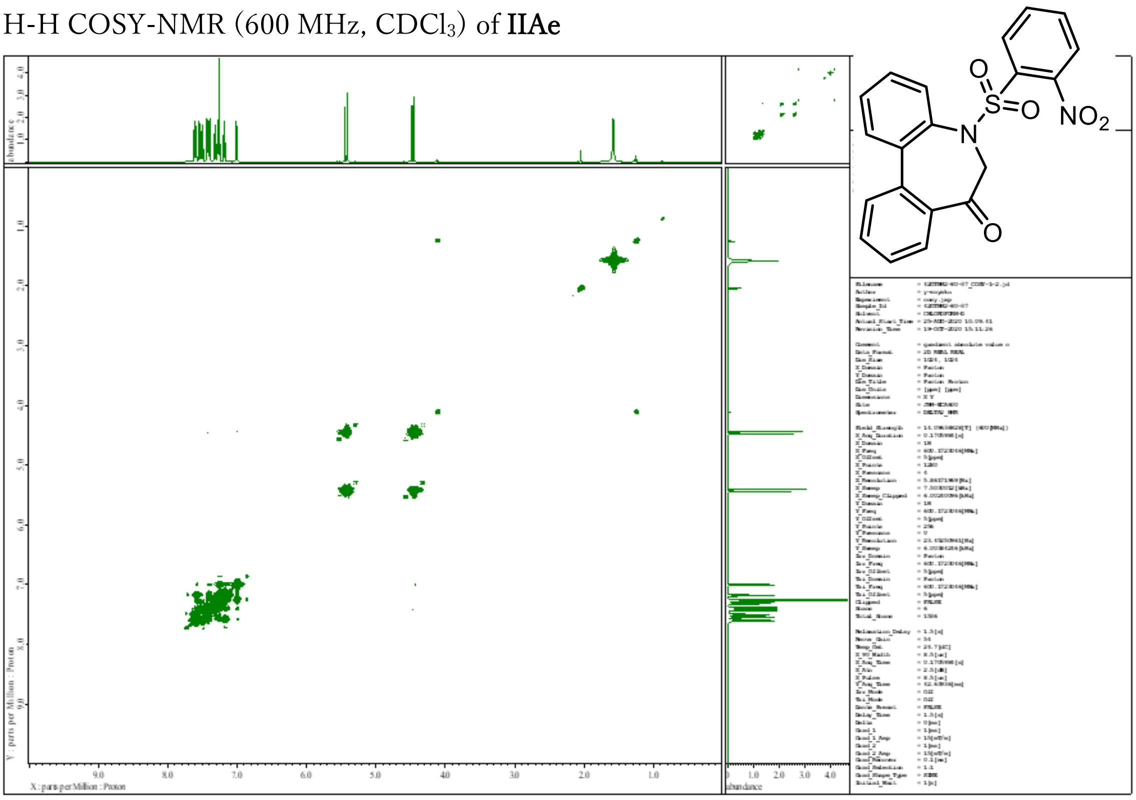

HMQC-NMR ($CDCl_3$) of **IIAe**

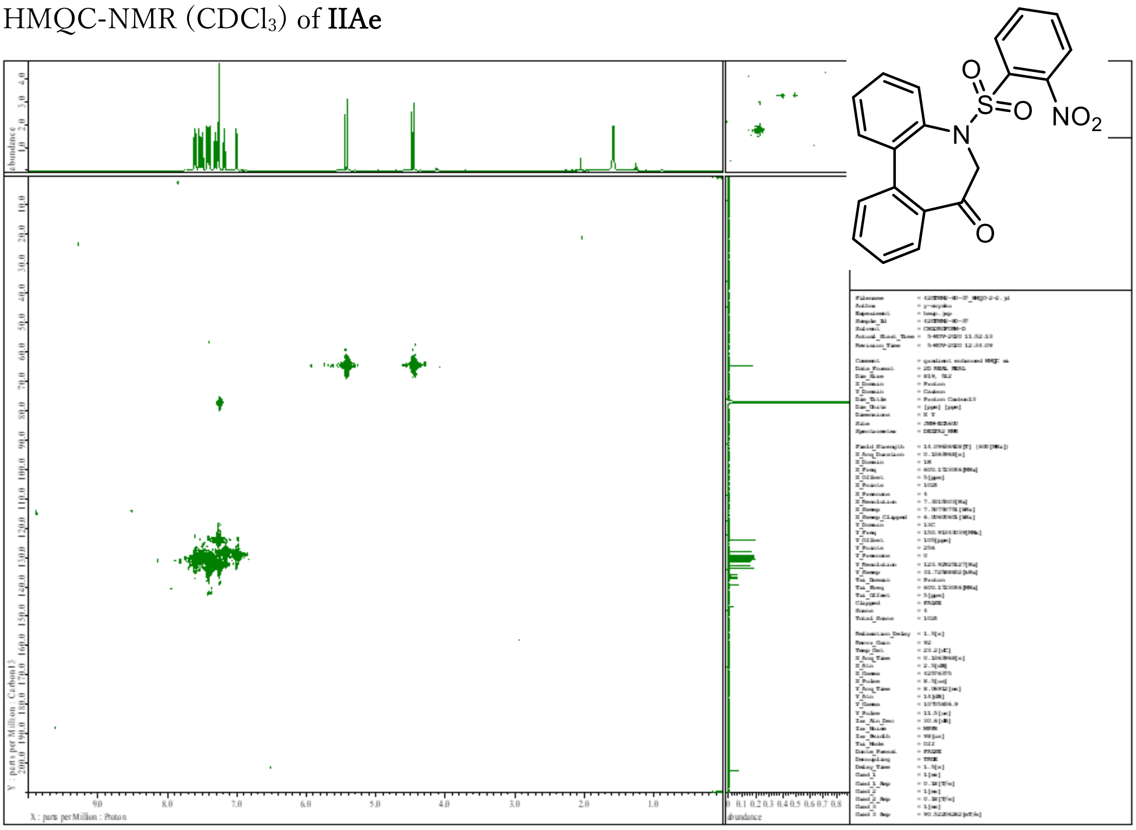

^{1}H-NMR (600 MHz, $CDCl_3$) of **IIAf**

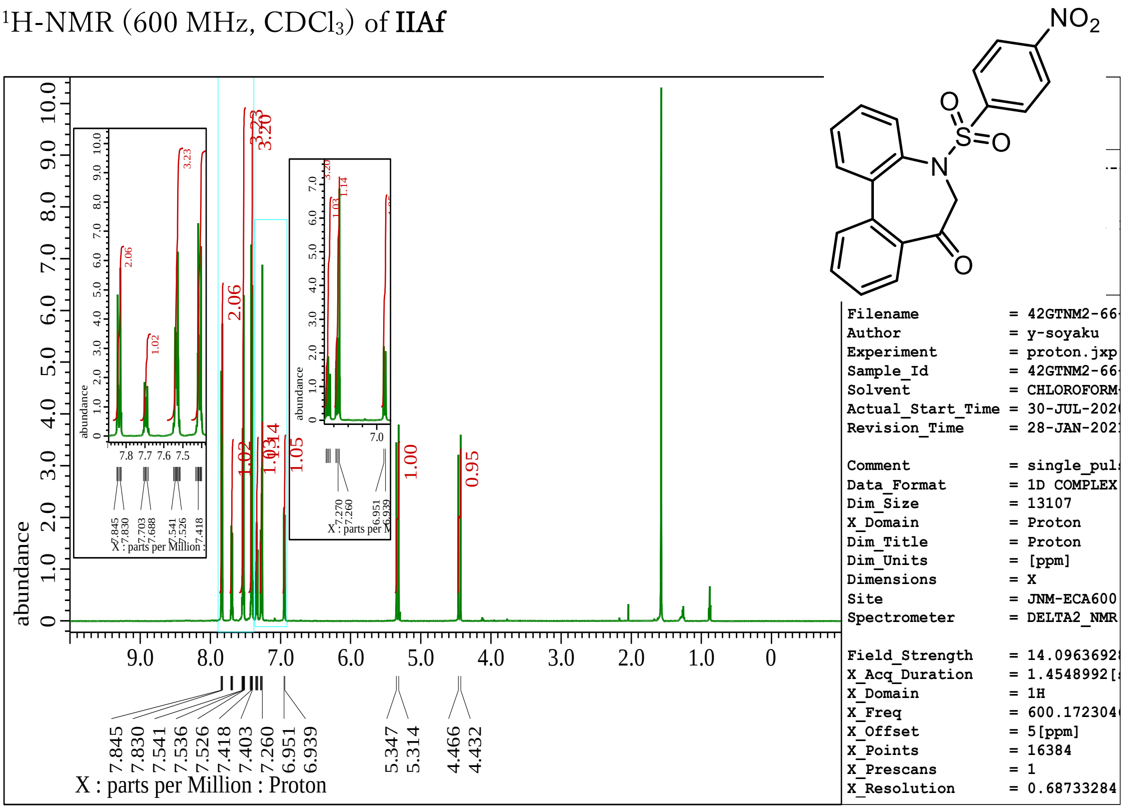

$^{13}C\{^{1}H\}$-NMR (150 MHz, $CDCl_3$) of **IIAf**

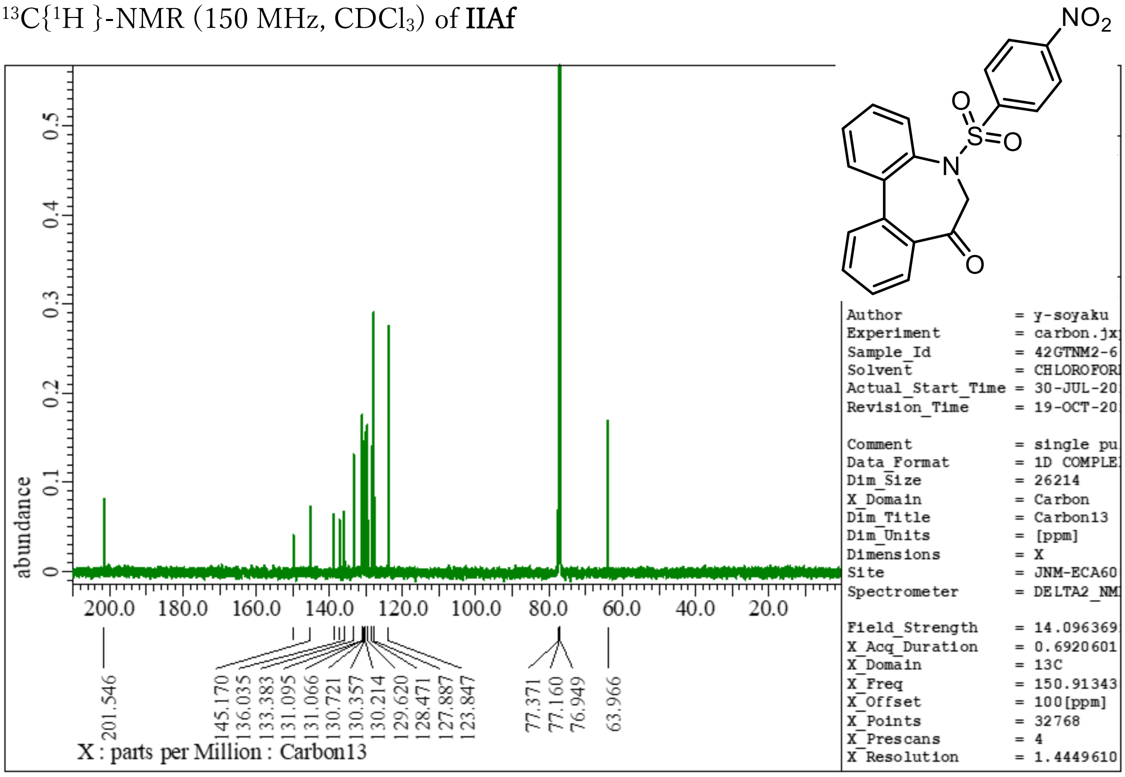

H-H COSY-NMR (600 MHz, $CDCl_3$) of **IIAf**

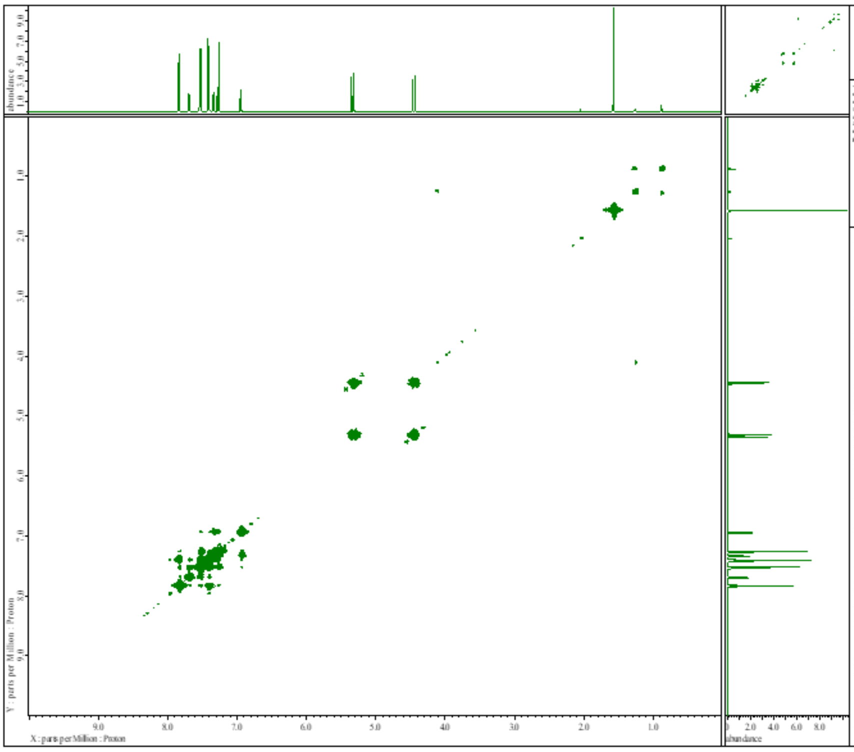

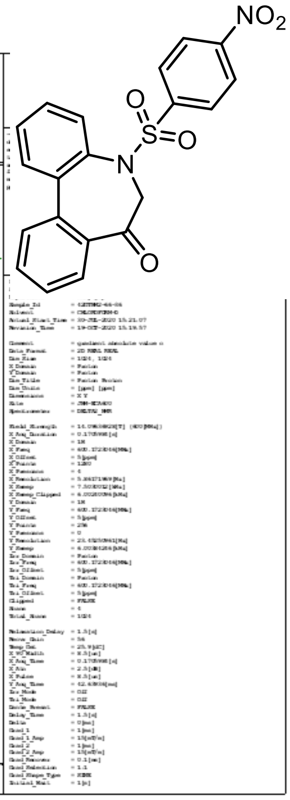

HMQC-NMR ($CDCl_3$) of **IIAf**

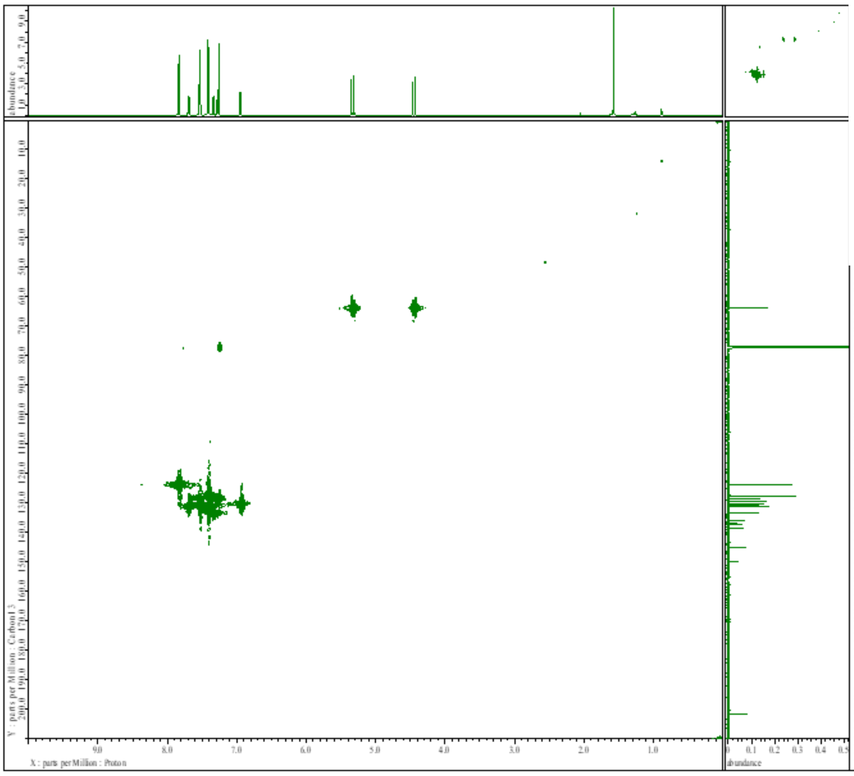

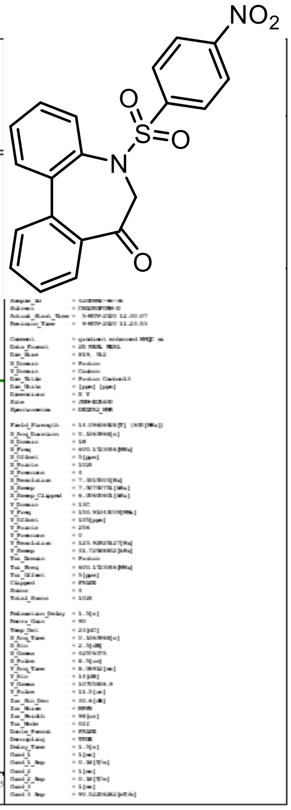

^{1}H-NMR (600 MHz, $CDCl_3$) of **IIBc**

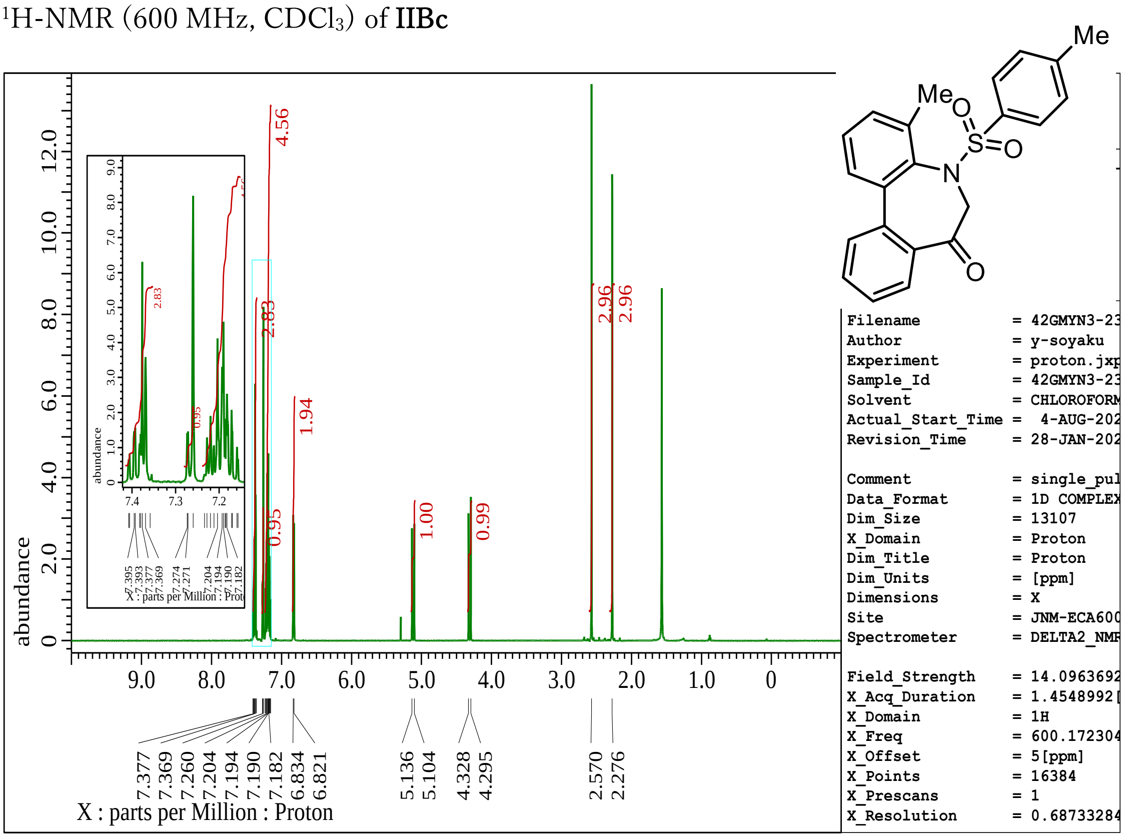

^{13}C{^{1}H }-NMR (150 MHz, $CDCl_3$) of **IIBc**

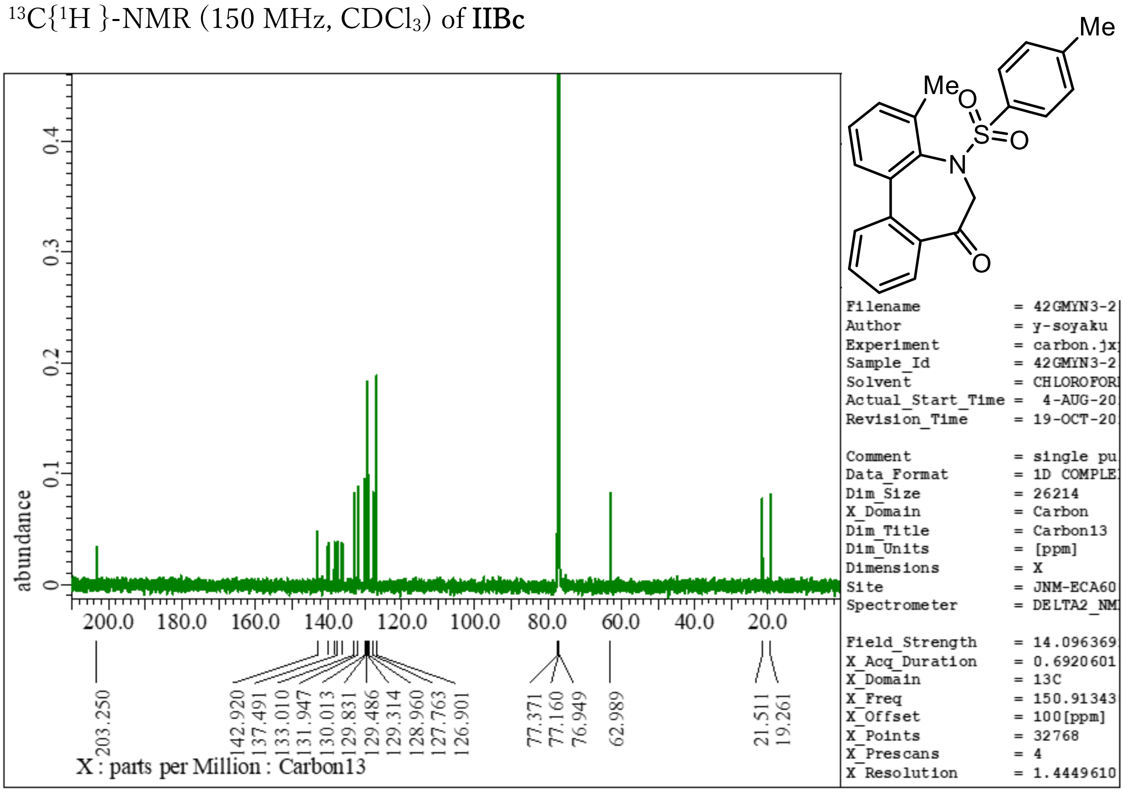

H-H COSY-NMR (600 MHz, $CDCl_3$) of **IIBc**

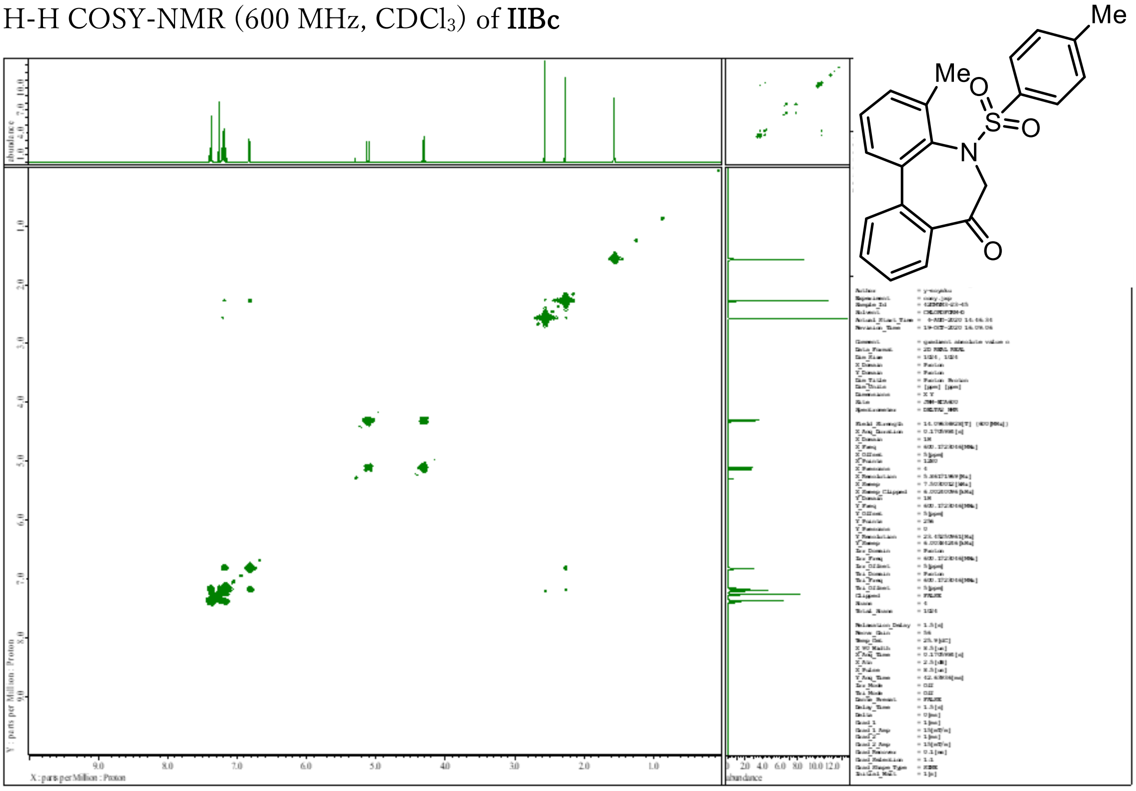

HMQC-NMR ($CDCl_3$) of **IIBc**

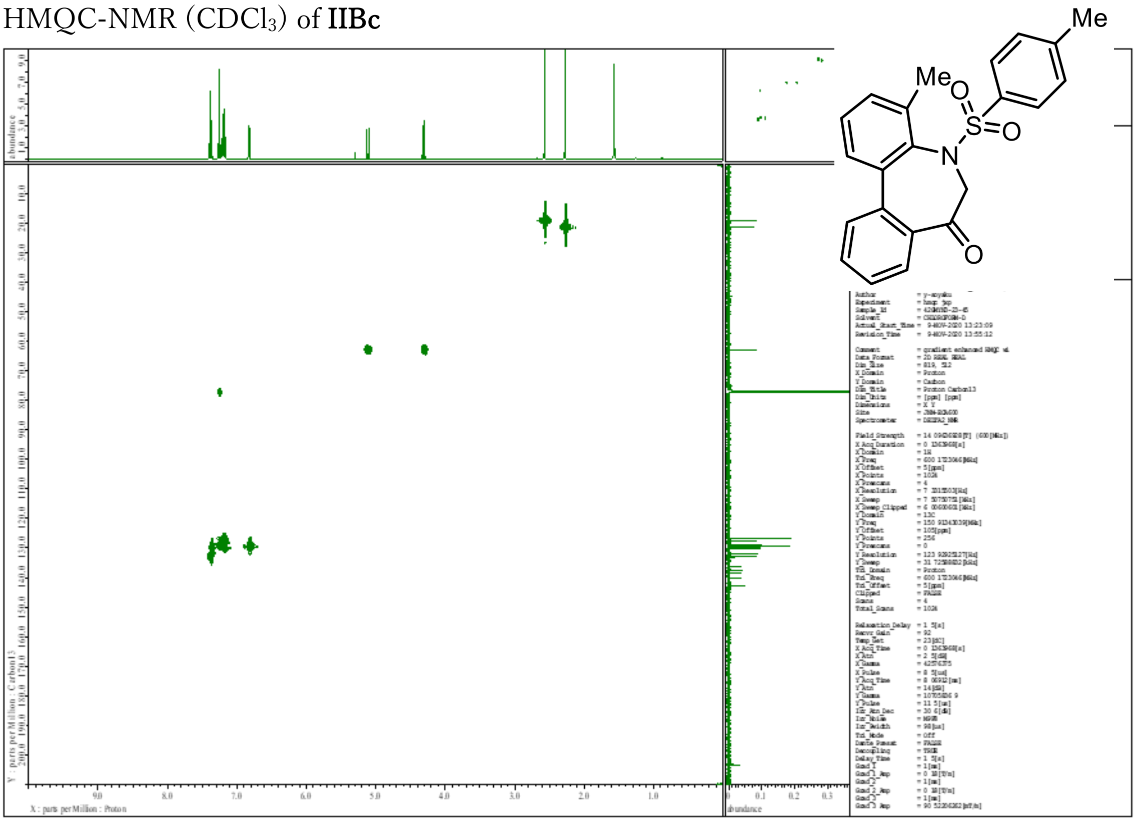

^{1}H-NMR (600 MHz, $CDCl_3$) of **IIBd**

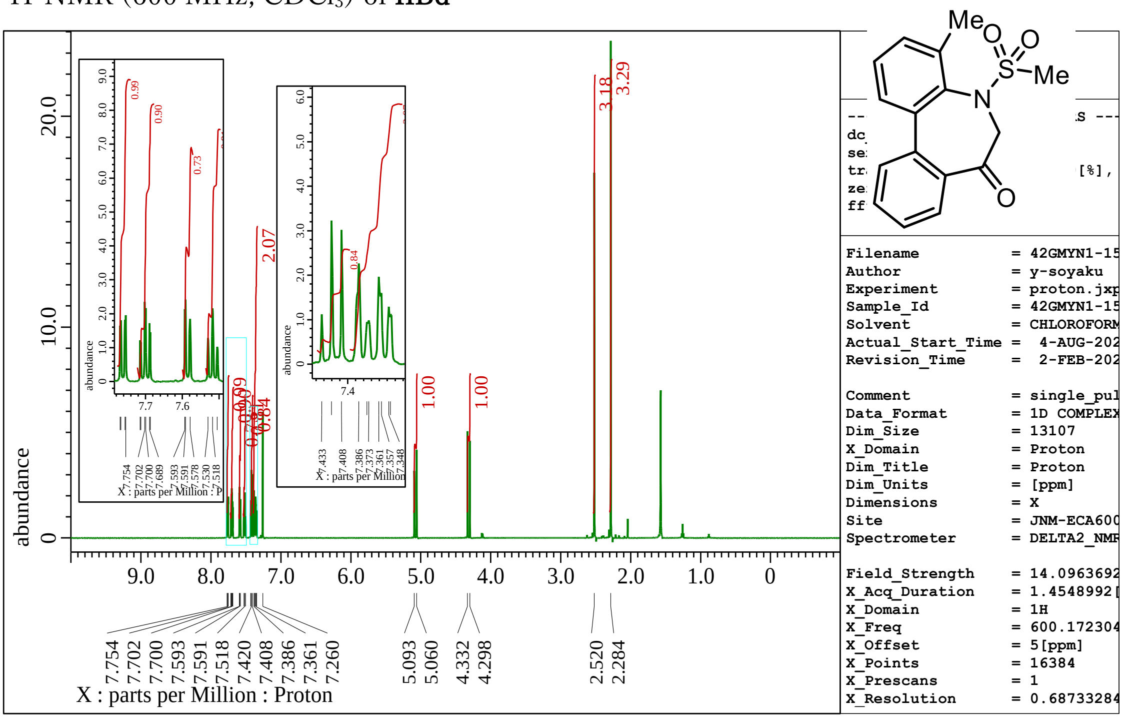

^{13}C{^{1}H }-NMR (150 MHz, $CDCl_3$) of **IIBd**

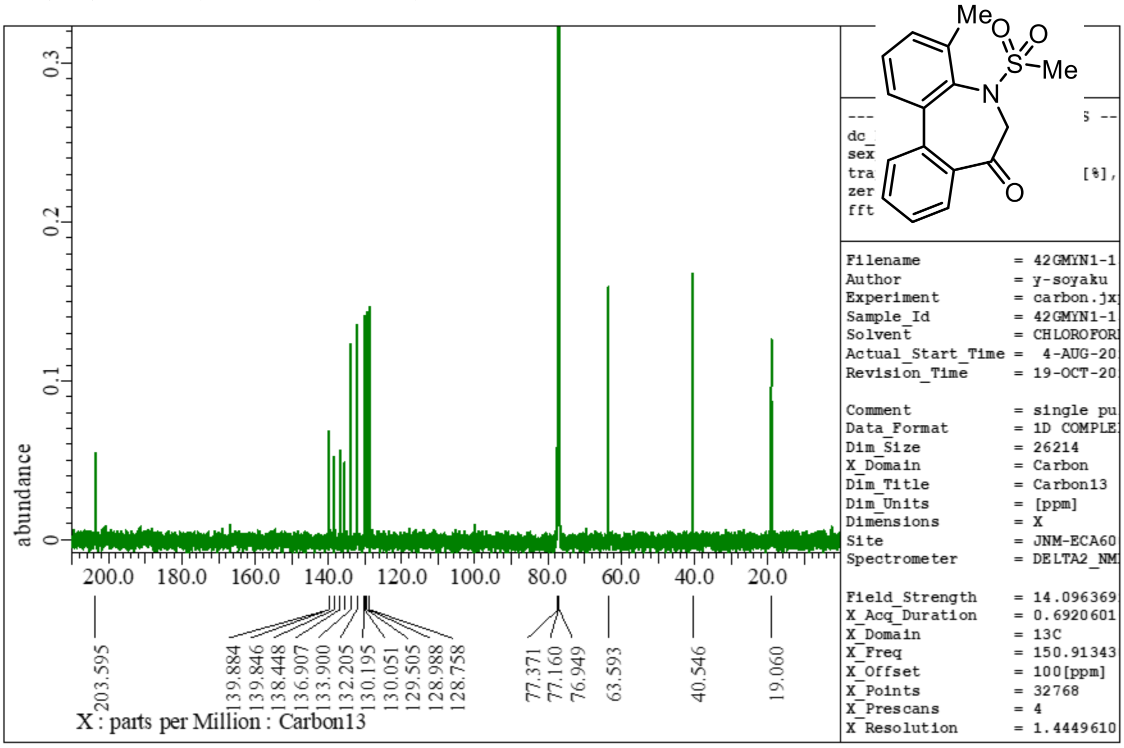

H-H COSY-NMR (600 MHz, $CDCl_3$) of **IIBd**

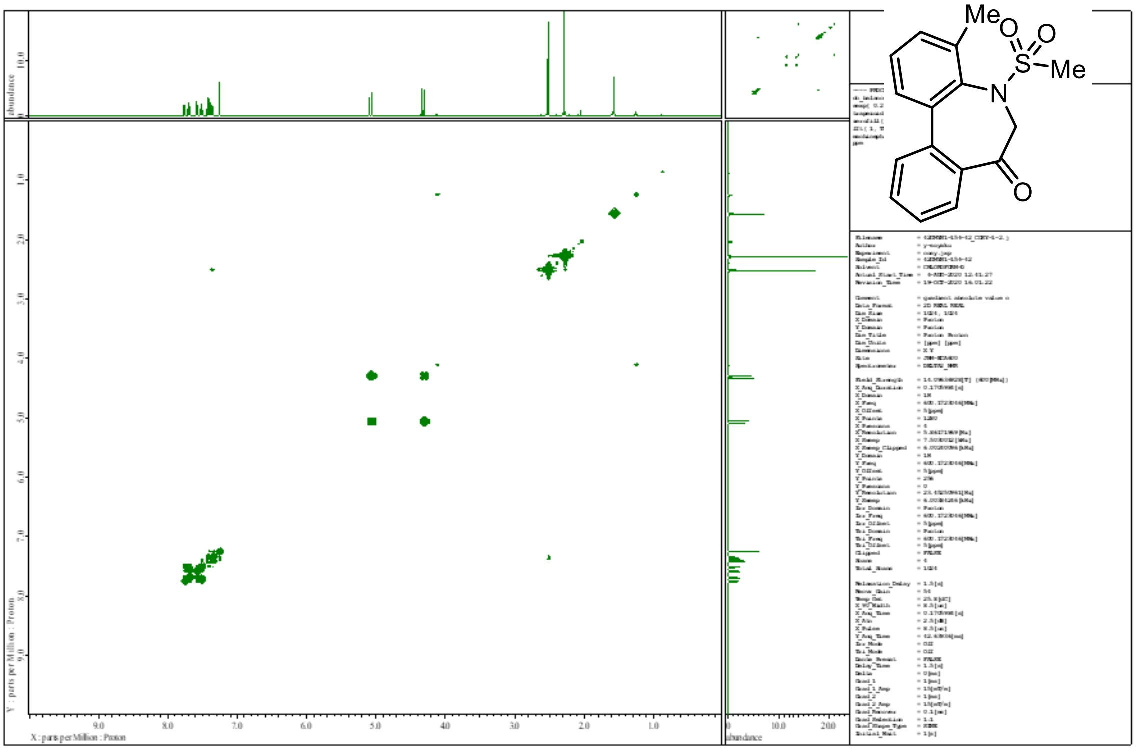

HMQC-NMR ($CDCl_3$) of **IIBd**

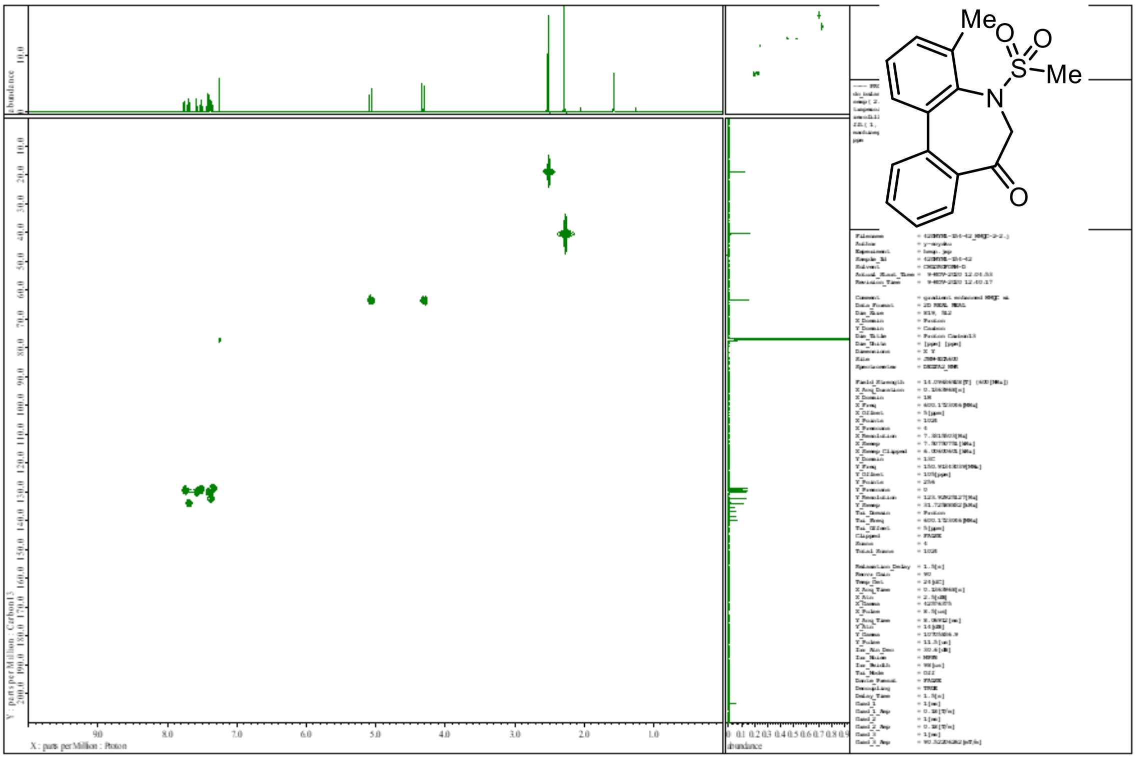

^{1}H-NMR (600 MHz, $CDCl_3$) of **IIBe**

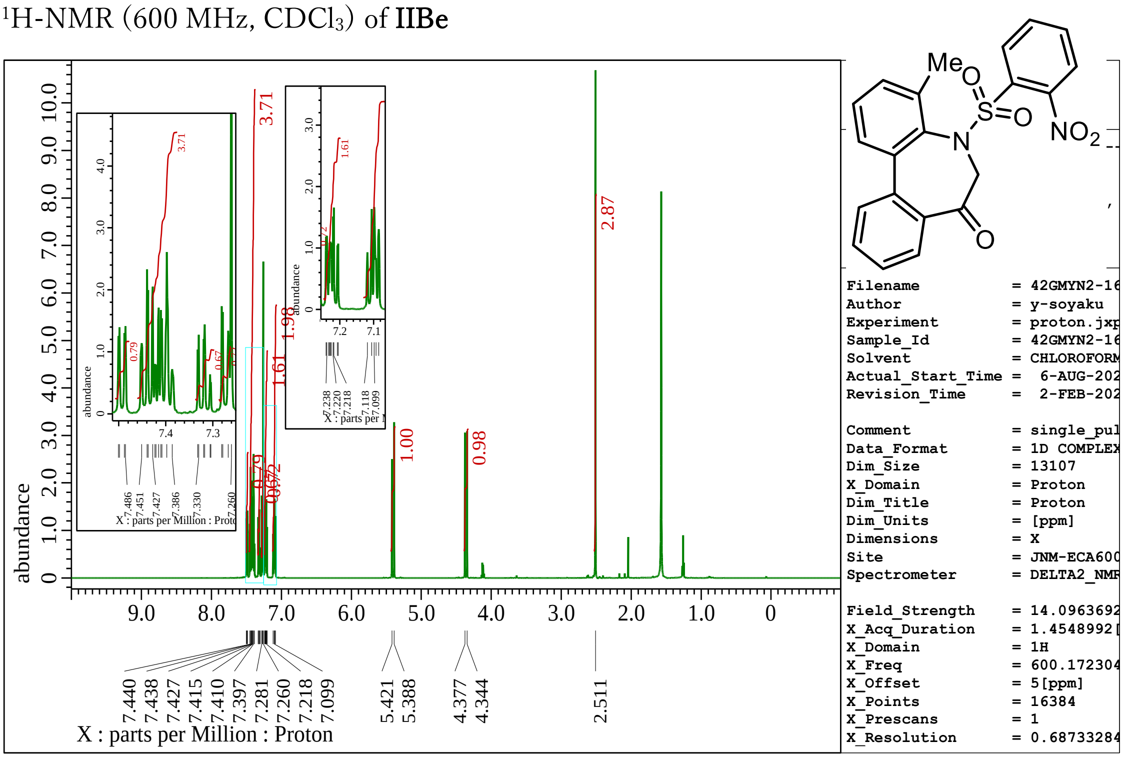

^{13}C{^{1}H }-NMR (150 MHz, $CDCl_3$) of **IIBe**

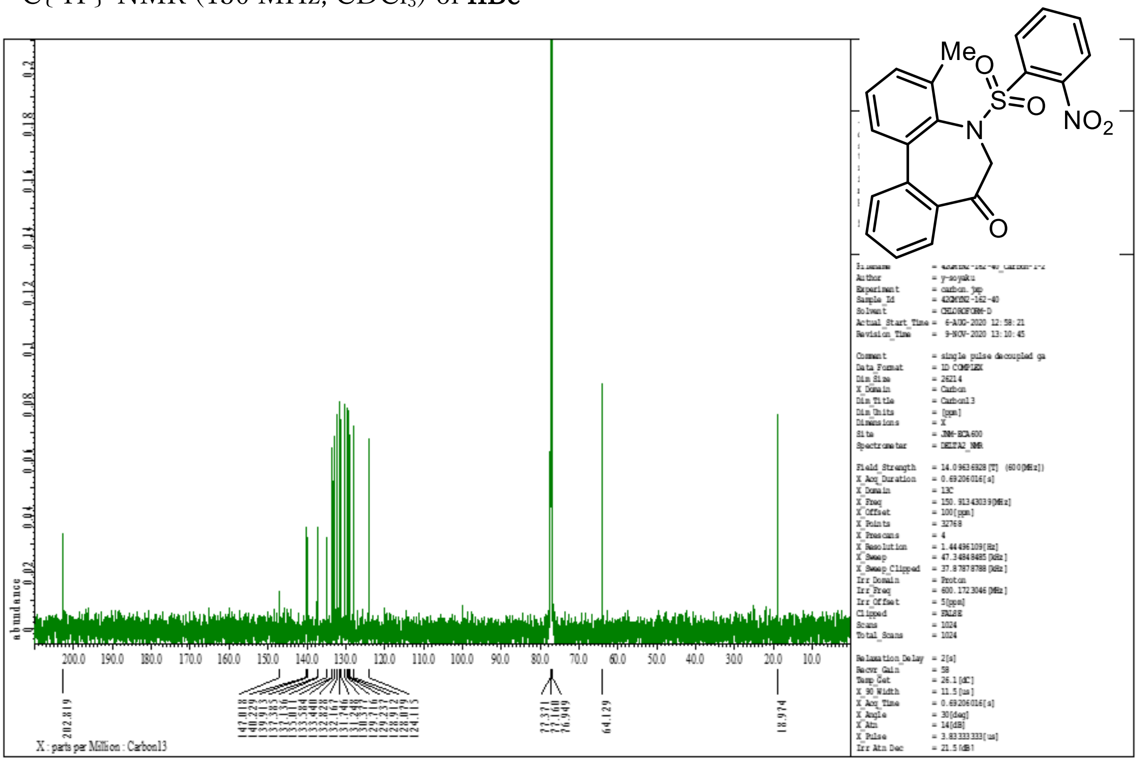

H-H COSY-NMR (600 MHz, $CDCl_3$) of **IIBe**

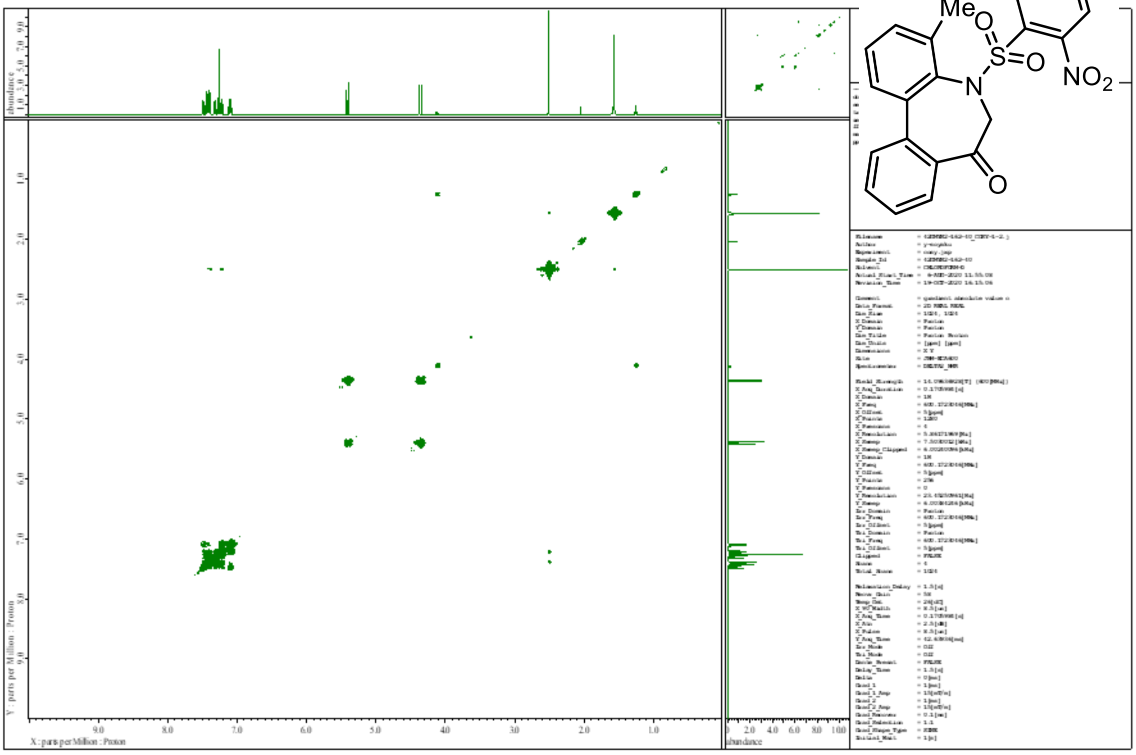

HMQC-NMR ($CDCl_3$) of **IIBe**

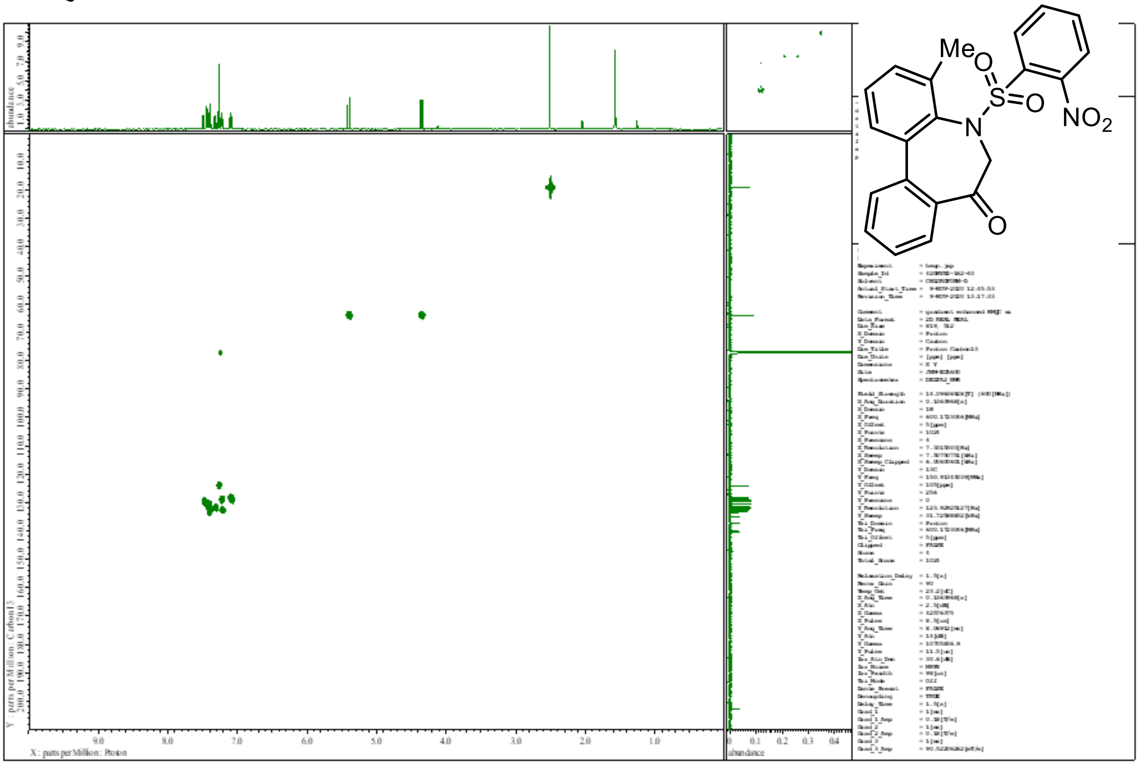

^{1}H-NMR (600 MHz, $CDCl_3$) of **IIBf**

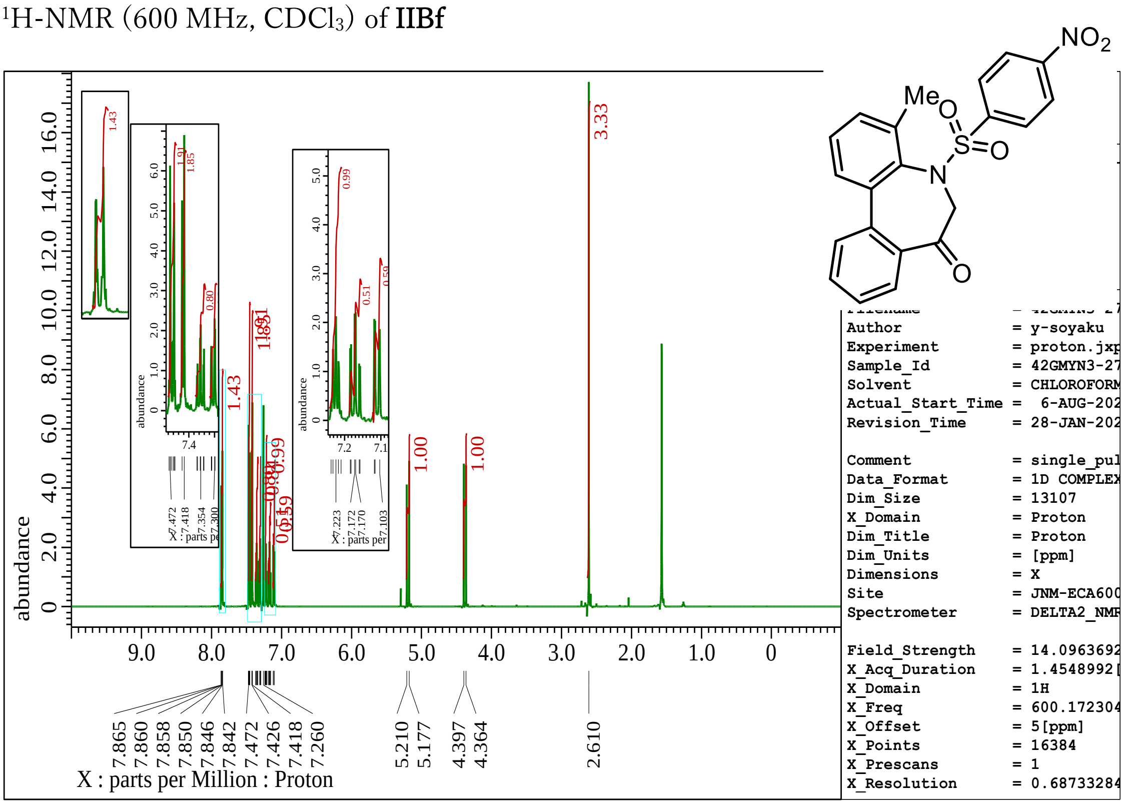

^{13}C{^{1}H }-NMR (150 MHz, $CDCl_3$) of **IIBf**

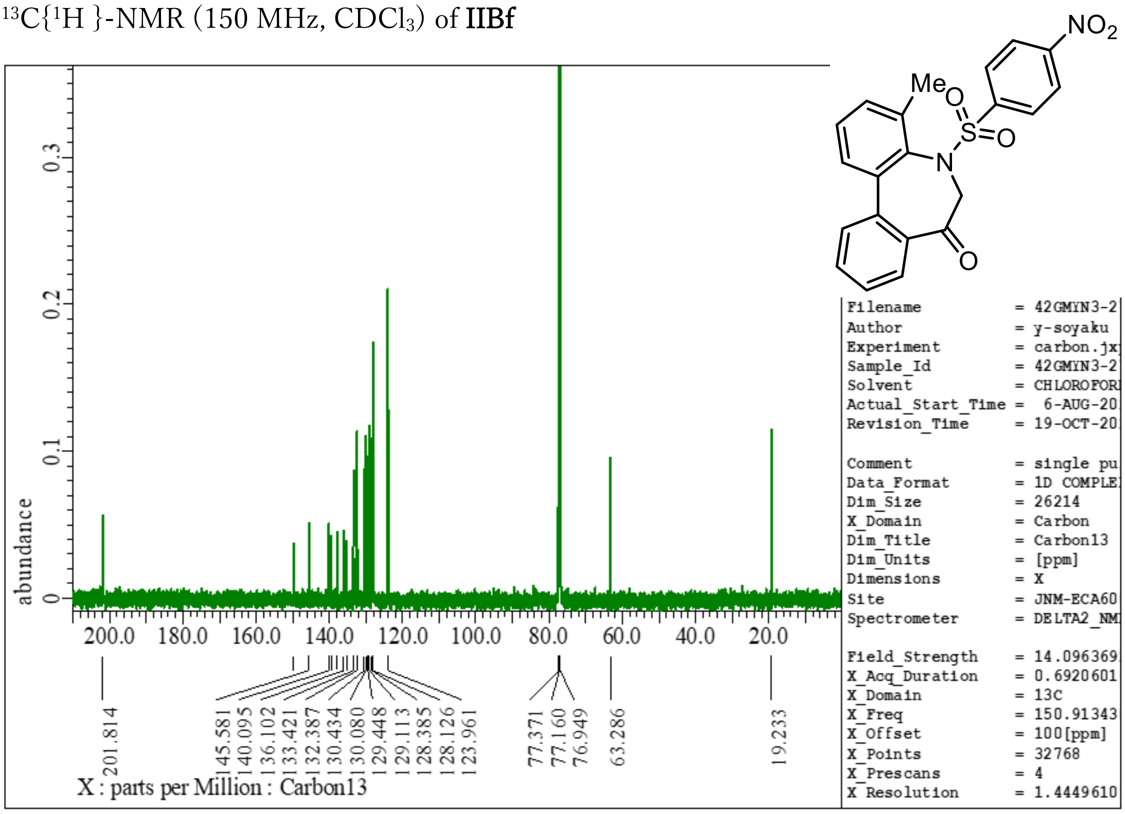

H-H COSY-NMR (600 MHz, $CDCl_3$) of **IIBf**

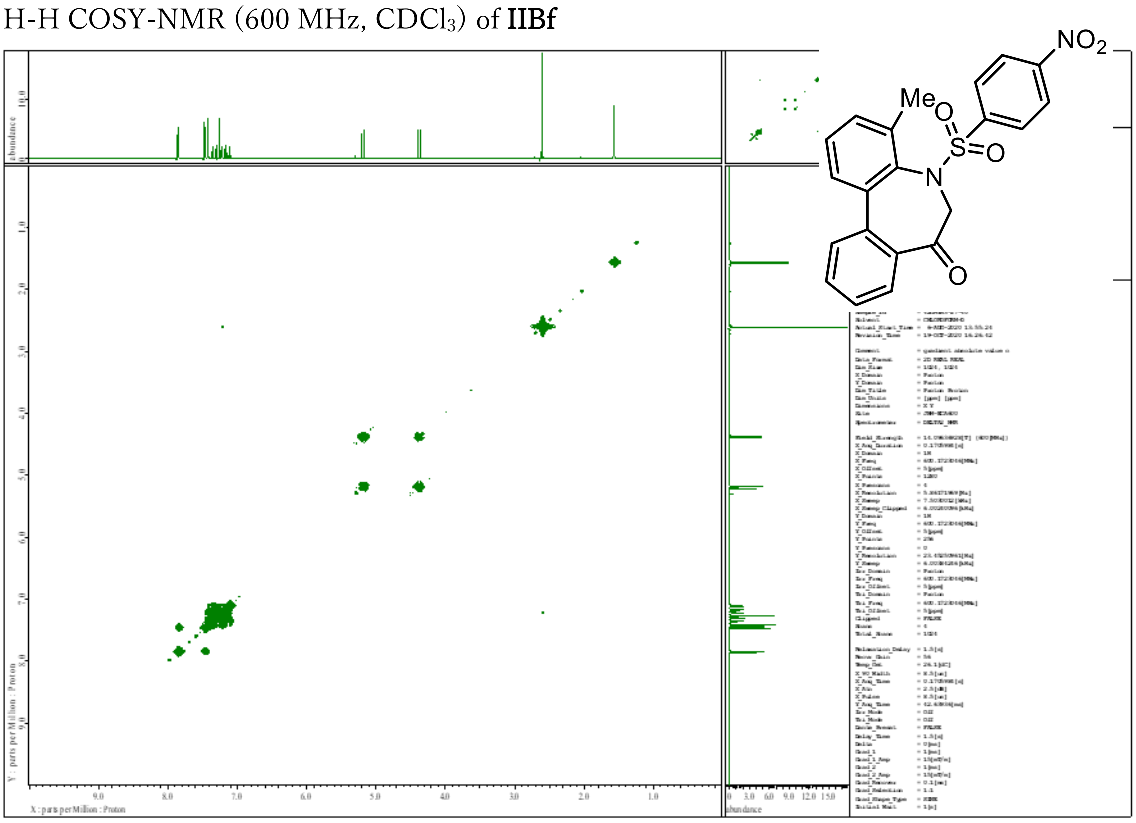

HMQC-NMR ($CDCl_3$) of **IIBf**

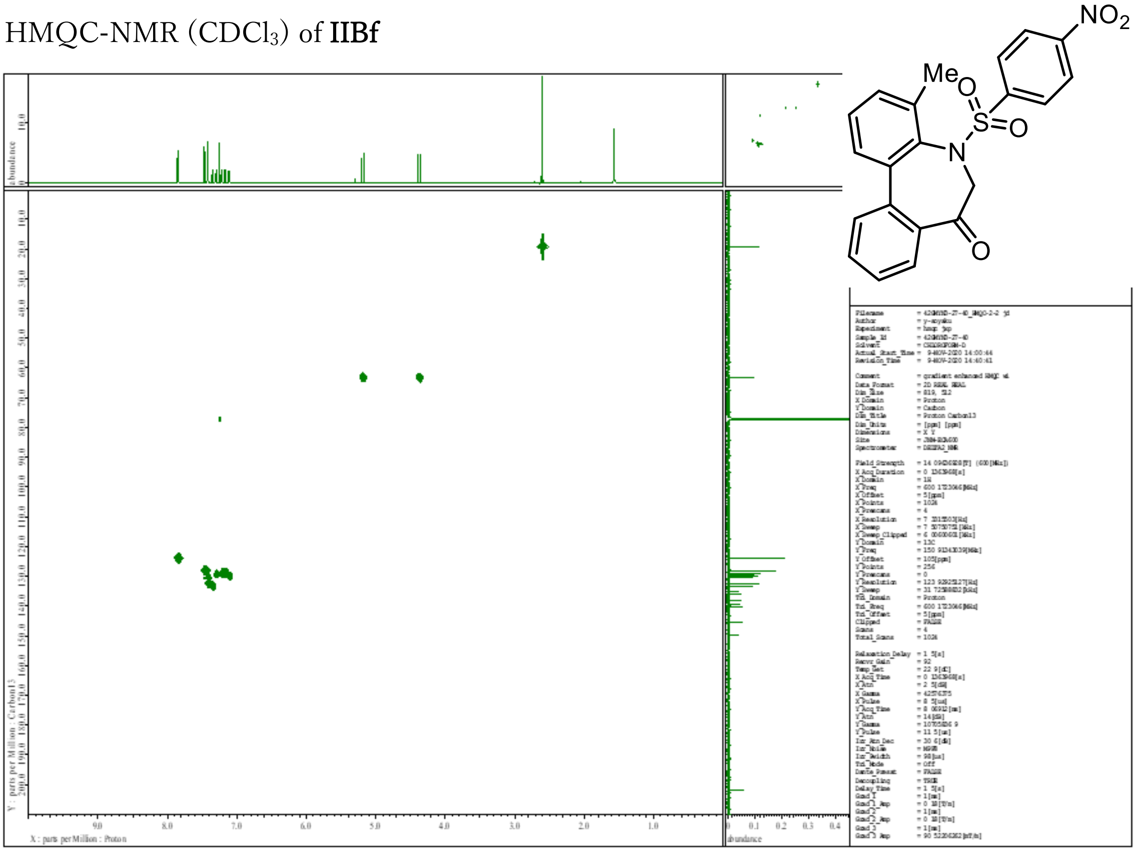

Supplement: Supplementary file 1 — jo1c00594_si_001.pdf [file jo1c00594_si_001.pdf]
